# Establishment of an *in vitro* model of cultured viable human, porcine and canine skin and comparison of different media supplements

**DOI:** 10.7717/peerj.7811

**Published:** 2019-10-03

**Authors:** Isa Bauhammer, Manuel Sacha, Eleonore Haltner

**Affiliations:** Across Barriers GmbH, Saarbrücken, Germany

**Keywords:** *In vitro*, Human skin model, Animal skin model, Skin viability, Medium supplements

## Abstract

Transdermal drug delivery provides several advantages over conventional drug administration, such as the avoidance of first-pass metabolism and better patient compliance. * In vitro* research can abbreviate and facilitate the pharmaceutical development considerably compared to * in vivo* research as drug screening and clinical studies can be reduced. These advantages led to the development of corresponding skin models. Viable skin models are more useful than non-viable ones, due to the influence of skin metabolism on the results. While most * in vitro* studies concentrate on evaluating human-based models, the current study is designed for the investigation of both human and animal diseases. So far, there is little information available in the literature about viable animal skin cultures which are in fact intended for application in the veterinary and not the human field. Hence, the current study aims to fill the gap. For the * in vitro* viable skin model, specimens of human, porcine and canine skin were cultured over two weeks under serum-free conditions. To evaluate the influence of medium supplementation on skin viability, two different supplement mixtures were compared with basic medium. The skin specimens were maintained at a viability-level >50% until the end of the study. From the tested supplements, the addition of bovine pituitary extract and epidermal growth factor increased skin viability whereas hydrocortisone and insulin induced a decrease. This * in vitro* viable skin model may be a useful tool for the investigation of skin diseases, especially for the veterinary field.

## Introduction

Transdermal drug delivery (TDD), compared to oral and parenteral drug administration, offers several advantages such as a decreased risk for toxicity and adverse effects, avoidance of first-pass metabolism and better patient compliance (Lee et al., 2018; Prausnitz, Mitragotri & Langer, 2004). In order to facilitate and abbreviate the pharmaceutical development process, an increasing trend to the transfer from *in vivo* to *in vitro* research has been observed during the last few decades (Kim, Stein & O’Hare, 2004; Tsang & Bhatia, 2004). Simultaneously, the social acceptance for animal testing has remarkably decreased (Bekoff, 2009; Kolar, 2006; PETA UK, 0000), resulting in corresponding changes in legislation. Examples include the 3R-principle in 1959, the European Centre for the Validation of Alternative Methods (ECVAM) established in 1991, the “Declaration of Bologna” in 1999 and the enactment of Registration, Evaluation and Authorization of Chemicals (REACH) in 2007 (Erkekoğlu, Giray & Basaran, 2011). Consequently, *in vitro* research is being promoted as future standard, requiring the establishment of suitable models. The evaluation of percutaneous absorption (skin permeation) is essential in the design and development of various drug formulations. Only if a drug is able to overcome the outermost layer of the skin, the stratum corneum (SC), which constitutes the major part of skin barrier, can the drug be considered as a promising candidate for TDD. Whereas the SC poses a stronger barrier for hydrophilic substances, the permeation of lipophilic substances is limited by viable epidermis and dermis (Nitsche Johannes & Kasting Gerald, 2013; Yamaguchi et al., 2008). However, the assessment of permeation is just one aspect of TDD and mainly useful for substance screening and defining rank orders to categorize drugs in different permeability classes. For the investigation of complex issues such as skin inflammation and disease, a more comprehensive approach has to be chosen. With chronic inflammatory and infectious skin diseases being a continuous problem (Fadok, 2014; Mohammedamin et al., 2006; Thomsen, 2014) and decreasing efficacy of antibiotic treatment due to bacterial resistances (Canavan, Chen & Elewski, 2016; Cunliffe, 2002; Jesitus, 2017; Oberemok & Shalita, 2002), new therapeutic options are imperative. The popular view of the SC as the only factor influencing skin absorption is not tenable anymore due to the detection of considerable metabolic capabilities of living skin (Ahmad & Mukhtar, 2004; Haberland et al., 2006; Hikima, Tojo & Maibach, 2005). Hence, viable skin is preferable for studying complex skin conditions (Mathes, Ruffner & Graf-Hausner, 2014). One factor among others, affecting transdermal drug delivery, is skin resistance. A higher level of skin resistance is associated with skin integrity and an intact barrier (Rachakonda et al., 2008), influencing methods such as iontophoresis (Roustit, Blaise & Cracowski, 2014). This method employs a non-invasive electric current at low voltages for the delivery of ionisable drugs into the skin (Shimizu & Krištof, 2017).

Further skin properties need to be considered for TDD. Skin thickness varies considerably between different body regions (Abd et al., 2016; Flaten et al., 2015; Kanitakis, 2002; Wickett & Visscher, 2006). Human skin, e.g., is especially thin at the eyelids or directly behind the ears while on palms and soles it is very thick (Kanitakis, 2002; Wickett & Visscher, 2006). Intensity of blood flow (*in vivo* or perfused models) to different skin locations proved to be an important parameter as well as functional properties, i.e., resistance toward stress or strain and the amount of elastic fibers (Hao et al., 2016; Kamboj, 2013; Kanitakis, 2002; Tagami, 2008). General metabolic activity of the skin and amounts of metabolic enzymes are also variable but tend to increase along with blood supply (Davis et al., 2006). Fluctuations of the density of hair follicles between body regions but even more between different species (e.g., human vs. sheep) have to be taken into account as well as variations in lipid content and composition (Flaten et al., 2015; Mills & Cross, 2006). All the aforementioned skin properties are also subject to considerable variation between human individuals depending on sex, body weight, age and ethnicity with variations up to 66 % (Abd et al., 2016; Flaten et al., 2015). For this study, viable skin was chosen due to better predictability of *in vivo* conditions (Abd et al., 2016) and potential metabolic activity although no exact determination of the metabolic capacities was intended. If the skin has to be maintained viable over a prolonged time, the usage of an appropriate viability marker is crucial.

However, most of them are designed for cell culture and not for tissue, such as the standard assays MTT (3-(4,5-dimethylthiazol-2(yl)-2,5-diphenyltetrazolium bromide) and WST (water soluble tetrazolium salts). Furthermore, they are destructive to the tissue and often very time-consuming. (Bopp & Lettieri, 2008; Stoddart, 2011) The finally selected LDH (lactate dehydrogenase) release assay, however, is shorter, easy to use and non-destructive. Therefore, it can be used for repeated measurements with the same skin specimen which is crucial for this study (Roche, 2016). As this assay is also in principle designed for cell culture, a modified version, adapted to tissue (Bauhammer, Sacha & Haltner, 2019a) and validated according to FDA (Food and Drug Administration, USA) guidelines (Bauhammer, Sacha & Haltner, 2019b), was used in the current study.

In most if not all studies involving *in vitro* skin models, the focus has been on human research, with animal skin models as mere approximations of the properties of human skin, e.g., such as the pig ear model (Flaten et al., 2015; Mathes, Ruffner & Graf-Hausner, 2014). Therefore, although human skin was employed as well, this study aims to benefit the veterinary field by adding valuable information about *in vitro* cultured skin models with viable animal skin which can scarcely be found in the literature (Serra et al., 2007).

Hence, a long-term cultured *in vitro* viable skin model from human, porcine and canine skin was established under simplest and serum-free conditions. Here, the aim was not human research but to fill the empty space in the literature regarding *ex vivo* cultured animal skin models and bridge the gap between human centered and animal centered research and medicine. Due to availability restrictions, the planned number of species for this study (including feline, equine and bovine skin) was not achieved. Therefore, porcine skin was not only selected for comparison to human skin, but also to represent the group of livestock animals. Canine skin represented the group of companion animals but was also chosen because of a known predisposition of dogs for skin diseases (Fadok, 2014). Several criteria for selection of the particular skin donors applied (same sex and age group, normal body weight). Only full-thickness trunk skin was considered. The selected body regions were determined by the human skin donor since only abdominal skin was available. Abdominal skin, however, was avoided for the canine donor due to mammary complexes and scar tissue following mastectomy. Back skin was also avoided due to pigmentation and higher amount of hair follicles. Therefore, skin from the flank/lateral abdominal region was taken. Back skin was chosen for the porcine skin specimen based on the results of Khiao In et al. (2019) who compared histological and functional properties of porcine skin from different anatomical regions with human abdominal skin. According to these results, both back and flank skin are most similar to human abdominal skin. However, due to skin injuries in the flank region of the pig, back skin was deemed as better option in this case.

The absence of nutrients provided by serum had to be compensated by adding other supplements to achieve better viability results. Therefore, the influence of supplementation with insulin, hydrocortisone, human epidermal growth factor (hEGF) and bovine pituitary extract (BPE) was evaluated. These four supplements were selected as they are known to have beneficial effects on skin and skin cells (Hu et al., 2013; Li, Fukunaga-Kalabis & Herlyn, 2011; Oku et al., 1994; Xu et al., 2013) which is why they are included in media for keratinocyte growth and proliferation (e.g., Keratinocyte growth medium 2 from Promocell). The selection of these supplements was hence based on findings in the literature where their effects were described separately and in combination (Li, Fukunaga-Kalabis & Herlyn, 2011; Stark et al., 1999). Based on the literature findings and due to practical reasons, insulin and hydrocortisone were evaluated as one supplement mixture and EGF plus BPE as another.

## Materials & Methods

### Skin preparation and cultivation

**Figure 1:**
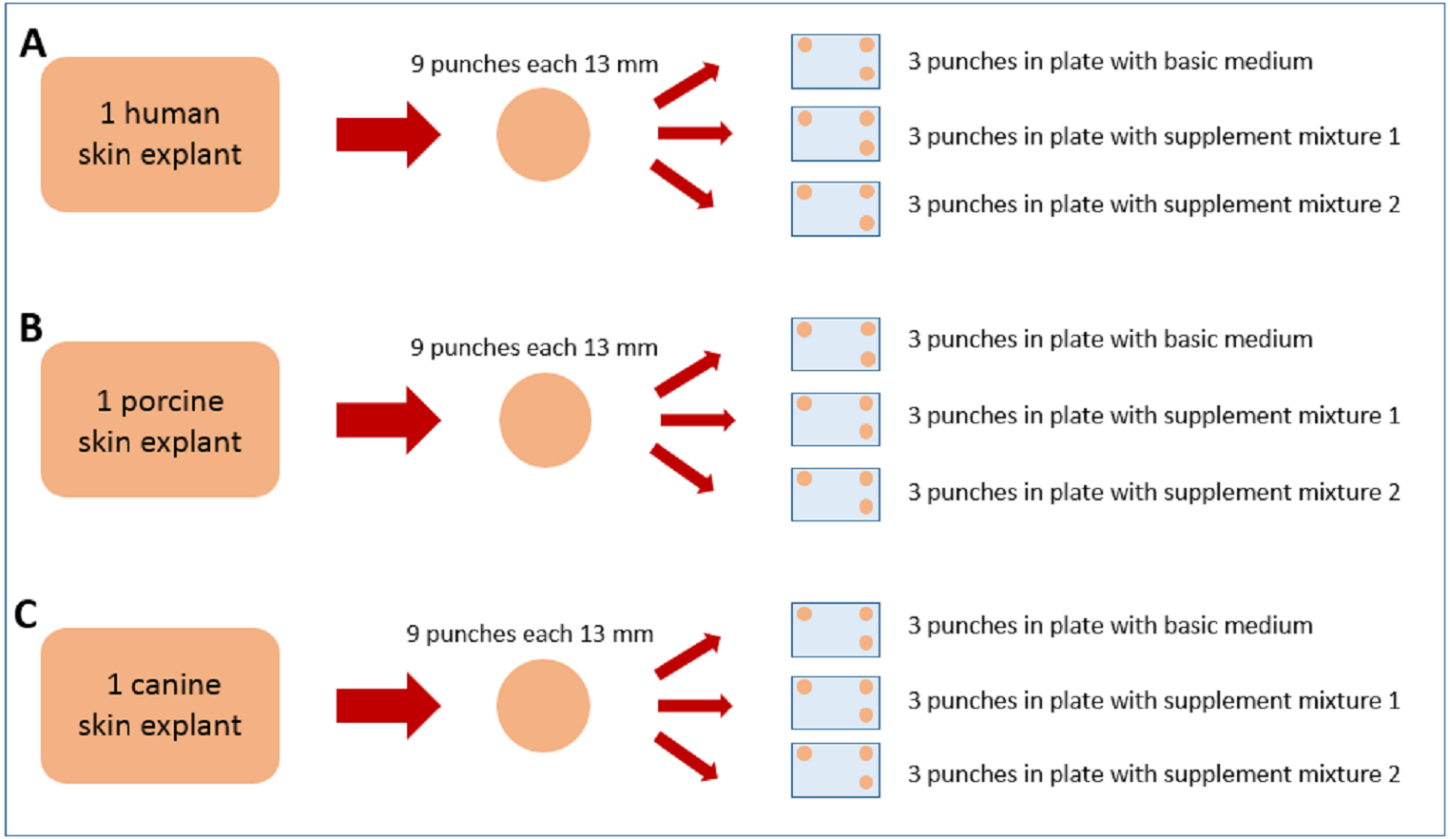
Graphical illustration of the sample distribution for cultivation. From the human (A) skin specimen, 9 skin punches were made and were distributed into three 12-well plates, three punches per plate. Each of the three plates was filled up with either basic medium, supplement mixture 1 or supplement mixture 2. The porcine (B) and canine (C) skin explants were treated accordingly.

Human abdominal human skin (53 y/o, female, Caucasian) was obtained after informed consent of the patient according to the Declaration of Helsinki from esthetic surgery (Cabinet Dr. Pierre Sibille, Luxembourg, Luxemburg), porcine back skin (ca. 6 months old, female, Deutsche Landrasse) from a slaughterhouse (Schwamm und Cie, Saarbrücken) and canine flank/lateral abdominal skin (11 y/o, female, middle-sized mongrel) from a local veterinary practitioner (Dr. Norbert Paulus, Saarbrücken) after the dog’s euthanasia with informed owner consent. All skin specimens were freshly excised and stored/transported for less than 2 h at 4 °C and high humidity to maintain their viability as high as possible. The subcutaneous fat was carefully removed with a scalpel, the tissue surface was rinsed with water and then gently dried. From each of the three skin specimens, nine full skin punches of 13 mm diameter were taken and weighed. They were cleaned under sterile conditions (sterile bench laminar airflow, Heto-Holten GmbH, Wettenberg, Germany) with a mixture of phosphate buffered saline (PBS) solution (Merck Millipore, Darmstadt, Germany) and 70% ethanol (Waldeck GmbH & Co KG, Münster, Germany) and placed dermal side down in 12-well plates (Costar 3513 12-well plates from Corning Life Sciences, Kaiserslautern, Germany). From the nine punches per species, three skin punches were placed together in one plate. Three plates per species were cultivated using three different media, so that in total 27 skin punches have been cultivated as shown in Fig. 1.

One plate from each species was filled up to 0.7 mL per well with Dulbecco’s modified Eagle’s medium solution (Sigma Aldrich Chemie GmbH, Taufkirchen, Germany), containing 3.7 g/L sodium bicarbonate (Sigma Aldrich Chemie GmbH, Taufkirchen, Germany) and glucose (VWR International GmbH, Darmstadt, Germany). 75.5 mg/L gentamycin sulfate (Merck Millipore, Darmstadt, Germany) were added from a stock solution of 15 g/L gentamycin sulfateThis solution is further referred to as basic medium. Another plate was filled up with the same DMEM solution, but with an added supplementation of insulin and hydrocortisone, each two mL/L of DMEM (KGM single quots; Lonza, Walkersville, MD, USA), further referred to as supplement mixture 1. The third plate was filled up with DMEM solution and supplementation of bovine pituitary extract (BPE) eight mL/L and human epidermal growth factor (hEGF) (KGM single quots; Lonza, Walkersville, MD, USA), two mL/L of DMEM, further referred to as supplement mixture 2 (see Fig. 1).

The supplements were originally designed for keratinocyte growth medium. For the cultured skin model, a higher need of nutrients for full skin compared to cell monolayers was assumed, hence the recommended concentrations for cell culture were doubled, based on some ranges given in the literature (Li, Fukunaga-Kalabis & Herlyn, 2011; Stark et al., 1999). These concentrations may be optimized for receiving best results in future experiments but serve as working standard in this study. The exact concentration of the supplements themselves could not be determined as no further information was provided by the supplier (KGM single quots: CC-4002E BPE, CC-4015E hEGF, CC-4021E insulin, CC-4031E hydrocortisone).

**Figure 2:**
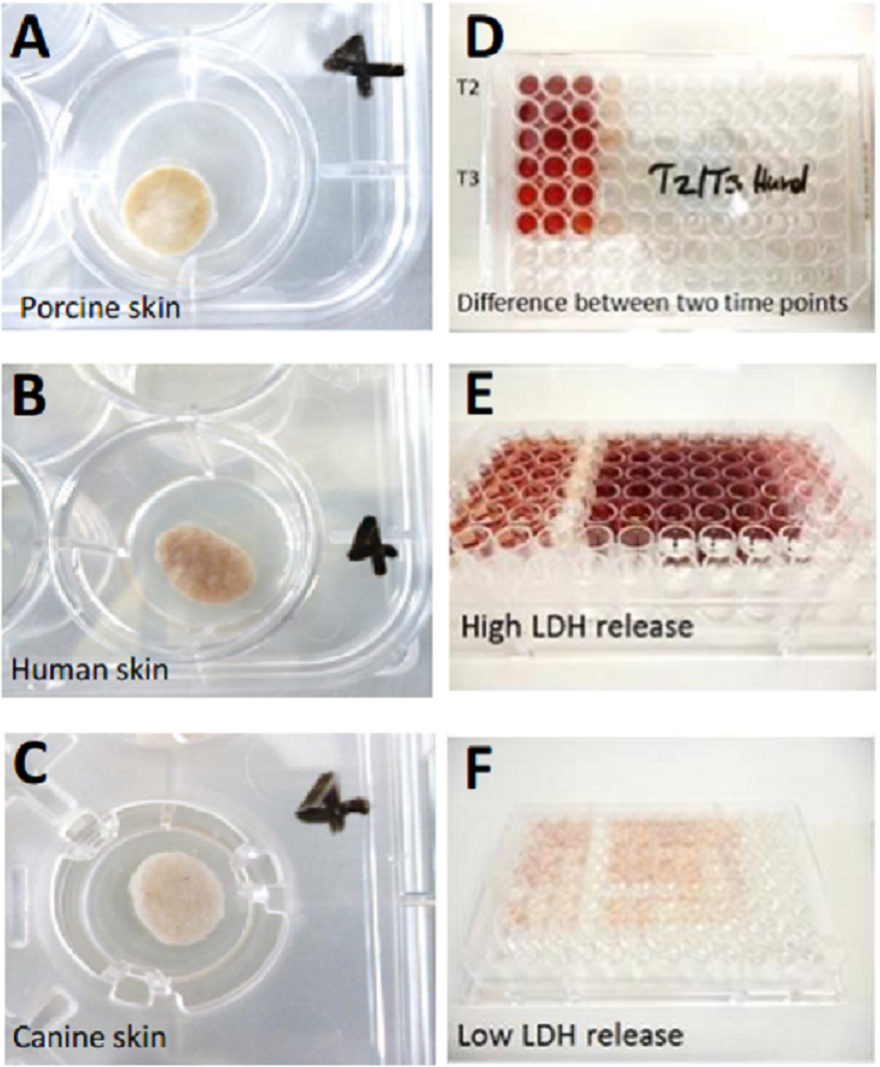
Illustration of skin cultivation samples and LDH measurement. (A) Porcine skin punch on cultivation day 7, well 4. (B) Human skin punch on cultivation day 7, well 4. (C) canine skin punch on cultivation day 7, well 4. (D) Exemplary LDH measurement to demonstrate the difference in LDH content between two timepoints (T2 and 3) which is shown in the slight color change from T2 (upper nine wells) to T3 (lower nine wells). (E) Exemplary LDH measurement displaying high amounts of LDH (dark red). (F) Exemplary LDH measurement displaying low amounts of LDH (light red/pink). The LDH measurements in panels D-F do not correlate with the skin punches in A–C.

The SC (stratum corneum) of every skin punch remained uncovered with medium at the air-liquid interface. The skin explants were then cultivated over 14 days with daily sampling and medium change in a sterile incubator (Heracell Incubator; Heraeus, Hanau, Germany) at 37 °C in 5 % CO_2_/air to keep the pH of the medium at 7.4 (Schmitz, 2011) which is not only important for the cultured skin but also for the enzymatic reaction employed for measurement (Bisswanger, 2014). Photographs of the plates were taken every other day to monitor the medium levels and changes in the skin’s appearance (see Fig. 2).

105 µL polyethylene glycol (PEG) 400 were added to the daily collected samples from medium supernatant containing LDH before storage at −20 °C for up to 4 weeks until measurement. These conditions were chosen according to the results of a previous stability study (Bauhammer, Sacha & Haltner, 2019b).

### Controls

In order to quantify the highest possible LDH release from the skin of each species, a positive control was established for which a piece of skin was weighed, added to a defined amount of DMEM and mechanically destroyed with an immersion blender. After skin destruction the suspension was filtered through a nylon net, the filtrate was centrifuged and the supernatant was aliquoted and stored at −20 °C for measurement. Measurement result of every positive control sample was considered as 100% of the total LDH amount of this skin specimen. A negative control was obtained by heating one aliquot of positive control to 65  °C for 30 min (Bisswanger, 2014) to denaturize all the contained LDH. The negative controls serve as blank sample for positive controls, i.e., the measured absorption of the negative control was subtracted from the absorption of positive controls to remove the influence of turbidity of the medium due to skin matrix. From the absorbance values of the positive control, the LDH activity per gram skin relative to those of the control was determined in order to calculate the viability of the skin in percent. Fifty percent were set as limit, and lower values were considered as indicators of a non-viable skin specimen.

### Measurement by plate reader

Photometric measurement of the obtained medium samples for the quantification of LDH release into the medium was conducted with a plate reader (microplate reader Wallac 1420 Victor 2) using an absorbance wavelength of 450 nm (see Fig. 2). The generally cell-based LDH release assay was modified and validated for its usage on tissue beforehand (Bauhammer, Sacha & Haltner, 2019b).

For measurement, the samples were thawed at room temperature, shaken, diluted with DMEM into the linear range of the LDH calibration curve and pipetted with piston-stroke pipettes (Eppendorf GmbH, Wesseling, Germany) into a generic 96-well plate (here Nunclon Delta Surface; Thermo Fisher Scientific, Karlsruhe, Germany). Tubes from Eppendorf and Corning (Corning Life Sciences, Kaiserslautern, Germany) were used for sampling and measurement preparation. All the cultivation samples were measured in duplicate, except the control samples which were measured in triplicate. Reported values are mean values if not specified otherwise.

All calculations were performed using Microsoft Excel 2010.

### Data analysis

Statistical evaluation was conducted using statistics software OriginPro 9.0 (Additive GmbH, Friedrichsdorf, Germany). Non-parametric analysis such as Kruskal-Wallis ANOVA (KWA), NPH k independent samples test (NPH) combining Kruskal-Wallis ANOVA and Mood’s Median Test (MM) has been employed. A *p*-value of <0.05 was considered significant. The exact *p*-value and degrees of freedom (DF) are reported.

## Results

**Figure 3:**
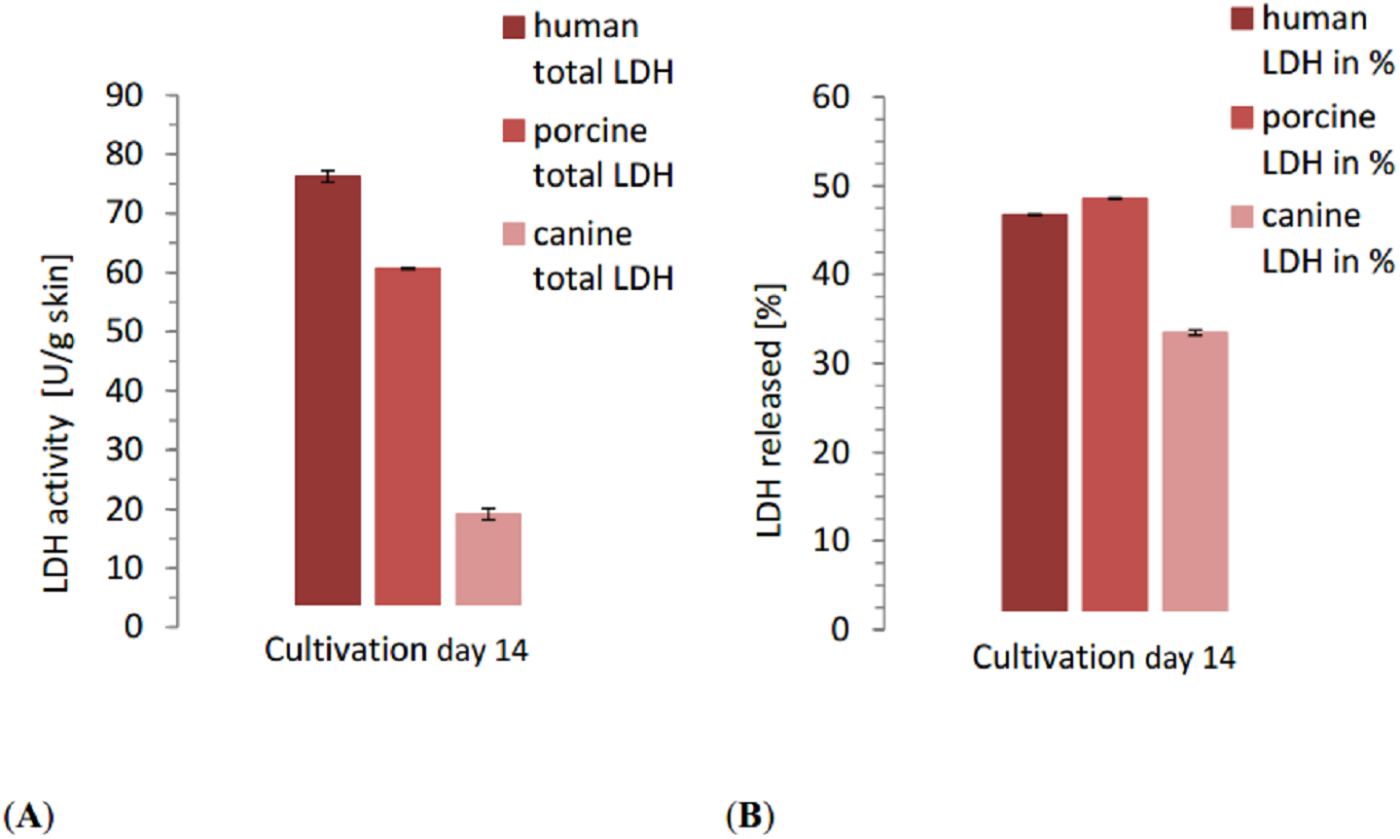
Comparison of the total released LDH of each species at the end of the study with the relative LDH release as percentage of the positive control of each species. (A) Total amount of LDH released by all the human, porcine and canine skin samples in catalytic units per gram skin until end of cultivation (day 14). The columns are calculated from the cumulated sum (e.g., sum of LDH release of all human samples at timepoint d0+d1+d2+...d14; same for porcine and canine samples) of all the means from each species, respectively. (B) Amount of LDH released by all the human, porcine and canine skin samples as percentage of the corresponding positive control. Same principle for calculation applies as for A.

The LDH content of the positive control samples showed considerable variation between species. The human control reached 163.1 U/g skin, porcine control 124.8 U/g skin and canine control 57.3 U/g skin. The total LDH release over the whole cultivation time reflected the differences seen in control samples. With 76.19 ±0.03 U/g skin, LDH release of human skin was the highest in comparison to the other species (Fig. 3) with significant differences between the three species (NPH: KWA *p* = 0.02732 DF 2, MM *p* = 0.04285 DF 2). Relative to the positive control, however, human skin was with 46.72% release in the middle, slightly lower than porcine skin. The total release of porcine skin was 60.66 ±0.09 U/g skin while the relative release was the highest one with 48.60%. Canine skin displayed a total release of 19.17 ±0.3 U/g skin, considerably lower than the other ones. Also the relative release of canine skin was 33.47%, and thus lower than human and porcine samples with significant differences between the three species (NPH: KWA *p* = 0.00179 DF 2, MM *p* = 0.02732 DF 2).

**Figure 4:**
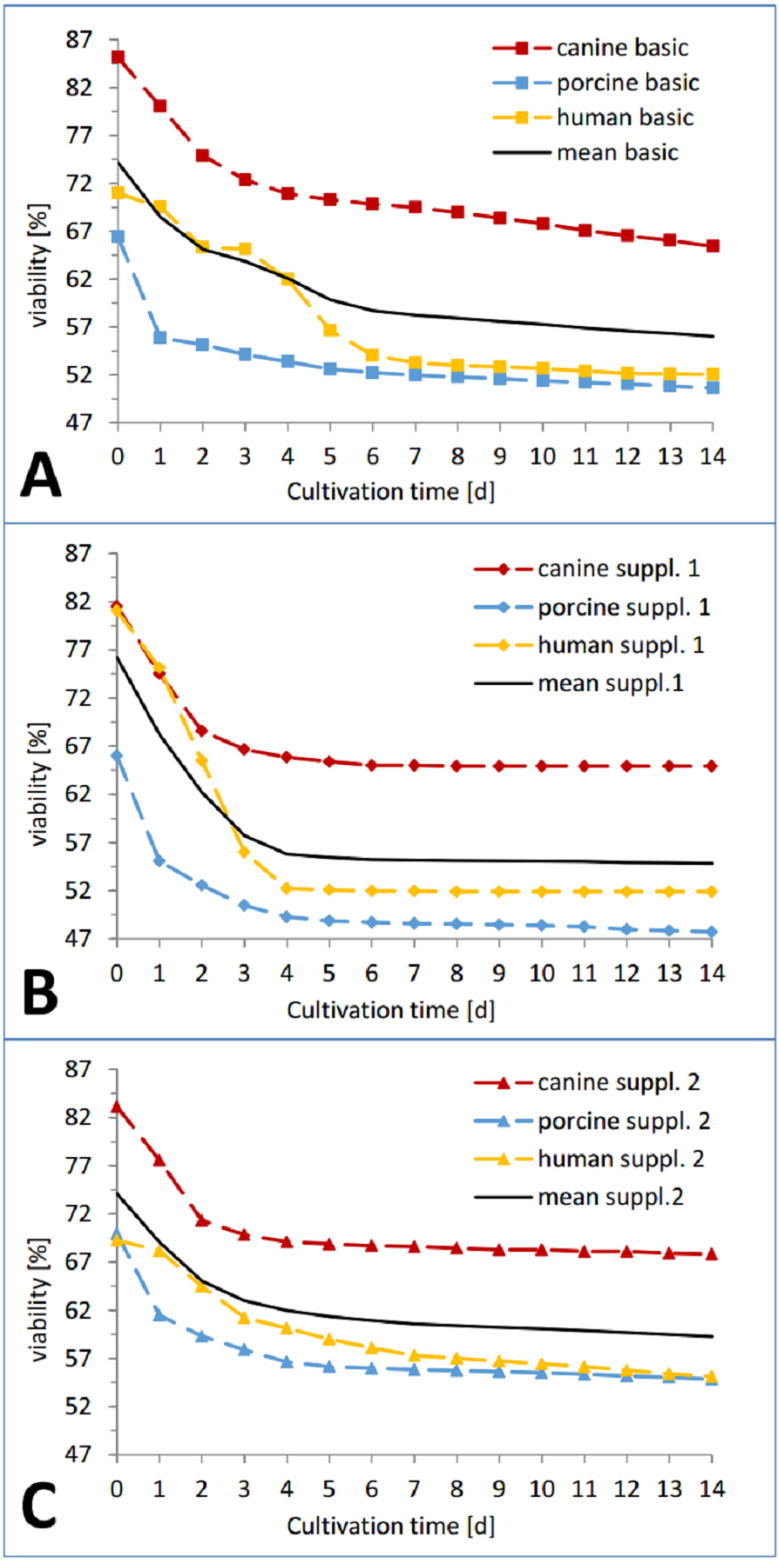
Viability trend of the skin samples in percent over 14 days of cultivation, comparing cultivation media. Viability trend (measurement timepoints connected via trend-lines) in percent relative to the corresponding positive control for the three investigated species: human (yellow), pig (blue) and dog (red) in order to compare differences between cultivation media. All the data points are mean values from three skin punches cultivated in the same condition, measured *n* = 2. (A) Basic medium, (B) medium with added supplement mixture 1, (C) medium with added supplement mixture 2.

**Figure 5:**
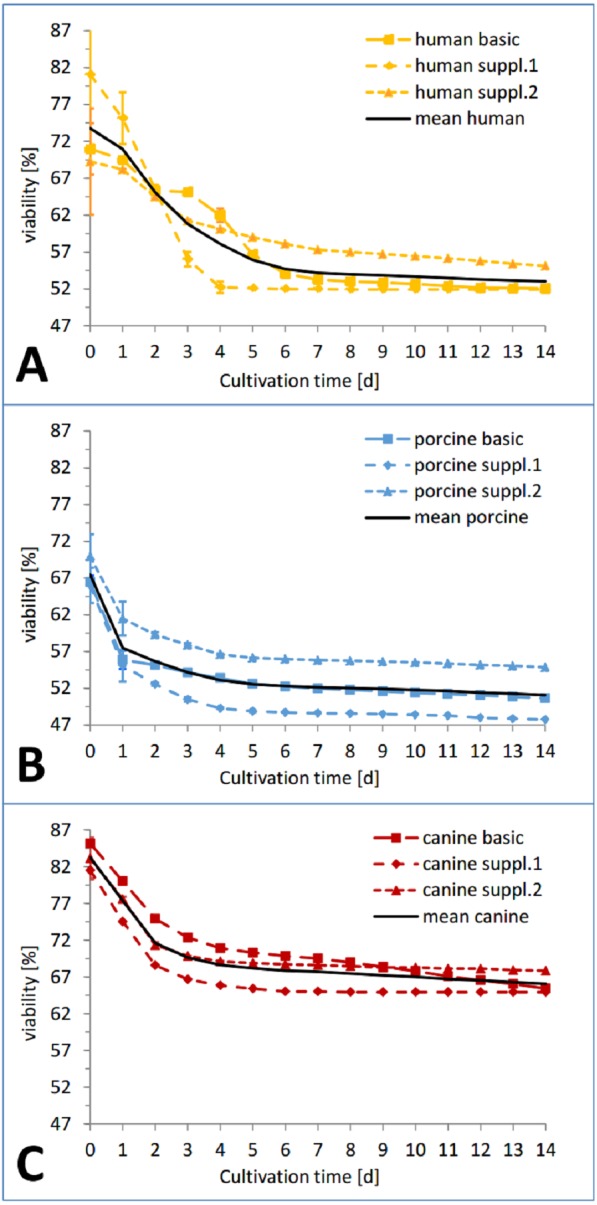
Viability trend of the skin samples in percent over 14 days of cultivation, comparing species. Viability trend (measurement timepoints connected via trend-lines) in percent relative to the corresponding positive control for the three investigated cultivation conditions: basic medium, supplement mixture 1 and supplement mixture 2 in order to compare differences between species. All the data points are mean values from three skin punches cultivated in the same condition, measured *n* = 2. (A) Human skin, (B) porcine skin, (C) canine skin.

**Figure 6:**
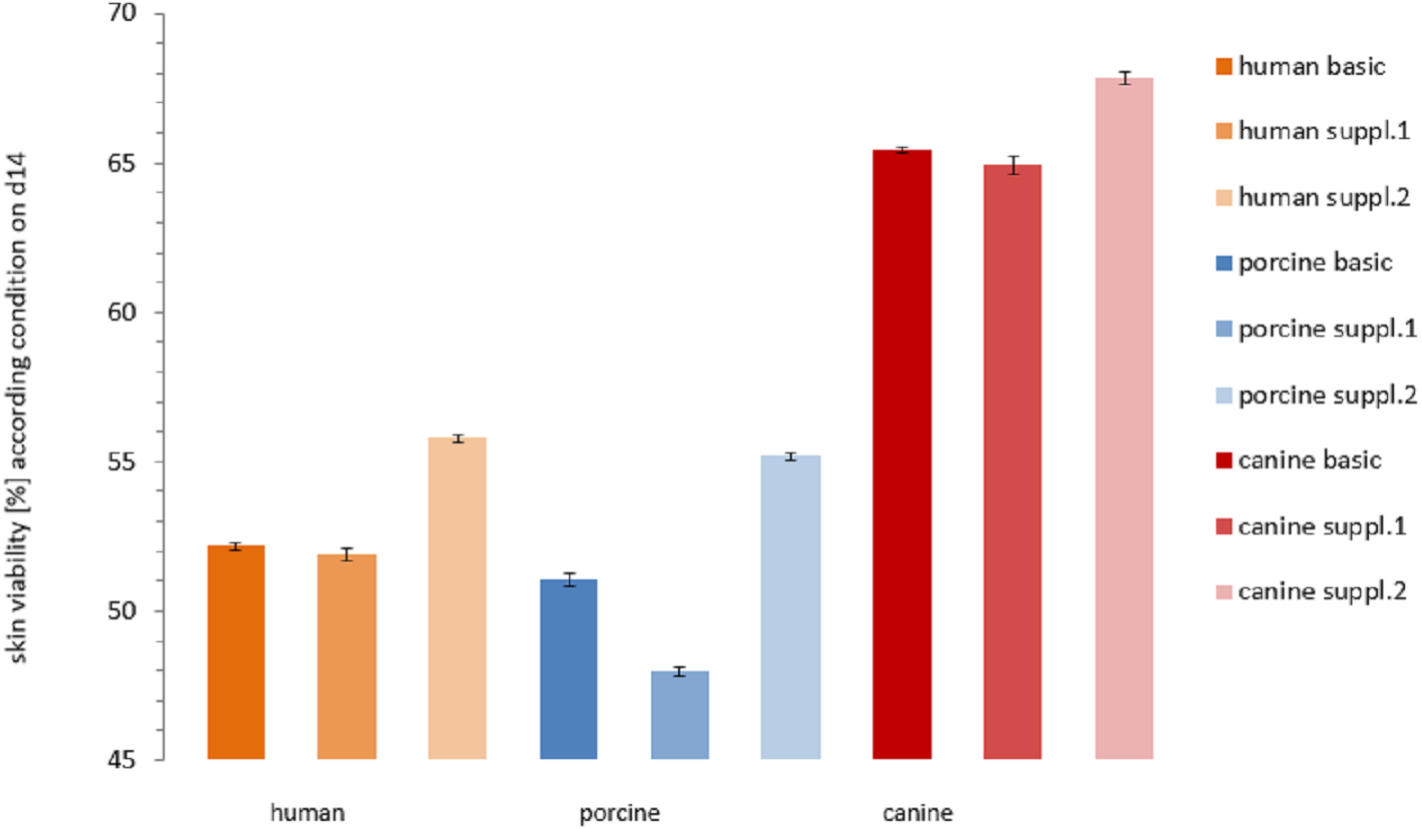
Comparison of the viability of the skin samples at the end of the study. Remaining viability of the skin samples at the end of the study (day 14). Differences in viability levels between the three species: human (yellow), pig (blue) and dog (red) and between the three cultivation media: basic medium (plain), medium with supplement mixture 1 (dotted) and supplement mixture 2 (striped) are compared. The values are given as mean of each cultivation condition (e.g. human skin in basic medium).

In the viability trend (Fig. 4 and Fig. 5), the mean percentage of viability of the three investigated species already ranged from 83.26% (canine) over 73.76% (human) to 67.46% (porcine) on the first measurement time point, displaying an order that was maintained until day 7 (67,70% canine, 54.18% human, 52,13% porcine) and throughout the whole cultivation time. The curves of the different cultivation media for one species were similar in the case of porcine and canine skin (Figs. 5B and 5C) but not for human skin where the three curves exhibited a different behavior and intersected on the second day of cultivation (Fig. 5A). A rather rapid drop in viability occurred during the first two to three cultivation days, followed by a gradual decrease afterwards. This decrease happened until day 1 in porcine skin (mean), until day 2 for canine skin and until day 3 for human skin with a slope being slighter in the latter than the other ones (Fig. 5). The difference in viability between the three species at the beginning of the cultivation was not significant (NPH: KWA *p* = 0.36788 DF 2, MM *p* = 0.22313 DF 2) while the difference after seven cultivation days was significant (NPH: KWA *p* = 0.01832 DF 2, MM *p* = 0.01111 DF 2). The statistical species difference in viability for day 14 is reported with Fig. 6.

In the course of the first cultivation week (roughly until day 5 to 7) a marked decrease in viability was visible for each curve while afterwards either a plateau was reached (Fig. 4B) or a slight further decrease could be observed (Figs. 4A and 4C). The effect of the respective cultivation medium can be seen in Fig. 4, where for all three species, supplement mixture 1 obtained the lowest (Fig. 4B) and supplement mixture 2 the highest viability results (Fig. 4C) are shown.

The viability of each plate on the last day of cultivation is shown in Fig. 6. Human skin in basic medium reached a viability of 52.16 ± 0.10%. In supplemented medium it obtained a viability of 51.90 ± 0.20% (Supplemental Information 1 = insulin + hydrocortisone) and 55.77 ± 0.10% (Supplemental Information 2 = BPE + hEGF), respectively. Porcine skin viability ranged from 51.06 ± 0.21 % (basic medium) over 47.98 ± 0.15% (Supplemental Information 1) to 55.17 ± 0.12% (Supplemental Information 2). Canine skin viability extended from 65.43 ± 0.09% (basic) over 64.93 ± 0.30% (Supplemental Information 1) to 67.83 ± 0.22%. The differences were significant between canine skin and human skin (KWA *p* = 0.04953 DF1) and between canine and porcine skin (KWA *p* = 0.04953 DF1), but not between human and porcine skin (KWA *p* = 0.05091 DF2). The difference between all three conditions was again significant (MM *p* = 0.04285 DF2). The absolute difference between the cultivation condition with the highest and the lowest viability were 2.9% for canine, 3.78% for human and 7.19% for porcine skin. By referring to the corresponding highest value as 100%, hence calculating the relative variance describing the ratio of the observed difference compared to the absolute values, canine skin showed 4.28% difference, human skin 6.78% and porcine skin 13.03%. The variation within porcine skin was therefore about double compared to human skin and four times higher than within canine skin.

The effects of medium supplements on viability were significant comparing supplement mixture 1 with supplement mixture 2 (MM *p* = 0.0455 1DF) and basic medium with supplement mixture 2 for all three species (MM *p* = 0.0455 1DF). Comparison of basic medium with supplement mixture 1 resulted in a significant difference only for porcine skin but not for the other two species (MM *p* = 0.0455 DF1). However, these findings might have been more significant if a larger sample size was available.

## Discussion

In this study, a viable in vitro model of cultured human, porcine and canine skin was established and cultivated for two weeks with regular viability measurements using a modified LDH release assay (Bauhammer, Sacha & Haltner, 2019a). The total LDH content of each skin specimen and the influence of two different medium supplements on the skin integrity and viability were investigated.

By now, a large variety of skin models from human skin has been established, closely followed by different porcine models while for canine skin very scarce information can be found in the literature, emphasizing the importance of this research. One of the most common porcine cultured skin models is the pig ear skin model. The skin of a pig’s ear is comparable to human skin in terms of skin thickness, lipids, hair follicle density and permeability (Flaten et al., 2015). Percutaneous absorption and skin metabolism of pig ear skin was evaluated by Jacques et al. (2010) While the sample processing and cultivation conditions were similar to the current study, the skin was sectioned to a defined thickness with a dermatome (vs usage of full skin) and only a short-term culture of 48 h was performed (vs. 14 days). Further examples include Scognamiglio et al. (2013) and Gomes et al. (2014) who used pig ear skin for the evaluation of deformable liposomes and ethosomes or nanostructured lipid carriers, respectively. Porcine abdominal skin (dermatomed) was employed by Nagelreiter et al. (2013). One example of a dog skin model is described by Serra et al. (2007) who developed a canine skin equivalent with isolated keratinocytes and fibroblasts which differentiated into a multilayer epidermis starting from day 15. A cultivation method with canine skin was reported by Abramo et al. (2016) who used full-thickness adult canine skin in serum-free medium. These conditions are similar to the ones employed in the current study, however, the skin was only maintained until day 7 and four mm skin biopsies have been collected in contrast to skin culture over 14 days and 13 mm punches in this study.

Skin specimens from the three species were cultured for two weeks. The skin could be maintained at minimum 50% viability for the whole 14 days of cultivation which is a better result compared to other studies with a similar approach, such as described by Castagnoli et al. (2003), Suarato et al. (2018) and Wever, Kurdykowski & Descargues (2015). De Wever investigated the NativeSkin^®^ model where human skin biopsies from plastic surgery were placed in a solid nourishing matrix and were maintained viable for 7 days, using MTT assay and histologic characterization as viability markers. Castagnoli used human post-mortem allograft skin biopsies. Their viability was also assessed with the MTT assay. A viability level of the fresh skin of around 75% was reported after 12–30 h following harvesting which decreased to 40% after 60 h. After 6 days of cultivation, the viability further decreased to 0% which held also true for cryopreserved samples using 10% DMSO. Storage at 4 °C extended viability but did not prevent its decrease to 25% after 15 days. Suarato, however, used explanted mouse skin in a 3D printed diffusion cell which could be maintained viable for maximum 24 h, assessed again with the MTT assay.

Due to the different cultivation conditions (e.g., sample size, number of samples, cultivation medium) and the necessity for repeated measurements to assess the skin viability, the LDH assay was deemed a suitable alternative. The viability profile of the three cultivation conditions for each species showed a notable difference in viability starting from the first measurement which, however did not prove to be statistically significant. A sharp decrease in viability from the start until the first (pig), second (dog) and third (human) cultivation day is typical for this kind of tissue culture, also the following plateau or relatively flat interim period. A second decrease phase is reported in the literature (Legrand et al., 1992; Reus et al., 2011) but was not observed here.

The evaluation of the LDH content of human, porcine and canine skin based on the positive control samples revealed a rank order of LDH content as follows: human >porcine >canine skin. The total LDH release over the complete cultivation time reflected this. The relative LDH release of each skin specimen in relation to the positive control, however, was in the order porcine >human >canine skin. This, combined with the rather low initial viability, might be a contributing factor to porcine skin yielding the lowest viability results at the end of cultivation. The low initial viability values could be due to the longer transport of the pig cadaver from the slaughterhouse to the place of processing, which was in another city. However, the differences in the initial viability levels were not significant and the viability of human skin after 14 days only slightly exceeded that of porcine skin, so that a major influence seems improbable. Over the whole study, the canine skin specimen maintained the highest viability.

Still, these results do not claim to prove a generally higher viability of canine skin which is not possible due to a notable amount of factors influencing skin properties as mentioned in the introduction. Although with human abdominal, canine flank skin and porcine back skin three physically adjacent regions (with assumed functional similarities) have been selected and the influence of sex, age and weight has been considered, variations in viability due to different anatomical and functional properties (e.g., skin thickness, lipid content and composition, absorption of nutrients etc.) could not be excluded. This may be especially the case considering canine skin, as morphological and functional analysis of porcine back skin showed great similarities to human abdominal skin (Khiao In et al., 2019). However, there is little reliable information available about these properties in other animal skin and how and to which extent they may have affected the outcome of this study or about their influence on TDD in general. Therefore, a new concept such as employed in this study has to be based on a certain amount of assumptions and speculations which will have to be verified or falsified in the future.

It should also be taken into account that different skin models with rodent skin (e.g., rat, mouse, guinea pig and several hairless breeds of those) have been widely used as surrogates for human skin despite substantial differences in size, skin thickness, lipid composition, metabolism and skin barrier function (Flaten et al., 2015; Mathes, Ruffner & Graf-Hausner, 2014; Rodríguez-Luna et al., 2018). In view of this, the current selection of species and body regions of the explants may be justified for the scope of such a pilot study.

The two supplement mixtures consisting of insulin plus hydrocortisone supplement mixture 1 and BPE plus hEGF supplement mixture 2 were selected according to Lasnitzky (1993). For all the samples, the medium supplement mixture 2 was the most effective in promoting health and viability of the skin specimens. This could be supported by a statistically significant difference between basic medium and supplement mixture 1 compared to supplement mixture 2. This effect was significant in every species which makes usage and optimization of supplement mixture 2 the most promising approach for further studies. Among the cultivated samples, the porcine skin samples cultured with supplement mixture 1 failed as only ones to maintain a viability >50% until the end of the study and had therefore been deemed as non-viable. While for all the species, supplement mixture 1 had a certain negative effect on the viability even compared to basic medium, for porcine skin which generally expressed the highest relative variation between the three culture conditions, this effect was more pronounced and statistically significant. Therefore, supplement mixture 1 seems less suitable for further experiments. All the other skin samples (except porcine + supplement mixture 1) could be kept at a viability level of over 50% until the end of the study.

In this study, no serum was used; hence, some of the nutrients for the skin cells were introduced via these medium supplements. The positive effect of the supplement mixture 2 will have to be assessed in detail but may be due to the mitogen, proliferative and lifespan-extending effects of EGF and BPE on different cell types such as keratinocytes, which are described in the literature (Blaimauer et al., 2006; Gharibi & Hughes, 2012; Hebert et al., 2009; Pastore et al., 2008; Xu et al., 2013). The negative effect of the supplement mixture 1 is most likely induced by hydrocortisone, as this component is known to possibly having both positive and negative effects which are strongly dose-dependent (Zoller et al., 2008). The higher influence of supplement mixture 1 on porcine skin may therefore be explained by high levels of stress-induced corticoids in the body of the corresponding pig at the time of slaughter (Dokmanovic et al., 2015). Together with the supplement, the resulting amount of hydrocortisone may have been high enough to induce a viability decrease. However, choosing a lower concentration of hydrocortisone might be an option to be considered.

## Conclusions

This *in vitro* viable cultured skin model is a first step towards the investigation of canine and porcine skin diseases. In its current shape, it may be employed for topical application studies, percutaneous absorption and, due to its viability, basic cutaneous research, e.g., keratinocyte reactions to irritation. Upon further optimization, it may be used for assessment of skin metabolism which can be essential when applying xenobiotics (e.g., drugs) to the skin (Jacques et al., 2010). New cosmetic products or nanoformulations may be tested based on this model as well (Kraeling et al., 2018). For these purposes, the skin viability and its structural integrity are crucial. Therefore, skin cultivated with the supplement mixture 2 is the most suitable candidate between the three tested conditions for further research. An increase of the current viability levels is planned for the future. Compared to the first step of this research (Bauhammer, Sacha & Haltner, 2019a), the lifespan of the skin has already been almost doubled. Different concentrations of the supplements, other cultivation media and supplements may be tested for further viability improvement.

The development of a complex disease model may later be based on the current *in vitro* cultivated skin model, being especially relevant for canine skin as only relatively few models exist (Serra et al., 2007). Introduction of skin from other species may considerably expand its scope. Skin properties and permeability may be evaluated and categorized with the aim to create a comparable data set and standards similar to those in human medicine.

##  Supplemental Information

10.7717/peerj.7811/supp-1 Supplemental Information 1 Skin cultivation measurements and calibration curve

10.7717/peerj.7811/supp-2 Supplemental Information 2 Non-parametric statistical analysis: Kruskal-Wallis-ANOVA, K-Sample Test, Moods Median Test with 0.05 significance level and exact *p*-values. Evaluation of absolute and relative amount LDH released, species difference in viability throughout the cultivatio

10.7717/peerj.7811/supp-3 Supplemental Information 3 Non-parametric statistical analysis: Moods Median test with 0.05 significance level and exact p-values. Evaluation of the effect of the medium supplements on viability

10.7717/peerj.7811/supp-4 Supplemental Information 4 Summary of skin cultivation measurements and calculation of viability

10.7717/peerj.7811/supp-5 Supplemental Information 5 Word list translated into English The protocols in the supplementary files containing German references are locked and cannot be changed as they are validated for the Lab. Relevant German references and evaluation comments have been translated to English in this list to facilitate the understanding of these files.

## Additional Information and Declarations

### Data Availability

The following information was supplied regarding data availability: The raw data is available in the Supplemental Files.
