# Supplementary material for: Establishment of an in vitro model of cultured viable human, porcine and canine skin and comparison of different media supplements
Source: PeerJ. 2019 Oct 3;7:e7811. doi: 10.7717/peerj.7811 (PMC6778665; doi:10.7717/peerj.7811)
Supplement: Supplemental Information 1 [file peerj-07-7811-s001.pdf]

IsBa_180815_Evaluation_skin_cult_humanT0-3.pdf

IsBa_180815_Evaluation_skin_cult_humanT4-7.pdf

IsBa_180815_Evaluation_skin_cult_humanT8-11.pdf

IsBa_180815_Evaluation_skin_cult_humanT12-14.pdf

IsBa_180815_Evaluation_skin_cult_porcineT0-3.pdf

IsBa_180815_Evaluation_skin_cult_porcineT4-7.pdf

IsBa_180815_Evaluation_skin_cult_porcineT8-11.pdf

IsBa_180815_Evaluation_skin_cult_porcineT12-14.pdf

IsBa_180815_Evaluation_skin_cult_canineT0-7.pdf

IsBa_180815_Evaluation_skin_cult_canineT8-14.pdf

IsBa_180820_Evaluation_+-controls_all_skins.pdf

Project number		F-120	Apparatus	Wallac Victor	Operator	IsBa
GLP Study (Number)		n.a.	Protocol (Instrument method)	LDH test 2016	Date of preparation	19-04-18
hot substance	isotope	n.a.	File name (results)	IsBa_180419/20_LDH_full_va	Date of measurement	19-04-18
	name	n.a.	Kind of well plate	96 well	shaking time [min]	30
	ACB-ID	n.a.	sample volume [µL]	100	stirring rate (Target) [rpm]	150
	Batch number	n.a.	Cocktail volume [µL]	175	Kind of measurement	UV-vis
cold substance	name	LDH	ACB-ID of cocktail		Wave length [nm]	450
	ACB-ID	L23982R	Matrix	DMEM (from powder)+PEG	Remarks	Cocktail 100µl RM, 75µl STOP
	Batch number	SLBK6345V	Blank description	DMEM/PEG, H2O	Remarks	7 standards split low/high
n.a.		n.a.	Pipettes (No. / volume)	50-200µl	Remarks	KLP4 common for both
n.a.		n.a.	Pipettes (No. / volume)	n.a.	Remarks	n.a.

Messdaten (diese Tabelle in Bericht übernehmen)

Sample name *	concentration (theor.) *	measured data	measured data	measured data	mean measured	SD	RSD	Blank *	measured data after * Blank subtraction	concentration (calc.) *	Deviation *	Residuen
	[µg/mL]	[AU]	[AU]	[AU]	[AU]	[AU]	[%]	[AU]		[µg/mL]	[%]	
KLP1								0.047				
KLP2								0.054				
KLP3								0.051				
KLP4	0.041	0.959	0.972	0.883	0.938	0.04	4.19	0.051	0.887	0.041	-0.57	0.00
KLP5	0.018	0.483	0.484	0.474	0.480	0.00	0.98	0.052	0.429	0.019	2.42	0.00
KLP6	0.012	0.360	0.359	0.326	0.348	0.02	4.54	0.051	0.297	0.013	5.00	0.00
KLP7	0.007	0.228	0.228	0.199	0.218	0.014	6.35		0.168	0.006	-11.29	0.00
KLP8												

Statistical data

Geradensteigung	Slope	m	21.03
Y-Achsenabschnitt	Y-Intercept	b	0.03
Standardabw. Geradensteigung	SD-Slope	s_m	0.648055501
Standardabw. Achsenabschnittes	SD-Y-Intercept	s_b	0.015177452
Anzahl Messpunkte	number of measuring points	n	4
Quadratsumme	sum of squares	Qxx	0.000664048
Bereichsmitte			0.019557193
Freiheitsgrade	degree of freedom	f	2
Student-t-Faktor für (P = 95 %; f = n-2)	Student-t-factor for (P = 95 %; f = n-2)	t	4.303
Vertrauensbereich Steig. (95 %) Obergrenze		$m + VB_m$	23.81763508
Vertrauensbereich Steig. (95 %) Untergrenze		$m - VB_m$	18.24046944
Vertrauensbereich Achsenabschnitt (95 %) Og.		$b + Vb_b$	0.099335829
Vertrauensbereich Achsenabschnitt (95 %) Ug.		$b - Vb_b$	-0.031281327
Korrelationskoeffizient	correlation coefficient	r	0.9991
Bestimmtheitsmaß	determiantion coefficient	r^2	0.9981
Reststandardabweichung		s_0	0.01669983
Summe Restquadrate		sd	0.657385838
Verfahrensstandardabw.		s_{x0}	0.000794131
Rel. Verfahrensstandardabw. %		V_{x0}	4.060559029

Berichten

mean Blank	**0**	
SD Blank	0.00	
RSD Blank	3.79	x=
x*SD (LLOQ)	0.01	5
x*SD (LOD)	0.01	3
**LLOQ (AU)**	**0.061**	
**LOD (AU)**	**0.057**	
ULOQ	0.887	
LLOQ (Lin)	0.168	

Evaluation / Comment

LDH linearity valid with 7 standards, split in lin high and lin low, each with 4 standards and KLP4 common standard for both. R² 0,9981, deviations for both between -13.34% and +13.04%

Date   Operator   Date   Control

Formblatt-Nr.		Version	
Titel			
Gültig ab		Ablage	Projektordner

Figure 1 Linearity

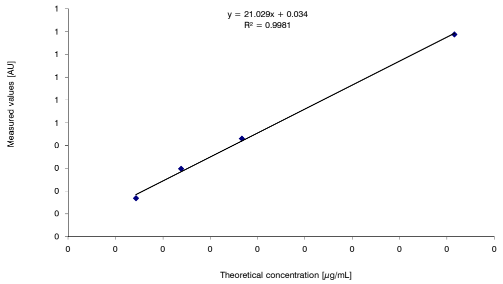

Figure 2 Method validation Residuen Plot

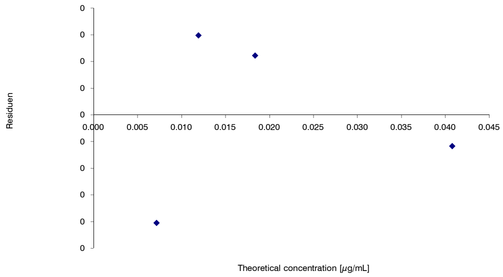

Evaluation / Comment

lin passed

Date	Operator	Date	Control

Sample name	Plate	Position	[AU]	[AU]-Blank	Rating	[µg/mL]	Dilution factor	[µg/mL]
0	T0	A01	0	0	kleiner LLOQ	#VALUE!	1	#VALUE!
0	T0	A02	0	0	kleiner LLOQ	#VALUE!	1	#VALUE!
0	T0	A03	0	0	kleiner LLOQ	#VALUE!	1	#VALUE!
0	T0	A04	0	0	kleiner LLOQ	#VALUE!	1	#VALUE!
T0HN1	T0	A05	0	0	kleiner LLOQ	#VALUE!	1	#VALUE!
T0HN1	T0	A06	0	0	kleiner LLOQ	#VALUE!	1	#VALUE!
T0HN2	T0	A07	0	0	kleiner LLOQ	#VALUE!	1	#VALUE!
T0HN2	T0	A08	0.702	0.651	0.651	0.029	176	5.164
T0HN3	T0	A09	0	0	kleiner LLOQ	#VALUE!	1	#VALUE!
T0HN3	T0	A10	0	0	kleiner LLOQ	#VALUE!	1	#VALUE!
T0HN4	T0	A11	0.475	0.424	0.424	0.019	2.75	0.051
T0HN4	T0	A12	0.393	0.342	0.342	0.015	2.75	0.040
0	T0	B01	0	0	kleiner LLOQ	#VALUE!	1	#VALUE!
0	T0	B02	0	0	kleiner LLOQ	#VALUE!	1	#VALUE!
0	T0	B03	0	0	kleiner LLOQ	#VALUE!	1	#VALUE!
0	T0	B04	0	0	kleiner LLOQ	#VALUE!	1	#VALUE!
T0HS11	T0	B05	0.879	0.828	0.828	0.038	22	0.831
T0HS11	T0	B06	0	0	kleiner LLOQ	#VALUE!	1	#VALUE!
T0HS12	T0	B07	0	0	kleiner LLOQ	#VALUE!	1	#VALUE!
T0HS12	T0	B08	0	0	kleiner LLOQ	#VALUE!	1	#VALUE!
T0HS13	T0	B09	0.811	0.760	0.760	0.035	176	6.076
T0HS13	T0	B10	0.727	0.676	0.676	0.031	176	5.373
T0HS14	T0	B11	0.349	0.298	0.298	0.013	2.75	0.035
T0HS14	T0	B12	0.252	0.201	0.201	0.008	2.75	0.022
0	T0	C01	0	0	kleiner LLOQ	#VALUE!	1	#VALUE!
0	T0	C02	0	0	kleiner LLOQ	#VALUE!	1	#VALUE!
0	T0	C03	0	0	kleiner LLOQ	#VALUE!	1	#VALUE!
0	T0	C04	0	0	kleiner LLOQ	#VALUE!	1	#VALUE!
T0HS21	T0	C05	0	0	kleiner LLOQ	#VALUE!	1	#VALUE!
T0HS21	T0	C06	0	0	kleiner LLOQ	#VALUE!	1	#VALUE!
T0HS22	T0	C07	0	0	kleiner LLOQ	#VALUE!	1	#VALUE!
T0HS22	T0	C08	0	0	kleiner LLOQ	#VALUE!	1	#VALUE!
T0HS23	T0	C09	0	0	kleiner LLOQ	#VALUE!	1	#VALUE!
T0HS23	T0	C10	0	0	kleiner LLOQ	#VALUE!	1	#VALUE!
T0HS24	T0	C11	0.283	0.232	0.232	0.009	2.75	0.026
T0HS24	T0	C12	0	0	kleiner LLOQ	#VALUE!	1	#VALUE!
0	T0	D01	0	0	kleiner LLOQ	#VALUE!	1	#VALUE!
0	T0	D02	0	0	kleiner LLOQ	#VALUE!	1	#VALUE!
0	T0	D03	0	0	kleiner LLOQ	#VALUE!	1	#VALUE!
0	T0	D04	0	0	kleiner LLOQ	#VALUE!	1	#VALUE!
0	T0	D05	0	0	kleiner LLOQ	#VALUE!	1	#VALUE!
0	T0	D06	0	0	kleiner LLOQ	#VALUE!	1	#VALUE!
0	T0	D07	0	0	kleiner LLOQ	#VALUE!	1	#VALUE!
0	T0	D08	0	0	kleiner LLOQ	#VALUE!	1	#VALUE!
0	T0	D09	0	0	kleiner LLOQ	#VALUE!	1	#VALUE!
0	T0	D10	0	0	kleiner LLOQ	#VALUE!	1	#VALUE!
0	T0	D11	0	0	kleiner LLOQ	#VALUE!	1	#VALUE!
0	T0	D12	0	0	kleiner LLOQ	#VALUE!	1	#VALUE!

Plate:	T0

	Test 1	Test 2	Test 3	Test 4	5	6	7	8	9	10	11	12
A								0.702			0.475	0.393
B					0.879				0.811	0.727	0.349	0.252
C											0.283	0.211
D												
E												
F												
G							0.061	0.065	0.056	0.055	0.055	0.057
H							0.051	0.05	0.051	0.05	0.05	0.049

Plattenbelegung

	1	2	3	4	5	6	7	8	9	10	11	12
A					T0HN1	T0HN1	T0HN2	T0HN2	T0HN3	T0HN3	T0HN4	T0HN4
B					T0HS11	T0HS11	T0HS12	T0HS12	T0HS13	T0HS13	T0HS14	T0HS14
C					T0HS21	T0HS21	T0HS22	T0HS22	T0HS23	T0HS23	T0HS24	T0HS24
D												
E												
F												
G							Blank PEG	Blank PEG	Blank PEG	Blank PEG	Blank PEG	Blank PEG
H							Blank no PEG	Blank no PEG	Blank no PEG	Blank no PEG	Blank no PEG	Blank no PEG

Samples aufgeteilt in lin high und low:höher konzentrierte Samples bei lin high zu finden.

Comment:

Erste 4 Reihen immer Testmessung zur Evaluierung der benötigten Verdünnung. Wird nicht mit ausgewertet. Unverdünnte Messungen außerhalb der Linearität werden ebenfalls nicht ausgewertet. Dil.Faktor unverdünnte samples=2,75 (100µl sample+100µlRM+75µl stop sol.) Verdünnung (gelb) Dil.8*Dil8*Dil2,75=Dil.176

Date Operator

Date Control

0	T0	E01	0	0	kleiner LLOQ	#VALUE!	1	#VALUE!
0	T0	E02	0	0	kleiner LLOQ	#VALUE!	1	#VALUE!
0	T0	E03	0	0	kleiner LLOQ	#VALUE!	1	#VALUE!
0	T0	E04	0	0	kleiner LLOQ	#VALUE!	1	#VALUE!
0	T0	E05	0	0	kleiner LLOQ	#VALUE!	1	#VALUE!
0	T0	E06	0	0	kleiner LLOQ	#VALUE!	1	#VALUE!
0	T0	E07	0	0	kleiner LLOQ	#VALUE!	1	#VALUE!
0	T0	E08	0	0	kleiner LLOQ	#VALUE!	1	#VALUE!
0	T0	E09	0	0	kleiner LLOQ	#VALUE!	1	#VALUE!
0	T0	E10	0	0	kleiner LLOQ	#VALUE!	1	#VALUE!
0	T0	E11	0	0	kleiner LLOQ	#VALUE!	1	#VALUE!
0	T0	E12	0	0	kleiner LLOQ	#VALUE!	1	#VALUE!
0	T0	F01	0	0	kleiner LLOQ	#VALUE!	1	#VALUE!
0	T0	F02	0	0	kleiner LLOQ	#VALUE!	1	#VALUE!
0	T0	F03	0	0	kleiner LLOQ	#VALUE!	1	#VALUE!
0	T0	F04	0	0	kleiner LLOQ	#VALUE!	1	#VALUE!
0	T0	F05	0	0	kleiner LLOQ	#VALUE!	1	#VALUE!
0	T0	F06	0	0	kleiner LLOQ	#VALUE!	1	#VALUE!
0	T0	F07	0	0	kleiner LLOQ	#VALUE!	1	#VALUE!
0	T0	F08	0	0	kleiner LLOQ	#VALUE!	1	#VALUE!
0	T0	F09	0	0	kleiner LLOQ	#VALUE!	1	#VALUE!
0	T0	F10	0	0	kleiner LLOQ	#VALUE!	1	#VALUE!
0	T0	F11	0	0	kleiner LLOQ	#VALUE!	1	#VALUE!
0	T0	F12	0	0	kleiner LLOQ	#VALUE!	1	#VALUE!
0	T0	G01	0	0	kleiner LLOQ	#VALUE!	1	#VALUE!
0	T0	G02	0	0	kleiner LLOQ	#VALUE!	1	#VALUE!
0	T0	G03	0	0	kleiner LLOQ	#VALUE!	1	#VALUE!
0	T0	G04	0	0	kleiner LLOQ	#VALUE!	1	#VALUE!
0	T0	G05	0	0	kleiner LLOQ	#VALUE!	1	#VALUE!
0	T0	G06	0	0	kleiner LLOQ	#VALUE!	1	#VALUE!
Blank PEG	T0	G07	0	0	kleiner LLOQ	#VALUE!	2.75	#VALUE!
Blank PEG	T0	G08	0	0	kleiner LLOQ	#VALUE!	2.75	#VALUE!
Blank PEG	T0	G09	0	0	kleiner LLOQ	#VALUE!	2.75	#VALUE!
Blank PEG	T0	G10	0	0	kleiner LLOQ	#VALUE!	2.75	#VALUE!
Blank PEG	T0	G11	0	0	kleiner LLOQ	#VALUE!	2.75	#VALUE!
Blank PEG	T0	G12	0	0	kleiner LLOQ	#VALUE!	2.75	#VALUE!
0	T0	H01	0	0	kleiner LLOQ	#VALUE!	1	#VALUE!
0	T0	H02	0	0	kleiner LLOQ	#VALUE!	1	#VALUE!
0	T0	H03	0	0	kleiner LLOQ	#VALUE!	1	#VALUE!
0	T0	H04	0	0	kleiner LLOQ	#VALUE!	1	#VALUE!
0	T0	H05	0	0	kleiner LLOQ	#VALUE!	1	#VALUE!
0	T0	H06	0	0	kleiner LLOQ	#VALUE!	1	#VALUE!
Blank no PEG	T0	H07	0	0	kleiner LLOQ	#VALUE!	2.75	#VALUE!
Blank no PEG	T0	H08	0	0	kleiner LLOQ	#VALUE!	2.75	#VALUE!
Blank no PEG	T0	H09	0	0	kleiner LLOQ	#VALUE!	2.75	#VALUE!
Blank no PEG	T0	H10	0	0	kleiner LLOQ	#VALUE!	2.75	#VALUE!
Blank no PEG	T0	H11	0	0	kleiner LLOQ	#VALUE!	2.75	#VALUE!
Blank no PEG	T0	H12	0	0	kleiner LLOQ	#VALUE!	2.75	#VALUE!

0	T1	A01	0	0	kleiner LLOQ	#VALUE!	1	#VALUE!
0	T1	A02	0	0	kleiner LLOQ	#VALUE!	1	#VALUE!
0	T1	A03	0	0	kleiner LLOQ	#VALUE!	1	#VALUE!
0	T1	A04	0	0	kleiner LLOQ	#VALUE!	1	#VALUE!
0	T1	A05	0	0	kleiner LLOQ	#VALUE!	1	#VALUE!
0	T1	A06	0	0	kleiner LLOQ	#VALUE!	1	#VALUE!
T1HN2	T1	A07	0.423	0.372	0.372	0.016	13.75	0.22
T1HN2	T1	A08	0.615	0.564	0.564	0.025	13.75	0.35
T1HN3	T1	A09	0.670	0.619	0.619	0.028	13.75	0.38
T1HN3	T1	A10	0.724	0.673	0.673	0.030	13.75	0.42
T1HN4	T1	A11	0.150	0.099	kleiner LLOQ	#VALUE!	2.75	#VALUE!
T1HN4	T1	A12	0.153	0.102	kleiner LLOQ	#VALUE!	2.75	#VALUE!
0	T1	B01	0	0	kleiner LLOQ	#VALUE!	1	#VALUE!
0	T1	B02	0	0	kleiner LLOQ	#VALUE!	1	#VALUE!
0	T1	B03	0	0	kleiner LLOQ	#VALUE!	1	#VALUE!
0	T1	B04	0	0	kleiner LLOQ	#VALUE!	1	#VALUE!
0	T1	B05	0	0	kleiner LLOQ	#VALUE!	1	#VALUE!
0	T1	B06	0	0	kleiner LLOQ	#VALUE!	1	#VALUE!
0	T1	B07	0	0	kleiner LLOQ	#VALUE!	1	#VALUE!
0	T1	B08	0	0	kleiner LLOQ	#VALUE!	1	#VALUE!
0	T1	B09	0	0	kleiner LLOQ	#VALUE!	1	#VALUE!
0	T1	B10	0	0	kleiner LLOQ	#VALUE!	1	#VALUE!
T1HS14	T1	B11	0.147	0.096	kleiner LLOQ	#VALUE!	2.75	#VALUE!
T1HS14	T1	B12	0.138	0.087	kleiner LLOQ	#VALUE!	2.75	#VALUE!
0	T1	C01	0	0	kleiner LLOQ	#VALUE!	1	#VALUE!
0	T1	C02	0	0	kleiner LLOQ	#VALUE!	1	#VALUE!
0	T1	C03	0	0	kleiner LLOQ	#VALUE!	1	#VALUE!
0	T1	C04	0	0	kleiner LLOQ	#VALUE!	1	#VALUE!
0	T1	C05	0	0	kleiner LLOQ	#VALUE!	1	#VALUE!
0	T1	C06	0	0	kleiner LLOQ	#VALUE!	1	#VALUE!
0	T1	C07	0	0	kleiner LLOQ	#VALUE!	1	#VALUE!
0	T1	C08	0	0	kleiner LLOQ	#VALUE!	1	#VALUE!
0	T1	C09	0	0	kleiner LLOQ	#VALUE!	1	#VALUE!
0	T1	C10	0	0	kleiner LLOQ	#VALUE!	1	#VALUE!
T1HS24	T1	C11	0.092	0.041	kleiner LLOQ	#VALUE!	2.75	#VALUE!
T1HS24	T1	C12	0.112	0.061	kleiner LLOQ	#VALUE!	2.75	#VALUE!
0	T1	D01	0	0	kleiner LLOQ	#VALUE!	1	#VALUE!
0	T1	D02	0	0	kleiner LLOQ	#VALUE!	1	#VALUE!
0	T1	D03	0	0	kleiner LLOQ	#VALUE!	1	#VALUE!
0	T1	D04	0	0	kleiner LLOQ	#VALUE!	1	#VALUE!
0	T1	D05	0	0	kleiner LLOQ	#VALUE!	1	#VALUE!
0	T1	D06	0	0	kleiner LLOQ	#VALUE!	1	#VALUE!
0	T1	D07	0	0	kleiner LLOQ	#VALUE!	1	#VALUE!
0	T1	D08	0	0	kleiner LLOQ	#VALUE!	1	#VALUE!
0	T1	D09	0	0	kleiner LLOQ	#VALUE!	1	#VALUE!
0	T1	D10	0	0	kleiner LLOQ	#VALUE!	1	#VALUE!
0	T1	D11	0	0	kleiner LLOQ	#VALUE!	1	#VALUE!
0	T1	D12	0	0	kleiner LLOQ	#VALUE!	1	#VALUE!

Plate: T1

	1	2	3	4	5	6	7	8	9	10	11	12
A							0.423	0.615	0.670	0.724	0.150	0.153
B											0.147	0.138
C											0.092	0.112
D												
E												
F												
G				0.911			0.064	0.054	0.052	0.056	0.051	0.055
H							0.051	0.051	0.051	0.052	0.052	0.052

gelb dil5*dil2,75=dil 13,75

Plattenbelegung

	1	2	3	4	5	6	7	8	9	10	11	12
A							T1HN2	T1HN2	T1HN3	T1HN3	T1HN4	T1HN4
B											T1HS14	T1HS14
C											T1HS24	T1HS24
D												
E												
F												
G				T1HS11WH			Blank PEG	Blank PEG	Blank PEG	Blank PEG	Blank PEG	Blank PEG
H							Blank no Peg	Blank no Peg	Blank no Peg	Blank no Peg	Blank no Peg	Blank no Peg

Comment:

Erste 4 Reihen immer Testmessung zur Evaluierung der benötigten Verdünnung. Wird nicht mit ausgewertet. Unverdünnte Messungen außerhalb der Linearität werden ebenfalls nicht ausgewertet. Dil.Faktor unverdünnte samples=2,75 (100µl sample+100µlRM+75µl stop sol.) Verdünnung (gelb) Dil.5*dil2,75=dil 13,5; rot dil 9*dil9*dil2,75=dil81

Date Operator

Date Control

0	T1	E01	0	0	kleiner LLOQ	#VALUE!	1	#VALUE!
0	T1	E02	0	0	kleiner LLOQ	#VALUE!	1	#VALUE!
0	T1	E03	0	0	kleiner LLOQ	#VALUE!	1	#VALUE!
0	T1	E04	0	0	kleiner LLOQ	#VALUE!	1	#VALUE!
0	T1	E05	0	0	kleiner LLOQ	#VALUE!	1	#VALUE!
0	T1	E06	0	0	kleiner LLOQ	#VALUE!	1	#VALUE!
0	T1	E07	0	0	kleiner LLOQ	#VALUE!	1	#VALUE!
0	T1	E08	0	0	kleiner LLOQ	#VALUE!	1	#VALUE!
0	T1	E09	0	0	kleiner LLOQ	#VALUE!	1	#VALUE!
0	T1	E10	0	0	kleiner LLOQ	#VALUE!	1	#VALUE!
0	T1	E11	0	0	kleiner LLOQ	#VALUE!	1	#VALUE!
0	T1	E12	0	0	kleiner LLOQ	#VALUE!	1	#VALUE!
0	T1	F01	0	0	kleiner LLOQ	#VALUE!	1	#VALUE!
0	T1	F02	0	0	kleiner LLOQ	#VALUE!	1	#VALUE!
0	T1	F03	0	0	kleiner LLOQ	#VALUE!	1	#VALUE!
0	T1	F04	0	0	kleiner LLOQ	#VALUE!	1	#VALUE!
0	T1	F05	0	0	kleiner LLOQ	#VALUE!	1	#VALUE!
0	T1	F06	0	0	kleiner LLOQ	#VALUE!	1	#VALUE!
0	T1	F07	0	0	kleiner LLOQ	#VALUE!	1	#VALUE!
0	T1	F08	0	0	kleiner LLOQ	#VALUE!	1	#VALUE!
0	T1	F09	0	0	kleiner LLOQ	#VALUE!	1	#VALUE!
0	T1	F10	0	0	kleiner LLOQ	#VALUE!	1	#VALUE!
0	T1	F11	0	0	kleiner LLOQ	#VALUE!	1	#VALUE!
0	T1	F12	0	0	kleiner LLOQ	#VALUE!	1	#VALUE!
0	T1	G01	0	0	kleiner LLOQ	#VALUE!	1	#VALUE!
0	T1	G02	0	0	kleiner LLOQ	#VALUE!	1	#VALUE!
0	T1	G03	0	0	kleiner LLOQ	#VALUE!	1	#VALUE!
T1HS11WH	T1	G04	0.911	0.860	0.860	0.039	81	3.181
0	T1	G05	0	0	kleiner LLOQ	#VALUE!	1	#VALUE!
0	T1	G06	0	0	kleiner LLOQ	#VALUE!	1	#VALUE!
Blank PEG	T1	G07	0	0	kleiner LLOQ	#VALUE!	2.75	#VALUE!
Blank PEG	T1	G08	0	0	kleiner LLOQ	#VALUE!	2.75	#VALUE!
Blank PEG	T1	G09	0	0	kleiner LLOQ	#VALUE!	2.75	#VALUE!
Blank PEG	T1	G10	0	0	kleiner LLOQ	#VALUE!	2.75	#VALUE!
Blank PEG	T1	G11	0	0	kleiner LLOQ	#VALUE!	2.75	#VALUE!
Blank PEG	T1	G12	0	0	kleiner LLOQ	#VALUE!	2.75	#VALUE!
0	T1	H01	0	0	kleiner LLOQ	#VALUE!	1	#VALUE!
0	T1	H02	0	0	kleiner LLOQ	#VALUE!	1	#VALUE!
0	T1	H03	0	0	kleiner LLOQ	#VALUE!	1	#VALUE!
0	T1	H04	0	0	kleiner LLOQ	#VALUE!	1	#VALUE!
0	T1	H05	0	0	kleiner LLOQ	#VALUE!	1	#VALUE!
0	T1	H06	0	0	kleiner LLOQ	#VALUE!	1	#VALUE!
Blank no Peg	T1	H07	0	0	kleiner LLOQ	#VALUE!	2.75	#VALUE!
Blank no Peg	T1	H08	0	0	kleiner LLOQ	#VALUE!	2.75	#VALUE!
Blank no Peg	T1	H09	0	0	kleiner LLOQ	#VALUE!	2.75	#VALUE!
Blank no Peg	T1	H10	0	0	kleiner LLOQ	#VALUE!	2.75	#VALUE!
Blank no Peg	T1	H11	0	0	kleiner LLOQ	#VALUE!	2.75	#VALUE!
Blank no Peg	T1	H12	0	0	kleiner LLOQ	#VALUE!	2.75	#VALUE!
0	T2	A01	0	0	kleiner LLOQ	#VALUE!	1	#VALUE!
0	T2	A02	0	0	kleiner LLOQ	#VALUE!	1	#VALUE!
0	T2	A03	0	0	kleiner LLOQ	#VALUE!	1	#VALUE!
0	T2	A04	0	0	kleiner LLOQ	#VALUE!	1	#VALUE!
0	T2	A05	0.933	0.882	0.882	0.040	22	0.887
0	T2	A06	0.908	0.857	0.857	0.039	22	0.861
T2HN2	T2	A07	0.853	0.802	0.802	0.037	22	0.804
T2HN2	T2	A08	0.834	0.784	0.784	0.036	22	0.784
0	T2	A09	0	0	kleiner LLOQ	#VALUE!	1	#VALUE!
0	T2	A10	0	0	kleiner LLOQ	#VALUE!	1	#VALUE!
T2HN4	T2	A11	0	0	kleiner LLOQ	#VALUE!	2.75	#VALUE!
T2HN4	T2	A12	0	0	kleiner LLOQ	#VALUE!	2.75	#VALUE!
0	T2	B01	0	0	kleiner LLOQ	#VALUE!	1	#VALUE!
0	T2	B02	0	0	kleiner LLOQ	#VALUE!	1	#VALUE!
0	T2	B03	0	0	kleiner LLOQ	#VALUE!	1	#VALUE!
0	T2	B04	0	0	kleiner LLOQ	#VALUE!	1	#VALUE!
0	T2	B05	0	0	kleiner LLOQ	#VALUE!	1	#VALUE!
0	T2	B06	0	0	kleiner LLOQ	#VALUE!	1	#VALUE!
0	T2	B07	0	0	kleiner LLOQ	#VALUE!	1	#VALUE!
0	T2	B08	0	0	kleiner LLOQ	#VALUE!	1	#VALUE!
0	T2	B09	0	0	kleiner LLOQ	#VALUE!	1	#VALUE!
0	T2	B10	0	0	kleiner LLOQ	#VALUE!	1	#VALUE!
T2HS14	T2	B11	0	0	kleiner LLOQ	#VALUE!	2.75	#VALUE!
T2HS14	T2	B12	0	0	kleiner LLOQ	#VALUE!	2.75	#VALUE!

Plate: T2

	1	2	3	4	5	6	7	8	9	10	11	12
A					0.933	0.908	0.853	0.834			0.140	0.136
B											0.141	0.139
C							0.855	0.843	0.911	0.882	0.117	0.113
D												
E												
F												
G	0.490	0.485					0.056	0.055	0.055	0.055	0.056	0.063
H							0.052	0.053	0.053	0.053	0.052	0.052

Plattenbelegung

	1	2	3	4	5	6	7	8	9	10	11	12
A							T2HN2	T2HN2			T2HN4	T2HN4
B											T2HS14	T2HS14
C							T2HS22	T2HS22	T2HS13	T2HS13	T2HS24	T2HS24
D												

0	T2	C01	0	0	kleiner LLOQ	#VALUE!	1	#VALUE!
0	T2	C02	0	0	kleiner LLOQ	#VALUE!	1	#VALUE!
0	T2	C03	0	0	kleiner LLOQ	#VALUE!	1	#VALUE!
0	T2	C04	0	0	kleiner LLOQ	#VALUE!	1	#VALUE!
0	T2	C05	0	0	kleiner LLOQ	#VALUE!	1	#VALUE!
0	T2	C06	0	0	kleiner LLOQ	#VALUE!	1	#VALUE!
T2HS22	T2	C07	0.855	0.804	0.804	0.037	22	0.806
T2HS22	T2	C08	0.843	0.792	0.792	0.036	22	0.793
T2HS13	T2	C09	0.911	0.860	0.860	0.039	22	0.864
T2HS13	T2	C10	0.882	0.831	0.831	0.038	22	0.834
T2HS24	T2	C11	0	0	kleiner LLOQ	#VALUE!	2.75	#VALUE!
T2HS24	T2	C12	0	0	kleiner LLOQ	#VALUE!	2.75	#VALUE!
0	T2	D01	0	0	kleiner LLOQ	#VALUE!	1	#VALUE!
0	T2	D02	0	0	kleiner LLOQ	#VALUE!	1	#VALUE!
0	T2	D03	0	0	kleiner LLOQ	#VALUE!	1	#VALUE!
0	T2	D04	0	0	kleiner LLOQ	#VALUE!	1	#VALUE!
0	T2	D05	0	0	kleiner LLOQ	#VALUE!	1	#VALUE!
0	T2	D06	0	0	kleiner LLOQ	#VALUE!	1	#VALUE!
0	T2	D07	0	0	kleiner LLOQ	#VALUE!	1	#VALUE!
0	T2	D08	0	0	kleiner LLOQ	#VALUE!	1	#VALUE!
0	T2	D09	0	0	kleiner LLOQ	#VALUE!	1	#VALUE!
0	T2	D10	0	0	kleiner LLOQ	#VALUE!	1	#VALUE!
0	T2	D11	0	0	kleiner LLOQ	#VALUE!	1	#VALUE!
0	T2	D12	0	0	kleiner LLOQ	#VALUE!	1	#VALUE!
0	T2	E01	0	0	kleiner LLOQ	#VALUE!	1	#VALUE!
0	T2	E02	0	0	kleiner LLOQ	#VALUE!	1	#VALUE!
0	T2	E03	0	0	kleiner LLOQ	#VALUE!	1	#VALUE!
0	T2	E04	0	0	kleiner LLOQ	#VALUE!	1	#VALUE!
0	T2	E05	0	0	kleiner LLOQ	#VALUE!	1	#VALUE!
0	T2	E06	0	0	kleiner LLOQ	#VALUE!	1	#VALUE!
0	T2	E07	0	0	kleiner LLOQ	#VALUE!	1	#VALUE!
0	T2	E08	0	0	kleiner LLOQ	#VALUE!	1	#VALUE!
0	T2	E09	0	0	kleiner LLOQ	#VALUE!	1	#VALUE!
0	T2	E10	0	0	kleiner LLOQ	#VALUE!	1	#VALUE!
0	T2	E11	0	0	kleiner LLOQ	#VALUE!	1	#VALUE!
0	T2	E12	0	0	kleiner LLOQ	#VALUE!	1	#VALUE!
0	T2	F01	0	0	kleiner LLOQ	#VALUE!	1	#VALUE!
0	T2	F02	0	0	kleiner LLOQ	#VALUE!	1	#VALUE!
0	T2	F03	0	0	kleiner LLOQ	#VALUE!	1	#VALUE!
0	T2	F04	0	0	kleiner LLOQ	#VALUE!	1	#VALUE!
0	T2	F05	0	0	kleiner LLOQ	#VALUE!	1	#VALUE!
0	T2	F06	0	0	kleiner LLOQ	#VALUE!	1	#VALUE!
0	T2	F07	0	0	kleiner LLOQ	#VALUE!	1	#VALUE!
0	T2	F08	0	0	kleiner LLOQ	#VALUE!	1	#VALUE!
0	T2	F09	0	0	kleiner LLOQ	#VALUE!	1	#VALUE!
0	T2	F10	0	0	kleiner LLOQ	#VALUE!	1	#VALUE!
0	T2	F11	0	0	kleiner LLOQ	#VALUE!	1	#VALUE!
0	T2	F12	0	0	kleiner LLOQ	#VALUE!	1	#VALUE!
T2HS11	T2	G01	0.490	0.439	0.439	0.019	132	2.543
T2HS11	T2	G02	0.485	0.434	0.434	0.019	132	2.509
0	T2	G03	0	0	kleiner LLOQ	#VALUE!	1	#VALUE!
0	T2	G04	0	0	kleiner LLOQ	#VALUE!	1	#VALUE!
0	T2	G05	0	0	kleiner LLOQ	#VALUE!	1	#VALUE!
0	T2	G06	0	0	kleiner LLOQ	#VALUE!	1	#VALUE!
Blank PEG	T2	G07	0	0	kleiner LLOQ	#VALUE!	2.75	#VALUE!
Blank PEG	T2	G08	0	0	kleiner LLOQ	#VALUE!	2.75	#VALUE!
Blank PEG	T2	G09	0	0	kleiner LLOQ	#VALUE!	2.75	#VALUE!
Blank PEG	T2	G10	0	0	kleiner LLOQ	#VALUE!	2.75	#VALUE!
Blank PEG	T2	G11	0	0	kleiner LLOQ	#VALUE!	2.75	#VALUE!
Blank PEG	T2	G12	0	0	kleiner LLOQ	#VALUE!	2.75	#VALUE!
0	T2	H01	0	0	kleiner LLOQ	#VALUE!	1	#VALUE!
0	T2	H02	0	0	kleiner LLOQ	#VALUE!	1	#VALUE!
0	T2	H03	0	0	kleiner LLOQ	#VALUE!	1	#VALUE!
0	T2	H04	0	0	kleiner LLOQ	#VALUE!	1	#VALUE!
0	T2	H05	0	0	kleiner LLOQ	#VALUE!	1	#VALUE!
0	T2	H06	0	0	kleiner LLOQ	#VALUE!	1	#VALUE!
Blank no PEG	T2	H07	0	0	kleiner LLOQ	#VALUE!	2.75	#VALUE!
Blank no PEG	T2	H08	0	0	kleiner LLOQ	#VALUE!	2.75	#VALUE!
Blank no PEG	T2	H09	0	0	kleiner LLOQ	#VALUE!	2.75	#VALUE!
Blank no PEG	T2	H10	0	0	kleiner LLOQ	#VALUE!	2.75	#VALUE!
Blank no PEG	T2	H11	0	0	kleiner LLOQ	#VALUE!	2.75	#VALUE!
Blank no PEG	T2	H12	0	0	kleiner LLOQ	#VALUE!	2.75	#VALUE!
0	T3	A01	0	0	kleiner LLOQ	#VALUE!	1	#VALUE!
0	T3	A02	0	0	kleiner LLOQ	#VALUE!	1	#VALUE!
0	T3	A03	0	0	kleiner LLOQ	#VALUE!	1	#VALUE!
0	T3	A04	0	0	kleiner LLOQ	#VALUE!	1	#VALUE!

E												
F												
G	T2HS11	T2HS11					Blank PEG	Blank PEG	Blank PEG	Blank PEG	Blank PEG	Blank PEG
H							Blank no PEG	Blank no PEG	Blank no PEG	Blank no PEG	Blank no PEG	Blank no PEG

Dil faktor 8*2,75=dil.22
Dil faktor 8*6*2,75=dil.132

Comment:

Date Operator

Date Control

T3HN1	T3	A05	0.460	0.409	0.409	0.018	2.75	0.049
T3HN1	T3	A06	0.524	0.473	0.473	0.021	2.75	0.057
T3HN2	T3	A07	0.298	0.247	0.247	0.010	2.75	0.028
T3HN2	T3	A08	0.342	0.291	0.291	0.012	2.75	0.034
T3HN3	T3	A09	0.392	0.341	0.341	0.015	2.75	0.040
T3HN3	T3	A10	0.394	0.343	0.343	0.015	2.75	0.040
T3HN4	T3	A11	0	0	kleiner LLOQ	#VALUE!	1	#VALUE!
T3HN4	T3	A12	0	0	kleiner LLOQ	#VALUE!	1	#VALUE!
0	T3	B01	0	0	kleiner LLOQ	#VALUE!	1	#VALUE!
0	T3	B02	0	0	kleiner LLOQ	#VALUE!	1	#VALUE!
0	T3	B03	0	0	kleiner LLOQ	#VALUE!	1	#VALUE!
0	T3	B04	0	0	kleiner LLOQ	#VALUE!	1	#VALUE!
0	T3	B05	0	0	kleiner LLOQ	#VALUE!	1	#VALUE!
0	T3	B06	0	0	kleiner LLOQ	#VALUE!	1	#VALUE!
0	T3	B07	0	0	kleiner LLOQ	#VALUE!	1	#VALUE!
0	T3	B08	0	0	kleiner LLOQ	#VALUE!	1	#VALUE!
0	T3	B09	0	0	kleiner LLOQ	#VALUE!	1	#VALUE!
0	T3	B10	0	0	kleiner LLOQ	#VALUE!	1	#VALUE!
T3HS14	T3	B11	0.144	0.093	kleiner LLOQ	#VALUE!	2.75	#VALUE!
T3HS14	T3	B12	0.148	0.098	kleiner LLOQ	#VALUE!	2.75	#VALUE!
0	T3	C01	0	0	kleiner LLOQ	#VALUE!	1	#VALUE!
0	T3	C02	0	0	kleiner LLOQ	#VALUE!	1	#VALUE!
0	T3	C03	0	0	kleiner LLOQ	#VALUE!	1	#VALUE!
0	T3	C04	0	0	kleiner LLOQ	#VALUE!	1	#VALUE!
T3HS21	T3	C05	0.766	0.715	0.715	0.032	24.75	0.802
T3HS21	T3	C06	0.765	0.714	0.714	0.032	24.75	0.801
T3HS22	T3	C07	0.780	0.729	0.729	0.033	24.75	0.818
T3HS22	T3	C08	0.796	0.745	0.745	0.034	24.75	0.836
T3HS23	T3	C09	0.634	0.583	0.583	0.026	24.75	0.646
T3HS23	T3	C10	0.647	0.596	0.596	0.027	24.75	0.662
T3HS24	T3	C11	0.110	0.060	kleiner LLOQ	#VALUE!	1	#VALUE!
T3HS24	T3	C12	0.121	0.070	kleiner LLOQ	#VALUE!	1	#VALUE!
0	T3	D01	0	0	kleiner LLOQ	#VALUE!	1	#VALUE!
0	T3	D02	0	0	kleiner LLOQ	#VALUE!	1	#VALUE!
0	T3	D03	0	0	kleiner LLOQ	#VALUE!	1	#VALUE!
0	T3	D04	0	0	kleiner LLOQ	#VALUE!	1	#VALUE!
0	T3	D05	0	0	kleiner LLOQ	#VALUE!	1	#VALUE!
0	T3	D06	0	0	kleiner LLOQ	#VALUE!	1	#VALUE!
0	T3	D07	0	0	kleiner LLOQ	#VALUE!	1	#VALUE!
0	T3	D08	0	0	kleiner LLOQ	#VALUE!	1	#VALUE!
0	T3	D09	0	0	kleiner LLOQ	#VALUE!	1	#VALUE!
0	T3	D10	0	0	kleiner LLOQ	#VALUE!	1	#VALUE!
0	T3	D11	0	0	kleiner LLOQ	#VALUE!	1	#VALUE!
0	T3	D12	0	0	kleiner LLOQ	#VALUE!	1	#VALUE!
0	T3	E01	0	0	kleiner LLOQ	#VALUE!	1	#VALUE!
0	T3	E02	0	0	kleiner LLOQ	#VALUE!	1	#VALUE!
0	T3	E03	0	0	kleiner LLOQ	#VALUE!	1	#VALUE!
0	T3	E04	0	0	kleiner LLOQ	#VALUE!	1	#VALUE!
0	T3	E05	0	0	kleiner LLOQ	#VALUE!	1	#VALUE!
0	T3	E06	0	0	kleiner LLOQ	#VALUE!	1	#VALUE!
0	T3	E07	0	0	kleiner LLOQ	#VALUE!	1	#VALUE!
0	T3	E08	0	0	kleiner LLOQ	#VALUE!	1	#VALUE!
0	T3	E09	0	0	kleiner LLOQ	#VALUE!	1	#VALUE!
0	T3	E10	0	0	kleiner LLOQ	#VALUE!	1	#VALUE!
0	T3	E11	0	0	kleiner LLOQ	#VALUE!	1	#VALUE!
0	T3	E12	0	0	kleiner LLOQ	#VALUE!	1	#VALUE!
0	T3	F01	0	0	kleiner LLOQ	#VALUE!	1	#VALUE!
0	T3	F02	0	0	kleiner LLOQ	#VALUE!	1	#VALUE!
0	T3	F03	0	0	kleiner LLOQ	#VALUE!	1	#VALUE!
0	T3	F04	0	0	kleiner LLOQ	#VALUE!	1	#VALUE!
0	T3	F05	0	0	kleiner LLOQ	#VALUE!	1	#VALUE!
0	T3	F06	0	0	kleiner LLOQ	#VALUE!	1	#VALUE!
0	T3	F07	0	0	kleiner LLOQ	#VALUE!	1	#VALUE!
0	T3	F08	0	0	kleiner LLOQ	#VALUE!	1	#VALUE!
0	T3	F09	0	0	kleiner LLOQ	#VALUE!	1	#VALUE!
0	T3	F10	0	0	kleiner LLOQ	#VALUE!	1	#VALUE!
0	T3	F11	0	0	kleiner LLOQ	#VALUE!	1	#VALUE!
0	T3	F12	0	0	kleiner LLOQ	#VALUE!	1	#VALUE!
0	T3	G01	0	0	kleiner LLOQ	#VALUE!	1	#VALUE!
0	T3	G02	0	0	kleiner LLOQ	#VALUE!	1	#VALUE!
0	T3	G03	0	0	kleiner LLOQ	#VALUE!	1	#VALUE!
0	T3	G04	0	0	kleiner LLOQ	#VALUE!	1	#VALUE!
0	T3	G05	0	0	kleiner LLOQ	#VALUE!	1	#VALUE!
0	T3	G06	0	0	kleiner LLOQ	#VALUE!	1	#VALUE!
Blank PEG	T3	G07	0	0	kleiner LLOQ	#VALUE!	2.75	#VALUE!
Blank PEG	T3	G08	0	0	kleiner LLOQ	#VALUE!	2.75	#VALUE!

Plate: T3

	1	2	3	4	5	6	7	8	9	10	11	12
A					0.460	0.524	0.298	0.342	0.392	0.394	0.138	0.152
B											0.144	0.148
C					0.766	0.765	0.780	0.796	0.634	0.647	0.110	0.121
D												
E												
F												
G							0.052	0.048	0.052	0.052	0.047	0.046
H							0.051	0.060	0.059	0.054	0.055	0.055

Plattenbelegung

	1	2	3	4	5	6	7	8	9	10	11	12
A					T3HN1	T3HN1	T3HN2	T3HN2	T3HN3	T3HN3	T3HN4	T3HN4
B											T3HS14	T3HS14
C					T3HS21	T3HS21	T3HS22	T3HS22	T3HS23	T3HS23	T3HS24	T3HS24
D												
E												
F												
G							Blank PEG	Blank PEG	Blank PEG	Blank PEG	Blank PEG	Blank PEG
H							Blank no PEG	Blank no PEG	Blank no PEG	Blank no PEG	Blank no PEG	Blank no PEG

dil9*2,75=dil24,75

Comment:

Date Operator

Date Control

Blank PEG	T3	G09	0	0	kleiner LLOQ	#VALUE!	2.75	#VALUE!
Blank PEG	T3	G10	0	0	kleiner LLOQ	#VALUE!	2.75	#VALUE!
Blank PEG	T3	G11	0	0	kleiner LLOQ	#VALUE!	2.75	#VALUE!
Blank PEG	T3	G12	0	0	kleiner LLOQ	#VALUE!	2.75	#VALUE!
0	T3	H01	0	0	kleiner LLOQ	#VALUE!	1	#VALUE!
0	T3	H02	0	0	kleiner LLOQ	#VALUE!	1	#VALUE!
0	T3	H03	0	0	kleiner LLOQ	#VALUE!	1	#VALUE!
0	T3	H04	0	0	kleiner LLOQ	#VALUE!	1	#VALUE!
0	T3	H05	0	0	kleiner LLOQ	#VALUE!	1	#VALUE!
0	T3	H06	0	0	kleiner LLOQ	#VALUE!	1	#VALUE!
Blank no PEG	T3	H07	0	0	kleiner LLOQ	#VALUE!	2.75	#VALUE!
Blank no PEG	T3	H08	0	0	kleiner LLOQ	#VALUE!	2.75	#VALUE!
Blank no PEG	T3	H09	0	0	kleiner LLOQ	#VALUE!	2.75	#VALUE!
Blank no PEG	T3	H10	0	0	kleiner LLOQ	#VALUE!	2.75	#VALUE!
Blank no PEG	T3	H11	0	0	kleiner LLOQ	#VALUE!	2.75	#VALUE!
Blank no PEG	T3	H12	0	0	kleiner LLOQ	#VALUE!	2.75	#VALUE!

Messdaten (diese Tabelle in Bericht übernehmen)

Sample name *	concentration (theor.) *	measured data	measured data	measured data	mean measured	SD	RSD	Blank *	measured data after * Blank subtraction	concentration (calc.) *	Deviation *	Residuen
	[µg/mL]	[AU]	[AU]	[AU]	[AU]	[AU]	[%]	[AU]		[µg/mL]	[%]	
KLP1	0.148	1.579	1.652	1.627	1.62	0.03	1.87	0.047	1.568	0.148	-0.24	0.00
KLP2	0.114	1.357	1.443	1.372	1.39	0.04	2.70	0.054	1.340	0.110	-3.40	0.00
KLP3	0.074	1.272	1.193	1.228	1.23	0.03	2.63	0.051	1.180	0.084	13.04	0.01
KLP4	0.041	0.959	0.972	0.883	0.94	0.04	4.19	0.051	0.887	0.035	-13.32	-0.01
KLP5								0.052				
KLP6								0.051				
KLP7												
KLP8												

Statistical data

Geradensteigung	Slope	m	6.05
Y-Achsenabschnitt	Y-Intercept	b	0.67
Standardabw. Geradensteigung	SD-Slope	s_m	0.620291227
Standardabw. Achsenabschnittes	SD-Y-Intercept	s_b	0.063694294
Anzahl Messpunkte	number of measuring points	n	4
Quadratsumme	sum of squares	Qxx	0.006578604
Bereichsmitte			0.094336926
Freiheitsgrade	degree of freedom	f	2
Student-t-Faktor für (P = 95 %; f = n-2)	Student-t-factor for (P = 95 %; f = n-2)	t	4.303
Vertrauensbereich Steig. (95 %) Obergrenze		$m + VB_m$	8.722319527
Vertrauensbereich Steig. (95 %) Untergrenze		$m - VB_m$	3.384093228
Vertrauensbereich Achsenabschnitt (95 %) Og.		$b + Vb_b$	0.946914615
Vertrauensbereich Achsenabschnitt (95 %) Ug.		$b - Vb_b$	0.398761518
Korrelationskoeffizient	correlation coefficient	r	0.9897
Bestimmtheitsmaß	determiantion coefficient	r^2	0.9794
Reststandardabweichung		s_0	0.050310948
Summe Restquadrate		sd	2.895583345
Verfahrensstandardabw.		s_{x0}	0.008311454
Rel. Verfahrensstandardabw. %		V_{x0}	8.810393374

mean Blank	**0**	
SD Blank	0.00	
RSD Blank	3.79	x=
x*SD (LLOQ)	0	5
x*SD (LOD)	0	3
**LLOQ (AU)**	**0**	
**LOD (AU)**	**0**	
ULOQ	1.568	
LLOQ (Lin)	0.887	

Evaluation / Comment

LDH linearity valid with 7 standards, split in lin high and lin low, each with 4 standards and KLP4 common standard for both. R² 0,9981, deviations for both between -13.34% and +13.04%

Date	Operator	Date	Control

Figure 1 **Linearity**

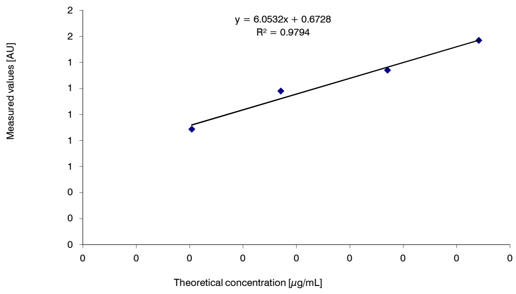

Figure 2 **Method validation Residuen Plot**

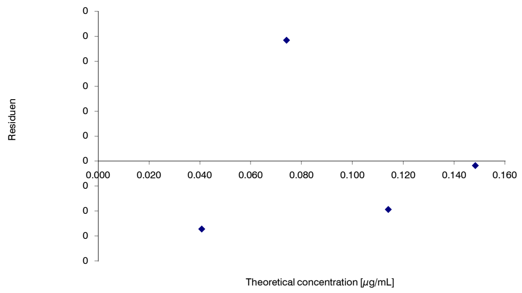

Evaluation / Comment

lin passed

Date	Operator	Date	Control

Sample name	Plate	Position	[AU]	[AU]-Blank	Rating	[µg/mL]	Dilution factor	[µg/mL]
0	T0	A01	0	0	kleiner LLOQ	#VALUE!	1	#VALUE!
0	T0	A02	0	0	kleiner LLOQ	#VALUE!	1	#VALUE!
0	T0	A03	0	0	kleiner LLOQ	#VALUE!	1	#VALUE!
0	T0	A04	0	0	kleiner LLOQ	#VALUE!	1	#VALUE!
T0HN1	T0	A05	1.126	1.075	1.075	0.066	88	5.847
T0HN1	T0	A06	1.411	1.360	1.360	0.114	88	9.991
T0HN2	T0	A07	0	0	kleiner LLOQ	#VALUE!	1	#VALUE!
T0HN2	T0	A08	0	0	kleiner LLOQ	#VALUE!	1	#VALUE!
T0HN3	T0	A09	1.266	1.215	1.215	0.090	88	7.883
T0HN3	T0	A10	1.141	1.090	1.090	0.069	88	6.066
T0HN4	T0	A11	0	0	kleiner LLOQ	#VALUE!	1	#VALUE!
T0HN4	T0	A12	0	0	kleiner LLOQ	#VALUE!	1	#VALUE!
0	T0	B01	0	0	kleiner LLOQ	#VALUE!	1	#VALUE!
0	T0	B02	0	0	kleiner LLOQ	#VALUE!	1	#VALUE!
0	T0	B03	0	0	kleiner LLOQ	#VALUE!	1	#VALUE!
0	T0	B04	0	0	kleiner LLOQ	#VALUE!	1	#VALUE!
T0HS11	T0	B05	0	0	kleiner LLOQ	#VALUE!	1	#VALUE!
T0HS11	T0	B06	1.610	1.559	1.559	0.146	88	12.884
T0HS12	T0	B07	1.471	1.420	1.420	0.123	88	10.863
T0HS12	T0	B08	1.476	1.425	1.425	0.124	88	10.936
T0HS13	T0	B09	0	0	kleiner LLOQ	#VALUE!	1	#VALUE!
T0HS13	T0	B10	0	0	kleiner LLOQ	#VALUE!	1	#VALUE!
T0HS14	T0	B11	0	0	kleiner LLOQ	#VALUE!	1	#VALUE!
T0HS14	T0	B12	0	0	kleiner LLOQ	#VALUE!	1	#VALUE!
0	T0	C01	0	0	kleiner LLOQ	#VALUE!	1	#VALUE!
0	T0	C02	0	0	kleiner LLOQ	#VALUE!	1	#VALUE!
0	T0	C03	0	0	kleiner LLOQ	#VALUE!	1	#VALUE!
0	T0	C04	0	0	kleiner LLOQ	#VALUE!	1	#VALUE!
T0HS21	T0	C05	1.619	1.568	1.568	0.148	88	13.015
T0HS21	T0	C06	1.359	1.308	1.308	0.105	88	9.235
T0HS22	T0	C07	1.118	1.067	1.067	0.065	88	5.731
T0HS22	T0	C08	1.137	1.086	1.086	0.068	88	6.007
T0HS23	T0	C09	1.055	1.004	1.004	0.055	88	4.815
T0HS23	T0	C10	0.994	0.943	0.943	0.045	88	3.928
T0HS24	T0	C11	0	0	kleiner LLOQ	#VALUE!	1	#VALUE!
T0HS24	T0	C12	0	0	kleiner LLOQ	#VALUE!	1	#VALUE!
0	T0	D01	0	0	kleiner LLOQ	#VALUE!	1	#VALUE!
0	T0	D02	0	0	kleiner LLOQ	#VALUE!	1	#VALUE!
0	T0	D03	0	0	kleiner LLOQ	#VALUE!	1	#VALUE!
0	T0	D04	0	0	kleiner LLOQ	#VALUE!	1	#VALUE!
0	T0	D05	0	0	kleiner LLOQ	#VALUE!	1	#VALUE!
0	T0	D06	0	0	kleiner LLOQ	#VALUE!	1	#VALUE!
0	T0	D07	0	0	kleiner LLOQ	#VALUE!	1	#VALUE!
0	T0	D08	0	0	kleiner LLOQ	#VALUE!	1	#VALUE!
0	T0	D09	0	0	kleiner LLOQ	#VALUE!	1	#VALUE!
0	T0	D10	0	0	kleiner LLOQ	#VALUE!	1	#VALUE!
0	T0	D11	0	0	kleiner LLOQ	#VALUE!	1	#VALUE!
0	T0	D12	0	0	kleiner LLOQ	#VALUE!	1	#VALUE!

Plate: T0

	1	2	3	4	5	6	7	8	9	10	11	12
A					1.126	1.411			1.266	1.141		
B						1.610	1.471	1.476				
C					1.619	1.359	1.118	1.137	1.055	0.994		
D												
E												
F												
G							0.061	0.065	0.056	0.055	0.055	0.057
H							0.051	0.05	0.051	0.05	0.05	0.049

Plattenbelegung

	1	2	3	4	5	6	7	8	9	10	11	12
A					T0HN1	T0HN1	T0HN2	T0HN2	T0HN3	T0HN3	T0HN4	T0HN4
B					T0HS11	T0HS11	T0HS12	T0HS12	T0HS13	T0HS13	T0HS14	T0HS14
C					T0HS21	T0HS21	T0HS22	T0HS22	T0HS23	T0HS23	T0HS24	T0HS24
D												
E												
F												
G							Blank PEG	Blank PEG	Blank PEG	Blank PEG	Blank PEG	Blank PEG
H							Blank no PEG	Blank no PEG	Blank no PEG	Blank no PEG	Blank no PEG	Blank no PEG

Comment:

Erste 4 Reihen immer Testmessung zur Evaluierung der benötigten Verdünnung. Wird nicht mit ausgewertet. Unverdünnte Messungen außerhalb der Linearität werden ebenfalls nicht ausgewertet. Dil.Faktor unverdünnte samples=2,75 (100µl sample+100µlRM+75µl stop sol.) Verdünnung Dil.8 Dil8*Dil2,75=Dil.22, Rot= 2* verdünnt, Dil4 und Dil 8, also Dil 2,75*Dil8*Dil4=Dil88

Samples aufgeteilt in lin high und low:niedriger konzentrierte Samples bei lin low zu finden.

Date Operator

Date Control

0	T0	E01	0	0	kleiner LLOQ	#VALUE!	1	#VALUE!
0	T0	E02	0	0	kleiner LLOQ	#VALUE!	1	#VALUE!
0	T0	E03	0	0	kleiner LLOQ	#VALUE!	1	#VALUE!
0	T0	E04	0	0	kleiner LLOQ	#VALUE!	1	#VALUE!
0	T0	E05	0	0	kleiner LLOQ	#VALUE!	1	#VALUE!
0	T0	E06	0	0	kleiner LLOQ	#VALUE!	1	#VALUE!
0	T0	E07	0	0	kleiner LLOQ	#VALUE!	1	#VALUE!
0	T0	E08	0	0	kleiner LLOQ	#VALUE!	1	#VALUE!
0	T0	E09	0	0	kleiner LLOQ	#VALUE!	1	#VALUE!
0	T0	E10	0	0	kleiner LLOQ	#VALUE!	1	#VALUE!
0	T0	E11	0	0	kleiner LLOQ	#VALUE!	1	#VALUE!
0	T0	E12	0	0	kleiner LLOQ	#VALUE!	1	#VALUE!
0	T0	F01	0	0	kleiner LLOQ	#VALUE!	1	#VALUE!
0	T0	F02	0	0	kleiner LLOQ	#VALUE!	1	#VALUE!
0	T0	F03	0	0	kleiner LLOQ	#VALUE!	1	#VALUE!
0	T0	F04	0	0	kleiner LLOQ	#VALUE!	1	#VALUE!
0	T0	F05	0	0	kleiner LLOQ	#VALUE!	1	#VALUE!
0	T0	F06	0	0	kleiner LLOQ	#VALUE!	1	#VALUE!
0	T0	F07	0	0	kleiner LLOQ	#VALUE!	1	#VALUE!
0	T0	F08	0	0	kleiner LLOQ	#VALUE!	1	#VALUE!
0	T0	F09	0	0	kleiner LLOQ	#VALUE!	1	#VALUE!
0	T0	F10	0	0	kleiner LLOQ	#VALUE!	1	#VALUE!
0	T0	F11	0	0	kleiner LLOQ	#VALUE!	1	#VALUE!
0	T0	F12	0	0	kleiner LLOQ	#VALUE!	1	#VALUE!
0	T0	G01	0	0	kleiner LLOQ	#VALUE!	1	#VALUE!
0	T0	G02	0	0	kleiner LLOQ	#VALUE!	1	#VALUE!
0	T0	G03	0	0	kleiner LLOQ	#VALUE!	1	#VALUE!
0	T0	G04	0	0	kleiner LLOQ	#VALUE!	1	#VALUE!
0	T0	G05	0	0	kleiner LLOQ	#VALUE!	1	#VALUE!
0	T0	G06	0	0	kleiner LLOQ	#VALUE!	1	#VALUE!
Blank PEG	T0	G07	0.061	0.010	kleiner LLOQ	#VALUE!	2.75	#VALUE!
Blank PEG	T0	G08	0.065	0.014	kleiner LLOQ	#VALUE!	2.75	#VALUE!
Blank PEG	T0	G09	0.056	0.005	kleiner LLOQ	#VALUE!	2.75	#VALUE!
Blank PEG	T0	G10	0.055	0.004	kleiner LLOQ	#VALUE!	2.75	#VALUE!
Blank PEG	T0	G11	0.055	0.004	kleiner LLOQ	#VALUE!	2.75	#VALUE!
Blank PEG	T0	G12	0.057	0.006	kleiner LLOQ	#VALUE!	2.75	#VALUE!
0	T0	H01	0	0	kleiner LLOQ	#VALUE!	1	#VALUE!
0	T0	H02	0	0	kleiner LLOQ	#VALUE!	1	#VALUE!
0	T0	H03	0	0	kleiner LLOQ	#VALUE!	1	#VALUE!
0	T0	H04	0	0	kleiner LLOQ	#VALUE!	1	#VALUE!
0	T0	H05	0	0	kleiner LLOQ	#VALUE!	1	#VALUE!
0	T0	H06	0	0	kleiner LLOQ	#VALUE!	1	#VALUE!
Blank no PEG	T0	H07	0.051	0.000	kleiner LLOQ	#VALUE!	2.75	#VALUE!
Blank no PEG	T0	H08	0.050	-0.001	kleiner LLOQ	#VALUE!	2.75	#VALUE!
Blank no PEG	T0	H09	0.051	0.000	kleiner LLOQ	#VALUE!	2.75	#VALUE!
Blank no PEG	T0	H10	0.050	-0.001	kleiner LLOQ	#VALUE!	2.75	#VALUE!
Blank no PEG	T0	H11	0.050	-0.001	kleiner LLOQ	#VALUE!	2.75	#VALUE!
Blank no PEG	T0	H12	0.049	-0.002	kleiner LLOQ	#VALUE!	2.75	#VALUE!

0	T1	A01	0	0	kleiner LLOQ	#VALUE!	1	#VALUE!
0	T1	A02	0	0	kleiner LLOQ	#VALUE!	1	#VALUE!
0	T1	A03	0	0	kleiner LLOQ	#VALUE!	1	#VALUE!
0	T1	A04	0	0	kleiner LLOQ	#VALUE!	1	#VALUE!
T1HN1	T1	A05	1.200	1.149	1.149	0.079	2.75	0.216
T1HN1	T1	A06	1.072	1.021	1.021	0.058	2.75	0.158
0	T1	A07	0	0	kleiner LLOQ	#VALUE!	1	#VALUE!
0	T1	A08	0	0	kleiner LLOQ	#VALUE!	1	#VALUE!
0	T1	A09	1.266	1.215	1.215	0.090	2.75	0.246
0	T1	A10	1.141	1.090	1.090	0.069	2.75	0.190
0	T1	A11	0	0	kleiner LLOQ	#VALUE!	1	#VALUE!
0	T1	A12	0	0	kleiner LLOQ	#VALUE!	1	#VALUE!
0	T1	B01	0	0	kleiner LLOQ	#VALUE!	1	#VALUE!
0	T1	B02	0	0	kleiner LLOQ	#VALUE!	1	#VALUE!
0	T1	B03	0	0	kleiner LLOQ	#VALUE!	1	#VALUE!
0	T1	B04	0	0	kleiner LLOQ	#VALUE!	1	#VALUE!
T1HS11	T1	B05	0	0	kleiner LLOQ	#VALUE!	1	#VALUE!
T1HS11	T1	B06	0	0	kleiner LLOQ	#VALUE!	1	#VALUE!
T1HS12	T1	B07	1.575	1.524	1.524	0.141	2.75	0.387
T1HS12	T1	B08	1.474	1.423	1.423	0.124	2.75	0.341
T1HS13	T1	B09	1.325	1.274	1.274	0.099	2.75	0.273
T1HS13	T1	B10	1.299	1.248	1.248	0.095	2.75	0.261
0	T1	B11	0	0	kleiner LLOQ	#VALUE!	1	#VALUE!
0	T1	B12	0	0	kleiner LLOQ	#VALUE!	1	#VALUE!
0	T1	C01	0	0	kleiner LLOQ	#VALUE!	1	#VALUE!
0	T1	C02	0	0	kleiner LLOQ	#VALUE!	1	#VALUE!
0	T1	C03	0	0	kleiner LLOQ	#VALUE!	1	#VALUE!
0	T1	C04	0	0	kleiner LLOQ	#VALUE!	1	#VALUE!
T1HS21	T1	C05	1.216	1.165	1.165	0.081	2.75	0.223
T1HS21	T1	C06	1.428	1.377	1.377	0.116	2.75	0.320
T1HS22	T1	C07	1.153	1.102	1.102	0.071	2.75	0.195
T1HS22	T1	C08	1.237	1.186	1.186	0.085	2.75	0.233
T1HS23	T1	C09	1.217	1.166	1.166	0.082	2.75	0.224
T1HS23	T1	C10	1.351	1.300	1.300	0.104	2.75	0.285
0	T1	C11	0	0	kleiner LLOQ	#VALUE!	1	#VALUE!
0	T1	C12	0	0	kleiner LLOQ	#VALUE!	1	#VALUE!
0	T1	D01	0	0	kleiner LLOQ	#VALUE!	1	#VALUE!
0	T1	D02	0	0	kleiner LLOQ	#VALUE!	1	#VALUE!
0	T1	D03	0	0	kleiner LLOQ	#VALUE!	1	#VALUE!
0	T1	D04	0	0	kleiner LLOQ	#VALUE!	1	#VALUE!
0	T1	D05	0	0	kleiner LLOQ	#VALUE!	1	#VALUE!
0	T1	D06	0	0	kleiner LLOQ	#VALUE!	1	#VALUE!
0	T1	D07	0	0	kleiner LLOQ	#VALUE!	1	#VALUE!
0	T1	D08	0	0	kleiner LLOQ	#VALUE!	1	#VALUE!
0	T1	D09	0	0	kleiner LLOQ	#VALUE!	1	#VALUE!
0	T1	D10	0	0	kleiner LLOQ	#VALUE!	1	#VALUE!
0	T1	D11	0	0	kleiner LLOQ	#VALUE!	1	#VALUE!
0	T1	D12	0	0	kleiner LLOQ	#VALUE!	1	#VALUE!

Plate: T1

	1	2	3	4	5	6	7	8	9	10	11	12
A					1.200	1.072			1.266	1.141		
B							1.575	1.474	1.325	1.299		
C					1.216	1.428	1.153	1.237	1.217	1.351		
D												
E												
F												
G			0.998				0.064	0.054	0.052	0.056	0.051	0.055
H							0.051	0.051	0.051	0.052	0.052	0.052

Plattenbelegung

	1	2	3	4	5	6	7	8	9	10	11	12
A					T1HN1	T1HN1						
B					T1HS11	T1HS11	T1HS12	T1HS12	T1HS13	T1HS13		
C					T1HS21	T1HS21	T1HS22	T1HS22	T1HS23	T1HS23		
D												
E												
F												
G			T1HS11WH	T1HS11WH			Blank PEG	Blank PEG	Blank PEG	Blank PEG	Blank PEG	Blank PEG
H							Blank no Peg	Blank no Peg	Blank no Peg	Blank no Peg	Blank no Peg	Blank no Peg

Comment:

Erste 4 Reihen immer Testmessung zur Evaluierung der benötigten Verdünnung. Wird nicht mit ausgewertet. Unverdünnte Messungen außerhalb der Linearität werden ebenfalls nicht ausgewertet. Dil.Faktor unverdünnte samples=2,75 (100µl sample+100µlRM+75µl stop sol.) Verdünnung (gelb) Dil.5*dil2,75=dil 13,5; rot dil 9*dil9*dil2,75=dil81

Date Operator

Date Control

0	T1	E01	0	0	kleiner LLOQ	#VALUE!	1	#VALUE!
0	T1	E02	0	0	kleiner LLOQ	#VALUE!	1	#VALUE!
0	T1	E03	0	0	kleiner LLOQ	#VALUE!	1	#VALUE!
0	T1	E04	0	0	kleiner LLOQ	#VALUE!	1	#VALUE!
0	T1	E05	0	0	kleiner LLOQ	#VALUE!	1	#VALUE!
0	T1	E06	0	0	kleiner LLOQ	#VALUE!	1	#VALUE!
0	T1	E07	0	0	kleiner LLOQ	#VALUE!	1	#VALUE!
0	T1	E08	0	0	kleiner LLOQ	#VALUE!	1	#VALUE!
0	T1	E09	0	0	kleiner LLOQ	#VALUE!	1	#VALUE!
0	T1	E10	0	0	kleiner LLOQ	#VALUE!	1	#VALUE!
0	T1	E11	0	0	kleiner LLOQ	#VALUE!	1	#VALUE!
0	T1	E12	0	0	kleiner LLOQ	#VALUE!	1	#VALUE!
0	T1	F01	0	0	kleiner LLOQ	#VALUE!	1	#VALUE!
0	T1	F02	0	0	kleiner LLOQ	#VALUE!	1	#VALUE!
0	T1	F03	0	0	kleiner LLOQ	#VALUE!	1	#VALUE!
0	T1	F04	0	0	kleiner LLOQ	#VALUE!	1	#VALUE!
0	T1	F05	0	0	kleiner LLOQ	#VALUE!	1	#VALUE!
0	T1	F06	0	0	kleiner LLOQ	#VALUE!	1	#VALUE!
0	T1	F07	0	0	kleiner LLOQ	#VALUE!	1	#VALUE!
0	T1	F08	0	0	kleiner LLOQ	#VALUE!	1	#VALUE!
0	T1	F09	0	0	kleiner LLOQ	#VALUE!	1	#VALUE!
0	T1	F10	0	0	kleiner LLOQ	#VALUE!	1	#VALUE!
0	T1	F11	0	0	kleiner LLOQ	#VALUE!	1	#VALUE!
0	T1	F12	0	0	kleiner LLOQ	#VALUE!	1	#VALUE!
0	T1	G01	0	0	kleiner LLOQ	#VALUE!	1	#VALUE!
0	T1	G02	0	0	kleiner LLOQ	#VALUE!	1	#VALUE!
T1HS11WH	T1	G03	0.998	0.947	0.947	0.045	81	3.671
T1HS11WH	T1	G04	0	0	kleiner LLOQ	#VALUE!	1	#VALUE!
0	T1	G05	0	0	kleiner LLOQ	#VALUE!	1	#VALUE!
0	T1	G06	0	0	kleiner LLOQ	#VALUE!	1	#VALUE!
Blank PEG	T1	G07	0	0	kleiner LLOQ	#VALUE!	2.75	#VALUE!
Blank PEG	T1	G08	0	0	kleiner LLOQ	#VALUE!	2.75	#VALUE!
Blank PEG	T1	G09	0	0	kleiner LLOQ	#VALUE!	2.75	#VALUE!
Blank PEG	T1	G10	0	0	kleiner LLOQ	#VALUE!	2.75	#VALUE!
Blank PEG	T1	G11	0	0	kleiner LLOQ	#VALUE!	2.75	#VALUE!
Blank PEG	T1	G12	0	0	kleiner LLOQ	#VALUE!	2.75	#VALUE!
0	T1	H01	0	0	kleiner LLOQ	#VALUE!	1	#VALUE!
0	T1	H02	0	0	kleiner LLOQ	#VALUE!	1	#VALUE!
0	T1	H03	0	0	kleiner LLOQ	#VALUE!	1	#VALUE!
0	T1	H04	0	0	kleiner LLOQ	#VALUE!	1	#VALUE!
0	T1	H05	0	0	kleiner LLOQ	#VALUE!	1	#VALUE!
0	T1	H06	0	0	kleiner LLOQ	#VALUE!	1	#VALUE!
Blank no Peg	T1	H07	0	0	kleiner LLOQ	#VALUE!	2.75	#VALUE!
Blank no Peg	T1	H08	0	0	kleiner LLOQ	#VALUE!	2.75	#VALUE!
Blank no Peg	T1	H09	0	0	kleiner LLOQ	#VALUE!	2.75	#VALUE!
Blank no Peg	T1	H10	0	0	kleiner LLOQ	#VALUE!	2.75	#VALUE!
Blank no Peg	T1	H11	0	0	kleiner LLOQ	#VALUE!	2.75	#VALUE!
Blank no Peg	T1	H12	0	0	kleiner LLOQ	#VALUE!	2.75	#VALUE!
0	T2	A01	0	0	kleiner LLOQ	#VALUE!	1	#VALUE!
0	T2	A02	0	0	kleiner LLOQ	#VALUE!	1	#VALUE!
0	T2	A03	0	0	kleiner LLOQ	#VALUE!	1	#VALUE!
0	T2	A04	0	0	kleiner LLOQ	#VALUE!	1	#VALUE!
T2HN1	T2	A05	0	0	kleiner LLOQ	#VALUE!	1	#VALUE!
T2HN1	T2	A06	0	0	kleiner LLOQ	#VALUE!	1	#VALUE!
0	T2	A07	0	0	kleiner LLOQ	#VALUE!	1	#VALUE!
0	T2	A08	0	0	kleiner LLOQ	#VALUE!	1	#VALUE!
T2HN3	T2	A09	1.078	1.027	1.027	0.059	22	1.289
T2HN3	T2	A10	1.053	1.003	1.003	0.054	22	1.198
0	T2	A11	0	0	kleiner LLOQ	#VALUE!	1	#VALUE!
0	T2	A12	0	0	kleiner LLOQ	#VALUE!	1	#VALUE!
0	T2	B01	0	0	kleiner LLOQ	#VALUE!	1	#VALUE!
0	T2	B02	0	0	kleiner LLOQ	#VALUE!	1	#VALUE!
0	T2	B03	0	0	kleiner LLOQ	#VALUE!	1	#VALUE!
0	T2	B04	0	0	kleiner LLOQ	#VALUE!	1	#VALUE!
0	T2	B05	0	0	kleiner LLOQ	#VALUE!	1	#VALUE!
0	T2	B06	0	0	kleiner LLOQ	#VALUE!	1	#VALUE!
T2HS12	T2	B07	1.246	1.195	1.195	0.086	22	1.897
T2HS12	T2	B08	1.362	1.311	1.311	0.105	22	2.320
T2HS13	T2	B09	1.168	1.117	1.117	0.073	22	1.616
T2HS13	T2	B10	1.384	1.333	1.333	0.109	22	2.399
0	T2	B11	0	0	kleiner LLOQ	#VALUE!	1	#VALUE!
0	T2	B12	0	0	kleiner LLOQ	#VALUE!	1	#VALUE!

Plate: T2

	1	2	3	4	5	6	7	8	9	10	11	12
A									1.078	1.053		
B							1.246	1.362	1.168	1.384		
C					0.958	0.995						
D												
E												
F												
G							0.056	0.055	0.055	0.055	0.056	0.063
H							0.052	0.053	0.053	0.053	0.052	0.052

Plattenbelegung

	1	2	3	4	5	6	7	8	9	10	11	12
A					T2HN1	T2HN1			T2HN3	T2HN3		
B							T2HS12	T2HS12	T2HS13	T2HS13		
C					T2HS21	T2HS21			T2HS23	T2HS23		
D												

0	T2	C01	0	0	kleiner LLOQ	#VALUE!	1	#VALUE!
0	T2	C02	0	0	kleiner LLOQ	#VALUE!	1	#VALUE!
0	T2	C03	0	0	kleiner LLOQ	#VALUE!	1	#VALUE!
0	T2	C04	0	0	kleiner LLOQ	#VALUE!	1	#VALUE!
T2HS21	T2	C05	0.958	0.907	0.907	0.039	22	0.852
T2HS21	T2	C06	0.995	0.944	0.944	0.045	22	0.984
0	T2	C07	0	0	kleiner LLOQ	#VALUE!	1	#VALUE!
0	T2	C08	0	0	kleiner LLOQ	#VALUE!	1	#VALUE!
T2HS23	T2	C09	0	0	kleiner LLOQ	#VALUE!	1	#VALUE!
T2HS23	T2	C10	0	0	kleiner LLOQ	#VALUE!	1	#VALUE!
0	T2	C11	0	0	kleiner LLOQ	#VALUE!	1	#VALUE!
0	T2	C12	0	0	kleiner LLOQ	#VALUE!	1	#VALUE!
0	T2	D01	0	0	kleiner LLOQ	#VALUE!	1	#VALUE!
0	T2	D02	0	0	kleiner LLOQ	#VALUE!	1	#VALUE!
0	T2	D03	0	0	kleiner LLOQ	#VALUE!	1	#VALUE!
0	T2	D04	0	0	kleiner LLOQ	#VALUE!	1	#VALUE!
0	T2	D05	0	0	kleiner LLOQ	#VALUE!	1	#VALUE!
0	T2	D06	0	0	kleiner LLOQ	#VALUE!	1	#VALUE!
0	T2	D07	0	0	kleiner LLOQ	#VALUE!	1	#VALUE!
0	T2	D08	0	0	kleiner LLOQ	#VALUE!	1	#VALUE!
0	T2	D09	0	0	kleiner LLOQ	#VALUE!	1	#VALUE!
0	T2	D10	0	0	kleiner LLOQ	#VALUE!	1	#VALUE!
0	T2	D11	0	0	kleiner LLOQ	#VALUE!	1	#VALUE!
0	T2	D12	0	0	kleiner LLOQ	#VALUE!	1	#VALUE!
0	T2	E01	0	0	kleiner LLOQ	#VALUE!	1	#VALUE!
0	T2	E02	0	0	kleiner LLOQ	#VALUE!	1	#VALUE!
0	T2	E03	0	0	kleiner LLOQ	#VALUE!	1	#VALUE!
0	T2	E04	0	0	kleiner LLOQ	#VALUE!	1	#VALUE!
0	T2	E05	0	0	kleiner LLOQ	#VALUE!	1	#VALUE!
0	T2	E06	0	0	kleiner LLOQ	#VALUE!	1	#VALUE!
0	T2	E07	0	0	kleiner LLOQ	#VALUE!	1	#VALUE!
0	T2	E08	0	0	kleiner LLOQ	#VALUE!	1	#VALUE!
0	T2	E09	0	0	kleiner LLOQ	#VALUE!	1	#VALUE!
0	T2	E10	0	0	kleiner LLOQ	#VALUE!	1	#VALUE!
0	T2	E11	0	0	kleiner LLOQ	#VALUE!	1	#VALUE!
0	T2	E12	0	0	kleiner LLOQ	#VALUE!	1	#VALUE!
0	T2	F01	0	0	kleiner LLOQ	#VALUE!	1	#VALUE!
0	T2	F02	0	0	kleiner LLOQ	#VALUE!	1	#VALUE!
0	T2	F03	0	0	kleiner LLOQ	#VALUE!	1	#VALUE!
0	T2	F04	0	0	kleiner LLOQ	#VALUE!	1	#VALUE!
0	T2	F05	0	0	kleiner LLOQ	#VALUE!	1	#VALUE!
0	T2	F06	0	0	kleiner LLOQ	#VALUE!	1	#VALUE!
0	T2	F07	0	0	kleiner LLOQ	#VALUE!	1	#VALUE!
0	T2	F08	0	0	kleiner LLOQ	#VALUE!	1	#VALUE!
0	T2	F09	0	0	kleiner LLOQ	#VALUE!	1	#VALUE!
0	T2	F10	0	0	kleiner LLOQ	#VALUE!	1	#VALUE!
0	T2	F11	0	0	kleiner LLOQ	#VALUE!	1	#VALUE!
0	T2	F12	0	0	kleiner LLOQ	#VALUE!	1	#VALUE!
0	T2	G01	0	0	kleiner LLOQ	#VALUE!	1	#VALUE!
0	T2	G02	0	0	kleiner LLOQ	#VALUE!	1	#VALUE!
0	T2	G03	0	0	kleiner LLOQ	#VALUE!	1	#VALUE!
0	T2	G04	0	0	kleiner LLOQ	#VALUE!	1	#VALUE!
0	T2	G05	0	0	kleiner LLOQ	#VALUE!	1	#VALUE!
0	T2	G06	0	0	kleiner LLOQ	#VALUE!	1	#VALUE!
Blank PEG	T2	G07	0	0	kleiner LLOQ	#VALUE!	2.75	#VALUE!
Blank PEG	T2	G08	0	0	kleiner LLOQ	#VALUE!	2.75	#VALUE!
Blank PEG	T2	G09	0	0	kleiner LLOQ	#VALUE!	2.75	#VALUE!
Blank PEG	T2	G10	0	0	kleiner LLOQ	#VALUE!	2.75	#VALUE!
Blank PEG	T2	G11	0	0	kleiner LLOQ	#VALUE!	2.75	#VALUE!
Blank PEG	T2	G12	0	0	kleiner LLOQ	#VALUE!	2.75	#VALUE!
0	T2	H01	0	0	kleiner LLOQ	#VALUE!	1	#VALUE!
0	T2	H02	0	0	kleiner LLOQ	#VALUE!	1	#VALUE!
0	T2	H03	0	0	kleiner LLOQ	#VALUE!	1	#VALUE!
0	T2	H04	0	0	kleiner LLOQ	#VALUE!	1	#VALUE!
0	T2	H05	0	0	kleiner LLOQ	#VALUE!	1	#VALUE!
0	T2	H06	0	0	kleiner LLOQ	#VALUE!	1	#VALUE!
Blank no PEG	T2	H07	0	0	kleiner LLOQ	#VALUE!	2.75	#VALUE!
Blank no PEG	T2	H08	0	0	kleiner LLOQ	#VALUE!	2.75	#VALUE!
Blank no PEG	T2	H09	0	0	kleiner LLOQ	#VALUE!	2.75	#VALUE!
Blank no PEG	T2	H10	0	0	kleiner LLOQ	#VALUE!	2.75	#VALUE!
Blank no PEG	T2	H11	0	0	kleiner LLOQ	#VALUE!	2.75	#VALUE!
Blank no PEG	T2	H12	0	0	kleiner LLOQ	#VALUE!	2.75	#VALUE!

E												
F												
G							Blank PEG	Blank PEG	Blank PEG	Blank PEG	Blank PEG	Blank PEG
H							Blank no PEG	Blank no PEG	Blank no PEG	Blank no PEG	Blank no PEG	Blank no PEG

dil8*2,75=dil.22

Comment:

Date Operator

Date Control

0	T3	A01	0	0	kleiner LLOQ	#VALUE!	1	#VALUE!
0	T3	A02	0	0	kleiner LLOQ	#VALUE!	1	#VALUE!
0	T3	A03	0	0	kleiner LLOQ	#VALUE!	1	#VALUE!
0	T3	A04	0	0	kleiner LLOQ	#VALUE!	1	#VALUE!
0	T3	A05	0	0	kleiner LLOQ	#VALUE!	1	#VALUE!
0	T3	A06	0	0	kleiner LLOQ	#VALUE!	1	#VALUE!
0	T3	A07	0	0	kleiner LLOQ	#VALUE!	1	#VALUE!
0	T3	A08	0	0	kleiner LLOQ	#VALUE!	1	#VALUE!
0	T3	A09	0	0	kleiner LLOQ	#VALUE!	1	#VALUE!
0	T3	A10	0	0	kleiner LLOQ	#VALUE!	1	#VALUE!
0	T3	A11	0	0	kleiner LLOQ	#VALUE!	1	#VALUE!
0	T3	A12	0	0	kleiner LLOQ	#VALUE!	1	#VALUE!
0	T3	B01	0	0	kleiner LLOQ	#VALUE!	1	#VALUE!
0	T3	B02	0	0	kleiner LLOQ	#VALUE!	1	#VALUE!
0	T3	B03	0	0	kleiner LLOQ	#VALUE!	1	#VALUE!
0	T3	B04	0	0	kleiner LLOQ	#VALUE!	1	#VALUE!
T3HS11	T3	B05	1.359	1.308	1.308	0.105	24.75	2.599
T3HS11	T3	B06	1.418	1.367	1.367	0.115	24.75	2.839
T3HS12	T3	B07	1.251	1.200	1.200	0.087	24.75	2.156
T3HS12	T3	B08	1.253	1.202	1.202	0.087	24.75	2.165
T3HS13	T3	B09	1.184	1.133	1.133	0.076	24.75	1.881
T3HS13	T3	B10	1.089	1.038	1.038	0.060	24.75	1.491
0	T3	B11	0	0	kleiner LLOQ	#VALUE!	1	#VALUE!
0	T3	B12	0	0	kleiner LLOQ	#VALUE!	1	#VALUE!
0	T3	C01	0	0	kleiner LLOQ	#VALUE!	1	#VALUE!
0	T3	C02	0	0	kleiner LLOQ	#VALUE!	1	#VALUE!
0	T3	C03	0	0	kleiner LLOQ	#VALUE!	1	#VALUE!
0	T3	C04	0	0	kleiner LLOQ	#VALUE!	1	#VALUE!
0	T3	C05	0	0	kleiner LLOQ	#VALUE!	1	#VALUE!
0	T3	C06	0	0	kleiner LLOQ	#VALUE!	1	#VALUE!
0	T3	C07	0	0	kleiner LLOQ	#VALUE!	1	#VALUE!
0	T3	C08	0	0	kleiner LLOQ	#VALUE!	1	#VALUE!
0	T3	C09	0	0	kleiner LLOQ	#VALUE!	1	#VALUE!
0	T3	C10	0	0	kleiner LLOQ	#VALUE!	1	#VALUE!
0	T3	C11	0	0	kleiner LLOQ	#VALUE!	1	#VALUE!
0	T3	C12	0	0	kleiner LLOQ	#VALUE!	1	#VALUE!
0	T3	D01	0	0	kleiner LLOQ	#VALUE!	1	#VALUE!
0	T3	D02	0	0	kleiner LLOQ	#VALUE!	1	#VALUE!
0	T3	D03	0	0	kleiner LLOQ	#VALUE!	1	#VALUE!
0	T3	D04	0	0	kleiner LLOQ	#VALUE!	1	#VALUE!
0	T3	D05	0	0	kleiner LLOQ	#VALUE!	1	#VALUE!
0	T3	D06	0	0	kleiner LLOQ	#VALUE!	1	#VALUE!
0	T3	D07	0	0	kleiner LLOQ	#VALUE!	1	#VALUE!
0	T3	D08	0	0	kleiner LLOQ	#VALUE!	1	#VALUE!
0	T3	D09	0	0	kleiner LLOQ	#VALUE!	1	#VALUE!
0	T3	D10	0	0	kleiner LLOQ	#VALUE!	1	#VALUE!
0	T3	D11	0	0	kleiner LLOQ	#VALUE!	1	#VALUE!
0	T3	D12	0	0	kleiner LLOQ	#VALUE!	1	#VALUE!
0	T3	E01	0	0	kleiner LLOQ	#VALUE!	1	#VALUE!
0	T3	E02	0	0	kleiner LLOQ	#VALUE!	1	#VALUE!
0	T3	E03	0	0	kleiner LLOQ	#VALUE!	1	#VALUE!
0	T3	E04	0	0	kleiner LLOQ	#VALUE!	1	#VALUE!
0	T3	E05	0	0	kleiner LLOQ	#VALUE!	1	#VALUE!
0	T3	E06	0	0	kleiner LLOQ	#VALUE!	1	#VALUE!
0	T3	E07	0	0	kleiner LLOQ	#VALUE!	1	#VALUE!
0	T3	E08	0	0	kleiner LLOQ	#VALUE!	1	#VALUE!
0	T3	E09	0	0	kleiner LLOQ	#VALUE!	1	#VALUE!

Plate: T3

	1	2	3	4	5	6	7	8	9	10	11	12
A												
B					1.359	1.418	1.251	1.253	1.184	1.089		
C												
D												
E												
F												
G							0.052	0.048	0.052	0.052	0.047	0.046
H							0.051	0.060	0.059	0.054	0.055	0.055

Plattenbelegung

	1	2	3	4	5	6	7	8	9	10	11	12
A												
B					T3HS11	T3HS11	T3HS12	T3HS12	T3HS13	T3HS13		
C												
D												
E												
F												
G							Blank PEG	Blank PEG	Blank PEG	Blank PEG	Blank PEG	Blank PEG
H							Blank no PEG	Blank no PEG	Blank no PEG	Blank no PEG	Blank no PEG	Blank no PEG

dil9*2,75=dil24,75

Comment:

Date Operator

Date Control

0	T3	E10	0	0	kleiner LLOQ	#VALUE!	1	#VALUE!
0	T3	E11	0	0	kleiner LLOQ	#VALUE!	1	#VALUE!
0	T3	E12	0	0	kleiner LLOQ	#VALUE!	1	#VALUE!
0	T3	F01	0	0	kleiner LLOQ	#VALUE!	1	#VALUE!
0	T3	F02	0	0	kleiner LLOQ	#VALUE!	1	#VALUE!
0	T3	F03	0	0	kleiner LLOQ	#VALUE!	1	#VALUE!
0	T3	F04	0	0	kleiner LLOQ	#VALUE!	1	#VALUE!
0	T3	F05	0	0	kleiner LLOQ	#VALUE!	1	#VALUE!
0	T3	F06	0	0	kleiner LLOQ	#VALUE!	1	#VALUE!
0	T3	F07	0	0	kleiner LLOQ	#VALUE!	1	#VALUE!
0	T3	F08	0	0	kleiner LLOQ	#VALUE!	1	#VALUE!
0	T3	F09	0	0	kleiner LLOQ	#VALUE!	1	#VALUE!
0	T3	F10	0	0	kleiner LLOQ	#VALUE!	1	#VALUE!
0	T3	F11	0	0	kleiner LLOQ	#VALUE!	1	#VALUE!
0	T3	F12	0	0	kleiner LLOQ	#VALUE!	1	#VALUE!
0	T3	G01	0	0	kleiner LLOQ	#VALUE!	1	#VALUE!
0	T3	G02	0	0	kleiner LLOQ	#VALUE!	1	#VALUE!
0	T3	G03	0	0	kleiner LLOQ	#VALUE!	1	#VALUE!
0	T3	G04	0	0	kleiner LLOQ	#VALUE!	1	#VALUE!
0	T3	G05	0	0	kleiner LLOQ	#VALUE!	1	#VALUE!
0	T3	G06	0	0	kleiner LLOQ	#VALUE!	1	#VALUE!
Blank PEG	T3	G07	0	0	kleiner LLOQ	#VALUE!	2.75	#VALUE!
Blank PEG	T3	G08	0	0	kleiner LLOQ	#VALUE!	2.75	#VALUE!
Blank PEG	T3	G09	0	0	kleiner LLOQ	#VALUE!	2.75	#VALUE!
Blank PEG	T3	G10	0	0	kleiner LLOQ	#VALUE!	2.75	#VALUE!
Blank PEG	T3	G11	0	0	kleiner LLOQ	#VALUE!	2.75	#VALUE!
Blank PEG	T3	G12	0	0	kleiner LLOQ	#VALUE!	2.75	#VALUE!
0	T3	H01	0	0	kleiner LLOQ	#VALUE!	1	#VALUE!
0	T3	H02	0	0	kleiner LLOQ	#VALUE!	1	#VALUE!
0	T3	H03	0	0	kleiner LLOQ	#VALUE!	1	#VALUE!
0	T3	H04	0	0	kleiner LLOQ	#VALUE!	1	#VALUE!
0	T3	H05	0	0	kleiner LLOQ	#VALUE!	1	#VALUE!
0	T3	H06	0	0	kleiner LLOQ	#VALUE!	1	#VALUE!
Blank no PEG	T3	H07	0	0	kleiner LLOQ	#VALUE!	2.75	#VALUE!
Blank no PEG	T3	H08	0	0	kleiner LLOQ	#VALUE!	2.75	#VALUE!
Blank no PEG	T3	H09	0	0	kleiner LLOQ	#VALUE!	2.75	#VALUE!
Blank no PEG	T3	H10	0	0	kleiner LLOQ	#VALUE!	2.75	#VALUE!
Blank no PEG	T3	H11	0	0	kleiner LLOQ	#VALUE!	2.75	#VALUE!
Blank no PEG	T3	H12	0	0	kleiner LLOQ	#VALUE!	2.75	#VALUE!

1.447	1.739		1.170						0.667	0.440	0.043	1
1.447	1.332		1.392	0.137	0.146	0.130	0.138	0.109	0.729	0.478	0.042	
1.391	1.281		1.311	0.204	0.184	0.184	0.198	0.201	0.743	0.474	0.043	
0.961	0.835		1.405	0.336	0.347	0.375	0.387	0.300	0.824	0.483	0.044	
0.445	0.441		1.280	1.085	1.086	1.218	1.004	0.873	0.704	0.457	0.041	
0.355	0.315		1.332	1.768	1.925	1.753	1.739	1.606			0.036	
0.223	0.188	1.666	1.499	1.632		0.048	0.046	0.047	0.048	0.048	0.048	
0.118	0.106	1.556	1.622	1.573		0.047	0.054	0.051	0.051	0.052	0.051	

1.652	1.627		1.188	0.748	0.811	0.764	0.753	0.674	0.751	0.529	0.044	2
1.443	1.372		1.364	0.139	0.147	0.131	0.139	0.110	0.811	0.561	0.043	
1.377	1.281		1.284	0.206	0.189	0.184	0.200	0.202	0.821	0.560	0.044	
0.972	0.883		1.387	0.339	0.349	0.386	0.406	0.312	0.903	0.565	0.044	
0.484	0.474		1.298	1.087	1.113	1.203	1.022	0.903	0.790	0.544	0.042	
0.359	0.326		1.308	1.622	1.622	1.630	1.625	1.550			0.037	
0.228	0.199	1.527	1.373	1.462		0.048	0.046	0.048	0.049	0.049	0.049	
0.125	0.109	1.473	1.410	1.371		0.048	0.054	0.052	0.052	0.052	0.051	

1.579	1.475		1.150	1.210	1.303	1.202	1.219	1.125	0.759	0.549	0.043	3
1.357	1.303		1.283	0.140	0.148	0.131	0.139	0.111	0.820	0.575	0.043	
1.323	1.228		1.227	0.206	0.188	0.184	0.198	0.201	0.824	0.579	0.043	
0.959	0.872		1.321	0.339	0.348	0.387	0.406	0.314	0.909	0.580	0.044	
0.483	0.473		1.270	1.069	1.103	1.181	1.017	0.903	0.798	0.560	0.042	
0.360	0.326		1.270	1.497	1.448	1.496	1.494	1.449			0.037	
0.228	0.199	1.374	1.262	1.314		0.048	0.046	0.048	0.049	0.049	0.049	
0.125	0.110	1.346	1.262	1.253		0.048	0.054	0.052	0.052	0.052	0.051	

1.511	1.369			1.538	1.712	1.528	1.502	1.425			0.043	4
1.296	1.261										0.043	
1.272	1.193										0.043	
0.947	0.859										0.044	
0.481	0.471										0.042	
0.361	0.325										0.037	
0.229	0.199					0.048	0.046	0.048	0.049	0.049	0.049	
0.125	0.110					0.047	0.054	0.052	0.052	0.052	0.051	

1.493	1.317	1.678	1.110	1.621	1.807	1.618	1.584	1.508	0.765	0.597	0.043	5
1.259	1.238	1.850	1.197	0.139	0.150	0.132	0.139	0.111	0.821	0.588	0.043	
1.248	1.175	1.816	1.153	0.204	0.188	0.184	0.197	0.199	0.831	0.599	0.044	
0.943	0.859	1.970	1.231	0.338	0.357	0.386	0.406	0.316	0.916	0.595	0.044	
0.480	0.470	2.220	1.220	1.054	1.088	1.154	1.006	0.902	0.805	0.579	0.042	
0.359	0.325	2.149	1.211	1.395	1.324	1.348	1.345	1.317	0.037	0.037	0.037	
0.228	0.199	1.240	1.151	1.183	0.034	0.049	0.046	0.048	0.049	0.049	0.049	
0.125	0.110	1.223	1.132	1.146	0.038	0.048	0.054	0.052	0.052	0.052	0.051	

1.434	1.258		1.090	1.648	1.876	1.649	1.627	1.535				6
1.217	1.201		1.149									
1.205	1.143		1.109									
0.935	0.845		1.182									
0.477	0.469		1.181									
0.360	0.323		1.167									
0.229	0.200					0.049	0.047	0.048	0.049	0.049	0.049	
0.125	0.110					0.048	0.054	0.052	0.052	0.052	0.051	

1.013	0.810		0.668	0.808	0.940	0.720	0.798	0.687	0.904	0.845	0.043	7
0.709	0.827		0.703	0.142	0.156	0.136	0.137	0.116	0.990	0.846	0.042	
0.682	0.692		0.680	0.197	0.188	0.190	0.191	0.184	1.023	0.861	0.043	
0.507	0.519		0.740	0.316	0.264	0.364	0.377	0.312	1.111	0.870	0.043	
0.394	0.406		0.682	0.580	0.561	0.600	0.529	0.563	0.962	0.834	0.042	
0.333	0.322		0.661	0.995	0.930	0.848	0.832	0.797	0.037	0.037	0.037	
0.229	0.201	0.843	0.679	0.732		0.048	0.046	0.047			0.047	
0.124	0.111	0.773	0.682	0.701		0.047	0.053	0.051	0.051	0.052	0.051	

Absorbance @ 450 (1.0s) (A)
0.000

Skin cultivation

T0 1 hum sui

1.459	1.152	1.423	0.357								
1.296	2.288	1.578	0.313								
2.179	1.936	1.737	0.247								
1.984	2.795	2.377	2.503								
2.245	1.949	2.433	1.796								
1.826	1.746	2.254	1.121								
						0.061	0.065	0.056	0.055	0.055	0.057
						0.051	0.05	0.051	0.05	0.05	0.049

T0 2 hum sui

				1.525	2.025		0.864	1.844	1.522	0.429	0.431
				1.248	2.278	1.692	1.755	0.855	0.894	0.237	0.249
				1.831	1.521	1.306	1.483	1.366	1.226	0.199	0.209
				2.68	2.613	2.821	2.765	2.739	2.522	1.226	1.007
				2.824	2.685	2.57	2.293	2.352	2.402	0.689	0.637
				2.742	2.589	2.376	1.965	2.348	2.564	1.208	1.157
						0.056	0.057	0.057	0.055	0.056	0.054
						0.051	0.051	0.051	0.054	0.05	0.049

T0 3 hum sui

1.146	0.718	0.999	0.334	1.126	1.411		0.702	1.266	1.141	0.475	0.393
1.079	1.702	1.052	0.332	0.879	1.610	1.471	1.476	0.811	0.727	0.349	0.252
1.569	1.355	1.089	0.334	1.638	1.359	1.118	1.137	1.055	0.994	0.283	0.211
1.482	2.065	1.7	2.17	2.322	2.252	2.398	2.423	2.316	2.134	0.951	0.742
1.431	1.376	1.754	1.439	2.494	2.177	2.199	2.054	2.029	2.004	0.618	0.508
1.277	1.075	1.556	0.841	2.342	2.042	1.836	1.62	1.988	2.138	0.912	0.852
						0.056	0.233	0.207	0.213	0.063	0.053
						0.052	0.05	0.112	0.06	0.05	0.048

T0 4 hum sui

1.198	1.291	1.335	1.239	1.265	1.305						
1.302	1.349	1.214	1.161	1.334	1.291						
1.262	1.442	1.168	1.090	1.247	1.062						
1.150	1.191	1.027									
0.065	0.066	0.062	0.072	0.060	0.070						
0.044	0.045	0.048	0.046	0.047	0.044						

T1 1 hum sui

1.180	1.681	1.830	0.163								
1.567	1.334	1.200	0.100								
1.195	0.788	0.933	0.102								
2.900	2.806	0.758	2.806								
1.946	2.631	2.402	2.890								
2.868	2.795	2.317	3.094								
						0.063	0.052	0.051	0.055	0.051	0.054
						0.050	0.050	0.050	0.051	0.051	0.052

T1 2 hum sui

				1.200	1.072	0.423	0.615	0.670	0.724	0.150	0.153
				1.198	1.883	1.575	1.474	1.325	1.299	0.147	0.138
				1.216	1.428	1.153	1.237	1.217	1.351	0.092	0.112
				1.874	1.939	1.016	1.042	2.511	2.747	0.342	0.313
				0.964	1.027	2.081	2.098	2.017	1.779	0.439	0.439
				0.965	1.043	1.423	1.549	0.497	0.475	0.692	0.690
0.285	0.298					0.065	0.053	0.052	0.056	0.050	0.055
						0.051	0.051	0.051	0.052	0.052	0.051

T1 3 hum sui

		0.998	0.911	0.555	0.519	0.064	0.054	0.052	0.056	0.051	0.055
0.381	0.357	0.306	0.303			0.051	0.051	0.051	0.052	0.052	0.052

1.835	2.383	2.469	0.131									T2 1 hum sui
2.283	2.561	2.352	0.149									
1.978	1.929	2.009	0.110									
	2.292	1.277	1.608									
1.300	2.309	1.954	1.414									
2.308	1.373	1.384	1.407									
						0.054	0.053	0.053	0.053	0.053	0.052	
						0.050	0.051	0.051	0.051	0.051	0.051	

				0.933	0.908	0.853	0.834	1.078	1.053	0.140	0.136	T2 2 hum sui
				1.706	1.731	1.246	1.362	1.168	1.384	0.141	0.139	
				0.958	0.995	0.855	0.843	0.911	0.882	0.117	0.113	
						0.727		1.460	1.257	1.233	1.375	
				2.072	2.091	0.574	0.573	0.572	0.589	1.554	1.513	
				0.606	0.608	2.006	1.811	2.076	1.983	1.751	1.706	
						0.056	0.055	0.055	0.055	0.056	0.063	
						0.052	0.053	0.053	0.053	0.052	0.052	

												T2 3 hum sui
0.490	0.485	0.511	0.494	0.744	0.774	0.056	0.055	0.055	0.057	0.056	0.068	
0.503	0.559	0.312	0.313	2.569	2.528	0.053	0.053	0.053	0.053	0.052	0.052	

0.486	0.355	0.412	0.161									T3 1 hum sui
2.733	2.788	2.574	0.143									
2.165	2.221	2.003	0.121									
	2.184	1.908	1.678									
1.487	2.296	1.702	1.452									
1.489	1.603	1.140	1.130			0.052	0.051	0.052	0.052	0.046	0.048	
						0.050	0.059	0.056	0.054	0.055	0.054	

				0.460	0.524	0.298	0.342	0.392	0.394	0.138	0.152	T3 2 hum sui
				1.359	1.418	1.251	1.253	1.184	1.089	0.144	0.148	
				0.766	0.765	0.780	0.796	0.634	0.647	0.110	0.121	
						0.410	0.080	0.262	0.231	0.503	0.547	
				0.557	0.530	0.621	0.561	0.682	0.634	0.414	0.402	
				0.475	0.468	0.368	0.400	1.557	1.542	0.945	1.229	
						0.052	0.048	0.052	0.052	0.047	0.046	
						0.051	0.060	0.059	0.054	0.055	0.055	

**Project number**		F-120	**Apparatus**	**Wallac Victor**	**Operator**	**IsBa**
**GLP Study (Number)**		n.a.	**Protocol (Instrument method)**	**LDH test 2016**	**Date of preparation**	**19-04-18**
**hot substance**	**isotope**	n.a.	**File name (results)**	**IsBa_180419/20_LDH_full_va**	**Date of measurement**	**19-04-18**
	**name**	n.a.	**Kind of well plate**	**96 well**	**shaking time [min]**	**30**
	**ACB-ID**	n.a.	**sample volume [µL]**	**100**	**stirring rate (Target) [rpm]**	**150**
	**Batch number**	n.a.	**Cocktail volume [µL]**	**175**	**Kind of measurement**	**UV-vis**
**cold substance**	**name**	LDH	**ACB-ID of cocktail**	**n.a.**	**Wave length [nm]**	**450**
	**ACB-ID**	L23982R	**Matrix**	**DMEM (from powder)+PEG**	**Remarks**	**Cocktail 100µl RM, 75µl STOP**
	**Batch number**	SLBK6345V	**Blank description**	**DMEM/PEG, H2O**	**Remarks**	**7 standards split low/high**
**n.a.**		n.a.	**Pipettes (No. / volume)**	**50-200µl**	**Remarks**	**KLP4 common for both**
**n.a.**		n.a.	**Pipettes (No. / volume)**	**n.a.**	**Remarks**	**n.a.**

Messdaten (diese Tabelle in Bericht übernehmen)

Sample name *	concentration (theor.) *	measured data	measured data	measured data	mean measured	SD	RSD	Blank *	measured data after * Blank subtraction	concentration (calc.) *	Deviation *	Residuen
	[µg/mL]	[AU]	[AU]	[AU]	[AU]	[AU]	[%]	[AU]		[µg/mL]	[%]	
KLP1								0.047				
KLP2								0.054				
KLP3								0.051				
KLP4	0.041	0.959	0.972	0.883	0.938	0.04	4.19	0.051	0.887	0.041	-0.57	0.00
KLP5	0.018	0.483	0.484	0.474	0.480	0.00	0.98	0.052	0.429	0.019	2.42	0.00
KLP6	0.012	0.360	0.359	0.326	0.348	0.02	4.54	0.051	0.297	0.013	5.00	0.00
KLP7	0.007	0.228	0.228	0.199	0.218	0.014	6.35		0.168	0.006	-11.29	0.00
KLP8												

Statistical data

Geradensteigung	Slope	m	21.03
Y-Achsenabschnitt	Y-Intercept	b	0.03
Standardabw. Geradensteigung	SD-Slope	s_m	0.648055501
Standardabw. Achsenabschnittes	SD-Y-Intercept	s_b	0.015177452
Anzahl Messpunkte	number of measuring points	n	4
Quadratsumme	sum of squares	Qxx	0.000664048
Bereichsmitte			0.019557193
Freiheitsgrade	degree of freedom	f	2
Student-t-Faktor für (P = 95 %; f = n-2)	Student-t-factor for (P = 95 %; f = n-2)	t	4.303
Vertrauensbereich Steig. (95 %) Obergrenze		$m + VB_m$	23.81763508
Vertrauensbereich Steig. (95 %) Untergrenze		$m - VB_m$	18.24046944
Vertrauensbereich Achsenabschnitt (95 %) Og.		$b + Vb_b$	0.099335829
Vertrauensbereich Achsenabschnitt (95 %) Ug.		$b - Vb_b$	-0.031281327
Korrelationskoeffizient	correlation coefficient	r	0.9991
Bestimmtheitsmaß	determiantion coefficient	r^2	0.9981
Reststandardabweichung		s_0	0.01669983
Summe Restquadrate		sd	0.657385838
Verfahrensstandardabw.		s_{x0}	0.000794131
Rel. Verfahrensstandardabw. %		V_{x0}	4.060559029

Berichten

mean Blank	0	
SD Blank	0.00	
RSD Blank	3.79	x=
x*SD (LLOQ)	0.01	5
x*SD (LOD)	0.01	3
**LLOQ (AU)**	**0.061**	
**LOD (AU)**	**0.057**	
ULOQ	0.887	
LLOQ (Lin)	0.168	

Evaluation / Comment

LDH linearity valid with 7 standards, split in lin high and lin low, each with 4 standards and KLP4 common standard for both. R² 0,9981, deviations for both between -13.34% and +13.04%

Date Operator Date Control

Figure 1 Linearity

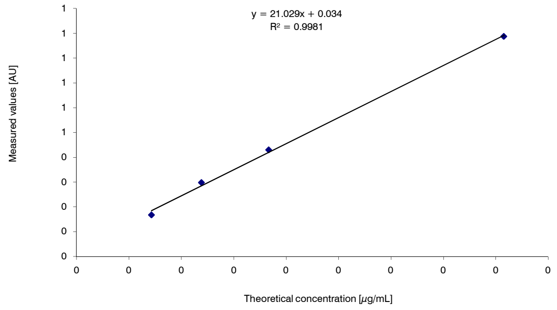

Figure 2 Method validation Residuen Plot

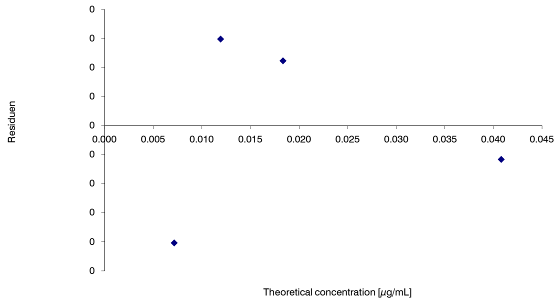

Evaluation / Comment

lin passed

Date	Operator	Date	Control

Project number		F-120	Apparatus	Wallac Victor	Operator	IsBa	LLOQ [AU]	0.168
GLP Study (Number)		n.a.	Protocol (Instrument method)	LDH test 2016	Date of preparation	43209	ULOQ [AU]	0.887
hot substance	Isotope	n.a.	File name (results)	IsBa_180419/20_LDH_full_val_1/2	Date of measurement	43209	Blank	0.051
	Name	n.a.	Kind of well plate	96 well	Shaking time [min]	30		n.a.
	ACB-ID	n.a.	sample volume [µL]	100	Stirring rate (Target) [rpm]	150		n.a.
	Batch number	n.a.	Cocktail volume [µL]	175	Kind of measurement	UV-vis	Pipettes (No. / volume)	n.a.
cold substance	Name	LDH	ACB-ID of cocktail	n.a.	Wave length [nm]	450	Pipettes (No. / volume)	n.a.
	ACB-ID	L23982R	Matrix	DMEM (from powder)+PEG	Remarks	Sui mitgemessen auf der Platte aber Aus	Pipettes (No. / volume)	n.a.
	Batch number	SLBK6345V	Blank description	DMEM/PEG, H2O	Remarks	n.a.	Remarks	n.a.

Sample name	Plate	Position	[AU]	[AU]-Blank	Rating	[µg/mL]	Dilution factor	[µg/mL]
0	T4	A01	0	0	kleiner LLOQ	#VALUE!	1	#VALUE!
0	T4	A02	0	0	kleiner LLOQ	#VALUE!	1	#VALUE!
0	T4	A03	0	0	kleiner LLOQ	#VALUE!	1	#VALUE!
0	T4	A04	0	0	kleiner LLOQ	#VALUE!	1	#VALUE!
T4HN1	T4	A05	0	0	kleiner LLOQ	#VALUE!	1	#VALUE!
T4HN1	T4	A06	0	0	kleiner LLOQ	#VALUE!	1	#VALUE!
T4HN2	T4	A07	0	0	kleiner LLOQ	#VALUE!	1	#VALUE!
T4HN2	T4	A08	0.000	-0.051	kleiner LLOQ	#VALUE!	1	#VALUE!
T4HN3	T4	A09	0.915	0.864	0.864	0.039	24.75	0.977
T4HN3	T4	A10	0.921	0.870	0.870	0.040	24.75	0.984
T4HN4	T4	A11	0.436	0.385	0.385	0.017	2.75	0.046
T4HN4	T4	A12	0.431	0.380	0.380	0.016	2.75	0.045
0	T4	B01	0	0	kleiner LLOQ	#VALUE!	1	#VALUE!
0	T4	B02	0	0	kleiner LLOQ	#VALUE!	1	#VALUE!
0	T4	B03	0	0	kleiner LLOQ	#VALUE!	1	#VALUE!
0	T4	B04	0	0	kleiner LLOQ	#VALUE!	1	#VALUE!
T4HS11	T4	B05	0.000	-0.051	kleiner LLOQ	#VALUE!	1	#VALUE!
T4HS11	T4	B06	0.846	0.795	0.795	0.036	24.75	0.896
T4HS12	T4	B07	0.590	0.539	0.539	0.024	24.75	0.595
T4HS12	T4	B08	0.597	0.546	0.546	0.024	24.75	0.603
T4HS13	T4	B09	0	0	kleiner LLOQ	#VALUE!	1	#VALUE!
T4HS13	T4	B10	0	0	kleiner LLOQ	#VALUE!	1	#VALUE!
T4HS14	T4	B11	0.169	0.118	kleiner LLOQ	#VALUE!	2.75	#VALUE!
T4HS14	T4	B12	0.174	0.124	kleiner LLOQ	#VALUE!	2.75	#VALUE!
0	T4	C01	0	0	kleiner LLOQ	#VALUE!	1	#VALUE!
0	T4	C02	0	0	kleiner LLOQ	#VALUE!	1	#VALUE!
0	T4	C03	0	0	kleiner LLOQ	#VALUE!	1	#VALUE!
0	T4	C04	0	0	kleiner LLOQ	#VALUE!	1	#VALUE!
T4HS21	T4	C05	0.558	0.507	0.507	0.022	11	0.247
T4HS21	T4	C06	0.564	0.513	0.513	0.023	11	0.250
T4HS22	T4	C07	0.603	0.552	0.552	0.025	11	0.271
T4HS22	T4	C08	0.587	0.536	0.536	0.024	11	0.263
T4HS23	T4	C09	0.591	0.540	0.540	0.024	11	0.264
T4HS23	T4	C10	0.594	0.543	0.543	0.024	11	0.266
T4HS24	T4	C11	0.198	0.147	kleiner LLOQ	#VALUE!	2.75	#VALUE!
T4HS24	T4	C12	0.201	0.150	kleiner LLOQ	#VALUE!	2.75	#VALUE!
0	T4	D01	0	0	kleiner LLOQ	#VALUE!	1	#VALUE!
0	T4	D02	0	0	kleiner LLOQ	#VALUE!	1	#VALUE!
0	T4	D03	0	0	kleiner LLOQ	#VALUE!	1	#VALUE!
0	T4	D04	0	0	kleiner LLOQ	#VALUE!	1	#VALUE!
0	T4	D05	0	0	kleiner LLOQ	#VALUE!	1	#VALUE!
0	T4	D06	0	0	kleiner LLOQ	#VALUE!	1	#VALUE!
0	T4	D07	0	0	kleiner LLOQ	#VALUE!	1	#VALUE!
0	T4	D08	0	0	kleiner LLOQ	#VALUE!	1	#VALUE!
0	T4	D09	0	0	kleiner LLOQ	#VALUE!	1	#VALUE!
0	T4	D10	0	0	kleiner LLOQ	#VALUE!	1	#VALUE!
0	T4	D11	0	0	kleiner LLOQ	#VALUE!	1	#VALUE!
0	T4	D12	0	0	kleiner LLOQ	#VALUE!	1	#VALUE!

Plate: T4

	Test 1	Test 2	Test 3	Test 4								
	1	2	3	4	5	6	7	8	9	10	11	12
A									0.915	0.921	0.436	0.431
B						0.846	0.590	0.597			0.169	0.174
C					0.558	0.564	0.603	0.587	0.591	0.594	0.198	0.201
D												
E												
F												
G	0.633	0.717					0.052	0.054	0.052	0.052	0.052	0.051
H							0.050	0.050	0.053	0.050	0.051	0.050

Plattenbelegung

	1	2	3	4	5	6	7	8	9	10	11	12
A					T4HN1	T4HN1	T4HN2	T4HN2	T4HN3	T4HN3	T4HN4	T4HN4
B					T4HS11	T4HS11	T4HS12	T4HS12	T4HS13	T4HS13	T4HS14	T4HS14
C					T4HS21	T4HS21	T4HS22	T4HS22	T4HS23	T4HS23	T4HS24	T4HS24
D												
E												
F												
G	T4HS13	T4HS13					Blank PEG	Blank PEG	Blank PEG	Blank PEG	Blank PEG	Blank PEG
H							Blank no PEG	Blank no PEG	Blank no PEG	Blank no PEG	Blank no PEG	Blank no PEG

Samples aufgeteilt in lin high und low:höher konzentrierte Samples bei lin high zu finden.

dil 9*dil2,75=dil.24.75

dil 4*dil2,75=dil.11

Comment:

Date Operator

Date Control

0	T4	E01	0	0	kleiner LLOQ	#VALUE!	1	#VALUE!
0	T4	E02	0	0	kleiner LLOQ	#VALUE!	1	#VALUE!
0	T4	E03	0	0	kleiner LLOQ	#VALUE!	1	#VALUE!
0	T4	E04	0	0	kleiner LLOQ	#VALUE!	1	#VALUE!
0	T4	E05	0	0	kleiner LLOQ	#VALUE!	1	#VALUE!
0	T4	E06	0	0	kleiner LLOQ	#VALUE!	1	#VALUE!
0	T4	E07	0	0	kleiner LLOQ	#VALUE!	1	#VALUE!
0	T4	E08	0	0	kleiner LLOQ	#VALUE!	1	#VALUE!
0	T4	E09	0	0	kleiner LLOQ	#VALUE!	1	#VALUE!
0	T4	E10	0	0	kleiner LLOQ	#VALUE!	1	#VALUE!
0	T4	E11	0	0	kleiner LLOQ	#VALUE!	1	#VALUE!
0	T4	E12	0	0	kleiner LLOQ	#VALUE!	1	#VALUE!
0	T4	F01	0	0	kleiner LLOQ	#VALUE!	1	#VALUE!
0	T4	F02	0	0	kleiner LLOQ	#VALUE!	1	#VALUE!
0	T4	F03	0	0	kleiner LLOQ	#VALUE!	1	#VALUE!
0	T4	F04	0	0	kleiner LLOQ	#VALUE!	1	#VALUE!
0	T4	F05	0	0	kleiner LLOQ	#VALUE!	1	#VALUE!
0	T4	F06	0	0	kleiner LLOQ	#VALUE!	1	#VALUE!
0	T4	F07	0	0	kleiner LLOQ	#VALUE!	1	#VALUE!
0	T4	F08	0	0	kleiner LLOQ	#VALUE!	1	#VALUE!
0	T4	F09	0	0	kleiner LLOQ	#VALUE!	1	#VALUE!
0	T4	F10	0	0	kleiner LLOQ	#VALUE!	1	#VALUE!
0	T4	F11	0	0	kleiner LLOQ	#VALUE!	1	#VALUE!
0	T4	F12	0	0	kleiner LLOQ	#VALUE!	1	#VALUE!
T4HS13	T4	G01	0.633	0.582	0.582	0.026	24.75	0.645
T4HS13	T4	G02	0.717	0.666	0.666	0.030	24.75	0.744
0	T4	G03	0	0	kleiner LLOQ	#VALUE!	1	#VALUE!
0	T4	G04	0	0	kleiner LLOQ	#VALUE!	1	#VALUE!
0	T4	G05	0	0	kleiner LLOQ	#VALUE!	1	#VALUE!
0	T4	G06	0	0	kleiner LLOQ	#VALUE!	1	#VALUE!
Blank PEG	T4	G07	0	0	kleiner LLOQ	#VALUE!	2.75	#VALUE!
Blank PEG	T4	G08	0	0	kleiner LLOQ	#VALUE!	2.75	#VALUE!
Blank PEG	T4	G09	0	0	kleiner LLOQ	#VALUE!	2.75	#VALUE!
Blank PEG	T4	G10	0	0	kleiner LLOQ	#VALUE!	2.75	#VALUE!
Blank PEG	T4	G11	0	0	kleiner LLOQ	#VALUE!	2.75	#VALUE!
Blank PEG	T4	G12	0	0	kleiner LLOQ	#VALUE!	2.75	#VALUE!
0	T4	H01	0	0	kleiner LLOQ	#VALUE!	1	#VALUE!
0	T4	H02	0	0	kleiner LLOQ	#VALUE!	1	#VALUE!
0	T4	H03	0	0	kleiner LLOQ	#VALUE!	1	#VALUE!
0	T4	H04	0	0	kleiner LLOQ	#VALUE!	1	#VALUE!
0	T4	H05	0	0	kleiner LLOQ	#VALUE!	1	#VALUE!
0	T4	H06	0	0	kleiner LLOQ	#VALUE!	1	#VALUE!
Blank no PEG	T4	H07	0	0	kleiner LLOQ	#VALUE!	2.75	#VALUE!
Blank no PEG	T4	H08	0	0	kleiner LLOQ	#VALUE!	2.75	#VALUE!
Blank no PEG	T4	H09	0	0	kleiner LLOQ	#VALUE!	2.75	#VALUE!
Blank no PEG	T4	H10	0	0	kleiner LLOQ	#VALUE!	2.75	#VALUE!
Blank no PEG	T4	H11	0	0	kleiner LLOQ	#VALUE!	2.75	#VALUE!
Blank no PEG	T4	H12	0	0	kleiner LLOQ	#VALUE!	2.75	#VALUE!

0	T5	A01	0	0	kleiner LLOQ	#VALUE!	1	#VALUE!
0	T5	A02	0	0	kleiner LLOQ	#VALUE!	1	#VALUE!
0	T5	A03	0	0	kleiner LLOQ	#VALUE!	1	#VALUE!
0	T5	A04	0	0	kleiner LLOQ	#VALUE!	1	#VALUE!
T5HN1	T5	A05	0.749	0.699	0.699	0.032	27.5	0.869
T5HN1	T5	A06	0.763	0.712	0.712	0.032	27.5	0.886
T5HN2	T5	A07	0	0	kleiner LLOQ	#VALUE!	1	#VALUE!
T5HN2	T5	A08	0	0	kleiner LLOQ	#VALUE!	1	#VALUE!
T5HN3	T5	A09	0	0	kleiner LLOQ	#VALUE!	1	#VALUE!
T5HN3	T5	A10	0	0	kleiner LLOQ	#VALUE!	1	#VALUE!
T5HN4	T5	A11	0.735	0.684	0.684	0.031	2.75	0.085
T5HN4	T5	A12	0.717	0.666	0.666	0.030	2.75	0.083
0	T5	B01	0	0	kleiner LLOQ	#VALUE!	1	#VALUE!
0	T5	B02	0	0	kleiner LLOQ	#VALUE!	1	#VALUE!
0	T5	B03	0	0	kleiner LLOQ	#VALUE!	1	#VALUE!
0	T5	B04	0	0	kleiner LLOQ	#VALUE!	1	#VALUE!
T5HS11	T5	B05	0.323	0.272	0.272	0.011	2.75	0.031
T5HS11	T5	B06	0.315	0.265	0.265	0.011	2.75	0.030
T5HS12	T5	B07	0.263	0.212	0.212	0.008	2.75	0.023
T5HS12	T5	B08	0.273	0.222	0.222	0.009	2.75	0.025
T5HS13	T5	B09	0.403	0.352	0.352	0.015	2.75	0.042
T5HS13	T5	B10	0.405	0.354	0.354	0.015	2.75	0.042
T5HS14	T5	B11	0.192	0.141	kleiner LLOQ	#VALUE!	2.75	#VALUE!
T5HS14	T5	B12	0.198	0.147	kleiner LLOQ	#VALUE!	2.75	#VALUE!
0	T5	C01	0	0	kleiner LLOQ	#VALUE!	1	#VALUE!
0	T5	C02	0	0	kleiner LLOQ	#VALUE!	1	#VALUE!
0	T5	C03	0	0	kleiner LLOQ	#VALUE!	1	#VALUE!
0	T5	C04	0	0	kleiner LLOQ	#VALUE!	1	#VALUE!
T5HS21	T5	C05	0.636	0.585	0.585	0.026	8.25	0.216
T5HS21	T5	C06	0.661	0.610	0.610	0.027	8.25	0.226
T5HS22	T5	C07	0.715	0.664	0.664	0.030	8.25	0.247
T5HS22	T5	C08	0.706	0.655	0.655	0.030	8.25	0.244
T5HS23	T5	C09	0	0	kleiner LLOQ	#VALUE!	1	#VALUE!
T5HS23	T5	C10	0	0	kleiner LLOQ	#VALUE!	1	#VALUE!
T5HS24	T5	C11	0.232	0.181	0.181	0.007	2.75	0.019
T5HS24	T5	C12	0.230	0.179	0.179	0.007	2.75	0.019
0	T5	D01	0	0	kleiner LLOQ	#VALUE!	1	#VALUE!
0	T5	D02	0	0	kleiner LLOQ	#VALUE!	1	#VALUE!
0	T5	D03	0	0	kleiner LLOQ	#VALUE!	1	#VALUE!
0	T5	D04	0	0	kleiner LLOQ	#VALUE!	1	#VALUE!
0	T5	D05	0	0	kleiner LLOQ	#VALUE!	1	#VALUE!
0	T5	D06	0	0	kleiner LLOQ	#VALUE!	1	#VALUE!
0	T5	D07	0	0	kleiner LLOQ	#VALUE!	1	#VALUE!
0	T5	D08	0	0	kleiner LLOQ	#VALUE!	1	#VALUE!
0	T5	D09	0	0	kleiner LLOQ	#VALUE!	1	#VALUE!
0	T5	D10	0	0	kleiner LLOQ	#VALUE!	1	#VALUE!
0	T5	D11	0	0	kleiner LLOQ	#VALUE!	1	#VALUE!
0	T5	D12	0	0	kleiner LLOQ	#VALUE!	1	#VALUE!

Plate: T5

	1	2	3	4	5	6	7	8	9	10	11	12
A					0.749	0.763					0.735	0.717
B					0.323	0.315	0.263	0.273	0.403	0.405	0.192	0.198
C					0.636	0.661	0.715	0.706			0.232	0.230
D												
E												
F												
G							0.053	0.053	0.054	0.053	0.053	0.052
H							0.101	0.056	0.052	0.050	0.054	0.053

Plattenbelegung

	1	2	3	4	5	6	7	8	9	10	11	12
A					T5HN1	T5HN1	T5HN2	T5HN2	T5HN3	T5HN3	T5HN4	T5HN4
B					T5HS11	T5HS11	T5HS12	T5HS12	T5HS13	T5HS13	T5HS14	T5HS14
C					T5HS21	T5HS21	T5HS22	T5HS22	T5HS23	T5HS23	T5HS24	T5HS24
D												
E												
F												
G							Blank PEG	Blank PEG	Blank PEG	Blank PEG	Blank PEG	Blank PEG
H							Blank no PEG	Blank no PEG	Blank no PEG	Blank no PEG	Blank no PEG	Blank no PEG

dil10*dil2,75=dil.27,5
dil3*dil2,75=dil.8,25

Comment:

Date Operator

Date Control

0	T5	E01	0	0	kleiner LLOQ	#VALUE!	1	#VALUE!
0	T5	E02	0	0	kleiner LLOQ	#VALUE!	1	#VALUE!
0	T5	E03	0	0	kleiner LLOQ	#VALUE!	1	#VALUE!
0	T5	E04	0	0	kleiner LLOQ	#VALUE!	1	#VALUE!
0	T5	E05	0	0	kleiner LLOQ	#VALUE!	1	#VALUE!
0	T5	E06	0	0	kleiner LLOQ	#VALUE!	1	#VALUE!
0	T5	E07	0	0	kleiner LLOQ	#VALUE!	1	#VALUE!
0	T5	E08	0	0	kleiner LLOQ	#VALUE!	1	#VALUE!
0	T5	E09	0	0	kleiner LLOQ	#VALUE!	1	#VALUE!
0	T5	E10	0	0	kleiner LLOQ	#VALUE!	1	#VALUE!
0	T5	E11	0	0	kleiner LLOQ	#VALUE!	1	#VALUE!
0	T5	E12	0	0	kleiner LLOQ	#VALUE!	1	#VALUE!
0	T5	F01	0	0	kleiner LLOQ	#VALUE!	1	#VALUE!
0	T5	F02	0	0	kleiner LLOQ	#VALUE!	1	#VALUE!
0	T5	F03	0	0	kleiner LLOQ	#VALUE!	1	#VALUE!
0	T5	F04	0	0	kleiner LLOQ	#VALUE!	1	#VALUE!
0	T5	F05	0	0	kleiner LLOQ	#VALUE!	1	#VALUE!
0	T5	F06	0	0	kleiner LLOQ	#VALUE!	1	#VALUE!
0	T5	F07	0	0	kleiner LLOQ	#VALUE!	1	#VALUE!
0	T5	F08	0	0	kleiner LLOQ	#VALUE!	1	#VALUE!
0	T5	F09	0	0	kleiner LLOQ	#VALUE!	1	#VALUE!
0	T5	F10	0	0	kleiner LLOQ	#VALUE!	1	#VALUE!
0	T5	F11	0	0	kleiner LLOQ	#VALUE!	1	#VALUE!
0	T5	F12	0	0	kleiner LLOQ	#VALUE!	1	#VALUE!
0	T5	G01	0	0	kleiner LLOQ	#VALUE!	1	#VALUE!
0	T5	G02	0	0	kleiner LLOQ	#VALUE!	1	#VALUE!
0	T5	G03	0	0	kleiner LLOQ	#VALUE!	1	#VALUE!
0	T5	G04	0	0	kleiner LLOQ	#VALUE!	1	#VALUE!
0	T5	G05	0	0	kleiner LLOQ	#VALUE!	1	#VALUE!
0	T5	G06	0	0	kleiner LLOQ	#VALUE!	1	#VALUE!
Blank PEG	T5	G07	0.053	0.002	kleiner LLOQ	#VALUE!	2.75	#VALUE!
Blank PEG	T5	G08	0.053	0.002	kleiner LLOQ	#VALUE!	2.75	#VALUE!
Blank PEG	T5	G09	0.054	0.003	kleiner LLOQ	#VALUE!	2.75	#VALUE!
Blank PEG	T5	G10	0.053	0.002	kleiner LLOQ	#VALUE!	2.75	#VALUE!
Blank PEG	T5	G11	0.053	0.002	kleiner LLOQ	#VALUE!	2.75	#VALUE!
Blank PEG	T5	G12	0.052	0.001	kleiner LLOQ	#VALUE!	2.75	#VALUE!
0	T5	H01	0	0	kleiner LLOQ	#VALUE!	1	#VALUE!
0	T5	H02	0	0	kleiner LLOQ	#VALUE!	1	#VALUE!
0	T5	H03	0	0	kleiner LLOQ	#VALUE!	1	#VALUE!
0	T5	H04	0	0	kleiner LLOQ	#VALUE!	1	#VALUE!
0	T5	H05	0	0	kleiner LLOQ	#VALUE!	1	#VALUE!
0	T5	H06	0	0	kleiner LLOQ	#VALUE!	1	#VALUE!
Blank no PEG	T5	H07	0.101	0.050	kleiner LLOQ	#VALUE!	2.75	#VALUE!
Blank no PEG	T5	H08	0.056	0.005	kleiner LLOQ	#VALUE!	2.75	#VALUE!
Blank no PEG	T5	H09	0.052	0.001	kleiner LLOQ	#VALUE!	2.75	#VALUE!
Blank no PEG	T5	H10	0.050	-0.001	kleiner LLOQ	#VALUE!	2.75	#VALUE!
Blank no PEG	T5	H11	0.054	0.003	kleiner LLOQ	#VALUE!	2.75	#VALUE!
Blank no PEG	T5	H12	0.053	0.002	kleiner LLOQ	#VALUE!	2.75	#VALUE!
0	T6	A01	0	0	kleiner LLOQ	#VALUE!	1	#VALUE!
0	T6	A02	0	0	kleiner LLOQ	#VALUE!	1	#VALUE!
0	T6	A03	0	0	kleiner LLOQ	#VALUE!	1	#VALUE!
0	T6	A04	0	0	kleiner LLOQ	#VALUE!	1	#VALUE!
T6HN1	T6	A05	0.873	0.822	0.822	0.037	22	0.824
T6HN1	T6	A06	0.588	0.537	0.537	0.024	22	0.526
T6HN2	T6	A07	0.744	0.693	0.693	0.031	22	0.690
T6HN2	T6	A08	0.778	0.727	0.727	0.033	22	0.725
T6HN3	T6	A09	0.518	0.467	0.467	0.021	22	0.453
T6HN3	T6	A10	0.513	0.462	0.462	0.020	22	0.448
T6HN4	T6	A11	0.634	0.583	0.583	0.026	2.75	0.072
T6HN4	T6	A12	0.620	0.569	0.569	0.025	2.75	0.070
0	T6	B01	0	0	kleiner LLOQ	#VALUE!	1	#VALUE!
0	T6	B02	0	0	kleiner LLOQ	#VALUE!	1	#VALUE!
0	T6	B03	0	0	kleiner LLOQ	#VALUE!	1	#VALUE!
0	T6	B04	0	0	kleiner LLOQ	#VALUE!	1	#VALUE!
T6HS11	T6	B05	0.274	0.223	0.223	0.009	2.75	0.025
T6HS11	T6	B06	0.273	0.222	0.222	0.009	2.75	0.025
T6HS12	T6	B07	0.262	0.211	0.211	0.008	2.75	0.023
T6HS12	T6	B08	0.271	0.220	0.220	0.009	2.75	0.024
T6HS13	T6	B09	0.261	0.210	0.210	0.008	2.75	0.023
T6HS13	T6	B10	0.272	0.221	0.221	0.009	2.75	0.024
T6HS14	T6	B11	0.190	0.139	kleiner LLOQ	#VALUE!	2.75	#VALUE!
T6HS14	T6	B12	0.194	0.143	kleiner LLOQ	#VALUE!	2.75	#VALUE!
0	T6	C01	0	0	kleiner LLOQ	#VALUE!	1	#VALUE!

Plate: T6

	1	2	3	4	5	6	7	8	9	10	11	12
A					0.873	0.588	0.744	0.778	0.518	0.513	0.634	0.620
B					0.274	0.273	0.262	0.271	0.261	0.272	0.190	0.194
C											0.232	0.227
D												
E												
F												
G							0.051	0.050	0.050	0.052	0.051	0.051
H							0.050	0.050	0.050	0.050	0.049	0.049

dil8*dil2,75=dil22

Plattenbelegung

	1	2	3	4	5	6	7	8	9	10	11	12
A					T6HN1	T6HN1	T6HN2	T6HN2	T6HN3	T6HN3	T6HN4	T6HN4
B					T6HS11	T6HS11	T6HS12	T6HS12	T6HS13	T6HS13	T6HS14	T6HS14
C					T6HS21	T6HS21	T6HS22	T6HS22	T6HS23	T6HS23	T6HS24	T6HS24
D												
E												

0	T6	C02	0	0	kleiner LLOQ	#VALUE!	1	#VALUE!
0	T6	C03	0	0	kleiner LLOQ	#VALUE!	1	#VALUE!
0	T6	C04	0	0	kleiner LLOQ	#VALUE!	1	#VALUE!
T6HS21	T6	C05	0	0	kleiner LLOQ	#VALUE!	1	#VALUE!
T6HS21	T6	C06	0	0	kleiner LLOQ	#VALUE!	1	#VALUE!
T6HS22	T6	C07	0	0	kleiner LLOQ	#VALUE!	1	#VALUE!
T6HS22	T6	C08	0	0	kleiner LLOQ	#VALUE!	1	#VALUE!
T6HS23	T6	C09	0	0	kleiner LLOQ	#VALUE!	1	#VALUE!
T6HS23	T6	C10	0	0	kleiner LLOQ	#VALUE!	1	#VALUE!
T6HS24	T6	C11	0.232	0.181	0.181	0.007	2.75	0.019
T6HS24	T6	C12	0.227	0.176	0.176	0.007	2.75	0.019
0	T6	D01	0	0	kleiner LLOQ	#VALUE!	1	#VALUE!
0	T6	D02	0	0	kleiner LLOQ	#VALUE!	1	#VALUE!
0	T6	D03	0	0	kleiner LLOQ	#VALUE!	1	#VALUE!
0	T6	D04	0	0	kleiner LLOQ	#VALUE!	1	#VALUE!
0	T6	D05	0	0	kleiner LLOQ	#VALUE!	1	#VALUE!
0	T6	D06	0	0	kleiner LLOQ	#VALUE!	1	#VALUE!
0	T6	D07	0	0	kleiner LLOQ	#VALUE!	1	#VALUE!
0	T6	D08	0	0	kleiner LLOQ	#VALUE!	1	#VALUE!
0	T6	D09	0	0	kleiner LLOQ	#VALUE!	1	#VALUE!
0	T6	D10	0	0	kleiner LLOQ	#VALUE!	1	#VALUE!
0	T6	D11	0	0	kleiner LLOQ	#VALUE!	1	#VALUE!
0	T6	D12	0	0	kleiner LLOQ	#VALUE!	1	#VALUE!
0	T6	E01	0	0	kleiner LLOQ	#VALUE!	1	#VALUE!
0	T6	E02	0	0	kleiner LLOQ	#VALUE!	1	#VALUE!
0	T6	E03	0	0	kleiner LLOQ	#VALUE!	1	#VALUE!
0	T6	E04	0	0	kleiner LLOQ	#VALUE!	1	#VALUE!
0	T6	E05	0	0	kleiner LLOQ	#VALUE!	1	#VALUE!
0	T6	E06	0	0	kleiner LLOQ	#VALUE!	1	#VALUE!
0	T6	E07	0	0	kleiner LLOQ	#VALUE!	1	#VALUE!
0	T6	E08	0	0	kleiner LLOQ	#VALUE!	1	#VALUE!
0	T6	E09	0	0	kleiner LLOQ	#VALUE!	1	#VALUE!
0	T6	E10	0	0	kleiner LLOQ	#VALUE!	1	#VALUE!
0	T6	E11	0	0	kleiner LLOQ	#VALUE!	1	#VALUE!
0	T6	E12	0	0	kleiner LLOQ	#VALUE!	1	#VALUE!
0	T6	F01	0	0	kleiner LLOQ	#VALUE!	1	#VALUE!
0	T6	F02	0	0	kleiner LLOQ	#VALUE!	1	#VALUE!
0	T6	F03	0	0	kleiner LLOQ	#VALUE!	1	#VALUE!
0	T6	F04	0	0	kleiner LLOQ	#VALUE!	1	#VALUE!
0	T6	F05	0	0	kleiner LLOQ	#VALUE!	1	#VALUE!
0	T6	F06	0	0	kleiner LLOQ	#VALUE!	1	#VALUE!
0	T6	F07	0	0	kleiner LLOQ	#VALUE!	1	#VALUE!
0	T6	F08	0	0	kleiner LLOQ	#VALUE!	1	#VALUE!
0	T6	F09	0	0	kleiner LLOQ	#VALUE!	1	#VALUE!
0	T6	F10	0	0	kleiner LLOQ	#VALUE!	1	#VALUE!
0	T6	F11	0	0	kleiner LLOQ	#VALUE!	1	#VALUE!
0	T6	F12	0	0	kleiner LLOQ	#VALUE!	1	#VALUE!
0	T6	G01	0	0	kleiner LLOQ	#VALUE!	1	#VALUE!
0	T6	G02	0	0	kleiner LLOQ	#VALUE!	1	#VALUE!
0	T6	G03	0	0	kleiner LLOQ	#VALUE!	1	#VALUE!
0	T6	G04	0	0	kleiner LLOQ	#VALUE!	1	#VALUE!
0	T6	G05	0	0	kleiner LLOQ	#VALUE!	1	#VALUE!
0	T6	G06	0	0	kleiner LLOQ	#VALUE!	1	#VALUE!
Blank PEG	T6	G07	0	0	kleiner LLOQ	#VALUE!	2.75	#VALUE!
Blank PEG	T6	G08	0	0	kleiner LLOQ	#VALUE!	2.75	#VALUE!
Blank PEG	T6	G09	0	0	kleiner LLOQ	#VALUE!	2.75	#VALUE!
Blank PEG	T6	G10	0	0	kleiner LLOQ	#VALUE!	2.75	#VALUE!
Blank PEG	T6	G11	0	0	kleiner LLOQ	#VALUE!	2.75	#VALUE!
Blank PEG	T6	G12	0	0	kleiner LLOQ	#VALUE!	2.75	#VALUE!
0	T6	H01	0	0	kleiner LLOQ	#VALUE!	1	#VALUE!
0	T6	H02	0	0	kleiner LLOQ	#VALUE!	1	#VALUE!
0	T6	H03	0	0	kleiner LLOQ	#VALUE!	1	#VALUE!
0	T6	H04	0	0	kleiner LLOQ	#VALUE!	1	#VALUE!
0	T6	H05	0	0	kleiner LLOQ	#VALUE!	1	#VALUE!
0	T6	H06	0	0	kleiner LLOQ	#VALUE!	1	#VALUE!
Blank no PEG	T6	H07	0	0	kleiner LLOQ	#VALUE!	2.75	#VALUE!
Blank no PEG	T6	H08	0	0	kleiner LLOQ	#VALUE!	2.75	#VALUE!
Blank no PEG	T6	H09	0	0	kleiner LLOQ	#VALUE!	2.75	#VALUE!
Blank no PEG	T6	H10	0	0	kleiner LLOQ	#VALUE!	2.75	#VALUE!
Blank no PEG	T6	H11	0	0	kleiner LLOQ	#VALUE!	2.75	#VALUE!
Blank no PEG	T6	H12	0	0	kleiner LLOQ	#VALUE!	2.75	#VALUE!
0	T7	A01	0	0	kleiner LLOQ	#VALUE!	1	#VALUE!
0	T7	A02	0	0	kleiner LLOQ	#VALUE!	1	#VALUE!
0	T7	A03	0	0	kleiner LLOQ	#VALUE!	1	#VALUE!
0	T7	A04	0	0	kleiner LLOQ	#VALUE!	1	#VALUE!
T7HN1	T7	A05	0.292	0.241	0.241	0.010	19.25	0.189

F												
G							Blank PEG	Blank PEG	Blank PEG	Blank PEG	Blank PEG	Blank PEG
H							Blank no PEG	Blank no PEG	Blank no PEG	Blank no PEG	Blank no PEG	Blank no PEG

Comment:

Date Operator

Date Control

T7HN1	T7	A06	0.301	0.250	0.250	0.010	19.25	0.198
T7HN2	T7	A07	0.404	0.353	0.353	0.015	19.25	0.292
T7HN2	T7	A08	0.394	0.343	0.343	0.015	19.25	0.283
T7HN3	T7	A09	0.647	0.596	0.596	0.027	2.75	0.073
T7HN3	T7	A10	0.835	0.784	0.784	0.036	2.75	0.098
T7HN4	T7	A11	0.282	0.231	0.231	0.009	2.75	0.026
T7HN4	T7	A12	0.295	0.244	0.244	0.010	2.75	0.027
0	T7	B01	0	0	kleiner LLOQ	#VALUE!	1	#VALUE!
0	T7	B02	0	0	kleiner LLOQ	#VALUE!	1	#VALUE!
0	T7	B03	0	0	kleiner LLOQ	#VALUE!	1	#VALUE!
0	T7	B04	0	0	kleiner LLOQ	#VALUE!	1	#VALUE!
T7HS11	T7	B05	0	0	kleiner LLOQ	#VALUE!	2.75	#VALUE!
T7HS11	T7	B06	0	0	kleiner LLOQ	#VALUE!	2.75	#VALUE!
T7HS12	T7	B07	0	0	kleiner LLOQ	#VALUE!	2.75	#VALUE!
T7HS12	T7	B08	0	0	kleiner LLOQ	#VALUE!	2.75	#VALUE!
T7HS13	T7	B09	0	0	kleiner LLOQ	#VALUE!	2.75	#VALUE!
T7HS13	T7	B10	0	0	kleiner LLOQ	#VALUE!	2.75	#VALUE!
T7HS14	T7	B11	0	0	kleiner LLOQ	#VALUE!	2.75	#VALUE!
T7HS14	T7	B12	0	0	kleiner LLOQ	#VALUE!	2.75	#VALUE!
0	T7	C01	0	0	kleiner LLOQ	#VALUE!	1	#VALUE!
0	T7	C02	0	0	kleiner LLOQ	#VALUE!	1	#VALUE!
0	T7	C03	0	0	kleiner LLOQ	#VALUE!	1	#VALUE!
0	T7	C04	0	0	kleiner LLOQ	#VALUE!	1	#VALUE!
T7HS21	T7	C05	0.522	0.471	0.471	0.021	8.25	0.171
T7HS21	T7	C06	0.505	0.454	0.454	0.020	8.25	0.165
T7HS22	T7	C07	0.598	0.547	0.547	0.024	8.25	0.201
T7HS22	T7	C08	0.588	0.537	0.537	0.024	8.25	0.197
T7HS23	T7	C09	0	0	kleiner LLOQ	#VALUE!	1	#VALUE!
T7HS23	T7	C10	0	0	kleiner LLOQ	#VALUE!	1	#VALUE!
T7HS24	T7	C11	0.219	0.168	0.168	0.006	2.75	0.018
T7HS24	T7	C12	0.236	0.185	0.185	0.007	2.75	0.020
0	T7	D01	0	0	kleiner LLOQ	#VALUE!	1	#VALUE!
0	T7	D02	0	0	kleiner LLOQ	#VALUE!	1	#VALUE!
0	T7	D03	0	0	kleiner LLOQ	#VALUE!	1	#VALUE!
0	T7	D04	0	0	kleiner LLOQ	#VALUE!	1	#VALUE!
0	T7	D05	0	0	kleiner LLOQ	#VALUE!	1	#VALUE!
0	T7	D06	0	0	kleiner LLOQ	#VALUE!	1	#VALUE!
0	T7	D07	0	0	kleiner LLOQ	#VALUE!	1	#VALUE!
0	T7	D08	0	0	kleiner LLOQ	#VALUE!	1	#VALUE!
0	T7	D09	0	0	kleiner LLOQ	#VALUE!	1	#VALUE!
0	T7	D10	0	0	kleiner LLOQ	#VALUE!	1	#VALUE!
0	T7	D11	0	0	kleiner LLOQ	#VALUE!	1	#VALUE!
0	T7	D12	0	0	kleiner LLOQ	#VALUE!	1	#VALUE!
0	T7	E01	0	0	kleiner LLOQ	#VALUE!	1	#VALUE!
0	T7	E02	0	0	kleiner LLOQ	#VALUE!	1	#VALUE!
0	T7	E03	0	0	kleiner LLOQ	#VALUE!	1	#VALUE!
0	T7	E04	0	0	kleiner LLOQ	#VALUE!	1	#VALUE!
0	T7	E05	0	0	kleiner LLOQ	#VALUE!	1	#VALUE!
0	T7	E06	0	0	kleiner LLOQ	#VALUE!	1	#VALUE!
0	T7	E07	0	0	kleiner LLOQ	#VALUE!	1	#VALUE!
0	T7	E08	0	0	kleiner LLOQ	#VALUE!	1	#VALUE!
0	T7	E09	0	0	kleiner LLOQ	#VALUE!	1	#VALUE!
0	T7	E10	0	0	kleiner LLOQ	#VALUE!	1	#VALUE!
0	T7	E11	0	0	kleiner LLOQ	#VALUE!	1	#VALUE!
0	T7	E12	0	0	kleiner LLOQ	#VALUE!	1	#VALUE!
0	T7	F01	0	0	kleiner LLOQ	#VALUE!	1	#VALUE!
0	T7	F02	0	0	kleiner LLOQ	#VALUE!	1	#VALUE!
0	T7	F03	0	0	kleiner LLOQ	#VALUE!	1	#VALUE!
0	T7	F04	0	0	kleiner LLOQ	#VALUE!	1	#VALUE!
0	T7	F05	0	0	kleiner LLOQ	#VALUE!	1	#VALUE!
0	T7	F06	0	0	kleiner LLOQ	#VALUE!	1	#VALUE!
0	T7	F07	0	0	kleiner LLOQ	#VALUE!	1	#VALUE!
0	T7	F08	0	0	kleiner LLOQ	#VALUE!	1	#VALUE!
0	T7	F09	0	0	kleiner LLOQ	#VALUE!	1	#VALUE!
0	T7	F10	0	0	kleiner LLOQ	#VALUE!	1	#VALUE!
0	T7	F11	0	0	kleiner LLOQ	#VALUE!	1	#VALUE!
0	T7	F12	0	0	kleiner LLOQ	#VALUE!	1	#VALUE!
0	T7	G01	0	0	kleiner LLOQ	#VALUE!	1	#VALUE!
0	T7	G02	0	0	kleiner LLOQ	#VALUE!	1	#VALUE!
0	T7	G03	0	0	kleiner LLOQ	#VALUE!	1	#VALUE!
0	T7	G04	0	0	kleiner LLOQ	#VALUE!	1	#VALUE!
0	T7	G05	0	0	kleiner LLOQ	#VALUE!	1	#VALUE!
0	T7	G06	0	0	kleiner LLOQ	#VALUE!	1	#VALUE!
Blank PEG	T7	G07	0	0	kleiner LLOQ	#VALUE!	2.75	#VALUE!

Plate: T7

	1	2	3	4	5	6	7	8	9	10	11	12
A					0.292	0.301	0.404	0.394	0.647	0.835	0.282	0.295
B					0.199	0.200	0.188	0.186	0.204	0.205	0.121	0.122
C					0.522	0.505	0.598	0.588			0.219	0.236
D												
E												
F												
G							0.051	0.051	0.051	0.053	0.052	0.051
H							0.050	0.050	0.050	0.050	0.050	0.050

Plattenbelegung

	1	2	3	4	5	6	7	8	9	10	11	12
A					T7HN1	T7HN1	T7HN2	T7HN2	T7HN3	T7HN3	T7HN4	T7HN4
B					T7HS11	T7HS11	T7HS12	T7HS12	T7HS13	T7HS13	T7HS14	T7HS14
C					T7HS21	T7HS21	T7HS22	T7HS22	T7HS23	T7HS23	T7HS24	T7HS24
D												
E												
F												
G							Blank PEG	Blank PEG	Blank PEG	Blank PEG	Blank PEG	Blank PEG
H							Blank no PEG	Blank no PEG	Blank no PEG	Blank no PEG	Blank no PEG	Blank no PEG

dil3*dil2,75=8,25
dil7*dil2,75=19,25

Comment:

Date Operator

Date Control

Blank PEG	T7	G08	0	0	kleiner LLOQ	#VALUE!	2.75	#VALUE!
Blank PEG	T7	G09	0	0	kleiner LLOQ	#VALUE!	2.75	#VALUE!
Blank PEG	T7	G10	0	0	kleiner LLOQ	#VALUE!	2.75	#VALUE!
Blank PEG	T7	G11	0	0	kleiner LLOQ	#VALUE!	2.75	#VALUE!
Blank PEG	T7	G12	0	0	kleiner LLOQ	#VALUE!	2.75	#VALUE!
0	T7	H01	0	0	kleiner LLOQ	#VALUE!	1	#VALUE!
0	T7	H02	0	0	kleiner LLOQ	#VALUE!	1	#VALUE!
0	T7	H03	0	0	kleiner LLOQ	#VALUE!	1	#VALUE!
0	T7	H04	0	0	kleiner LLOQ	#VALUE!	1	#VALUE!
0	T7	H05	0	0	kleiner LLOQ	#VALUE!	1	#VALUE!
0	T7	H06	0	0	kleiner LLOQ	#VALUE!	1	#VALUE!
Blank no PEG	T7	H07	0	0	kleiner LLOQ	#VALUE!	2.75	#VALUE!
Blank no PEG	T7	H08	0	0	kleiner LLOQ	#VALUE!	2.75	#VALUE!
Blank no PEG	T7	H09	0	0	kleiner LLOQ	#VALUE!	2.75	#VALUE!
Blank no PEG	T7	H10	0	0	kleiner LLOQ	#VALUE!	2.75	#VALUE!
Blank no PEG	T7	H11	0	0	kleiner LLOQ	#VALUE!	2.75	#VALUE!
Blank no PEG	T7	H12	0	0	kleiner LLOQ	#VALUE!	2.75	#VALUE!

**Project number**		**F-120**	**Apparatus**	**Wallac Victor**	**Operator**	**IsBa**
**GLP Study (Number)**		**n.a.**	**Protocol (Instrument meth**	**LDH test 2016**	**Date of preparation**	**19-04-18**
**hot substance**	**isotope**	**n.a.**	**File name (results)**	**IsBa_180419/20_LDH_full_**	**Date of measurement**	**19-04-18**
	**name**	**n.a.**	**Kind of well plate**	**96 well**	**shaking time [min]**	**30**
	**ACB-ID**	**n.a.**	**sample volume [µL]**	**100**	**stirring rate (Target) [rpm]**	**150**
	**Batch number**	**n.a.**	**Cocktail volume [µL]**	**175**	**Kind of measurement**	**UV-vis**
**cold substance**	**name**	**LDH**	**ACB-ID of cocktail**	**n.a.**	**Wave length [nm]**	**450**
	**ACB-ID**	**L23982R**	**Matrix**	**DMEM (from powder)+PE(**	**Remarks**	**Cocktail 100µl RM, 75µl STOP**
	**Batch number**	**SLBK6345V**	**Blank description**	**DMEM/PEG, H2O**	**Remarks**	**7 standards split low/high**
**n.a.**		**n.a.**	**Pipettes (No. / volume)**	**50-200µl**	**Remarks**	**KLP4 common for both**
**n.a.**		**n.a.**	**Pipettes (No. / volume)**	**n.a.**	**Remarks**	**n.a.**

Messdaten (diese Tabelle in Bericht übernehmen)

Sample name *	concentration (theor.) *	measured data	measured data	measured data	mean measured	SD	RSD	Blank *	measured data after * Blank subtraction	concentration (calc.) *	Deviation *	Residuen
	[µg/mL]	[AU]	[AU]	[AU]	[AU]	[AU]	[%]	[AU]		[µg/mL]	[%]	
KLP1	0.148	1.579	1.652	1.627	1.62	0.03	1.87	0.047	1.568	0.148	-0.24	0.00
KLP2	0.114	1.357	1.443	1.372	1.39	0.04	2.70	0.054	1.340	0.110	-3.40	0.00
KLP3	0.074	1.272	1.193	1.228	1.23	0.03	2.63	0.051	1.180	0.084	13.04	0.01
KLP4	0.041	0.959	0.972	0.883	0.94	0.04	4.19	0.051	0.887	0.035	-13.32	-0.01
KLP5								0.052				
KLP6								0.051				
KLP7												
KLP8												

Statistical data

Geradensteigung	Slope	m	6.05
Y-Achsenabschnitt	Y-Intercept	b	0.67
Standardabw. Geradensteigung	SD-Slope	s_m	0.620291227
Standardabw. Achsenabschnittes	SD-Y-Intercept	s_b	0.063694294
Anzahl Messpunkte	number of measuring points	n	4
Quadratsumme	sum of squares	Qxx	0.006578604
Bereichsmitte			0.094336926
Freiheitsgrade	degree of freedom	f	2
Student-t-Faktor für (P = 95 %; f = n-2)	Student-t-factor for (P = 95 %; f = n-2)	t	4.303
Vertrauensbereich Steig. (95 %) Obergrenze		$m + VB_m$	8.722319527
Vertrauensbereich Steig. (95 %) Untergrenze		$m - VB_m$	3.384093228
Vertrauensbereich Achsenabschnitt (95 %) Og.		$b + Vb_b$	0.946914615
Vertrauensbereich Achsenabschnitt (95 %) Ug.		$b - Vb_b$	0.398761518
Korrelationskoeffizient	correlation coefficient	r	0.9897
Bestimmtheitsmaß	determiantion coefficient	r^2	0.9794
Reststandardabweichung		s_0	0.050310948
Summe Restquadrate		sd	2.895583345
Verfahrensstandardabw.		s_{x0}	0.008311454
Rel. Verfahrensstandardabw. %		V_{x0}	8.810393374

mean Blank	**0**	
SD Blank	0.00	
RSD Blank	3.79	x=
x*SD (LLOQ)	0	5
x*SD (LOD)	0	3
**LLOQ (AU)**	**0**	
**LOD (AU)**	**0**	
ULOQ	1.568	
LLOQ (Lin)	0.887	

Evaluation / Comment

LDH linearity valid with 7 standards, split in lin high and lin low, each with 4 standards and KLP4 common standard for both. R² 0,9981, deviations for both between -13.34% and +13.04%

Date Operator Date Control

Figure 1 Linearity

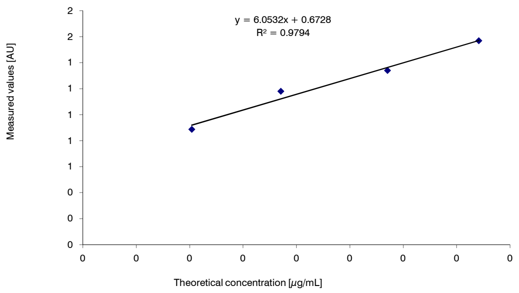

Figure 2 Method validation Residuen Plot

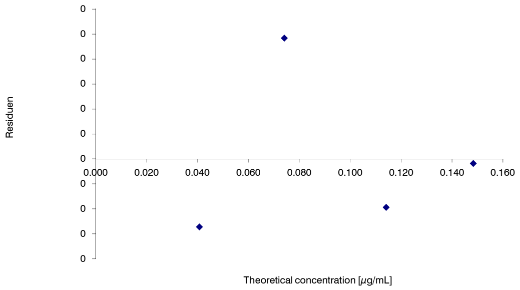

Evaluation / Comment

lin passed

Date	Operator	Date	Control

**Project number**		F-120	**Apparatus**	**Wallac Victor**	**Operator**	**IsBa**	**LLOQ [AU]**	0.887
**GLP Study (Number)**		n.a.	**Protocol (Instrument metho**	**LDH test 2016**	**Date of preparation**	**43209**	**ULOQ [AU]**	1.568
	**Isotope**	n.a.	**File name (results)**	**IsBa_180419/20_LDH_full_val_1/2/**	**Date of measurement**	**43209**	**Blank**	0.051
**hot**	**Name**	n.a.	**Kind of well plate**	**96 well**	**Shaking time [min]**	**30**		n.a.
**substance**	**ACB-ID**	n.a.	**sample volume [µL]**	**100**	**Stirring rate (Target) [rpm**	**150**		n.a.
	**Batch number**	n.a.	**Cocktail volume [µL]**	**175**	**Kind of measurement**	**UV-vis**	**Pipettes (No. / volume)**	n.a.
**cold**	**Name**	LDH	**ACB-ID of cocktail**	**n.a.**	**Wave length [nm]**	**450**	**Pipettes (No. / volume)**	n.a.
**substance**	**ACB-ID**	L23982R	**Matrix**	**DMEM (from powder)+PEG**	**Remarks**	**Sui mitgemessen auf der Platte aber Au**	**Pipettes (No. / volume)**	n.a.
	**Batch number**	SLBK6345V	**Blank description**	**DMEM/PEG, H2O**	**Remarks**	n.a.	**Remarks**	n.a.
		n.a.						
		n.a.						

Sample name	Plate	Position	[AU]	[AU]-Blank	Rating	[µg/mL]	Dilution factor	[µg/mL]
0	T4	A01	0	0	kleiner LLOQ	#VALUE!	1	#VALUE!
0	T4	A02	0	0	kleiner LLOQ	#VALUE!	1	#VALUE!
0	T4	A03	0	0	kleiner LLOQ	#VALUE!	1	#VALUE!
0	T4	A04	0	0	kleiner LLOQ	#VALUE!	1	#VALUE!
T4HN1	T4	A05	1.128	1.077	1.077	0.067	2.75	0.184
T4HN1	T4	A06	1.099	1.048	1.048	0.062	2.75	0.171
T4HN2	T4	A07	1.007	0.956	0.956	0.047	24.75	1.158
T4HN2	T4	A08	0.969	0.918	0.918	0.040	24.75	1.002
T4HN3	T4	A09	0.000	-0.051	kleiner LLOQ	#VALUE!	1	#VALUE!
T4HN3	T4	A10	0.000	-0.051	kleiner LLOQ	#VALUE!	1	#VALUE!
T4HN4	T4	A11	0	0	kleiner LLOQ	#VALUE!	1	#VALUE!
T4HN4	T4	A12	0	0	kleiner LLOQ	#VALUE!	1	#VALUE!
0	T4	B01	0	0	kleiner LLOQ	#VALUE!	1	#VALUE!
0	T4	B02	0	0	kleiner LLOQ	#VALUE!	1	#VALUE!
0	T4	B03	0	0	kleiner LLOQ	#VALUE!	1	#VALUE!
0	T4	B04	0	0	kleiner LLOQ	#VALUE!	1	#VALUE!
T4HS11	T4	B05	1.143	1.093	1.093	0.069	24.75	1.716
T4HS11	T4	B06	0	0	kleiner LLOQ	#VALUE!	1	#VALUE!
T4HS12	T4	B07	0	0	kleiner LLOQ	#VALUE!	1	#VALUE!
T4HS12	T4	B08	0	0	kleiner LLOQ	#VALUE!	1	#VALUE!
T4HS13	T4	B09	0	0	kleiner LLOQ	#VALUE!	1	#VALUE!
T4HS13	T4	B10	0	0	kleiner LLOQ	#VALUE!	1	#VALUE!
T4HS14	T4	B11	0	0	kleiner LLOQ	#VALUE!	1	#VALUE!
T4HS14	T4	B12	0	0	kleiner LLOQ	#VALUE!	1	#VALUE!
0	T4	C01	0	0	kleiner LLOQ	#VALUE!	1	#VALUE!
0	T4	C02	0	0	kleiner LLOQ	#VALUE!	1	#VALUE!
0	T4	C03	0	0	kleiner LLOQ	#VALUE!	1	#VALUE!
0	T4	C04	0	0	kleiner LLOQ	#VALUE!	1	#VALUE!
T4HS21	T4	C05	0	0	kleiner LLOQ	#VALUE!	1	#VALUE!
T4HS21	T4	C06	0	0	kleiner LLOQ	#VALUE!	1	#VALUE!
T4HS22	T4	C07	0	0	kleiner LLOQ	#VALUE!	1	#VALUE!
T4HS22	T4	C08	0	0	kleiner LLOQ	#VALUE!	1	#VALUE!
T4HS23	T4	C09	0	0	kleiner LLOQ	#VALUE!	1	#VALUE!
T4HS23	T4	C10	0	0	kleiner LLOQ	#VALUE!	1	#VALUE!
T4HS24	T4	C11	0	0	kleiner LLOQ	#VALUE!	1	#VALUE!
T4HS24	T4	C12	0	0	kleiner LLOQ	#VALUE!	1	#VALUE!
0	T4	D01	0	0	kleiner LLOQ	#VALUE!	1	#VALUE!
0	T4	D02	0	0	kleiner LLOQ	#VALUE!	1	#VALUE!
0	T4	D03	0	0	kleiner LLOQ	#VALUE!	1	#VALUE!
0	T4	D04	0	0	kleiner LLOQ	#VALUE!	1	#VALUE!
0	T4	D05	0	0	kleiner LLOQ	#VALUE!	1	#VALUE!
0	T4	D06	0	0	kleiner LLOQ	#VALUE!	1	#VALUE!
0	T4	D07	0	0	kleiner LLOQ	#VALUE!	1	#VALUE!
0	T4	D08	0	0	kleiner LLOQ	#VALUE!	1	#VALUE!
0	T4	D09	0	0	kleiner LLOQ	#VALUE!	1	#VALUE!
0	T4	D10	0	0	kleiner LLOQ	#VALUE!	1	#VALUE!
0	T4	D11	0	0	kleiner LLOQ	#VALUE!	1	#VALUE!
0	T4	D12	0	0	kleiner LLOQ	#VALUE!	1	#VALUE!

Plate: T4

	1	2	3	4	5	6	7	8	9	10	11	12
A					1.128	1.099	1.007	0.969				
B					1.143							
C												
D												
E												
F												
G							0.052	0.054	0.052	0.052	0.052	0.051
H							0.050	0.050	0.053	0.050	0.051	0.050

Plattenbelegung

	1	2	3	4	5	6	7	8	9	10	11	12
A					T4HN1	T4HN1	T4HN2	T4HN2	T4HN3	T4HN3	T4HN4	T4HN4
B					T4HS11	T4HS11	T4HS12	T4HS12	T4HS13	T4HS13	T4HS14	T4HS14
C					T4HS21	T4HS21	T4HS22	T4HS22	T4HS23	T4HS23	T4HS24	T4HS24
D												
E												
F												
G							Blank PEG	Blank PEG	Blank PEG	Blank PEG	Blank PEG	Blank PEG
H							Blank no PEG	Blank no PEG	Blank no PEG	Blank no PEG	Blank no PEG	Blank no PEG

dil 9*dil2,75=dil.24.75

Comment:

Samples aufgeteilt in lin high und low:niedriger konzentrierte Samples bei lin low zu finden.

Date Operator

Date Control

0	T4	E01	0	0	kleiner LLOQ	#VALUE!	1	#VALUE!
0	T4	E02	0	0	kleiner LLOQ	#VALUE!	1	#VALUE!
0	T4	E03	0	0	kleiner LLOQ	#VALUE!	1	#VALUE!
0	T4	E04	0	0	kleiner LLOQ	#VALUE!	1	#VALUE!
0	T4	E05	0	0	kleiner LLOQ	#VALUE!	1	#VALUE!
0	T4	E06	0	0	kleiner LLOQ	#VALUE!	1	#VALUE!
0	T4	E07	0	0	kleiner LLOQ	#VALUE!	1	#VALUE!
0	T4	E08	0	0	kleiner LLOQ	#VALUE!	1	#VALUE!
0	T4	E09	0	0	kleiner LLOQ	#VALUE!	1	#VALUE!
0	T4	E10	0	0	kleiner LLOQ	#VALUE!	1	#VALUE!
0	T4	E11	0	0	kleiner LLOQ	#VALUE!	1	#VALUE!
0	T4	E12	0	0	kleiner LLOQ	#VALUE!	1	#VALUE!
0	T4	F01	0	0	kleiner LLOQ	#VALUE!	1	#VALUE!
0	T4	F02	0	0	kleiner LLOQ	#VALUE!	1	#VALUE!
0	T4	F03	0	0	kleiner LLOQ	#VALUE!	1	#VALUE!
0	T4	F04	0	0	kleiner LLOQ	#VALUE!	1	#VALUE!
0	T4	F05	0	0	kleiner LLOQ	#VALUE!	1	#VALUE!
0	T4	F06	0	0	kleiner LLOQ	#VALUE!	1	#VALUE!
0	T4	F07	0	0	kleiner LLOQ	#VALUE!	1	#VALUE!
0	T4	F08	0	0	kleiner LLOQ	#VALUE!	1	#VALUE!
0	T4	F09	0	0	kleiner LLOQ	#VALUE!	1	#VALUE!
0	T4	F10	0	0	kleiner LLOQ	#VALUE!	1	#VALUE!
0	T4	F11	0	0	kleiner LLOQ	#VALUE!	1	#VALUE!
0	T4	F12	0	0	kleiner LLOQ	#VALUE!	1	#VALUE!
0	T4	G01	0	0	kleiner LLOQ	#VALUE!	1	#VALUE!
0	T4	G02	0	0	kleiner LLOQ	#VALUE!	1	#VALUE!
0	T4	G03	0	0	kleiner LLOQ	#VALUE!	1	#VALUE!
0	T4	G04	0	0	kleiner LLOQ	#VALUE!	1	#VALUE!
0	T4	G05	0	0	kleiner LLOQ	#VALUE!	1	#VALUE!
0	T4	G06	0	0	kleiner LLOQ	#VALUE!	1	#VALUE!
Blank PEG	T4	G07	0.052	0.001	kleiner LLOQ	#VALUE!	2.75	#VALUE!
Blank PEG	T4	G08	0.054	0.003	kleiner LLOQ	#VALUE!	2.75	#VALUE!
Blank PEG	T4	G09	0.052	0.001	kleiner LLOQ	#VALUE!	2.75	#VALUE!
Blank PEG	T4	G10	0.052	0.001	kleiner LLOQ	#VALUE!	2.75	#VALUE!
Blank PEG	T4	G11	0.052	0.001	kleiner LLOQ	#VALUE!	2.75	#VALUE!
Blank PEG	T4	G12	0.051	0.000	kleiner LLOQ	#VALUE!	2.75	#VALUE!
0	T4	H01	0	0	kleiner LLOQ	#VALUE!	1	#VALUE!
0	T4	H02	0	0	kleiner LLOQ	#VALUE!	1	#VALUE!
0	T4	H03	0	0	kleiner LLOQ	#VALUE!	1	#VALUE!
0	T4	H04	0	0	kleiner LLOQ	#VALUE!	1	#VALUE!
0	T4	H05	0	0	kleiner LLOQ	#VALUE!	1	#VALUE!
0	T4	H06	0	0	kleiner LLOQ	#VALUE!	1	#VALUE!
Blank no PEG	T4	H07	0.050	-0.001	kleiner LLOQ	#VALUE!	2.75	#VALUE!
Blank no PEG	T4	H08	0.050	-0.001	kleiner LLOQ	#VALUE!	2.75	#VALUE!
Blank no PEG	T4	H09	0.053	0.002	kleiner LLOQ	#VALUE!	2.75	#VALUE!
Blank no PEG	T4	H10	0.050	-0.001	kleiner LLOQ	#VALUE!	2.75	#VALUE!
Blank no PEG	T4	H11	0.051	0.000	kleiner LLOQ	#VALUE!	2.75	#VALUE!
Blank no PEG	T4	H12	0.050	-0.001	kleiner LLOQ	#VALUE!	2.75	#VALUE!

0	T5	A01	0	0	kleiner LLOQ	#VALUE!	1	#VALUE!
0	T5	A02	0	0	kleiner LLOQ	#VALUE!	1	#VALUE!
0	T5	A03	0	0	kleiner LLOQ	#VALUE!	1	#VALUE!
0	T5	A04	0	0	kleiner LLOQ	#VALUE!	1	#VALUE!
T5HN1	T5	A05	0	0	kleiner LLOQ	#VALUE!	1	#VALUE!
T5HN1	T5	A06	0	0	kleiner LLOQ	#VALUE!	1	#VALUE!
T5HN2	T5	A07	1.096	1.045	1.045	0.061	27.5	1.690
T5HN2	T5	A08	1.121	1.070	1.070	0.066	27.5	1.805
T5HN3	T5	A09	0.981	0.930	0.930	0.042	27.5	1.167
T5HN3	T5	A10	0.973	0.922	0.922	0.041	27.5	1.133
T5HN4	T5	A11	0	0	kleiner LLOQ	#VALUE!	1	#VALUE!
T5HN4	T5	A12	0	0	kleiner LLOQ	#VALUE!	1	#VALUE!
0	T5	B01	0	0	kleiner LLOQ	#VALUE!	1	#VALUE!
0	T5	B02	0	0	kleiner LLOQ	#VALUE!	1	#VALUE!
0	T5	B03	0	0	kleiner LLOQ	#VALUE!	1	#VALUE!
0	T5	B04	0	0	kleiner LLOQ	#VALUE!	1	#VALUE!
T5HS11	T5	B05	0	0	kleiner LLOQ	#VALUE!	1	#VALUE!
T5HS11	T5	B06	0	0	kleiner LLOQ	#VALUE!	1	#VALUE!
T5HS12	T5	B07	0	0	kleiner LLOQ	#VALUE!	1	#VALUE!
T5HS12	T5	B08	0	0	kleiner LLOQ	#VALUE!	1	#VALUE!
T5HS13	T5	B09	0	0	kleiner LLOQ	#VALUE!	1	#VALUE!
T5HS13	T5	B10	0	0	kleiner LLOQ	#VALUE!	1	#VALUE!
T5HS14	T5	B11	0	0	kleiner LLOQ	#VALUE!	1	#VALUE!
T5HS14	T5	B12	0	0	kleiner LLOQ	#VALUE!	1	#VALUE!
0	T5	C01	0	0	kleiner LLOQ	#VALUE!	1	#VALUE!
0	T5	C02	0	0	kleiner LLOQ	#VALUE!	1	#VALUE!
0	T5	C03	0	0	kleiner LLOQ	#VALUE!	1	#VALUE!
0	T5	C04	0	0	kleiner LLOQ	#VALUE!	1	#VALUE!
T5HS21	T5	C05	0	0	kleiner LLOQ	#VALUE!	1	#VALUE!
T5HS21	T5	C06	0	0	kleiner LLOQ	#VALUE!	1	#VALUE!
T5HS22	T5	C07	0	0	kleiner LLOQ	#VALUE!	1	#VALUE!
T5HS22	T5	C08	0	0	kleiner LLOQ	#VALUE!	1	#VALUE!
T5HS23	T5	C09	1.430	1.379	1.379	0.117	2.75	0.321
T5HS23	T5	C10	1.378	1.327	1.327	0.108	2.75	0.297
T5HS24	T5	C11	0	0	kleiner LLOQ	#VALUE!	1	#VALUE!
T5HS24	T5	C12	0	0	kleiner LLOQ	#VALUE!	1	#VALUE!
0	T5	D01	0	0	kleiner LLOQ	#VALUE!	1	#VALUE!
0	T5	D02	0	0	kleiner LLOQ	#VALUE!	1	#VALUE!
0	T5	D03	0	0	kleiner LLOQ	#VALUE!	1	#VALUE!
0	T5	D04	0	0	kleiner LLOQ	#VALUE!	1	#VALUE!
0	T5	D05	0	0	kleiner LLOQ	#VALUE!	1	#VALUE!
0	T5	D06	0	0	kleiner LLOQ	#VALUE!	1	#VALUE!
0	T5	D07	0	0	kleiner LLOQ	#VALUE!	1	#VALUE!
0	T5	D08	0	0	kleiner LLOQ	#VALUE!	1	#VALUE!
0	T5	D09	0	0	kleiner LLOQ	#VALUE!	1	#VALUE!
0	T5	D10	0	0	kleiner LLOQ	#VALUE!	1	#VALUE!
0	T5	D11	0	0	kleiner LLOQ	#VALUE!	1	#VALUE!
0	T5	D12	0	0	kleiner LLOQ	#VALUE!	1	#VALUE!

Plate: T5

	1	2	3	4	5	6	7	8	9	10	11	12
A							1.096	1.121	0.981	0.973		
B												
C									1.430	1.378		
D												
E												
F												
G							0.053	0.053	0.054	0.053	0.053	0.052
H							0.101	0.056	0.052	0.050	0.054	0.053

Plattenbelegung

	1	2	3	4	5	6	7	8	9	10	11	12
A					T5HN1	T5HN1	T5HN2	T5HN2	T5HN3	T5HN3	T5HN4	T5HN4
B					T5HS11	T5HS11	T5HS12	T5HS12	T5HS13	T5HS13	T5HS14	T5HS14
C					T5HS21	T5HS21	T5HS22	T5HS22	T5HS23	T5HS23	T5HS24	T5HS24
D												
E												
F												
G							Blank PEG	Blank PEG	Blank PEG	Blank PEG	Blank PEG	Blank PEG
H							Blank no PEG	Blank no PEG	Blank no PEG	Blank no PEG	Blank no PEG	Blank no PEG

dil10*dil2,75=dil.27,5

Comment:

Date Operator

Date Control

0	T5	E01	0	0	kleiner LLOQ	#VALUE!	1	#VALUE!
0	T5	E02	0	0	kleiner LLOQ	#VALUE!	1	#VALUE!
0	T5	E03	0	0	kleiner LLOQ	#VALUE!	1	#VALUE!
0	T5	E04	0	0	kleiner LLOQ	#VALUE!	1	#VALUE!
0	T5	E05	0	0	kleiner LLOQ	#VALUE!	1	#VALUE!
0	T5	E06	0	0	kleiner LLOQ	#VALUE!	1	#VALUE!
0	T5	E07	0	0	kleiner LLOQ	#VALUE!	1	#VALUE!
0	T5	E08	0	0	kleiner LLOQ	#VALUE!	1	#VALUE!
0	T5	E09	0	0	kleiner LLOQ	#VALUE!	1	#VALUE!
0	T5	E10	0	0	kleiner LLOQ	#VALUE!	1	#VALUE!
0	T5	E11	0	0	kleiner LLOQ	#VALUE!	1	#VALUE!
0	T5	E12	0	0	kleiner LLOQ	#VALUE!	1	#VALUE!
0	T5	F01	0	0	kleiner LLOQ	#VALUE!	1	#VALUE!
0	T5	F02	0	0	kleiner LLOQ	#VALUE!	1	#VALUE!
0	T5	F03	0	0	kleiner LLOQ	#VALUE!	1	#VALUE!
0	T5	F04	0	0	kleiner LLOQ	#VALUE!	1	#VALUE!
0	T5	F05	0	0	kleiner LLOQ	#VALUE!	1	#VALUE!
0	T5	F06	0	0	kleiner LLOQ	#VALUE!	1	#VALUE!
0	T5	F07	0	0	kleiner LLOQ	#VALUE!	1	#VALUE!
0	T5	F08	0	0	kleiner LLOQ	#VALUE!	1	#VALUE!
0	T5	F09	0	0	kleiner LLOQ	#VALUE!	1	#VALUE!
0	T5	F10	0	0	kleiner LLOQ	#VALUE!	1	#VALUE!
0	T5	F11	0	0	kleiner LLOQ	#VALUE!	1	#VALUE!
0	T5	F12	0	0	kleiner LLOQ	#VALUE!	1	#VALUE!
0	T5	G01	0	0	kleiner LLOQ	#VALUE!	1	#VALUE!
0	T5	G02	0	0	kleiner LLOQ	#VALUE!	1	#VALUE!
0	T5	G03	0	0	kleiner LLOQ	#VALUE!	1	#VALUE!
0	T5	G04	0	0	kleiner LLOQ	#VALUE!	1	#VALUE!
0	T5	G05	0	0	kleiner LLOQ	#VALUE!	1	#VALUE!
0	T5	G06	0	0	kleiner LLOQ	#VALUE!	1	#VALUE!
Blank PEG	T5	G07	0	0	kleiner LLOQ	#VALUE!	2.75	#VALUE!
Blank PEG	T5	G08	0	0	kleiner LLOQ	#VALUE!	2.75	#VALUE!
Blank PEG	T5	G09	0	0	kleiner LLOQ	#VALUE!	2.75	#VALUE!
Blank PEG	T5	G10	0	0	kleiner LLOQ	#VALUE!	2.75	#VALUE!
Blank PEG	T5	G11	0	0	kleiner LLOQ	#VALUE!	2.75	#VALUE!
Blank PEG	T5	G12	0	0	kleiner LLOQ	#VALUE!	2.75	#VALUE!
0	T5	H01	0	0	kleiner LLOQ	#VALUE!	1	#VALUE!
0	T5	H02	0	0	kleiner LLOQ	#VALUE!	1	#VALUE!
0	T5	H03	0	0	kleiner LLOQ	#VALUE!	1	#VALUE!
0	T5	H04	0	0	kleiner LLOQ	#VALUE!	1	#VALUE!
0	T5	H05	0	0	kleiner LLOQ	#VALUE!	1	#VALUE!
0	T5	H06	0	0	kleiner LLOQ	#VALUE!	1	#VALUE!
Blank no PEG	T5	H07	0	0	kleiner LLOQ	#VALUE!	2.75	#VALUE!
Blank no PEG	T5	H08	0	0	kleiner LLOQ	#VALUE!	2.75	#VALUE!
Blank no PEG	T5	H09	0	0	kleiner LLOQ	#VALUE!	2.75	#VALUE!
Blank no PEG	T5	H10	0	0	kleiner LLOQ	#VALUE!	2.75	#VALUE!
Blank no PEG	T5	H11	0	0	kleiner LLOQ	#VALUE!	2.75	#VALUE!
Blank no PEG	T5	H12	0	0	kleiner LLOQ	#VALUE!	2.75	#VALUE!
0	T6	A01	0	0	kleiner LLOQ	#VALUE!	1	#VALUE!
0	T6	A02	0	0	kleiner LLOQ	#VALUE!	1	#VALUE!
0	T6	A03	0	0	kleiner LLOQ	#VALUE!	1	#VALUE!
0	T6	A04	0	0	kleiner LLOQ	#VALUE!	1	#VALUE!
T6HN1	T6	A05	0	0	kleiner LLOQ	#VALUE!	1	#VALUE!
T6HN1	T6	A06	0	0	kleiner LLOQ	#VALUE!	1	#VALUE!
T6HN2	T6	A07	0	0	kleiner LLOQ	#VALUE!	1	#VALUE!
T6HN2	T6	A08	0	0	kleiner LLOQ	#VALUE!	1	#VALUE!
T6HN3	T6	A09	0	0	kleiner LLOQ	#VALUE!	1	#VALUE!
T6HN3	T6	A10	0	0	kleiner LLOQ	#VALUE!	1	#VALUE!
T6HN4	T6	A11	0	0	kleiner LLOQ	#VALUE!	1	#VALUE!
T6HN4	T6	A12	0	0	kleiner LLOQ	#VALUE!	1	#VALUE!
0	T6	B01	0	0	kleiner LLOQ	#VALUE!	1	#VALUE!
0	T6	B02	0	0	kleiner LLOQ	#VALUE!	1	#VALUE!
0	T6	B03	0	0	kleiner LLOQ	#VALUE!	1	#VALUE!
0	T6	B04	0	0	kleiner LLOQ	#VALUE!	1	#VALUE!
T6HS11	T6	B05	0	0	kleiner LLOQ	#VALUE!	1	#VALUE!
T6HS11	T6	B06	0	0	kleiner LLOQ	#VALUE!	1	#VALUE!
T6HS12	T6	B07	0	0	kleiner LLOQ	#VALUE!	1	#VALUE!
T6HS12	T6	B08	0	0	kleiner LLOQ	#VALUE!	1	#VALUE!
T6HS13	T6	B09	0	0	kleiner LLOQ	#VALUE!	1	#VALUE!
T6HS13	T6	B10	0	0	kleiner LLOQ	#VALUE!	1	#VALUE!
T6HS14	T6	B11	0	0	kleiner LLOQ	#VALUE!	1	#VALUE!
T6HS14	T6	B12	0	0	kleiner LLOQ	#VALUE!	1	#VALUE!
0	T6	C01	0	0	kleiner LLOQ	#VALUE!	1	#VALUE!

Plate:	T6

	1	2	3	4	5	6	7	8	9	10	11	12
A												
B												
C					1.181	1.211	1.314	1.355	1.060	1.076		
D												
E												
F												
G							0.051	0.050	0.050	0.052	0.051	0.051
H							0.050	0.050	0.050	0.050	0.049	0.049

Plattenbelegung

	1	2	3	4	5	6	7	8	9	10	11	12
A					T6HN1	T6HN1	T6HN2	T6HN2	T6HN3	T6HN3	T6HN4	T6HN4
B					T6HS11	T6HS11	T6HS12	T6HS12	T6HS13	T6HS13	T6HS14	T6HS14
C					T6HS21	T6HS21	T6HS22	T6HS22	T6HS23	T6HS23	T6HS24	T6HS24
D												
E												

0	T6	C02	0	0	kleiner LLOQ	#VALUE!	1	#VALUE!
0	T6	C03	0	0	kleiner LLOQ	#VALUE!	1	#VALUE!
0	T6	C04	0	0	kleiner LLOQ	#VALUE!	1	#VALUE!
T6HS21	T6	C05	1.181	1.130	1.130	0.076	2.75	0.208
T6HS21	T6	C06	1.211	1.160	1.160	0.080	2.75	0.221
T6HS22	T6	C07	1.314	1.263	1.263	0.098	2.75	0.268
T6HS22	T6	C08	1.355	1.304	1.304	0.104	2.75	0.287
T6HS23	T6	C09	1.060	1.009	1.009	0.055	2.75	0.153
T6HS23	T6	C10	1.076	1.025	1.025	0.058	2.75	0.160
T6HS24	T6	C11	0	0	kleiner LLOQ	#VALUE!	1	#VALUE!
T6HS24	T6	C12	0	0	kleiner LLOQ	#VALUE!	1	#VALUE!
0	T6	D01	0	0	kleiner LLOQ	#VALUE!	1	#VALUE!
0	T6	D02	0	0	kleiner LLOQ	#VALUE!	1	#VALUE!
0	T6	D03	0	0	kleiner LLOQ	#VALUE!	1	#VALUE!
0	T6	D04	0	0	kleiner LLOQ	#VALUE!	1	#VALUE!
0	T6	D05	0	0	kleiner LLOQ	#VALUE!	1	#VALUE!
0	T6	D06	0	0	kleiner LLOQ	#VALUE!	1	#VALUE!
0	T6	D07	0	0	kleiner LLOQ	#VALUE!	1	#VALUE!
0	T6	D08	0	0	kleiner LLOQ	#VALUE!	1	#VALUE!
0	T6	D09	0	0	kleiner LLOQ	#VALUE!	1	#VALUE!
0	T6	D10	0	0	kleiner LLOQ	#VALUE!	1	#VALUE!
0	T6	D11	0	0	kleiner LLOQ	#VALUE!	1	#VALUE!
0	T6	D12	0	0	kleiner LLOQ	#VALUE!	1	#VALUE!
0	T6	E01	0	0	kleiner LLOQ	#VALUE!	1	#VALUE!
0	T6	E02	0	0	kleiner LLOQ	#VALUE!	1	#VALUE!
0	T6	E03	0	0	kleiner LLOQ	#VALUE!	1	#VALUE!
0	T6	E04	0	0	kleiner LLOQ	#VALUE!	1	#VALUE!
0	T6	E05	0	0	kleiner LLOQ	#VALUE!	1	#VALUE!
0	T6	E06	0	0	kleiner LLOQ	#VALUE!	1	#VALUE!
0	T6	E07	0	0	kleiner LLOQ	#VALUE!	1	#VALUE!
0	T6	E08	0	0	kleiner LLOQ	#VALUE!	1	#VALUE!
0	T6	E09	0	0	kleiner LLOQ	#VALUE!	1	#VALUE!
0	T6	E10	0	0	kleiner LLOQ	#VALUE!	1	#VALUE!
0	T6	E11	0	0	kleiner LLOQ	#VALUE!	1	#VALUE!
0	T6	E12	0	0	kleiner LLOQ	#VALUE!	1	#VALUE!
0	T6	F01	0	0	kleiner LLOQ	#VALUE!	1	#VALUE!
0	T6	F02	0	0	kleiner LLOQ	#VALUE!	1	#VALUE!
0	T6	F03	0	0	kleiner LLOQ	#VALUE!	1	#VALUE!
0	T6	F04	0	0	kleiner LLOQ	#VALUE!	1	#VALUE!
0	T6	F05	0	0	kleiner LLOQ	#VALUE!	1	#VALUE!
0	T6	F06	0	0	kleiner LLOQ	#VALUE!	1	#VALUE!
0	T6	F07	0	0	kleiner LLOQ	#VALUE!	1	#VALUE!
0	T6	F08	0	0	kleiner LLOQ	#VALUE!	1	#VALUE!
0	T6	F09	0	0	kleiner LLOQ	#VALUE!	1	#VALUE!
0	T6	F10	0	0	kleiner LLOQ	#VALUE!	1	#VALUE!
0	T6	F11	0	0	kleiner LLOQ	#VALUE!	1	#VALUE!
0	T6	F12	0	0	kleiner LLOQ	#VALUE!	1	#VALUE!
0	T6	G01	0	0	kleiner LLOQ	#VALUE!	1	#VALUE!
0	T6	G02	0	0	kleiner LLOQ	#VALUE!	1	#VALUE!
0	T6	G03	0	0	kleiner LLOQ	#VALUE!	1	#VALUE!
0	T6	G04	0	0	kleiner LLOQ	#VALUE!	1	#VALUE!
0	T6	G05	0	0	kleiner LLOQ	#VALUE!	1	#VALUE!
0	T6	G06	0	0	kleiner LLOQ	#VALUE!	1	#VALUE!
Blank PEG	T6	G07	0	0	kleiner LLOQ	#VALUE!	2.75	#VALUE!
Blank PEG	T6	G08	0	0	kleiner LLOQ	#VALUE!	2.75	#VALUE!
Blank PEG	T6	G09	0	0	kleiner LLOQ	#VALUE!	2.75	#VALUE!
Blank PEG	T6	G10	0	0	kleiner LLOQ	#VALUE!	2.75	#VALUE!
Blank PEG	T6	G11	0	0	kleiner LLOQ	#VALUE!	2.75	#VALUE!
Blank PEG	T6	G12	0	0	kleiner LLOQ	#VALUE!	2.75	#VALUE!
0	T6	H01	0	0	kleiner LLOQ	#VALUE!	1	#VALUE!
0	T6	H02	0	0	kleiner LLOQ	#VALUE!	1	#VALUE!
0	T6	H03	0	0	kleiner LLOQ	#VALUE!	1	#VALUE!
0	T6	H04	0	0	kleiner LLOQ	#VALUE!	1	#VALUE!
0	T6	H05	0	0	kleiner LLOQ	#VALUE!	1	#VALUE!
0	T6	H06	0	0	kleiner LLOQ	#VALUE!	1	#VALUE!
Blank no PEG	T6	H07	0	0	kleiner LLOQ	#VALUE!	2.75	#VALUE!
Blank no PEG	T6	H08	0	0	kleiner LLOQ	#VALUE!	2.75	#VALUE!
Blank no PEG	T6	H09	0	0	kleiner LLOQ	#VALUE!	2.75	#VALUE!
Blank no PEG	T6	H10	0	0	kleiner LLOQ	#VALUE!	2.75	#VALUE!
Blank no PEG	T6	H11	0	0	kleiner LLOQ	#VALUE!	2.75	#VALUE!
Blank no PEG	T6	H12	0	0	kleiner LLOQ	#VALUE!	2.75	#VALUE!

F												
G							Blank PEG	Blank PEG	Blank PEG	Blank PEG	Blank PEG	Blank PEG
H							Blank no PEG	Blank no PEG	Blank no PEG	Blank no PEG	Blank no PEG	Blank no PEG

Comment:

Date Operator

Date Control

0	T7	A01	0	0	kleiner LLOQ	#VALUE!	1	#VALUE!
0	T7	A02	0	0	kleiner LLOQ	#VALUE!	1	#VALUE!
0	T7	A03	0	0	kleiner LLOQ	#VALUE!	1	#VALUE!
0	T7	A04	0	0	kleiner LLOQ	#VALUE!	1	#VALUE!
T7HN1	T7	A05	0	0	kleiner LLOQ	#VALUE!	1	#VALUE!
T7HN1	T7	A06	0	0	kleiner LLOQ	#VALUE!	1	#VALUE!
T7HN2	T7	A07	0	0	kleiner LLOQ	#VALUE!	1	#VALUE!
T7HN2	T7	A08	0	0	kleiner LLOQ	#VALUE!	1	#VALUE!
T7HN3	T7	A09	0	0	kleiner LLOQ	#VALUE!	1	#VALUE!
T7HN3	T7	A10	0	0	kleiner LLOQ	#VALUE!	1	#VALUE!
T7HN4	T7	A11	0	0	kleiner LLOQ	#VALUE!	1	#VALUE!
T7HN4	T7	A12	0	0	kleiner LLOQ	#VALUE!	1	#VALUE!
0	T7	B01	0	0	kleiner LLOQ	#VALUE!	1	#VALUE!
0	T7	B02	0	0	kleiner LLOQ	#VALUE!	1	#VALUE!
0	T7	B03	0	0	kleiner LLOQ	#VALUE!	1	#VALUE!
0	T7	B04	0	0	kleiner LLOQ	#VALUE!	1	#VALUE!
T7HS11	T7	B05	0	0	kleiner LLOQ	#VALUE!	1	#VALUE!
T7HS11	T7	B06	0	0	kleiner LLOQ	#VALUE!	1	#VALUE!
T7HS12	T7	B07	0	0	kleiner LLOQ	#VALUE!	1	#VALUE!
T7HS12	T7	B08	0	0	kleiner LLOQ	#VALUE!	1	#VALUE!
T7HS13	T7	B09	0	0	kleiner LLOQ	#VALUE!	1	#VALUE!
T7HS13	T7	B10	0	0	kleiner LLOQ	#VALUE!	1	#VALUE!
T7HS14	T7	B11	0	0	kleiner LLOQ	#VALUE!	1	#VALUE!
T7HS14	T7	B12	0	0	kleiner LLOQ	#VALUE!	1	#VALUE!
0	T7	C01	0	0	kleiner LLOQ	#VALUE!	1	#VALUE!
0	T7	C02	0	0	kleiner LLOQ	#VALUE!	1	#VALUE!
0	T7	C03	0	0	kleiner LLOQ	#VALUE!	1	#VALUE!
0	T7	C04	0	0	kleiner LLOQ	#VALUE!	1	#VALUE!
T7HS21	T7	C05	0	0	kleiner LLOQ	#VALUE!	1	#VALUE!
T7HS21	T7	C06	0	0	kleiner LLOQ	#VALUE!	1	#VALUE!
T7HS22	T7	C07	0	0	kleiner LLOQ	#VALUE!	1	#VALUE!
T7HS22	T7	C08	0	0	kleiner LLOQ	#VALUE!	1	#VALUE!
T7HS23	T7	C09	1.123	1.072	1.072	0.066	2.75	0.182
T7HS23	T7	C10	1.101	1.050	1.050	0.062	2.75	0.171
T7HS24	T7	C11	0	0	kleiner LLOQ	#VALUE!	1	#VALUE!
T7HS24	T7	C12	0	0	kleiner LLOQ	#VALUE!	1	#VALUE!
0	T7	D01	0	0	kleiner LLOQ	#VALUE!	1	#VALUE!
0	T7	D02	0	0	kleiner LLOQ	#VALUE!	1	#VALUE!
0	T7	D03	0	0	kleiner LLOQ	#VALUE!	1	#VALUE!
0	T7	D04	0	0	kleiner LLOQ	#VALUE!	1	#VALUE!
0	T7	D05	0	0	kleiner LLOQ	#VALUE!	1	#VALUE!
0	T7	D06	0	0	kleiner LLOQ	#VALUE!	1	#VALUE!
0	T7	D07	0	0	kleiner LLOQ	#VALUE!	1	#VALUE!
0	T7	D08	0	0	kleiner LLOQ	#VALUE!	1	#VALUE!
0	T7	D09	0	0	kleiner LLOQ	#VALUE!	1	#VALUE!
0	T7	D10	0	0	kleiner LLOQ	#VALUE!	1	#VALUE!
0	T7	D11	0	0	kleiner LLOQ	#VALUE!	1	#VALUE!
0	T7	D12	0	0	kleiner LLOQ	#VALUE!	1	#VALUE!
0	T7	E01	0	0	kleiner LLOQ	#VALUE!	1	#VALUE!
0	T7	E02	0	0	kleiner LLOQ	#VALUE!	1	#VALUE!
0	T7	E03	0	0	kleiner LLOQ	#VALUE!	1	#VALUE!
0	T7	E04	0	0	kleiner LLOQ	#VALUE!	1	#VALUE!
0	T7	E05	0	0	kleiner LLOQ	#VALUE!	1	#VALUE!
0	T7	E06	0	0	kleiner LLOQ	#VALUE!	1	#VALUE!
0	T7	E07	0	0	kleiner LLOQ	#VALUE!	1	#VALUE!
0	T7	E08	0	0	kleiner LLOQ	#VALUE!	1	#VALUE!
0	T7	E09	0	0	kleiner LLOQ	#VALUE!	1	#VALUE!

Plate: T7

	1	2	3	4	5	6	7	8	9	10	11	12
A												
B												
C									1.123	1.101		
D												
E												
F												
G							0.051	0.051	0.051	0.053	0.052	0.051
H							0.050	0.050	0.050	0.050	0.050	0.050

Plattenbelegung

	1	2	3	4	5	6	7	8	9	10	11	12
A					T7HN1	T7HN1	T7HN2	T7HN2	T7HN3	T7HN3	T7HN4	T7HN4
B					T7HS11	T7HS11	T7HS12	T7HS12	T7HS13	T7HS13	T7HS14	T7HS14
C					T7HS21	T7HS21	T7HS22	T7HS22	T7HS23	T7HS23	T7HS24	T7HS24
D												
E												
F												
G							Blank PEG	Blank PEG	Blank PEG	Blank PEG	Blank PEG	Blank PEG
H							Blank no PEG	Blank no PEG	Blank no PEG	Blank no PEG	Blank no PEG	Blank no PEG

Comment:

Date Operator

Date Control

0	T7	E10	0	0	kleiner LLOQ	#VALUE!	1	#VALUE!
0	T7	E11	0	0	kleiner LLOQ	#VALUE!	1	#VALUE!
0	T7	E12	0	0	kleiner LLOQ	#VALUE!	1	#VALUE!
0	T7	F01	0	0	kleiner LLOQ	#VALUE!	1	#VALUE!
0	T7	F02	0	0	kleiner LLOQ	#VALUE!	1	#VALUE!
0	T7	F03	0	0	kleiner LLOQ	#VALUE!	1	#VALUE!
0	T7	F04	0	0	kleiner LLOQ	#VALUE!	1	#VALUE!
0	T7	F05	0	0	kleiner LLOQ	#VALUE!	1	#VALUE!
0	T7	F06	0	0	kleiner LLOQ	#VALUE!	1	#VALUE!
0	T7	F07	0	0	kleiner LLOQ	#VALUE!	1	#VALUE!
0	T7	F08	0	0	kleiner LLOQ	#VALUE!	1	#VALUE!
0	T7	F09	0	0	kleiner LLOQ	#VALUE!	1	#VALUE!
0	T7	F10	0	0	kleiner LLOQ	#VALUE!	1	#VALUE!
0	T7	F11	0	0	kleiner LLOQ	#VALUE!	1	#VALUE!
0	T7	F12	0	0	kleiner LLOQ	#VALUE!	1	#VALUE!
0	T7	G01	0	0	kleiner LLOQ	#VALUE!	1	#VALUE!
0	T7	G02	0	0	kleiner LLOQ	#VALUE!	1	#VALUE!
0	T7	G03	0	0	kleiner LLOQ	#VALUE!	1	#VALUE!
0	T7	G04	0	0	kleiner LLOQ	#VALUE!	1	#VALUE!
0	T7	G05	0	0	kleiner LLOQ	#VALUE!	1	#VALUE!
0	T7	G06	0	0	kleiner LLOQ	#VALUE!	1	#VALUE!
Blank PEG	T7	G07	0	0	kleiner LLOQ	#VALUE!	2.75	#VALUE!
Blank PEG	T7	G08	0	0	kleiner LLOQ	#VALUE!	2.75	#VALUE!
Blank PEG	T7	G09	0	0	kleiner LLOQ	#VALUE!	2.75	#VALUE!
Blank PEG	T7	G10	0	0	kleiner LLOQ	#VALUE!	2.75	#VALUE!
Blank PEG	T7	G11	0	0	kleiner LLOQ	#VALUE!	2.75	#VALUE!
Blank PEG	T7	G12	0	0	kleiner LLOQ	#VALUE!	2.75	#VALUE!
0	T7	H01	0	0	kleiner LLOQ	#VALUE!	1	#VALUE!
0	T7	H02	0	0	kleiner LLOQ	#VALUE!	1	#VALUE!
0	T7	H03	0	0	kleiner LLOQ	#VALUE!	1	#VALUE!
0	T7	H04	0	0	kleiner LLOQ	#VALUE!	1	#VALUE!
0	T7	H05	0	0	kleiner LLOQ	#VALUE!	1	#VALUE!
0	T7	H06	0	0	kleiner LLOQ	#VALUE!	1	#VALUE!
Blank no PEG	T7	H07	0	0	kleiner LLOQ	#VALUE!	2.75	#VALUE!
Blank no PEG	T7	H08	0	0	kleiner LLOQ	#VALUE!	2.75	#VALUE!
Blank no PEG	T7	H09	0	0	kleiner LLOQ	#VALUE!	2.75	#VALUE!
Blank no PEG	T7	H10	0	0	kleiner LLOQ	#VALUE!	2.75	#VALUE!
Blank no PEG	T7	H11	0	0	kleiner LLOQ	#VALUE!	2.75	#VALUE!
Blank no PEG	T7	H12	0	0	kleiner LLOQ	#VALUE!	2.75	#VALUE!

1.447	1.739		1.170						0.667	0.440	0.043	1
1.447	1.332		1.392	0.137	0.146	0.130	0.138	0.109	0.729	0.478	0.042	
1.391	1.281		1.311	0.204	0.184	0.184	0.198	0.201	0.743	0.474	0.043	
0.961	0.835		1.405	0.336	0.347	0.375	0.387	0.300	0.824	0.483	0.044	
0.445	0.441		1.280	1.085	1.086	1.218	1.004	0.873	0.704	0.457	0.041	
0.355	0.315		1.332	1.768	1.925	1.753	1.739	1.606			0.036	
0.223	0.188	1.666	1.499	1.632		0.048	0.046	0.047	0.048	0.048	0.048	
0.118	0.106	1.556	1.622	1.573		0.047	0.054	0.051	0.051	0.052	0.051	

1.652	1.627		1.188	0.748	0.811	0.764	0.753	0.674	0.751	0.529	0.044	2
1.443	1.372		1.364	0.139	0.147	0.131	0.139	0.110	0.811	0.561	0.043	
1.377	1.281		1.284	0.206	0.189	0.184	0.200	0.202	0.821	0.560	0.044	
0.972	0.883		1.387	0.339	0.349	0.386	0.406	0.312	0.903	0.565	0.044	
0.484	0.474		1.298	1.087	1.113	1.203	1.022	0.903	0.790	0.544	0.042	
0.359	0.326		1.308	1.622	1.622	1.630	1.625	1.550			0.037	
0.228	0.199	1.527	1.373	1.462		0.048	0.046	0.048	0.049	0.049	0.049	
0.125	0.109	1.473	1.410	1.371		0.048	0.054	0.052	0.052	0.052	0.051	

1.579	1.475		1.150	1.210	1.303	1.202	1.219	1.125	0.759	0.549	0.043	3
1.357	1.303		1.283	0.140	0.148	0.131	0.139	0.111	0.820	0.575	0.043	
1.323	1.228		1.227	0.206	0.188	0.184	0.198	0.201	0.824	0.579	0.043	
0.959	0.872		1.321	0.339	0.348	0.387	0.406	0.314	0.909	0.580	0.044	
0.483	0.473		1.270	1.069	1.103	1.181	1.017	0.903	0.798	0.560	0.042	
0.360	0.326		1.270	1.497	1.448	1.496	1.494	1.449			0.037	
0.228	0.199	1.374	1.262	1.314		0.048	0.046	0.048	0.049	0.049	0.049	
0.125	0.110	1.346	1.262	1.253		0.048	0.054	0.052	0.052	0.052	0.051	

1.511	1.369			1.538	1.712	1.528	1.502	1.425			0.043	4
1.296	1.261										0.043	
1.272	1.193										0.043	
0.947	0.859										0.044	
0.481	0.471										0.042	
0.361	0.325										0.037	
0.229	0.199					0.048	0.046	0.048	0.049	0.049	0.049	
0.125	0.110					0.047	0.054	0.052	0.052	0.052	0.051	

1.493	1.317	1.678	1.110	1.621	1.807	1.618	1.584	1.508	0.765	0.597	0.043	5
1.259	1.238	1.850	1.197	0.139	0.150	0.132	0.139	0.111	0.821	0.588	0.043	
1.248	1.175	1.816	1.153	0.204	0.188	0.184	0.197	0.199	0.831	0.599	0.044	
0.943	0.859	1.970	1.231	0.338	0.357	0.386	0.406	0.316	0.916	0.595	0.044	
0.480	0.470	2.220	1.220	1.054	1.088	1.154	1.006	0.902	0.805	0.579	0.042	
0.359	0.325	2.149	1.211	1.395	1.324	1.348	1.345	1.317	0.037	0.037	0.037	
0.228	0.199	1.240	1.151	1.183	0.034	0.049	0.046	0.048	0.049	0.049	0.049	
0.125	0.110	1.223	1.132	1.146	0.038	0.048	0.054	0.052	0.052	0.052	0.051	

1.434	1.258		1.090	1.648	1.876	1.649	1.627	1.535				6
1.217	1.201		1.149									
1.205	1.143		1.109									
0.935	0.845		1.182									
0.477	0.469		1.181									
0.360	0.323		1.167									
0.229	0.200					0.049	0.047	0.048	0.049	0.049	0.049	
0.125	0.110					0.048	0.054	0.052	0.052	0.052	0.051	

1.013	0.810		0.668	0.808	0.940	0.720	0.798	0.687	0.904	0.845	0.043	7
0.709	0.827		0.703	0.142	0.156	0.136	0.137	0.116	0.990	0.846	0.042	
0.682	0.692		0.680	0.197	0.188	0.190	0.191	0.184	1.023	0.861	0.043	
0.507	0.519		0.740	0.316	0.264	0.364	0.377	0.312	1.111	0.870	0.043	
0.394	0.406		0.682	0.580	0.561	0.600	0.529	0.563	0.962	0.834	0.042	
0.333	0.322		0.661	0.995	0.930	0.848	0.832	0.797	0.037	0.037	0.037	
0.229	0.201	0.843	0.679	0.732		0.048	0.046	0.047			0.047	
0.124	0.111	0.773	0.682	0.701		0.047	0.053	0.051	0.051	0.052	0.051	

skin cultivation

1.107	2.478	2.285	0.495									T4 1 hum sui
2.599	2.221	2.620	0.160									
1.529	1.413	1.337	0.179									
	1.715	1.096	0.716									
2.420	1.332	1.841	1.177									
2.159	1.669	1.286	1.300									
						0.051	0.053	0.051	0.052	0.051	0.050	
						0.050	0.050	0.053	0.050	0.050	0.050	

				1.128	1.099	1.007	0.969	0.915	0.921	0.436	0.431	T4 2 hum sui
				1.143	0.846	0.590	0.597	0.077	0.074	0.169	0.174	
				0.558	0.564	0.603	0.587	0.591	0.594	0.198	0.201	
				0.037	0.037	0.427	0.827	1.653	1.703	0.745	0.741	
				0.307	0.302	0.444	0.439	0.274	0.233	1.258	1.343	
				0.286	0.293	0.642	0.631	0.377	0.392	0.431	0.437	
						0.052	0.054	0.052	0.052	0.052	0.051	
						0.050	0.050	0.053	0.050	0.051	0.050	

												T4 3 hum sui
0.633	0.717	0.331	0.350	0.298	0.250	0.154	0.043	0.054	0.056	0.052	0.053	
						0.036	0.044	0.040	0.040	0.042	0.051	

2.884	2.987	2.990	0.834									T5 1 hum sui
0.339	0.285	0.380	0.181									
1.303	1.242	1.088	0.213									
0.035	1.234	0.923	0.438									
0.488	0.743	0.416	0.596									
0.745	0.507	0.617	0.808									
						0.051	0.051	0.053	0.051	0.052	0.051	
						0.056	0.053	0.051	0.049	0.053	0.052	

				0.749	0.763	1.096	1.121	0.981	0.973	0.735	0.717	T5 2 hum sui
				0.323	0.315	0.263	0.273	0.403	0.405	0.192	0.198	
				0.636	0.661	0.715	0.706	1.430	1.378	0.232	0.230	
						0.790	0.621	1.131	1.140	0.500	0.517	
				0.528	0.542	0.908	0.926	0.448	0.444	0.768	0.780	
				0.841	0.840	0.606	0.597	0.712	0.741	0.916	0.915	
						0.053	0.053	0.054	0.053	0.053	0.052	
						0.101	0.056	0.052	0.050	0.054	0.053	

2.565	2.187	2.054	0.598									T6 1 hum sui
0.238	0.241	0.246	0.191									
0.974	1.028	0.879	0.207									
0.82984877	0.556	0.366										
0.311	0.314	0.220	0.248									
0.234	0.277	0.377	0.512									
						0.050	0.049	0.050	0.051	0.050	0.050	
						0.050	0.049	0.050	0.049	0.049	0.049	

				0.873	0.588	0.744	0.778	0.518	0.513	0.634	0.620	T6 2 hum sui
				0.274	0.273	0.262	0.271	0.261	0.272	0.190	0.194	
				1.181	1.211	1.314	1.355	1.060	1.076	0.232	0.227	
						1.027	1.0255289	0.607	0.625	0.409	0.413	
				0.341	0.356	0.341	0.349	0.231	0.239	0.257	0.254	
				0.251	0.260	0.283	0.286	0.421	0.436	0.559	0.563	
						0.051	0.050	0.050	0.052	0.051	0.051	
						0.050	0.050	0.050	0.050	0.049	0.049	

1.641	1.926	0.976	0.329									T7 1 hum sui
0.225	0.191	0.208	0.124									
1.266	1.460	1.113	0.215									
	0.480	0.706	0.480									
0.362	0.307	0.162	0.225									
0.257	0.293	0.346	0.425			0.050	0.049	0.050	0.052	0.051	0.049	
						0.049	0.049	0.049	0.049	0.049	0.049	

				0.292	0.301	0.404	0.394	0.647	0.835	0.282	0.295	T7 2 hum sui
				0.199	0.200	0.188	0.186	0.204	0.205	0.121	0.122	
				0.522	0.505	0.598	0.588	1.123	1.101	0.219	0.236	
						0.494	0.470	0.771	0.761	0.487	0.504	
				0.355	0.359	0.315	0.311	0.190	0.179	0.241	0.243	
				0.246	0.258	0.225	0.260	0.351	0.350	0.431	0.444	
						0.051	0.051	0.051	0.053	0.052	0.051	
						0.050	0.050	0.050	0.050	0.050	0.050	

Project number		F-120	Apparatus	Wallac Victor	Operator	IsBa
GLP Study (Number)		n.a.	Protocol (Instrument method)	LDH test 2016	Date of preparation	19-04-18
hot substance	isotope	n.a.	File name (results)	IsBa_180419/20_LDH_full_va	Date of measurement	19-04-18
	name	n.a.	Kind of well plate	96 well	shaking time [min]	30
	ACB-ID	n.a.	sample volume [µL]	100	stirring rate (Target) [rpm]	150
	Batch number	n.a.	Cocktail volume [µL]	175	Kind of measurement	UV-vis
cold substance	name	LDH	ACB-ID of cocktail		Wave length [nm]	450
	ACB-ID	L23982R	Matrix	DMEM (from powder)+PEG	Remarks	Cocktail 100µl RM, 75µl STOP
	Batch number	SLBK6345V	Blank description	DMEM/PEG, H2O	Remarks	7 standards split low/high
n.a.		n.a.	Pipettes (No. / volume)	50-200µl	Remarks	KLP4 common for both
n.a.		n.a.	Pipettes (No. / volume)	n.a.	Remarks	n.a.

Messdaten (diese Tabelle in Bericht übernehmen)

Sample name *	concentration (theor.) *	measured data	measured data	measured data	mean measured	SD	RSD	Blank *	measured data after * Blank subtraction	concentration (calc.) *	Deviation *	Residuen
	[µg/mL]	[AU]	[AU]	[AU]	[AU]	[AU]	[%]	[AU]		[µg/mL]	[%]	
KLP1								0.047				
KLP2								0.054				
KLP3								0.051				
KLP4	0.041	0.959	0.972	0.883	0.938	0.04	4.19	0.051	0.887	0.041	-0.57	0.00
KLP5	0.018	0.483	0.484	0.474	0.480	0.00	0.98	0.052	0.429	0.019	2.42	0.00
KLP6	0.012	0.360	0.359	0.326	0.348	0.02	4.54	0.051	0.297	0.013	5.00	0.00
KLP7	0.007	0.228	0.228	0.199	0.218	0.014	6.35		0.168	0.006	-11.29	0.00
KLP8												

Statistical data

Geradensteigung	Slope	m	21.03
Y-Achsenabschnitt	Y-Intercept	b	0.03
Standardabw. Geradensteigung	SD-Slope	s_m	0.648055501
Standardabw. Achsenabschnittes	SD-Y-Intercept	s_b	0.015177452
Anzahl Messpunkte	number of measuring points	n	4
Quadratsumme	sum of squares	Qxx	0.000664048
Bereichsmitte			0.019557193
Freiheitsgrade	degree of freedom	f	2
Student-t-Faktor für (P = 95 %; f = n-2)	Student-t-factor for (P = 95 %; f = n-2)	t	4.303
Vertrauensbereich Steig. (95 %) Obergrenze		$m + VB_m$	23.81763508
Vertrauensbereich Steig. (95 %) Untergrenze		$m - VB_m$	18.24046944
Vertrauensbereich Achsenabschnitt (95 %) Og.		$b + Vb_b$	0.099335829
Vertrauensbereich Achsenabschnitt (95 %) Ug.		$b - Vb_b$	-0.031281327
Korrelationskoeffizient	correlation coefficient	r	0.9991
Bestimmtheitsmaß	determiantion coefficient	r^2	0.9981
Reststandardabweichung		s_0	0.01669983
Summe Restquadrate		sd	0.657385838
Verfahrensstandardabw.		s_{x0}	0.000794131
Rel. Verfahrensstandardabw. %		V_{x0}	4.060559029

Berichten

mean Blank	0		
SD Blank	0.00		
RSD Blank	3.79	x=	
x*SD (LLOQ)	0.01		5
x*SD (LOD)	0.01		3
**LLOQ (AU)**	**0.061**		
**LOD (AU)**	**0.057**		
ULOQ	0.887		
LLOQ (Lin)	0.168		

Evaluation / Comment

LDH linearity valid with 7 standards, split in lin high and lin low, each with 4 standards and KLP4 common standard for both. R² 0,9981, deviations for both between -13.34% and +13.04%

Date | Operator | Date | Control

Figure 1 Linearity

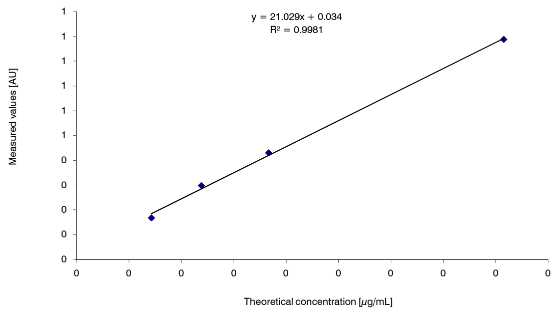

Figure 2 Method validation Residuen Plot

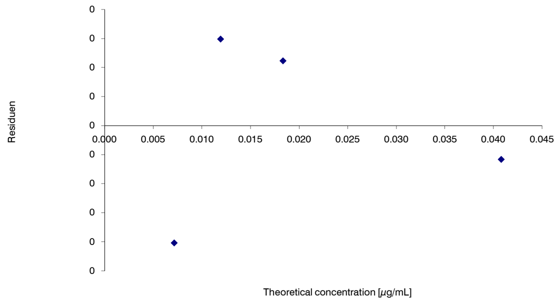

Evaluation / Comment

lin passed

Date	Operator	Date	Control

Sample name	Plate	Position	[AU]	[AU]-Blank	Rating	[µg/mL]	Dilution factor	[µg/mL]
0	T8	A01	0	0	kleiner LLOQ	#VALUE!	1	#VALUE!
0	T8	A02	0	0	kleiner LLOQ	#VALUE!	1	#VALUE!
0	T8	A03	0	0	kleiner LLOQ	#VALUE!	1	#VALUE!
0	T8	A04	0	0	kleiner LLOQ	#VALUE!	1	#VALUE!
T8HN1	T8	A05	0.635	0.585	0.585	0.026	2.75	0.072
T8HN1	T8	A06	0.607	0.556	0.556	0.025	2.75	0.068
T8HN2	T8	A07	0.628	0.577	0.577	0.026	2.75	0.071
T8HN2	T8	A08	0.661	0.610	0.610	0.027	2.75	0.075
T8HN3	T8	A09	0.404	0.353	0.353	0.015	2.75	0.042
T8HN3	T8	A10	0.309	0.258	0.258	0.011	2.75	0.029
T8HN4	T8	A11	0.229	0.178	0.178	0.007	2.75	0.019
T8HN4	T8	A12	0.222	0.171	0.171	0.007	2.75	0.018
0	T8	B01	0	0	kleiner LLOQ	#VALUE!	1	#VALUE!
0	T8	B02	0	0	kleiner LLOQ	#VALUE!	1	#VALUE!
0	T8	B03	0	0	kleiner LLOQ	#VALUE!	1	#VALUE!
0	T8	B04	0	0	kleiner LLOQ	#VALUE!	1	#VALUE!
T8HS11	T8	B05	0.460	0.409	0.409	0.018	2.75	0.0491
T8HS11	T8	B06	0	0	kleiner LLOQ	#VALUE!	2.75	#VALUE!
T8HS12	T8	B07	0	0	kleiner LLOQ	#VALUE!	2.75	#VALUE!
T8HS12	T8	B08	0	0	kleiner LLOQ	#VALUE!	2.75	#VALUE!
T8HS13	T8	B09	0.129	0.078	kleiner LLOQ	#VALUE!	2.75	#VALUE!
T8HS13	T8	B10	0.136	0.085	kleiner LLOQ	#VALUE!	2.75	#VALUE!
T8HS14	T8	B11	0.081	0.030	kleiner LLOQ	#VALUE!	2.75	#VALUE!
T8HS14	T8	B12	0.084	0.033	kleiner LLOQ	#VALUE!	2.75	#VALUE!
0	T8	C01	0	0	kleiner LLOQ	#VALUE!	1	#VALUE!
0	T8	C02	0	0	kleiner LLOQ	#VALUE!	1	#VALUE!
0	T8	C03	0	0	kleiner LLOQ	#VALUE!	1	#VALUE!
0	T8	C04	0	0	kleiner LLOQ	#VALUE!	1	#VALUE!
T8HS21	T8	C05	0.458	0.407	0.407	0.018	2.75	0.049
T8HS21	T8	C06	0.708	0.657	0.657	0.030	2.75	0.081
T8HS22	T8	C07	0.553	0.502	0.502	0.022	2.75	0.061
T8HS22	T8	C08	0.584	0.533	0.533	0.024	2.75	0.065
T8HS23	T8	C09	0.670	0.619	0.619	0.028	2.75	0.076
T8HS23	T8	C10	0.659	0.608	0.608	0.027	2.75	0.075
T8HS24	T8	C11	0.222	0.171	0.171	0.007	2.75	0.018
T8HS24	T8	C12	0.224	0.173	0.173	0.007	2.75	0.018
0	T8	D01	0	0	kleiner LLOQ	#VALUE!	1	#VALUE!
0	T8	D02	0	0	kleiner LLOQ	#VALUE!	1	#VALUE!
0	T8	D03	0	0	kleiner LLOQ	#VALUE!	1	#VALUE!
0	T8	D04	0	0	kleiner LLOQ	#VALUE!	1	#VALUE!
0	T8	D05	0	0	kleiner LLOQ	#VALUE!	1	#VALUE!
0	T8	D06	0	0	kleiner LLOQ	#VALUE!	1	#VALUE!
0	T8	D07	0	0	kleiner LLOQ	#VALUE!	1	#VALUE!
0	T8	D08	0	0	kleiner LLOQ	#VALUE!	1	#VALUE!
0	T8	D09	0	0	kleiner LLOQ	#VALUE!	1	#VALUE!
0	T8	D10	0	0	kleiner LLOQ	#VALUE!	1	#VALUE!
0	T8	D11	0	0	kleiner LLOQ	#VALUE!	1	#VALUE!
0	T8	D12	0	0	kleiner LLOQ	#VALUE!	1	#VALUE!

Plate: T8

	Test	Test	Test	Test								
	1	2	3	4	5	6	7	8	9	10	11	12
A					0.635	0.607	0.628	0.661	0.404	0.309	0.229	0.222
B					0.460	0.171	0.156	0.167	0.129	0.136	0.081	0.084
C					0.458	0.708	0.553	0.584	0.670	0.659	0.222	0.224
D												
E												
F												
G							0.053	0.055	0.053	0.054	0.053	0.053
H							0.053	0.052	0.051	0.052	0.052	0.052

Plattenbelegung

	1	2	3	4	5	6	7	8	9	10	11	12
A					T8HN1	T8HN1	T8HN2	T8HN2	T8HN3	T8HN3	T8HN4	T8HN4
B					T8HS11	T8HS11	T8HS12	T8HS12	T8HS13	T8HS13	T8HS14	T8HS14
C					T8HS21	T8HS21	T8HS22	T8HS22	T8HS23	T8HS23	T8HS24	T8HS24
D												
E												
F												
G							Blank PEG	Blank PEG	Blank PEG	Blank PEG	Blank PEG	Blank PEG
H							Blank no PEG	Blank no PEG	Blank no PEG	Blank no PEG	Blank no PEG	Blank no PEG

Samples aufgeteilt in lin high und low:höher konzentrierte Samples bei lin high zu finden.

Comment:

Date Operator

Date Control

0	T8	E01	0	0	kleiner LLOQ	#VALUE!	1	#VALUE!
0	T8	E02	0	0	kleiner LLOQ	#VALUE!	1	#VALUE!
0	T8	E03	0	0	kleiner LLOQ	#VALUE!	1	#VALUE!
0	T8	E04	0	0	kleiner LLOQ	#VALUE!	1	#VALUE!
0	T8	E05	0	0	kleiner LLOQ	#VALUE!	1	#VALUE!
0	T8	E06	0	0	kleiner LLOQ	#VALUE!	1	#VALUE!
0	T8	E07	0	0	kleiner LLOQ	#VALUE!	1	#VALUE!
0	T8	E08	0	0	kleiner LLOQ	#VALUE!	1	#VALUE!
0	T8	E09	0	0	kleiner LLOQ	#VALUE!	1	#VALUE!
0	T8	E10	0	0	kleiner LLOQ	#VALUE!	1	#VALUE!
0	T8	E11	0	0	kleiner LLOQ	#VALUE!	1	#VALUE!
0	T8	E12	0	0	kleiner LLOQ	#VALUE!	1	#VALUE!
0	T8	F01	0	0	kleiner LLOQ	#VALUE!	1	#VALUE!
0	T8	F02	0	0	kleiner LLOQ	#VALUE!	1	#VALUE!
0	T8	F03	0	0	kleiner LLOQ	#VALUE!	1	#VALUE!
0	T8	F04	0	0	kleiner LLOQ	#VALUE!	1	#VALUE!
0	T8	F05	0	0	kleiner LLOQ	#VALUE!	1	#VALUE!
0	T8	F06	0	0	kleiner LLOQ	#VALUE!	1	#VALUE!
0	T8	F07	0	0	kleiner LLOQ	#VALUE!	1	#VALUE!
0	T8	F08	0	0	kleiner LLOQ	#VALUE!	1	#VALUE!
0	T8	F09	0	0	kleiner LLOQ	#VALUE!	1	#VALUE!
0	T8	F10	0	0	kleiner LLOQ	#VALUE!	1	#VALUE!
0	T8	F11	0	0	kleiner LLOQ	#VALUE!	1	#VALUE!
0	T8	F12	0	0	kleiner LLOQ	#VALUE!	1	#VALUE!
0	T8	G01	0	0	kleiner LLOQ	#VALUE!	1	#VALUE!
0	T8	G02	0	0	kleiner LLOQ	#VALUE!	1	#VALUE!
0	T8	G03	0	0	kleiner LLOQ	#VALUE!	1	#VALUE!
0	T8	G04	0	0	kleiner LLOQ	#VALUE!	1	#VALUE!
0	T8	G05	0	0	kleiner LLOQ	#VALUE!	1	#VALUE!
0	T8	G06	0	0	kleiner LLOQ	#VALUE!	1	#VALUE!
Blank PEG	T8	G07	0	0	kleiner LLOQ	#VALUE!	2.75	#VALUE!
Blank PEG	T8	G08	0	0	kleiner LLOQ	#VALUE!	2.75	#VALUE!
Blank PEG	T8	G09	0	0	kleiner LLOQ	#VALUE!	2.75	#VALUE!
Blank PEG	T8	G10	0	0	kleiner LLOQ	#VALUE!	2.75	#VALUE!
Blank PEG	T8	G11	0	0	kleiner LLOQ	#VALUE!	2.75	#VALUE!
Blank PEG	T8	G12	0	0	kleiner LLOQ	#VALUE!	2.75	#VALUE!
0	T8	H01	0	0	kleiner LLOQ	#VALUE!	1	#VALUE!
0	T8	H02	0	0	kleiner LLOQ	#VALUE!	1	#VALUE!
0	T8	H03	0	0	kleiner LLOQ	#VALUE!	1	#VALUE!
0	T8	H04	0	0	kleiner LLOQ	#VALUE!	1	#VALUE!
0	T8	H05	0	0	kleiner LLOQ	#VALUE!	1	#VALUE!
0	T8	H06	0	0	kleiner LLOQ	#VALUE!	1	#VALUE!
Blank no PEG	T8	H07	0	0	kleiner LLOQ	#VALUE!	2.75	#VALUE!
Blank no PEG	T8	H08	0	0	kleiner LLOQ	#VALUE!	2.75	#VALUE!
Blank no PEG	T8	H09	0	0	kleiner LLOQ	#VALUE!	2.75	#VALUE!
Blank no PEG	T8	H10	0	0	kleiner LLOQ	#VALUE!	2.75	#VALUE!
Blank no PEG	T8	H11	0	0	kleiner LLOQ	#VALUE!	2.75	#VALUE!
Blank no PEG	T8	H12	0	0	kleiner LLOQ	#VALUE!	2.75	#VALUE!

0	T9	A01	0	0	kleiner LLOQ	#VALUE!	1	#VALUE!
0	T9	A02	0	0	kleiner LLOQ	#VALUE!	1	#VALUE!
0	T9	A03	0	0	kleiner LLOQ	#VALUE!	1	#VALUE!
0	T9	A04	0	0	kleiner LLOQ	#VALUE!	1	#VALUE!
T9HN1	T9	A05	0.379	0.328	0.328	0.014	2.75	0.038
T9HN1	T9	A06	0.370	0.319	0.319	0.014	2.75	0.037
T9HN2	T9	A07	0.484	0.433	0.433	0.019	2.75	0.052
T9HN2	T9	A08	0.478	0.427	0.427	0.019	2.75	0.051
T9HN3	T9	A09	0.220	0.169	0.169	0.006	2.75	0.018
T9HN3	T9	A10	0.291	0.240	0.240	0.010	2.75	0.027
T9HN4	T9	A11	0.404	0.353	0.353	0.015	2.75	0.042
T9HN4	T9	A12	0.398	0.347	0.347	0.015	2.75	0.041
0	T9	B01	0	0	kleiner LLOQ	#VALUE!	1	#VALUE!
0	T9	B02	0	0	kleiner LLOQ	#VALUE!	1	#VALUE!
0	T9	B03	0	0	kleiner LLOQ	#VALUE!	1	#VALUE!
0	T9	B04	0	0	kleiner LLOQ	#VALUE!	1	#VALUE!
T9HS11	T9	B05	0.157	0.106	kleiner LLOQ	#VALUE!	2.75	#VALUE!
T9HS11	T9	B06	0.155	0.104	kleiner LLOQ	#VALUE!	2.75	#VALUE!
T9HS12	T9	B07	0.155	0.104	kleiner LLOQ	#VALUE!	2.75	#VALUE!
T9HS12	T9	B08	0.157	0.106	kleiner LLOQ	#VALUE!	2.75	#VALUE!
T9HS13	T9	B09	0.194	0.143	kleiner LLOQ	#VALUE!	2.75	#VALUE!
T9HS13	T9	B10	0.200	0.149	kleiner LLOQ	#VALUE!	2.75	#VALUE!
T9HS14	T9	B11	0.080	0.030	kleiner LLOQ	#VALUE!	2.75	#VALUE!
T9HS14	T9	B12	0.091	0.040	kleiner LLOQ	#VALUE!	2.75	#VALUE!
0	T9	C01	0	0	kleiner LLOQ	#VALUE!	1	#VALUE!
0	T9	C02	0	0	kleiner LLOQ	#VALUE!	1	#VALUE!
0	T9	C03	0	0	kleiner LLOQ	#VALUE!	1	#VALUE!
0	T9	C04	0	0	kleiner LLOQ	#VALUE!	1	#VALUE!
T9HS21	T9	C05	0.623	0.573	0.573	0.026	2.75	0.070
T9HS21	T9	C06	0.633	0.582	0.582	0.026	2.75	0.072
T9HS22	T9	C07	0.542	0.491	0.491	0.022	2.75	0.060
T9HS22	T9	C08	0.548	0.497	0.497	0.022	2.75	0.061
T9HS23	T9	C09	0.625	0.574	0.574	0.026	2.75	0.071
T9HS23	T9	C10	0.640	0.589	0.589	0.026	2.75	0.073
T9HS24	T9	C11	0.197	0.146	kleiner LLOQ	#VALUE!	2.75	#VALUE!
T9HS24	T9	C12	0.192	0.141	kleiner LLOQ	#VALUE!	2.75	#VALUE!
0	T9	D01	0	0	kleiner LLOQ	#VALUE!	1	#VALUE!
0	T9	D02	0	0	kleiner LLOQ	#VALUE!	1	#VALUE!
0	T9	D03	0	0	kleiner LLOQ	#VALUE!	1	#VALUE!
0	T9	D04	0	0	kleiner LLOQ	#VALUE!	1	#VALUE!
0	T9	D05	0	0	kleiner LLOQ	#VALUE!	1	#VALUE!
0	T9	D06	0	0	kleiner LLOQ	#VALUE!	1	#VALUE!
0	T9	D07	0	0	kleiner LLOQ	#VALUE!	1	#VALUE!
0	T9	D08	0	0	kleiner LLOQ	#VALUE!	1	#VALUE!
0	T9	D09	0	0	kleiner LLOQ	#VALUE!	1	#VALUE!
0	T9	D10	0	0	kleiner LLOQ	#VALUE!	1	#VALUE!
0	T9	D11	0	0	kleiner LLOQ	#VALUE!	1	#VALUE!
0	T9	D12	0	0	kleiner LLOQ	#VALUE!	1	#VALUE!

Plate: T9

	1	2	3	4	5	6	7	8	9	10	11	12
A					0.379	0.370	0.484	0.478	0.220	0.291	0.404	0.398
B					0.157	0.155	0.155	0.157	0.194	0.200	0.080	0.091
C					0.623	0.633	0.542	0.548	0.625	0.640	0.197	0.192
D												
E												
F												
G							0.051	0.051	0.051	0.051	0.051	0.050
H							0.050	0.049	0.050	0.049	0.050	0.050

Plattenbelegung

	1	2	3	4	5	6	7	8	9	10	11	12
A					T9HN1	T9HN1	T9HN2	T9HN2	T9HN3	T9HN3	T9HN4	T9HN4
B					T9HS11	T9HS11	T9HS12	T9HS12	T9HS13	T9HS13	T9HS14	T9HS14
C					T9HS21	T9HS21	T9HS22	T9HS22	T9HS23	T9HS23	T9HS24	T9HS24
D												
E												
F												
G							Blank PEG	Blank PEG	Blank PEG	Blank PEG	Blank PEG	Blank PEG
H							Blank no PEG	Blank no PEG	Blank no PEG	Blank no PEG	Blank no PEG	Blank no PEG

Comment:

Date Operator

Date Control

0	T9	E01	0	0	kleiner LLOQ	#VALUE!	1	#VALUE!
0	T9	E02	0	0	kleiner LLOQ	#VALUE!	1	#VALUE!
0	T9	E03	0	0	kleiner LLOQ	#VALUE!	1	#VALUE!
0	T9	E04	0	0	kleiner LLOQ	#VALUE!	1	#VALUE!
0	T9	E05	0	0	kleiner LLOQ	#VALUE!	1	#VALUE!
0	T9	E06	0	0	kleiner LLOQ	#VALUE!	1	#VALUE!
0	T9	E07	0	0	kleiner LLOQ	#VALUE!	1	#VALUE!
0	T9	E08	0	0	kleiner LLOQ	#VALUE!	1	#VALUE!
0	T9	E09	0	0	kleiner LLOQ	#VALUE!	1	#VALUE!
0	T9	E10	0	0	kleiner LLOQ	#VALUE!	1	#VALUE!
0	T9	E11	0	0	kleiner LLOQ	#VALUE!	1	#VALUE!
0	T9	E12	0	0	kleiner LLOQ	#VALUE!	1	#VALUE!
0	T9	F01	0	0	kleiner LLOQ	#VALUE!	1	#VALUE!
0	T9	F02	0	0	kleiner LLOQ	#VALUE!	1	#VALUE!
0	T9	F03	0	0	kleiner LLOQ	#VALUE!	1	#VALUE!
0	T9	F04	0	0	kleiner LLOQ	#VALUE!	1	#VALUE!
0	T9	F05	0	0	kleiner LLOQ	#VALUE!	1	#VALUE!
0	T9	F06	0	0	kleiner LLOQ	#VALUE!	1	#VALUE!
0	T9	F07	0	0	kleiner LLOQ	#VALUE!	1	#VALUE!
0	T9	F08	0	0	kleiner LLOQ	#VALUE!	1	#VALUE!
0	T9	F09	0	0	kleiner LLOQ	#VALUE!	1	#VALUE!
0	T9	F10	0	0	kleiner LLOQ	#VALUE!	1	#VALUE!
0	T9	F11	0	0	kleiner LLOQ	#VALUE!	1	#VALUE!
0	T9	F12	0	0	kleiner LLOQ	#VALUE!	1	#VALUE!
0	T9	G01	0	0	kleiner LLOQ	#VALUE!	1	#VALUE!
0	T9	G02	0	0	kleiner LLOQ	#VALUE!	1	#VALUE!
0	T9	G03	0	0	kleiner LLOQ	#VALUE!	1	#VALUE!
0	T9	G04	0	0	kleiner LLOQ	#VALUE!	1	#VALUE!
0	T9	G05	0	0	kleiner LLOQ	#VALUE!	1	#VALUE!
0	T9	G06	0	0	kleiner LLOQ	#VALUE!	1	#VALUE!
Blank PEG	T9	G07	0	0	kleiner LLOQ	#VALUE!	2.75	#VALUE!
Blank PEG	T9	G08	0	0	kleiner LLOQ	#VALUE!	2.75	#VALUE!
Blank PEG	T9	G09	0	0	kleiner LLOQ	#VALUE!	2.75	#VALUE!
Blank PEG	T9	G10	0	0	kleiner LLOQ	#VALUE!	2.75	#VALUE!
Blank PEG	T9	G11	0	0	kleiner LLOQ	#VALUE!	2.75	#VALUE!
Blank PEG	T9	G12	0	0	kleiner LLOQ	#VALUE!	2.75	#VALUE!
0	T9	H01	0	0	kleiner LLOQ	#VALUE!	1	#VALUE!
0	T9	H02	0	0	kleiner LLOQ	#VALUE!	1	#VALUE!
0	T9	H03	0	0	kleiner LLOQ	#VALUE!	1	#VALUE!
0	T9	H04	0	0	kleiner LLOQ	#VALUE!	1	#VALUE!
0	T9	H05	0	0	kleiner LLOQ	#VALUE!	1	#VALUE!
0	T9	H06	0	0	kleiner LLOQ	#VALUE!	1	#VALUE!
Blank no PEG	T9	H07	0	0	kleiner LLOQ	#VALUE!	2.75	#VALUE!
Blank no PEG	T9	H08	0	0	kleiner LLOQ	#VALUE!	2.75	#VALUE!
Blank no PEG	T9	H09	0	0	kleiner LLOQ	#VALUE!	2.75	#VALUE!
Blank no PEG	T9	H10	0	0	kleiner LLOQ	#VALUE!	2.75	#VALUE!
Blank no PEG	T9	H11	0	0	kleiner LLOQ	#VALUE!	2.75	#VALUE!
Blank no PEG	T9	H12	0	0	kleiner LLOQ	#VALUE!	2.75	#VALUE!
0	T10	A01	0	0	kleiner LLOQ	#VALUE!	1	#VALUE!
0	T10	A02	0	0	kleiner LLOQ	#VALUE!	1	#VALUE!
0	T10	A03	0	0	kleiner LLOQ	#VALUE!	1	#VALUE!
0	T10	A04	0	0	kleiner LLOQ	#VALUE!	1	#VALUE!
T10HN1	T10	A05	0.337	0.286	0.286	0.012	2.75	0.033
T10HN1	T10	A06	0.335	0.285	0.285	0.012	2.75	0.033
T10HN2	T10	A07	0.457	0.406	0.406	0.018	2.75	0.049
T10HN2	T10	A08	0.454	0.403	0.403	0.018	2.75	0.048
T10HN3	T10	A09	0.421	0.370	0.370	0.016	2.75	0.044
T10HN3	T10	A10	0.439	0.388	0.388	0.017	2.75	0.046
T10HN4	T10	A11	0.367	0.316	0.316	0.013	2.75	0.037
T10HN4	T10	A12	0.373	0.322	0.322	0.014	2.75	0.038
0	T10	B01	0	0	kleiner LLOQ	#VALUE!	1	#VALUE!
0	T10	B02	0	0	kleiner LLOQ	#VALUE!	1	#VALUE!
0	T10	B03	0	0	kleiner LLOQ	#VALUE!	1	#VALUE!
0	T10	B04	0	0	kleiner LLOQ	#VALUE!	1	#VALUE!
T10HS11	T10	B05	0.137	0.086	kleiner LLOQ	#VALUE!	2.75	#VALUE!
T10HS11	T10	B06	0.138	0.087	kleiner LLOQ	#VALUE!	2.75	#VALUE!
T10HS12	T10	B07	0.156	0.105	kleiner LLOQ	#VALUE!	2.75	#VALUE!
T10HS12	T10	B08	0.166	0.115	kleiner LLOQ	#VALUE!	2.75	#VALUE!
T10HS13	T10	B09	0.178	0.127	kleiner LLOQ	#VALUE!	2.75	#VALUE!
T10HS13	T10	B10	0.182	0.131	kleiner LLOQ	#VALUE!	2.75	#VALUE!
T10HS14	T10	B11	0.087	0.037	kleiner LLOQ	#VALUE!	2.75	#VALUE!
T10HS14	T10	B12	0.082	0.031	kleiner LLOQ	#VALUE!	2.75	#VALUE!
0	T10	C01	0.000	-0.051	kleiner LLOQ	#VALUE!	1	#VALUE!

Plate:	T10

	1	2	3	4	5	6	7	8	9	10	11	12
A					0.337	0.335	0.457	0.454	0.421	0.439	0.367	0.373
B					0.137	0.138	0.156	0.166	0.178	0.182	0.087	0.082
C					0.645	0.646	0.519	0.550	0.557	0.572	0.193	0.182
D												
E												
F												
G							0.051	0.051	0.051	0.053	0.051	0.054
H							0.050	0.050	0.052	0.051	0.050	0.050

Plattenbelegung

	1	2	3	4	5	6	7	8	9	10	11	12
A					T10HN1	T10HN1	T10HN2	T10HN2	T10HN3	T10HN3	T10HN4	T10HN4
B					T10HS11	T10HS11	T10HS12	T10HS12	T10HS13	T10HS13	T10HS14	T10HS14
C					T10HS21	T10HS21	T10HS22	T10HS22	T10HS23	T10HS23	T10HS24	T10HS24
D												
E												

0	T10	C02	0.000	-0.051	kleiner LLOQ	#VALUE!	1	#VALUE!
0	T10	C03	0.000	-0.051	kleiner LLOQ	#VALUE!	1	#VALUE!
0	T10	C04	0.000	-0.051	kleiner LLOQ	#VALUE!	1	#VALUE!
T10HS21	T10	C05	0.645	0.594	0.594	0.027	2.75	0.073
T10HS21	T10	C06	0.646	0.595	0.595	0.027	2.75	0.073
T10HS22	T10	C07	0.519	0.468	0.468	0.021	2.75	0.057
T10HS22	T10	C08	0.550	0.499	0.499	0.022	2.75	0.061
T10HS23	T10	C09	0.557	0.506	0.506	0.022	2.75	0.062
T10HS23	T10	C10	0.572	0.521	0.521	0.023	2.75	0.064
T10HS24	T10	C11	0.193	0.142	kleiner LLOQ	#VALUE!	2.75	#VALUE!
T10HS24	T10	C12	0.182	0.131	kleiner LLOQ	#VALUE!	2.75	#VALUE!
0	T10	D01	0.000	-0.051	kleiner LLOQ	#VALUE!	1	#VALUE!
0	T10	D02	0.000	-0.051	kleiner LLOQ	#VALUE!	1	#VALUE!
0	T10	D03	0.000	-0.051	kleiner LLOQ	#VALUE!	1	#VALUE!
0	T10	D04	0	0	kleiner LLOQ	#VALUE!	1	#VALUE!
0	T10	D05	0	0	kleiner LLOQ	#VALUE!	1	#VALUE!
0	T10	D06	0	0	kleiner LLOQ	#VALUE!	1	#VALUE!
0	T10	D07	0	0	kleiner LLOQ	#VALUE!	1	#VALUE!
0	T10	D08	0	0	kleiner LLOQ	#VALUE!	1	#VALUE!
0	T10	D09	0	0	kleiner LLOQ	#VALUE!	1	#VALUE!
0	T10	D10	0	0	kleiner LLOQ	#VALUE!	1	#VALUE!
0	T10	D11	0	0	kleiner LLOQ	#VALUE!	1	#VALUE!
0	T10	D12	0	0	kleiner LLOQ	#VALUE!	1	#VALUE!
0	T10	E01	0	0	kleiner LLOQ	#VALUE!	1	#VALUE!
0	T10	E02	0	0	kleiner LLOQ	#VALUE!	1	#VALUE!
0	T10	E03	0	0	kleiner LLOQ	#VALUE!	1	#VALUE!
0	T10	E04	0	0	kleiner LLOQ	#VALUE!	1	#VALUE!
0	T10	E05	0	0	kleiner LLOQ	#VALUE!	1	#VALUE!
0	T10	E06	0	0	kleiner LLOQ	#VALUE!	1	#VALUE!
0	T10	E07	0	0	kleiner LLOQ	#VALUE!	1	#VALUE!
0	T10	E08	0	0	kleiner LLOQ	#VALUE!	1	#VALUE!
0	T10	E09	0	0	kleiner LLOQ	#VALUE!	1	#VALUE!
0	T10	E10	0	0	kleiner LLOQ	#VALUE!	1	#VALUE!
0	T10	E11	0	0	kleiner LLOQ	#VALUE!	1	#VALUE!
0	T10	E12	0	0	kleiner LLOQ	#VALUE!	1	#VALUE!
0	T10	F01	0	0	kleiner LLOQ	#VALUE!	1	#VALUE!
0	T10	F02	0	0	kleiner LLOQ	#VALUE!	1	#VALUE!
0	T10	F03	0	0	kleiner LLOQ	#VALUE!	1	#VALUE!
0	T10	F04	0	0	kleiner LLOQ	#VALUE!	1	#VALUE!
0	T10	F05	0	0	kleiner LLOQ	#VALUE!	1	#VALUE!
0	T10	F06	0	0	kleiner LLOQ	#VALUE!	1	#VALUE!
0	T10	F07	0	0	kleiner LLOQ	#VALUE!	1	#VALUE!
0	T10	F08	0	0	kleiner LLOQ	#VALUE!	1	#VALUE!
0	T10	F09	0	0	kleiner LLOQ	#VALUE!	1	#VALUE!
0	T10	F10	0	0	kleiner LLOQ	#VALUE!	1	#VALUE!
0	T10	F11	0	0	kleiner LLOQ	#VALUE!	1	#VALUE!
0	T10	F12	0	0	kleiner LLOQ	#VALUE!	1	#VALUE!
0	T10	G01	0	0	kleiner LLOQ	#VALUE!	1	#VALUE!
0	T10	G02	0	0	kleiner LLOQ	#VALUE!	1	#VALUE!
0	T10	G03	0	0	kleiner LLOQ	#VALUE!	1	#VALUE!
0	T10	G04	0	0	kleiner LLOQ	#VALUE!	1	#VALUE!
0	T10	G05	0	0	kleiner LLOQ	#VALUE!	1	#VALUE!
0	T10	G06	0	0	kleiner LLOQ	#VALUE!	1	#VALUE!
Blank PEG	T10	G07	0	0	kleiner LLOQ	#VALUE!	2.75	#VALUE!
Blank PEG	T10	G08	0	0	kleiner LLOQ	#VALUE!	2.75	#VALUE!
Blank PEG	T10	G09	0	0	kleiner LLOQ	#VALUE!	2.75	#VALUE!
Blank PEG	T10	G10	0	0	kleiner LLOQ	#VALUE!	2.75	#VALUE!
Blank PEG	T10	G11	0	0	kleiner LLOQ	#VALUE!	2.75	#VALUE!
Blank PEG	T10	G12	0	0	kleiner LLOQ	#VALUE!	2.75	#VALUE!
0	T10	H01	0	0	kleiner LLOQ	#VALUE!	1	#VALUE!
0	T10	H02	0	0	kleiner LLOQ	#VALUE!	1	#VALUE!
0	T10	H03	0	0	kleiner LLOQ	#VALUE!	1	#VALUE!
0	T10	H04	0	0	kleiner LLOQ	#VALUE!	1	#VALUE!
0	T10	H05	0	0	kleiner LLOQ	#VALUE!	1	#VALUE!
0	T10	H06	0	0	kleiner LLOQ	#VALUE!	1	#VALUE!
Blank no PEG	T10	H07	0	0	kleiner LLOQ	#VALUE!	2.75	#VALUE!
Blank no PEG	T10	H08	0	0	kleiner LLOQ	#VALUE!	2.75	#VALUE!
Blank no PEG	T10	H09	0	0	kleiner LLOQ	#VALUE!	2.75	#VALUE!
Blank no PEG	T10	H10	0	0	kleiner LLOQ	#VALUE!	2.75	#VALUE!
Blank no PEG	T10	H11	0	0	kleiner LLOQ	#VALUE!	2.75	#VALUE!
Blank no PEG	T10	H12	0	0	kleiner LLOQ	#VALUE!	2.75	#VALUE!
0	T11	A01	0	0	kleiner LLOQ	#VALUE!	1	#VALUE!
0	T11	A02	0	0	kleiner LLOQ	#VALUE!	1	#VALUE!
0	T11	A03	0	0	kleiner LLOQ	#VALUE!	1	#VALUE!
0	T11	A04	0	0	kleiner LLOQ	#VALUE!	1	#VALUE!
T11HN1	T11	A05	0.502	0.451	0.451	0.020	2.75	0.055

F											
G							Blank PEG	Blank PEG	Blank PEG	Blank PEG	Blank PEG	Blank PEG
H							Blank no PEG	Blank no PEG	Blank no PEG	Blank no PEG	Blank no PEG	Blank no PEG

Comment:

Date Operator

Date Control

T11HN1	T11	A06	0.545	0.494	0.494	0.022	2.75	0.060
T11HN2	T11	A07	0.613	0.562	0.562	0.025	2.75	0.069
T11HN2	T11	A08	0.593	0.543	0.543	0.024	2.75	0.066
T11HN3	T11	A09	0.579	0.528	0.528	0.023	2.75	0.065
T11HN3	T11	A10	0.537	0.486	0.486	0.021	2.75	0.059
T11HN4	T11	A11	0.385	0.334	0.334	0.014	2.75	0.039
T11HN4	T11	A12	0.388	0.337	0.337	0.014	2.75	0.040
0	T11	B01	0.000	-0.051	kleiner LLOQ	#VALUE!	1	#VALUE!
0	T11	B02	0.000	-0.051	kleiner LLOQ	#VALUE!	1	#VALUE!
0	T11	B03	0.000	-0.051	kleiner LLOQ	#VALUE!	1	#VALUE!
0	T11	B04	0.000	-0.051	kleiner LLOQ	#VALUE!	1	#VALUE!
T11HS11	T11	B05	0.206	0.156	kleiner LLOQ	#VALUE!	2.75	#VALUE!
T11HS11	T11	B06	0.205	0.154	kleiner LLOQ	#VALUE!	2.75	#VALUE!
T11HS12	T11	B07	0.187	0.136	kleiner LLOQ	#VALUE!	2.75	#VALUE!
T11HS12	T11	B08	0.189	0.138	kleiner LLOQ	#VALUE!	2.75	#VALUE!
T11HS13	T11	B09	0.187	0.136	kleiner LLOQ	#VALUE!	2.75	#VALUE!
T11HS13	T11	B10	0.177	0.126	kleiner LLOQ	#VALUE!	2.75	#VALUE!
T11HS14	T11	B11	0.122	0.071	kleiner LLOQ	#VALUE!	2.75	#VALUE!
T11HS14	T11	B12	0.114	0.063	kleiner LLOQ	#VALUE!	2.75	#VALUE!
0	T11	C01	0.000	-0.051	kleiner LLOQ	#VALUE!	1	#VALUE!
0	T11	C02	0.000	-0.051	kleiner LLOQ	#VALUE!	1	#VALUE!
0	T11	C03	0.000	-0.051	kleiner LLOQ	#VALUE!	1	#VALUE!
0	T11	C04	0.000	-0.051	kleiner LLOQ	#VALUE!	1	#VALUE!
T11HS21	T11	C05	0.731	0.680	0.680	0.031	2.75	0.084
T11HS21	T11	C06	0.737	0.686	0.686	0.031	2.75	0.085
T11HS22	T11	C07	0.575	0.524	0.524	0.023	2.75	0.064
T11HS22	T11	C08	0.570	0.519	0.519	0.023	2.75	0.063
T11HS23	T11	C09	0.538	0.488	0.488	0.022	2.75	0.059
T11HS23	T11	C10	0.531	0.480	0.480	0.021	2.75	0.058
T11HS24	T11	C11	0.208	0.157	kleiner LLOQ	#VALUE!	2.75	#VALUE!
T11HS24	T11	C12	0.207	0.156	kleiner LLOQ	#VALUE!	2.75	#VALUE!
0	T11	D01	0.000	-0.051	kleiner LLOQ	#VALUE!	1	#VALUE!
0	T11	D02	0.000	-0.051	kleiner LLOQ	#VALUE!	1	#VALUE!
0	T11	D03	0	0	kleiner LLOQ	#VALUE!	1	#VALUE!
0	T11	D04	0	0	kleiner LLOQ	#VALUE!	1	#VALUE!
0	T11	D05	0	0	kleiner LLOQ	#VALUE!	1	#VALUE!
0	T11	D06	0	0	kleiner LLOQ	#VALUE!	1	#VALUE!
0	T11	D07	0	0	kleiner LLOQ	#VALUE!	1	#VALUE!
0	T11	D08	0	0	kleiner LLOQ	#VALUE!	1	#VALUE!
0	T11	D09	0	0	kleiner LLOQ	#VALUE!	1	#VALUE!
0	T11	D10	0	0	kleiner LLOQ	#VALUE!	1	#VALUE!
0	T11	D11	0	0	kleiner LLOQ	#VALUE!	1	#VALUE!
0	T11	D12	0	0	kleiner LLOQ	#VALUE!	1	#VALUE!
0	T11	E01	0	0	kleiner LLOQ	#VALUE!	1	#VALUE!
0	T11	E02	0	0	kleiner LLOQ	#VALUE!	1	#VALUE!
0	T11	E03	0	0	kleiner LLOQ	#VALUE!	1	#VALUE!
0	T11	E04	0	0	kleiner LLOQ	#VALUE!	1	#VALUE!
0	T11	E05	0	0	kleiner LLOQ	#VALUE!	1	#VALUE!
0	T11	E06	0	0	kleiner LLOQ	#VALUE!	1	#VALUE!
0	T11	E07	0	0	kleiner LLOQ	#VALUE!	1	#VALUE!
0	T11	E08	0	0	kleiner LLOQ	#VALUE!	1	#VALUE!
0	T11	E09	0	0	kleiner LLOQ	#VALUE!	1	#VALUE!
0	T11	E10	0	0	kleiner LLOQ	#VALUE!	1	#VALUE!
0	T11	E11	0	0	kleiner LLOQ	#VALUE!	1	#VALUE!
0	T11	E12	0	0	kleiner LLOQ	#VALUE!	1	#VALUE!
0	T11	F01	0	0	kleiner LLOQ	#VALUE!	1	#VALUE!
0	T11	F02	0	0	kleiner LLOQ	#VALUE!	1	#VALUE!
0	T11	F03	0	0	kleiner LLOQ	#VALUE!	1	#VALUE!
0	T11	F04	0	0	kleiner LLOQ	#VALUE!	1	#VALUE!
0	T11	F05	0	0	kleiner LLOQ	#VALUE!	1	#VALUE!
0	T11	F06	0	0	kleiner LLOQ	#VALUE!	1	#VALUE!
0	T11	F07	0	0	kleiner LLOQ	#VALUE!	1	#VALUE!
0	T11	F08	0	0	kleiner LLOQ	#VALUE!	1	#VALUE!
0	T11	F09	0	0	kleiner LLOQ	#VALUE!	1	#VALUE!
0	T11	F10	0	0	kleiner LLOQ	#VALUE!	1	#VALUE!
0	T11	F11	0	0	kleiner LLOQ	#VALUE!	1	#VALUE!
0	T11	F12	0	0	kleiner LLOQ	#VALUE!	1	#VALUE!
0	T11	G01	0	0	kleiner LLOQ	#VALUE!	1	#VALUE!
0	T11	G02	0	0	kleiner LLOQ	#VALUE!	1	#VALUE!
0	T11	G03	0	0	kleiner LLOQ	#VALUE!	1	#VALUE!
0	T11	G04	0	0	kleiner LLOQ	#VALUE!	1	#VALUE!
0	T11	G05	0	0	kleiner LLOQ	#VALUE!	1	#VALUE!
0	T11	G06	0	0	kleiner LLOQ	#VALUE!	1	#VALUE!
Blank PEG	T11	G07	0	0	kleiner LLOQ	#VALUE!	2.75	#VALUE!

Plate: T11

	1	2	3	4	5	6	7	8	9	10	11	12
A					0.502	0.545	0.613	0.593	0.579	0.537	0.385	0.388
B					0.206	0.205	0.187	0.189	0.187	0.177	0.122	0.114
C					0.731	0.737	0.575	0.570	0.538	0.531	0.208	0.207
D												
E												
F												
G							0.052	0.052	0.052	0.056	0.052	0.052
H							0.050	0.050	0.050	0.050	0.050	0.049

Plattenbelegung

	1	2	3	4	5	6	7	8	9	10	11	12
A					T11HN1	T11HN1	T11HN2	T11HN2	T11HN3	T11HN3	T11HN4	T11HN4
B					T11HS11	T11HS11	T11HS12	T11HS12	T11HS13	T11HS13	T11HS14	T11HS14
C					T11HS21	T11HS21	T11HS22	T11HS22	T11HS23	T11HS23	T11HS24	T11HS24
D												
E												
F												
G							Blank PEG	Blank PEG	Blank PEG	Blank PEG	Blank PEG	Blank PEG
H							Blank no PEG	Blank no PEG	Blank no PEG	Blank no PEG	Blank no PEG	Blank no PEG

Comment:

Date Operator

Date Control

Blank PEG	T11	G08	0	0	kleiner LLOQ	#VALUE!	2.75	#VALUE!	
Blank PEG	T11	G09	0	0	kleiner LLOQ	#VALUE!	2.75	#VALUE!	
Blank PEG	T11	G10	0	0	kleiner LLOQ	#VALUE!	2.75	#VALUE!	
Blank PEG	T11	G11	0	0	kleiner LLOQ	#VALUE!	2.75	#VALUE!	
Blank PEG	T11	G12	0	0	kleiner LLOQ	#VALUE!	2.75	#VALUE!	
0	T11	H01	0	0	kleiner LLOQ	#VALUE!	1	#VALUE!	
0	T11	H02	0	0	kleiner LLOQ	#VALUE!	1	#VALUE!	
0	T11	H03	0	0	kleiner LLOQ	#VALUE!	1	#VALUE!	
0	T11	H04	0	0	kleiner LLOQ	#VALUE!	1	#VALUE!	
0	T11	H05	0	0	kleiner LLOQ	#VALUE!	1	#VALUE!	
0	T11	H06	0	0	kleiner LLOQ	#VALUE!	1	#VALUE!	
Blank no PEG	T11	H07	0	0	kleiner LLOQ	#VALUE!	2.75	#VALUE!	
Blank no PEG	T11	H08	0	0	kleiner LLOQ	#VALUE!	2.75	#VALUE!	
Blank no PEG	T11	H09	0	0	kleiner LLOQ	#VALUE!	2.75	#VALUE!	
Blank no PEG	T11	H10	0	0	kleiner LLOQ	#VALUE!	2.75	#VALUE!	
Blank no PEG	T11	H11	0	0	kleiner LLOQ	#VALUE!	2.75	#VALUE!	
Blank no PEG	T11	H12	0	0	kleiner LLOQ	#VALUE!	2.75	#VALUE!	

**Project number**		F-120	**Apparatus**	**Wallac Victor**	**Operator**	**IsBa**
**GLP Study (Number)**		n.a.	**Protocol (Instrument meth**	**LDH test 2016**	**Date of preparation**	**19-04-18**
**hot substance**	**isotope**	n.a.	**File name (results)**	**IsBa_180419/20_LDH_full_**	**Date of measurement**	**19-04-18**
	**name**	n.a.	**Kind of well plate**	**96 well**	**shaking time [min]**	**30**
	**ACB-ID**	n.a.	**sample volume [µL]**	**100**	**stirring rate (Target) [rpm]**	**150**
	**Batch number**	n.a.	**Cocktail volume [µL]**	**175**	**Kind of measurement**	**UV-vis**
**cold substance**	**name**	LDH	**ACB-ID of cocktail**		**Wave length [nm]**	**450**
	**ACB-ID**	L23982R	**Matrix**	**DMEM (from powder)+PE(**	**Remarks**	**Cocktail 100µl RM, 75µl STOP**
	**Batch number**	SLBK6345V	**Blank description**	**DMEM/PEG, H2O**	**Remarks**	**7 standards split low/high**
**n.a.**		n.a.	**Pipettes (No. / volume)**	**50-200µl**	**Remarks**	**KLP4 common for both**
**n.a.**		n.a.	**Pipettes (No. / volume)**	**n.a.**	**Remarks**	**n.a.**

Messdaten (diese Tabelle in Bericht übernehmen)

Sample name *	concentration (theor.) *	measured data	measured data	measured data	mean measured	SD	RSD	Blank *	measured data after * Blank subtraction	concentration (calc.) *	Deviation *	Residuen
	[µg/mL]	[AU]	[AU]	[AU]	[AU]	[AU]	[%]	[AU]		[µg/mL]	[%]	
KLP1	0.148	1.579	1.652	1.627	1.62	0.03	1.87	0.047	1.568	0.148	-0.24	0.00
KLP2	0.114	1.357	1.443	1.372	1.39	0.04	2.70	0.054	1.340	0.110	-3.40	0.00
KLP3	0.074	1.272	1.193	1.228	1.23	0.03	2.63	0.051	1.180	0.084	13.04	0.01
KLP4	0.041	0.959	0.972	0.883	0.94	0.04	4.19	0.051	0.887	0.035	-13.32	-0.01
KLP5								0.052				
KLP6								0.051				
KLP7												
KLP8												

Statistical data

Geradensteigung	Slope	m	6.05
Y-Achsenabschnitt	Y-Intercept	b	0.67
Standardabw. Geradensteigung	SD-Slope	s_m	0.620291227
Standardabw. Achsenabschnittes	SD-Y-Intercept	s_b	0.063694294
Anzahl Messpunkte	number of measuring points	n	4
Quadratsumme	sum of squares	Qxx	0.006578604
Bereichsmitte			0.094336926
Freiheitsgrade	degree of freedom	f	2
Student-t-Faktor für (P = 95 %; f = n-2)	Student-t-factor for (P = 95 %; f = n-2)	t	4.303
Vertrauensbereich Steig. (95 %) Obergrenze		$m + VB_m$	8.722319527
Vertrauensbereich Steig. (95 %) Untergrenze		$m - VB_m$	3.384093228
Vertrauensbereich Achsenabschnitt (95 %) Og.		$b + Vb_b$	0.946914615
Vertrauensbereich Achsenabschnitt (95 %) Ug.		$b - Vb_b$	0.398761518
Korrelationskoeffizient	correlation coefficient	r	0.9897
Bestimmtheitsmaß	determiantion coefficient	r^2	0.9794
Reststandardabweichung		s_0	0.050310948
Summe Restquadrate		sd	2.895583345
Verfahrensstandardabw.		s_{x0}	0.008311454
Rel. Verfahrensstandardabw. %		V_{x0}	8.810393374

mean Blank	**0**	
SD Blank	0.00	
RSD Blank	3.79	x=
x*SD (LLOQ)	0	5
x*SD (LOD)	0	3
**LLOQ (AU)**	**0**	
**LOD (AU)**	**0**	
ULOQ	1.568	
LLOQ (Lin)	0.887	

Evaluation / Comment

LDH linearity valid with 7 standards, split in lin high and lin low, each with 4 standards and KLP4 common standard for both. R^2 0,9981, deviations for both between -13.34% and +13.04%

Date	Operator	Date	Control

Across Barriers GmbH
Science Park 1
66123 Saarbrücken

Tel: 0681 95918-800
Fax: 0681 95918-802
e-Mail: sales@acrossbarriers.de
http://www.acrossbarriers.de

Formblatt-Nr.		Version	
Titel			
Gültig ab		Ablage	Projektordner

Figure 1 **Linearity**

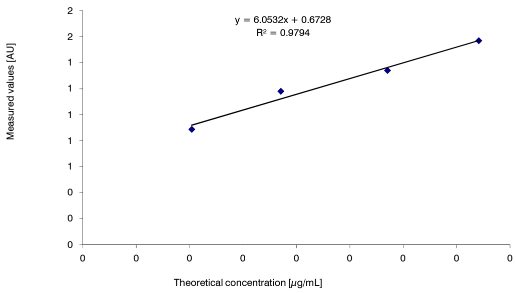

Figure 2 **Method validation Residuen Plot**

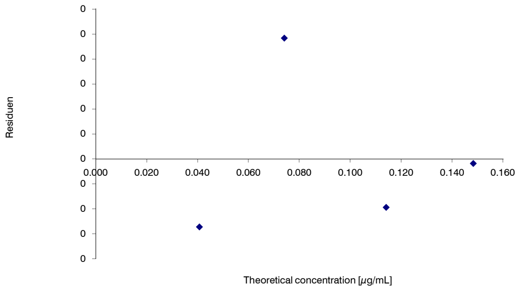

Evaluation / Comment

lin passed

Date	Operator	Date	Control

Sample name	Plate	Position	[AU]	[AU]-Blank	Rating	[µg/mL]	Dilution factor	[µg/mL]
0	T8	A01	0	0	kleiner LLOQ	#VALUE!	1	#VALUE!
0	T8	A02	0	0	kleiner LLOQ	#VALUE!	1	#VALUE!
0	T8	A03	0	0	kleiner LLOQ	#VALUE!	1	#VALUE!
0	T8	A04	0	0	kleiner LLOQ	#VALUE!	1	#VALUE!
T8HN1	T8	A05	0.000	-0.051	kleiner LLOQ	#VALUE!		#VALUE!
T8HN1	T8	A06	0.000	-0.051	kleiner LLOQ	#VALUE!		#VALUE!
T8HN2	T8	A07	0	0	kleiner LLOQ	#VALUE!		#VALUE!
T8HN2	T8	A08	0	0	kleiner LLOQ	#VALUE!		#VALUE!
T8HN3	T8	A09	0.000	-0.051	kleiner LLOQ	#VALUE!		#VALUE!
T8HN3	T8	A10	0.000	-0.051	kleiner LLOQ	#VALUE!		#VALUE!
T8HN4	T8	A11	0	0	kleiner LLOQ	#VALUE!		#VALUE!
T8HN4	T8	A12	0	0	kleiner LLOQ	#VALUE!		#VALUE!
0	T8	B01	0	0	kleiner LLOQ	#VALUE!		#VALUE!
0	T8	B02	0	0	kleiner LLOQ	#VALUE!		#VALUE!
0	T8	B03	0	0	kleiner LLOQ	#VALUE!		#VALUE!
0	T8	B04	0	0	kleiner LLOQ	#VALUE!		#VALUE!
T8HS11	T8	B05	0	0	kleiner LLOQ	#VALUE!		#VALUE!
T8HS11	T8	B06	0.000	-0.051	kleiner LLOQ	#VALUE!		#VALUE!
T8HS12	T8	B07	0.000	-0.051	kleiner LLOQ	#VALUE!		#VALUE!
T8HS12	T8	B08	0.000	-0.051	kleiner LLOQ	#VALUE!		#VALUE!
T8HS13	T8	B09	0	0	kleiner LLOQ	#VALUE!		#VALUE!
T8HS13	T8	B10	0	0	kleiner LLOQ	#VALUE!		#VALUE!
T8HS14	T8	B11	0	0	kleiner LLOQ	#VALUE!		#VALUE!
T8HS14	T8	B12	0	0	kleiner LLOQ	#VALUE!		#VALUE!
0	T8	C01	0	0	kleiner LLOQ	#VALUE!		#VALUE!
0	T8	C02	0	0	kleiner LLOQ	#VALUE!		#VALUE!
0	T8	C03	0	0	kleiner LLOQ	#VALUE!		#VALUE!
0	T8	C04	0	0	kleiner LLOQ	#VALUE!		#VALUE!
T8HS21	T8	C05	0.000	-0.051	kleiner LLOQ	#VALUE!		#VALUE!
T8HS21	T8	C06	0.000	-0.051	kleiner LLOQ	#VALUE!		#VALUE!
T8HS22	T8	C07	0.000	-0.051	kleiner LLOQ	#VALUE!		#VALUE!
T8HS22	T8	C08	0.000	-0.051	kleiner LLOQ	#VALUE!		#VALUE!
T8HS23	T8	C09	0.000	-0.051	kleiner LLOQ	#VALUE!		#VALUE!
T8HS23	T8	C10	0.000	-0.051	kleiner LLOQ	#VALUE!		#VALUE!
T8HS24	T8	C11	0	0	kleiner LLOQ	#VALUE!	1	#VALUE!
T8HS24	T8	C12	0	0	kleiner LLOQ	#VALUE!	1	#VALUE!
0	T8	D01	0	0	kleiner LLOQ	#VALUE!	1	#VALUE!
0	T8	D02	0	0	kleiner LLOQ	#VALUE!	1	#VALUE!
0	T8	D03	0	0	kleiner LLOQ	#VALUE!	1	#VALUE!
0	T8	D04	0	0	kleiner LLOQ	#VALUE!	1	#VALUE!
0	T8	D05	0	0	kleiner LLOQ	#VALUE!	1	#VALUE!
0	T8	D06	0	0	kleiner LLOQ	#VALUE!	1	#VALUE!
0	T8	D07	0	0	kleiner LLOQ	#VALUE!	1	#VALUE!
0	T8	D08	0	0	kleiner LLOQ	#VALUE!	1	#VALUE!
0	T8	D09	0	0	kleiner LLOQ	#VALUE!	1	#VALUE!
0	T8	D10	0	0	kleiner LLOQ	#VALUE!	1	#VALUE!
0	T8	D11	0	0	kleiner LLOQ	#VALUE!	1	#VALUE!
0	T8	D12	0	0	kleiner LLOQ	#VALUE!	1	#VALUE!

Plate: T8

	1	2	3	4	5	6	7	8	9	10	11	12
A												
B												
C												
D												
E												
F												
G							0.053	0.055	0.053	0.054	0.053	0.053
H							0.053	0.052	0.051	0.052	0.052	0.052

Plattenbelegung

	1	2	3	4	5	6	7	8	9	10	11	12
A					T8HN1	T8HN1	T8HN2	T8HN2	T8HN3	T8HN3	T8HN4	T8HN4
B					T8HS11	T8HS11	T8HS12	T8HS12	T8HS13	T8HS13	T8HS14	T8HS14
C					T8HS21	T8HS21	T8HS22	T8HS22	T8HS23	T8HS23	T8HS24	T8HS24
D												
E												
F												
G							Blank PEG	Blank PEG	Blank PEG	Blank PEG	Blank PEG	Blank PEG
H							Blank no PEG	Blank no PEG	Blank no PEG	Blank no PEG	Blank no PEG	Blank no PEG

Comment:

alle Messwerte bei lin low.

Samples aufgeteilt in lin high und low:niedriger konzentrierte Samples bei lin low zu finden.

Date Operator

Date Control

0	T8	E01	0	0	kleiner LLOQ	#VALUE!	1	#VALUE!
0	T8	E02	0	0	kleiner LLOQ	#VALUE!	1	#VALUE!
0	T8	E03	0	0	kleiner LLOQ	#VALUE!	1	#VALUE!
0	T8	E04	0	0	kleiner LLOQ	#VALUE!	1	#VALUE!
0	T8	E05	0	0	kleiner LLOQ	#VALUE!	1	#VALUE!
0	T8	E06	0	0	kleiner LLOQ	#VALUE!	1	#VALUE!
0	T8	E07	0	0	kleiner LLOQ	#VALUE!	1	#VALUE!
0	T8	E08	0	0	kleiner LLOQ	#VALUE!	1	#VALUE!
0	T8	E09	0	0	kleiner LLOQ	#VALUE!	1	#VALUE!
0	T8	E10	0	0	kleiner LLOQ	#VALUE!	1	#VALUE!
0	T8	E11	0	0	kleiner LLOQ	#VALUE!	1	#VALUE!
0	T8	E12	0	0	kleiner LLOQ	#VALUE!	1	#VALUE!
0	T8	F01	0	0	kleiner LLOQ	#VALUE!	1	#VALUE!
0	T8	F02	0	0	kleiner LLOQ	#VALUE!	1	#VALUE!
0	T8	F03	0	0	kleiner LLOQ	#VALUE!	1	#VALUE!
0	T8	F04	0	0	kleiner LLOQ	#VALUE!	1	#VALUE!
0	T8	F05	0	0	kleiner LLOQ	#VALUE!	1	#VALUE!
0	T8	F06	0	0	kleiner LLOQ	#VALUE!	1	#VALUE!
0	T8	F07	0	0	kleiner LLOQ	#VALUE!	1	#VALUE!
0	T8	F08	0	0	kleiner LLOQ	#VALUE!	1	#VALUE!
0	T8	F09	0	0	kleiner LLOQ	#VALUE!	1	#VALUE!
0	T8	F10	0	0	kleiner LLOQ	#VALUE!	1	#VALUE!
0	T8	F11	0	0	kleiner LLOQ	#VALUE!	1	#VALUE!
0	T8	F12	0	0	kleiner LLOQ	#VALUE!	1	#VALUE!
0	T8	G01	0	0	kleiner LLOQ	#VALUE!	1	#VALUE!
0	T8	G02	0	0	kleiner LLOQ	#VALUE!	1	#VALUE!
0	T8	G03	0	0	kleiner LLOQ	#VALUE!	1	#VALUE!
0	T8	G04	0	0	kleiner LLOQ	#VALUE!	1	#VALUE!
0	T8	G05	0	0	kleiner LLOQ	#VALUE!	1	#VALUE!
0	T8	G06	0	0	kleiner LLOQ	#VALUE!	1	#VALUE!
Blank PEG	T8	G07	0.053	0.002	kleiner LLOQ	#VALUE!	2.75	#VALUE!
Blank PEG	T8	G08	0.055	0.004	kleiner LLOQ	#VALUE!	2.75	#VALUE!
Blank PEG	T8	G09	0.053	0.002	kleiner LLOQ	#VALUE!	2.75	#VALUE!
Blank PEG	T8	G10	0.054	0.003	kleiner LLOQ	#VALUE!	2.75	#VALUE!
Blank PEG	T8	G11	0.053	0.002	kleiner LLOQ	#VALUE!	2.75	#VALUE!
Blank PEG	T8	G12	0.053	0.002	kleiner LLOQ	#VALUE!	2.75	#VALUE!
0	T8	H01	0	0	kleiner LLOQ	#VALUE!	1	#VALUE!
0	T8	H02	0	0	kleiner LLOQ	#VALUE!	1	#VALUE!
0	T8	H03	0	0	kleiner LLOQ	#VALUE!	1	#VALUE!
0	T8	H04	0	0	kleiner LLOQ	#VALUE!	1	#VALUE!
0	T8	H05	0	0	kleiner LLOQ	#VALUE!	1	#VALUE!
0	T8	H06	0	0	kleiner LLOQ	#VALUE!	1	#VALUE!
Blank no PEG	T8	H07	0.053	0.002	kleiner LLOQ	#VALUE!	2.75	#VALUE!
Blank no PEG	T8	H08	0.052	0.001	kleiner LLOQ	#VALUE!	2.75	#VALUE!
Blank no PEG	T8	H09	0.051	0.000	kleiner LLOQ	#VALUE!	2.75	#VALUE!
Blank no PEG	T8	H10	0.052	0.001	kleiner LLOQ	#VALUE!	2.75	#VALUE!
Blank no PEG	T8	H11	0.052	0.001	kleiner LLOQ	#VALUE!	2.75	#VALUE!
Blank no PEG	T8	H12	0.052	0.001	kleiner LLOQ	#VALUE!	2.75	#VALUE!

0	T9	A01	0	0	kleiner LLOQ	#VALUE!	1	#VALUE!
0	T9	A02	0	0	kleiner LLOQ	#VALUE!	1	#VALUE!
0	T9	A03	0	0	kleiner LLOQ	#VALUE!	1	#VALUE!
0	T9	A04	0	0	kleiner LLOQ	#VALUE!	1	#VALUE!
T9HN1	T9	A05	0	0	kleiner LLOQ	#VALUE!	1	#VALUE!
T9HN1	T9	A06	0	0	kleiner LLOQ	#VALUE!	1	#VALUE!
T9HN2	T9	A07	0	0	kleiner LLOQ	#VALUE!	1	#VALUE!
T9HN2	T9	A08	0	0	kleiner LLOQ	#VALUE!	1	#VALUE!
T9HN3	T9	A09	0	0	kleiner LLOQ	#VALUE!	1	#VALUE!
T9HN3	T9	A10	0	0	kleiner LLOQ	#VALUE!	1	#VALUE!
T9HN4	T9	A11	0	0	kleiner LLOQ	#VALUE!	1	#VALUE!
T9HN4	T9	A12	0	0	kleiner LLOQ	#VALUE!	1	#VALUE!
0	T9	B01	0	0	kleiner LLOQ	#VALUE!	1	#VALUE!
0	T9	B02	0	0	kleiner LLOQ	#VALUE!	1	#VALUE!
0	T9	B03	0	0	kleiner LLOQ	#VALUE!	1	#VALUE!
0	T9	B04	0	0	kleiner LLOQ	#VALUE!	1	#VALUE!
T9HS11	T9	B05	0	0	kleiner LLOQ	#VALUE!	1	#VALUE!
T9HS11	T9	B06	0	0	kleiner LLOQ	#VALUE!	1	#VALUE!
T9HS12	T9	B07	0	0	kleiner LLOQ	#VALUE!	1	#VALUE!
T9HS12	T9	B08	0	0	kleiner LLOQ	#VALUE!	1	#VALUE!
T9HS13	T9	B09	0	0	kleiner LLOQ	#VALUE!	1	#VALUE!
T9HS13	T9	B10	0	0	kleiner LLOQ	#VALUE!	1	#VALUE!
T9HS14	T9	B11	0	0	kleiner LLOQ	#VALUE!	1	#VALUE!
T9HS14	T9	B12	0	0	kleiner LLOQ	#VALUE!	1	#VALUE!
0	T9	C01	0	0	kleiner LLOQ	#VALUE!	1	#VALUE!
0	T9	C02	0	0	kleiner LLOQ	#VALUE!	1	#VALUE!
0	T9	C03	0	0	kleiner LLOQ	#VALUE!	1	#VALUE!
0	T9	C04	0	0	kleiner LLOQ	#VALUE!	1	#VALUE!
T9HS21	T9	C05	0	0	kleiner LLOQ	#VALUE!	1	#VALUE!
T9HS21	T9	C06	0	0	kleiner LLOQ	#VALUE!	1	#VALUE!
T9HS22	T9	C07	0	0	kleiner LLOQ	#VALUE!	1	#VALUE!
T9HS22	T9	C08	0	0	kleiner LLOQ	#VALUE!	1	#VALUE!
T9HS23	T9	C09	0	0	kleiner LLOQ	#VALUE!	1	#VALUE!
T9HS23	T9	C10	0	0	kleiner LLOQ	#VALUE!	1	#VALUE!
T9HS24	T9	C11	0	0	kleiner LLOQ	#VALUE!	1	#VALUE!
T9HS24	T9	C12	0	0	kleiner LLOQ	#VALUE!	1	#VALUE!
0	T9	D01	0	0	kleiner LLOQ	#VALUE!	1	#VALUE!
0	T9	D02	0	0	kleiner LLOQ	#VALUE!	1	#VALUE!
0	T9	D03	0	0	kleiner LLOQ	#VALUE!	1	#VALUE!
0	T9	D04	0	0	kleiner LLOQ	#VALUE!	1	#VALUE!
0	T9	D05	0	0	kleiner LLOQ	#VALUE!	1	#VALUE!
0	T9	D06	0	0	kleiner LLOQ	#VALUE!	1	#VALUE!
0	T9	D07	0	0	kleiner LLOQ	#VALUE!	1	#VALUE!
0	T9	D08	0	0	kleiner LLOQ	#VALUE!	1	#VALUE!
0	T9	D09	0	0	kleiner LLOQ	#VALUE!	1	#VALUE!
0	T9	D10	0	0	kleiner LLOQ	#VALUE!	1	#VALUE!
0	T9	D11	0	0	kleiner LLOQ	#VALUE!	1	#VALUE!
0	T9	D12	0	0	kleiner LLOQ	#VALUE!	1	#VALUE!

Plate: T9

	1	2	3	4	5	6	7	8	9	10	11	12
A												
B												
C												
D												
E												
F												
G							0.0509	0.0505	0.0511	0.0506	0.0508	0.0498
H							0.0496	0.0490	0.0495	0.0494	0.0496	0.0497

Plattenbelegung

	1	2	3	4	5	6	7	8	9	10	11	12
A					T9HN1	T9HN1	T9HN2	T9HN2	T9HN3	T9HN3	T9HN4	T9HN4
B					T9HS11	T9HS11	T9HS12	T9HS12	T9HS13	T9HS13	T9HS14	T9HS14
C					T9HS21	T9HS21	T9HS22	T9HS22	T9HS23	T9HS23	T9HS24	T9HS24
D												
E												
F												
G							Blank PEG	Blank PEG	Blank PEG	Blank PEG	Blank PEG	Blank PEG
H							Blank no PEG	Blank no PEG	Blank no PEG	Blank no PEG	Blank no PEG	Blank no PEG

Comment:

alle Messwerte bei lin low.

Date Operator

Date Control

0	T9	E01	0	0	kleiner LLOQ	#VALUE!	1	#VALUE!
0	T9	E02	0	0	kleiner LLOQ	#VALUE!	1	#VALUE!
0	T9	E03	0	0	kleiner LLOQ	#VALUE!	1	#VALUE!
0	T9	E04	0	0	kleiner LLOQ	#VALUE!	1	#VALUE!
0	T9	E05	0	0	kleiner LLOQ	#VALUE!	1	#VALUE!
0	T9	E06	0	0	kleiner LLOQ	#VALUE!	1	#VALUE!
0	T9	E07	0	0	kleiner LLOQ	#VALUE!	1	#VALUE!
0	T9	E08	0	0	kleiner LLOQ	#VALUE!	1	#VALUE!
0	T9	E09	0	0	kleiner LLOQ	#VALUE!	1	#VALUE!
0	T9	E10	0	0	kleiner LLOQ	#VALUE!	1	#VALUE!
0	T9	E11	0	0	kleiner LLOQ	#VALUE!	1	#VALUE!
0	T9	E12	0	0	kleiner LLOQ	#VALUE!	1	#VALUE!
0	T9	F01	0	0	kleiner LLOQ	#VALUE!	1	#VALUE!
0	T9	F02	0	0	kleiner LLOQ	#VALUE!	1	#VALUE!
0	T9	F03	0	0	kleiner LLOQ	#VALUE!	1	#VALUE!
0	T9	F04	0	0	kleiner LLOQ	#VALUE!	1	#VALUE!
0	T9	F05	0	0	kleiner LLOQ	#VALUE!	1	#VALUE!
0	T9	F06	0	0	kleiner LLOQ	#VALUE!	1	#VALUE!
0	T9	F07	0	0	kleiner LLOQ	#VALUE!	1	#VALUE!
0	T9	F08	0	0	kleiner LLOQ	#VALUE!	1	#VALUE!
0	T9	F09	0	0	kleiner LLOQ	#VALUE!	1	#VALUE!
0	T9	F10	0	0	kleiner LLOQ	#VALUE!	1	#VALUE!
0	T9	F11	0	0	kleiner LLOQ	#VALUE!	1	#VALUE!
0	T9	F12	0	0	kleiner LLOQ	#VALUE!	1	#VALUE!
0	T9	G01	0	0	kleiner LLOQ	#VALUE!	1	#VALUE!
0	T9	G02	0	0	kleiner LLOQ	#VALUE!	1	#VALUE!
0	T9	G03	0	0	kleiner LLOQ	#VALUE!	1	#VALUE!
0	T9	G04	0	0	kleiner LLOQ	#VALUE!	1	#VALUE!
0	T9	G05	0	0	kleiner LLOQ	#VALUE!	1	#VALUE!
0	T9	G06	0	0	kleiner LLOQ	#VALUE!	1	#VALUE!
Blank PEG	T9	G07	0	0	kleiner LLOQ	#VALUE!	1	#VALUE!
Blank PEG	T9	G08	0	0	kleiner LLOQ	#VALUE!	1	#VALUE!
Blank PEG	T9	G09	0	0	kleiner LLOQ	#VALUE!	1	#VALUE!
Blank PEG	T9	G10	0	0	kleiner LLOQ	#VALUE!	1	#VALUE!
Blank PEG	T9	G11	0	0	kleiner LLOQ	#VALUE!	1	#VALUE!
Blank PEG	T9	G12	0	0	kleiner LLOQ	#VALUE!	1	#VALUE!
0	T9	H01	0	0	kleiner LLOQ	#VALUE!	1	#VALUE!
0	T9	H02	0	0	kleiner LLOQ	#VALUE!	1	#VALUE!
0	T9	H03	0	0	kleiner LLOQ	#VALUE!	1	#VALUE!
0	T9	H04	0	0	kleiner LLOQ	#VALUE!	1	#VALUE!
0	T9	H05	0	0	kleiner LLOQ	#VALUE!	1	#VALUE!
0	T9	H06	0	0	kleiner LLOQ	#VALUE!	1	#VALUE!
Blank no PEG	T9	H07	0	0	kleiner LLOQ	#VALUE!	1	#VALUE!
Blank no PEG	T9	H08	0	0	kleiner LLOQ	#VALUE!	1	#VALUE!
Blank no PEG	T9	H09	0	0	kleiner LLOQ	#VALUE!	1	#VALUE!
Blank no PEG	T9	H10	0	0	kleiner LLOQ	#VALUE!	1	#VALUE!
Blank no PEG	T9	H11	0	0	kleiner LLOQ	#VALUE!	1	#VALUE!
Blank no PEG	T9	H12	0	0	kleiner LLOQ	#VALUE!	1	#VALUE!
0	T10	A01	0	0	kleiner LLOQ	#VALUE!	1	#VALUE!
0	T10	A02	0	0	kleiner LLOQ	#VALUE!	1	#VALUE!
0	T10	A03	0	0	kleiner LLOQ	#VALUE!	1	#VALUE!
0	T10	A04	0	0	kleiner LLOQ	#VALUE!	1	#VALUE!
T10HN1	T10	A05	0	0	kleiner LLOQ	#VALUE!	1	#VALUE!
T10HN1	T10	A06	0	0	kleiner LLOQ	#VALUE!	1	#VALUE!
T10HN2	T10	A07	0	0	kleiner LLOQ	#VALUE!	1	#VALUE!
T10HN2	T10	A08	0	0	kleiner LLOQ	#VALUE!	1	#VALUE!
T10HN3	T10	A09	0	0	kleiner LLOQ	#VALUE!	1	#VALUE!
T10HN3	T10	A10	0	0	kleiner LLOQ	#VALUE!	1	#VALUE!
T10HN4	T10	A11	0	0	kleiner LLOQ	#VALUE!	1	#VALUE!
T10HN4	T10	A12	0	0	kleiner LLOQ	#VALUE!	1	#VALUE!
0	T10	B01	0	0	kleiner LLOQ	#VALUE!	1	#VALUE!
0	T10	B02	0	0	kleiner LLOQ	#VALUE!	1	#VALUE!
0	T10	B03	0	0	kleiner LLOQ	#VALUE!	1	#VALUE!
0	T10	B04	0	0	kleiner LLOQ	#VALUE!	1	#VALUE!
T10HS11	T10	B05	0	0	kleiner LLOQ	#VALUE!	1	#VALUE!
T10HS11	T10	B06	0	0	kleiner LLOQ	#VALUE!	1	#VALUE!
T10HS12	T10	B07	0	0	kleiner LLOQ	#VALUE!	1	#VALUE!
T10HS12	T10	B08	0	0	kleiner LLOQ	#VALUE!	1	#VALUE!
T10HS13	T10	B09	0	0	kleiner LLOQ	#VALUE!	1	#VALUE!
T10HS13	T10	B10	0	0	kleiner LLOQ	#VALUE!	1	#VALUE!
T10HS14	T10	B11	0	0	kleiner LLOQ	#VALUE!	1	#VALUE!
T10HS14	T10	B12	0	0	kleiner LLOQ	#VALUE!	1	#VALUE!
0	T10	C01	0	0	kleiner LLOQ	#VALUE!	1	#VALUE!

Plate:	T10

	1	2	3	4	5	6	7	8	9	10	11	12
A												
B												
C												
D												
E												
F												
G							0.051	0.051	0.051	0.053	0.051	0.054
H							0.050	0.050	0.052	0.051	0.050	0.050

Plattenbelegung

	1	2	3	4	5	6	7	8	9	10	11	12
A					T10HN1	T10HN1	T10HN2	T10HN2	T10HN3	T10HN3	T10HN4	T10HN4
B					T10HS11	T10HS11	T10HS12	T10HS12	T10HS13	T10HS13	T10HS14	T10HS14
C					T10HS21	T10HS21	T10HS22	T10HS22	T10HS23	T10HS23	T10HS24	T10HS24
D												
E												

0	T10	C02	0	0	kleiner LLOQ	#VALUE!	1	#VALUE!
0	T10	C03	0	0	kleiner LLOQ	#VALUE!	1	#VALUE!
0	T10	C04	0	0	kleiner LLOQ	#VALUE!	1	#VALUE!
T10HS21	T10	C05	0	0	kleiner LLOQ	#VALUE!	1	#VALUE!
T10HS21	T10	C06	0	0	kleiner LLOQ	#VALUE!	1	#VALUE!
T10HS22	T10	C07	0	0	kleiner LLOQ	#VALUE!	1	#VALUE!
T10HS22	T10	C08	0	0	kleiner LLOQ	#VALUE!	1	#VALUE!
T10HS23	T10	C09	0	0	kleiner LLOQ	#VALUE!	1	#VALUE!
T10HS23	T10	C10	0	0	kleiner LLOQ	#VALUE!	1	#VALUE!
T10HS24	T10	C11	0	0	kleiner LLOQ	#VALUE!	1	#VALUE!
T10HS24	T10	C12	0	0	kleiner LLOQ	#VALUE!	1	#VALUE!
0	T10	D01	0	0	kleiner LLOQ	#VALUE!	1	#VALUE!
0	T10	D02	0	0	kleiner LLOQ	#VALUE!	1	#VALUE!
0	T10	D03	0	0	kleiner LLOQ	#VALUE!	1	#VALUE!
0	T10	D04	0	0	kleiner LLOQ	#VALUE!	1	#VALUE!
0	T10	D05	0	0	kleiner LLOQ	#VALUE!	1	#VALUE!
0	T10	D06	0	0	kleiner LLOQ	#VALUE!	1	#VALUE!
0	T10	D07	0	0	kleiner LLOQ	#VALUE!	1	#VALUE!
0	T10	D08	0	0	kleiner LLOQ	#VALUE!	1	#VALUE!
0	T10	D09	0	0	kleiner LLOQ	#VALUE!	1	#VALUE!
0	T10	D10	0	0	kleiner LLOQ	#VALUE!	1	#VALUE!
0	T10	D11	0	0	kleiner LLOQ	#VALUE!	1	#VALUE!
0	T10	D12	0	0	kleiner LLOQ	#VALUE!	1	#VALUE!
0	T10	E01	0	0	kleiner LLOQ	#VALUE!	1	#VALUE!
0	T10	E02	0	0	kleiner LLOQ	#VALUE!	1	#VALUE!
0	T10	E03	0	0	kleiner LLOQ	#VALUE!	1	#VALUE!
0	T10	E04	0	0	kleiner LLOQ	#VALUE!	1	#VALUE!
0	T10	E05	0	0	kleiner LLOQ	#VALUE!	1	#VALUE!
0	T10	E06	0	0	kleiner LLOQ	#VALUE!	1	#VALUE!
0	T10	E07	0	0	kleiner LLOQ	#VALUE!	1	#VALUE!
0	T10	E08	0	0	kleiner LLOQ	#VALUE!	1	#VALUE!
0	T10	E09	0	0	kleiner LLOQ	#VALUE!	1	#VALUE!
0	T10	E10	0	0	kleiner LLOQ	#VALUE!	1	#VALUE!
0	T10	E11	0	0	kleiner LLOQ	#VALUE!	1	#VALUE!
0	T10	E12	0	0	kleiner LLOQ	#VALUE!	1	#VALUE!
0	T10	F01	0	0	kleiner LLOQ	#VALUE!	1	#VALUE!
0	T10	F02	0	0	kleiner LLOQ	#VALUE!	1	#VALUE!
0	T10	F03	0	0	kleiner LLOQ	#VALUE!	1	#VALUE!
0	T10	F04	0	0	kleiner LLOQ	#VALUE!	1	#VALUE!
0	T10	F05	0	0	kleiner LLOQ	#VALUE!	1	#VALUE!
0	T10	F06	0	0	kleiner LLOQ	#VALUE!	1	#VALUE!
0	T10	F07	0	0	kleiner LLOQ	#VALUE!	1	#VALUE!
0	T10	F08	0	0	kleiner LLOQ	#VALUE!	1	#VALUE!
0	T10	F09	0	0	kleiner LLOQ	#VALUE!	1	#VALUE!
0	T10	F10	0	0	kleiner LLOQ	#VALUE!	1	#VALUE!
0	T10	F11	0	0	kleiner LLOQ	#VALUE!	1	#VALUE!
0	T10	F12	0	0	kleiner LLOQ	#VALUE!	1	#VALUE!
0	T10	G01	0	0	kleiner LLOQ	#VALUE!	1	#VALUE!
0	T10	G02	0	0	kleiner LLOQ	#VALUE!	1	#VALUE!
0	T10	G03	0	0	kleiner LLOQ	#VALUE!	1	#VALUE!
0	T10	G04	0	0	kleiner LLOQ	#VALUE!	1	#VALUE!
0	T10	G05	0	0	kleiner LLOQ	#VALUE!	1	#VALUE!
0	T10	G06	0	0	kleiner LLOQ	#VALUE!	1	#VALUE!
Blank PEG	T10	G07	0	0	kleiner LLOQ	#VALUE!	1	#VALUE!
Blank PEG	T10	G08	0	0	kleiner LLOQ	#VALUE!	1	#VALUE!
Blank PEG	T10	G09	0	0	kleiner LLOQ	#VALUE!	1	#VALUE!
Blank PEG	T10	G10	0	0	kleiner LLOQ	#VALUE!	1	#VALUE!
Blank PEG	T10	G11	0	0	kleiner LLOQ	#VALUE!	1	#VALUE!
Blank PEG	T10	G12	0	0	kleiner LLOQ	#VALUE!	1	#VALUE!
0	T10	H01	0	0	kleiner LLOQ	#VALUE!	1	#VALUE!
0	T10	H02	0	0	kleiner LLOQ	#VALUE!	1	#VALUE!
0	T10	H03	0	0	kleiner LLOQ	#VALUE!	1	#VALUE!
0	T10	H04	0	0	kleiner LLOQ	#VALUE!	1	#VALUE!
0	T10	H05	0	0	kleiner LLOQ	#VALUE!	1	#VALUE!
0	T10	H06	0	0	kleiner LLOQ	#VALUE!	1	#VALUE!
Blank no PEG	T10	H07	0	0	kleiner LLOQ	#VALUE!	1	#VALUE!
Blank no PEG	T10	H08	0	0	kleiner LLOQ	#VALUE!	1	#VALUE!
Blank no PEG	T10	H09	0	0	kleiner LLOQ	#VALUE!	1	#VALUE!
Blank no PEG	T10	H10	0	0	kleiner LLOQ	#VALUE!	1	#VALUE!
Blank no PEG	T10	H11	0	0	kleiner LLOQ	#VALUE!	1	#VALUE!
Blank no PEG	T10	H12	0	0	kleiner LLOQ	#VALUE!	1	#VALUE!

F												
G							Blank PEG	Blank PEG	Blank PEG	Blank PEG	Blank PEG	Blank PEG
H							Blank no PEG	Blank no PEG	Blank no PEG	Blank no PEG	Blank no PEG	Blank no PEG

Comment:

alle Messwerte bei lin low.

Date Operator

Date Control

0	T11	A01	0	0	kleiner LLOQ	#VALUE!	1	#VALUE!
0	T11	A02	0	0	kleiner LLOQ	#VALUE!	1	#VALUE!
0	T11	A03	0	0	kleiner LLOQ	#VALUE!	1	#VALUE!
0	T11	A04	0	0	kleiner LLOQ	#VALUE!	1	#VALUE!
T11HN1	T11	A05	0	0	kleiner LLOQ	#VALUE!	1	#VALUE!
T11HN1	T11	A06	0	0	kleiner LLOQ	#VALUE!	1	#VALUE!
T11HN2	T11	A07	0	0	kleiner LLOQ	#VALUE!	1	#VALUE!
T11HN2	T11	A08	0	0	kleiner LLOQ	#VALUE!	1	#VALUE!
T11HN3	T11	A09	0	0	kleiner LLOQ	#VALUE!	1	#VALUE!
T11HN3	T11	A10	0	0	kleiner LLOQ	#VALUE!	1	#VALUE!
T11HN4	T11	A11	0	0	kleiner LLOQ	#VALUE!	1	#VALUE!
T11HN4	T11	A12	0	0	kleiner LLOQ	#VALUE!	1	#VALUE!
0	T11	B01	0	0	kleiner LLOQ	#VALUE!	1	#VALUE!
0	T11	B02	0	0	kleiner LLOQ	#VALUE!	1	#VALUE!
0	T11	B03	0	0	kleiner LLOQ	#VALUE!	1	#VALUE!
0	T11	B04	0	0	kleiner LLOQ	#VALUE!	1	#VALUE!
T11HS11	T11	B05	0	0	kleiner LLOQ	#VALUE!	1	#VALUE!
T11HS11	T11	B06	0	0	kleiner LLOQ	#VALUE!	1	#VALUE!
T11HS12	T11	B07	0	0	kleiner LLOQ	#VALUE!	1	#VALUE!
T11HS12	T11	B08	0	0	kleiner LLOQ	#VALUE!	1	#VALUE!
T11HS13	T11	B09	0	0	kleiner LLOQ	#VALUE!	1	#VALUE!
T11HS13	T11	B10	0	0	kleiner LLOQ	#VALUE!	1	#VALUE!
T11HS14	T11	B11	0	0	kleiner LLOQ	#VALUE!	1	#VALUE!
T11HS14	T11	B12	0	0	kleiner LLOQ	#VALUE!	1	#VALUE!
0	T11	C01	0	0	kleiner LLOQ	#VALUE!	1	#VALUE!
0	T11	C02	0	0	kleiner LLOQ	#VALUE!	1	#VALUE!
0	T11	C03	0	0	kleiner LLOQ	#VALUE!	1	#VALUE!
0	T11	C04	0	0	kleiner LLOQ	#VALUE!	1	#VALUE!
T11HS21	T11	C05	0	0	kleiner LLOQ	#VALUE!	1	#VALUE!
T11HS21	T11	C06	0	0	kleiner LLOQ	#VALUE!	1	#VALUE!
T11HS22	T11	C07	0	0	kleiner LLOQ	#VALUE!	1	#VALUE!
T11HS22	T11	C08	0	0	kleiner LLOQ	#VALUE!	1	#VALUE!
T11HS23	T11	C09	0	0	kleiner LLOQ	#VALUE!	1	#VALUE!
T11HS23	T11	C10	0	0	kleiner LLOQ	#VALUE!	1	#VALUE!
T11HS24	T11	C11	0	0	kleiner LLOQ	#VALUE!	1	#VALUE!
T11HS24	T11	C12	0	0	kleiner LLOQ	#VALUE!	1	#VALUE!
0	T11	D01	0	0	kleiner LLOQ	#VALUE!	1	#VALUE!
0	T11	D02	0	0	kleiner LLOQ	#VALUE!	1	#VALUE!
0	T11	D03	0	0	kleiner LLOQ	#VALUE!	1	#VALUE!
0	T11	D04	0	0	kleiner LLOQ	#VALUE!	1	#VALUE!
0	T11	D05	0	0	kleiner LLOQ	#VALUE!	1	#VALUE!
0	T11	D06	0	0	kleiner LLOQ	#VALUE!	1	#VALUE!
0	T11	D07	0	0	kleiner LLOQ	#VALUE!	1	#VALUE!
0	T11	D08	0	0	kleiner LLOQ	#VALUE!	1	#VALUE!
0	T11	D09	0	0	kleiner LLOQ	#VALUE!	1	#VALUE!
0	T11	D10	0	0	kleiner LLOQ	#VALUE!	1	#VALUE!
0	T11	D11	0	0	kleiner LLOQ	#VALUE!	1	#VALUE!
0	T11	D12	0	0	kleiner LLOQ	#VALUE!	1	#VALUE!
0	T11	E01	0	0	kleiner LLOQ	#VALUE!	1	#VALUE!
0	T11	E02	0	0	kleiner LLOQ	#VALUE!	1	#VALUE!
0	T11	E03	0	0	kleiner LLOQ	#VALUE!	1	#VALUE!
0	T11	E04	0	0	kleiner LLOQ	#VALUE!	1	#VALUE!
0	T11	E05	0	0	kleiner LLOQ	#VALUE!	1	#VALUE!
0	T11	E06	0	0	kleiner LLOQ	#VALUE!	1	#VALUE!
0	T11	E07	0	0	kleiner LLOQ	#VALUE!	1	#VALUE!
0	T11	E08	0	0	kleiner LLOQ	#VALUE!	1	#VALUE!
0	T11	E09	0	0	kleiner LLOQ	#VALUE!	1	#VALUE!

Plate: T11

	1	2	3	4	5	6	7	8	9	10	11	12
A												
B												
C												
D												
E												
F												
G							0.052	0.052	0.052	0.056	0.052	0.052
H							0.050	0.050	0.050	0.050	0.050	0.049

Plattenbelegung

	1	2	3	4	5	6	7	8	9	10	11	12
A					T11HN1	T11HN1	T11HN2	T11HN2	T11HN3	T11HN3	T11HN4	T11HN4
B					T11HS11	T11HS11	T11HS12	T11HS12	T11HS13	T11HS13	T11HS14	T11HS14
C					T11HS21	T11HS21	T11HS22	T11HS22	T11HS23	T11HS23	T11HS24	T11HS24
D												
E												
F												
G							Blank PEG	Blank PEG	Blank PEG	Blank PEG	Blank PEG	Blank PEG
H							Blank no PEG	Blank no PEG	Blank no PEG	Blank no PEG	Blank no PEG	Blank no PEG

Comment:

alle Messwerte bei lin low.

Date Operator

Date Control

0	T11	E10	0	0	kleiner LLOQ	#VALUE!	1	#VALUE!
0	T11	E11	0	0	kleiner LLOQ	#VALUE!	1	#VALUE!
0	T11	E12	0	0	kleiner LLOQ	#VALUE!	1	#VALUE!
0	T11	F01	0	0	kleiner LLOQ	#VALUE!	1	#VALUE!
0	T11	F02	0	0	kleiner LLOQ	#VALUE!	1	#VALUE!
0	T11	F03	0	0	kleiner LLOQ	#VALUE!	1	#VALUE!
0	T11	F04	0	0	kleiner LLOQ	#VALUE!	1	#VALUE!
0	T11	F05	0	0	kleiner LLOQ	#VALUE!	1	#VALUE!
0	T11	F06	0	0	kleiner LLOQ	#VALUE!	1	#VALUE!
0	T11	F07	0	0	kleiner LLOQ	#VALUE!	1	#VALUE!
0	T11	F08	0	0	kleiner LLOQ	#VALUE!	1	#VALUE!
0	T11	F09	0	0	kleiner LLOQ	#VALUE!	1	#VALUE!
0	T11	F10	0	0	kleiner LLOQ	#VALUE!	1	#VALUE!
0	T11	F11	0	0	kleiner LLOQ	#VALUE!	1	#VALUE!
0	T11	F12	0	0	kleiner LLOQ	#VALUE!	1	#VALUE!
0	T11	G01	0	0	kleiner LLOQ	#VALUE!	1	#VALUE!
0	T11	G02	0	0	kleiner LLOQ	#VALUE!	1	#VALUE!
0	T11	G03	0	0	kleiner LLOQ	#VALUE!	1	#VALUE!
0	T11	G04	0	0	kleiner LLOQ	#VALUE!	1	#VALUE!
0	T11	G05	0	0	kleiner LLOQ	#VALUE!	1	#VALUE!
0	T11	G06	0	0	kleiner LLOQ	#VALUE!	1	#VALUE!
Blank PEG	T11	G07	0	0	kleiner LLOQ	#VALUE!	1	#VALUE!
Blank PEG	T11	G08	0	0	kleiner LLOQ	#VALUE!	1	#VALUE!
Blank PEG	T11	G09	0	0	kleiner LLOQ	#VALUE!	1	#VALUE!
Blank PEG	T11	G10	0	0	kleiner LLOQ	#VALUE!	1	#VALUE!
Blank PEG	T11	G11	0	0	kleiner LLOQ	#VALUE!	1	#VALUE!
Blank PEG	T11	G12	0	0	kleiner LLOQ	#VALUE!	1	#VALUE!
0	T11	H01	0	0	kleiner LLOQ	#VALUE!	1	#VALUE!
0	T11	H02	0	0	kleiner LLOQ	#VALUE!	1	#VALUE!
0	T11	H03	0	0	kleiner LLOQ	#VALUE!	1	#VALUE!
0	T11	H04	0	0	kleiner LLOQ	#VALUE!	1	#VALUE!
0	T11	H05	0	0	kleiner LLOQ	#VALUE!	1	#VALUE!
0	T11	H06	0	0	kleiner LLOQ	#VALUE!	1	#VALUE!
Blank no PEG	T11	H07	0	0	kleiner LLOQ	#VALUE!	1	#VALUE!
Blank no PEG	T11	H08	0	0	kleiner LLOQ	#VALUE!	1	#VALUE!
Blank no PEG	T11	H09	0	0	kleiner LLOQ	#VALUE!	1	#VALUE!
Blank no PEG	T11	H10	0	0	kleiner LLOQ	#VALUE!	1	#VALUE!
Blank no PEG	T11	H11	0	0	kleiner LLOQ	#VALUE!	1	#VALUE!
Blank no PEG	T11	H12	0	0	kleiner LLOQ	#VALUE!	1	#VALUE!

1.447	1.739		1.170						0.667	0.440	0.043	1
1.447	1.332		1.392	0.137	0.146	0.130	0.138	0.109	0.729	0.478	0.042	
1.391	1.281		1.311	0.204	0.184	0.184	0.198	0.201	0.743	0.474	0.043	
0.961	0.835		1.405	0.336	0.347	0.375	0.387	0.300	0.824	0.483	0.044	
0.445	0.441		1.280	1.085	1.086	1.218	1.004	0.873	0.704	0.457	0.041	
0.355	0.315		1.332	1.768	1.925	1.753	1.739	1.606			0.036	
0.223	0.188	1.666	1.499	1.632		0.048	0.046	0.047	0.048	0.048	0.048	
0.118	0.106	1.556	1.622	1.573		0.047	0.054	0.051	0.051	0.052	0.051	

1.652	1.627		1.188	0.748	0.811	0.764	0.753	0.674	0.751	0.529	0.044	2
1.443	1.372		1.364	0.139	0.147	0.131	0.139	0.110	0.811	0.561	0.043	
1.377	1.281		1.284	0.206	0.189	0.184	0.200	0.202	0.821	0.560	0.044	
0.972	0.883		1.387	0.339	0.349	0.386	0.406	0.312	0.903	0.565	0.044	
0.484	0.474		1.298	1.087	1.113	1.203	1.022	0.903	0.790	0.544	0.042	
0.359	0.326		1.308	1.622	1.622	1.630	1.625	1.550			0.037	
0.228	0.199	1.527	1.373	1.462		0.048	0.046	0.048	0.049	0.049	0.049	
0.125	0.109	1.473	1.410	1.371		0.048	0.054	0.052	0.052	0.052	0.051	

1.579	1.475		1.150	1.210	1.303	1.202	1.219	1.125	0.759	0.549	0.043	3
1.357	1.303		1.283	0.140	0.148	0.131	0.139	0.111	0.820	0.575	0.043	
1.323	1.228		1.227	0.206	0.188	0.184	0.198	0.201	0.824	0.579	0.043	
0.959	0.872		1.321	0.339	0.348	0.387	0.406	0.314	0.909	0.580	0.044	
0.483	0.473		1.270	1.069	1.103	1.181	1.017	0.903	0.798	0.560	0.042	
0.360	0.326		1.270	1.497	1.448	1.496	1.494	1.449			0.037	
0.228	0.199	1.374	1.262	1.314		0.048	0.046	0.048	0.049	0.049	0.049	
0.125	0.110	1.346	1.262	1.253		0.048	0.054	0.052	0.052	0.052	0.051	

1.511	1.369			1.538	1.712	1.528	1.502	1.425			0.043	4
1.296	1.261										0.043	
1.272	1.193										0.043	
0.947	0.859										0.044	
0.481	0.471										0.042	
0.361	0.325										0.037	
0.229	0.199					0.048	0.046	0.048	0.049	0.049	0.049	
0.125	0.110					0.047	0.054	0.052	0.052	0.052	0.051	

1.493	1.317	1.678	1.110	1.621	1.807	1.618	1.584	1.508	0.765	0.597	0.043	5
1.259	1.238	1.850	1.197	0.139	0.150	0.132	0.139	0.111	0.821	0.588	0.043	
1.248	1.175	1.816	1.153	0.204	0.188	0.184	0.197	0.199	0.831	0.599	0.044	
0.943	0.859	1.970	1.231	0.338	0.357	0.386	0.406	0.316	0.916	0.595	0.044	
0.480	0.470	2.220	1.220	1.054	1.088	1.154	1.006	0.902	0.805	0.579	0.042	
0.359	0.325	2.149	1.211	1.395	1.324	1.348	1.345	1.317	0.037	0.037	0.037	
0.228	0.199	1.240	1.151	1.183	0.034	0.049	0.046	0.048	0.049	0.049	0.049	
0.125	0.110	1.223	1.132	1.146	0.038	0.048	0.054	0.052	0.052	0.052	0.051	

1.434	1.258		1.090	1.648	1.876	1.649	1.627	1.535				6
1.217	1.201		1.149									
1.205	1.143		1.109									
0.935	0.845		1.182									
0.477	0.469		1.181									
0.360	0.323		1.167									
0.229	0.200					0.049	0.047	0.048	0.049	0.049	0.049	
0.125	0.110					0.048	0.054	0.052	0.052	0.052	0.051	

1.013	0.810		0.668	0.808	0.940	0.720	0.798	0.687	0.904	0.845	0.043	7
0.709	0.827		0.703	0.142	0.156	0.136	0.137	0.116	0.990	0.846	0.042	
0.682	0.692		0.680	0.197	0.188	0.190	0.191	0.184	1.023	0.861	0.043	
0.507	0.519		0.740	0.316	0.264	0.364	0.377	0.312	1.111	0.870	0.043	
0.394	0.406		0.682	0.580	0.561	0.600	0.529	0.563	0.962	0.834	0.042	
0.333	0.322		0.661	0.995	0.930	0.848	0.832	0.797	0.037	0.037	0.037	
0.229	0.201	0.843	0.679	0.732		0.048	0.046	0.047			0.047	
0.124	0.111	0.773	0.682	0.701		0.047	0.053	0.051	0.051	0.052	0.051	

skin cultivation

0.685	0.649	0.387	0.342									T8 1 hum sui
0.154	0.141	0.128	0.091									
0.558	0.521	0.531	0.224									
	0.277	0.599	0.442									
0.305	0.254	0.135	0.201									
0.266	0.234	0.279	0.262									
						0.051	0.054	0.050	0.052	0.051	0.051	
						0.051	0.051	0.050	0.050	0.051	0.050	

				0.635	0.607	0.628	0.661	0.404	0.309	0.229	0.222	T8 2 hum sui
				0.460	0.171	0.156	0.167	0.129	0.136	0.081	0.084	
				0.458	0.708	0.553	0.584	0.670	0.659	0.222	0.224	
						0.262	0.241	0.621	0.621	0.449	0.458	
				0.260	0.234	0.197	0.193	0.128	0.132	0.204	0.210	
				0.245	0.247	0.151	0.146	0.271	0.265	0.268	0.253	
						0.053	0.055	0.053	0.054	0.053	0.053	
						0.053	0.052	0.051	0.052	0.052	0.052	

0.488	0.481	0.313	0.358									T9 1 hum sui
0.137	0.134	0.176	0.097									
0.473	0.445	0.459	0.179									
	0.323	0.536	0.588									
0.374	0.244	0.122	0.203									
0.333	0.253	0.304	0.282									
						0.049	0.049	0.049	0.050	0.049	0.049	
						0.050	0.048	0.048	0.048	0.047	0.048	

				0.379	0.370	0.484	0.478	0.220	0.291	0.404	0.398	T9 2 hum sui
				0.157	0.155	0.155	0.157	0.194	0.200	0.080	0.091	
				0.623	0.633	0.542	0.548	0.625	0.640	0.197	0.192	
						0.331	0.343	0.621	0.661	0.691	0.644	
				0.415	0.405	0.204	0.207			0.233	0.231	
				0.295	0.327	0.199	0.193	0.277	0.307	0.271	0.291	
						0.051	0.051	0.051	0.051	0.051	0.050	
						0.050	0.049	0.050	0.049	0.050	0.050	

												T9 hum sui 2 wells vergessen
								0.138	0.133			
						0.051	0.051	0.051	0.051	0.051	0.050	
						0.050	0.049	0.050	0.050	0.050	0.050	

0.349	0.421	0.408	0.372									T10 1 hum sui
0.125	0.140	0.183	0.089									
0.538	0.449	0.477	0.178									
	0.486	0.476	0.666									
0.367	0.243		0.213									
0.431	0.273	0.263	0.314									
						0.049	0.049	0.049	0.051	0.049	0.052	
						0.049	0.048	0.052	0.050	0.049	0.049	

				0.337	0.335	0.457	0.454	0.421	0.439	0.367	0.373	T10 2 hum sui
				0.137	0.138	0.156	0.166	0.178	0.182	0.087	0.082	
				0.645	0.646	0.519	0.550	0.557	0.572	0.193	0.182	
						0.493	0.502	0.540	0.537	0.680	0.722	
				0.377	0.373	0.217	0.208			0.232	0.233	

				0.367	0.392	0.213	0.195	0.226	0.257	0.308	0.408
						0.051	0.051	0.051	0.053	0.051	0.054
						0.050	0.050	0.052	0.051	0.050	0.050

0.491	0.574	0.518	0.380									T11 1 hum sui
0.074	0.073	0.106	0.089									
0.689	0.516	0.535	0.209									
	0.352	0.464	0.727									
0.433	0.269	0.165	0.224									
0.402	0.254	0.296	0.329									
						0.052	0.051	0.051	0.053	0.051	0.051	
						0.049	0.049	0.050	0.050	0.049	0.049	

				0.502	0.545	0.613	0.593	0.579	0.537	0.385	0.388	T11 2 hum sui
				0.206	0.205	0.187	0.189	0.187	0.177	0.122	0.114	
				0.731	0.737	0.575	0.570	0.538	0.531	0.208	0.207	
						0.358	0.336	0.471	0.480	0.747	0.716	
				0.423	0.402	0.227	0.267	0.154	0.156	0.234	0.235	
				0.354	0.398	0.217	0.229	0.248	0.272	0.308	0.317	
						0.052	0.052	0.052	0.056	0.052	0.052	
						0.050	0.050	0.050	0.050	0.050	0.049	

**Project number**		F-120	**Apparatus**	**Wallac Victor**	**Operator**	**IsBa**
**GLP Study (Number)**		n.a.	**Protocol (Instrument method)**	**LDH test 2016**	**Date of preparation**	**19-04-18**
**hot substance**	**isotope**	n.a.	**File name (results)**	**IsBa_180419/20_LDH_full_va**	**Date of measurement**	**19-04-18**
	**name**	n.a.	**Kind of well plate**	**96 well**	**shaking time [min]**	**30**
	**ACB-ID**	n.a.	**sample volume [µL]**	**100**	**stirring rate (Target) [rpm]**	**150**
	**Batch number**	n.a.	**Cocktail volume [µL]**	**175**	**Kind of measurement**	**UV-vis**
**cold substance**	**name**	LDH	**ACB-ID of cocktail**		**Wave length [nm]**	**450**
	**ACB-ID**	L23982R	**Matrix**	**DMEM (from powder)+PEG**	**Remarks**	**Cocktail 100µl RM, 75µl STOP**
	**Batch number**	SLBK6345V	**Blank description**	**DMEM/PEG, H2O**	**Remarks**	**7 standards split low/high**
**n.a.**		n.a.	**Pipettes (No. / volume)**	**50-200µl**	**Remarks**	**KLP4 common for both**
**n.a.**		n.a.	**Pipettes (No. / volume)**	**n.a.**	**Remarks**	**n.a.**

Messdaten (diese Tabelle in Bericht übernehmen)

Sample name *	concentration (theor.) *	measured data	measured data	measured data	mean measured	SD	RSD	Blank *	measured data after * Blank subtraction	concentration (calc.) *	Deviation *	Residuen
	[µg/mL]	[AU]	[AU]	[AU]	[AU]	[AU]	[%]	[AU]		[µg/mL]	[%]	
KLP1								0.047				
KLP2								0.054				
KLP3								0.051				
KLP4	0.041	0.959	0.972	0.883	0.938	0.04	4.19	0.051	0.887	0.041	-0.57	0.00
KLP5	0.018	0.483	0.484	0.474	0.480	0.00	0.98	0.052	0.429	0.019	2.42	0.00
KLP6	0.012	0.360	0.359	0.326	0.348	0.02	4.54	0.051	0.297	0.013	5.00	0.00
KLP7	0.007	0.228	0.228	0.199	0.218	0.014	6.35		0.168	0.006	-11.29	0.00
KLP8												

Statistical data

Geradensteigung	Slope	m	21.03
Y-Achsenabschnitt	Y-Intercept	b	0.03
Standardabw. Geradensteigung	SD-Slope	s_m	0.648055501
Standardabw. Achsenabschnittes	SD-Y-Intercept	s_b	0.015177452
Anzahl Messpunkte	number of measuring points	n	4
Quadratsumme	sum of squares	Qxx	0.000664048
Bereichsmitte			0.019557193
Freiheitsgrade	degree of freedom	f	2
Student-t-Faktor für (P = 95 %; f = n-2)	Student-t-factor for (P = 95 %; f = n-2)	t	4.303
Vertrauensbereich Steig. (95 %) Obergrenze		$m + VB_m$	23.81763508
Vertrauensbereich Steig. (95 %) Untergrenze		$m - VB_m$	18.24046944
Vertrauensbereich Achsenabschnitt (95 %) Og.		$b + Vb_b$	0.099335829
Vertrauensbereich Achsenabschnitt (95 %) Ug.		$b - Vb_b$	-0.031281327
Korrelationskoeffizient	correlation coefficient	r	0.9991
Bestimmtheitsmaß	determiantion coefficient	r^2	0.9981
Reststandardabweichung		s_0	0.01669983
Summe Restquadrate		sd	0.657385838
Verfahrensstandardabw.		s_{x0}	0.000794131
Rel. Verfahrensstandardabw. %		V_{x0}	4.060559029

Berichten

mean Blank	**0**	
SD Blank	0.00	
RSD Blank	3.79	x=
x*SD (LLOQ)	0.01	5
x*SD (LOD)	0.01	3
**LLOQ (AU)**	**0.061**	
**LOD (AU)**	**0.057**	
ULOQ	0.887	
LLOQ (Lin)	0.168	

Evaluation / Comment

LDH linearity valid with 7 standards, split in lin high and lin low, each with 4 standards and KLP4 common standard for both. R² 0,9981, deviations for both between -13.34% and +13.04%

Date | Operator | Date | Control

Figure 1 Linearity

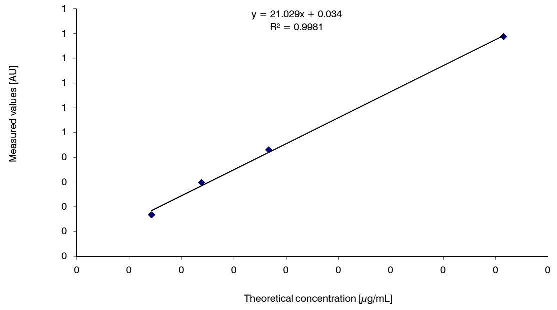

Figure 2 Method validation Residuen Plot

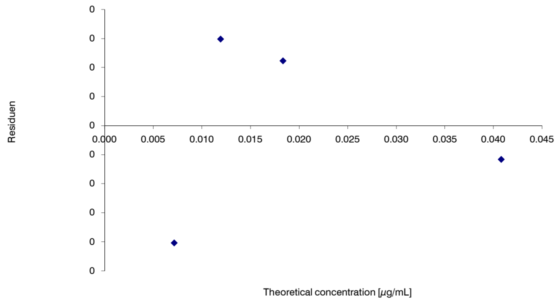

Evaluation / Comment

lin passed

Date	Operator	Date	Control

Sample name	Plate	Position	[AU]	[AU]-Blank	Rating	[µg/mL]	Dilution factor	[µg/mL]
0	T12	A01	0	0	kleiner LLOQ	#VALUE!	1	#VALUE!
0	T12	A02	0	0	kleiner LLOQ	#VALUE!	1	#VALUE!
0	T12	A03	0	0	kleiner LLOQ	#VALUE!	1	#VALUE!
0	T12	A04	0	0	kleiner LLOQ	#VALUE!	1	#VALUE!
T12HN1	T12	A05	0.519	0.468	0.468	0.021	2.75	0.057
T12HN1	T12	A06	0.533	0.482	0.482	0.021	2.75	0.059
T12HN2	T12	A07	0.518	0.467	0.467	0.021	2.75	0.057
T12HN2	T12	A08	0.519	0.468	0.468	0.021	2.75	0.057
T12HN3	T12	A09	0.484	0.433	0.433	0.019	2.75	0.052
T12HN3	T12	A10	0.513	0.462	0.462	0.020	2.75	0.056
T12HN4	T12	A11	0.362	0.311	0.311	0.013	2.75	0.036
T12HN4	T12	A12	0.354	0.303	0.303	0.013	2.75	0.035
0	T12	B01	0.000	-0.051	kleiner LLOQ	#VALUE!	1	#VALUE!
0	T12	B02	0.000	-0.051	kleiner LLOQ	#VALUE!	1	#VALUE!
0	T12	B03	0.000	-0.051	kleiner LLOQ	#VALUE!	1	#VALUE!
0	T12	B04	0.000	-0.051	kleiner LLOQ	#VALUE!	1	#VALUE!
T12HS11	T12	B05	0.206	0.156	kleiner LLOQ	#VALUE!	2.75	#VALUE!
T12HS11	T12	B06	0.207	0.156	kleiner LLOQ	#VALUE!	2.75	#VALUE!
T12HS12	T12	B07	0.164	0.113	kleiner LLOQ	#VALUE!	2.75	#VALUE!
T12HS12	T12	B08	0.166	0.115	kleiner LLOQ	#VALUE!	2.75	#VALUE!
T12HS13	T12	B09	0.196	0.145	kleiner LLOQ	#VALUE!	2.75	#VALUE!
T12HS13	T12	B10	0.182	0.132	kleiner LLOQ	#VALUE!	2.75	#VALUE!
T12HS14	T12	B11	0.199	0.148	kleiner LLOQ	#VALUE!	2.75	#VALUE!
T12HS14	T12	B12	0.199	0.148	kleiner LLOQ	#VALUE!	2.75	#VALUE!
0	T12	C01	0.000	-0.051	kleiner LLOQ	#VALUE!	1	#VALUE!
0	T12	C02	0.000	-0.051	kleiner LLOQ	#VALUE!	1	#VALUE!
0	T12	C03	0.000	-0.051	kleiner LLOQ	#VALUE!	1	#VALUE!
0	T12	C04	0.000	-0.051	kleiner LLOQ	#VALUE!	1	#VALUE!
T12HS21	T12	C05	0.835	0.784	0.784	0.036	2.75	0.098
T12HS21	T12	C06	0.831	0.780	0.780	0.035	2.75	0.098
T12HS22	T12	C07	0.740	0.689	0.689	0.031	2.75	0.086
T12HS22	T12	C08	0.760	0.709	0.709	0.032	2.75	0.088
T12HS23	T12	C09	0.624	0.573	0.573	0.026	2.75	0.070
T12HS23	T12	C10	0.633	0.582	0.582	0.026	2.75	0.072
T12HS24	T12	C11	0.245	0.194	0.194	0.008	2.75	0.021
T12HS24	T12	C12	0.234	0.183	0.183	0.007	2.75	0.020
0	T12	D01	0.000	-0.051	kleiner LLOQ	#VALUE!	1	#VALUE!
0	T12	D02	0.000	-0.051	kleiner LLOQ	#VALUE!	1	#VALUE!
0	T12	D03	0.000	-0.051	kleiner LLOQ	#VALUE!	1	#VALUE!
0	T12	D04	0.000	-0.051	kleiner LLOQ	#VALUE!	1	#VALUE!
0	T12	D05	0.000	-0.051	kleiner LLOQ	#VALUE!	1	#VALUE!
0	T12	D06	0.000	-0.051	kleiner LLOQ	#VALUE!	1	#VALUE!
0	T12	D07	0	0	kleiner LLOQ	#VALUE!	1	#VALUE!
0	T12	D08	0	0	kleiner LLOQ	#VALUE!	1	#VALUE!
0	T12	D09	0	0	kleiner LLOQ	#VALUE!	1	#VALUE!
0	T12	D10	0	0	kleiner LLOQ	#VALUE!	1	#VALUE!
0	T12	D11	0	0	kleiner LLOQ	#VALUE!	1	#VALUE!
0	T12	D12	0	0	kleiner LLOQ	#VALUE!	1	#VALUE!

Plate: T12

	Test 1	Test 2	Test 3	Test 4	5	6	7	8	9	10	11	12
A					0.5187	0.5327	0.5178	0.5189	0.4838	0.5125	0.3623	0.3541
B					0.2065	0.2068	0.1642	0.1658	0.1957	0.1825	0.1990	0.1987
C					0.8350	0.8308	0.7404	0.7598	0.6239	0.6333	0.2450	0.2342
D												
E												
F												
G							0.0490	0.0493	0.0495	0.0492	0.0492	0.0487
H							0.0475	0.0473	0.0474	0.0475	0.0475	0.0469

Plattenbelegung

	1	2	3	4	5	6	7	8	9	10	11	12
A					T12HN1	T12HN1	T12HN2	T12HN2	T12HN3	T12HN3	T12HN4	T12HN4
B					T12HS11	T12HS11	T12HS12	T12HS12	T12HS13	T12HS13	T12HS14	T12HS14
C					T12HS21	T12HS21	T12HS22	T12HS22	T12HS23	T12HS23	T12HS24	T12HS24
D												
E												
F												
G							Blank PEG	Blank PEG	Blank PEG	Blank PEG	Blank PEG	Blank PEG
H							Blank no PEG	Blank no PEG	Blank no PEG	Blank no PEG	Blank no PEG	Blank no PEG

Samples aufgeteilt in lin high und low:höher konzentrierte Samples bei lin high zu finden.

Comment:

Date Operator

Date Control

0	T12	E01	0	0	kleiner LLOQ	#VALUE!	1	#VALUE!
0	T12	E02	0	0	kleiner LLOQ	#VALUE!	1	#VALUE!
0	T12	E03	0	0	kleiner LLOQ	#VALUE!	1	#VALUE!
0	T12	E04	0	0	kleiner LLOQ	#VALUE!	1	#VALUE!
0	T12	E05	0	0	kleiner LLOQ	#VALUE!	1	#VALUE!
0	T12	E06	0	0	kleiner LLOQ	#VALUE!	1	#VALUE!
0	T12	E07	0	0	kleiner LLOQ	#VALUE!	1	#VALUE!
0	T12	E08	0	0	kleiner LLOQ	#VALUE!	1	#VALUE!
0	T12	E09	0	0	kleiner LLOQ	#VALUE!	1	#VALUE!
0	T12	E10	0	0	kleiner LLOQ	#VALUE!	1	#VALUE!
0	T12	E11	0	0	kleiner LLOQ	#VALUE!	1	#VALUE!
0	T12	E12	0	0	kleiner LLOQ	#VALUE!	1	#VALUE!
0	T12	F01	0	0	kleiner LLOQ	#VALUE!	1	#VALUE!
0	T12	F02	0	0	kleiner LLOQ	#VALUE!	1	#VALUE!
0	T12	F03	0	0	kleiner LLOQ	#VALUE!	1	#VALUE!
0	T12	F04	0	0	kleiner LLOQ	#VALUE!	1	#VALUE!
0	T12	F05	0	0	kleiner LLOQ	#VALUE!	1	#VALUE!
0	T12	F06	0	0	kleiner LLOQ	#VALUE!	1	#VALUE!
0	T12	F07	0	0	kleiner LLOQ	#VALUE!	1	#VALUE!
0	T12	F08	0	0	kleiner LLOQ	#VALUE!	1	#VALUE!
0	T12	F09	0	0	kleiner LLOQ	#VALUE!	1	#VALUE!
0	T12	F10	0	0	kleiner LLOQ	#VALUE!	1	#VALUE!
0	T12	F11	0	0	kleiner LLOQ	#VALUE!	1	#VALUE!
0	T12	F12	0	0	kleiner LLOQ	#VALUE!	1	#VALUE!
0	T12	G01	0	0	kleiner LLOQ	#VALUE!	1	#VALUE!
0	T12	G02	0	0	kleiner LLOQ	#VALUE!	1	#VALUE!
0	T12	G03	0	0	kleiner LLOQ	#VALUE!	1	#VALUE!
0	T12	G04	0	0	kleiner LLOQ	#VALUE!	1	#VALUE!
0	T12	G05	0	0	kleiner LLOQ	#VALUE!	1	#VALUE!
0	T12	G06	0	0	kleiner LLOQ	#VALUE!	1	#VALUE!
Blank PEG	T12	G07	0	0	kleiner LLOQ	#VALUE!	2.75	#VALUE!
Blank PEG	T12	G08	0	0	kleiner LLOQ	#VALUE!	2.75	#VALUE!
Blank PEG	T12	G09	0	0	kleiner LLOQ	#VALUE!	2.75	#VALUE!
Blank PEG	T12	G10	0	0	kleiner LLOQ	#VALUE!	2.75	#VALUE!
Blank PEG	T12	G11	0	0	kleiner LLOQ	#VALUE!	2.75	#VALUE!
Blank PEG	T12	G12	0	0	kleiner LLOQ	#VALUE!	2.75	#VALUE!
0	T12	H01	0	0	kleiner LLOQ	#VALUE!	1	#VALUE!
0	T12	H02	0	0	kleiner LLOQ	#VALUE!	1	#VALUE!
0	T12	H03	0	0	kleiner LLOQ	#VALUE!	1	#VALUE!
0	T12	H04	0	0	kleiner LLOQ	#VALUE!	1	#VALUE!
0	T12	H05	0	0	kleiner LLOQ	#VALUE!	1	#VALUE!
0	T12	H06	0	0	kleiner LLOQ	#VALUE!	1	#VALUE!
Blank no PEG	T12	H07	0	0	kleiner LLOQ	#VALUE!	2.75	#VALUE!
Blank no PEG	T12	H08	0	0	kleiner LLOQ	#VALUE!	2.75	#VALUE!
Blank no PEG	T12	H09	0	0	kleiner LLOQ	#VALUE!	2.75	#VALUE!
Blank no PEG	T12	H10	0	0	kleiner LLOQ	#VALUE!	2.75	#VALUE!
Blank no PEG	T12	H11	0	0	kleiner LLOQ	#VALUE!	2.75	#VALUE!
Blank no PEG	T12	H12	0	0	kleiner LLOQ	#VALUE!	2.75	#VALUE!

0	T13	A01	0	0	kleiner LLOQ	#VALUE!	1	#VALUE!
0	T13	A02	0	0	kleiner LLOQ	#VALUE!	1	#VALUE!
0	T13	A03	0	0	kleiner LLOQ	#VALUE!	1	#VALUE!
0	T13	A04	0	0	kleiner LLOQ	#VALUE!	1	#VALUE!
T13HN1	T13	A05	0.454	0.403	0.403	0.018	2.75	0.048
T13HN1	T13	A06	0.424	0.373	0.373	0.016	2.75	0.044
T13HN2	T13	A07	0.350	0.299	0.299	0.013	2.75	0.035
T13HN2	T13	A08	0.386	0.335	0.335	0.014	2.75	0.039
T13HN3	T13	A09	0.444	0.393	0.393	0.017	2.75	0.047
T13HN3	T13	A10	0.434	0.383	0.383	0.017	2.75	0.046
T13HN4	T13	A11	0.382	0.331	0.331	0.014	2.75	0.039
T13HN4	T13	A12	0.382	0.332	0.332	0.014	2.75	0.039
0	T13	B01	0.000	-0.051	kleiner LLOQ	#VALUE!	1	#VALUE!
0	T13	B02	0.000	-0.051	kleiner LLOQ	#VALUE!	1	#VALUE!
0	T13	B03	0.000	-0.051	kleiner LLOQ	#VALUE!	1	#VALUE!
0	T13	B04	0.000	-0.051	kleiner LLOQ	#VALUE!	1	#VALUE!
T13HS11	T13	B05	0.158	0.107	kleiner LLOQ	#VALUE!	2.75	#VALUE!
T13HS11	T13	B06	0.201	0.150	kleiner LLOQ	#VALUE!	2.75	#VALUE!
T13HS12	T13	B07	0.183	0.132	kleiner LLOQ	#VALUE!	2.75	#VALUE!
T13HS12	T13	B08	0.182	0.131	kleiner LLOQ	#VALUE!	2.75	#VALUE!
T13HS13	T13	B09	0.159	0.108	kleiner LLOQ	#VALUE!	2.75	#VALUE!
T13HS13	T13	B10	0.156	0.105	kleiner LLOQ	#VALUE!	2.75	#VALUE!
T13HS14	T13	B11	0.184	0.133	kleiner LLOQ	#VALUE!	2.75	#VALUE!
T13HS14	T13	B12	0.190	0.139	kleiner LLOQ	#VALUE!	2.75	#VALUE!
0	T13	C01	0.000	-0.051	kleiner LLOQ	#VALUE!	1	#VALUE!
0	T13	C02	0.000	-0.051	kleiner LLOQ	#VALUE!	1	#VALUE!
0	T13	C03	0.000	-0.051	kleiner LLOQ	#VALUE!	1	#VALUE!
0	T13	C04	0.000	-0.051	kleiner LLOQ	#VALUE!	1	#VALUE!
T13HS21	T13	C05	0.933	0.882	0.882	0.040	2.75	0.111
T13HS21	T13	C06	0.873	0.822	0.822	0.037	2.75	0.103
T13HS22	T13	C07	0.744	0.694	0.694	0.031	2.75	0.086
T13HS22	T13	C08	0.758	0.707	0.707	0.032	2.75	0.088
T13HS23	T13	C09	0.539	0.488	0.488	0.022	2.75	0.059
T13HS23	T13	C10	0.581	0.530	0.530	0.024	2.75	0.065
T13HS24	T13	C11	0.256	0.205	0.205	0.008	2.75	0.022
T13HS24	T13	C12	0.289	0.238	0.238	0.010	2.75	0.027
0	T13	D01	0	0	kleiner LLOQ	#VALUE!	1	#VALUE!
0	T13	D02	0	0	kleiner LLOQ	#VALUE!	1	#VALUE!
0	T13	D03	0	0	kleiner LLOQ	#VALUE!	1	#VALUE!
0	T13	D04	0	0	kleiner LLOQ	#VALUE!	1	#VALUE!
0	T13	D05	0	0	kleiner LLOQ	#VALUE!	1	#VALUE!
0	T13	D06	0	0	kleiner LLOQ	#VALUE!	1	#VALUE!
0	T13	D07	0	0	kleiner LLOQ	#VALUE!	1	#VALUE!
0	T13	D08	0	0	kleiner LLOQ	#VALUE!	1	#VALUE!
0	T13	D09	0	0	kleiner LLOQ	#VALUE!	1	#VALUE!
0	T13	D10	0	0	kleiner LLOQ	#VALUE!	1	#VALUE!
0	T13	D11	0	0	kleiner LLOQ	#VALUE!	1	#VALUE!
0	T13	D12	0	0	kleiner LLOQ	#VALUE!	1	#VALUE!

Plate: T13

	1	2	3	4	5	6	7	8	9	10	11	12
A					0.454	0.424	0.350	0.386	0.444	0.434	0.382	0.382
B					0.158	0.201	0.183	0.182	0.159	0.156	0.184	0.190
C					0.933	0.873	0.744	0.758	0.539	0.581	0.256	0.289
D												
E												
F												
G							0.050	0.050	0.053	0.051	0.051	0.050
H							0.048	0.048	0.048	0.048	0.048	0.048

Plattenbelegung

	1	2	3	4	5	6	7	8	9	10	11	12
A					T13HN1	T13HN1	T13HN2	T13HN2	T13HN3	T13HN3	T13HN4	T13HN4
B					T13HS11	T13HS11	T13HS12	T13HS12	T13HS13	T13HS13	T13HS14	T13HS14
C					T13HS21	T13HS21	T13HS22	T13HS22	T13HS23	T13HS23	T13HS24	T13HS24
D												
E												
F												
G							Blank PEG	Blank PEG	Blank PEG	Blank PEG	Blank PEG	Blank PEG
H							Blank no PEG	Blank no PEG	Blank no PEG	Blank no PEG	Blank no PEG	Blank no PEG

Comment:

Date Operator

Date Control

0	T13	E01	0	0	kleiner LLOQ	#VALUE!	1	#VALUE!
0	T13	E02	0	0	kleiner LLOQ	#VALUE!	1	#VALUE!
0	T13	E03	0	0	kleiner LLOQ	#VALUE!	1	#VALUE!
0	T13	E04	0	0	kleiner LLOQ	#VALUE!	1	#VALUE!
0	T13	E05	0	0	kleiner LLOQ	#VALUE!	1	#VALUE!
0	T13	E06	0	0	kleiner LLOQ	#VALUE!	1	#VALUE!
0	T13	E07	0	0	kleiner LLOQ	#VALUE!	1	#VALUE!
0	T13	E08	0	0	kleiner LLOQ	#VALUE!	1	#VALUE!
0	T13	E09	0	0	kleiner LLOQ	#VALUE!	1	#VALUE!
0	T13	E10	0	0	kleiner LLOQ	#VALUE!	1	#VALUE!
0	T13	E11	0	0	kleiner LLOQ	#VALUE!	1	#VALUE!
0	T13	E12	0	0	kleiner LLOQ	#VALUE!	1	#VALUE!
0	T13	F01	0	0	kleiner LLOQ	#VALUE!	1	#VALUE!
0	T13	F02	0	0	kleiner LLOQ	#VALUE!	1	#VALUE!
0	T13	F03	0	0	kleiner LLOQ	#VALUE!	1	#VALUE!
0	T13	F04	0	0	kleiner LLOQ	#VALUE!	1	#VALUE!
0	T13	F05	0	0	kleiner LLOQ	#VALUE!	1	#VALUE!
0	T13	F06	0	0	kleiner LLOQ	#VALUE!	1	#VALUE!
0	T13	F07	0	0	kleiner LLOQ	#VALUE!	1	#VALUE!
0	T13	F08	0	0	kleiner LLOQ	#VALUE!	1	#VALUE!
0	T13	F09	0	0	kleiner LLOQ	#VALUE!	1	#VALUE!
0	T13	F10	0	0	kleiner LLOQ	#VALUE!	1	#VALUE!
0	T13	F11	0	0	kleiner LLOQ	#VALUE!	1	#VALUE!
0	T13	F12	0	0	kleiner LLOQ	#VALUE!	1	#VALUE!
0	T13	G01	0	0	kleiner LLOQ	#VALUE!	1	#VALUE!
0	T13	G02	0	0	kleiner LLOQ	#VALUE!	1	#VALUE!
0	T13	G03	0	0	kleiner LLOQ	#VALUE!	1	#VALUE!
0	T13	G04	0	0	kleiner LLOQ	#VALUE!	1	#VALUE!
0	T13	G05	0	0	kleiner LLOQ	#VALUE!	1	#VALUE!
0	T13	G06	0	0	kleiner LLOQ	#VALUE!	1	#VALUE!
Blank PEG	T13	G07	0	0	kleiner LLOQ	#VALUE!	2.75	#VALUE!
Blank PEG	T13	G08	0	0	kleiner LLOQ	#VALUE!	2.75	#VALUE!
Blank PEG	T13	G09	0	0	kleiner LLOQ	#VALUE!	2.75	#VALUE!
Blank PEG	T13	G10	0	0	kleiner LLOQ	#VALUE!	2.75	#VALUE!
Blank PEG	T13	G11	0	0	kleiner LLOQ	#VALUE!	2.75	#VALUE!
Blank PEG	T13	G12	0	0	kleiner LLOQ	#VALUE!	2.75	#VALUE!
0	T13	H01	0	0	kleiner LLOQ	#VALUE!	1	#VALUE!
0	T13	H02	0	0	kleiner LLOQ	#VALUE!	1	#VALUE!
0	T13	H03	0	0	kleiner LLOQ	#VALUE!	1	#VALUE!
0	T13	H04	0	0	kleiner LLOQ	#VALUE!	1	#VALUE!
0	T13	H05	0	0	kleiner LLOQ	#VALUE!	1	#VALUE!
0	T13	H06	0	0	kleiner LLOQ	#VALUE!	1	#VALUE!
Blank no PEG	T13	H07	0	0	kleiner LLOQ	#VALUE!	2.75	#VALUE!
Blank no PEG	T13	H08	0	0	kleiner LLOQ	#VALUE!	2.75	#VALUE!
Blank no PEG	T13	H09	0	0	kleiner LLOQ	#VALUE!	2.75	#VALUE!
Blank no PEG	T13	H10	0	0	kleiner LLOQ	#VALUE!	2.75	#VALUE!
Blank no PEG	T13	H11	0	0	kleiner LLOQ	#VALUE!	2.75	#VALUE!
Blank no PEG	T13	H12	0	0	kleiner LLOQ	#VALUE!	2.75	#VALUE!
0	T14	A01	0	0	kleiner LLOQ	#VALUE!	1	#VALUE!
0	T14	A02	0	0	kleiner LLOQ	#VALUE!	1	#VALUE!
0	T14	A03	0	0	kleiner LLOQ	#VALUE!	1	#VALUE!
0	T14	A04	0	0	kleiner LLOQ	#VALUE!	1	#VALUE!
T14HN1	T14	A05	0.308	0.257	0.257	0.011	2.75	0.029
T14HN1	T14	A06	0.290	0.239	0.239	0.010	2.75	0.027
T14HN2	T14	A07	0.352	0.301	0.301	0.013	2.75	0.035
T14HN2	T14	A08	0.377	0.327	0.327	0.014	2.75	0.038
T14HN3	T14	A09	0.386	0.335	0.335	0.014	2.75	0.039
T14HN3	T14	A10	0.406	0.355	0.355	0.015	2.75	0.042
T14HN4	T14	A11	0.362	0.311	0.311	0.013	2.75	0.036
T14HN4	T14	A12	0.392	0.341	0.341	0.015	2.75	0.040
0	T14	B01	0.000	-0.051	kleiner LLOQ	#VALUE!	1	#VALUE!
0	T14	B02	0.000	-0.051	kleiner LLOQ	#VALUE!	1	#VALUE!
0	T14	B03	0.000	-0.051	kleiner LLOQ	#VALUE!	1	#VALUE!
0	T14	B04	0.000	-0.051	kleiner LLOQ	#VALUE!	1	#VALUE!
T14HS11	T14	B05	0.183	0.132	kleiner LLOQ	#VALUE!	2.75	#VALUE!
T14HS11	T14	B06	0.180	0.129	kleiner LLOQ	#VALUE!	2.75	#VALUE!
T14HS12	T14	B07	0.174	0.124	kleiner LLOQ	#VALUE!	2.75	#VALUE!
T14HS12	T14	B08	0.178	0.127	kleiner LLOQ	#VALUE!	2.75	#VALUE!
T14HS13	T14	B09	0.170	0.119	kleiner LLOQ	#VALUE!	2.75	#VALUE!
T14HS13	T14	B10	0.174	0.123	kleiner LLOQ	#VALUE!	2.75	#VALUE!
T14HS14	T14	B11	0.187	0.136	kleiner LLOQ	#VALUE!	2.75	#VALUE!
T14HS14	T14	B12	0.256	0.205	0.205	0.008	2.75	0.022
0	T14	C01	0.000	-0.051	kleiner LLOQ	#VALUE!	1	#VALUE!

Plate: T14

	1	2	3	4	5	6	7	8	9	10	11	12
A					0.308	0.290	0.352	0.377	0.386	0.406	0.362	0.392
B					0.183	0.180	0.174	0.178	0.170	0.174	0.187	0.256
C					0.667	0.663	0.572	0.566	0.541	0.553	0.416	0.389
D												
E												
F												
G							0.041	0.048	0.048	0.048	0.048	0.047
H							0.044	0.045	0.046	0.048	0.046	0.048

Plattenbelegung

	1	2	3	4	5	6	7	8	9	10	11	12
A					T14HN1	T14HN1	T14HN2	T14HN2	T14HN3	T14HN3	T14HN4	T14HN4
B					T14HS11	T14HS11	T14HS12	T14HS12	T14HS13	T14HS13	T14HS14	T14HS14
C					T14HS21	T14HS21	T14HS22	T14HS22	T14HS23	T14HS23	T14HS24	T14HS24
D												
E												

0	T14	C02	0.000	-0.051	kleiner LLOQ	#VALUE!	1	#VALUE!
0	T14	C03	0.000	-0.051	kleiner LLOQ	#VALUE!	1	#VALUE!
0	T14	C04	0.000	-0.051	kleiner LLOQ	#VALUE!	1	#VALUE!
T14HS21	T14	C05	0.667	0.616	0.616	0.028	2.75	0.076
T14HS21	T14	C06	0.663	0.612	0.612	0.027	2.75	0.076
T14HS22	T14	C07	0.572	0.521	0.521	0.023	2.75	0.064
T14HS22	T14	C08	0.566	0.515	0.515	0.023	2.75	0.063
T14HS23	T14	C09	0.541	0.490	0.490	0.022	2.75	0.060
T14HS23	T14	C10	0.553	0.502	0.502	0.022	2.75	0.061
T14HS24	T14	C11	0.416	0.365	0.365	0.016	2.75	0.043
T14HS24	T14	C12	0.389	0.338	0.338	0.014	2.75	0.040
0	T14	D01	0.000	-0.051	kleiner LLOQ	#VALUE!	1	#VALUE!
0	T14	D02	0.000	-0.051	kleiner LLOQ	#VALUE!	1	#VALUE!
0	T14	D03	0	0	kleiner LLOQ	#VALUE!	1	#VALUE!
0	T14	D04	0	0	kleiner LLOQ	#VALUE!	1	#VALUE!
0	T14	D05	0	0	kleiner LLOQ	#VALUE!	1	#VALUE!
0	T14	D06	0	0	kleiner LLOQ	#VALUE!	1	#VALUE!
0	T14	D07	0	0	kleiner LLOQ	#VALUE!	1	#VALUE!
0	T14	D08	0	0	kleiner LLOQ	#VALUE!	1	#VALUE!
0	T14	D09	0	0	kleiner LLOQ	#VALUE!	1	#VALUE!
0	T14	D10	0	0	kleiner LLOQ	#VALUE!	1	#VALUE!
0	T14	D11	0	0	kleiner LLOQ	#VALUE!	1	#VALUE!
0	T14	D12	0	0	kleiner LLOQ	#VALUE!	1	#VALUE!
0	T14	E01	0	0	kleiner LLOQ	#VALUE!	1	#VALUE!
0	T14	E02	0	0	kleiner LLOQ	#VALUE!	1	#VALUE!
0	T14	E03	0	0	kleiner LLOQ	#VALUE!	1	#VALUE!
0	T14	E04	0	0	kleiner LLOQ	#VALUE!	1	#VALUE!
0	T14	E05	0	0	kleiner LLOQ	#VALUE!	1	#VALUE!
0	T14	E06	0	0	kleiner LLOQ	#VALUE!	1	#VALUE!
0	T14	E07	0	0	kleiner LLOQ	#VALUE!	1	#VALUE!
0	T14	E08	0	0	kleiner LLOQ	#VALUE!	1	#VALUE!
0	T14	E09	0	0	kleiner LLOQ	#VALUE!	1	#VALUE!
0	T14	E10	0	0	kleiner LLOQ	#VALUE!	1	#VALUE!
0	T14	E11	0	0	kleiner LLOQ	#VALUE!	1	#VALUE!
0	T14	E12	0	0	kleiner LLOQ	#VALUE!	1	#VALUE!
0	T14	F01	0	0	kleiner LLOQ	#VALUE!	1	#VALUE!
0	T14	F02	0	0	kleiner LLOQ	#VALUE!	1	#VALUE!
0	T14	F03	0	0	kleiner LLOQ	#VALUE!	1	#VALUE!
0	T14	F04	0	0	kleiner LLOQ	#VALUE!	1	#VALUE!
0	T14	F05	0	0	kleiner LLOQ	#VALUE!	1	#VALUE!
0	T14	F06	0	0	kleiner LLOQ	#VALUE!	1	#VALUE!
0	T14	F07	0	0	kleiner LLOQ	#VALUE!	1	#VALUE!
0	T14	F08	0	0	kleiner LLOQ	#VALUE!	1	#VALUE!
0	T14	F09	0	0	kleiner LLOQ	#VALUE!	1	#VALUE!
0	T14	F10	0	0	kleiner LLOQ	#VALUE!	1	#VALUE!
0	T14	F11	0	0	kleiner LLOQ	#VALUE!	1	#VALUE!
0	T14	F12	0	0	kleiner LLOQ	#VALUE!	1	#VALUE!
0	T14	G01	0	0	kleiner LLOQ	#VALUE!	1	#VALUE!
0	T14	G02	0	0	kleiner LLOQ	#VALUE!	1	#VALUE!
0	T14	G03	0	0	kleiner LLOQ	#VALUE!	1	#VALUE!
0	T14	G04	0	0	kleiner LLOQ	#VALUE!	1	#VALUE!
0	T14	G05	0	0	kleiner LLOQ	#VALUE!	1	#VALUE!
0	T14	G06	0	0	kleiner LLOQ	#VALUE!	1	#VALUE!
Blank PEG	T14	G07	0	0	kleiner LLOQ	#VALUE!	1	#VALUE!
Blank PEG	T14	G08	0	0	kleiner LLOQ	#VALUE!	1	#VALUE!
Blank PEG	T14	G09	0	0	kleiner LLOQ	#VALUE!	1	#VALUE!
Blank PEG	T14	G10	0	0	kleiner LLOQ	#VALUE!	1	#VALUE!
Blank PEG	T14	G11	0	0	kleiner LLOQ	#VALUE!	1	#VALUE!
Blank PEG	T14	G12	0	0	kleiner LLOQ	#VALUE!	1	#VALUE!
0	T14	H01	0	0	kleiner LLOQ	#VALUE!	1	#VALUE!
0	T14	H02	0	0	kleiner LLOQ	#VALUE!	1	#VALUE!
0	T14	H03	0	0	kleiner LLOQ	#VALUE!	1	#VALUE!
0	T14	H04	0	0	kleiner LLOQ	#VALUE!	1	#VALUE!
0	T14	H05	0	0	kleiner LLOQ	#VALUE!	1	#VALUE!
0	T14	H06	0	0	kleiner LLOQ	#VALUE!	1	#VALUE!
Blank no PEG	T14	H07	0	0	kleiner LLOQ	#VALUE!	1	#VALUE!
Blank no PEG	T14	H08	0	0	kleiner LLOQ	#VALUE!	1	#VALUE!
Blank no PEG	T14	H09	0	0	kleiner LLOQ	#VALUE!	1	#VALUE!
Blank no PEG	T14	H10	0	0	kleiner LLOQ	#VALUE!	1	#VALUE!
Blank no PEG	T14	H11	0	0	kleiner LLOQ	#VALUE!	1	#VALUE!
Blank no PEG	T14	H12	0	0	kleiner LLOQ	#VALUE!	1	#VALUE!

F												
G							Blank PEG	Blank PEG	Blank PEG	Blank PEG	Blank PEG	Blank PEG
H							Blank no PEG	Blank no PEG	Blank no PEG	Blank no PEG	Blank no PEG	Blank no PEG

Comment:

Date Operator

Date Control

**Project number**		F-120	**Apparatus**	**Wallac Victor**	**Operator**	**IsBa**
**GLP Study (Number)**		n.a.	**Protocol (Instrument meth**	**LDH test 2016**	**Date of preparation**	**19-04-18**
**hot substance**	**isotope**	n.a.	**File name (results)**	**IsBa_180419/20_LDH_full_**	**Date of measurement**	**19-04-18**
	**name**	n.a.	**Kind of well plate**	**96 well**	**shaking time [min]**	**30**
	**ACB-ID**	n.a.	**sample volume [µL]**	**100**	**stirring rate (Target) [rpm]**	**150**
	**Batch number**	n.a.	**Cocktail volume [µL]**	**175**	**Kind of measurement**	**UV-vis**
**cold substance**	**name**	LDH	**ACB-ID of cocktail**		**Wave length [nm]**	**450**
	**ACB-ID**	L23982R	**Matrix**	**DMEM (from powder)+PE(**	**Remarks**	**Cocktail 100µl RM, 75µl STOP**
	**Batch number**	SLBK6345V	**Blank description**	**DMEM/PEG, H2O**	**Remarks**	**7 standards split low/high**
**n.a.**		n.a.	**Pipettes (No. / volume)**	**50-200µl**	**Remarks**	**KLP4 common for both**
**n.a.**		n.a.	**Pipettes (No. / volume)**	**n.a.**	**Remarks**	**n.a.**

Messdaten (diese Tabelle in Bericht übernehmen)

Sample name *	concentration (theor.) *	measured data	measured data	measured data	mean measured	SD	RSD	Blank *	measured data after * Blank subtraction	concentration (calc.) *	Deviation *	Residuen
	[µg/mL]	[AU]	[AU]	[AU]	[AU]	[AU]	[%]	[AU]		[µg/mL]	[%]	
KLP1	0.148	1.579	1.652	1.627	1.62	0.03	1.87	0.047	1.568	0.148	-0.24	0.00
KLP2	0.114	1.357	1.443	1.372	1.39	0.04	2.70	0.054	1.340	0.110	-3.40	0.00
KLP3	0.074	1.272	1.193	1.228	1.23	0.03	2.63	0.051	1.180	0.084	13.04	0.01
KLP4	0.041	0.959	0.972	0.883	0.94	0.04	4.19	0.051	0.887	0.035	-13.32	-0.01
KLP5								0.052				
KLP6								0.051				
KLP7												
KLP8												

Statistical data

Geradensteigung	Slope	m	6.05
Y-Achsenabschnitt	Y-Intercept	b	0.67
Standardabw. Geradensteigung	SD-Slope	s_m	0.620291227
Standardabw. Achsenabschnittes	SD-Y-Intercept	s_b	0.063694294
Anzahl Messpunkte	number of measuring points	n	4
Quadratsumme	sum of squares	Qxx	0.006578604
Bereichsmitte			0.094336926
Freiheitsgrade	degree of freedom	f	2
Student-t-Faktor für (P = 95 %; f = n-2)	Student-t-factor for (P = 95 %; f = n-2)	t	4.303
Vertrauensbereich Steig. (95 %) Obergrenze		$m + VB_m$	8.722319527
Vertrauensbereich Steig. (95 %) Untergrenze		$m - VB_m$	3.384093228
Vertrauensbereich Achsenabschnitt (95 %) Og.		$b + Vb_b$	0.946914615
Vertrauensbereich Achsenabschnitt (95 %) Ug.		$b - Vb_b$	0.398761518
Korrelationskoeffizient	correlation coefficient	r	0.9897
Bestimmtheitsmaß	determiantion coefficient	r^2	0.9794
Reststandardabweichung		s_0	0.050310948
Summe Restquadrate		sd	2.895583345
Verfahrensstandardabw.		s_{x0}	0.008311454
Rel. Verfahrensstandardabw. %		V_{x0}	8.810393374

mean Blank	**0**	
SD Blank	0.00	
RSD Blank	3.79	x=
x*SD (LLOQ)	0	5
x*SD (LOD)	0	3
**LLOQ (AU)**	**0**	
**LOD (AU)**	**0**	
ULOQ	1.568	
LLOQ (Lin)	0.887	

Evaluation / Comment

LDH linearity valid with 7 standards, split in lin high and lin low, each with 4 standards and KLP4 common standard for both. R² 0,9981, deviations for both between -13.34% and +13.04%

Date | Operator | Date | Control

Figure 1 Linearity

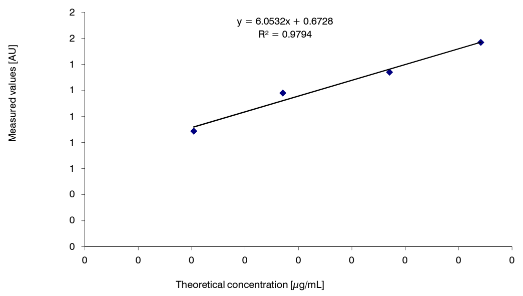

Figure 2 Method validation Residuen Plot

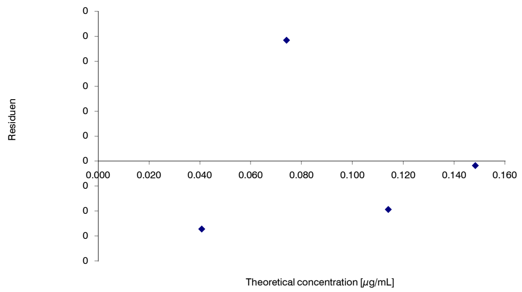

Evaluation / Comment

lin passed

Date	Operator	Date	Control

Sample name	Plate	Position	[AU]	[AU]-Blank	Rating	[µg/mL]	Dilution factor	[µg/mL]
0	T12	A01	0	0	kleiner LLOQ	#VALUE!	1	#VALUE!
0	T12	A02	0	0	kleiner LLOQ	#VALUE!	1	#VALUE!
0	T12	A03	0	0	kleiner LLOQ	#VALUE!	1	#VALUE!
0	T12	A04	0	0	kleiner LLOQ	#VALUE!	1	#VALUE!
0	T12	A05	0.000	-0.051	kleiner LLOQ	#VALUE!		#VALUE!
0	T12	A06	0.000	-0.051	kleiner LLOQ	#VALUE!		#VALUE!
0	T12	A07	0	0	kleiner LLOQ	#VALUE!		#VALUE!
0	T12	A08	0	0	kleiner LLOQ	#VALUE!		#VALUE!
0	T12	A09	0.000	-0.051	kleiner LLOQ	#VALUE!		#VALUE!
0	T12	A10	0.000	-0.051	kleiner LLOQ	#VALUE!		#VALUE!
0	T12	A11	0	0	kleiner LLOQ	#VALUE!		#VALUE!
0	T12	A12	0	0	kleiner LLOQ	#VALUE!		#VALUE!
0	T12	B01	0	0	kleiner LLOQ	#VALUE!		#VALUE!
0	T12	B02	0	0	kleiner LLOQ	#VALUE!		#VALUE!
0	T12	B03	0	0	kleiner LLOQ	#VALUE!		#VALUE!
0	T12	B04	0	0	kleiner LLOQ	#VALUE!		#VALUE!
0	T12	B05	0	0	kleiner LLOQ	#VALUE!		#VALUE!
0	T12	B06	0.000	-0.051	kleiner LLOQ	#VALUE!		#VALUE!
0	T12	B07	0.000	-0.051	kleiner LLOQ	#VALUE!		#VALUE!
0	T12	B08	0.000	-0.051	kleiner LLOQ	#VALUE!		#VALUE!
0	T12	B09	0	0	kleiner LLOQ	#VALUE!		#VALUE!
0	T12	B10	0	0	kleiner LLOQ	#VALUE!		#VALUE!
0	T12	B11	0	0	kleiner LLOQ	#VALUE!		#VALUE!
0	T12	B12	0	0	kleiner LLOQ	#VALUE!		#VALUE!
0	T12	C01	0	0	kleiner LLOQ	#VALUE!		#VALUE!
0	T12	C02	0	0	kleiner LLOQ	#VALUE!		#VALUE!
0	T12	C03	0	0	kleiner LLOQ	#VALUE!		#VALUE!
0	T12	C04	0	0	kleiner LLOQ	#VALUE!		#VALUE!
0	T12	C05	0.000	-0.051	kleiner LLOQ	#VALUE!		#VALUE!
0	T12	C06	0.000	-0.051	kleiner LLOQ	#VALUE!		#VALUE!
0	T12	C07	0.000	-0.051	kleiner LLOQ	#VALUE!		#VALUE!
0	T12	C08	0.000	-0.051	kleiner LLOQ	#VALUE!		#VALUE!
0	T12	C09	0.000	-0.051	kleiner LLOQ	#VALUE!		#VALUE!
0	T12	C10	0.000	-0.051	kleiner LLOQ	#VALUE!		#VALUE!
0	T12	C11	0	0	kleiner LLOQ	#VALUE!	1	#VALUE!
0	T12	C12	0	0	kleiner LLOQ	#VALUE!	1	#VALUE!
0	T12	D01	0	0	kleiner LLOQ	#VALUE!	1	#VALUE!
0	T12	D02	0	0	kleiner LLOQ	#VALUE!	1	#VALUE!
0	T12	D03	0	0	kleiner LLOQ	#VALUE!	1	#VALUE!
0	T12	D04	0	0	kleiner LLOQ	#VALUE!	1	#VALUE!
0	T12	D05	0	0	kleiner LLOQ	#VALUE!	1	#VALUE!
0	T12	D06	0	0	kleiner LLOQ	#VALUE!	1	#VALUE!
0	T12	D07	0	0	kleiner LLOQ	#VALUE!	1	#VALUE!
0	T12	D08	0	0	kleiner LLOQ	#VALUE!	1	#VALUE!
0	T12	D09	0	0	kleiner LLOQ	#VALUE!	1	#VALUE!
0	T12	D10	0	0	kleiner LLOQ	#VALUE!	1	#VALUE!
0	T12	D11	0	0	kleiner LLOQ	#VALUE!	1	#VALUE!
0	T12	D12	0	0	kleiner LLOQ	#VALUE!	1	#VALUE!

Plate: T12

	1	2	3	4	5	6	7	8	9	10	11	12
A												
B												
C												
D												
E												
F												
G												
H												

Plattenbelegung

	1	2	3	4	5	6	7	8	9	10	11	12
A												
B												
C												
D												
E												
F												
G							Blank PEG	Blank PEG	Blank PEG	Blank PEG	Blank PEG	Blank PEG
H							Blank no PEG	Blank no PEG	Blank no PEG	Blank no PEG	Blank no PEG	Blank no PEG

Comment:

alle Messwerte bei lin low

Samples aufgeteilt in lin high und low:niedriger konzentrierte Samples bei lin low zu finden.

Date Operator

Date Control

0	T12	E01	0	0	kleiner LLOQ	#VALUE!	1	#VALUE!
0	T12	E02	0	0	kleiner LLOQ	#VALUE!	1	#VALUE!
0	T12	E03	0	0	kleiner LLOQ	#VALUE!	1	#VALUE!
0	T12	E04	0	0	kleiner LLOQ	#VALUE!	1	#VALUE!
0	T12	E05	0	0	kleiner LLOQ	#VALUE!	1	#VALUE!
0	T12	E06	0	0	kleiner LLOQ	#VALUE!	1	#VALUE!
0	T12	E07	0	0	kleiner LLOQ	#VALUE!	1	#VALUE!
0	T12	E08	0	0	kleiner LLOQ	#VALUE!	1	#VALUE!
0	T12	E09	0	0	kleiner LLOQ	#VALUE!	1	#VALUE!
0	T12	E10	0	0	kleiner LLOQ	#VALUE!	1	#VALUE!
0	T12	E11	0	0	kleiner LLOQ	#VALUE!	1	#VALUE!
0	T12	E12	0	0	kleiner LLOQ	#VALUE!	1	#VALUE!
0	T12	F01	0	0	kleiner LLOQ	#VALUE!	1	#VALUE!
0	T12	F02	0	0	kleiner LLOQ	#VALUE!	1	#VALUE!
0	T12	F03	0	0	kleiner LLOQ	#VALUE!	1	#VALUE!
0	T12	F04	0	0	kleiner LLOQ	#VALUE!	1	#VALUE!
0	T12	F05	0	0	kleiner LLOQ	#VALUE!	1	#VALUE!
0	T12	F06	0	0	kleiner LLOQ	#VALUE!	1	#VALUE!
0	T12	F07	0	0	kleiner LLOQ	#VALUE!	1	#VALUE!
0	T12	F08	0	0	kleiner LLOQ	#VALUE!	1	#VALUE!
0	T12	F09	0	0	kleiner LLOQ	#VALUE!	1	#VALUE!
0	T12	F10	0	0	kleiner LLOQ	#VALUE!	1	#VALUE!
0	T12	F11	0	0	kleiner LLOQ	#VALUE!	1	#VALUE!
0	T12	F12	0	0	kleiner LLOQ	#VALUE!	1	#VALUE!
0	T12	G01	0	0	kleiner LLOQ	#VALUE!	1	#VALUE!
0	T12	G02	0	0	kleiner LLOQ	#VALUE!	1	#VALUE!
0	T12	G03	0	0	kleiner LLOQ	#VALUE!	1	#VALUE!
0	T12	G04	0	0	kleiner LLOQ	#VALUE!	1	#VALUE!
0	T12	G05	0	0	kleiner LLOQ	#VALUE!	1	#VALUE!
0	T12	G06	0	0	kleiner LLOQ	#VALUE!	1	#VALUE!
Blank PEG	T12	G07	0.000	-0.051	kleiner LLOQ	#VALUE!	2.75	#VALUE!
Blank PEG	T12	G08	0.000	-0.051	kleiner LLOQ	#VALUE!	2.75	#VALUE!
Blank PEG	T12	G09	0.000	-0.051	kleiner LLOQ	#VALUE!	2.75	#VALUE!
Blank PEG	T12	G10	0.000	-0.051	kleiner LLOQ	#VALUE!	2.75	#VALUE!
Blank PEG	T12	G11	0.000	-0.051	kleiner LLOQ	#VALUE!	2.75	#VALUE!
Blank PEG	T12	G12	0.000	-0.051	kleiner LLOQ	#VALUE!	2.75	#VALUE!
0	T12	H01	0	0	kleiner LLOQ	#VALUE!	1	#VALUE!
0	T12	H02	0	0	kleiner LLOQ	#VALUE!	1	#VALUE!
0	T12	H03	0	0	kleiner LLOQ	#VALUE!	1	#VALUE!
0	T12	H04	0	0	kleiner LLOQ	#VALUE!	1	#VALUE!
0	T12	H05	0	0	kleiner LLOQ	#VALUE!	1	#VALUE!
0	T12	H06	0	0	kleiner LLOQ	#VALUE!	1	#VALUE!
Blank no PEG	T12	H07	0.000	-0.051	kleiner LLOQ	#VALUE!	2.75	#VALUE!
Blank no PEG	T12	H08	0.000	-0.051	kleiner LLOQ	#VALUE!	2.75	#VALUE!
Blank no PEG	T12	H09	0.000	-0.051	kleiner LLOQ	#VALUE!	2.75	#VALUE!
Blank no PEG	T12	H10	0.000	-0.051	kleiner LLOQ	#VALUE!	2.75	#VALUE!
Blank no PEG	T12	H11	0.000	-0.051	kleiner LLOQ	#VALUE!	2.75	#VALUE!
Blank no PEG	T12	H12	0.000	-0.051	kleiner LLOQ	#VALUE!	2.75	#VALUE!

0	B	A01	0	0	kleiner LLOQ	#VALUE!	2	#VALUE!
0	B	A02	0	0	kleiner LLOQ	#VALUE!	3	#VALUE!
0	B	A03	0	0	kleiner LLOQ	#VALUE!	4	#VALUE!
0	B	A04	0	0	kleiner LLOQ	#VALUE!	5	#VALUE!
0	B	A05	0	0	kleiner LLOQ	#VALUE!	6	#VALUE!
0	B	A06	0	0	kleiner LLOQ	#VALUE!	7	#VALUE!
0	B	A07	0	0	kleiner LLOQ	#VALUE!	8	#VALUE!
0	B	A08	0	0	kleiner LLOQ	#VALUE!	9	#VALUE!
0	B	A09	0	0	kleiner LLOQ	#VALUE!	10	#VALUE!
0	B	A10	0	0	kleiner LLOQ	#VALUE!	11	#VALUE!
0	B	A11	0	0	kleiner LLOQ	#VALUE!	12	#VALUE!
0	B	A12	0	0	kleiner LLOQ	#VALUE!	13	#VALUE!
0	B	B01	0	0	kleiner LLOQ	#VALUE!	14	#VALUE!
0	B	B02	0	0	kleiner LLOQ	#VALUE!	15	#VALUE!
0	B	B03	0	0	kleiner LLOQ	#VALUE!	16	#VALUE!
0	B	B04	0	0	kleiner LLOQ	#VALUE!	17	#VALUE!
0	B	B05	0	0	kleiner LLOQ	#VALUE!	18	#VALUE!
0	B	B06	0	0	kleiner LLOQ	#VALUE!	19	#VALUE!
0	B	B07	0	0	kleiner LLOQ	#VALUE!	20	#VALUE!
0	B	B08	0	0	kleiner LLOQ	#VALUE!	21	#VALUE!
0	B	B09	0	0	kleiner LLOQ	#VALUE!	22	#VALUE!
0	B	B10	0	0	kleiner LLOQ	#VALUE!	23	#VALUE!
0	B	B11	0	0	kleiner LLOQ	#VALUE!	24	#VALUE!
0	B	B12	0	0	kleiner LLOQ	#VALUE!	25	#VALUE!
0	B	C01	0	0	kleiner LLOQ	#VALUE!	26	#VALUE!
0	B	C02	0	0	kleiner LLOQ	#VALUE!	27	#VALUE!
0	B	C03	0	0	kleiner LLOQ	#VALUE!	28	#VALUE!
0	B	C04	0	0	kleiner LLOQ	#VALUE!	29	#VALUE!
0	B	C05	0	0	kleiner LLOQ	#VALUE!	30	#VALUE!
0	B	C06	0	0	kleiner LLOQ	#VALUE!	31	#VALUE!
0	B	C07	0	0	kleiner LLOQ	#VALUE!	32	#VALUE!
0	B	C08	0	0	kleiner LLOQ	#VALUE!	33	#VALUE!
0	B	C09	0	0	kleiner LLOQ	#VALUE!	34	#VALUE!
0	B	C10	0	0	kleiner LLOQ	#VALUE!	35	#VALUE!
0	B	C11	0	0	kleiner LLOQ	#VALUE!	36	#VALUE!
0	B	C12	0	0	kleiner LLOQ	#VALUE!	37	#VALUE!
0	B	D01	0	0	kleiner LLOQ	#VALUE!	38	#VALUE!
0	B	D02	0	0	kleiner LLOQ	#VALUE!	39	#VALUE!
0	B	D03	0	0	kleiner LLOQ	#VALUE!	40	#VALUE!
0	B	D04	0	0	kleiner LLOQ	#VALUE!	41	#VALUE!
0	B	D05	0	0	kleiner LLOQ	#VALUE!	42	#VALUE!
0	B	D06	0	0	kleiner LLOQ	#VALUE!	43	#VALUE!
0	B	D07	0	0	kleiner LLOQ	#VALUE!	44	#VALUE!
0	B	D08	0	0	kleiner LLOQ	#VALUE!	45	#VALUE!
0	B	D09	0	0	kleiner LLOQ	#VALUE!	46	#VALUE!
0	B	D10	0	0	kleiner LLOQ	#VALUE!	47	#VALUE!
0	B	D11	0	0	kleiner LLOQ	#VALUE!	48	#VALUE!
0	B	D12	0	0	kleiner LLOQ	#VALUE!	49	#VALUE!

Plate: B

	1	2	3	4	5	6	7	8	9	10	11	12
A												
B												
C												
D												
E												
F												
G												
H												

Plattenbelegung

	1	2	3	4	5	6	7	8	9	10	11	12
A												
B												
C												
D												
E												
F												
G												
H												

Comment:

Date Operator

Date Control

0	B	E01	0	0	kleiner LLOQ	#VALUE!	50	#VALUE!
0	B	E02	0	0	kleiner LLOQ	#VALUE!	51	#VALUE!
0	B	E03	0	0	kleiner LLOQ	#VALUE!	52	#VALUE!
0	B	E04	0	0	kleiner LLOQ	#VALUE!	53	#VALUE!
0	B	E05	0	0	kleiner LLOQ	#VALUE!	54	#VALUE!
0	B	E06	0	0	kleiner LLOQ	#VALUE!	55	#VALUE!
0	B	E07	0	0	kleiner LLOQ	#VALUE!	56	#VALUE!
0	B	E08	0	0	kleiner LLOQ	#VALUE!	57	#VALUE!
0	B	E09	0	0	kleiner LLOQ	#VALUE!	58	#VALUE!
0	B	E10	0	0	kleiner LLOQ	#VALUE!	59	#VALUE!
0	B	E11	0	0	kleiner LLOQ	#VALUE!	60	#VALUE!
0	B	E12	0	0	kleiner LLOQ	#VALUE!	61	#VALUE!
0	B	F01	0	0	kleiner LLOQ	#VALUE!	62	#VALUE!
0	B	F02	0	0	kleiner LLOQ	#VALUE!	63	#VALUE!
0	B	F03	0	0	kleiner LLOQ	#VALUE!	64	#VALUE!
0	B	F04	0	0	kleiner LLOQ	#VALUE!	65	#VALUE!
0	B	F05	0	0	kleiner LLOQ	#VALUE!	66	#VALUE!
0	B	F06	0	0	kleiner LLOQ	#VALUE!	67	#VALUE!
0	B	F07	0	0	kleiner LLOQ	#VALUE!	68	#VALUE!
0	B	F08	0	0	kleiner LLOQ	#VALUE!	69	#VALUE!
0	B	F09	0	0	kleiner LLOQ	#VALUE!	70	#VALUE!
0	B	F10	0	0	kleiner LLOQ	#VALUE!	71	#VALUE!
0	B	F11	0	0	kleiner LLOQ	#VALUE!	72	#VALUE!
0	B	F12	0	0	kleiner LLOQ	#VALUE!	73	#VALUE!
0	B	G01	0	0	kleiner LLOQ	#VALUE!	74	#VALUE!
0	B	G02	0	0	kleiner LLOQ	#VALUE!	75	#VALUE!
0	B	G03	0	0	kleiner LLOQ	#VALUE!	76	#VALUE!
0	B	G04	0	0	kleiner LLOQ	#VALUE!	77	#VALUE!
0	B	G05	0	0	kleiner LLOQ	#VALUE!	78	#VALUE!
0	B	G06	0	0	kleiner LLOQ	#VALUE!	79	#VALUE!
0	B	G07	0	0	kleiner LLOQ	#VALUE!	80	#VALUE!
0	B	G08	0	0	kleiner LLOQ	#VALUE!	81	#VALUE!
0	B	G09	0	0	kleiner LLOQ	#VALUE!	82	#VALUE!
0	B	G10	0	0	kleiner LLOQ	#VALUE!	83	#VALUE!
0	B	G11	0	0	kleiner LLOQ	#VALUE!	84	#VALUE!
0	B	G12	0	0	kleiner LLOQ	#VALUE!	85	#VALUE!
0	B	H01	0	0	kleiner LLOQ	#VALUE!	86	#VALUE!
0	B	H02	0	0	kleiner LLOQ	#VALUE!	87	#VALUE!
0	B	H03	0	0	kleiner LLOQ	#VALUE!	88	#VALUE!
0	B	H04	0	0	kleiner LLOQ	#VALUE!	89	#VALUE!
0	B	H05	0	0	kleiner LLOQ	#VALUE!	90	#VALUE!
0	B	H06	0	0	kleiner LLOQ	#VALUE!	91	#VALUE!
0	B	H07	0	0	kleiner LLOQ	#VALUE!	92	#VALUE!
0	B	H08	0	0	kleiner LLOQ	#VALUE!	93	#VALUE!
0	B	H09	0	0	kleiner LLOQ	#VALUE!	94	#VALUE!
0	B	H10	0	0	kleiner LLOQ	#VALUE!	95	#VALUE!
0	B	H11	0	0	kleiner LLOQ	#VALUE!	96	#VALUE!
0	B	H12	0	0	kleiner LLOQ	#VALUE!	97	#VALUE!

1.447	1.739		1.170						0.667	0.440	0.043	1
1.447	1.332		1.392	0.137	0.146	0.130	0.138	0.109	0.729	0.478	0.042	
1.391	1.281		1.311	0.204	0.184	0.184	0.198	0.201	0.743	0.474	0.043	
0.961	0.835		1.405	0.336	0.347	0.375	0.387	0.300	0.824	0.483	0.044	
0.445	0.441		1.280	1.085	1.086	1.218	1.004	0.873	0.704	0.457	0.041	
0.355	0.315		1.332	1.768	1.925	1.753	1.739	1.606			0.036	
0.223	0.188	1.666	1.499	1.632		0.048	0.046	0.047	0.048	0.048	0.048	
0.118	0.106	1.556	1.622	1.573		0.047	0.054	0.051	0.051	0.052	0.051	

1.652	1.627		1.188	0.748	0.811	0.764	0.753	0.674	0.751	0.529	0.044	2
1.443	1.372		1.364	0.139	0.147	0.131	0.139	0.110	0.811	0.561	0.043	
1.377	1.281		1.284	0.206	0.189	0.184	0.200	0.202	0.821	0.560	0.044	
0.972	0.883		1.387	0.339	0.349	0.386	0.406	0.312	0.903	0.565	0.044	
0.484	0.474		1.298	1.087	1.113	1.203	1.022	0.903	0.790	0.544	0.042	
0.359	0.326		1.308	1.622	1.622	1.630	1.625	1.550			0.037	
0.228	0.199	1.527	1.373	1.462		0.048	0.046	0.048	0.049	0.049	0.049	
0.125	0.109	1.473	1.410	1.371		0.048	0.054	0.052	0.052	0.052	0.051	

1.579	1.475		1.150	1.210	1.303	1.202	1.219	1.125	0.759	0.549	0.043	3
1.357	1.303		1.283	0.140	0.148	0.131	0.139	0.111	0.820	0.575	0.043	
1.323	1.228		1.227	0.206	0.188	0.184	0.198	0.201	0.824	0.579	0.043	
0.959	0.872		1.321	0.339	0.348	0.387	0.406	0.314	0.909	0.580	0.044	
0.483	0.473		1.270	1.069	1.103	1.181	1.017	0.903	0.798	0.560	0.042	
0.360	0.326		1.270	1.497	1.448	1.496	1.494	1.449			0.037	
0.228	0.199	1.374	1.262	1.314		0.048	0.046	0.048	0.049	0.049	0.049	
0.125	0.110	1.346	1.262	1.253		0.048	0.054	0.052	0.052	0.052	0.051	

1.511	1.369			1.538	1.712	1.528	1.502	1.425			0.043	4
1.296	1.261										0.043	
1.272	1.193										0.043	
0.947	0.859										0.044	
0.481	0.471										0.042	
0.361	0.325										0.037	
0.229	0.199					0.048	0.046	0.048	0.049	0.049	0.049	
0.125	0.110					0.047	0.054	0.052	0.052	0.052	0.051	

1.493	1.317	1.678	1.110	1.621	1.807	1.618	1.584	1.508	0.765	0.597	0.043	5
1.259	1.238	1.850	1.197	0.139	0.150	0.132	0.139	0.111	0.821	0.588	0.043	
1.248	1.175	1.816	1.153	0.204	0.188	0.184	0.197	0.199	0.831	0.599	0.044	
0.943	0.859	1.970	1.231	0.338	0.357	0.386	0.406	0.316	0.916	0.595	0.044	
0.480	0.470	2.220	1.220	1.054	1.088	1.154	1.006	0.902	0.805	0.579	0.042	
0.359	0.325	2.149	1.211	1.395	1.324	1.348	1.345	1.317	0.037	0.037	0.037	
0.228	0.199	1.240	1.151	1.183	0.034	0.049	0.046	0.048	0.049	0.049	0.049	
0.125	0.110	1.223	1.132	1.146	0.038	0.048	0.054	0.052	0.052	0.052	0.051	

1.434	1.258		1.090	1.648	1.876	1.649	1.627	1.535				6
1.217	1.201		1.149									
1.205	1.143		1.109									
0.935	0.845		1.182									
0.477	0.469		1.181									
0.360	0.323		1.167									
0.229	0.200					0.049	0.047	0.048	0.049	0.049	0.049	
0.125	0.110					0.048	0.054	0.052	0.052	0.052	0.051	

1.013	0.810		0.668	0.808	0.940	0.720	0.798	0.687	0.904	0.845	0.043	7
0.709	0.827		0.703	0.142	0.156	0.136	0.137	0.116	0.990	0.846	0.042	
0.682	0.692		0.680	0.197	0.188	0.190	0.191	0.184	1.023	0.861	0.043	
0.507	0.519		0.740	0.316	0.264	0.364	0.377	0.312	1.111	0.870	0.043	
0.394	0.406		0.682	0.580	0.561	0.600	0.529	0.563	0.962	0.834	0.042	
0.333	0.322		0.661	0.995	0.930	0.848	0.832	0.797	0.037	0.037	0.037	
0.229	0.201	0.843	0.679	0.732		0.048	0.046	0.047			0.047	
0.124	0.111	0.773	0.682	0.701		0.047	0.053	0.051	0.051	0.052	0.051	

skin cultivation

T12 1 hum sui

0.462	0.469	0.446	0.350								
0.210	0.164	0.206	0.204								
0.846	0.781	0.669	0.234								
	0.384	0.451	0.605								
0.660	0.298	0.139	0.235								
0.568	0.248	0.331	0.314								
						0.048	0.049	0.049	0.049	0.048	0.048
						0.047	0.047	0.047	0.047	0.047	0.047

T12 2 hum sui

				0.519	0.533	0.518	0.519	0.484	0.513	0.362	0.354
				0.206	0.207	0.164	0.166	0.196	0.182	0.199	0.199
				0.835	0.831	0.740	0.760	0.624	0.633	0.245	0.234
						0.392	0.404	0.444	0.467	0.574	0.587
				0.636	0.622	0.342	0.371	0.439	0.463	0.228	0.226
				0.481	0.487	0.294	0.226	0.298	0.299	0.306	0.308
						0.049	0.049	0.050	0.049	0.049	0.049
						0.047	0.047	0.047	0.048	0.048	0.047

T13 1 hum sui

0.425	0.375	0.413	0.352								
0.157	0.171	0.185	0.174								
0.885	0.650	0.522	0.254								
	0.490	0.430	0.632								
0.474	0.257	0.146	0.232								
0.497	0.247	0.286	0.298								
						0.049	0.049	0.049	0.050	0.050	0.049
						0.048	0.047	0.049	0.048	0.047	0.048

T13 2 hum sui

				0.454	0.424	0.350	0.386	0.444	0.434	0.382	0.382
				0.158	0.201	0.183	0.182	0.159	0.156	0.184	0.190
				0.933	0.873	0.744	0.758	0.539	0.581	0.256	0.289
						0.554	0.540	0.464	0.471	0.680	0.708
				0.472	0.430	0.233	0.247	0.137	0.142	0.228	0.235
				0.320	0.258	0.191	0.220	0.250	0.250	0.294	0.305
						0.050	0.050	0.053	0.051	0.051	0.050
						0.048	0.048	0.048	0.048	0.048	0.048

T14 1 hum sui

0.389	0.411	0.492	0.417								
0.172	0.168	0.194	0.181								
0.877	0.727	0.696	0.348								
0.040	0.140	0.155	0.244								
0.511	0.320	0.164	0.243								
0.617	0.263	0.314	0.321								
						0.042	0.048	0.048	0.050	0.049	0.048
						0.045	0.046	0.047	0.049	0.047	0.048

T14 2 hum sui

				0.308	0.290	0.352	0.377	0.386	0.406	0.362	0.392
				0.183	0.180	0.174	0.178	0.170	0.174	0.187	0.256
				0.667	0.663	0.572	0.566	0.541	0.553	0.416	0.389
						0.467	0.518	0.607	0.573	0.510	0.574
				0.448	0.454	0.290	0.288	0.177	0.183	0.311	0.279
				0.471	0.511	0.236	0.261	0.259	0.349	0.350	0.224
						0.041	0.048	0.048	0.048	0.048	0.047
						0.044	0.045	0.046	0.048	0.046	0.048

Project number		F-120	Apparatus	Wallac Victor	Operator	IsBa
GLP Study (Number)		n.a.	Protocol (Instrument method)	LDH test 2016	Date of preparation	19-04-18
hot substance	isotope	n.a.	File name (results)	IsBa_180419/20_LDH_full_va	Date of measurement	19-04-18
	name	n.a.	Kind of well plate	96 well	shaking time [min]	30
	ACB-ID	n.a.	sample volume [µL]	100	stirring rate (Target) [rpm]	150
	Batch number	n.a.	Cocktail volume [µL]	175	Kind of measurement	UV-vis
cold substance	name	LDH	ACB-ID of cocktail		Wave length [nm]	450
	ACB-ID	L23982R	Matrix	DMEM (from powder)+PEG	Remarks	Cocktail 100µl RM, 75µl STOP
	Batch number	SLBK6345V	Blank description	DMEM/PEG, H2O	Remarks	7 standards split low/high
n.a.		n.a.	Pipettes (No. / volume)	50-200µl	Remarks	KLP4 common for both
n.a.		n.a.	Pipettes (No. / volume)	n.a.	Remarks	n.a.

Messdaten (diese Tabelle in Bericht übernehmen)

Sample name *	concentration (theor.) *	measured data	measured data	measured data	mean measured	SD	RSD	Blank *	measured data after * Blank subtraction	concentration (calc.) *	Deviation *	Residuen
	[µg/mL]	[AU]	[AU]	[AU]	[AU]	[AU]	[%]	[AU]		[µg/mL]	[%]	
KLP1								0.047				
KLP2								0.054				
KLP3								0.051				
KLP4	0.041	0.959	0.972	0.883	0.938	0.04	4.19	0.051	0.887	0.041	-0.57	0.00
KLP5	0.018	0.483	0.484	0.474	0.480	0.00	0.98	0.052	0.429	0.019	2.42	0.00
KLP6	0.012	0.360	0.359	0.326	0.348	0.02	4.54	0.051	0.297	0.013	5.00	0.00
KLP7	0.007	0.228	0.228	0.199	0.218	0.014	6.35		0.168	0.006	-11.29	0.00
KLP8												

Statistical data

Geradensteigung	Slope	m	21.03
Y-Achsenabschnitt	Y-Intercept	b	0.03
Standardabw. Geradensteigung	SD-Slope	s_m	0.648055501
Standardabw. Achsenabschnittes	SD-Y-Intercept	s_b	0.015177452
Anzahl Messpunkte	number of measuring points	n	4
Quadratsumme	sum of squares	Qxx	0.000664048
Bereichsmitte			0.019557193
Freiheitsgrade	degree of freedom	f	2
Student-t-Faktor für (P = 95 %; f = n-2)	Student-t-factor for (P = 95 %; f = n-2)	t	4.303
Vertrauensbereich Steig. (95 %) Obergrenze		$m + VB_m$	23.81763508
Vertrauensbereich Steig. (95 %) Untergrenze		$m - VB_m$	18.24046944
Vertrauensbereich Achsenabschnitt (95 %) Og.		$b + Vb_b$	0.099335829
Vertrauensbereich Achsenabschnitt (95 %) Ug.		$b - Vb_b$	-0.031281327
Korrelationskoeffizient	correlation coefficient	r	0.9991
Bestimmtheitsmaß	determiantion coefficient	r^2	0.9981
Reststandardabweichung		s_0	0.01669983
Summe Restquadrate		sd	0.657385838
Verfahrensstandardabw.		s_{x0}	0.000794131
Rel. Verfahrensstandardabw. %		V_{x0}	4.060559029

Berichten

mean Blank	0	
SD Blank	0.00	
RSD Blank	3.79	x=
x*SD (LLOQ)	0.01	5
x*SD (LOD)	0.01	3
**LLOQ (AU)**	**0.061**	
**LOD (AU)**	**0.057**	
ULOQ	0.887	
LLOQ (Lin)	0.168	

Evaluation / Comment

LDH linearity valid with 7 standards, split in lin high and lin low, each with 4 standards and KLP4 common standard for both. R² 0,9981, deviations for both between -13.34% and +13.04%

Date | Operator | Date | Control

Figure 1 **Linearity**

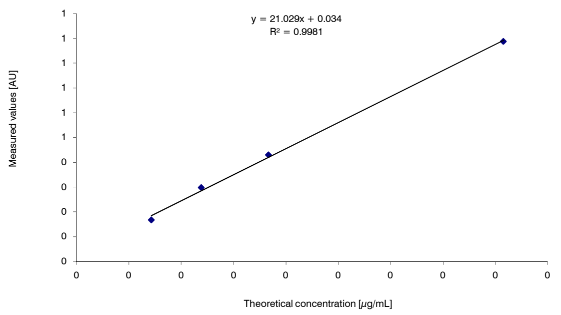

Figure 2 **Method validation Residuen Plot**

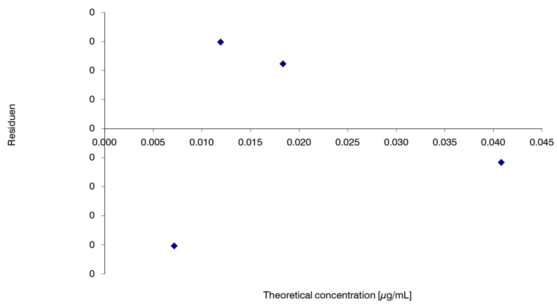

Evaluation / Comment

lin passed

Date	Operator	Date	Control

Sample name	Plate	Position	[AU]	[AU]-Blank	Rating	[µg/mL]	Dilution factor	[µg/mL]
0	T0	A01	0	0	kleiner LLOQ	#VALUE!	1	#VALUE!
0	T0	A02	0	0	kleiner LLOQ	#VALUE!	1	#VALUE!
0	T0	A03	0	0	kleiner LLOQ	#VALUE!	1	#VALUE!
0	T0	A04	0	0	kleiner LLOQ	#VALUE!	1	#VALUE!
0	T0	A05	0	0	kleiner LLOQ	#VALUE!	1	#VALUE!
0	T0	A06	0	0	kleiner LLOQ	#VALUE!	1	#VALUE!
0	T0	A07	0	0	kleiner LLOQ	#VALUE!	1	#VALUE!
0	T0	A08	0	0	kleiner LLOQ	#VALUE!	1	#VALUE!
0	T0	A09	0	0	kleiner LLOQ	#VALUE!	1	#VALUE!
0	T0	A10	0	0	kleiner LLOQ	#VALUE!	1	#VALUE!
0	T0	A11	0	0	kleiner LLOQ	#VALUE!	1	#VALUE!
0	T0	A12	0	0	kleiner LLOQ	#VALUE!	1	#VALUE!
0	T0	B01	0	0	kleiner LLOQ	#VALUE!	1	#VALUE!
0	T0	B02	0	0	kleiner LLOQ	#VALUE!	1	#VALUE!
0	T0	B03	0	0	kleiner LLOQ	#VALUE!	1	#VALUE!
0	T0	B04	0	0	kleiner LLOQ	#VALUE!	1	#VALUE!
0	T0	B05	0	0	kleiner LLOQ	#VALUE!	1	#VALUE!
0	T0	B06	0	0	kleiner LLOQ	#VALUE!	1	#VALUE!
0	T0	B07	0	0	kleiner LLOQ	#VALUE!	1	#VALUE!
0	T0	B08	0	0	kleiner LLOQ	#VALUE!	1	#VALUE!
0	T0	B09	0	0	kleiner LLOQ	#VALUE!	1	#VALUE!
0	T0	B10	0	0	kleiner LLOQ	#VALUE!	1	#VALUE!
0	T0	B11	0	0	kleiner LLOQ	#VALUE!	1	#VALUE!
0	T0	B12	0	0	kleiner LLOQ	#VALUE!	1	#VALUE!
0	T0	C01	0	0	kleiner LLOQ	#VALUE!	1	#VALUE!
0	T0	C02	0	0	kleiner LLOQ	#VALUE!	1	#VALUE!
0	T0	C03	0	0	kleiner LLOQ	#VALUE!	1	#VALUE!
0	T0	C04	0	0	kleiner LLOQ	#VALUE!	1	#VALUE!
0	T0	C05	0	0	kleiner LLOQ	#VALUE!	1	#VALUE!
0	T0	C06	0	0	kleiner LLOQ	#VALUE!	1	#VALUE!
0	T0	C07	0	0	kleiner LLOQ	#VALUE!	1	#VALUE!
0	T0	C08	0	0	kleiner LLOQ	#VALUE!	1	#VALUE!
0	T0	C09	0	0	kleiner LLOQ	#VALUE!	1	#VALUE!
0	T0	C10	0	0	kleiner LLOQ	#VALUE!	1	#VALUE!
0	T0	C11	0	0	kleiner LLOQ	#VALUE!	1	#VALUE!
0	T0	C12	0	0	kleiner LLOQ	#VALUE!	1	#VALUE!
0	T0	D01	0	0	kleiner LLOQ	#VALUE!	1	#VALUE!
0	T0	D02	0	0	kleiner LLOQ	#VALUE!	1	#VALUE!
0	T0	D03	0	0	kleiner LLOQ	#VALUE!	1	#VALUE!
0	T0	D04	0	0	kleiner LLOQ	#VALUE!	1	#VALUE!
T04SN1	T0	D05	0	0	kleiner LLOQ	#VALUE!	1	#VALUE!
T04SN1	T0	D06	0	0	kleiner LLOQ	#VALUE!	1	#VALUE!
T04SN2	T0	D07	0	0	kleiner LLOQ	#VALUE!	1	#VALUE!
T04SN2	T0	D08	0	0	kleiner LLOQ	#VALUE!	1	#VALUE!
T04SN3	T0	D09	0	0	kleiner LLOQ	#VALUE!	1	#VALUE!
T04SN3	T0	D10	0	0	kleiner LLOQ	#VALUE!	1	#VALUE!
T04SN4	T0	D11	0	0	kleiner LLOQ	#VALUE!	1	#VALUE!
T04SN4	T0	D12	0.742	0.691	0.691	0.031	11	0.344

Plate:	T0

	1	2	3	4	5	6	7	8	9	10	11	12
A												
B												
C												
D												0.742
E											0.618	0.508
F											0.912	0.852
G	0.065	0.066	0.062	0.072	0.060	0.070	0.056	0.057	0.057	0.055	0.056	0.054
H	0.044	0.045	0.048	0.046	0.047	0.044	0.051	0.051	0.051	0.054	0.05	0.049

Plattenbelegung

	1	2	3	4	5	6	7	8	9	10	11	12
A												
B												
C												
D					T04SN1	T04SN1	T04SN2	T04SN2	T04SN3	T04SN3	T04SN4	T04SN4
E					T04SS11	T04SS11	T04SS12	T04SS12	T04SS13	T04SS13	T04SS14	T04SS14
F					T04SS21	T04SS21	T04SS22	T04SS22	T04SS23	T04SS23	T04SS24	T04SS24
G	Blank PEG	Blank PEG	Blank PEG	Blank PEG	Blank PEG	Blank PEG	Blank PEG	Blank PEG	Blank PEG	Blank PEG	Blank PEG	Blank PEG
H	Blank no PEG	Blank no PEG	Blank no PEG	Blank no PEG	Blank no PEG	Blank no PEG	Blank no PEG	Blank no PEG	Blank no PEG	Blank no PEG	Blank no PEG	Blank no PEG

Samples aufgeteilt in lin high und low:höher konzentrierte Samples bei lin high zu finden.

dil.4*dil2,75=dil.11

dil4*dil.8*dil.2,75=dil.88 oder nur von ursprüngl. Dil 4 auf dil 8 erhöht???

Comment:

Date Operator

Date Control

0	T0	E01	0	0	kleiner LLOQ	#VALUE!	1	#VALUE!
0	T0	E02	0	0	kleiner LLOQ	#VALUE!	1	#VALUE!
0	T0	E03	0	0	kleiner LLOQ	#VALUE!	1	#VALUE!
0	T0	E04	0	0	kleiner LLOQ	#VALUE!	1	#VALUE!
T04SS11	T0	E05	0	0	kleiner LLOQ	#VALUE!	1	#VALUE!
T04SS11	T0	E06	0	0	kleiner LLOQ	#VALUE!	1	#VALUE!
T04SS12	T0	E07	0	0	kleiner LLOQ	#VALUE!	1	#VALUE!
T04SS12	T0	E08	0	0	kleiner LLOQ	#VALUE!	1	#VALUE!
T04SS13	T0	E09	0	0	kleiner LLOQ	#VALUE!	1	#VALUE!
T04SS13	T0	E10	0	0	kleiner LLOQ	#VALUE!	1	#VALUE!
T04SS14	T0	E11	0.618	0.567	0.567	0.025	11	0.279
T04SS14	T0	E12	0.508	0.457	0.457	0.020	11	0.221
0	T0	F01	0	0	kleiner LLOQ	#VALUE!	1	#VALUE!
0	T0	F02	0	0	kleiner LLOQ	#VALUE!	1	#VALUE!
0	T0	F03	0	0	kleiner LLOQ	#VALUE!	1	#VALUE!
0	T0	F04	0	0	kleiner LLOQ	#VALUE!	1	#VALUE!
T04SS21	T0	F05	0	0	kleiner LLOQ	#VALUE!	1	#VALUE!
T04SS21	T0	F06	0	0	kleiner LLOQ	#VALUE!	1	#VALUE!
T04SS22	T0	F07	0	0	kleiner LLOQ	#VALUE!	1	#VALUE!
T04SS22	T0	F08	0	0	kleiner LLOQ	#VALUE!	1	#VALUE!
T04SS23	T0	F09	0	0	kleiner LLOQ	#VALUE!	1	#VALUE!
T04SS23	T0	F10	0	0	kleiner LLOQ	#VALUE!	1	#VALUE!
T04SS24	T0	F11	0.912	0.861	0.861	0.039	11	0.433
T04SS24	T0	F12	0.852	0.801	0.801	0.036	11	0.401
Blank PEG	T0	G01	0	0	kleiner LLOQ	#VALUE!	2.75	#VALUE!
Blank PEG	T0	G02	0	0	kleiner LLOQ	#VALUE!	2.75	#VALUE!
Blank PEG	T0	G03	0	0	kleiner LLOQ	#VALUE!	2.75	#VALUE!
Blank PEG	T0	G04	0	0	kleiner LLOQ	#VALUE!	2.75	#VALUE!
Blank PEG	T0	G05	0	0	kleiner LLOQ	#VALUE!	2.75	#VALUE!
Blank PEG	T0	G06	0	0	kleiner LLOQ	#VALUE!	2.75	#VALUE!
Blank PEG	T0	G07	0	0	kleiner LLOQ	#VALUE!	2.75	#VALUE!
Blank PEG	T0	G08	0	0	kleiner LLOQ	#VALUE!	2.75	#VALUE!
Blank PEG	T0	G09	0	0	kleiner LLOQ	#VALUE!	2.75	#VALUE!
Blank PEG	T0	G10	0	0	kleiner LLOQ	#VALUE!	2.75	#VALUE!
Blank PEG	T0	G11	0	0	kleiner LLOQ	#VALUE!	2.75	#VALUE!
Blank PEG	T0	G12	0	0	kleiner LLOQ	#VALUE!	2.75	#VALUE!
Blank no PEG	T0	H01	0	0	kleiner LLOQ	#VALUE!	2.75	#VALUE!
Blank no PEG	T0	H02	0	0	kleiner LLOQ	#VALUE!	2.75	#VALUE!
Blank no PEG	T0	H03	0	0	kleiner LLOQ	#VALUE!	2.75	#VALUE!
Blank no PEG	T0	H04	0	0	kleiner LLOQ	#VALUE!	2.75	#VALUE!
Blank no PEG	T0	H05	0	0	kleiner LLOQ	#VALUE!	2.75	#VALUE!
Blank no PEG	T0	H06	0	0	kleiner LLOQ	#VALUE!	2.75	#VALUE!
Blank no PEG	T0	H07	0	0	kleiner LLOQ	#VALUE!	2.75	#VALUE!
Blank no PEG	T0	H08	0	0	kleiner LLOQ	#VALUE!	2.75	#VALUE!
Blank no PEG	T0	H09	0	0	kleiner LLOQ	#VALUE!	2.75	#VALUE!
Blank no PEG	T0	H10	0	0	kleiner LLOQ	#VALUE!	2.75	#VALUE!
Blank no PEG	T0	H11	0	0	kleiner LLOQ	#VALUE!	2.75	#VALUE!
Blank no PEG	T0	H12	0	0	kleiner LLOQ	#VALUE!	2.75	#VALUE!

0	T1	A01	0	0	kleiner LLOQ	#VALUE!	1	#VALUE!
0	T1	A02	0	0	kleiner LLOQ	#VALUE!	1	#VALUE!
0	T1	A03	0	0	kleiner LLOQ	#VALUE!	1	#VALUE!
0	T1	A04	0	0	kleiner LLOQ	#VALUE!	1	#VALUE!
0	T1	A05	0	0	kleiner LLOQ	#VALUE!	1	#VALUE!
0	T1	A06	0	0	kleiner LLOQ	#VALUE!	1	#VALUE!
0	T1	A07	0	0	kleiner LLOQ	#VALUE!	1	#VALUE!
0	T1	A08	0	0	kleiner LLOQ	#VALUE!	1	#VALUE!
0	T1	A09	0	0	kleiner LLOQ	#VALUE!	1	#VALUE!
0	T1	A10	0	0	kleiner LLOQ	#VALUE!	1	#VALUE!
0	T1	A11	0	0	kleiner LLOQ	#VALUE!	1	#VALUE!
0	T1	A12	0	0	kleiner LLOQ	#VALUE!	1	#VALUE!
0	T1	B01	0	0	kleiner LLOQ	#VALUE!	1	#VALUE!
0	T1	B02	0	0	kleiner LLOQ	#VALUE!	1	#VALUE!
0	T1	B03	0	0	kleiner LLOQ	#VALUE!	1	#VALUE!
0	T1	B04	0	0	kleiner LLOQ	#VALUE!	1	#VALUE!
0	T1	B05	0	0	kleiner LLOQ	#VALUE!	1	#VALUE!
0	T1	B06	0	0	kleiner LLOQ	#VALUE!	1	#VALUE!
0	T1	B07	0	0	kleiner LLOQ	#VALUE!	1	#VALUE!
0	T1	B08	0	0	kleiner LLOQ	#VALUE!	1	#VALUE!
0	T1	B09	0	0	kleiner LLOQ	#VALUE!	1	#VALUE!
0	T1	B10	0	0	kleiner LLOQ	#VALUE!	1	#VALUE!
0	T1	B11	0	0	kleiner LLOQ	#VALUE!	1	#VALUE!
0	T1	B12	0	0	kleiner LLOQ	#VALUE!	1	#VALUE!
0	T1	C01	0	0	kleiner LLOQ	#VALUE!	1	#VALUE!
0	T1	C02	0	0	kleiner LLOQ	#VALUE!	1	#VALUE!
0	T1	C03	0	0	kleiner LLOQ	#VALUE!	1	#VALUE!
0	T1	C04	0	0	kleiner LLOQ	#VALUE!	1	#VALUE!
0	T1	C05	0	0	kleiner LLOQ	#VALUE!	1	#VALUE!
0	T1	C06	0	0	kleiner LLOQ	#VALUE!	1	#VALUE!
0	T1	C07	0	0	kleiner LLOQ	#VALUE!	1	#VALUE!
0	T1	C08	0	0	kleiner LLOQ	#VALUE!	1	#VALUE!
0	T1	C09	0	0	kleiner LLOQ	#VALUE!	1	#VALUE!
0	T1	C10	0	0	kleiner LLOQ	#VALUE!	1	#VALUE!
0	T1	C11	0	0	kleiner LLOQ	#VALUE!	1	#VALUE!
0	T1	C12	0	0	kleiner LLOQ	#VALUE!	1	#VALUE!
0	T1	D01	0	0	kleiner LLOQ	#VALUE!	1	#VALUE!
0	T1	D02	0	0	kleiner LLOQ	#VALUE!	1	#VALUE!
0	T1	D03	0	0	kleiner LLOQ	#VALUE!	1	#VALUE!
0	T1	D04	0	0	kleiner LLOQ	#VALUE!	1	#VALUE!
0	T1	D05	0	0	kleiner LLOQ	#VALUE!	1	#VALUE!
0	T1	D06	0	0	kleiner LLOQ	#VALUE!	1	#VALUE!
T1SN2	T1	D07	0	0	kleiner LLOQ	#VALUE!	1	#VALUE!
T1SN2	T1	D08	0	0	kleiner LLOQ	#VALUE!	1	#VALUE!
0	T1	D09	0	0	kleiner LLOQ	#VALUE!	1	#VALUE!
0	T1	D10	0	0	kleiner LLOQ	#VALUE!	1	#VALUE!
T1SN4	T1	D11	0.342	0.291	0.291	0.012	24.75	0.303
T1SN4	T1	D12	0.313	0.262	0.262	0.011	24.75	0.268

Plate:	T1

	1	2	3	4	5	6	7	8	9	10	11	12
A												
B												
C												
D											0.342	0.313
E											0.439	0.439
F									0.497	0.475	0.692	0.690
G	0.285	0.298			0.555	0.519	0.065	0.053	0.052	0.056	0.050	0.055
H	0.381	0.357	0.306	0.303			0.051	0.051	0.051	0.052	0.052	0.051

Plattenbelegung

	1	2	3	4	5	6	7	8	9	10	11	12
A												
B												
C												
D							T1SN2	T1SN2			T1SN4	T1SN4
E											T1SS14	T1SS14
F									T1SS23	T1SS23	T1SS24	T1SS24
G	T1SN3	T1SN3			T1SN1	T1SN1	Blank PEG	Blank PEG	Blank PEG	Blank PEG	Blank PEG	Blank PEG
H	T1SS12	T1SS12	T1SS13	T1SS13			Blank no PEG	Blank no PEG	Blank no PEG	Blank no PEG	Blank no PEG	Blank no PEG

dil.5*dil.2,75=dil.13,75
dil.9*dil.2,75=dil.24,75
dil.9*dil.4*dil.2,75=dil.99
dil.9*dil.9*dil.2,75=dil.222,75

Comment:

Date Operator

Date Control

0	T1	E01	0	0	kleiner LLOQ	#VALUE!	1	#VALUE!
0	T1	E02	0	0	kleiner LLOQ	#VALUE!	1	#VALUE!
0	T1	E03	0	0	kleiner LLOQ	#VALUE!	1	#VALUE!
0	T1	E04	0	0	kleiner LLOQ	#VALUE!	1	#VALUE!
0	T1	E05	0	0	kleiner LLOQ	#VALUE!	1	#VALUE!
0	T1	E06	0	0	kleiner LLOQ	#VALUE!	1	#VALUE!
0	T1	E07	0	0	kleiner LLOQ	#VALUE!	1	#VALUE!
0	T1	E08	0	0	kleiner LLOQ	#VALUE!	1	#VALUE!
0	T1	E09	0	0	kleiner LLOQ	#VALUE!	1	#VALUE!
0	T1	E10	0	0	kleiner LLOQ	#VALUE!	1	#VALUE!
T1SS14	T1	E11	0.439	0.388	0.388	0.017	24.75	0.416
T1SS14	T1	E12	0.439	0.388	0.388	0.017	24.75	0.416
0	T1	F01	0	0	kleiner LLOQ	#VALUE!	1	#VALUE!
0	T1	F02	0	0	kleiner LLOQ	#VALUE!	1	#VALUE!
0	T1	F03	0	0	kleiner LLOQ	#VALUE!	1	#VALUE!
0	T1	F04	0	0	kleiner LLOQ	#VALUE!	1	#VALUE!
0	T1	F05	0	0	kleiner LLOQ	#VALUE!	1	#VALUE!
0	T1	F06	0	0	kleiner LLOQ	#VALUE!	1	#VALUE!
0	T1	F07	0	0	kleiner LLOQ	#VALUE!	1	#VALUE!
0	T1	F08	0	0	kleiner LLOQ	#VALUE!	1	#VALUE!
T1SS23	T1	F09	0.497	0.447	0.447	0.020	24.75	0.486
T1SS23	T1	F10	0.475	0.424	0.424	0.019	24.75	0.459
T1SS24	T1	F11	0.692	0.641	0.641	0.029	24.75	0.714
T1SS24	T1	F12	0.690	0.639	0.639	0.029	24.75	0.712
T1SN3	T1	G01	0.285	0.234	0.234	0.010	222.75	2.118
T1SN3	T1	G02	0.298	0.247	0.247	0.010	222.75	2.254
0	T1	G03	0	0	kleiner LLOQ	#VALUE!	1	#VALUE!
0	T1	G04	0	0	kleiner LLOQ	#VALUE!	1	#VALUE!
T1SN1	T1	G05	0.555	0.504	0.504	0.022	99	2.214
T1SN1	T1	G06	0.519	0.468	0.468	0.021	99	2.043
Blank PEG	T1	G07	0	0	kleiner LLOQ	#VALUE!	2.75	#VALUE!
Blank PEG	T1	G08	0	0	kleiner LLOQ	#VALUE!	2.75	#VALUE!
Blank PEG	T1	G09	0	0	kleiner LLOQ	#VALUE!	2.75	#VALUE!
Blank PEG	T1	G10	0	0	kleiner LLOQ	#VALUE!	2.75	#VALUE!
Blank PEG	T1	G11	0	0	kleiner LLOQ	#VALUE!	2.75	#VALUE!
Blank PEG	T1	G12	0	0	kleiner LLOQ	#VALUE!	2.75	#VALUE!
T1SS12	T1	H01	0.381	0.330	0.330	0.014	222.75	3.134
T1SS12	T1	H02	0.357	0.306	0.306	0.013	222.75	2.880
T1SS13	T1	H03	0.306	0.255	0.255	0.010	222.75	2.337
T1SS13	T1	H04	0.303	0.252	0.252	0.010	222.75	2.312
0	T1	H05	0	0	kleiner LLOQ	#VALUE!	1	#VALUE!
0	T1	H06	0	0	kleiner LLOQ	#VALUE!	1	#VALUE!
Blank no PEG	T1	H07	0	0	kleiner LLOQ	#VALUE!	2.75	#VALUE!
Blank no PEG	T1	H08	0	0	kleiner LLOQ	#VALUE!	2.75	#VALUE!
Blank no PEG	T1	H09	0	0	kleiner LLOQ	#VALUE!	2.75	#VALUE!
Blank no PEG	T1	H10	0	0	kleiner LLOQ	#VALUE!	2.75	#VALUE!
Blank no PEG	T1	H11	0	0	kleiner LLOQ	#VALUE!	2.75	#VALUE!
Blank no PEG	T1	H12	0	0	kleiner LLOQ	#VALUE!	2.75	#VALUE!
0	T2	A01	0	0	kleiner LLOQ	#VALUE!	1	#VALUE!
0	T2	A02	0	0	kleiner LLOQ	#VALUE!	1	#VALUE!
0	T2	A03	0	0	kleiner LLOQ	#VALUE!	1	#VALUE!
0	T2	A04	0	0	kleiner LLOQ	#VALUE!	1	#VALUE!
0	T2	A05	0	0	kleiner LLOQ	#VALUE!	1	#VALUE!
0	T2	A06	0	0	kleiner LLOQ	#VALUE!	1	#VALUE!
0	T2	A07	0	0	kleiner LLOQ	#VALUE!	1	#VALUE!
0	T2	A08	0	0	kleiner LLOQ	#VALUE!	1	#VALUE!
0	T2	A09	0	0	kleiner LLOQ	#VALUE!	1	#VALUE!
0	T2	A10	0	0	kleiner LLOQ	#VALUE!	1	#VALUE!
0	T2	A11	0	0	kleiner LLOQ	#VALUE!	1	#VALUE!
0	T2	A12	0	0	kleiner LLOQ	#VALUE!	1	#VALUE!
0	T2	B01	0	0	kleiner LLOQ	#VALUE!	1	#VALUE!
0	T2	B02	0	0	kleiner LLOQ	#VALUE!	1	#VALUE!
0	T2	B03	0	0	kleiner LLOQ	#VALUE!	1	#VALUE!
0	T2	B04	0	0	kleiner LLOQ	#VALUE!	1	#VALUE!
0	T2	B05	0	0	kleiner LLOQ	#VALUE!	1	#VALUE!
0	T2	B06	0	0	kleiner LLOQ	#VALUE!	1	#VALUE!
0	T2	B07	0	0	kleiner LLOQ	#VALUE!	1	#VALUE!
0	T2	B08	0	0	kleiner LLOQ	#VALUE!	1	#VALUE!
0	T2	B09	0	0	kleiner LLOQ	#VALUE!	1	#VALUE!
0	T2	B10	0	0	kleiner LLOQ	#VALUE!	1	#VALUE!
0	T2	B11	0	0	kleiner LLOQ	#VALUE!	1	#VALUE!
0	T2	B12	0	0	kleiner LLOQ	#VALUE!	1	#VALUE!

Plate: T2

	1	2	3	4	5	6	7	8	9	10	11	12
A												
B												
C												
D												
E							0.574	0.573	0.572	0.589		
F					0.606	0.608						
G			0.511	0.494	0.744	0.774	0.056	0.055	0.055	0.055	0.056	0.063
H	0.503	0.559	0.312	0.313	0.298	0.250	0.052	0.053	0.053	0.053	0.052	0.052

Plattenbelegung

	1	2	3	4	5	6	7	8	9	10	11	12
A												
B												
C												
D												

0	T2	C01	0	0	kleiner LLOQ	#VALUE!	1	#VALUE!
0	T2	C02	0	0	kleiner LLOQ	#VALUE!	1	#VALUE!
0	T2	C03	0	0	kleiner LLOQ	#VALUE!	1	#VALUE!
0	T2	C04	0	0	kleiner LLOQ	#VALUE!	1	#VALUE!
0	T2	C05	0	0	kleiner LLOQ	#VALUE!	1	#VALUE!
0	T2	C06	0	0	kleiner LLOQ	#VALUE!	1	#VALUE!
0	T2	C07	0	0	kleiner LLOQ	#VALUE!	1	#VALUE!
0	T2	C08	0	0	kleiner LLOQ	#VALUE!	1	#VALUE!
0	T2	C09	0	0	kleiner LLOQ	#VALUE!	1	#VALUE!
0	T2	C10	0	0	kleiner LLOQ	#VALUE!	1	#VALUE!
0	T2	C11	0	0	kleiner LLOQ	#VALUE!	1	#VALUE!
0	T2	C12	0	0	kleiner LLOQ	#VALUE!	1	#VALUE!
0	T2	D01	0	0	kleiner LLOQ	#VALUE!	1	#VALUE!
0	T2	D02	0	0	kleiner LLOQ	#VALUE!	1	#VALUE!
0	T2	D03	0	0	kleiner LLOQ	#VALUE!	1	#VALUE!
0	T2	D04	0	0	kleiner LLOQ	#VALUE!	1	#VALUE!
0	T2	D05	0	0	kleiner LLOQ	#VALUE!	1	#VALUE!
0	T2	D06	0	0	kleiner LLOQ	#VALUE!	1	#VALUE!
0	T2	D07	0	0	kleiner LLOQ	#VALUE!	1	#VALUE!
0	T2	D08	0	0	kleiner LLOQ	#VALUE!	1	#VALUE!
0	T2	D09	0	0	kleiner LLOQ	#VALUE!	1	#VALUE!
0	T2	D10	0	0	kleiner LLOQ	#VALUE!	1	#VALUE!
0	T2	D11	0	0	kleiner LLOQ	#VALUE!	1	#VALUE!
0	T2	D12	0	0	kleiner LLOQ	#VALUE!	1	#VALUE!
0	T2	E01	0	0	kleiner LLOQ	#VALUE!	1	#VALUE!
0	T2	E02	0	0	kleiner LLOQ	#VALUE!	1	#VALUE!
0	T2	E03	0	0	kleiner LLOQ	#VALUE!	1	#VALUE!
0	T2	E04	0	0	kleiner LLOQ	#VALUE!	1	#VALUE!
0	T2	E05	0	0	kleiner LLOQ	#VALUE!	1	#VALUE!
0	T2	E06	0	0	kleiner LLOQ	#VALUE!	1	#VALUE!
T2SS12	T2	E07	0.574	0.523	0.523	0.023	22	0.512
T2SS12	T2	E08	0.573	0.522	0.522	0.023	22	0.511
T2SS13	T2	E09	0.572	0.522	0.522	0.023	22	0.510
T2SS13	T2	E10	0.589	0.538	0.538	0.024	22	0.527
0	T2	E11	0	0	kleiner LLOQ	#VALUE!	1	#VALUE!
0	T2	E12	0	0	kleiner LLOQ	#VALUE!	1	#VALUE!
0	T2	F01	0	0	kleiner LLOQ	#VALUE!	1	#VALUE!
0	T2	F02	0	0	kleiner LLOQ	#VALUE!	1	#VALUE!
0	T2	F03	0	0	kleiner LLOQ	#VALUE!	1	#VALUE!
0	T2	F04	0	0	kleiner LLOQ	#VALUE!	1	#VALUE!
T2SS21	T2	F05	0.606	0.556	0.556	0.025	22	0.546
T2SS21	T2	F06	0.608	0.557	0.557	0.025	22	0.547
0	T2	F07	0	0	kleiner LLOQ	#VALUE!	1	#VALUE!
0	T2	F08	0	0	kleiner LLOQ	#VALUE!	1	#VALUE!
0	T2	F09	0	0	kleiner LLOQ	#VALUE!	1	#VALUE!
0	T2	F10	0	0	kleiner LLOQ	#VALUE!	1	#VALUE!
0	T2	F11	0	0	kleiner LLOQ	#VALUE!	1	#VALUE!
0	T2	F12	0	0	kleiner LLOQ	#VALUE!	1	#VALUE!
0	T2	G01	0	0	kleiner LLOQ	#VALUE!	1	#VALUE!
0	T2	G02	0	0	kleiner LLOQ	#VALUE!	1	#VALUE!
T2SS11	T2	G03	0.511	0.460	0.460	0.020	16.5	0.334
T2SS11	T2	G04	0.494	0.443	0.443	0.019	16.5	0.321
T2SS22	T2	G05	0.744	0.694	0.694	0.031	16.5	0.517
T2SS22	T2	G06	0.774	0.723	0.723	0.033	16.5	0.541
Blank PEG	T2	G07	0	0	kleiner LLOQ	#VALUE!	2.75	#VALUE!
Blank PEG	T2	G08	0	0	kleiner LLOQ	#VALUE!	2.75	#VALUE!
Blank PEG	T2	G09	0	0	kleiner LLOQ	#VALUE!	2.75	#VALUE!
Blank PEG	T2	G10	0	0	kleiner LLOQ	#VALUE!	2.75	#VALUE!
Blank PEG	T2	G11	0	0	kleiner LLOQ	#VALUE!	2.75	#VALUE!
Blank PEG	T2	G12	0	0	kleiner LLOQ	#VALUE!	2.75	#VALUE!
T2SS23	T2	H01	0.503	0.452	0.452	0.020	16.5	0.328
T2SS23	T2	H02	0.559	0.508	0.508	0.023	16.5	0.372
T2SS24	T2	H03	0.312	0.261	0.261	0.011	16.5	0.178
T2SS24	T2	H04	0.313	0.262	0.262	0.011	16.5	0.179
T2SN2	T2	H05	0	0	0	0.01	22	0.223
T2SN2	T2	H06	0	0	0	0.01	22	0.173
Blank no PEG	T2	H07	0	0	kleiner LLOQ	#VALUE!	2.75	#VALUE!
Blank no PEG	T2	H08	0	0	kleiner LLOQ	#VALUE!	2.75	#VALUE!
Blank no PEG	T2	H09	0	0	kleiner LLOQ	#VALUE!	2.75	#VALUE!
Blank no PEG	T2	H10	0	0	kleiner LLOQ	#VALUE!	2.75	#VALUE!
Blank no PEG	T2	H11	0	0	kleiner LLOQ	#VALUE!	2.75	#VALUE!
Blank no PEG	T2	H12	0	0	kleiner LLOQ	#VALUE!	2.75	#VALUE!
0	T3	A01	0	0	kleiner LLOQ	#VALUE!	1	#VALUE!
0	T3	A02	0	0	kleiner LLOQ	#VALUE!	1	#VALUE!
0	T3	A03	0	0	kleiner LLOQ	#VALUE!	1	#VALUE!

E							T2SS12	T2SS12	T2SS13	T2SS13		
F					T2SS21	T2SS21						
G			T2SS11	T2SS11	T2SS22	T2SS22	Blank PEG	Blank PEG	Blank PEG	Blank PEG	Blank PEG	Blank PEG
H	T2SS23	T2SS23	T2SS24	T2SS24	T2SN2	T2SN2	Blank no PEG	Blank no PEG	Blank no PEG	Blank no PEG	Blank no PEG	Blank no PEG

dil.8*dil.2,75=dil.22

dil.6*dil.2,75=dil.16,5

Comment:

Date Operator

Date Control

0	T3	A04	0	0	kleiner LLOQ	#VALUE!	1	#VALUE!
0	T3	A05	0	0	kleiner LLOQ	#VALUE!	1	#VALUE!
0	T3	A06	0	0	kleiner LLOQ	#VALUE!	1	#VALUE!
0	T3	A07	0	0	kleiner LLOQ	#VALUE!	1	#VALUE!
0	T3	A08	0	0	kleiner LLOQ	#VALUE!	1	#VALUE!
0	T3	A09	0	0	kleiner LLOQ	#VALUE!	1	#VALUE!
0	T3	A10	0	0	kleiner LLOQ	#VALUE!	1	#VALUE!
0	T3	A11	0	0	kleiner LLOQ	#VALUE!	1	#VALUE!
0	T3	A12	0	0	kleiner LLOQ	#VALUE!	1	#VALUE!
0	T3	B01	0	0	kleiner LLOQ	#VALUE!	1	#VALUE!
0	T3	B02	0	0	kleiner LLOQ	#VALUE!	1	#VALUE!
0	T3	B03	0	0	kleiner LLOQ	#VALUE!	1	#VALUE!
0	T3	B04	0	0	kleiner LLOQ	#VALUE!	1	#VALUE!
0	T3	B05	0	0	kleiner LLOQ	#VALUE!	1	#VALUE!
0	T3	B06	0	0	kleiner LLOQ	#VALUE!	1	#VALUE!
0	T3	B07	0	0	kleiner LLOQ	#VALUE!	1	#VALUE!
0	T3	B08	0	0	kleiner LLOQ	#VALUE!	1	#VALUE!
0	T3	B09	0	0	kleiner LLOQ	#VALUE!	1	#VALUE!
0	T3	B10	0	0	kleiner LLOQ	#VALUE!	1	#VALUE!
0	T3	B11	0	0	kleiner LLOQ	#VALUE!	1	#VALUE!
0	T3	B12	0	0	kleiner LLOQ	#VALUE!	1	#VALUE!
0	T3	C01	0	0	kleiner LLOQ	#VALUE!	1	#VALUE!
0	T3	C02	0	0	kleiner LLOQ	#VALUE!	1	#VALUE!
0	T3	C03	0	0	kleiner LLOQ	#VALUE!	1	#VALUE!
0	T3	C04	0	0	kleiner LLOQ	#VALUE!	1	#VALUE!
0	T3	C05	0	0	kleiner LLOQ	#VALUE!	1	#VALUE!
0	T3	C06	0	0	kleiner LLOQ	#VALUE!	1	#VALUE!
0	T3	C07	0	0	kleiner LLOQ	#VALUE!	1	#VALUE!
0	T3	C08	0	0	kleiner LLOQ	#VALUE!	1	#VALUE!
0	T3	C09	0	0	kleiner LLOQ	#VALUE!	1	#VALUE!
0	T3	C10	0	0	kleiner LLOQ	#VALUE!	1	#VALUE!
0	T3	C11	0	0	kleiner LLOQ	#VALUE!	1	#VALUE!
0	T3	C12	0	0	kleiner LLOQ	#VALUE!	1	#VALUE!
0	T3	D01	0	0	kleiner LLOQ	#VALUE!	1	#VALUE!
0	T3	D02	0	0	kleiner LLOQ	#VALUE!	1	#VALUE!
0	T3	D03	0	0	kleiner LLOQ	#VALUE!	1	#VALUE!
0	T3	D04	0	0	kleiner LLOQ	#VALUE!	1	#VALUE!
0	T3	D05	0	0	kleiner LLOQ	#VALUE!	1	#VALUE!
0	T3	D06	0	0	kleiner LLOQ	#VALUE!	1	#VALUE!
T3SN2	T3	D07	0.410	0.359	0.359	0.015	24.75	0.383
T3SN2	T3	D08	0.080	0.029	kleiner LLOQ	#VALUE!	24.75	#VALUE!
T3SN3	T3	D09	0.262	0.211	0.211	0.008	24.75	0.208
T3SN3	T3	D10	0.231	0.180	0.180	0.007	24.75	0.172
T3SN4	T3	D11	0.503	0.452	0.452	0.020	11	0.219
T3SN4	T3	D12	0.547	0.496	0.496	0.022	11	0.242
0	T3	E01	0	0	kleiner LLOQ	#VALUE!	1	#VALUE!
0	T3	E02	0	0	kleiner LLOQ	#VALUE!	1	#VALUE!
0	T3	E03	0	0	kleiner LLOQ	#VALUE!	1	#VALUE!
0	T3	E04	0	0	kleiner LLOQ	#VALUE!	1	#VALUE!
T3SS11	T3	E05	0.557	0.506	0.506	0.022	11	0.247
T3SS11	T3	E06	0.530	0.479	0.479	0.021	11	0.233
T3SS12	T3	E07	0.621	0.570	0.570	0.025	24.75	0.631
T3SS12	T3	E08	0.561	0.510	0.510	0.023	24.75	0.560
T3SS13	T3	E09	0.682	0.631	0.631	0.028	11	0.312
T3SS13	T3	E10	0.634	0.584	0.584	0.026	11	0.287
T3SS14	T3	E11	0.414	0.363	0.363	0.016	11	0.172
T3SS14	T3	E12	0.402	0.351	0.351	0.015	11	0.166
0	T3	F01	0	0	kleiner LLOQ	#VALUE!	1	#VALUE!
0	T3	F02	0	0	kleiner LLOQ	#VALUE!	1	#VALUE!
0	T3	F03	0	0	kleiner LLOQ	#VALUE!	1	#VALUE!
0	T3	F04	0	0	kleiner LLOQ	#VALUE!	1	#VALUE!
T3SS21	T3	F05	0.475	0.424	0.424	0.019	11	0.204
T3SS21	T3	F06	0.468	0.417	0.417	0.018	11	0.200
T3SS22	T3	F07	0.368	0.317	0.317	0.013	11	0.148
T3SS22	T3	F08	0.400	0.349	0.349	0.015	11	0.165
T3SS23	T3	F09	0	0	kleiner LLOQ	#VALUE!	1	#VALUE!
T3SS23	T3	F10	0	0	kleiner LLOQ	#VALUE!	1	#VALUE!
T3SS24	T3	F11	0	0	kleiner LLOQ	#VALUE!	1	#VALUE!
T3SS24	T3	F12	0	0	kleiner LLOQ	#VALUE!	1	#VALUE!
0	T3	G01	0	0	kleiner LLOQ	#VALUE!	1	#VALUE!
0	T3	G02	0	0	kleiner LLOQ	#VALUE!	1	#VALUE!
0	T3	G03	0	0	kleiner LLOQ	#VALUE!	1	#VALUE!
0	T3	G04	0	0	kleiner LLOQ	#VALUE!	1	#VALUE!

Plate: T3

	1	2	3	4	5	6	7	8	9	10	11	12
A												
B												
C												
D							0.410	0.080	0.262	0.231	0.503	0.547
E					0.557	0.530	0.621	0.561	0.682	0.634	0.414	0.402
F					0.475	0.468	0.368	0.400				
G							0.052	0.048	0.052	0.052	0.047	0.046
H							0.051	0.060	0.059	0.054	0.055	0.055

Plattenbelegung

	1	2	3	4	5	6	7	8	9	10	11	12
A												
B												
C												
D							T3SN2	T3SN2	T3SN3	T3SN3	T3SN4	T3SN4
E					T3SS11	T3SS11	T3SS12	T3SS12	T3SS13	T3SS13	T3SS14	T3SS14
F					T3SS21	T3SS21	T3SS22	T3SS22	T3SS23	T3SS23	T3SS24	T3SS24
G							Blank PEG	Blank PEG	Blank PEG	Blank PEG	Blank PEG	Blank PEG
H							Blank no PEG	Blank no PEG	Blank no PEG	Blank no PEG	Blank no PEG	Blank no PEG

dil9*dil2,75=24,75
dil4*dil2,75=dil11

Comment:

Date Operator

Date Control

0	T3	G05	0	0	kleiner LLOQ	#VALUE!	1	#VALUE!
0	T3	G06	0	0	kleiner LLOQ	#VALUE!	1	#VALUE!
Blank PEG	T3	G07	0	0	kleiner LLOQ	#VALUE!	2.75	#VALUE!
Blank PEG	T3	G08	0	0	kleiner LLOQ	#VALUE!	2.75	#VALUE!
Blank PEG	T3	G09	0	0	kleiner LLOQ	#VALUE!	2.75	#VALUE!
Blank PEG	T3	G10	0	0	kleiner LLOQ	#VALUE!	2.75	#VALUE!
Blank PEG	T3	G11	0	0	kleiner LLOQ	#VALUE!	2.75	#VALUE!
Blank PEG	T3	G12	0	0	kleiner LLOQ	#VALUE!	2.75	#VALUE!
0	T3	H01	0	0	kleiner LLOQ	#VALUE!	1	#VALUE!
0	T3	H02	0	0	kleiner LLOQ	#VALUE!	1	#VALUE!
0	T3	H03	0	0	kleiner LLOQ	#VALUE!	1	#VALUE!
0	T3	H04	0	0	kleiner LLOQ	#VALUE!	1	#VALUE!
0	T3	H05	0	0	kleiner LLOQ	#VALUE!	1	#VALUE!
0	T3	H06	0	0	kleiner LLOQ	#VALUE!	1	#VALUE!
Blank no PEG	T3	H07	0	0	kleiner LLOQ	#VALUE!	2.75	#VALUE!
Blank no PEG	T3	H08	0	0	kleiner LLOQ	#VALUE!	2.75	#VALUE!
Blank no PEG	T3	H09	0	0	kleiner LLOQ	#VALUE!	2.75	#VALUE!
Blank no PEG	T3	H10	0	0	kleiner LLOQ	#VALUE!	2.75	#VALUE!
Blank no PEG	T3	H11	0	0	kleiner LLOQ	#VALUE!	2.75	#VALUE!
Blank no PEG	T3	H12	0	0	kleiner LLOQ	#VALUE!	2.75	#VALUE!

**Project number**		F-120	**Apparatus**	**Wallac Victor**	**Operator**	**IsBa**
**GLP Study (Number)**		n.a.	**Protocol (Instrument meth**	**LDH test 2016**	**Date of preparation**	**19-04-18**
**hot substance**	**isotope**	n.a.	**File name (results)**	**IsBa_180419/20_LDH_full_**	**Date of measurement**	**19-04-18**
	**name**	n.a.	**Kind of well plate**	**96 well**	**shaking time [min]**	**30**
	**ACB-ID**	n.a.	**sample volume [µL]**	**100**	**stirring rate (Target) [rpm]**	**150**
	**Batch number**	n.a.	**Cocktail volume [µL]**	**175**	**Kind of measurement**	**UV-vis**
**cold substance**	**name**	LDH	**ACB-ID of cocktail**		**Wave length [nm]**	**450**
	**ACB-ID**	L23982R	**Matrix**	**DMEM (from powder)+PE(**	**Remarks**	**Cocktail 100µl RM, 75µl STOP**
	**Batch number**	SLBK6345V	**Blank description**	**DMEM/PEG, H2O**	**Remarks**	**7 standards split low/high**
**n.a.**		n.a.	**Pipettes (No. / volume)**	**50-200µl**	**Remarks**	**KLP4 common for both**
**n.a.**		n.a.	**Pipettes (No. / volume)**	**n.a.**	**Remarks**	**n.a.**

Messdaten (diese Tabelle in Bericht übernehmen)

Sample name *	concentration (theor.) *	measured data	measured data	measured data	mean measured	SD	RSD	Blank *	measured data after * Blank subtraction	concentration (calc.) *	Deviation *	Residuen
	[µg/mL]	[AU]	[AU]	[AU]	[AU]	[AU]	[%]	[AU]		[µg/mL]	[%]	
KLP1	0.148	1.579	1.652	1.627	1.62	0.03	1.87	0.047	1.568	0.148	-0.24	0.00
KLP2	0.114	1.357	1.443	1.372	1.39	0.04	2.70	0.054	1.340	0.110	-3.40	0.00
KLP3	0.074	1.272	1.193	1.228	1.23	0.03	2.63	0.051	1.180	0.084	13.04	0.01
KLP4	0.041	0.959	0.972	0.883	0.94	0.04	4.19	0.051	0.887	0.035	-13.32	-0.01
KLP5								0.052				
KLP6								0.051				
KLP7												
KLP8												

Statistical data

Geradensteigung	Slope	m	6.05
Y-Achsenabschnitt	Y-Intercept	b	0.67
Standardabw. Geradensteigung	SD-Slope	s_m	0.620291227
Standardabw. Achsenabschnittes	SD-Y-Intercept	s_b	0.063694294
Anzahl Messpunkte	number of measuring points	n	4
Quadratsumme	sum of squares	Qxx	0.006578604
Bereichsmitte			0.094336926
Freiheitsgrade	degree of freedom	f	2
Student-t-Faktor für (P = 95 %; f = n-2)	Student-t-factor for (P = 95 %; f = n-2)	t	4.303
Vertrauensbereich Steig. (95 %) Obergrenze		$m + VB_m$	8.722319527
Vertrauensbereich Steig. (95 %) Untergrenze		$m - VB_m$	3.384093228
Vertrauensbereich Achsenabschnitt (95 %) Og.		$b + Vb_b$	0.946914615
Vertrauensbereich Achsenabschnitt (95 %) Ug.		$b - Vb_b$	0.398761518
Korrelationskoeffizient	correlation coefficient	r	0.9897
Bestimmtheitsmaß	determiantion coefficient	r^2	0.9794
Reststandardabweichung		s_0	0.050310948
Summe Restquadrate		sd	2.895583345
Verfahrensstandardabw.		s_{x0}	0.008311454
Rel. Verfahrensstandardabw. %		V_{x0}	8.810393374

mean Blank	**0**	
SD Blank	0.00	
RSD Blank	3.79	x=
x*SD (LLOQ)	0	5
x*SD (LOD)	0	3
**LLOQ (AU)**	**0**	
**LOD (AU)**	**0**	
ULOQ	1.568	
LLOQ (Lin)	0.887	

Evaluation / Comment

LDH linearity valid with 7 standards, split in lin high and lin low, each with 4 standards and KLP4 common standard for both. R² 0,9981, deviations for both between -13.34% and +13.04%

Date Operator Date Control

Across Barriers GmbH
Science Park 1
66123 Saarbrücken

Tel: 0681 95918-800
Fax: 0681 95918-802
e-Mail: sales@acrossbarriers.de
http://www.acrossbarriers.de

Formblatt-Nr.		Version	
Titel			
Gültig ab		Ablage	Projektordner

Figure 1 Linearity

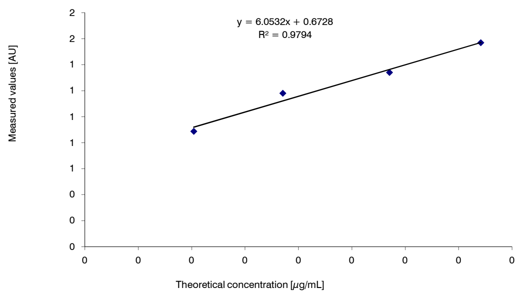

Figure 2 Method validation Residuen Plot

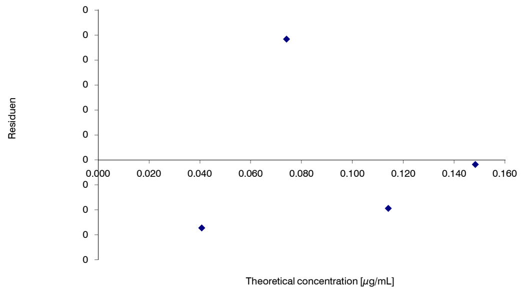

Evaluation / Comment

lin passed

Date	Operator	Date	Control

Sample name	Plate	Position	[AU]	[AU]-Blank	Rating	[µg/mL]	Dilution factor	[µg/mL]
T04SN1	T0	A01	1.198	1.147	1.147	0.078	66	5.175
T04SN1	T0	A02	1.291	1.240	1.240	0.094	66	6.184
T04SN2	T0	A03	1.335	1.284	1.284	0.101	66	6.666
T04SN2	T0	A04	1.239	1.188	1.188	0.085	66	5.618
T04SN3	T0	A05	1.265	1.214	1.214	0.089	66	5.897
T04SN3	T0	A06	1.305	1.254	1.254	0.096	66	6.332
0	T0	A07	0	0	kleiner LLOQ	#VALUE!	1	#VALUE!
0	T0	A08	0	0	kleiner LLOQ	#VALUE!	1	#VALUE!
0	T0	A09	0	0	kleiner LLOQ	#VALUE!	22	#VALUE!
0	T0	A10	0	0	kleiner LLOQ	#VALUE!	22	#VALUE!
0	T0	A11	0	0	kleiner LLOQ	#VALUE!	1	#VALUE!
0	T0	A12	0	0	kleiner LLOQ	#VALUE!	1	#VALUE!
T04SS11	T0	B01	1.302	1.251	1.251	0.095	66	6.303
T04SS11	T0	B02	1.349	1.298	1.298	0.103	66	6.813
T04SS12	T0	B03	1.214	1.163	1.163	0.081	66	5.342
T04SS12	T0	B04	1.161	1.110	1.110	0.072	66	4.769
T04SS13	T0	B05	1.334	1.283	1.283	0.101	66	6.653
T04SS13	T0	B06	1.291	1.240	1.240	0.094	66	6.181
0	T0	B07	0	0	kleiner LLOQ	#VALUE!	66	#VALUE!
0	T0	B08	0	0	kleiner LLOQ	#VALUE!	66	#VALUE!
0	T0	B09	0	0	kleiner LLOQ	#VALUE!	1	#VALUE!
0	T0	B10	0	0	kleiner LLOQ	#VALUE!	1	#VALUE!
0	T0	B11	0	0	kleiner LLOQ	#VALUE!	1	#VALUE!
0	T0	B12	0	0	kleiner LLOQ	#VALUE!	1	#VALUE!
T04SS21	T0	C01	1.262	1.211	1.211	0.089	66	5.870
T04SS21	T0	C02	1.442	1.391	1.391	0.119	66	7.826
T04SS22	T0	C03	1.168	1.117	1.117	0.073	66	4.842
T04SS22	T0	C04	1.090	1.039	1.039	0.060	66	3.992
T04SS23	T0	C05	1.247	1.196	1.196	0.086	66	5.701
T04SS23	T0	C06	1.062	1.011	1.011	0.056	66	3.683
0	T0	C07	0	0	kleiner LLOQ	#VALUE!	66	#VALUE!
0	T0	C08	0	0	kleiner LLOQ	#VALUE!	66	#VALUE!
0	T0	C09	0	0	kleiner LLOQ	#VALUE!	66	#VALUE!
0	T0	C10	0	0	kleiner LLOQ	#VALUE!	66	#VALUE!
0	T0	C11	0	0	kleiner LLOQ	#VALUE!	1	#VALUE!
0	T0	C12	0	0	kleiner LLOQ	#VALUE!	1	#VALUE!
0	T0	D01	0	0	kleiner LLOQ	#VALUE!	1	#VALUE!
0	T0	D02	0	0	kleiner LLOQ	#VALUE!	1	#VALUE!
0	T0	D03	0	0	kleiner LLOQ	#VALUE!	1	#VALUE!
0	T0	D04	0	0	kleiner LLOQ	#VALUE!	1	#VALUE!
0	T0	D05	0	0	kleiner LLOQ	#VALUE!	1	#VALUE!
0	T0	D06	0	0	kleiner LLOQ	#VALUE!	1	#VALUE!
0	T0	D07	0	0	kleiner LLOQ	#VALUE!	1	#VALUE!
0	T0	D08	0	0	kleiner LLOQ	#VALUE!	1	#VALUE!
0	T0	D09	0	0	kleiner LLOQ	#VALUE!	1	#VALUE!
0	T0	D10	0	0	kleiner LLOQ	#VALUE!	1	#VALUE!
T04SN4	T0	D11	0.951	0.900	0.900	0.038	11	0.413
T04SN4	T0	D12	0	0	kleiner LLOQ	#VALUE!	1	#VALUE!

Plate:	T0

	1	2	3	4	5	6	7	8	9	10	11	12
A	1.198	1.291	1.335	1.239	1.265	1.305						
B	1.302	1.349	1.214	1.161	1.334	1.291						
C	1.262	1.442	1.168	1.090	1.247	1.062						
D											0.951	
E												
F												
G	0.065	0.066	0.062	0.072	0.060	0.070	0.056	0.233	0.207	0.213	0.063	0.053
H	0.044	0.045	0.048	0.046	0.047	0.044	0.052	0.050	0.112	0.060	0.050	0.048

Plattenbelegung

	1	2	3	4	5	6	7	8	9	10	11	12
A	T04SN1	T04SN1	T04SN2	T04SN2	T04SN3	T04SN3						
B	T04SS11	T04SS11	T04SS12	T04SS12	T04SS13	T04SS13						
C	T04SS21	T04SS21	T04SS22	T04SS22	T04SS23	T04SS23						
D											T04SN4	T04SN4
E											T04SS14	T04SS14
F											T04SS24	T04SS24
G	Blank PEG	Blank PEG	Blank PEG	Blank PEG	Blank PEG	Blank PEG	Blank PEG	Blank PEG	Blank PEG	Blank PEG	Blank PEG	Blank PEG
H	Blank no PEG	Blank no PEG	Blank no PEG	Blank no PEG	Blank no PEG	Blank no PEG	Blank no PEG	Blank no PEG	Blank no PEG	Blank no PEG	Blank no PEG	Blank no PEG

dil.4*dil2,75=dil.11

dil4*dil.8*dil.2,75=dil.88 oder nur von ursprüngl. Dil 4 auf dil 8 erhöht??? dann dil 22

Comment:

Erste 4 Reihen immer Testmessung zur Evaluierung der benötigten Verdünnung. Wird nicht mit ausgewertet. Unverdünnte Messungen außerhalb der Linearität werden ebenfalls nicht ausgewertet. Dil.Faktor unverdünnte samples=2,75 (100µl sample+100µlRM+75µl stop sol.) Verdünnung Dil.8 Dil8*Dil2,75=Dil.22, Rot= 2* verdünnt, Dil4 und Dil 8, also Dil 2,75*Dil8*Dil3=Dil66

Samples aufgeteilt in lin high und low:niedriger konzentrierte Samples bei lin low zu finden.

Date Operator

Date Control

0	T0	E01	0	0	kleiner LLOQ	#VALUE!	1	#VALUE!
0	T0	E02	0	0	kleiner LLOQ	#VALUE!	1	#VALUE!
0	T0	E03	0	0	kleiner LLOQ	#VALUE!	1	#VALUE!
0	T0	E04	0	0	kleiner LLOQ	#VALUE!	1	#VALUE!
0	T0	E05	0	0	kleiner LLOQ	#VALUE!	1	#VALUE!
0	T0	E06	0	0	kleiner LLOQ	#VALUE!	1	#VALUE!
0	T0	E07	0	0	kleiner LLOQ	#VALUE!	1	#VALUE!
0	T0	E08	0	0	kleiner LLOQ	#VALUE!	1	#VALUE!
0	T0	E09	0	0	kleiner LLOQ	#VALUE!	1	#VALUE!
0	T0	E10	0	0	kleiner LLOQ	#VALUE!	1	#VALUE!
T04SS14	T0	E11	0	0	kleiner LLOQ	#VALUE!	1	#VALUE!
T04SS14	T0	E12	0	0	kleiner LLOQ	#VALUE!	1	#VALUE!
0	T0	F01	0	0	kleiner LLOQ	#VALUE!	1	#VALUE!
0	T0	F02	0	0	kleiner LLOQ	#VALUE!	1	#VALUE!
0	T0	F03	0	0	kleiner LLOQ	#VALUE!	1	#VALUE!
0	T0	F04	0	0	kleiner LLOQ	#VALUE!	1	#VALUE!
0	T0	F05	0	0	kleiner LLOQ	#VALUE!	1	#VALUE!
0	T0	F06	0	0	kleiner LLOQ	#VALUE!	1	#VALUE!
0	T0	F07	0	0	kleiner LLOQ	#VALUE!	1	#VALUE!
0	T0	F08	0	0	kleiner LLOQ	#VALUE!	1	#VALUE!
0	T0	F09	0	0	kleiner LLOQ	#VALUE!	1	#VALUE!
0	T0	F10	0	0	kleiner LLOQ	#VALUE!	1	#VALUE!
T04SS24	T0	F11	0.000	-0.051	kleiner LLOQ	#VALUE!	11	#VALUE!
T04SS24	T0	F12	0	0	kleiner LLOQ	#VALUE!	1	#VALUE!
Blank PEG	T0	G01	0	0	kleiner LLOQ	#VALUE!	1	#VALUE!
Blank PEG	T0	G02	0	0	kleiner LLOQ	#VALUE!	1	#VALUE!
Blank PEG	T0	G03	0	0	kleiner LLOQ	#VALUE!	1	#VALUE!
Blank PEG	T0	G04	0	0	kleiner LLOQ	#VALUE!	1	#VALUE!
Blank PEG	T0	G05	0	0	kleiner LLOQ	#VALUE!	1	#VALUE!
Blank PEG	T0	G06	0	0	kleiner LLOQ	#VALUE!	1	#VALUE!
Blank PEG	T0	G07	0.056	0.005	kleiner LLOQ	#VALUE!	2.75	#VALUE!
Blank PEG	T0	G08	0.233	0.182	kleiner LLOQ	#VALUE!	2.75	#VALUE!
Blank PEG	T0	G09	0.207	0.156	kleiner LLOQ	#VALUE!	2.75	#VALUE!
Blank PEG	T0	G10	0.213	0.162	kleiner LLOQ	#VALUE!	2.75	#VALUE!
Blank PEG	T0	G11	0.063	0.012	kleiner LLOQ	#VALUE!	2.75	#VALUE!
Blank PEG	T0	G12	0.053	0.002	kleiner LLOQ	#VALUE!	2.75	#VALUE!
Blank no PEG	T0	H01	0	0	kleiner LLOQ	#VALUE!	1	#VALUE!
Blank no PEG	T0	H02	0	0	kleiner LLOQ	#VALUE!	1	#VALUE!
Blank no PEG	T0	H03	0	0	kleiner LLOQ	#VALUE!	1	#VALUE!
Blank no PEG	T0	H04	0	0	kleiner LLOQ	#VALUE!	1	#VALUE!
Blank no PEG	T0	H05	0	0	kleiner LLOQ	#VALUE!	1	#VALUE!
Blank no PEG	T0	H06	0	0	kleiner LLOQ	#VALUE!	1	#VALUE!
Blank no PEG	T0	H07	0.052	0.001	kleiner LLOQ	#VALUE!	2.75	#VALUE!
Blank no PEG	T0	H08	0.050	-0.001	kleiner LLOQ	#VALUE!	2.75	#VALUE!
Blank no PEG	T0	H09	0.112	0.061	kleiner LLOQ	#VALUE!	2.75	#VALUE!
Blank no PEG	T0	H10	0.060	0.009	kleiner LLOQ	#VALUE!	2.75	#VALUE!
Blank no PEG	T0	H11	0.050	-0.001	kleiner LLOQ	#VALUE!	2.75	#VALUE!
Blank no PEG	T0	H12	0.048	-0.003	kleiner LLOQ	#VALUE!	2.75	#VALUE!

0	T1	A01	0	0	kleiner LLOQ	#VALUE!	1	#VALUE!
0	T1	A02	0	0	kleiner LLOQ	#VALUE!	1	#VALUE!
0	T1	A03	0	0	kleiner LLOQ	#VALUE!	1	#VALUE!
0	T1	A04	0	0	kleiner LLOQ	#VALUE!	1	#VALUE!
0	T1	A05	0	0	kleiner LLOQ	#VALUE!	1	#VALUE!
0	T1	A06	0	0	kleiner LLOQ	#VALUE!	1	#VALUE!
0	T1	A07	0	0	kleiner LLOQ	#VALUE!	1	#VALUE!
0	T1	A08	0	0	kleiner LLOQ	#VALUE!	1	#VALUE!
0	T1	A09	0	0	kleiner LLOQ	#VALUE!	1	#VALUE!
0	T1	A10	0	0	kleiner LLOQ	#VALUE!	1	#VALUE!
0	T1	A11	0	0	kleiner LLOQ	#VALUE!	1	#VALUE!
0	T1	A12	0	0	kleiner LLOQ	#VALUE!	1	#VALUE!
0	T1	B01	0	0	kleiner LLOQ	#VALUE!	1	#VALUE!
0	T1	B02	0	0	kleiner LLOQ	#VALUE!	1	#VALUE!
0	T1	B03	0	0	kleiner LLOQ	#VALUE!	1	#VALUE!
0	T1	B04	0	0	kleiner LLOQ	#VALUE!	1	#VALUE!
0	T1	B05	0	0	kleiner LLOQ	#VALUE!	1	#VALUE!
0	T1	B06	0	0	kleiner LLOQ	#VALUE!	1	#VALUE!
0	T1	B07	0	0	kleiner LLOQ	#VALUE!	1	#VALUE!
0	T1	B08	0	0	kleiner LLOQ	#VALUE!	1	#VALUE!
0	T1	B09	0	0	kleiner LLOQ	#VALUE!	1	#VALUE!
0	T1	B10	0	0	kleiner LLOQ	#VALUE!	1	#VALUE!
0	T1	B11	0	0	kleiner LLOQ	#VALUE!	1	#VALUE!
0	T1	B12	0	0	kleiner LLOQ	#VALUE!	1	#VALUE!
0	T1	C01	0	0	kleiner LLOQ	#VALUE!	1	#VALUE!
0	T1	C02	0	0	kleiner LLOQ	#VALUE!	1	#VALUE!
0	T1	C03	0	0	kleiner LLOQ	#VALUE!	1	#VALUE!
0	T1	C04	0	0	kleiner LLOQ	#VALUE!	1	#VALUE!
0	T1	C05	0	0	kleiner LLOQ	#VALUE!	1	#VALUE!
0	T1	C06	0	0	kleiner LLOQ	#VALUE!	1	#VALUE!
0	T1	C07	0	0	kleiner LLOQ	#VALUE!	1	#VALUE!
0	T1	C08	0	0	kleiner LLOQ	#VALUE!	1	#VALUE!
0	T1	C09	0	0	kleiner LLOQ	#VALUE!	1	#VALUE!
0	T1	C10	0	0	kleiner LLOQ	#VALUE!	1	#VALUE!
0	T1	C11	0	0	kleiner LLOQ	#VALUE!	1	#VALUE!
0	T1	C12	0	0	kleiner LLOQ	#VALUE!	1	#VALUE!
0	T1	D01	0	0	kleiner LLOQ	#VALUE!	1	#VALUE!
0	T1	D02	0	0	kleiner LLOQ	#VALUE!	1	#VALUE!
0	T1	D03	0	0	kleiner LLOQ	#VALUE!	1	#VALUE!
0	T1	D04	0	0	kleiner LLOQ	#VALUE!	1	#VALUE!
0	T1	D05	0	0	kleiner LLOQ	#VALUE!	1	#VALUE!
0	T1	D06	0	0	kleiner LLOQ	#VALUE!	1	#VALUE!
T1SN2	T1	D07	1.016	0.965	0.965	0.048	24.75	1.196
T1SN2	T1	D08	1.042	0.991	0.991	0.053	24.75	1.302
0	T1	D09	0	0	kleiner LLOQ	#VALUE!	1	#VALUE!
0	T1	D10	0	0	kleiner LLOQ	#VALUE!	1	#VALUE!
0	T1	D11	0	0	kleiner LLOQ	#VALUE!	1	#VALUE!
0	T1	D12	0	0	kleiner LLOQ	#VALUE!	1	#VALUE!

Plate: T1

	1	2	3	4	5	6	7	8	9	10	11	12
A												
B												
C												
D							1.016	1.042				
E					0.964	1.027						
F					0.965	1.043	1.423	1.549				
G							0.065	0.053	0.052	0.056	0.050	0.055
H							0.051	0.051	0.051	0.052	0.052	0.051

Plattenbelegung

	1	2	3	4	5	6	7	8	9	10	11	12
A												
B												
C												
D							T1SN2	T1SN2				
E					T1SS11	T1SS11						
F					T1SS21	T1SS21	T1SS22	T1SS22				
G							Blank PEG	Blank PEG	Blank PEG	Blank PEG	Blank PEG	Blank PEG
H							Blank no PEG	Blank no PEG	Blank no PEG	Blank no PEG	Blank no PEG	Blank no PEG

dil.5*dil.2,75=dil.13,75
dil.9*dil.2,75=dil.24,75
dil.9*dil.4*dil.2,75=dil.99
dil.9*dil.9*dil.2,75=dil.222,75

Comment:

Date Operator

Date Control

0	T1	E01	0	0	kleiner LLOQ	#VALUE!	1	#VALUE!
0	T1	E02	0	0	kleiner LLOQ	#VALUE!	1	#VALUE!
0	T1	E03	0	0	kleiner LLOQ	#VALUE!	1	#VALUE!
0	T1	E04	0	0	kleiner LLOQ	#VALUE!	1	#VALUE!
T1SS11	T1	E05	0.964	0.913	0.913	0.040	13.75	0.546
T1SS11	T1	E06	1.027	0.976	0.976	0.050	13.75	0.688
0	T1	E07	0	0	kleiner LLOQ	#VALUE!	1	#VALUE!
0	T1	E08	0	0	kleiner LLOQ	#VALUE!	1	#VALUE!
0	T1	E09	0	0	kleiner LLOQ	#VALUE!	1	#VALUE!
0	T1	E10	0	0	kleiner LLOQ	#VALUE!	1	#VALUE!
0	T1	E11	0	0	kleiner LLOQ	#VALUE!	1	#VALUE!
0	T1	E12	0	0	kleiner LLOQ	#VALUE!	1	#VALUE!
0	T1	F01	0	0	kleiner LLOQ	#VALUE!	1	#VALUE!
0	T1	F02	0	0	kleiner LLOQ	#VALUE!	1	#VALUE!
0	T1	F03	0	0	kleiner LLOQ	#VALUE!	1	#VALUE!
0	T1	F04	0	0	kleiner LLOQ	#VALUE!	1	#VALUE!
T1SS21	T1	F05	0.965	0.914	0.914	0.040	24.75	0.986
T1SS21	T1	F06	1.043	0.992	0.992	0.053	24.75	1.306
T1SS22	T1	F07	1.423	1.372	1.372	0.116	24.75	2.859
T1SS22	T1	F08	1.549	1.498	1.498	0.136	24.75	3.375
0	T1	F09	0	0	kleiner LLOQ	#VALUE!	1	#VALUE!
0	T1	F10	0	0	kleiner LLOQ	#VALUE!	1	#VALUE!
0	T1	F11	0	0	kleiner LLOQ	#VALUE!	1	#VALUE!
0	T1	F12	0	0	kleiner LLOQ	#VALUE!	1	#VALUE!
0	T1	G01	0	0	kleiner LLOQ	#VALUE!	1	#VALUE!
0	T1	G02	0	0	kleiner LLOQ	#VALUE!	1	#VALUE!
0	T1	G03	0	0	kleiner LLOQ	#VALUE!	1	#VALUE!
0	T1	G04	0	0	kleiner LLOQ	#VALUE!	1	#VALUE!
0	T1	G05	0	0	kleiner LLOQ	#VALUE!	1	#VALUE!
0	T1	G06	0	0	kleiner LLOQ	#VALUE!	1	#VALUE!
Blank PEG	T1	G07	0	0	kleiner LLOQ	#VALUE!	2.75	#VALUE!
Blank PEG	T1	G08	0	0	kleiner LLOQ	#VALUE!	2.75	#VALUE!
Blank PEG	T1	G09	0	0	kleiner LLOQ	#VALUE!	2.75	#VALUE!
Blank PEG	T1	G10	0	0	kleiner LLOQ	#VALUE!	2.75	#VALUE!
Blank PEG	T1	G11	0	0	kleiner LLOQ	#VALUE!	2.75	#VALUE!
Blank PEG	T1	G12	0	0	kleiner LLOQ	#VALUE!	2.75	#VALUE!
0	T1	H01	0	0	kleiner LLOQ	#VALUE!	1	#VALUE!
0	T1	H02	0	0	kleiner LLOQ	#VALUE!	1	#VALUE!
0	T1	H03	0	0	kleiner LLOQ	#VALUE!	1	#VALUE!
0	T1	H04	0	0	kleiner LLOQ	#VALUE!	1	#VALUE!
0	T1	H05	0	0	kleiner LLOQ	#VALUE!	1	#VALUE!
0	T1	H06	0	0	kleiner LLOQ	#VALUE!	1	#VALUE!
Blank no PEG	T1	H07	0	0	kleiner LLOQ	#VALUE!	2.75	#VALUE!
Blank no PEG	T1	H08	0	0	kleiner LLOQ	#VALUE!	2.75	#VALUE!
Blank no PEG	T1	H09	0	0	kleiner LLOQ	#VALUE!	2.75	#VALUE!
Blank no PEG	T1	H10	0	0	kleiner LLOQ	#VALUE!	2.75	#VALUE!
Blank no PEG	T1	H11	0	0	kleiner LLOQ	#VALUE!	2.75	#VALUE!
Blank no PEG	T1	H12	0	0	kleiner LLOQ	#VALUE!	2.75	#VALUE!
0	T2	A01	0	0	kleiner LLOQ	#VALUE!	1	#VALUE!
0	T2	A02	0	0	kleiner LLOQ	#VALUE!	1	#VALUE!
0	T2	A03	0	0	kleiner LLOQ	#VALUE!	1	#VALUE!
0	T2	A04	0	0	kleiner LLOQ	#VALUE!	1	#VALUE!
0	T2	A05	0	0	kleiner LLOQ	#VALUE!	1	#VALUE!
0	T2	A06	0	0	kleiner LLOQ	#VALUE!	1	#VALUE!
0	T2	A07	0	0	kleiner LLOQ	#VALUE!	1	#VALUE!
0	T2	A08	0	0	kleiner LLOQ	#VALUE!	1	#VALUE!
0	T2	A09	0	0	kleiner LLOQ	#VALUE!	1	#VALUE!
0	T2	A10	0	0	kleiner LLOQ	#VALUE!	1	#VALUE!
0	T2	A11	0	0	kleiner LLOQ	#VALUE!	1	#VALUE!
0	T2	A12	0	0	kleiner LLOQ	#VALUE!	1	#VALUE!
0	T2	B01	0	0	kleiner LLOQ	#VALUE!	1	#VALUE!
0	T2	B02	0	0	kleiner LLOQ	#VALUE!	1	#VALUE!
0	T2	B03	0	0	kleiner LLOQ	#VALUE!	1	#VALUE!
0	T2	B04	0	0	kleiner LLOQ	#VALUE!	1	#VALUE!
0	T2	B05	0	0	kleiner LLOQ	#VALUE!	1	#VALUE!
0	T2	B06	0	0	kleiner LLOQ	#VALUE!	1	#VALUE!
0	T2	B07	0	0	kleiner LLOQ	#VALUE!	1	#VALUE!
0	T2	B08	0	0	kleiner LLOQ	#VALUE!	1	#VALUE!
0	T2	B09	0	0	kleiner LLOQ	#VALUE!	1	#VALUE!
0	T2	B10	0	0	kleiner LLOQ	#VALUE!	1	#VALUE!
0	T2	B11	0	0	kleiner LLOQ	#VALUE!	1	#VALUE!
0	T2	B12	0	0	kleiner LLOQ	#VALUE!	1	#VALUE!

Plate:	T2

	1	2	3	4	5	6	7	8	9	10	11	12
A												
B												
C												
D									1.460	1.257	1.233	1.375
E											1.554	1.513
F												
G							0.056	0.055	0.055	0.055	0.056	0.063
H					2.569	2.528	0.052	0.053	0.053	0.053	0.052	0.052

Plattenbelegung

	1	2	3	4	5	6	7	8	9	10	11	12
A												
B												
C												
D									T2SN3	T2SN3	T2SN4	T2SN4

0	T2	C01	0	0	kleiner LLOQ	#VALUE!	1	#VALUE!
0	T2	C02	0	0	kleiner LLOQ	#VALUE!	1	#VALUE!
0	T2	C03	0	0	kleiner LLOQ	#VALUE!	1	#VALUE!
0	T2	C04	0	0	kleiner LLOQ	#VALUE!	1	#VALUE!
0	T2	C05	0	0	kleiner LLOQ	#VALUE!	1	#VALUE!
0	T2	C06	0	0	kleiner LLOQ	#VALUE!	1	#VALUE!
0	T2	C07	0	0	kleiner LLOQ	#VALUE!	1	#VALUE!
0	T2	C08	0	0	kleiner LLOQ	#VALUE!	1	#VALUE!
0	T2	C09	0	0	kleiner LLOQ	#VALUE!	1	#VALUE!
0	T2	C10	0	0	kleiner LLOQ	#VALUE!	1	#VALUE!
0	T2	C11	0	0	kleiner LLOQ	#VALUE!	1	#VALUE!
0	T2	C12	0	0	kleiner LLOQ	#VALUE!	1	#VALUE!
0	T2	D01	0	0	kleiner LLOQ	#VALUE!	1	#VALUE!
0	T2	D02	0	0	kleiner LLOQ	#VALUE!	1	#VALUE!
0	T2	D03	0	0	kleiner LLOQ	#VALUE!	1	#VALUE!
0	T2	D04	0	0	kleiner LLOQ	#VALUE!	1	#VALUE!
0	T2	D05	0	0	kleiner LLOQ	#VALUE!	1	#VALUE!
0	T2	D06	0	0	kleiner LLOQ	#VALUE!	1	#VALUE!
0	T2	D07	0	0	kleiner LLOQ	#VALUE!	1	#VALUE!
0	T2	D08	0	0	kleiner LLOQ	#VALUE!	1	#VALUE!
T2SN3	T2	D09	1.460	1.409	1.409	0.122	2.75	0.335
T2SN3	T2	D10	1.257	1.206	1.206	0.088	2.75	0.242
T2SN4	T2	D11	1.233	1.182	1.182	0.084	2.75	0.231
T2SN4	T2	D12	1.375	1.324	1.324	0.108	2.75	0.296
0	T2	E01	0	0	kleiner LLOQ	#VALUE!	1	#VALUE!
0	T2	E02	0	0	kleiner LLOQ	#VALUE!	1	#VALUE!
0	T2	E03	0	0	kleiner LLOQ	#VALUE!	1	#VALUE!
0	T2	E04	0	0	kleiner LLOQ	#VALUE!	1	#VALUE!
0	T2	E05	0	0	kleiner LLOQ	#VALUE!	1	#VALUE!
0	T2	E06	0	0	kleiner LLOQ	#VALUE!	1	#VALUE!
0	T2	E07	0	0	kleiner LLOQ	#VALUE!	1	#VALUE!
0	T2	E08	0	0	kleiner LLOQ	#VALUE!	1	#VALUE!
0	T2	E09	0	0	kleiner LLOQ	#VALUE!	1	#VALUE!
0	T2	E10	0	0	kleiner LLOQ	#VALUE!	1	#VALUE!
T2SS14	T2	E11	1.554	1.503	1.503	0.137	2.75	0.377
T2SS14	T2	E12	1.513	1.462	1.462	0.130	2.75	0.358
0	T2	F01	0	0	kleiner LLOQ	#VALUE!	1	#VALUE!
0	T2	F02	0	0	kleiner LLOQ	#VALUE!	1	#VALUE!
0	T2	F03	0	0	kleiner LLOQ	#VALUE!	1	#VALUE!
0	T2	F04	0	0	kleiner LLOQ	#VALUE!	1	#VALUE!
0	T2	F05	0	0	kleiner LLOQ	#VALUE!	1	#VALUE!
0	T2	F06	0	0	kleiner LLOQ	#VALUE!	1	#VALUE!
0	T2	F07	0	0	kleiner LLOQ	#VALUE!	1	#VALUE!
0	T2	F08	0	0	kleiner LLOQ	#VALUE!	1	#VALUE!
0	T2	F09	0	0	kleiner LLOQ	#VALUE!	1	#VALUE!
0	T2	F10	0	0	kleiner LLOQ	#VALUE!	1	#VALUE!
0	T2	F11	0	0	kleiner LLOQ	#VALUE!	1	#VALUE!
0	T2	F12	0	0	kleiner LLOQ	#VALUE!	1	#VALUE!
0	T2	G01	0	0	kleiner LLOQ	#VALUE!	1	#VALUE!
0	T2	G02	0	0	kleiner LLOQ	#VALUE!	1	#VALUE!
0	T2	G03	0	0	kleiner LLOQ	#VALUE!	1	#VALUE!
0	T2	G04	0	0	kleiner LLOQ	#VALUE!	1	#VALUE!
0	T2	G05	0	0	kleiner LLOQ	#VALUE!	1	#VALUE!
0	T2	G06	0	0	kleiner LLOQ	#VALUE!	1	#VALUE!
Blank PEG	T2	G07	0	0	kleiner LLOQ	#VALUE!	2.75	#VALUE!
Blank PEG	T2	G08	0	0	kleiner LLOQ	#VALUE!	2.75	#VALUE!
Blank PEG	T2	G09	0	0	kleiner LLOQ	#VALUE!	2.75	#VALUE!
Blank PEG	T2	G10	0	0	kleiner LLOQ	#VALUE!	2.75	#VALUE!
Blank PEG	T2	G11	0	0	kleiner LLOQ	#VALUE!	2.75	#VALUE!
Blank PEG	T2	G12	0	0	kleiner LLOQ	#VALUE!	2.75	#VALUE!
0	T2	H01	0	0	kleiner LLOQ	#VALUE!	1	#VALUE!
0	T2	H02	0	0	kleiner LLOQ	#VALUE!	1	#VALUE!
0	T2	H03	0	0	kleiner LLOQ	#VALUE!	1	#VALUE!
0	T2	H04	0	0	kleiner LLOQ	#VALUE!	1	#VALUE!
T2SN2	T2	H05	2.569	2.518	größer ULOQ	#VALUE!	1	#VALUE!
T2SN2	T2	H06	2.528	2.477	größer ULOQ	#VALUE!	1	#VALUE!
Blank no PEG	T2	H07	0	0	kleiner LLOQ	#VALUE!	2.75	#VALUE!
Blank no PEG	T2	H08	0	0	kleiner LLOQ	#VALUE!	2.75	#VALUE!
Blank no PEG	T2	H09	0	0	kleiner LLOQ	#VALUE!	2.75	#VALUE!
Blank no PEG	T2	H10	0	0	kleiner LLOQ	#VALUE!	2.75	#VALUE!
Blank no PEG	T2	H11	0	0	kleiner LLOQ	#VALUE!	2.75	#VALUE!
Blank no PEG	T2	H12	0	0	kleiner LLOQ	#VALUE!	2.75	#VALUE!

E											T2SS14	T2SS14
F												
G							Blank PEG	Blank PEG	Blank PEG	Blank PEG	Blank PEG	Blank PEG
H					T2SN2	T2SN2	Blank no PEG	Blank no PEG	Blank no PEG	Blank no PEG	Blank no PEG	Blank no PEG

Comment:

Date Operator

Date Control

0	T3	A01	0	0	kleiner LLOQ	#VALUE!	1	#VALUE!
0	T3	A02	0	0	kleiner LLOQ	#VALUE!	1	#VALUE!
0	T3	A03	0	0	kleiner LLOQ	#VALUE!	1	#VALUE!
0	T3	A04	0	0	kleiner LLOQ	#VALUE!	1	#VALUE!
0	T3	A05	0	0	kleiner LLOQ	#VALUE!	1	#VALUE!
0	T3	A06	0	0	kleiner LLOQ	#VALUE!	1	#VALUE!
0	T3	A07	0	0	kleiner LLOQ	#VALUE!	1	#VALUE!
0	T3	A08	0	0	kleiner LLOQ	#VALUE!	1	#VALUE!
0	T3	A09	0	0	kleiner LLOQ	#VALUE!	1	#VALUE!
0	T3	A10	0	0	kleiner LLOQ	#VALUE!	1	#VALUE!
0	T3	A11	0	0	kleiner LLOQ	#VALUE!	1	#VALUE!
0	T3	A12	0	0	kleiner LLOQ	#VALUE!	1	#VALUE!
0	T3	B01	0	0	kleiner LLOQ	#VALUE!	1	#VALUE!
0	T3	B02	0	0	kleiner LLOQ	#VALUE!	1	#VALUE!
0	T3	B03	0	0	kleiner LLOQ	#VALUE!	1	#VALUE!
0	T3	B04	0	0	kleiner LLOQ	#VALUE!	1	#VALUE!
0	T3	B05	0	0	kleiner LLOQ	#VALUE!	1	#VALUE!
0	T3	B06	0	0	kleiner LLOQ	#VALUE!	1	#VALUE!
0	T3	B07	0	0	kleiner LLOQ	#VALUE!	1	#VALUE!
0	T3	B08	0	0	kleiner LLOQ	#VALUE!	1	#VALUE!
0	T3	B09	0	0	kleiner LLOQ	#VALUE!	1	#VALUE!
0	T3	B10	0	0	kleiner LLOQ	#VALUE!	1	#VALUE!
0	T3	B11	0	0	kleiner LLOQ	#VALUE!	1	#VALUE!
0	T3	B12	0	0	kleiner LLOQ	#VALUE!	1	#VALUE!
0	T3	C01	0	0	kleiner LLOQ	#VALUE!	1	#VALUE!
0	T3	C02	0	0	kleiner LLOQ	#VALUE!	1	#VALUE!
0	T3	C03	0	0	kleiner LLOQ	#VALUE!	1	#VALUE!
0	T3	C04	0	0	kleiner LLOQ	#VALUE!	1	#VALUE!
0	T3	C05	0	0	kleiner LLOQ	#VALUE!	1	#VALUE!
0	T3	C06	0	0	kleiner LLOQ	#VALUE!	1	#VALUE!
0	T3	C07	0	0	kleiner LLOQ	#VALUE!	1	#VALUE!
0	T3	C08	0	0	kleiner LLOQ	#VALUE!	1	#VALUE!
0	T3	C09	0	0	kleiner LLOQ	#VALUE!	1	#VALUE!
0	T3	C10	0	0	kleiner LLOQ	#VALUE!	1	#VALUE!
0	T3	C11	0	0	kleiner LLOQ	#VALUE!	1	#VALUE!
0	T3	C12	0	0	kleiner LLOQ	#VALUE!	1	#VALUE!
0	T3	D01	0	0	kleiner LLOQ	#VALUE!	1	#VALUE!
0	T3	D02	0	0	kleiner LLOQ	#VALUE!	1	#VALUE!
0	T3	D03	0	0	kleiner LLOQ	#VALUE!	1	#VALUE!
0	T3	D04	0	0	kleiner LLOQ	#VALUE!	1	#VALUE!
0	T3	D05	0	0	kleiner LLOQ	#VALUE!	1	#VALUE!
0	T3	D06	0	0	kleiner LLOQ	#VALUE!	1	#VALUE!
T3SN2	T3	D07	0	0	kleiner LLOQ	#VALUE!	1	#VALUE!
T3SN2	T3	D08	0	0	kleiner LLOQ	#VALUE!	1	#VALUE!
T3SN3	T3	D09	0	0	kleiner LLOQ	#VALUE!	1	#VALUE!
T3SN3	T3	D10	0	0	kleiner LLOQ	#VALUE!	1	#VALUE!
T3SN4	T3	D11	0	0	kleiner LLOQ	#VALUE!	1	#VALUE!
T3SN4	T3	D12	0	0	kleiner LLOQ	#VALUE!	1	#VALUE!
0	T3	E01	0	0	kleiner LLOQ	#VALUE!	1	#VALUE!
0	T3	E02	0	0	kleiner LLOQ	#VALUE!	1	#VALUE!
0	T3	E03	0	0	kleiner LLOQ	#VALUE!	1	#VALUE!
0	T3	E04	0	0	kleiner LLOQ	#VALUE!	1	#VALUE!
T3SS11	T3	E05	0	0	kleiner LLOQ	#VALUE!	1	#VALUE!
T3SS11	T3	E06	0	0	kleiner LLOQ	#VALUE!	1	#VALUE!
T3SS12	T3	E07	0	0	kleiner LLOQ	#VALUE!	1	#VALUE!
T3SS12	T3	E08	0	0	kleiner LLOQ	#VALUE!	1	#VALUE!
T3SS13	T3	E09	0	0	kleiner LLOQ	#VALUE!	1	#VALUE!

Plate: T3

	1	2	3	4	5	6	7	8	9	10	11	12
A												
B												
C												
D												
E												
F									1.557	1.542	0.945	1.229
G							0.052	0.048	0.052	0.052	0.047	0.046
H							0.051	0.060	0.059	0.054	0.055	0.055

Plattenbelegung

	1	2	3	4	5	6	7	8	9	10	11	12
A												
B												
C												
D							T3SN2	T3SN2	T3SN3	T3SN3	T3SN4	T3SN4
E					T3SS11	T3SS11	T3SS12	T3SS12	T3SS13	T3SS13	T3SS14	T3SS14
F					T3SS21	T3SS21	T3SS22	T3SS22	T3SS23	T3SS23	T3SS24	T3SS24
G							Blank PEG	Blank PEG	Blank PEG	Blank PEG	Blank PEG	Blank PEG
H							Blank no PEG	Blank no PEG	Blank no PEG	Blank no PEG	Blank no PEG	Blank no PEG

Comment:

Date Operator

Date Control

T3SS13	T3	E10	0	0	kleiner LLOQ	#VALUE!	1	#VALUE!
T3SS14	T3	E11	0	0	kleiner LLOQ	#VALUE!	1	#VALUE!
T3SS14	T3	E12	0	0	kleiner LLOQ	#VALUE!	1	#VALUE!
0	T3	F01	0	0	kleiner LLOQ	#VALUE!	1	#VALUE!
0	T3	F02	0	0	kleiner LLOQ	#VALUE!	1	#VALUE!
0	T3	F03	0	0	kleiner LLOQ	#VALUE!	1	#VALUE!
0	T3	F04	0	0	kleiner LLOQ	#VALUE!	1	#VALUE!
T3SS21	T3	F05	0	0	kleiner LLOQ	#VALUE!	1	#VALUE!
T3SS21	T3	F06	0	0	kleiner LLOQ	#VALUE!	1	#VALUE!
T3SS22	T3	F07	0	0	kleiner LLOQ	#VALUE!	1	#VALUE!
T3SS22	T3	F08	0	0	kleiner LLOQ	#VALUE!	1	#VALUE!
T3SS23	T3	F09	1.557	1.506	1.506	0.138	2.75	0.378
T3SS23	T3	F10	1.542	1.491	1.491	0.135	2.75	0.372
T3SS24	T3	F11	0.945	0.894	0.894	0.037	2.75	0.100
T3SS24	T3	F12	1.229	1.178	1.178	0.083	2.75	0.229
0	T3	G01	0	0	kleiner LLOQ	#VALUE!	1	#VALUE!
0	T3	G02	0	0	kleiner LLOQ	#VALUE!	1	#VALUE!
0	T3	G03	0	0	kleiner LLOQ	#VALUE!	1	#VALUE!
0	T3	G04	0	0	kleiner LLOQ	#VALUE!	1	#VALUE!
0	T3	G05	0	0	kleiner LLOQ	#VALUE!	1	#VALUE!
0	T3	G06	0	0	kleiner LLOQ	#VALUE!	1	#VALUE!
Blank PEG	T3	G07	0	0	kleiner LLOQ	#VALUE!	2.75	#VALUE!
Blank PEG	T3	G08	0	0	kleiner LLOQ	#VALUE!	2.75	#VALUE!
Blank PEG	T3	G09	0	0	kleiner LLOQ	#VALUE!	2.75	#VALUE!
Blank PEG	T3	G10	0	0	kleiner LLOQ	#VALUE!	2.75	#VALUE!
Blank PEG	T3	G11	0	0	kleiner LLOQ	#VALUE!	2.75	#VALUE!
Blank PEG	T3	G12	0	0	kleiner LLOQ	#VALUE!	2.75	#VALUE!
0	T3	H01	0	0	kleiner LLOQ	#VALUE!	1	#VALUE!
0	T3	H02	0	0	kleiner LLOQ	#VALUE!	1	#VALUE!
0	T3	H03	0	0	kleiner LLOQ	#VALUE!	1	#VALUE!
0	T3	H04	0	0	kleiner LLOQ	#VALUE!	1	#VALUE!
0	T3	H05	0	0	kleiner LLOQ	#VALUE!	1	#VALUE!
0	T3	H06	0	0	kleiner LLOQ	#VALUE!	1	#VALUE!
Blank no PEG	T3	H07	0	0	kleiner LLOQ	#VALUE!	2.75	#VALUE!
Blank no PEG	T3	H08	0	0	kleiner LLOQ	#VALUE!	2.75	#VALUE!
Blank no PEG	T3	H09	0	0	kleiner LLOQ	#VALUE!	2.75	#VALUE!
Blank no PEG	T3	H10	0	0	kleiner LLOQ	#VALUE!	2.75	#VALUE!
Blank no PEG	T3	H11	0	0	kleiner LLOQ	#VALUE!	2.75	#VALUE!
Blank no PEG	T3	H12	0	0	kleiner LLOQ	#VALUE!	2.75	#VALUE!

1.447	1.739		1.170						0.667	0.440	0.043	1
1.447	1.332		1.392	0.137	0.146	0.130	0.138	0.109	0.729	0.478	0.042	
1.391	1.281		1.311	0.204	0.184	0.184	0.198	0.201	0.743	0.474	0.043	
0.961	0.835		1.405	0.336	0.347	0.375	0.387	0.300	0.824	0.483	0.044	
0.445	0.441		1.280	1.085	1.086	1.218	1.004	0.873	0.704	0.457	0.041	
0.355	0.315		1.332	1.768	1.925	1.753	1.739	1.606			0.036	
0.223	0.188	1.666	1.499	1.632		0.048	0.046	0.047	0.048	0.048	0.048	
0.118	0.106	1.556	1.622	1.573		0.047	0.054	0.051	0.051	0.052	0.051	

1.652	1.627		1.188	0.748	0.811	0.764	0.753	0.674	0.751	0.529	0.044	2
1.443	1.372		1.364	0.139	0.147	0.131	0.139	0.110	0.811	0.561	0.043	
1.377	1.281		1.284	0.206	0.189	0.184	0.200	0.202	0.821	0.560	0.044	
0.972	0.883		1.387	0.339	0.349	0.386	0.406	0.312	0.903	0.565	0.044	
0.484	0.474		1.298	1.087	1.113	1.203	1.022	0.903	0.790	0.544	0.042	
0.359	0.326		1.308	1.622	1.622	1.630	1.625	1.550			0.037	
0.228	0.199	1.527	1.373	1.462		0.048	0.046	0.048	0.049	0.049	0.049	
0.125	0.109	1.473	1.410	1.371		0.048	0.054	0.052	0.052	0.052	0.051	

1.579	1.475		1.150	1.210	1.303	1.202	1.219	1.125	0.759	0.549	0.043	3
1.357	1.303		1.283	0.140	0.148	0.131	0.139	0.111	0.820	0.575	0.043	
1.323	1.228		1.227	0.206	0.188	0.184	0.198	0.201	0.824	0.579	0.043	
0.959	0.872		1.321	0.339	0.348	0.387	0.406	0.314	0.909	0.580	0.044	
0.483	0.473		1.270	1.069	1.103	1.181	1.017	0.903	0.798	0.560	0.042	
0.360	0.326		1.270	1.497	1.448	1.496	1.494	1.449			0.037	
0.228	0.199	1.374	1.262	1.314		0.048	0.046	0.048	0.049	0.049	0.049	
0.125	0.110	1.346	1.262	1.253		0.048	0.054	0.052	0.052	0.052	0.051	

1.511	1.369			1.538	1.712	1.528	1.502	1.425			0.043	4
1.296	1.261										0.043	
1.272	1.193										0.043	
0.947	0.859										0.044	
0.481	0.471										0.042	
0.361	0.325										0.037	
0.229	0.199					0.048	0.046	0.048	0.049	0.049	0.049	
0.125	0.110					0.047	0.054	0.052	0.052	0.052	0.051	

1.493	1.317	1.678	1.110	1.621	1.807	1.618	1.584	1.508	0.765	0.597	0.043	5
1.259	1.238	1.850	1.197	0.139	0.150	0.132	0.139	0.111	0.821	0.588	0.043	
1.248	1.175	1.816	1.153	0.204	0.188	0.184	0.197	0.199	0.831	0.599	0.044	
0.943	0.859	1.970	1.231	0.338	0.357	0.386	0.406	0.316	0.916	0.595	0.044	
0.480	0.470	2.220	1.220	1.054	1.088	1.154	1.006	0.902	0.805	0.579	0.042	
0.359	0.325	2.149	1.211	1.395	1.324	1.348	1.345	1.317	0.037	0.037	0.037	
0.228	0.199	1.240	1.151	1.183	0.034	0.049	0.046	0.048	0.049	0.049	0.049	
0.125	0.110	1.223	1.132	1.146	0.038	0.048	0.054	0.052	0.052	0.052	0.051	

1.434	1.258		1.090	1.648	1.876	1.649	1.627	1.535				6
1.217	1.201		1.149									
1.205	1.143		1.109									
0.935	0.845		1.182									
0.477	0.469		1.181									
0.360	0.323		1.167									
0.229	0.200					0.049	0.047	0.048	0.049	0.049	0.049	
0.125	0.110					0.048	0.054	0.052	0.052	0.052	0.051	

1.013	0.810		0.668	0.808	0.940	0.720	0.798	0.687	0.904	0.845	0.043	7
0.709	0.827		0.703	0.142	0.156	0.136	0.137	0.116	0.990	0.846	0.042	
0.682	0.692		0.680	0.197	0.188	0.190	0.191	0.184	1.023	0.861	0.043	
0.507	0.519		0.740	0.316	0.264	0.364	0.377	0.312	1.111	0.870	0.043	
0.394	0.406		0.682	0.580	0.561	0.600	0.529	0.563	0.962	0.834	0.042	
0.333	0.322		0.661	0.995	0.930	0.848	0.832	0.797	0.037	0.037	0.037	
0.229	0.201	0.843	0.679	0.732		0.048	0.046	0.047			0.047	
0.124	0.111	0.773	0.682	0.701		0.047	0.053	0.051	0.051	0.052	0.051	

Skin cultivation

1.459	1.152	1.423	0.357	T0 1 hum sui

1.296	2.288	1.578	0.313									
2.179	1.936	1.737	0.247									
1.984	2.795	2.377	2.503									
2.245	1.949	2.433	1.796									
1.826	1.746	2.254	1.121									
						0.061	0.065	0.056	0.055	0.055	0.057	
						0.051	0.05	0.051	0.05	0.05	0.049	

				1.525	2.025		0.864	1.844	1.522	0.429	0.431	T0 2 hum sui
				1.248	2.278	1.692	1.755	0.855	0.894	0.237	0.249	
				1.831	1.521	1.306	1.483	1.366	1.226	0.199	0.209	
				2.68	2.613	2.821	2.765	2.739	2.522	1.226	1.007	
				2.824	2.685	2.57	2.293	2.352	2.402	0.689	0.637	
				2.742	2.589	2.376	1.965	2.348	2.564	1.208	1.157	
						0.056	0.057	0.057	0.055	0.056	0.054	
						0.051	0.051	0.051	0.054	0.05	0.049	

1.146	0.718	0.999	0.334	1.126	1.411		0.702	1.266	1.141	0.475	0.393	T0 3 hum sui
1.079	1.702	1.052	0.332	0.879	1.61	1.471	1.476	0.811	0.727	0.349	0.252	
1.569	1.355	1.089	0.334	1.638	1.359	1.118	1.137	1.055	0.994	0.283	0.211	
1.482	2.065	1.7	2.17	2.322	2.252	2.398	2.423	2.316	2.134	0.951	0.742	
1.431	1.376	1.754	1.439	2.494	2.177	2.199	2.054	2.029	2.004	0.618	0.508	
1.277	1.075	1.556	0.841	2.342	2.042	1.836	1.62	1.988	2.138	0.912	0.852	
						0.056	0.233	0.207	0.213	0.063	0.053	
						0.052	0.05	0.112	0.06	0.05	0.048	

1.198	1.291	1.335	1.239	1.265	1.305							T0 4 hum sui
1.302	1.349	1.214	1.161	1.334	1.291							
1.262	1.442	1.168	1.090	1.247	1.062							
1.150	1.191	1.027										
0.065	0.066	0.062	0.072	0.060	0.070							
0.044	0.045	0.048	0.046	0.047	0.044							

1.180	1.681	1.830	0.163									T1 1 hum sui
1.567	1.334	1.200	0.100									
1.195	0.788	0.933	0.102									
2.900	2.806	0.758	2.806									
1.946	2.631	2.402	2.890									
2.868	2.795	2.317	3.094									
						0.063	0.052	0.051	0.055	0.051	0.054	
						0.050	0.050	0.050	0.051	0.051	0.052	

				1.200	1.072	0.423	0.615	0.670	0.724	0.150	0.153	T1 2 hum sui
				1.198	1.883	1.575	1.474	1.325	1.299	0.147	0.138	
				1.216	1.428	1.153	1.237	1.217	1.351	0.092	0.112	
				1.874	1.939	1.016	1.042	2.511	2.747	0.342	0.313	
				0.964	1.027	2.081	2.098	2.017	1.779	0.439	0.439	
				0.965	1.043	1.423	1.549	0.497	0.475	0.692	0.690	
0.285	0.298					0.065	0.053	0.052	0.056	0.050	0.055	
						0.051	0.051	0.051	0.052	0.052	0.051	

												T1 3 hum sui
		0.998	0.911	0.555	0.519	0.064	0.054	0.052	0.056	0.051	0.055	
0.381	0.357	0.306	0.303			0.051	0.051	0.051	0.052	0.052	0.052	

1.835	2.383	2.469	0.131									T2 1 hum sui
2.283	2.561	2.352	0.149									
1.978	1.929	2.009	0.110									
	2.292	1.277	1.608									
1.300	2.309	1.954	1.414									
2.308	1.373	1.384	1.407									
						0.054	0.053	0.053	0.053	0.053	0.052	
						0.050	0.051	0.051	0.051	0.051	0.051	

				0.933	0.908	0.853	0.834	1.078	1.053	0.140	0.136	T2 2 hum sui
				1.706	1.731	1.246	1.362	1.168	1.384	0.141	0.139	
				0.958	0.995	0.855	0.843	0.911	0.882	0.117	0.113	
						0.727		1.460	1.257	1.233	1.375	
				2.072	2.091	0.574	0.573	0.572	0.589	1.554	1.513	
				0.606	0.608	2.006	1.811	2.076	1.983	1.751	1.706	
						0.056	0.055	0.055	0.055	0.056	0.063	
						0.052	0.053	0.053	0.053	0.052	0.052	

												T2 3 hum sui
0.490	0.485	0.511	0.494	0.744	0.774	0.056	0.055	0.055	0.057	0.056	0.068	
0.503	0.559	0.312	0.313	2.569	2.528	0.053	0.053	0.053	0.053	0.052	0.052	

0.486	0.355	0.412	0.161									T3 1 hum sui
2.733	2.788	2.574	0.143									
2.165	2.221	2.003	0.121									
	2.184	1.908	1.678									
1.487	2.296	1.702	1.452									
1.489	1.603	1.140	1.130			0.052	0.051	0.052	0.052	0.046	0.048	
						0.050	0.059	0.056	0.054	0.055	0.054	

				0.460	0.524	0.298	0.342	0.392	0.394	0.138	0.152	T3 2 hum sui
				1.359	1.418	1.251	1.253	1.184	1.089	0.144	0.148	
				0.766	0.765	0.780	0.796	0.634	0.647	0.110	0.121	
						0.410	0.080	0.262	0.231	0.503	0.547	
				0.557	0.530	0.621	0.561	0.682	0.634	0.414	0.402	
				0.475	0.468	0.368	0.400	1.557	1.542	0.945	1.229	
						0.052	0.048	0.052	0.052	0.047	0.046	
						0.051	0.060	0.059	0.054	0.055	0.055	

**Project number**		F-120	**Apparatus**	**Wallac Victor**	**Operator**	**IsBa**
**GLP Study (Number)**		n.a.	**Protocol (Instrument method**	**LDH test 2016**	**Date of preparation**	**19-04-18**
**hot substance**	**isotope**	n.a.	**File name (results)**	**IsBa_180419/20_LDH_full_va**	**Date of measurement**	**19-04-18**
	**name**	n.a.	**Kind of well plate**	**96 well**	**shaking time [min]**	**30**
	**ACB-ID**	n.a.	**sample volume [µL]**	**100**	**stirring rate (Target) [rpm]**	**150**
	**Batch number**	n.a.	**Cocktail volume [µL]**	**175**	**Kind of measurement**	**UV-vis**
**cold substance**	**name**	LDH	**ACB-ID of cocktail**		**Wave length [nm]**	**450**
	**ACB-ID**	L23982R	**Matrix**	**DMEM (from powder)+PEG**	**Remarks**	**Cocktail 100µl RM, 75µl STOP**
	**Batch number**	SLBK6345V	**Blank description**	**DMEM/PEG, H2O**	**Remarks**	**7 standards split low/high**
**n.a.**		n.a.	**Pipettes (No. / volume)**	**50-200µl**	**Remarks**	**KLP4 common for both**
**n.a.**		n.a.	**Pipettes (No. / volume)**	**n.a.**	**Remarks**	**n.a.**

Messdaten (diese Tabelle in Bericht übernehmen)

Sample name *	concentration (theor.) *	measured data	measured data	measured data	mean measured	SD	RSD	Blank *	measured data after * Blank subtraction	concentration (calc.) *	Deviation *	Residuen
	[µg/mL]	[AU]	[AU]	[AU]	[AU]	[AU]	[%]	[AU]		[µg/mL]	[%]	
KLP1								0.047				
KLP2								0.054				
KLP3								0.051				
KLP4	0.041	0.959	0.972	0.883	0.938	0.04	4.19	0.051	0.887	0.041	-0.57	0.00
KLP5	0.018	0.483	0.484	0.474	0.480	0.00	0.98	0.052	0.429	0.019	2.42	0.00
KLP6	0.012	0.360	0.359	0.326	0.348	0.02	4.54	0.051	0.297	0.013	5.00	0.00
KLP7	0.007	0.228	0.228	0.199	0.218	0.014	6.35		0.168	0.006	-11.29	0.00
KLP8												

Statistical data

Geradensteigung	Slope	m	21.03
Y-Achsenabschnitt	Y-Intercept	b	0.03
Standardabw. Geradensteigung	SD-Slope	s_m	0.648055501
Standardabw. Achsenabschnittes	SD-Y-Intercept	s_b	0.015177452
Anzahl Messpunkte	number of measuring points	n	4
Quadratsumme	sum of squares	Qxx	0.000664048
Bereichsmitte			0.019557193
Freiheitsgrade	degree of freedom	f	2
Student-t-Faktor für (P = 95 %; f = n-2)	Student-t-factor for (P = 95 %; f = n-2)	t	4.303
Vertrauensbereich Steig. (95 %) Obergrenze		$m + VB_m$	23.81763508
Vertrauensbereich Steig. (95 %) Untergrenze		$m - VB_m$	18.24046944
Vertrauensbereich Achsenabschnitt (95 %) Og.		$b + Vb_b$	0.099335829
Vertrauensbereich Achsenabschnitt (95 %) Ug.		$b - Vb_b$	-0.031281327
Korrelationskoeffizient	correlation coefficient	r	0.9991
Bestimmtheitsmaß	determiantion coefficient	r^2	0.9981
Reststandardabweichung		s_0	0.01669983
Summe Restquadrate		sd	0.657385838
Verfahrensstandardabw.		s_{x0}	0.000794131
Rel. Verfahrensstandardabw. %		V_{x0}	4.060559029

Berichten

mean Blank	**0**	
SD Blank	0.00	
RSD Blank	3.79	x=
x*SD (LLOQ)	0.01	5
x*SD (LOD)	0.01	3
**LLOQ (AU)**	**0.061**	
**LOD (AU)**	**0.057**	
ULOQ	0.887	
LLOQ (Lin)	0.168	

Evaluation / Comment

LDH linearity valid with 7 standards, split in lin high and lin low, each with 4 standards and KLP4 common standard for both. R² 0,9981, deviations for both between -13.34% and +13.04%

Date Operator Date Control

Figure 1 **Linearity**

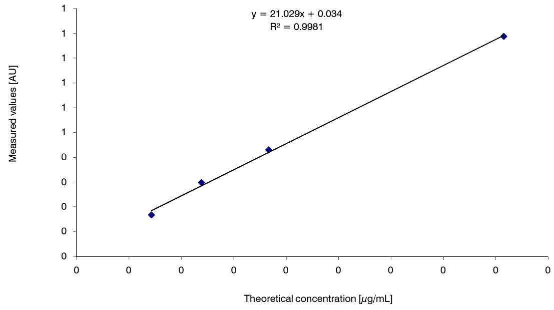

Figure 2 **Method validation Residuen Plot**

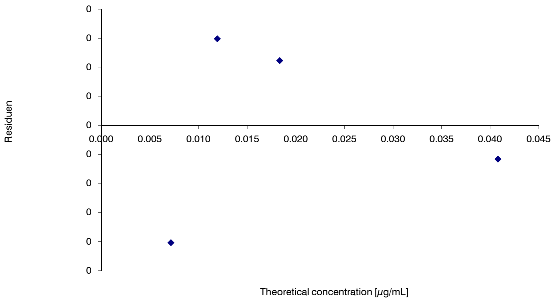

Evaluation / Comment

lin passed

Date	Operator	Date	Control

Sample name	Plate	Position	[AU]	[AU]-Blank	Rating	[µg/mL]	Dilution factor	[µg/mL]
0	T4	A01	0	0	kleiner LLOQ	#VALUE!	1	#VALUE!
0	T4	A02	0	0	kleiner LLOQ	#VALUE!	1	#VALUE!
0	T4	A03	0	0	kleiner LLOQ	#VALUE!	1	#VALUE!
0	T4	A04	0	0	kleiner LLOQ	#VALUE!	1	#VALUE!
0	T4	A05	0	0	kleiner LLOQ	#VALUE!	1	#VALUE!
0	T4	A06	0	0	kleiner LLOQ	#VALUE!	1	#VALUE!
0	T4	A07	0	0	kleiner LLOQ	#VALUE!	1	#VALUE!
0	T4	A08	0	0	kleiner LLOQ	#VALUE!	1	#VALUE!
0	T4	A09	0	0	kleiner LLOQ	#VALUE!	1	#VALUE!
0	T4	A10	0	0	kleiner LLOQ	#VALUE!	1	#VALUE!
0	T4	A11	0	0	kleiner LLOQ	#VALUE!	1	#VALUE!
0	T4	A12	0	0	kleiner LLOQ	#VALUE!	1	#VALUE!
0	T4	B01	0	0	kleiner LLOQ	#VALUE!	1	#VALUE!
0	T4	B02	0	0	kleiner LLOQ	#VALUE!	1	#VALUE!
0	T4	B03	0	0	kleiner LLOQ	#VALUE!	1	#VALUE!
0	T4	B04	0	0	kleiner LLOQ	#VALUE!	1	#VALUE!
0	T4	B05	0	0	kleiner LLOQ	#VALUE!	1	#VALUE!
0	T4	B06	0	0	kleiner LLOQ	#VALUE!	1	#VALUE!
0	T4	B07	0	0	kleiner LLOQ	#VALUE!	1	#VALUE!
0	T4	B08	0	0	kleiner LLOQ	#VALUE!	1	#VALUE!
0	T4	B09	0	0	kleiner LLOQ	#VALUE!	1	#VALUE!
0	T4	B10	0	0	kleiner LLOQ	#VALUE!	1	#VALUE!
0	T4	B11	0	0	kleiner LLOQ	#VALUE!	1	#VALUE!
0	T4	B12	0	0	kleiner LLOQ	#VALUE!	1	#VALUE!
0	T4	C01	0	0	kleiner LLOQ	#VALUE!	1	#VALUE!
0	T4	C02	0	0	kleiner LLOQ	#VALUE!	1	#VALUE!
0	T4	C03	0	0	kleiner LLOQ	#VALUE!	1	#VALUE!
0	T4	C04	0	0	kleiner LLOQ	#VALUE!	1	#VALUE!
0	T4	C05	0	0	kleiner LLOQ	#VALUE!	1	#VALUE!
0	T4	C06	0	0	kleiner LLOQ	#VALUE!	1	#VALUE!
0	T4	C07	0	0	kleiner LLOQ	#VALUE!	1	#VALUE!
0	T4	C08	0	0	kleiner LLOQ	#VALUE!	1	#VALUE!
0	T4	C09	0	0	kleiner LLOQ	#VALUE!	1	#VALUE!
0	T4	C10	0	0	kleiner LLOQ	#VALUE!	1	#VALUE!
0	T4	C11	0	0	kleiner LLOQ	#VALUE!	1	#VALUE!
0	T4	C12	0	0	kleiner LLOQ	#VALUE!	1	#VALUE!
0	T4	D01	0	0	kleiner LLOQ	#VALUE!	1	#VALUE!
0	T4	D02	0	0	kleiner LLOQ	#VALUE!	1	#VALUE!
0	T4	D03	0	0	kleiner LLOQ	#VALUE!	1	#VALUE!
0	T4	D04	0	0	kleiner LLOQ	#VALUE!	1	#VALUE!
0	T4	D05	0	0	kleiner LLOQ	#VALUE!	1	#VALUE!
0	T4	D06	0	0	kleiner LLOQ	#VALUE!	1	#VALUE!
T4SN2	T4	D07	0.427	0.376	0.376	0.016	11	0.179
T4SN2	T4	D08	0.827	0.776	0.776	0.035	11	0.388
T4SN3	T4	D09	0	0	kleiner LLOQ	#VALUE!	1	#VALUE!
T4SN3	T4	D10	0	0	kleiner LLOQ	#VALUE!	1	#VALUE!
T4SN4	T4	D11	0.745	0.694	0.694	0.031	2.75	0.086
T4SN4	T4	D12	0.741	0.690	0.690	0.031	2.75	0.086

Plate:	T4

	Test 1	Test 2	Test 3	Test 4	5	6	7	8	9	10	11	12
A												
B												
C												
D							0.427	0.827			0.745	0.741
E					0.307	0.302	0.444	0.439	0.274	0.233		
F					0.286	0.293	0.642	0.631	0.377	0.392	0.431	0.437
G			0.331	0.350			0.052	0.054	0.052	0.052	0.052	0.051
H							0.050	0.050	0.053	0.050	0.051	0.050

Plattenbelegung

	1	2	3	4	5	6	7	8	9	10	11	12
A												
B												
C												
D							T4SN2	T4SN2	T4SN3	T4SN3	T4SN4	T4SN4
E					T4SS11	T4SS11	T4SS12	T4SS12	T4SS13	T4SS13	T4SS14	T4SS14
F					T4SS21	T4SS21	T4SS22	T4SS22	T4SS23	T4SS23	T4SS24	T4SS24
G			T4SN3	T4SN3			Blank PEG	Blank PEG	Blank PEG	Blank PEG	Blank PEG	Blank PEG
H							Blank no PEG	Blank no PEG	Blank no PEG	Blank no PEG	Blank no PEG	Blank no PEG

Samples aufgeteilt in lin high und low:höher konzentrierte Samples bei lin high zu finden.

dil4*dil2,75=dil11

dil.9*2,75=dil24,75

Comment:

Erste 4 Reihen immer Testmessung zur Evaluierung der benötigten Verdünnung. Wird nicht mit ausgewertet. Unverdünnte Messungen außerhalb der Linearität werden ebenfalls nicht ausgewertet. Dil.Faktor unverdünnte samples=2,75 (100µl sample+100µlRM+75µl stop sol.) Verdünnung (gelb) Dil.8 Dil8*Dil2,75=Dil.22

Date Operator

Date Control

0	T4	E01	0	0	kleiner LLOQ	#VALUE!	1	#VALUE!
0	T4	E02	0	0	kleiner LLOQ	#VALUE!	1	#VALUE!
0	T4	E03	0	0	kleiner LLOQ	#VALUE!	1	#VALUE!
0	T4	E04	0	0	kleiner LLOQ	#VALUE!	1	#VALUE!
T4SS11	T4	E05	0.307	0.256	0.256	0.011	24.75	0.261
T4SS11	T4	E06	0.302	0.251	0.251	0.010	24.75	0.256
T4SS12	T4	E07	0.444	0.393	0.393	0.017	11	0.188
T4SS12	T4	E08	0.439	0.388	0.388	0.017	11	0.185
T4SS13	T4	E09	0.274	0.223	0.223	0.009	24.75	0.222
T4SS13	T4	E10	0.233	0.182	0.182	0.007	24.75	0.174
T4SS14	T4	E11	0	0	kleiner LLOQ	#VALUE!	1	#VALUE!
T4SS14	T4	E12	0	0	kleiner LLOQ	#VALUE!	1	#VALUE!
0	T4	F01	0	0	kleiner LLOQ	#VALUE!	1	#VALUE!
0	T4	F02	0	0	kleiner LLOQ	#VALUE!	1	#VALUE!
0	T4	F03	0	0	kleiner LLOQ	#VALUE!	1	#VALUE!
0	T4	F04	0	0	kleiner LLOQ	#VALUE!	1	#VALUE!
T4SS21	T4	F05	0.286	0.235	0.235	0.010	24.75	0.237
T4SS21	T4	F06	0.293	0.242	0.242	0.010	24.75	0.245
T4SS22	T4	F07	0.642	0.591	0.591	0.027	11	0.292
T4SS22	T4	F08	0.631	0.580	0.580	0.026	11	0.286
T4SS23	T4	F09	0.377	0.326	0.326	0.014	11	0.153
T4SS23	T4	F10	0.392	0.341	0.341	0.015	11	0.160
T4SS24	T4	F11	0.431	0.380	0.380	0.016	11	0.181
T4SS24	T4	F12	0.437	0.386	0.386	0.017	11	0.184
0	T4	G01	0	0	kleiner LLOQ	#VALUE!	1	#VALUE!
0	T4	G02	0	0	kleiner LLOQ	#VALUE!	1	#VALUE!
T4SN3	T4	G03	0.331	0.281	0.281	0.012	11	0.129
T4SN3	T4	G04	0.350	0.299	0.299	0.013	11	0.139
0	T4	G05	0	0	kleiner LLOQ	#VALUE!	1	#VALUE!
0	T4	G06	0	0	kleiner LLOQ	#VALUE!	1	#VALUE!
Blank PEG	T4	G07	0	0	kleiner LLOQ	#VALUE!	2.75	#VALUE!
Blank PEG	T4	G08	0	0	kleiner LLOQ	#VALUE!	2.75	#VALUE!
Blank PEG	T4	G09	0	0	kleiner LLOQ	#VALUE!	2.75	#VALUE!
Blank PEG	T4	G10	0	0	kleiner LLOQ	#VALUE!	2.75	#VALUE!
Blank PEG	T4	G11	0	0	kleiner LLOQ	#VALUE!	2.75	#VALUE!
Blank PEG	T4	G12	0	0	kleiner LLOQ	#VALUE!	2.75	#VALUE!
0	T4	H01	0	0	kleiner LLOQ	#VALUE!	1	#VALUE!
0	T4	H02	0	0	kleiner LLOQ	#VALUE!	1	#VALUE!
0	T4	H03	0	0	kleiner LLOQ	#VALUE!	1	#VALUE!
0	T4	H04	0	0	kleiner LLOQ	#VALUE!	1	#VALUE!
0	T4	H05	0	0	kleiner LLOQ	#VALUE!	1	#VALUE!
0	T4	H06	0	0	kleiner LLOQ	#VALUE!	1	#VALUE!
Blank no PEG	T4	H07	0	0	kleiner LLOQ	#VALUE!	2.75	#VALUE!
Blank no PEG	T4	H08	0	0	kleiner LLOQ	#VALUE!	2.75	#VALUE!
Blank no PEG	T4	H09	0	0	kleiner LLOQ	#VALUE!	2.75	#VALUE!
Blank no PEG	T4	H10	0	0	kleiner LLOQ	#VALUE!	2.75	#VALUE!
Blank no PEG	T4	H11	0	0	kleiner LLOQ	#VALUE!	2.75	#VALUE!
Blank no PEG	T4	H12	0	0	kleiner LLOQ	#VALUE!	2.75	#VALUE!

0	T5	A01	0	0	kleiner LLOQ	#VALUE!	1	#VALUE!
0	T5	A02	0	0	kleiner LLOQ	#VALUE!	1	#VALUE!
0	T5	A03	0	0	kleiner LLOQ	#VALUE!	1	#VALUE!
0	T5	A04	0	0	kleiner LLOQ	#VALUE!	1	#VALUE!
0	T5	A05	0	0	kleiner LLOQ	#VALUE!	1	#VALUE!
0	T5	A06	0	0	kleiner LLOQ	#VALUE!	1	#VALUE!
0	T5	A07	0	0	kleiner LLOQ	#VALUE!	1	#VALUE!
0	T5	A08	0	0	kleiner LLOQ	#VALUE!	1	#VALUE!
0	T5	A09	0	0	kleiner LLOQ	#VALUE!	1	#VALUE!
0	T5	A10	0	0	kleiner LLOQ	#VALUE!	1	#VALUE!
0	T5	A11	0	0	kleiner LLOQ	#VALUE!	1	#VALUE!
0	T5	A12	0	0	kleiner LLOQ	#VALUE!	1	#VALUE!
0	T5	B01	0	0	kleiner LLOQ	#VALUE!	1	#VALUE!
0	T5	B02	0	0	kleiner LLOQ	#VALUE!	1	#VALUE!
0	T5	B03	0	0	kleiner LLOQ	#VALUE!	1	#VALUE!
0	T5	B04	0	0	kleiner LLOQ	#VALUE!	1	#VALUE!
0	T5	B05	0	0	kleiner LLOQ	#VALUE!	1	#VALUE!
0	T5	B06	0	0	kleiner LLOQ	#VALUE!	1	#VALUE!
0	T5	B07	0	0	kleiner LLOQ	#VALUE!	1	#VALUE!
0	T5	B08	0	0	kleiner LLOQ	#VALUE!	1	#VALUE!
0	T5	B09	0	0	kleiner LLOQ	#VALUE!	1	#VALUE!
0	T5	B10	0	0	kleiner LLOQ	#VALUE!	1	#VALUE!
0	T5	B11	0	0	kleiner LLOQ	#VALUE!	1	#VALUE!
0	T5	B12	0	0	kleiner LLOQ	#VALUE!	1	#VALUE!
0	T5	C01	0	0	kleiner LLOQ	#VALUE!	1	#VALUE!
0	T5	C02	0	0	kleiner LLOQ	#VALUE!	1	#VALUE!
0	T5	C03	0	0	kleiner LLOQ	#VALUE!	1	#VALUE!
0	T5	C04	0	0	kleiner LLOQ	#VALUE!	1	#VALUE!
0	T5	C05	0	0	kleiner LLOQ	#VALUE!	1	#VALUE!
0	T5	C06	0	0	kleiner LLOQ	#VALUE!	1	#VALUE!
0	T5	C07	0	0	kleiner LLOQ	#VALUE!	1	#VALUE!
0	T5	C08	0	0	kleiner LLOQ	#VALUE!	1	#VALUE!
0	T5	C09	0	0	kleiner LLOQ	#VALUE!	1	#VALUE!
0	T5	C10	0	0	kleiner LLOQ	#VALUE!	1	#VALUE!
0	T5	C11	0	0	kleiner LLOQ	#VALUE!	1	#VALUE!
0	T5	C12	0	0	kleiner LLOQ	#VALUE!	1	#VALUE!
0	T5	D01	0	0	kleiner LLOQ	#VALUE!	1	#VALUE!
0	T5	D02	0	0	kleiner LLOQ	#VALUE!	1	#VALUE!
0	T5	D03	0	0	kleiner LLOQ	#VALUE!	1	#VALUE!
0	T5	D04	0	0	kleiner LLOQ	#VALUE!	1	#VALUE!
0	T5	D05	0	0	kleiner LLOQ	#VALUE!	1	#VALUE!
0	T5	D06	0	0	kleiner LLOQ	#VALUE!	1	#VALUE!
T5SN2	T5	D07	0.790	0.739	0.739	0.034	8.25	0.276
T5SN2	T5	D08	0.621	0.570	0.570	0.025	8.25	0.210
T5SN3	T5	D09	0	0	kleiner LLOQ	#VALUE!	1	#VALUE!
T5SN3	T5	D10	0	0	kleiner LLOQ	#VALUE!	1	#VALUE!
T5SN4	T5	D11	0	0.449	0.449	0.020	2.75	0.054
T5SN4	T5	D12	1	0.466	0.466	0.021	2.75	0.056

Plate: T5

	1	2	3	4	5	6	7	8	9	10	11	12
A												
B												
C												
D							0.790	0.621			0.500	0.517
E					0.528	0.542	0.908	0.926	0.448	0.444	0.768	0.780
F					0.841	0.840	0.606	0.597	0.712	0.741	0.916	0.915
G							0.053	0.053	0.054	0.053	0.053	0.052
H							0.101	0.056	0.052	0.050	0.054	0.053

Plattenbelegung

	1	2	3	4	5	6	7	8	9	10	11	12
A												
B												
C												
D							T5SN2	T5SN2	T5SN3	T5SN3	T5SN4	T5SN4
E					T5SS11	T5SS11	T5SS12	T5SS12	T5SS13	T5SS13	T5SS14	T5SS14
F					T5SS21	T5SS21	T5SS22	T5SS22	T5SS23	T5SS23	T5SS24	T5SS24
G							Blank PEG	Blank PEG	Blank PEG	Blank PEG	Blank PEG	Blank PEG
H							Blank no PEG	Blank no PEG	Blank no PEG	Blank no PEG	Blank no PEG	Blank no PEG

dil3*dil2,75=dil8,25

Comment:

Date Operator

Date Control

0	T5	E01	0	0	kleiner LLOQ	#VALUE!	1	#VALUE!
0	T5	E02	0	0	kleiner LLOQ	#VALUE!	1	#VALUE!
0	T5	E03	0	0	kleiner LLOQ	#VALUE!	1	#VALUE!
0	T5	E04	0	0	kleiner LLOQ	#VALUE!	1	#VALUE!
T5SS11	T5	E05	0.528	0.477	0.477	0.021	2.75	0.058
T5SS11	T5	E06	0.542	0.491	0.491	0.022	2.75	0.060
T5SS12	T5	E07	0.908	0.857	0.857	0.039	2.75	0.108
T5SS12	T5	E08	0.926	0.875	0.875	0.040	2.75	0.110
T5SS13	T5	E09	0.448	0.397	0.397	0.017	2.75	0.048
T5SS13	T5	E10	0.444	0.393	0.393	0.017	2.75	0.047
T5SS14	T5	E11	0.768	0.717	0.717	0.032	2.75	0.089
T5SS14	T5	E12	0.780	0.729	0.729	0.033	2.75	0.091
0	T5	F01	0	0	kleiner LLOQ	#VALUE!	1	#VALUE!
0	T5	F02	0	0	kleiner LLOQ	#VALUE!	1	#VALUE!
0	T5	F03	0	0	kleiner LLOQ	#VALUE!	1	#VALUE!
0	T5	F04	0	0	kleiner LLOQ	#VALUE!	1	#VALUE!
T5SS21	T5	F05	0.841	0.790	0.790	0.036	2.75	0.099
T5SS21	T5	F06	0.840	0.789	0.789	0.036	2.75	0.099
T5SS22	T5	F07	0.606	0.555	0.555	0.025	2.75	0.068
T5SS22	T5	F08	0.597	0.546	0.546	0.024	2.75	0.067
T5SS23	T5	F09	0.712	0.661	0.661	0.030	2.75	0.082
T5SS23	T5	F10	0.741	0.690	0.690	0.031	2.75	0.086
T5SS24	T5	F11	0.916	0.865	0.865	0.040	2.75	0.109
T5SS24	T5	F12	0.915	0.864	0.864	0.039	2.75	0.109
0	T5	G01	0	0	kleiner LLOQ	#VALUE!	1	#VALUE!
0	T5	G02	0	0	kleiner LLOQ	#VALUE!	1	#VALUE!
0	T5	G03	0	0	kleiner LLOQ	#VALUE!	1	#VALUE!
0	T5	G04	0	0	kleiner LLOQ	#VALUE!	1	#VALUE!
0	T5	G05	0	0	kleiner LLOQ	#VALUE!	1	#VALUE!
0	T5	G06	0	0	kleiner LLOQ	#VALUE!	1	#VALUE!
Blank PEG	T5	G07	0	0	kleiner LLOQ	#VALUE!	2.75	#VALUE!
Blank PEG	T5	G08	0	0	kleiner LLOQ	#VALUE!	2.75	#VALUE!
Blank PEG	T5	G09	0	0	kleiner LLOQ	#VALUE!	2.75	#VALUE!
Blank PEG	T5	G10	0	0	kleiner LLOQ	#VALUE!	2.75	#VALUE!
Blank PEG	T5	G11	0	0	kleiner LLOQ	#VALUE!	2.75	#VALUE!
Blank PEG	T5	G12	0	0	kleiner LLOQ	#VALUE!	2.75	#VALUE!
0	T5	H01	0	0	kleiner LLOQ	#VALUE!	1	#VALUE!
0	T5	H02	0	0	kleiner LLOQ	#VALUE!	1	#VALUE!
0	T5	H03	0	0	kleiner LLOQ	#VALUE!	1	#VALUE!
0	T5	H04	0	0	kleiner LLOQ	#VALUE!	1	#VALUE!
0	T5	H05	0	0	kleiner LLOQ	#VALUE!	1	#VALUE!
0	T5	H06	0	0	kleiner LLOQ	#VALUE!	1	#VALUE!
Blank no PEG	T5	H07	0	0	kleiner LLOQ	#VALUE!	2.75	#VALUE!
Blank no PEG	T5	H08	0	0	kleiner LLOQ	#VALUE!	2.75	#VALUE!
Blank no PEG	T5	H09	0	0	kleiner LLOQ	#VALUE!	2.75	#VALUE!
Blank no PEG	T5	H10	0	0	kleiner LLOQ	#VALUE!	2.75	#VALUE!
Blank no PEG	T5	H11	0	0	kleiner LLOQ	#VALUE!	2.75	#VALUE!
Blank no PEG	T5	H12	0	0	kleiner LLOQ	#VALUE!	2.75	#VALUE!
0	T6	A01	0	0	kleiner LLOQ	#VALUE!	1	#VALUE!
0	T6	A02	0	0	kleiner LLOQ	#VALUE!	1	#VALUE!
0	T6	A03	0	0	kleiner LLOQ	#VALUE!	1	#VALUE!
0	T6	A04	0	0	kleiner LLOQ	#VALUE!	1	#VALUE!
0	T6	A05	0	0	kleiner LLOQ	#VALUE!	1	#VALUE!
0	T6	A06	0	0	kleiner LLOQ	#VALUE!	1	#VALUE!
0	T6	A07	0	0	kleiner LLOQ	#VALUE!	1	#VALUE!
0	T6	A08	0	0	kleiner LLOQ	#VALUE!	1	#VALUE!
0	T6	A09	0	0	kleiner LLOQ	#VALUE!	1	#VALUE!
0	T6	A10	0	0	kleiner LLOQ	#VALUE!	1	#VALUE!
0	T6	A11	0	0	kleiner LLOQ	#VALUE!	1	#VALUE!
0	T6	A12	0	0	kleiner LLOQ	#VALUE!	1	#VALUE!
0	T6	B01	0	0	kleiner LLOQ	#VALUE!	1	#VALUE!
0	T6	B02	0	0	kleiner LLOQ	#VALUE!	1	#VALUE!
0	T6	B03	0	0	kleiner LLOQ	#VALUE!	1	#VALUE!
0	T6	B04	0	0	kleiner LLOQ	#VALUE!	1	#VALUE!
0	T6	B05	0	0	kleiner LLOQ	#VALUE!	1	#VALUE!
0	T6	B06	0	0	kleiner LLOQ	#VALUE!	1	#VALUE!
0	T6	B07	0	0	kleiner LLOQ	#VALUE!	1	#VALUE!
0	T6	B08	0	0	kleiner LLOQ	#VALUE!	1	#VALUE!
0	T6	B09	0	0	kleiner LLOQ	#VALUE!	1	#VALUE!
0	T6	B10	0	0	kleiner LLOQ	#VALUE!	1	#VALUE!
0	T6	B11	0	0	kleiner LLOQ	#VALUE!	1	#VALUE!
0	T6	B12	0	0	kleiner LLOQ	#VALUE!	1	#VALUE!
0	T6	C01	0	0	kleiner LLOQ	#VALUE!	1	#VALUE!

Plate:	T6

	1	2	3	4	5	6	7	8	9	10	11	12
A												
B												
C												
D									0.607	0.625	0.409	0.413
E					0.341	0.356	0.341	0.349	0.231	0.239	0.257	0.254
F					0.251	0.260	0.283	0.286	0.421	0.436	0.559	0.563
G							0.051	0.050	0.050	0.052	0.051	0.051
H							0.050	0.050	0.050	0.050	0.049	0.049

Plattenbelegung

	1	2	3	4	5	6	7	8	9	10	11	12
A												
B												
C												
D					T6SN1	T6SN1	T6SN2	T6SN2	T6SN3	T6SN3	T6SN4	T6SN4
E					T6SS11	T6SS11	T6SS12	T6SS12	T6SS13	T6SS13	T6SS14	T6SS14

0	T6	C02	0	0	kleiner LLOQ	#VALUE!	1	#VALUE!
0	T6	C03	0	0	kleiner LLOQ	#VALUE!	1	#VALUE!
0	T6	C04	0	0	kleiner LLOQ	#VALUE!	1	#VALUE!
0	T6	C05	0	0	kleiner LLOQ	#VALUE!	1	#VALUE!
0	T6	C06	0	0	kleiner LLOQ	#VALUE!	1	#VALUE!
0	T6	C07	0	0	kleiner LLOQ	#VALUE!	1	#VALUE!
0	T6	C08	0	0	kleiner LLOQ	#VALUE!	1	#VALUE!
0	T6	C09	0	0	kleiner LLOQ	#VALUE!	1	#VALUE!
0	T6	C10	0	0	kleiner LLOQ	#VALUE!	1	#VALUE!
0	T6	C11	0	0	kleiner LLOQ	#VALUE!	1	#VALUE!
0	T6	C12	0	0	kleiner LLOQ	#VALUE!	1	#VALUE!
0	T6	D01	0	0	kleiner LLOQ	#VALUE!	1	#VALUE!
0	T6	D02	0	0	kleiner LLOQ	#VALUE!	1	#VALUE!
0	T6	D03	0	0	kleiner LLOQ	#VALUE!	1	#VALUE!
0	T6	D04	0	0	kleiner LLOQ	#VALUE!	1	#VALUE!
T6SN1	T6	D05	0	0	kleiner LLOQ	#VALUE!	1	#VALUE!
T6SN1	T6	D06	0	0	kleiner LLOQ	#VALUE!	1	#VALUE!
T6SN2	T6	D07	0	0	kleiner LLOQ	#VALUE!	1	#VALUE!
T6SN2	T6	D08	0	0	kleiner LLOQ	#VALUE!	1	#VALUE!
T6SN3	T6	D09	0.607	0.556	0.556	0.025	2.75	0.068
T6SN3	T6	D10	0.625	0.574	0.574	0.026	2.75	0.071
T6SN4	T6	D11	0.409	0.358	0.358	0.015	2.75	0.042
T6SN4	T6	D12	0.413	0.362	0.362	0.016	2.75	0.043
0	T6	E01	0	0	kleiner LLOQ	#VALUE!	1	#VALUE!
0	T6	E02	0	0	kleiner LLOQ	#VALUE!	1	#VALUE!
0	T6	E03	0	0	kleiner LLOQ	#VALUE!	1	#VALUE!
0	T6	E04	0	0	kleiner LLOQ	#VALUE!	1	#VALUE!
T6SS11	T6	E05	0.341	0.290	0.290	0.012	2.75	0.033
T6SS11	T6	E06	0.356	0.305	0.305	0.013	2.75	0.035
T6SS12	T6	E07	0.341	0.290	0.290	0.012	2.75	0.034
T6SS12	T6	E08	0.349	0.298	0.298	0.013	2.75	0.035
T6SS13	T6	E09	0.231	0.180	0.180	0.007	2.75	0.019
T6SS13	T6	E10	0.239	0.188	0.188	0.007	2.75	0.020
T6SS14	T6	E11	0.257	0.206	0.206	0.008	2.75	0.023
T6SS14	T6	E12	0.254	0.204	0.204	0.008	2.75	0.022
0	T6	F01	0	0	kleiner LLOQ	#VALUE!	1	#VALUE!
0	T6	F02	0	0	kleiner LLOQ	#VALUE!	1	#VALUE!
0	T6	F03	0	0	kleiner LLOQ	#VALUE!	1	#VALUE!
0	T6	F04	0	0	kleiner LLOQ	#VALUE!	1	#VALUE!
T6SS21	T6	F05	0.251	0.200	0.200	0.008	2.75	0.022
T6SS21	T6	F06	0.260	0.209	0.209	0.008	2.75	0.023
T6SS22	T6	F07	0.283	0.232	0.232	0.009	2.75	0.026
T6SS22	T6	F08	0.286	0.235	0.235	0.010	2.75	0.026
T6SS23	T6	F09	0.421	0.370	0.370	0.016	2.75	0.044
T6SS23	T6	F10	0.436	0.385	0.385	0.017	2.75	0.046
T6SS24	T6	F11	0.559	0.508	0.508	0.023	2.75	0.062
T6SS24	T6	F12	0.563	0.512	0.512	0.023	2.75	0.063
0	T6	G01	0	0	kleiner LLOQ	#VALUE!	1	#VALUE!
0	T6	G02	0	0	kleiner LLOQ	#VALUE!	1	#VALUE!
0	T6	G03	0	0	kleiner LLOQ	#VALUE!	1	#VALUE!
0	T6	G04	0	0	kleiner LLOQ	#VALUE!	1	#VALUE!
0	T6	G05	0	0	kleiner LLOQ	#VALUE!	1	#VALUE!
0	T6	G06	0	0	kleiner LLOQ	#VALUE!	1	#VALUE!
Blank PEG	T6	G07	0	0	kleiner LLOQ	#VALUE!	2.75	#VALUE!
Blank PEG	T6	G08	0	0	kleiner LLOQ	#VALUE!	2.75	#VALUE!
Blank PEG	T6	G09	0	0	kleiner LLOQ	#VALUE!	2.75	#VALUE!
Blank PEG	T6	G10	0	0	kleiner LLOQ	#VALUE!	2.75	#VALUE!
Blank PEG	T6	G11	0	0	kleiner LLOQ	#VALUE!	2.75	#VALUE!
Blank PEG	T6	G12	0	0	kleiner LLOQ	#VALUE!	2.75	#VALUE!
0	T6	H01	0	0	kleiner LLOQ	#VALUE!	1	#VALUE!
0	T6	H02	0	0	kleiner LLOQ	#VALUE!	1	#VALUE!
0	T6	H03	0	0	kleiner LLOQ	#VALUE!	1	#VALUE!
0	T6	H04	0	0	kleiner LLOQ	#VALUE!	1	#VALUE!
0	T6	H05	0	0	kleiner LLOQ	#VALUE!	1	#VALUE!
0	T6	H06	0	0	kleiner LLOQ	#VALUE!	1	#VALUE!
Blank no PEG	T6	H07	0	0	kleiner LLOQ	#VALUE!	2.75	#VALUE!
Blank no PEG	T6	H08	0	0	kleiner LLOQ	#VALUE!	2.75	#VALUE!
Blank no PEG	T6	H09	0	0	kleiner LLOQ	#VALUE!	2.75	#VALUE!
Blank no PEG	T6	H10	0	0	kleiner LLOQ	#VALUE!	2.75	#VALUE!
Blank no PEG	T6	H11	0	0	kleiner LLOQ	#VALUE!	2.75	#VALUE!
Blank no PEG	T6	H12	0	0	kleiner LLOQ	#VALUE!	2.75	#VALUE!
0	T7	A01	0	0	kleiner LLOQ	#VALUE!	1	#VALUE!
0	T7	A02	0	0	kleiner LLOQ	#VALUE!	1	#VALUE!
0	T7	A03	0	0	kleiner LLOQ	#VALUE!	1	#VALUE!
0	T7	A04	0	0	kleiner LLOQ	#VALUE!	1	#VALUE!
0	T7	A05	0	0	kleiner LLOQ	#VALUE!	1	#VALUE!

F					T6SS21	T6SS21	T6SS22	T6SS22	T6SS23	T6SS23	T6SS24	T6SS24
G							Blank PEG	Blank PEG	Blank PEG	Blank PEG	Blank PEG	Blank PEG
H							Blank no PEG	Blank no PEG	Blank no PEG	Blank no PEG	Blank no PEG	Blank no PEG

Comment:

Date Operator

Date Control

0	T7	A06	0	0	kleiner LLOQ	#VALUE!	1	#VALUE!
0	T7	A07	0	0	kleiner LLOQ	#VALUE!	1	#VALUE!
0	T7	A08	0	0	kleiner LLOQ	#VALUE!	1	#VALUE!
0	T7	A09	0	0	kleiner LLOQ	#VALUE!	1	#VALUE!
0	T7	A10	0	0	kleiner LLOQ	#VALUE!	1	#VALUE!
0	T7	A11	0	0	kleiner LLOQ	#VALUE!	1	#VALUE!
0	T7	A12	0	0	kleiner LLOQ	#VALUE!	1	#VALUE!
0	T7	B01	0	0	kleiner LLOQ	#VALUE!	1	#VALUE!
0	T7	B02	0	0	kleiner LLOQ	#VALUE!	1	#VALUE!
0	T7	B03	0	0	kleiner LLOQ	#VALUE!	1	#VALUE!
0	T7	B04	0	0	kleiner LLOQ	#VALUE!	1	#VALUE!
0	T7	B05	0	0	kleiner LLOQ	#VALUE!	1	#VALUE!
0	T7	B06	0	0	kleiner LLOQ	#VALUE!	1	#VALUE!
0	T7	B07	0	0	kleiner LLOQ	#VALUE!	1	#VALUE!
0	T7	B08	0	0	kleiner LLOQ	#VALUE!	1	#VALUE!
0	T7	B09	0	0	kleiner LLOQ	#VALUE!	1	#VALUE!
0	T7	B10	0	0	kleiner LLOQ	#VALUE!	1	#VALUE!
0	T7	B11	0	0	kleiner LLOQ	#VALUE!	1	#VALUE!
0	T7	B12	0	0	kleiner LLOQ	#VALUE!	1	#VALUE!
0	T7	C01	0	0	kleiner LLOQ	#VALUE!	1	#VALUE!
0	T7	C02	0	0	kleiner LLOQ	#VALUE!	1	#VALUE!
0	T7	C03	0	0	kleiner LLOQ	#VALUE!	1	#VALUE!
0	T7	C04	0	0	kleiner LLOQ	#VALUE!	1	#VALUE!
0	T7	C05	0	0	kleiner LLOQ	#VALUE!	1	#VALUE!
0	T7	C06	0	0	kleiner LLOQ	#VALUE!	1	#VALUE!
0	T7	C07	0	0	kleiner LLOQ	#VALUE!	1	#VALUE!
0	T7	C08	0	0	kleiner LLOQ	#VALUE!	1	#VALUE!
0	T7	C09	0	0	kleiner LLOQ	#VALUE!	1	#VALUE!
0	T7	C10	0	0	kleiner LLOQ	#VALUE!	1	#VALUE!
0	T7	C11	0	0	kleiner LLOQ	#VALUE!	1	#VALUE!
0	T7	C12	0	0	kleiner LLOQ	#VALUE!	1	#VALUE!
0	T7	D01	0	0	kleiner LLOQ	#VALUE!	1	#VALUE!
0	T7	D02	0	0	kleiner LLOQ	#VALUE!	1	#VALUE!
0	T7	D03	0	0	kleiner LLOQ	#VALUE!	1	#VALUE!
0	T7	D04	0	0	kleiner LLOQ	#VALUE!	1	#VALUE!
0	T7	D05	0	0	kleiner LLOQ	#VALUE!	1	#VALUE!
0	T7	D06	0	0	kleiner LLOQ	#VALUE!	1	#VALUE!
T7SN2	T7	D07	0.494	0.443	0.443	0.019	2.75	0.054
T7SN2	T7	D08	0.470	0.419	0.419	0.018	2.75	0.050
T7SN3	T7	D09	0.771	0.720	0.720	0.033	2.75	0.090
T7SN3	T7	D10	0.761	0.710	0.710	0.032	2.75	0.088
T7SN4	T7	D11	0.487	0.436	0.436	0.019	2.75	0.053
T7SN4	T7	D12	0.504	0.453	0.453	0.020	2.75	0.055
0	T7	E01	0	0	kleiner LLOQ	#VALUE!	1	#VALUE!
0	T7	E02	0	0	kleiner LLOQ	#VALUE!	1	#VALUE!
0	T7	E03	0	0	kleiner LLOQ	#VALUE!	1	#VALUE!
0	T7	E04	0	0	kleiner LLOQ	#VALUE!	1	#VALUE!
T7SS11	T7	E05	0.355	0.304	0.304	0.013	2.75	0.035
T7SS11	T7	E06	0.359	0.308	0.308	0.013	2.75	0.036
T7SS12	T7	E07	0.315	0.264	0.264	0.011	2.75	0.030
T7SS12	T7	E08	0.311	0.260	0.260	0.011	2.75	0.030
T7SS13	T7	E09	0.190	0.139	kleiner LLOQ	#VALUE!	2.75	#VALUE!
T7SS13	T7	E10	0.179	0.128	kleiner LLOQ	#VALUE!	2.75	#VALUE!
T7SS14	T7	E11	0.241	0.190	0.190	0.007	2.75	0.020
T7SS14	T7	E12	0.243	0.192	0.192	0.008	2.75	0.021
0	T7	F01	0	0	kleiner LLOQ	#VALUE!	1	#VALUE!
0	T7	F02	0	0	kleiner LLOQ	#VALUE!	1	#VALUE!
0	T7	F03	0	0	kleiner LLOQ	#VALUE!	1	#VALUE!
0	T7	F04	0	0	kleiner LLOQ	#VALUE!	1	#VALUE!
T7SS21	T7	F05	0.246	0.195	0.195	0.008	2.75	0.021
T7SS21	T7	F06	0.258	0.207	0.207	0.008	2.75	0.023
T7SS22	T7	F07	0.225	0.174	0.174	0.007	2.75	0.018
T7SS22	T7	F08	0.260	0.210	0.210	0.008	2.75	0.023
T7SS23	T7	F09	0.351	0.300	0.300	0.013	2.75	0.035
T7SS23	T7	F10	0.350	0.299	0.299	0.013	2.75	0.035
T7SS24	T7	F11	0.431	0.380	0.380	0.016	2.75	0.045
T7SS24	T7	F12	0.444	0.393	0.393	0.017	2.75	0.047
0	T7	G01	0	0	kleiner LLOQ	#VALUE!	1	#VALUE!
0	T7	G02	0	0	kleiner LLOQ	#VALUE!	1	#VALUE!
0	T7	G03	0	0	kleiner LLOQ	#VALUE!	1	#VALUE!
0	T7	G04	0	0	kleiner LLOQ	#VALUE!	1	#VALUE!
0	T7	G05	0	0	kleiner LLOQ	#VALUE!	1	#VALUE!
0	T7	G06	0	0	kleiner LLOQ	#VALUE!	1	#VALUE!
Blank PEG	T7	G07	0	0	kleiner LLOQ	#VALUE!	2.75	#VALUE!

Plate: T7

	1	2	3	4	5	6	7	8	9	10	11	12
A												
B												
C												
D							0.494	0.470	0.771	0.761	0.487	0.504
E					0.355	0.359	0.315	0.311	0.190	0.179	0.241	0.243
F					0.246	0.258	0.225	0.260	0.351	0.350	0.431	0.444
G							0.051	0.051	0.051	0.053	0.052	0.051
H							0.050	0.050	0.050	0.050	0.050	0.050

Plattenbelegung

	1	2	3	4	5	6	7	8	9	10	11	12
A												
B												
C												
D							T7SN2	T7SN2	T7SN3	T7SN3	T7SN4	T7SN4
E					T7SS11	T7SS11	T7SS12	T7SS12	T7SS13	T7SS13	T7SS14	T7SS14
F					T7SS21	T7SS21	T7SS22	T7SS22	T7SS23	T7SS23	T7SS24	T7SS24
G							Blank PEG	Blank PEG	Blank PEG	Blank PEG	Blank PEG	Blank PEG
H							Blank no PEG	Blank no PEG	Blank no PEG	Blank no PEG	Blank no PEG	Blank no PEG

Comment:

Date Operator

Date Control

Blank PEG	T7	G08	0	0	kleiner LLOQ	#VALUE!	2.75	#VALUE!
Blank PEG	T7	G09	0	0	kleiner LLOQ	#VALUE!	2.75	#VALUE!
Blank PEG	T7	G10	0	0	kleiner LLOQ	#VALUE!	2.75	#VALUE!
Blank PEG	T7	G11	0	0	kleiner LLOQ	#VALUE!	2.75	#VALUE!
Blank PEG	T7	G12	0	0	kleiner LLOQ	#VALUE!	2.75	#VALUE!
0	T7	H01	0	0	kleiner LLOQ	#VALUE!	1	#VALUE!
0	T7	H02	0	0	kleiner LLOQ	#VALUE!	1	#VALUE!
0	T7	H03	0	0	kleiner LLOQ	#VALUE!	1	#VALUE!
0	T7	H04	0	0	kleiner LLOQ	#VALUE!	1	#VALUE!
0	T7	H05	0	0	kleiner LLOQ	#VALUE!	1	#VALUE!
0	T7	H06	0	0	kleiner LLOQ	#VALUE!	1	#VALUE!
Blank no PEG	T7	H07	0	0	kleiner LLOQ	#VALUE!	2.75	#VALUE!
Blank no PEG	T7	H08	0	0	kleiner LLOQ	#VALUE!	2.75	#VALUE!
Blank no PEG	T7	H09	0	0	kleiner LLOQ	#VALUE!	2.75	#VALUE!
Blank no PEG	T7	H10	0	0	kleiner LLOQ	#VALUE!	2.75	#VALUE!
Blank no PEG	T7	H11	0	0	kleiner LLOQ	#VALUE!	2.75	#VALUE!
Blank no PEG	T7	H12	0	0	kleiner LLOQ	#VALUE!	2.75	#VALUE!

**Project number**		F-120	**Apparatus**	**Wallac Victor**	**Operator**	**IsBa**
**GLP Study (Number)**		n.a.	**Protocol (Instrument meth**	**LDH test 2016**	**Date of preparation**	**19-04-18**
**hot substance**	**isotope**	n.a.	**File name (results)**	**IsBa_180419/20_LDH_full_**	**Date of measurement**	**19-04-18**
	**name**	n.a.	**Kind of well plate**	**96 well**	**shaking time [min]**	**30**
	**ACB-ID**	n.a.	**sample volume [µL]**	**100**	**stirring rate (Target) [rpm]**	**150**
	**Batch number**	n.a.	**Cocktail volume [µL]**	**175**	**Kind of measurement**	**UV-vis**
**cold substance**	**name**	LDH	**ACB-ID of cocktail**		**Wave length [nm]**	**450**
	**ACB-ID**	L23982R	**Matrix**	**DMEM (from powder)+PE(**	**Remarks**	**Cocktail 100µl RM, 75µl STOP**
	**Batch number**	SLBK6345V	**Blank description**	**DMEM/PEG, H2O**	**Remarks**	**7 standards split low/high**
**n.a.**		n.a.	**Pipettes (No. / volume)**	**50-200µl**	**Remarks**	**KLP4 common for both**
**n.a.**		n.a.	**Pipettes (No. / volume)**	**n.a.**	**Remarks**	**n.a.**

Messdaten (diese Tabelle in Bericht übernehmen)

Sample name *	concentration (theor.) *	measured data	measured data	measured data	mean measured	SD	RSD	Blank *	measured data after * Blank subtraction	concentration (calc.) *	Deviation *	Residuen
	[µg/mL]	[AU]	[AU]	[AU]	[AU]	[AU]	[%]	[AU]		[µg/mL]	[%]	
KLP1	0.148	1.579	1.652	1.627	1.62	0.03	1.87	0.047	1.568	0.148	-0.24	0.00
KLP2	0.114	1.357	1.443	1.372	1.39	0.04	2.70	0.054	1.340	0.110	-3.40	0.00
KLP3	0.074	1.272	1.193	1.228	1.23	0.03	2.63	0.051	1.180	0.084	13.04	0.01
KLP4	0.041	0.959	0.972	0.883	0.94	0.04	4.19	0.051	0.887	0.035	-13.32	-0.01
KLP5								0.052				
KLP6								0.051				
KLP7												
KLP8												

Statistical data

Geradensteigung	Slope	m	6.05
Y-Achsenabschnitt	Y-Intercept	b	0.67
Standardabw. Geradensteigung	SD-Slope	s_m	0.620291227
Standardabw. Achsenabschnittes	SD-Y-Intercept	s_b	0.063694294
Anzahl Messpunkte	number of measuring points	n	4
Quadratsumme	sum of squares	Qxx	0.006578604
Bereichsmitte			0.094336926
Freiheitsgrade	degree of freedom	f	2
Student-t-Faktor für (P = 95 %; f = n-2)	Student-t-factor for (P = 95 %; f = n-2)	t	4.303
Vertrauensbereich Steig. (95 %) Obergrenze		$m + VB_m$	8.722319527
Vertrauensbereich Steig. (95 %) Untergrenze		$m - VB_m$	3.384093228
Vertrauensbereich Achsenabschnitt (95 %) Og.		$b + Vb_b$	0.946914615
Vertrauensbereich Achsenabschnitt (95 %) Ug.		$b - Vb_b$	0.398761518
Korrelationskoeffizient	correlation coefficient	r	0.9897
Bestimmtheitsmaß	determiantion coefficient	r^2	0.9794
Reststandardabweichung		s_0	0.050310948
Summe Restquadrate		sd	2.895583345
Verfahrensstandardabw.		s_{x0}	0.008311454
Rel. Verfahrensstandardabw. %		V_{x0}	8.810393374

mean Blank	**0**	
SD Blank	0.00	
RSD Blank	3.79	x=
x*SD (LLOQ)	0	5
x*SD (LOD)	0	3
**LLOQ (AU)**	**0**	
**LOD (AU)**	**0**	
ULOQ	1.568	
LLOQ (Lin)	0.887	

Evaluation / Comment

LDH linearity valid with 7 standards, split in lin high and lin low, each with 4 standards and KLP4 common standard for both. R² 0,9981, deviations for both between -13.34% and +13.04%

Date Operator Date Control

Figure 1 Linearity

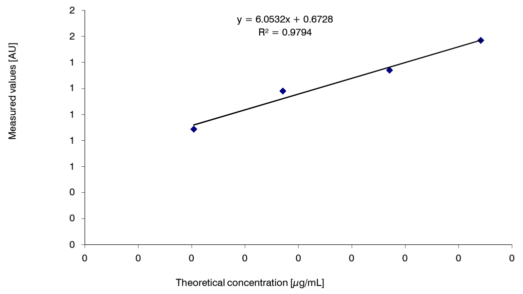

Figure 2 Method validation Residuen Plot

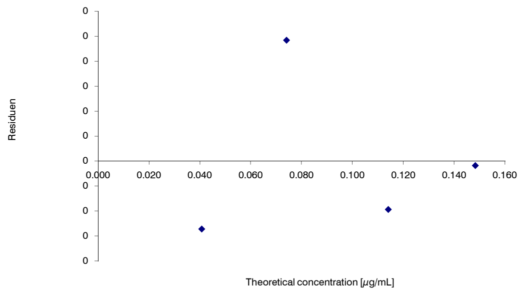

Evaluation / Comment

lin passed

Date	Operator	Date	Control

Sample name	Plate	Position	[AU]	[AU]-Blank	Rating	[µg/mL]	Dilution factor	[µg/mL]
0	T4	A01	0	0	kleiner LLOQ	#VALUE!	1	#VALUE!
0	T4	A02	0	0	kleiner LLOQ	#VALUE!	1	#VALUE!
0	T4	A03	0	0	kleiner LLOQ	#VALUE!	1	#VALUE!
0	T4	A04	0	0	kleiner LLOQ	#VALUE!	1	#VALUE!
0	T4	A05	0.000	-0.051	kleiner LLOQ	#VALUE!		#VALUE!
0	T4	A06	0.000	-0.051	kleiner LLOQ	#VALUE!		#VALUE!
0	T4	A07	0	0	kleiner LLOQ	#VALUE!		#VALUE!
0	T4	A08	0	0	kleiner LLOQ	#VALUE!		#VALUE!
0	T4	A09	0.000	-0.051	kleiner LLOQ	#VALUE!		#VALUE!
0	T4	A10	0.000	-0.051	kleiner LLOQ	#VALUE!		#VALUE!
0	T4	A11	0	0	kleiner LLOQ	#VALUE!		#VALUE!
0	T4	A12	0	0	kleiner LLOQ	#VALUE!		#VALUE!
0	T4	B01	0	0	kleiner LLOQ	#VALUE!		#VALUE!
0	T4	B02	0	0	kleiner LLOQ	#VALUE!		#VALUE!
0	T4	B03	0	0	kleiner LLOQ	#VALUE!		#VALUE!
0	T4	B04	0	0	kleiner LLOQ	#VALUE!		#VALUE!
0	T4	B05	0	0	kleiner LLOQ	#VALUE!		#VALUE!
0	T4	B06	0.000	-0.051	kleiner LLOQ	#VALUE!		#VALUE!
0	T4	B07	0.000	-0.051	kleiner LLOQ	#VALUE!		#VALUE!
0	T4	B08	0.000	-0.051	kleiner LLOQ	#VALUE!		#VALUE!
0	T4	B09	0	0	kleiner LLOQ	#VALUE!		#VALUE!
0	T4	B10	0	0	kleiner LLOQ	#VALUE!		#VALUE!
0	T4	B11	0	0	kleiner LLOQ	#VALUE!		#VALUE!
0	T4	B12	0	0	kleiner LLOQ	#VALUE!		#VALUE!
0	T4	C01	0	0	kleiner LLOQ	#VALUE!		#VALUE!
0	T4	C02	0	0	kleiner LLOQ	#VALUE!		#VALUE!
0	T4	C03	0	0	kleiner LLOQ	#VALUE!		#VALUE!
0	T4	C04	0	0	kleiner LLOQ	#VALUE!		#VALUE!
0	T4	C05	0.000	-0.051	kleiner LLOQ	#VALUE!		#VALUE!
0	T4	C06	0.000	-0.051	kleiner LLOQ	#VALUE!		#VALUE!
0	T4	C07	0.000	-0.051	kleiner LLOQ	#VALUE!		#VALUE!
0	T4	C08	0.000	-0.051	kleiner LLOQ	#VALUE!		#VALUE!
0	T4	C09	0.000	-0.051	kleiner LLOQ	#VALUE!		#VALUE!
0	T4	C10	0.000	-0.051	kleiner LLOQ	#VALUE!		#VALUE!
0	T4	C11	0	0	kleiner LLOQ	#VALUE!	1	#VALUE!
0	T4	C12	0	0	kleiner LLOQ	#VALUE!	1	#VALUE!
0	T4	D01	0	0	kleiner LLOQ	#VALUE!	1	#VALUE!
0	T4	D02	0	0	kleiner LLOQ	#VALUE!	1	#VALUE!
0	T4	D03	0	0	kleiner LLOQ	#VALUE!	1	#VALUE!
0	T4	D04	0	0	kleiner LLOQ	#VALUE!	1	#VALUE!
0	T4	D05	0	0	kleiner LLOQ	#VALUE!	1	#VALUE!
0	T4	D06	0	0	kleiner LLOQ	#VALUE!	1	#VALUE!
T4SN2	T4	D07	0	0	kleiner LLOQ	#VALUE!	1	#VALUE!
T4SN2	T4	D08	0	0	kleiner LLOQ	#VALUE!	1	#VALUE!
T4SN3	T4	D09	0	0	kleiner LLOQ	#VALUE!	1	#VALUE!
T4SN3	T4	D10	0	0	kleiner LLOQ	#VALUE!	1	#VALUE!
T4SN4	T4	D11	0	0	kleiner LLOQ	#VALUE!	1	#VALUE!
T4SN4	T4	D12	0	0	kleiner LLOQ	#VALUE!	1	#VALUE!

Plate: T4

	1	2	3	4	5	6	7	8	9	10	11	12
A												
B												
C												
D												
E											1.258	1.343
F												
G							0.052	0.054	0.052	0.052	0.052	0.051
H							0.050	0.050	0.053	0.050	0.051	0.050

Plattenbelegung

	1	2	3	4	5	6	7	8	9	10	11	12
A												
B												
C												
D							T4SN2	T4SN2	T4SN3	T4SN3	T4SN4	T4SN4
E					T4SS11	T4SS11	T4SS12	T4SS12	T4SS13	T4SS13	T4SS14	T4SS14
F					T4SS21	T4SS21	T4SS22	T4SS22	T4SS23	T4SS23	T4SS24	T4SS24
G							Blank PEG	Blank PEG	Blank PEG	Blank PEG	Blank PEG	Blank PEG
H							Blank no PEG	Blank no PEG	Blank no PEG	Blank no PEG	Blank no PEG	Blank no PEG

Comment:

Erste 4 Reihen immer Testmessung zur Evaluierung der benötigten Verdünnung. Wird nicht mit ausgewertet. Unverdünnte Messungen außerhalb der Linearität werden ebenfalls nicht ausgewertet. Dil.Faktor unverdünnte samples=2,75 (100µl sample+100µlRM+75µl stop sol.) Verdünnung Dil.8 Dil8*Dil2,75=Dil.22, Rot= 2* verdünnt, Dil4 und Dil 8, also Dil 2,75*Dil8*Dil4=Dil88

Samples aufgeteilt in lin high und low:niedriger konzentrierte Samples bei lin low zu finden.

Date Operator

Date Control

0	T4	E01	0	0	kleiner LLOQ	#VALUE!	1	#VALUE!
0	T4	E02	0	0	kleiner LLOQ	#VALUE!	1	#VALUE!
0	T4	E03	0	0	kleiner LLOQ	#VALUE!	1	#VALUE!
0	T4	E04	0	0	kleiner LLOQ	#VALUE!	1	#VALUE!
T4SS11	T4	E05	0	0	kleiner LLOQ	#VALUE!	1	#VALUE!
T4SS11	T4	E06	0	0	kleiner LLOQ	#VALUE!	1	#VALUE!
T4SS12	T4	E07	0	0	kleiner LLOQ	#VALUE!	1	#VALUE!
T4SS12	T4	E08	0	0	kleiner LLOQ	#VALUE!	1	#VALUE!
T4SS13	T4	E09	0	0	kleiner LLOQ	#VALUE!	1	#VALUE!
T4SS13	T4	E10	0	0	kleiner LLOQ	#VALUE!	1	#VALUE!
T4SS14	T4	E11	1.3	1.2	1.2	0.1	2.75	0.243
T4SS14	T4	E12	1.3	1.3	1.3	0.1	2.75	0.281
0	T4	F01	0	0	kleiner LLOQ	#VALUE!	1	#VALUE!
0	T4	F02	0	0	kleiner LLOQ	#VALUE!	1	#VALUE!
0	T4	F03	0	0	kleiner LLOQ	#VALUE!	1	#VALUE!
0	T4	F04	0	0	kleiner LLOQ	#VALUE!	1	#VALUE!
T4SS21	T4	F05	0	0	kleiner LLOQ	#VALUE!	1	#VALUE!
T4SS21	T4	F06	0	0	kleiner LLOQ	#VALUE!	1	#VALUE!
T4SS22	T4	F07	0	0	kleiner LLOQ	#VALUE!	1	#VALUE!
T4SS22	T4	F08	0	0	kleiner LLOQ	#VALUE!	1	#VALUE!
T4SS23	T4	F09	0	0	kleiner LLOQ	#VALUE!	1	#VALUE!
T4SS23	T4	F10	0	0	kleiner LLOQ	#VALUE!	1	#VALUE!
T4SS24	T4	F11	0	0	kleiner LLOQ	#VALUE!	1	#VALUE!
T4SS24	T4	F12	0	0	kleiner LLOQ	#VALUE!	1	#VALUE!
0	T4	G01	0	0	kleiner LLOQ	#VALUE!	1	#VALUE!
0	T4	G02	0	0	kleiner LLOQ	#VALUE!	1	#VALUE!
0	T4	G03	0	0	kleiner LLOQ	#VALUE!	1	#VALUE!
0	T4	G04	0	0	kleiner LLOQ	#VALUE!	1	#VALUE!
0	T4	G05	0	0	kleiner LLOQ	#VALUE!	1	#VALUE!
0	T4	G06	0	0	kleiner LLOQ	#VALUE!	1	#VALUE!
Blank PEG	T4	G07	0	0	kleiner LLOQ	#VALUE!	2.75	#VALUE!
Blank PEG	T4	G08	0	0	kleiner LLOQ	#VALUE!	2.75	#VALUE!
Blank PEG	T4	G09	0	0	kleiner LLOQ	#VALUE!	2.75	#VALUE!
Blank PEG	T4	G10	0	0	kleiner LLOQ	#VALUE!	2.75	#VALUE!
Blank PEG	T4	G11	0	0	kleiner LLOQ	#VALUE!	2.75	#VALUE!
Blank PEG	T4	G12	0	0	kleiner LLOQ	#VALUE!	2.75	#VALUE!
0	T4	H01	0	0	kleiner LLOQ	#VALUE!	1	#VALUE!
0	T4	H02	0	0	kleiner LLOQ	#VALUE!	1	#VALUE!
0	T4	H03	0	0	kleiner LLOQ	#VALUE!	1	#VALUE!
0	T4	H04	0	0	kleiner LLOQ	#VALUE!	1	#VALUE!
0	T4	H05	0	0	kleiner LLOQ	#VALUE!	1	#VALUE!
0	T4	H06	0	0	kleiner LLOQ	#VALUE!	1	#VALUE!
Blank no PEG	T4	H07	0	0	kleiner LLOQ	#VALUE!	2.75	#VALUE!
Blank no PEG	T4	H08	0	0	kleiner LLOQ	#VALUE!	2.75	#VALUE!
Blank no PEG	T4	H09	0	0	kleiner LLOQ	#VALUE!	2.75	#VALUE!
Blank no PEG	T4	H10	0	0	kleiner LLOQ	#VALUE!	2.75	#VALUE!
Blank no PEG	T4	H11	0	0	kleiner LLOQ	#VALUE!	2.75	#VALUE!
Blank no PEG	T4	H12	0	0	kleiner LLOQ	#VALUE!	2.75	#VALUE!

0	T5	A01	0	0	kleiner LLOQ	#VALUE!	1	#VALUE!
0	T5	A02	0	0	kleiner LLOQ	#VALUE!	1	#VALUE!
0	T5	A03	0	0	kleiner LLOQ	#VALUE!	1	#VALUE!
0	T5	A04	0	0	kleiner LLOQ	#VALUE!	1	#VALUE!
0	T5	A05	0	0	kleiner LLOQ	#VALUE!	1	#VALUE!
0	T5	A06	0	0	kleiner LLOQ	#VALUE!	1	#VALUE!
0	T5	A07	0	0	kleiner LLOQ	#VALUE!	1	#VALUE!
0	T5	A08	0	0	kleiner LLOQ	#VALUE!	1	#VALUE!
0	T5	A09	0	0	kleiner LLOQ	#VALUE!	1	#VALUE!
0	T5	A10	0	0	kleiner LLOQ	#VALUE!	1	#VALUE!
0	T5	A11	0	0	kleiner LLOQ	#VALUE!	1	#VALUE!
0	T5	A12	0	0	kleiner LLOQ	#VALUE!	1	#VALUE!
0	T5	B01	0	0	kleiner LLOQ	#VALUE!	1	#VALUE!
0	T5	B02	0	0	kleiner LLOQ	#VALUE!	1	#VALUE!
0	T5	B03	0	0	kleiner LLOQ	#VALUE!	1	#VALUE!
0	T5	B04	0	0	kleiner LLOQ	#VALUE!	1	#VALUE!
0	T5	B05	0	0	kleiner LLOQ	#VALUE!	1	#VALUE!
0	T5	B06	0	0	kleiner LLOQ	#VALUE!	1	#VALUE!
0	T5	B07	0	0	kleiner LLOQ	#VALUE!	1	#VALUE!
0	T5	B08	0	0	kleiner LLOQ	#VALUE!	1	#VALUE!
0	T5	B09	0	0	kleiner LLOQ	#VALUE!	1	#VALUE!
0	T5	B10	0	0	kleiner LLOQ	#VALUE!	1	#VALUE!
0	T5	B11	0	0	kleiner LLOQ	#VALUE!	1	#VALUE!
0	T5	B12	0	0	kleiner LLOQ	#VALUE!	1	#VALUE!
0	T5	C01	0	0	kleiner LLOQ	#VALUE!	1	#VALUE!
0	T5	C02	0	0	kleiner LLOQ	#VALUE!	1	#VALUE!
0	T5	C03	0	0	kleiner LLOQ	#VALUE!	1	#VALUE!
0	T5	C04	0	0	kleiner LLOQ	#VALUE!	1	#VALUE!
0	T5	C05	0	0	kleiner LLOQ	#VALUE!	1	#VALUE!
0	T5	C06	0	0	kleiner LLOQ	#VALUE!	1	#VALUE!
0	T5	C07	0	0	kleiner LLOQ	#VALUE!	1	#VALUE!
0	T5	C08	0	0	kleiner LLOQ	#VALUE!	1	#VALUE!
0	T5	C09	0	0	kleiner LLOQ	#VALUE!	1	#VALUE!
0	T5	C10	0	0	kleiner LLOQ	#VALUE!	1	#VALUE!
0	T5	C11	0	0	kleiner LLOQ	#VALUE!	1	#VALUE!
0	T5	C12	0	0	kleiner LLOQ	#VALUE!	1	#VALUE!
0	T5	D01	0	0	kleiner LLOQ	#VALUE!	1	#VALUE!
0	T5	D02	0	0	kleiner LLOQ	#VALUE!	1	#VALUE!
0	T5	D03	0	0	kleiner LLOQ	#VALUE!	1	#VALUE!
0	T5	D04	0	0	kleiner LLOQ	#VALUE!	1	#VALUE!
0	T5	D05	0	0	kleiner LLOQ	#VALUE!	1	#VALUE!
0	T5	D06	0	0	kleiner LLOQ	#VALUE!	1	#VALUE!
T5SN2	T5	D07	0	0	kleiner LLOQ	#VALUE!	1	#VALUE!
T5SN2	T5	D08	0	0	kleiner LLOQ	#VALUE!	1	#VALUE!
T5SN3	T5	D09	1.131	1.080	1.080	0.067	2.75	0.185
T5SN3	T5	D10	1.140	1.089	1.089	0.069	2.75	0.189
T5SN4	T5	D11	0	0	kleiner LLOQ	#VALUE!	1	#VALUE!
T5SN4	T5	D12	0	0	kleiner LLOQ	#VALUE!	1	#VALUE!

Plate: T5

	1	2	3	4	5	6	7	8	9	10	11	12
A												
B												
C												
D									1.131	1.140		
E												
F												
G							0.053	0.053	0.054	0.053	0.053	0.052
H							0.101	0.056	0.052	0.050	0.054	0.053

Plattenbelegung

	1	2	3	4	5	6	7	8	9	10	11	12
A												
B												
C												
D							T5SN2	T5SN2	T5SN3	T5SN3	T5SN4	T5SN4
E					T5SS11	T5SS11	T5SS12	T5SS12	T5SS13	T5SS13	T5SS14	T5SS14
F					T5SS21	T5SS21	T5SS22	T5SS22	T5SS23	T5SS23	T5SS24	T5SS24
G							Blank PEG	Blank PEG	Blank PEG	Blank PEG	Blank PEG	Blank PEG
H							Blank no PEG	Blank no PEG	Blank no PEG	Blank no PEG	Blank no PEG	Blank no PEG

Comment:

Date Operator

Date Control

0	T5	E01	0	0	kleiner LLOQ	#VALUE!	1	#VALUE!
0	T5	E02	0	0	kleiner LLOQ	#VALUE!	1	#VALUE!
0	T5	E03	0	0	kleiner LLOQ	#VALUE!	1	#VALUE!
0	T5	E04	0	0	kleiner LLOQ	#VALUE!	1	#VALUE!
T5SS11	T5	E05	0	0	kleiner LLOQ	#VALUE!	1	#VALUE!
T5SS11	T5	E06	0	0	kleiner LLOQ	#VALUE!	1	#VALUE!
T5SS12	T5	E07	0	0	kleiner LLOQ	#VALUE!	1	#VALUE!
T5SS12	T5	E08	0	0	kleiner LLOQ	#VALUE!	1	#VALUE!
T5SS13	T5	E09	0	0	kleiner LLOQ	#VALUE!	1	#VALUE!
T5SS13	T5	E10	0	0	kleiner LLOQ	#VALUE!	1	#VALUE!
T5SS14	T5	E11	0	0	kleiner LLOQ	#VALUE!	1	#VALUE!
T5SS14	T5	E12	0	0	kleiner LLOQ	#VALUE!	1	#VALUE!
0	T5	F01	0	0	kleiner LLOQ	#VALUE!	1	#VALUE!
0	T5	F02	0	0	kleiner LLOQ	#VALUE!	1	#VALUE!
0	T5	F03	0	0	kleiner LLOQ	#VALUE!	1	#VALUE!
0	T5	F04	0	0	kleiner LLOQ	#VALUE!	1	#VALUE!
T5SS21	T5	F05	0	0	kleiner LLOQ	#VALUE!	1	#VALUE!
T5SS21	T5	F06	0	0	kleiner LLOQ	#VALUE!	1	#VALUE!
T5SS22	T5	F07	0	0	kleiner LLOQ	#VALUE!	1	#VALUE!
T5SS22	T5	F08	0	0	kleiner LLOQ	#VALUE!	1	#VALUE!
T5SS23	T5	F09	0	0	kleiner LLOQ	#VALUE!	1	#VALUE!
T5SS23	T5	F10	0	0	kleiner LLOQ	#VALUE!	1	#VALUE!
T5SS24	T5	F11	0	0	kleiner LLOQ	#VALUE!	1	#VALUE!
T5SS24	T5	F12	0	0	kleiner LLOQ	#VALUE!	1	#VALUE!
0	T5	G01	0	0	kleiner LLOQ	#VALUE!	1	#VALUE!
0	T5	G02	0	0	kleiner LLOQ	#VALUE!	1	#VALUE!
0	T5	G03	0	0	kleiner LLOQ	#VALUE!	1	#VALUE!
0	T5	G04	0	0	kleiner LLOQ	#VALUE!	1	#VALUE!
0	T5	G05	0	0	kleiner LLOQ	#VALUE!	1	#VALUE!
0	T5	G06	0	0	kleiner LLOQ	#VALUE!	1	#VALUE!
Blank PEG	T5	G07	0	0	kleiner LLOQ	#VALUE!	2.75	#VALUE!
Blank PEG	T5	G08	0	0	kleiner LLOQ	#VALUE!	2.75	#VALUE!
Blank PEG	T5	G09	0	0	kleiner LLOQ	#VALUE!	2.75	#VALUE!
Blank PEG	T5	G10	0	0	kleiner LLOQ	#VALUE!	2.75	#VALUE!
Blank PEG	T5	G11	0	0	kleiner LLOQ	#VALUE!	2.75	#VALUE!
Blank PEG	T5	G12	0	0	kleiner LLOQ	#VALUE!	2.75	#VALUE!
0	T5	H01	0	0	kleiner LLOQ	#VALUE!	1	#VALUE!
0	T5	H02	0	0	kleiner LLOQ	#VALUE!	1	#VALUE!
0	T5	H03	0	0	kleiner LLOQ	#VALUE!	1	#VALUE!
0	T5	H04	0	0	kleiner LLOQ	#VALUE!	1	#VALUE!
0	T5	H05	0	0	kleiner LLOQ	#VALUE!	1	#VALUE!
0	T5	H06	0	0	kleiner LLOQ	#VALUE!	1	#VALUE!
Blank no PEG	T5	H07	0	0	kleiner LLOQ	#VALUE!	2.75	#VALUE!
Blank no PEG	T5	H08	0	0	kleiner LLOQ	#VALUE!	2.75	#VALUE!
Blank no PEG	T5	H09	0	0	kleiner LLOQ	#VALUE!	2.75	#VALUE!
Blank no PEG	T5	H10	0	0	kleiner LLOQ	#VALUE!	2.75	#VALUE!
Blank no PEG	T5	H11	0	0	kleiner LLOQ	#VALUE!	2.75	#VALUE!
Blank no PEG	T5	H12	0	0	kleiner LLOQ	#VALUE!	2.75	#VALUE!
0	T6	A01	0	0	kleiner LLOQ	#VALUE!	1	#VALUE!
0	T6	A02	0	0	kleiner LLOQ	#VALUE!	1	#VALUE!
0	T6	A03	0	0	kleiner LLOQ	#VALUE!	1	#VALUE!
0	T6	A04	0	0	kleiner LLOQ	#VALUE!	1	#VALUE!
0	T6	A05	0	0	kleiner LLOQ	#VALUE!	1	#VALUE!
0	T6	A06	0	0	kleiner LLOQ	#VALUE!	1	#VALUE!
0	T6	A07	0	0	kleiner LLOQ	#VALUE!	1	#VALUE!
0	T6	A08	0	0	kleiner LLOQ	#VALUE!	1	#VALUE!
0	T6	A09	0	0	kleiner LLOQ	#VALUE!	1	#VALUE!
0	T6	A10	0	0	kleiner LLOQ	#VALUE!	1	#VALUE!
0	T6	A11	0	0	kleiner LLOQ	#VALUE!	1	#VALUE!
0	T6	A12	0	0	kleiner LLOQ	#VALUE!	1	#VALUE!
0	T6	B01	0	0	kleiner LLOQ	#VALUE!	1	#VALUE!
0	T6	B02	0	0	kleiner LLOQ	#VALUE!	1	#VALUE!
0	T6	B03	0	0	kleiner LLOQ	#VALUE!	1	#VALUE!
0	T6	B04	0	0	kleiner LLOQ	#VALUE!	1	#VALUE!
0	T6	B05	0	0	kleiner LLOQ	#VALUE!	1	#VALUE!
0	T6	B06	0	0	kleiner LLOQ	#VALUE!	1	#VALUE!
0	T6	B07	0	0	kleiner LLOQ	#VALUE!	1	#VALUE!
0	T6	B08	0	0	kleiner LLOQ	#VALUE!	1	#VALUE!
0	T6	B09	0	0	kleiner LLOQ	#VALUE!	1	#VALUE!
0	T6	B10	0	0	kleiner LLOQ	#VALUE!	1	#VALUE!
0	T6	B11	0	0	kleiner LLOQ	#VALUE!	1	#VALUE!
0	T6	B12	0	0	kleiner LLOQ	#VALUE!	1	#VALUE!
0	T6	C01	0	0	kleiner LLOQ	#VALUE!	1	#VALUE!

Plate: T6

	1	2	3	4	5	6	7	8	9	10	11	12
A												
B												
C												
D							1.027	1.026				
E												
F												
G							0.051	0.050	0.050	0.052	0.051	0.051
H							0.050	0.050	0.050	0.050	0.049	0.049

Plattenbelegung

	1	2	3	4	5	6	7	8	9	10	11	12
A												
B												
C												
D					T6SN1	T6SN1	T6SN2	T6SN2	T6SN3	T6SN3	T6SN4	T6SN4
E					T6SS11	T6SS11	T6SS12	T6SS12	T6SS13	T6SS13	T6SS14	T6SS14

0	T6	C02	0	0	kleiner LLOQ	#VALUE!	1	#VALUE!
0	T6	C03	0	0	kleiner LLOQ	#VALUE!	1	#VALUE!
0	T6	C04	0	0	kleiner LLOQ	#VALUE!	1	#VALUE!
0	T6	C05	0	0	kleiner LLOQ	#VALUE!	1	#VALUE!
0	T6	C06	0	0	kleiner LLOQ	#VALUE!	1	#VALUE!
0	T6	C07	0	0	kleiner LLOQ	#VALUE!	1	#VALUE!
0	T6	C08	0	0	kleiner LLOQ	#VALUE!	1	#VALUE!
0	T6	C09	0	0	kleiner LLOQ	#VALUE!	1	#VALUE!
0	T6	C10	0	0	kleiner LLOQ	#VALUE!	1	#VALUE!
0	T6	C11	0	0	kleiner LLOQ	#VALUE!	1	#VALUE!
0	T6	C12	0	0	kleiner LLOQ	#VALUE!	1	#VALUE!
0	T6	D01	0	0	kleiner LLOQ	#VALUE!	1	#VALUE!
0	T6	D02	0	0	kleiner LLOQ	#VALUE!	1	#VALUE!
0	T6	D03	0	0	kleiner LLOQ	#VALUE!	1	#VALUE!
0	T6	D04	0	0	kleiner LLOQ	#VALUE!	1	#VALUE!
T6SN1	T6	D05	0	0	kleiner LLOQ	#VALUE!	1	#VALUE!
T6SN1	T6	D06	0	0	kleiner LLOQ	#VALUE!	1	#VALUE!
T6SN2	T6	D07	1.027	0.976	0.976	0.050	2.75	0.138
T6SN2	T6	D08	1.026	0.975	0.975	0.050	2.75	0.137
T6SN3	T6	D09	0	0	kleiner LLOQ	#VALUE!	1	#VALUE!
T6SN3	T6	D10	0	0	kleiner LLOQ	#VALUE!	1	#VALUE!
T6SN4	T6	D11	0	0	kleiner LLOQ	#VALUE!	1	#VALUE!
T6SN4	T6	D12	0	0	kleiner LLOQ	#VALUE!	1	#VALUE!
0	T6	E01	0	0	kleiner LLOQ	#VALUE!	1	#VALUE!
0	T6	E02	0	0	kleiner LLOQ	#VALUE!	1	#VALUE!
0	T6	E03	0	0	kleiner LLOQ	#VALUE!	1	#VALUE!
0	T6	E04	0	0	kleiner LLOQ	#VALUE!	1	#VALUE!
T6SS11	T6	E05	0	0	kleiner LLOQ	#VALUE!	1	#VALUE!
T6SS11	T6	E06	0	0	kleiner LLOQ	#VALUE!	1	#VALUE!
T6SS12	T6	E07	0	0	kleiner LLOQ	#VALUE!	1	#VALUE!
T6SS12	T6	E08	0	0	kleiner LLOQ	#VALUE!	1	#VALUE!
T6SS13	T6	E09	0	0	kleiner LLOQ	#VALUE!	1	#VALUE!
T6SS13	T6	E10	0	0	kleiner LLOQ	#VALUE!	1	#VALUE!
T6SS14	T6	E11	0	0	kleiner LLOQ	#VALUE!	1	#VALUE!
T6SS14	T6	E12	0	0	kleiner LLOQ	#VALUE!	1	#VALUE!
0	T6	F01	0	0	kleiner LLOQ	#VALUE!	1	#VALUE!
0	T6	F02	0	0	kleiner LLOQ	#VALUE!	1	#VALUE!
0	T6	F03	0	0	kleiner LLOQ	#VALUE!	1	#VALUE!
0	T6	F04	0	0	kleiner LLOQ	#VALUE!	1	#VALUE!
T6SS21	T6	F05	0	0	kleiner LLOQ	#VALUE!	1	#VALUE!
T6SS21	T6	F06	0	0	kleiner LLOQ	#VALUE!	1	#VALUE!
T6SS22	T6	F07	0	0	kleiner LLOQ	#VALUE!	1	#VALUE!
T6SS22	T6	F08	0	0	kleiner LLOQ	#VALUE!	1	#VALUE!
T6SS23	T6	F09	0	0	kleiner LLOQ	#VALUE!	1	#VALUE!
T6SS23	T6	F10	0	0	kleiner LLOQ	#VALUE!	1	#VALUE!
T6SS24	T6	F11	0	0	kleiner LLOQ	#VALUE!	1	#VALUE!
T6SS24	T6	F12	0	0	kleiner LLOQ	#VALUE!	1	#VALUE!
0	T6	G01	0	0	kleiner LLOQ	#VALUE!	1	#VALUE!
0	T6	G02	0	0	kleiner LLOQ	#VALUE!	1	#VALUE!
0	T6	G03	0	0	kleiner LLOQ	#VALUE!	1	#VALUE!
0	T6	G04	0	0	kleiner LLOQ	#VALUE!	1	#VALUE!
0	T6	G05	0	0	kleiner LLOQ	#VALUE!	1	#VALUE!
0	T6	G06	0	0	kleiner LLOQ	#VALUE!	1	#VALUE!
Blank PEG	T6	G07	0	0	kleiner LLOQ	#VALUE!	2.75	#VALUE!
Blank PEG	T6	G08	0	0	kleiner LLOQ	#VALUE!	2.75	#VALUE!
Blank PEG	T6	G09	0	0	kleiner LLOQ	#VALUE!	2.75	#VALUE!
Blank PEG	T6	G10	0	0	kleiner LLOQ	#VALUE!	2.75	#VALUE!
Blank PEG	T6	G11	0	0	kleiner LLOQ	#VALUE!	2.75	#VALUE!
Blank PEG	T6	G12	0	0	kleiner LLOQ	#VALUE!	2.75	#VALUE!
0	T6	H01	0	0	kleiner LLOQ	#VALUE!	1	#VALUE!
0	T6	H02	0	0	kleiner LLOQ	#VALUE!	1	#VALUE!
0	T6	H03	0	0	kleiner LLOQ	#VALUE!	1	#VALUE!
0	T6	H04	0	0	kleiner LLOQ	#VALUE!	1	#VALUE!
0	T6	H05	0	0	kleiner LLOQ	#VALUE!	1	#VALUE!
0	T6	H06	0	0	kleiner LLOQ	#VALUE!	1	#VALUE!
Blank no PEG	T6	H07	0	0	kleiner LLOQ	#VALUE!	2.75	#VALUE!
Blank no PEG	T6	H08	0	0	kleiner LLOQ	#VALUE!	2.75	#VALUE!
Blank no PEG	T6	H09	0	0	kleiner LLOQ	#VALUE!	2.75	#VALUE!
Blank no PEG	T6	H10	0	0	kleiner LLOQ	#VALUE!	2.75	#VALUE!
Blank no PEG	T6	H11	0	0	kleiner LLOQ	#VALUE!	2.75	#VALUE!
Blank no PEG	T6	H12	0	0	kleiner LLOQ	#VALUE!	2.75	#VALUE!

F					T6SS21	T6SS21	T6SS22	T6SS22	T6SS23	T6SS23	T6SS24	T6SS24
G							Blank PEG	Blank PEG	Blank PEG	Blank PEG	Blank PEG	Blank PEG
H							Blank no PEG	Blank no PEG	Blank no PEG	Blank no PEG	Blank no PEG	Blank no PEG

Comment:

Date Operator

Date Control

0	T7	A01	0	0	kleiner LLOQ	#VALUE!	1	#VALUE!
0	T7	A02	0	0	kleiner LLOQ	#VALUE!	1	#VALUE!
0	T7	A03	0	0	kleiner LLOQ	#VALUE!	1	#VALUE!
0	T7	A04	0	0	kleiner LLOQ	#VALUE!	1	#VALUE!
0	T7	A05	0	0	kleiner LLOQ	#VALUE!	1	#VALUE!
0	T7	A06	0	0	kleiner LLOQ	#VALUE!	1	#VALUE!
0	T7	A07	0	0	kleiner LLOQ	#VALUE!	1	#VALUE!
0	T7	A08	0	0	kleiner LLOQ	#VALUE!	1	#VALUE!
0	T7	A09	0	0	kleiner LLOQ	#VALUE!	1	#VALUE!
0	T7	A10	0	0	kleiner LLOQ	#VALUE!	1	#VALUE!
0	T7	A11	0	0	kleiner LLOQ	#VALUE!	1	#VALUE!
0	T7	A12	0	0	kleiner LLOQ	#VALUE!	1	#VALUE!
0	T7	B01	0	0	kleiner LLOQ	#VALUE!	1	#VALUE!
0	T7	B02	0	0	kleiner LLOQ	#VALUE!	1	#VALUE!
0	T7	B03	0	0	kleiner LLOQ	#VALUE!	1	#VALUE!
0	T7	B04	0	0	kleiner LLOQ	#VALUE!	1	#VALUE!
0	T7	B05	0	0	kleiner LLOQ	#VALUE!	1	#VALUE!
0	T7	B06	0	0	kleiner LLOQ	#VALUE!	1	#VALUE!
0	T7	B07	0	0	kleiner LLOQ	#VALUE!	1	#VALUE!
0	T7	B08	0	0	kleiner LLOQ	#VALUE!	1	#VALUE!
0	T7	B09	0	0	kleiner LLOQ	#VALUE!	1	#VALUE!
0	T7	B10	0	0	kleiner LLOQ	#VALUE!	1	#VALUE!
0	T7	B11	0	0	kleiner LLOQ	#VALUE!	1	#VALUE!
0	T7	B12	0	0	kleiner LLOQ	#VALUE!	1	#VALUE!
0	T7	C01	0	0	kleiner LLOQ	#VALUE!	1	#VALUE!
0	T7	C02	0	0	kleiner LLOQ	#VALUE!	1	#VALUE!
0	T7	C03	0	0	kleiner LLOQ	#VALUE!	1	#VALUE!
0	T7	C04	0	0	kleiner LLOQ	#VALUE!	1	#VALUE!
0	T7	C05	0	0	kleiner LLOQ	#VALUE!	1	#VALUE!
0	T7	C06	0	0	kleiner LLOQ	#VALUE!	1	#VALUE!
0	T7	C07	0	0	kleiner LLOQ	#VALUE!	1	#VALUE!
0	T7	C08	0	0	kleiner LLOQ	#VALUE!	1	#VALUE!
0	T7	C09	0	0	kleiner LLOQ	#VALUE!	1	#VALUE!
0	T7	C10	0	0	kleiner LLOQ	#VALUE!	1	#VALUE!
0	T7	C11	0	0	kleiner LLOQ	#VALUE!	1	#VALUE!
0	T7	C12	0	0	kleiner LLOQ	#VALUE!	1	#VALUE!
0	T7	D01	0	0	kleiner LLOQ	#VALUE!	1	#VALUE!
0	T7	D02	0	0	kleiner LLOQ	#VALUE!	1	#VALUE!
0	T7	D03	0	0	kleiner LLOQ	#VALUE!	1	#VALUE!
0	T7	D04	0	0	kleiner LLOQ	#VALUE!	1	#VALUE!
0	T7	D05	0	0	kleiner LLOQ	#VALUE!	1	#VALUE!
0	T7	D06	0	0	kleiner LLOQ	#VALUE!	1	#VALUE!
T7SN2	T7	D07	0	0	kleiner LLOQ	#VALUE!	1	#VALUE!
T7SN2	T7	D08	0	0	kleiner LLOQ	#VALUE!	1	#VALUE!
T7SN3	T7	D09	0	0	kleiner LLOQ	#VALUE!	1	#VALUE!
T7SN3	T7	D10	0	0	kleiner LLOQ	#VALUE!	1	#VALUE!
T7SN4	T7	D11	0	0	kleiner LLOQ	#VALUE!	1	#VALUE!
T7SN4	T7	D12	0	0	kleiner LLOQ	#VALUE!	1	#VALUE!
0	T7	E01	0	0	kleiner LLOQ	#VALUE!	1	#VALUE!
0	T7	E02	0	0	kleiner LLOQ	#VALUE!	1	#VALUE!
0	T7	E03	0	0	kleiner LLOQ	#VALUE!	1	#VALUE!
0	T7	E04	0	0	kleiner LLOQ	#VALUE!	1	#VALUE!
T7SS11	T7	E05	0	0	kleiner LLOQ	#VALUE!	1	#VALUE!
T7SS11	T7	E06	0	0	kleiner LLOQ	#VALUE!	1	#VALUE!
T7SS12	T7	E07	0	0	kleiner LLOQ	#VALUE!	1	#VALUE!
T7SS12	T7	E08	0	0	kleiner LLOQ	#VALUE!	1	#VALUE!
T7SS13	T7	E09	0	0	kleiner LLOQ	#VALUE!	1	#VALUE!

Plate: T7

	1	2	3	4	5	6	7	8	9	10	11	12
A												
B												
C												
D												
E												
F												
G							0.051	0.051	0.051	0.053	0.052	0.051
H							0.050	0.050	0.050	0.050	0.050	0.050

Plattenbelegung

	1	2	3	4	5	6	7	8	9	10	11	12
A												
B												
C												
D							T7SN2	T7SN2	T7SN3	T7SN3	T7SN4	T7SN4
E					T7SS11	T7SS11	T7SS12	T7SS12	T7SS13	T7SS13	T7SS14	T7SS14
F					T7SS21	T7SS21	T7SS22	T7SS22	T7SS23	T7SS23	T7SS24	T7SS24
G							Blank PEG	Blank PEG	Blank PEG	Blank PEG	Blank PEG	Blank PEG
H							Blank no PEG	Blank no PEG	Blank no PEG	Blank no PEG	Blank no PEG	Blank no PEG

Comment:

alle Messwerte bei lin low

Date Operator

Date Control

T7SS13	T7	E10	0	0	kleiner LLOQ	#VALUE!	1	#VALUE!
T7SS14	T7	E11	0	0	kleiner LLOQ	#VALUE!	1	#VALUE!
T7SS14	T7	E12	0	0	kleiner LLOQ	#VALUE!	1	#VALUE!
0	T7	F01	0	0	kleiner LLOQ	#VALUE!	1	#VALUE!
0	T7	F02	0	0	kleiner LLOQ	#VALUE!	1	#VALUE!
0	T7	F03	0	0	kleiner LLOQ	#VALUE!	1	#VALUE!
0	T7	F04	0	0	kleiner LLOQ	#VALUE!	1	#VALUE!
T7SS21	T7	F05	0	0	kleiner LLOQ	#VALUE!	1	#VALUE!
T7SS21	T7	F06	0	0	kleiner LLOQ	#VALUE!	1	#VALUE!
T7SS22	T7	F07	0	0	kleiner LLOQ	#VALUE!	1	#VALUE!
T7SS22	T7	F08	0	0	kleiner LLOQ	#VALUE!	1	#VALUE!
T7SS23	T7	F09	0	0	kleiner LLOQ	#VALUE!	1	#VALUE!
T7SS23	T7	F10	0	0	kleiner LLOQ	#VALUE!	1	#VALUE!
T7SS24	T7	F11	0	0	kleiner LLOQ	#VALUE!	1	#VALUE!
T7SS24	T7	F12	0	0	kleiner LLOQ	#VALUE!	1	#VALUE!
0	T7	G01	0	0	kleiner LLOQ	#VALUE!	1	#VALUE!
0	T7	G02	0	0	kleiner LLOQ	#VALUE!	1	#VALUE!
0	T7	G03	0	0	kleiner LLOQ	#VALUE!	1	#VALUE!
0	T7	G04	0	0	kleiner LLOQ	#VALUE!	1	#VALUE!
0	T7	G05	0	0	kleiner LLOQ	#VALUE!	1	#VALUE!
0	T7	G06	0	0	kleiner LLOQ	#VALUE!	1	#VALUE!
Blank PEG	T7	G07	0	0	kleiner LLOQ	#VALUE!	1	#VALUE!
Blank PEG	T7	G08	0	0	kleiner LLOQ	#VALUE!	1	#VALUE!
Blank PEG	T7	G09	0	0	kleiner LLOQ	#VALUE!	1	#VALUE!
Blank PEG	T7	G10	0	0	kleiner LLOQ	#VALUE!	1	#VALUE!
Blank PEG	T7	G11	0	0	kleiner LLOQ	#VALUE!	1	#VALUE!
Blank PEG	T7	G12	0	0	kleiner LLOQ	#VALUE!	1	#VALUE!
0	T7	H01	0	0	kleiner LLOQ	#VALUE!	1	#VALUE!
0	T7	H02	0	0	kleiner LLOQ	#VALUE!	1	#VALUE!
0	T7	H03	0	0	kleiner LLOQ	#VALUE!	1	#VALUE!
0	T7	H04	0	0	kleiner LLOQ	#VALUE!	1	#VALUE!
0	T7	H05	0	0	kleiner LLOQ	#VALUE!	1	#VALUE!
0	T7	H06	0	0	kleiner LLOQ	#VALUE!	1	#VALUE!
Blank no PEG	T7	H07	0	0	kleiner LLOQ	#VALUE!	1	#VALUE!
Blank no PEG	T7	H08	0	0	kleiner LLOQ	#VALUE!	1	#VALUE!
Blank no PEG	T7	H09	0	0	kleiner LLOQ	#VALUE!	1	#VALUE!
Blank no PEG	T7	H10	0	0	kleiner LLOQ	#VALUE!	1	#VALUE!
Blank no PEG	T7	H11	0	0	kleiner LLOQ	#VALUE!	1	#VALUE!
Blank no PEG	T7	H12	0	0	kleiner LLOQ	#VALUE!	1	#VALUE!

1.447	1.739		1.170						0.667	0.440	0.043	1
1.447	1.332		1.392	0.137	0.146	0.130	0.138	0.109	0.729	0.478	0.042	
1.391	1.281		1.311	0.204	0.184	0.184	0.198	0.201	0.743	0.474	0.043	
0.961	0.835		1.405	0.336	0.347	0.375	0.387	0.300	0.824	0.483	0.044	
0.445	0.441		1.280	1.085	1.086	1.218	1.004	0.873	0.704	0.457	0.041	
0.355	0.315		1.332	1.768	1.925	1.753	1.739	1.606			0.036	
0.223	0.188	1.666	1.499	1.632		0.048	0.046	0.047	0.048	0.048	0.048	
0.118	0.106	1.556	1.622	1.573		0.047	0.054	0.051	0.051	0.052	0.051	

1.652	1.627		1.188	0.748	0.811	0.764	0.753	0.674	0.751	0.529	0.044	2
1.443	1.372		1.364	0.139	0.147	0.131	0.139	0.110	0.811	0.561	0.043	
1.377	1.281		1.284	0.206	0.189	0.184	0.200	0.202	0.821	0.560	0.044	
0.972	0.883		1.387	0.339	0.349	0.386	0.406	0.312	0.903	0.565	0.044	
0.484	0.474		1.298	1.087	1.113	1.203	1.022	0.903	0.790	0.544	0.042	
0.359	0.326		1.308	1.622	1.622	1.630	1.625	1.550			0.037	
0.228	0.199	1.527	1.373	1.462		0.048	0.046	0.048	0.049	0.049	0.049	
0.125	0.109	1.473	1.410	1.371		0.048	0.054	0.052	0.052	0.052	0.051	

1.579	1.475		1.150	1.210	1.303	1.202	1.219	1.125	0.759	0.549	0.043	3
1.357	1.303		1.283	0.140	0.148	0.131	0.139	0.111	0.820	0.575	0.043	
1.323	1.228		1.227	0.206	0.188	0.184	0.198	0.201	0.824	0.579	0.043	
0.959	0.872		1.321	0.339	0.348	0.387	0.406	0.314	0.909	0.580	0.044	
0.483	0.473		1.270	1.069	1.103	1.181	1.017	0.903	0.798	0.560	0.042	
0.360	0.326		1.270	1.497	1.448	1.496	1.494	1.449			0.037	
0.228	0.199	1.374	1.262	1.314		0.048	0.046	0.048	0.049	0.049	0.049	
0.125	0.110	1.346	1.262	1.253		0.048	0.054	0.052	0.052	0.052	0.051	

1.511	1.369			1.538	1.712	1.528	1.502	1.425			0.043	4
1.296	1.261										0.043	
1.272	1.193										0.043	
0.947	0.859										0.044	
0.481	0.471										0.042	
0.361	0.325										0.037	
0.229	0.199					0.048	0.046	0.048	0.049	0.049	0.049	
0.125	0.110					0.047	0.054	0.052	0.052	0.052	0.051	

1.493	1.317	1.678	1.110	1.621	1.807	1.618	1.584	1.508	0.765	0.597	0.043	5
1.259	1.238	1.850	1.197	0.139	0.150	0.132	0.139	0.111	0.821	0.588	0.043	
1.248	1.175	1.816	1.153	0.204	0.188	0.184	0.197	0.199	0.831	0.599	0.044	
0.943	0.859	1.970	1.231	0.338	0.357	0.386	0.406	0.316	0.916	0.595	0.044	
0.480	0.470	2.220	1.220	1.054	1.088	1.154	1.006	0.902	0.805	0.579	0.042	
0.359	0.325	2.149	1.211	1.395	1.324	1.348	1.345	1.317	0.037	0.037	0.037	
0.228	0.199	1.240	1.151	1.183	0.034	0.049	0.046	0.048	0.049	0.049	0.049	
0.125	0.110	1.223	1.132	1.146	0.038	0.048	0.054	0.052	0.052	0.052	0.051	

1.434	1.258		1.090	1.648	1.876	1.649	1.627	1.535				6
1.217	1.201		1.149									
1.205	1.143		1.109									
0.935	0.845		1.182									
0.477	0.469		1.181									
0.360	0.323		1.167									
0.229	0.200					0.049	0.047	0.048	0.049	0.049	0.049	
0.125	0.110					0.048	0.054	0.052	0.052	0.052	0.051	

1.013	0.810		0.668	0.808	0.940	0.720	0.798	0.687	0.904	0.845	0.043	7
0.709	0.827		0.703	0.142	0.156	0.136	0.137	0.116	0.990	0.846	0.042	
0.682	0.692		0.680	0.197	0.188	0.190	0.191	0.184	1.023	0.861	0.043	
0.507	0.519		0.740	0.316	0.264	0.364	0.377	0.312	1.111	0.870	0.043	
0.394	0.406		0.682	0.580	0.561	0.600	0.529	0.563	0.962	0.834	0.042	
0.333	0.322		0.661	0.995	0.930	0.848	0.832	0.797	0.037	0.037	0.037	
0.229	0.201	0.843	0.679	0.732		0.048	0.046	0.047			0.047	
0.124	0.111	0.773	0.682	0.701		0.047	0.053	0.051	0.051	0.052	0.051	

skin cultivation

1.107	2.478	2.285	0.495									T4 1 hum sui
2.599	2.221	2.620	0.160									
1.529	1.413	1.337	0.179									
	1.715	1.096	0.716									
2.420	1.332	1.841	1.177									
2.159	1.669	1.286	1.300									
						0.051	0.053	0.051	0.052	0.051	0.050	
						0.050	0.050	0.053	0.050	0.050	0.050	

				1.128	1.099	1.007	0.969	0.915	0.921	0.436	0.431	T4 2 hum sui
				1.143	0.846	0.590	0.597	0.077	0.074	0.169	0.174	
				0.558	0.564	0.603	0.587	0.591	0.594	0.198	0.201	
				0.037	0.037	0.427	0.827	1.653	1.703	0.745	0.741	
				0.307	0.302	0.444	0.439	0.274	0.233	1.258	1.343	
				0.286	0.293	0.642	0.631	0.377	0.392	0.431	0.437	
						0.052	0.054	0.052	0.052	0.052	0.051	
						0.050	0.050	0.053	0.050	0.051	0.050	

												T4 3 hum sui
0.633	0.717	0.331	0.350	0.298	0.250	0.154	0.043	0.054	0.056	0.052	0.053	
						0.036	0.044	0.040	0.040	0.042	0.051	

2.884	2.987	2.990	0.834									T5 1 hum sui
0.339	0.285	0.380	0.181									
1.303	1.242	1.088	0.213									
0.035	1.234	0.923	0.438									
0.488	0.743	0.416	0.596									
0.745	0.507	0.617	0.808									
						0.051	0.051	0.053	0.051	0.052	0.051	
						0.056	0.053	0.051	0.049	0.053	0.052	

				0.749	0.763	1.096	1.121	0.981	0.973	0.735	0.717	T5 2 hum sui
				0.323	0.315	0.263	0.273	0.403	0.405	0.192	0.198	
				0.636	0.661	0.715	0.706	1.430	1.378	0.232	0.230	
						0.790	0.621	1.131	1.140	0.500	0.517	
				0.528	0.542	0.908	0.926	0.448	0.444	0.768	0.780	
				0.841	0.840	0.606	0.597	0.712	0.741	0.916	0.915	
						0.053	0.053	0.054	0.053	0.053	0.052	
						0.101	0.056	0.052	0.050	0.054	0.053	

2.565	2.187	2.054	0.598									T6 1 hum sui
0.238	0.241	0.246	0.191									
0.974	1.028	0.879	0.207									
0.82984877	0.556	0.366										
0.311	0.314	0.220	0.248									
0.234	0.277	0.377	0.512									
						0.050	0.049	0.050	0.051	0.050	0.050	
						0.050	0.049	0.050	0.049	0.049	0.049	

				0.873	0.588	0.744	0.778	0.518	0.513	0.634	0.620	T6 2 hum sui
				0.274	0.273	0.262	0.271	0.261	0.272	0.190	0.194	
				1.181	1.211	1.314	1.355	1.060	1.076	0.232	0.227	
						1.027	1.0255289	0.607	0.625	0.409	0.413	
				0.341	0.356	0.341	0.349	0.231	0.239	0.257	0.254	
				0.251	0.260	0.283	0.286	0.421	0.436	0.559	0.563	
						0.051	0.050	0.050	0.052	0.051	0.051	
						0.050	0.050	0.050	0.050	0.049	0.049	

**Project number**		F-120	**Apparatus**	**Wallac Victor**	**Operator**	**IsBa**
**GLP Study (Number)**		n.a.	**Protocol (Instrument method**	**LDH test 2016**	**Date of preparation**	**19-04-18**
**hot substance**	**isotope**	n.a.	**File name (results)**	**IsBa_180419/20_LDH_full_va**	**Date of measurement**	**19-04-18**
	**name**	n.a.	**Kind of well plate**	**96 well**	**shaking time [min]**	**30**
	**ACB-ID**	n.a.	**sample volume [µL]**	**100**	**stirring rate (Target) [rpm]**	**150**
	**Batch number**	n.a.	**Cocktail volume [µL]**	**175**	**Kind of measurement**	**UV-vis**
**cold substance**	**name**	LDH	**ACB-ID of cocktail**		**Wave length [nm]**	**450**
	**ACB-ID**	L23982R	**Matrix**	**DMEM (from powder)+PEG**	**Remarks**	**Cocktail 100µl RM, 75µl STOP**
	**Batch number**	SLBK6345V	**Blank description**	**DMEM/PEG, H2O**	**Remarks**	**7 standards split low/high**
**n.a.**		n.a.	**Pipettes (No. / volume)**	**50-200µl**	**Remarks**	**KLP4 common for both**
**n.a.**		n.a.	**Pipettes (No. / volume)**	**n.a.**	**Remarks**	**n.a.**

Messdaten (diese Tabelle in Bericht übernehmen)

Sample name *	concentration (theor.) *	measured data	measured data	measured data	mean measured	SD	RSD	Blank *	measured data after * Blank subtraction	concentration (calc.) *	Deviation *	Residuen
	[µg/mL]	[AU]	[AU]	[AU]	[AU]	[AU]	[%]	[AU]		[µg/mL]	[%]	
KLP1								0.047				
KLP2								0.054				
KLP3								0.051				
KLP4	0.041	0.959	0.972	0.883	0.938	0.04	4.19	0.051	0.887	0.041	-0.57	0.00
KLP5	0.018	0.483	0.484	0.474	0.480	0.00	0.98	0.052	0.429	0.019	2.42	0.00
KLP6	0.012	0.360	0.359	0.326	0.348	0.02	4.54	0.051	0.297	0.013	5.00	0.00
KLP7	0.007	0.228	0.228	0.199	0.218	0.014	6.35		0.168	0.006	-11.29	0.00
KLP8												

Statistical data

Geradensteigung	Slope	m	21.03
Y-Achsenabschnitt	Y-Intercept	b	0.03
Standardabw. Geradensteigung	SD-Slope	s_m	0.648055501
Standardabw. Achsenabschnittes	SD-Y-Intercept	s_b	0.015177452
Anzahl Messpunkte	number of measuring points	n	4
Quadratsumme	sum of squares	Qxx	0.000664048
Bereichsmitte			0.019557193
Freiheitsgrade	degree of freedom	f	2
Student-t-Faktor für (P = 95 %; f = n-2)	Student-t-factor for (P = 95 %; f = n-2)	t	4.303
Vertrauensbereich Steig. (95 %) Obergrenze		$m + VB_m$	23.81763508
Vertrauensbereich Steig. (95 %) Untergrenze		$m - VB_m$	18.24046944
Vertrauensbereich Achsenabschnitt (95 %) Og.		$b + Vb_b$	0.099335829
Vertrauensbereich Achsenabschnitt (95 %) Ug.		$b - Vb_b$	-0.031281327
Korrelationskoeffizient	correlation coefficient	r	0.9991
Bestimmtheitsmaß	determiantion coefficient	r^2	0.9981
Reststandardabweichung		s_0	0.01669983
Summe Restquadrate		sd	0.657385838
Verfahrensstandardabw.		s_{x0}	0.000794131
Rel. Verfahrensstandardabw. %		V_{x0}	4.060559029

Berichten

mean Blank	0		
SD Blank	0.00		
RSD Blank	3.79	x=	
x*SD (LLOQ)	0.01		5
x*SD (LOD)	0.01		3
**LLOQ (AU)**	**0.061**		
**LOD (AU)**	**0.057**		
ULOQ	0.887		
LLOQ (Lin)	0.168		

Evaluation / Comment

LDH linearity valid with 7 standards, split in lin high and lin low, each with 4 standards and KLP4 common standard for both. R² 0,9981, deviations for both between -13.34% and +13.04%

Date | Operator | Date | Control

Figure 1 **Linearity**

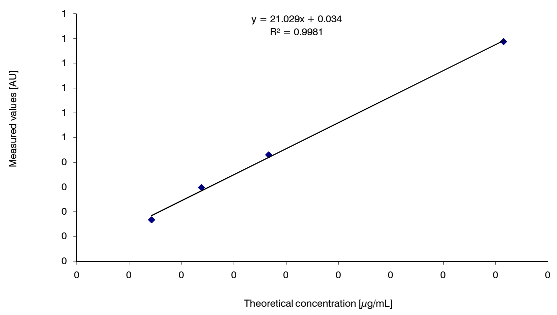

Figure 2 **Method validation Residuen Plot**

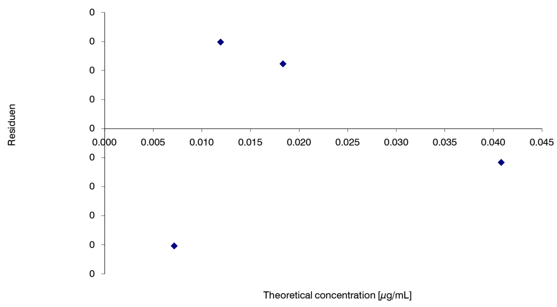

Evaluation / Comment

lin passed

Date	Operator	Date	Control

Sample name	Plate	Position	[AU]	[AU]-Blank	Rating	[µg/mL]	Dilution factor	[µg/mL]
0	T8	A01	0	0	kleiner LLOQ	#VALUE!	1	#VALUE!
0	T8	A02	0	0	kleiner LLOQ	#VALUE!	1	#VALUE!
0	T8	A03	0	0	kleiner LLOQ	#VALUE!	1	#VALUE!
0	T8	A04	0	0	kleiner LLOQ	#VALUE!	1	#VALUE!
0	T8	A05	0	0	kleiner LLOQ	#VALUE!	1	#VALUE!
0	T8	A06	0	0	kleiner LLOQ	#VALUE!	1	#VALUE!
0	T8	A07	0	0	kleiner LLOQ	#VALUE!	1	#VALUE!
0	T8	A08	0	0	kleiner LLOQ	#VALUE!	1	#VALUE!
0	T8	A09	0	0	kleiner LLOQ	#VALUE!	1	#VALUE!
0	T8	A10	0	0	kleiner LLOQ	#VALUE!	1	#VALUE!
0	T8	A11	0	0	kleiner LLOQ	#VALUE!	1	#VALUE!
0	T8	A12	0	0	kleiner LLOQ	#VALUE!	1	#VALUE!
0	T8	B01	0	0	kleiner LLOQ	#VALUE!	1	#VALUE!
0	T8	B02	0	0	kleiner LLOQ	#VALUE!	1	#VALUE!
0	T8	B03	0	0	kleiner LLOQ	#VALUE!	1	#VALUE!
0	T8	B04	0	0	kleiner LLOQ	#VALUE!	1	#VALUE!
0	T8	B05	0	0	kleiner LLOQ	#VALUE!	1	#VALUE!
0	T8	B06	0	0	kleiner LLOQ	#VALUE!	1	#VALUE!
0	T8	B07	0	0	kleiner LLOQ	#VALUE!	1	#VALUE!
0	T8	B08	0	0	kleiner LLOQ	#VALUE!	1	#VALUE!
0	T8	B09	0	0	kleiner LLOQ	#VALUE!	1	#VALUE!
0	T8	B10	0	0	kleiner LLOQ	#VALUE!	1	#VALUE!
0	T8	B11	0	0	kleiner LLOQ	#VALUE!	1	#VALUE!
0	T8	B12	0	0	kleiner LLOQ	#VALUE!	1	#VALUE!
0	T8	C01	0	0	kleiner LLOQ	#VALUE!	1	#VALUE!
0	T8	C02	0	0	kleiner LLOQ	#VALUE!	1	#VALUE!
0	T8	C03	0	0	kleiner LLOQ	#VALUE!	1	#VALUE!
0	T8	C04	0	0	kleiner LLOQ	#VALUE!	1	#VALUE!
0	T8	C05	0	0	kleiner LLOQ	#VALUE!	1	#VALUE!
0	T8	C06	0	0	kleiner LLOQ	#VALUE!	1	#VALUE!
0	T8	C07	0	0	kleiner LLOQ	#VALUE!	1	#VALUE!
0	T8	C08	0	0	kleiner LLOQ	#VALUE!	1	#VALUE!
0	T8	C09	0	0	kleiner LLOQ	#VALUE!	1	#VALUE!
0	T8	C10	0	0	kleiner LLOQ	#VALUE!	1	#VALUE!
0	T8	C11	0	0	kleiner LLOQ	#VALUE!	1	#VALUE!
0	T8	C12	0	0	kleiner LLOQ	#VALUE!	1	#VALUE!
0	T8	D01	0	0	kleiner LLOQ	#VALUE!	1	#VALUE!
0	T8	D02	0	0	kleiner LLOQ	#VALUE!	1	#VALUE!
0	T8	D03	0	0	kleiner LLOQ	#VALUE!	1	#VALUE!
0	T8	D04	0	0	kleiner LLOQ	#VALUE!	1	#VALUE!
T8SN1	T8	D05	0.000	-0.051	kleiner LLOQ	#VALUE!	2.75	#VALUE!
T8SN1	T8	D06	0.000	-0.051	kleiner LLOQ	#VALUE!	2.75	#VALUE!
T8SN2	T8	D07	0.262	0.211	0.211	0.008	2.75	0.023
T8SN2	T8	D08	0.241	0.190	0.190	0.007	2.75	0.020
T8SN3	T8	D09	0.621	0.570	0.570	0.025	2.75	0.070
T8SN3	T8	D10	0.621	0.570	0.570	0.026	2.75	0.070
T8SN4	T8	D11	0.449	0.398	0.398	0.017	2.75	0.048
T8SN4	T8	D12	0.458	0.407	0.407	0.018	2.75	0.049

Plate:	T8

	Test	Test	Test	Test								
	1	2	3	4	5	6	7	8	9	10	11	12
A												
B												
C												
D							0.262	0.241	0.621	0.621	0.449	0.458
E					0.260	0.234	0.197	0.193	0.128	0.132	0.204	0.210
F					0.245	0.247	0.151	0.146	0.271	0.265	0.268	0.253
G							0.053	0.055	0.053	0.054	0.053	0.053
H							0.053	0.052	0.051	0.052	0.052	0.052

Plattenbelegung

	1	2	3	4	5	6	7	8	9	10	11	12
A												
B												
C												
D					T8SN1	T8SN1	T8SN2	T8SN2	T8SN3	T8SN3	T8SN4	T8SN4
E					T8SS11	T8SS11	T8SS12	T8SS12	T8SS13	T8SS13	T8SS14	T8SS14
F					T8SS21	T8SS21	T8SS22	T8SS22	T8SS23	T8SS23	T8SS24	T8SS24
G							Blank PEG	Blank PEG	Blank PEG	Blank PEG	Blank PEG	Blank PEG
H							Blank no PEG	Blank no PEG	Blank no PEG	Blank no PEG	Blank no PEG	Blank no PEG

Samples aufgeteilt in lin high und low:höher konzentrierte Samples bei lin high zu finden.

Comment:

Date Operator

Date Control

0	T8	E01	0	0	kleiner LLOQ	#VALUE!	1	#VALUE!
0	T8	E02	0	0	kleiner LLOQ	#VALUE!	1	#VALUE!
0	T8	E03	0	0	kleiner LLOQ	#VALUE!	1	#VALUE!
0	T8	E04	0	0	kleiner LLOQ	#VALUE!	1	#VALUE!
T8SS11	T8	E05	0.260	0.209	0.209	0.008	2.75	0.023
T8SS11	T8	E06	0.234	0.183	0.183	0.007	2.75	0.019
T8SS12	T8	E07	0.197	0.146	kleiner LLOQ	#VALUE!	2.75	#VALUE!
T8SS12	T8	E08	0.193	0.142	kleiner LLOQ	#VALUE!	2.75	#VALUE!
T8SS13	T8	E09	0.128	0.078	kleiner LLOQ	#VALUE!	2.75	#VALUE!
T8SS13	T8	E10	0.132	0.081	kleiner LLOQ	#VALUE!	2.75	#VALUE!
T8SS14	T8	E11	0.204	0.153	kleiner LLOQ	#VALUE!	2.75	#VALUE!
T8SS14	T8	E12	0.210	0.159	kleiner LLOQ	#VALUE!	2.75	#VALUE!
0	T8	F01	0	0	kleiner LLOQ	#VALUE!	1	#VALUE!
0	T8	F02	0	0	kleiner LLOQ	#VALUE!	1	#VALUE!
0	T8	F03	0	0	kleiner LLOQ	#VALUE!	1	#VALUE!
0	T8	F04	0	0	kleiner LLOQ	#VALUE!	1	#VALUE!
T8SS21	T8	F05	0.245	0.194	0.194	0.008	2.75	0.021
T8SS21	T8	F06	0.247	0.196	0.196	0.008	2.75	0.021
T8SS22	T8	F07	0.151	0.100	kleiner LLOQ	#VALUE!	2.75	#VALUE!
T8SS22	T8	F08	0.146	0.095	kleiner LLOQ	#VALUE!	2.75	#VALUE!
T8SS23	T8	F09	0.271	0.220	0.220	0.009	2.75	0.024
T8SS23	T8	F10	0.265	0.214	0.214	0.009	2.75	0.023
T8SS24	T8	F11	0.268	0.217	0.217	0.009	2.75	0.024
T8SS24	T8	F12	0.253	0.202	0.202	0.008	2.75	0.022
0	T8	G01	0	0	kleiner LLOQ	#VALUE!	1	#VALUE!
0	T8	G02	0	0	kleiner LLOQ	#VALUE!	1	#VALUE!
0	T8	G03	0	0	kleiner LLOQ	#VALUE!	1	#VALUE!
0	T8	G04	0	0	kleiner LLOQ	#VALUE!	1	#VALUE!
0	T8	G05	0	0	kleiner LLOQ	#VALUE!	1	#VALUE!
0	T8	G06	0	0	kleiner LLOQ	#VALUE!	1	#VALUE!
Blank PEG	T8	G07	0	0	kleiner LLOQ	#VALUE!	2.75	#VALUE!
Blank PEG	T8	G08	0	0	kleiner LLOQ	#VALUE!	2.75	#VALUE!
Blank PEG	T8	G09	0	0	kleiner LLOQ	#VALUE!	2.75	#VALUE!
Blank PEG	T8	G10	0	0	kleiner LLOQ	#VALUE!	2.75	#VALUE!
Blank PEG	T8	G11	0	0	kleiner LLOQ	#VALUE!	2.75	#VALUE!
Blank PEG	T8	G12	0	0	kleiner LLOQ	#VALUE!	2.75	#VALUE!
0	T8	H01	0	0	kleiner LLOQ	#VALUE!	1	#VALUE!
0	T8	H02	0	0	kleiner LLOQ	#VALUE!	1	#VALUE!
0	T8	H03	0	0	kleiner LLOQ	#VALUE!	1	#VALUE!
0	T8	H04	0	0	kleiner LLOQ	#VALUE!	1	#VALUE!
0	T8	H05	0	0	kleiner LLOQ	#VALUE!	1	#VALUE!
0	T8	H06	0	0	kleiner LLOQ	#VALUE!	1	#VALUE!
Blank no PEG	T8	H07	0	0	kleiner LLOQ	#VALUE!	2.75	#VALUE!
Blank no PEG	T8	H08	0	0	kleiner LLOQ	#VALUE!	2.75	#VALUE!
Blank no PEG	T8	H09	0	0	kleiner LLOQ	#VALUE!	2.75	#VALUE!
Blank no PEG	T8	H10	0	0	kleiner LLOQ	#VALUE!	2.75	#VALUE!
Blank no PEG	T8	H11	0	0	kleiner LLOQ	#VALUE!	2.75	#VALUE!
Blank no PEG	T8	H12	0	0	kleiner LLOQ	#VALUE!	2.75	#VALUE!

0	T9	A01	0	0	kleiner LLOQ	#VALUE!	1	#VALUE!
0	T9	A02	0	0	kleiner LLOQ	#VALUE!	1	#VALUE!
0	T9	A03	0	0	kleiner LLOQ	#VALUE!	1	#VALUE!
0	T9	A04	0	0	kleiner LLOQ	#VALUE!	1	#VALUE!
0	T9	A05	0	0	kleiner LLOQ	#VALUE!	1	#VALUE!
0	T9	A06	0	0	kleiner LLOQ	#VALUE!	1	#VALUE!
0	T9	A07	0	0	kleiner LLOQ	#VALUE!	1	#VALUE!
0	T9	A08	0	0	kleiner LLOQ	#VALUE!	1	#VALUE!
0	T9	A09	0	0	kleiner LLOQ	#VALUE!	1	#VALUE!
0	T9	A10	0	0	kleiner LLOQ	#VALUE!	1	#VALUE!
0	T9	A11	0	0	kleiner LLOQ	#VALUE!	1	#VALUE!
0	T9	A12	0	0	kleiner LLOQ	#VALUE!	1	#VALUE!
0	T9	B01	0	0	kleiner LLOQ	#VALUE!	1	#VALUE!
0	T9	B02	0	0	kleiner LLOQ	#VALUE!	1	#VALUE!
0	T9	B03	0	0	kleiner LLOQ	#VALUE!	1	#VALUE!
0	T9	B04	0	0	kleiner LLOQ	#VALUE!	1	#VALUE!
0	T9	B05	0	0	kleiner LLOQ	#VALUE!	1	#VALUE!
0	T9	B06	0	0	kleiner LLOQ	#VALUE!	1	#VALUE!
0	T9	B07	0	0	kleiner LLOQ	#VALUE!	1	#VALUE!
0	T9	B08	0	0	kleiner LLOQ	#VALUE!	1	#VALUE!
0	T9	B09	0	0	kleiner LLOQ	#VALUE!	1	#VALUE!
0	T9	B10	0	0	kleiner LLOQ	#VALUE!	1	#VALUE!
0	T9	B11	0	0	kleiner LLOQ	#VALUE!	1	#VALUE!
0	T9	B12	0	0	kleiner LLOQ	#VALUE!	1	#VALUE!
0	T9	C01	0	0	kleiner LLOQ	#VALUE!	1	#VALUE!
0	T9	C02	0	0	kleiner LLOQ	#VALUE!	1	#VALUE!
0	T9	C03	0	0	kleiner LLOQ	#VALUE!	1	#VALUE!
0	T9	C04	0	0	kleiner LLOQ	#VALUE!	1	#VALUE!
0	T9	C05	0	0	kleiner LLOQ	#VALUE!	1	#VALUE!
0	T9	C06	0	0	kleiner LLOQ	#VALUE!	1	#VALUE!
0	T9	C07	0	0	kleiner LLOQ	#VALUE!	1	#VALUE!
0	T9	C08	0	0	kleiner LLOQ	#VALUE!	1	#VALUE!
0	T9	C09	0	0	kleiner LLOQ	#VALUE!	1	#VALUE!
0	T9	C10	0	0	kleiner LLOQ	#VALUE!	1	#VALUE!
0	T9	C11	0	0	kleiner LLOQ	#VALUE!	1	#VALUE!
0	T9	C12	0	0	kleiner LLOQ	#VALUE!	1	#VALUE!
0	T9	D01	0	0	kleiner LLOQ	#VALUE!	1	#VALUE!
0	T9	D02	0	0	kleiner LLOQ	#VALUE!	1	#VALUE!
0	T9	D03	0	0	kleiner LLOQ	#VALUE!	1	#VALUE!
0	T9	D04	0	0	kleiner LLOQ	#VALUE!	1	#VALUE!
0	T9	D05	0	0	kleiner LLOQ	#VALUE!	1	#VALUE!
0	T9	D06	0	0	kleiner LLOQ	#VALUE!	1	#VALUE!
T9SN2	T9	D07	0.331	0.280	0.280	0.012	2.75	0.032
T9SN2	T9	D08	0.343	0.292	0.292	0.012	2.75	0.034
T9SN3	T9	D09	0.621	0.570	0.570	0.025	2.75	0.070
T9SN3	T9	D10	0.661	0.610	0.610	0.027	2.75	0.075
T9SN4	T9	D11	0.691	0.641	0.641	0.029	2.75	0.079
T9SN4	T9	D12	0.644	0.593	0.593	0.027	2.75	0.073

Plate: T9

	1	2	3	4	5	6	7	8	9	10	11	12
A												
B												
C												
D							0.331	0.343	0.621	0.661	0.691	0.644
E					0.415	0.405	0.204	0.207	0.138	0.133	0.233	0.231
F					0.295	0.327	0.199	0.193	0.277	0.307	0.271	0.291
G							0.051	0.051	0.051	0.051	0.051	0.050
H							0.050	0.049	0.050	0.050	0.050	0.050

Plattenbelegung

	1	2	3	4	5	6	7	8	9	10	11	12
A												
B												
C												
D							T9SN2	T9SN2	T9SN3	T9SN3	T9SN4	T9SN4
E					T9SS11	T9SS11	T9SS12	T9SS12	T9SS13	T9SS13	T9SS14	T9SS14
F					T9SS21	T9SS21	T9SS22	T9SS22	T9SS23	T9SS23	T9SS24	T9SS24
G							Blank PEG	Blank PEG	Blank PEG	Blank PEG	Blank PEG	Blank PEG
H							Blank no PEG	Blank no PEG	Blank no PEG	Blank no PEG	Blank no PEG	Blank no PEG

Comment:

Date Operator

Date Control

0	T9	E01	0	0	kleiner LLOQ	#VALUE!	1	#VALUE!
0	T9	E02	0	0	kleiner LLOQ	#VALUE!	1	#VALUE!
0	T9	E03	0	0	kleiner LLOQ	#VALUE!	1	#VALUE!
0	T9	E04	0	0	kleiner LLOQ	#VALUE!	1	#VALUE!
T9SS11	T9	E05	0.415	0.364	0.364	0.016	2.75	0.043
T9SS11	T9	E06	0.405	0.354	0.354	0.015	2.75	0.042
T9SS12	T9	E07	0.204	0.153	kleiner LLOQ	#VALUE!	2.75	#VALUE!
T9SS12	T9	E08	0.207	0.156	kleiner LLOQ	#VALUE!	2.75	#VALUE!
T9SS13	T9	E09	0.138	0.087	kleiner LLOQ	#VALUE!	2.75	#VALUE!
T9SS13	T9	E10	0.133	0.083	kleiner LLOQ	#VALUE!	2.75	#VALUE!
T9SS14	T9	E11	0.233	0.183	0.183	0.007	2.75	0.019
T9SS14	T9	E12	0.231	0.180	0.180	0.007	2.75	0.019
0	T9	F01	0	0	kleiner LLOQ	#VALUE!	1	#VALUE!
0	T9	F02	0	0	kleiner LLOQ	#VALUE!	1	#VALUE!
0	T9	F03	0	0	kleiner LLOQ	#VALUE!	1	#VALUE!
0	T9	F04	0	0	kleiner LLOQ	#VALUE!	1	#VALUE!
T9SS21	T9	F05	0.295	0.244	0.244	0.010	2.75	0.027
T9SS21	T9	F06	0.327	0.276	0.276	0.012	2.75	0.032
T9SS22	T9	F07	0.199	0.149	kleiner LLOQ	#VALUE!	2.75	#VALUE!
T9SS22	T9	F08	0.193	0.142	kleiner LLOQ	#VALUE!	2.75	#VALUE!
T9SS23	T9	F09	0.277	0.226	0.226	0.009	2.75	0.025
T9SS23	T9	F10	0.307	0.256	0.256	0.011	2.75	0.029
T9SS24	T9	F11	0.271	0.220	0.220	0.009	2.75	0.024
T9SS24	T9	F12	0.291	0.240	0.240	0.010	2.75	0.027
0	T9	G01	0	0	kleiner LLOQ	#VALUE!	1	#VALUE!
0	T9	G02	0	0	kleiner LLOQ	#VALUE!	1	#VALUE!
0	T9	G03	0	0	kleiner LLOQ	#VALUE!	1	#VALUE!
0	T9	G04	0	0	kleiner LLOQ	#VALUE!	1	#VALUE!
0	T9	G05	0	0	kleiner LLOQ	#VALUE!	1	#VALUE!
0	T9	G06	0	0	kleiner LLOQ	#VALUE!	1	#VALUE!
Blank PEG	T9	G07	0	0	kleiner LLOQ	#VALUE!	2.75	#VALUE!
Blank PEG	T9	G08	0	0	kleiner LLOQ	#VALUE!	2.75	#VALUE!
Blank PEG	T9	G09	0	0	kleiner LLOQ	#VALUE!	2.75	#VALUE!
Blank PEG	T9	G10	0	0	kleiner LLOQ	#VALUE!	2.75	#VALUE!
Blank PEG	T9	G11	0	0	kleiner LLOQ	#VALUE!	2.75	#VALUE!
Blank PEG	T9	G12	0	0	kleiner LLOQ	#VALUE!	2.75	#VALUE!
0	T9	H01	0	0	kleiner LLOQ	#VALUE!	1	#VALUE!
0	T9	H02	0	0	kleiner LLOQ	#VALUE!	1	#VALUE!
0	T9	H03	0	0	kleiner LLOQ	#VALUE!	1	#VALUE!
0	T9	H04	0	0	kleiner LLOQ	#VALUE!	1	#VALUE!
0	T9	H05	0	0	kleiner LLOQ	#VALUE!	1	#VALUE!
0	T9	H06	0	0	kleiner LLOQ	#VALUE!	1	#VALUE!
Blank no PEG	T9	H07	0	0	kleiner LLOQ	#VALUE!	2.75	#VALUE!
Blank no PEG	T9	H08	0	0	kleiner LLOQ	#VALUE!	2.75	#VALUE!
Blank no PEG	T9	H09	0	0	kleiner LLOQ	#VALUE!	2.75	#VALUE!
Blank no PEG	T9	H10	0	0	kleiner LLOQ	#VALUE!	2.75	#VALUE!
Blank no PEG	T9	H11	0	0	kleiner LLOQ	#VALUE!	2.75	#VALUE!
Blank no PEG	T9	H12	0	0	kleiner LLOQ	#VALUE!	2.75	#VALUE!
0	T10	A01	0	0	kleiner LLOQ	#VALUE!	1	#VALUE!
0	T10	A02	0	0	kleiner LLOQ	#VALUE!	1	#VALUE!
0	T10	A03	0	0	kleiner LLOQ	#VALUE!	1	#VALUE!
0	T10	A04	0	0	kleiner LLOQ	#VALUE!	1	#VALUE!
0	T10	A05	0	0	kleiner LLOQ	#VALUE!	1	#VALUE!
0	T10	A06	0	0	kleiner LLOQ	#VALUE!	1	#VALUE!
0	T10	A07	0	0	kleiner LLOQ	#VALUE!	1	#VALUE!
0	T10	A08	0	0	kleiner LLOQ	#VALUE!	1	#VALUE!
0	T10	A09	0	0	kleiner LLOQ	#VALUE!	1	#VALUE!
0	T10	A10	0	0	kleiner LLOQ	#VALUE!	1	#VALUE!
0	T10	A11	0	0	kleiner LLOQ	#VALUE!	1	#VALUE!
0	T10	A12	0	0	kleiner LLOQ	#VALUE!	1	#VALUE!
0	T10	B01	0	0	kleiner LLOQ	#VALUE!	1	#VALUE!
0	T10	B02	0	0	kleiner LLOQ	#VALUE!	1	#VALUE!
0	T10	B03	0	0	kleiner LLOQ	#VALUE!	1	#VALUE!
0	T10	B04	0	0	kleiner LLOQ	#VALUE!	1	#VALUE!
0	T10	B05	0	0	kleiner LLOQ	#VALUE!	1	#VALUE!
0	T10	B06	0	0	kleiner LLOQ	#VALUE!	1	#VALUE!
0	T10	B07	0	0	kleiner LLOQ	#VALUE!	1	#VALUE!
0	T10	B08	0	0	kleiner LLOQ	#VALUE!	1	#VALUE!
0	T10	B09	0	0	kleiner LLOQ	#VALUE!	1	#VALUE!
0	T10	B10	0	0	kleiner LLOQ	#VALUE!	1	#VALUE!
0	T10	B11	0	0	kleiner LLOQ	#VALUE!	1	#VALUE!
0	T10	B12	0	0	kleiner LLOQ	#VALUE!	1	#VALUE!
0	T10	C01	0	0	kleiner LLOQ	#VALUE!	1	#VALUE!

Plate: T10

	1	2	3	4	5	6	7	8	9	10	11	12
A												
B												
C												
D							0.493	0.502	0.540	0.537	0.680	0.722
E					0.377	0.373	0.217	0.208			0.232	0.233
F					0.367	0.392	0.213	0.195	0.226	0.257	0.308	0.408
G							0.051	0.051	0.051	0.053	0.051	0.054
H							0.050	0.050	0.052	0.051	0.050	0.050

Plattenbelegung

	1	2	3	4	5	6	7	8	9	10	11	12
A												
B												
C												
D							T10SN2	T10SN2	T10SN3	T10SN3	T10SN4	T10SN4
E					T10SS11	T10SS11	T10SS12	T10SS12			T10SS14	T10SS14

0	T10	C02	0	0	kleiner LLOQ	#VALUE!	1	#VALUE!
0	T10	C03	0	0	kleiner LLOQ	#VALUE!	1	#VALUE!
0	T10	C04	0	0	kleiner LLOQ	#VALUE!	1	#VALUE!
0	T10	C05	0	0	kleiner LLOQ	#VALUE!	1	#VALUE!
0	T10	C06	0	0	kleiner LLOQ	#VALUE!	1	#VALUE!
0	T10	C07	0	0	kleiner LLOQ	#VALUE!	1	#VALUE!
0	T10	C08	0	0	kleiner LLOQ	#VALUE!	1	#VALUE!
0	T10	C09	0	0	kleiner LLOQ	#VALUE!	1	#VALUE!
0	T10	C10	0	0	kleiner LLOQ	#VALUE!	1	#VALUE!
0	T10	C11	0	0	kleiner LLOQ	#VALUE!	1	#VALUE!
0	T10	C12	0	0	kleiner LLOQ	#VALUE!	1	#VALUE!
0	T10	D01	0	0	kleiner LLOQ	#VALUE!	1	#VALUE!
0	T10	D02	0	0	kleiner LLOQ	#VALUE!	1	#VALUE!
0	T10	D03	0	0	kleiner LLOQ	#VALUE!	1	#VALUE!
0	T10	D04	0	0	kleiner LLOQ	#VALUE!	1	#VALUE!
0	T10	D05	0	0	kleiner LLOQ	#VALUE!	1	#VALUE!
0	T10	D06	0	0	kleiner LLOQ	#VALUE!	1	#VALUE!
T10SN2	T10	D07	0.493	0.442	0.442	0.019	2.75	0.053
T10SN2	T10	D08	0.502	0.451	0.451	0.020	2.75	0.054
T10SN3	T10	D09	0.540	0.489	0.489	0.022	2.75	0.060
T10SN3	T10	D10	0.537	0.486	0.486	0.022	2.75	0.059
T10SN4	T10	D11	0.680	0.629	0.629	0.028	2.75	0.078
T10SN4	T10	D12	0.722	0.671	0.671	0.030	2.75	0.083
0	T10	E01	0	0	kleiner LLOQ	#VALUE!	1	#VALUE!
0	T10	E02	0	0	kleiner LLOQ	#VALUE!	1	#VALUE!
0	T10	E03	0	0	kleiner LLOQ	#VALUE!	1	#VALUE!
0	T10	E04	0	0	kleiner LLOQ	#VALUE!	1	#VALUE!
T10SS11	T10	E05	0.377	0.326	0.326	0.014	2.75	0.038
T10SS11	T10	E06	0.373	0.322	0.322	0.014	2.75	0.038
T10SS12	T10	E07	0.217	0.167	kleiner LLOQ	#VALUE!	2.75	#VALUE!
T10SS12	T10	E08	0.208	0.157	kleiner LLOQ	#VALUE!	2.75	#VALUE!
0	T10	E09	0	0	kleiner LLOQ	#VALUE!	1	#VALUE!
0	T10	E10	0	0	kleiner LLOQ	#VALUE!	1	#VALUE!
T10SS14	T10	E11	0.232	0.181	0.181	0.007	2.750	0.019
T10SS14	T10	E12	0.233	0.182	0.182	0.007	2.750	0.019
0	T10	F01	0	0	kleiner LLOQ	#VALUE!	1	#VALUE!
0	T10	F02	0	0	kleiner LLOQ	#VALUE!	1	#VALUE!
0	T10	F03	0	0	kleiner LLOQ	#VALUE!	1	#VALUE!
0	T10	F04	0	0	kleiner LLOQ	#VALUE!	1	#VALUE!
T10SS21	T10	F05	0.367	0.316	0.316	0.013	2.75	0.037
T10SS21	T10	F06	0.392	0.341	0.341	0.015	2.75	0.040
T10SS22	T10	F07	0.213	0.162	kleiner LLOQ	#VALUE!	2.75	#VALUE!
T10SS22	T10	F08	0.195	0.144	kleiner LLOQ	#VALUE!	2.75	#VALUE!
T10SS23	T10	F09	0.226	0.175	0.175	0.007	2.75	0.018
T10SS23	T10	F10	0.257	0.206	0.206	0.008	2.75	0.022
T10SS24	T10	F11	0.308	0.257	0.257	0.011	2.75	0.029
T10SS24	T10	F12	0.408	0.357	0.357	0.015	2.75	0.042
0	T10	G01	0	0	kleiner LLOQ	#VALUE!	1	#VALUE!
0	T10	G02	0	0	kleiner LLOQ	#VALUE!	1	#VALUE!
0	T10	G03	0	0	kleiner LLOQ	#VALUE!	1	#VALUE!
0	T10	G04	0	0	kleiner LLOQ	#VALUE!	1	#VALUE!
0	T10	G05	0	0	kleiner LLOQ	#VALUE!	1	#VALUE!
0	T10	G06	0	0	kleiner LLOQ	#VALUE!	1	#VALUE!
Blank PEG	T10	G07	0	0	kleiner LLOQ	#VALUE!	2.75	#VALUE!
Blank PEG	T10	G08	0	0	kleiner LLOQ	#VALUE!	2.75	#VALUE!
Blank PEG	T10	G09	0	0	kleiner LLOQ	#VALUE!	2.75	#VALUE!
Blank PEG	T10	G10	0	0	kleiner LLOQ	#VALUE!	2.75	#VALUE!
Blank PEG	T10	G11	0	0	kleiner LLOQ	#VALUE!	2.75	#VALUE!
Blank PEG	T10	G12	0	0	kleiner LLOQ	#VALUE!	2.75	#VALUE!
0	T10	H01	0	0	kleiner LLOQ	#VALUE!	1	#VALUE!
0	T10	H02	0	0	kleiner LLOQ	#VALUE!	1	#VALUE!
0	T10	H03	0	0	kleiner LLOQ	#VALUE!	1	#VALUE!
0	T10	H04	0	0	kleiner LLOQ	#VALUE!	1	#VALUE!
0	T10	H05	0	0	kleiner LLOQ	#VALUE!	1	#VALUE!
0	T10	H06	0	0	kleiner LLOQ	#VALUE!	1	#VALUE!
Blank no PEG	T10	H07	0	0	kleiner LLOQ	#VALUE!	2.75	#VALUE!
Blank no PEG	T10	H08	0	0	kleiner LLOQ	#VALUE!	2.75	#VALUE!
Blank no PEG	T10	H09	0	0	kleiner LLOQ	#VALUE!	2.75	#VALUE!
Blank no PEG	T10	H10	0	0	kleiner LLOQ	#VALUE!	2.75	#VALUE!
Blank no PEG	T10	H11	0	0	kleiner LLOQ	#VALUE!	2.75	#VALUE!
Blank no PEG	T10	H12	0	0	kleiner LLOQ	#VALUE!	2.75	#VALUE!
0	T11	A01	0	0	kleiner LLOQ	#VALUE!	1	#VALUE!
0	T11	A02	0	0	kleiner LLOQ	#VALUE!	1	#VALUE!
0	T11	A03	0	0	kleiner LLOQ	#VALUE!	1	#VALUE!
0	T11	A04	0	0	kleiner LLOQ	#VALUE!	1	#VALUE!
0	T11	A05	0	0	kleiner LLOQ	#VALUE!	1	#VALUE!

F					T10SS21	T10SS21	T10SS22	T10SS22	T10SS23	T10SS23	T10SS24	T10SS24
G							Blank PEG	Blank PEG	Blank PEG	Blank PEG	Blank PEG	Blank PEG
H							Blank no PEG	Blank no PEG	Blank no PEG	Blank no PEG	Blank no PEG	Blank no PEG

Comment:

Date Operator

Date Control

0	T11	A06	0	0	kleiner LLOQ	#VALUE!	1	#VALUE!
0	T11	A07	0	0	kleiner LLOQ	#VALUE!	1	#VALUE!
0	T11	A08	0	0	kleiner LLOQ	#VALUE!	1	#VALUE!
0	T11	A09	0	0	kleiner LLOQ	#VALUE!	1	#VALUE!
0	T11	A10	0	0	kleiner LLOQ	#VALUE!	1	#VALUE!
0	T11	A11	0	0	kleiner LLOQ	#VALUE!	1	#VALUE!
0	T11	A12	0	0	kleiner LLOQ	#VALUE!	1	#VALUE!
0	T11	B01	0	0	kleiner LLOQ	#VALUE!	1	#VALUE!
0	T11	B02	0	0	kleiner LLOQ	#VALUE!	1	#VALUE!
0	T11	B03	0	0	kleiner LLOQ	#VALUE!	1	#VALUE!
0	T11	B04	0	0	kleiner LLOQ	#VALUE!	1	#VALUE!
0	T11	B05	0	0	kleiner LLOQ	#VALUE!	1	#VALUE!
0	T11	B06	0	0	kleiner LLOQ	#VALUE!	1	#VALUE!
0	T11	B07	0	0	kleiner LLOQ	#VALUE!	1	#VALUE!
0	T11	B08	0	0	kleiner LLOQ	#VALUE!	1	#VALUE!
0	T11	B09	0	0	kleiner LLOQ	#VALUE!	1	#VALUE!
0	T11	B10	0	0	kleiner LLOQ	#VALUE!	1	#VALUE!
0	T11	B11	0	0	kleiner LLOQ	#VALUE!	1	#VALUE!
0	T11	B12	0	0	kleiner LLOQ	#VALUE!	1	#VALUE!
0	T11	C01	0	0	kleiner LLOQ	#VALUE!	1	#VALUE!
0	T11	C02	0	0	kleiner LLOQ	#VALUE!	1	#VALUE!
0	T11	C03	0	0	kleiner LLOQ	#VALUE!	1	#VALUE!
0	T11	C04	0	0	kleiner LLOQ	#VALUE!	1	#VALUE!
0	T11	C05	0	0	kleiner LLOQ	#VALUE!	1	#VALUE!
0	T11	C06	0	0	kleiner LLOQ	#VALUE!	1	#VALUE!
0	T11	C07	0	0	kleiner LLOQ	#VALUE!	1	#VALUE!
0	T11	C08	0	0	kleiner LLOQ	#VALUE!	1	#VALUE!
0	T11	C09	0	0	kleiner LLOQ	#VALUE!	1	#VALUE!
0	T11	C10	0	0	kleiner LLOQ	#VALUE!	1	#VALUE!
0	T11	C11	0	0	kleiner LLOQ	#VALUE!	1	#VALUE!
0	T11	C12	0	0	kleiner LLOQ	#VALUE!	1	#VALUE!
0	T11	D01	0	0	kleiner LLOQ	#VALUE!	1	#VALUE!
0	T11	D02	0	0	kleiner LLOQ	#VALUE!	1	#VALUE!
0	T11	D03	0	0	kleiner LLOQ	#VALUE!	1	#VALUE!
0	T11	D04	0	0	kleiner LLOQ	#VALUE!	1	#VALUE!
0	T11	D05	0	0	kleiner LLOQ	#VALUE!	1	#VALUE!
0	T11	D06	0	0	kleiner LLOQ	#VALUE!	1	#VALUE!
T11SN2	T11	D07	0.358	0.307	0.307	0.013	2.75	0.036
T11SN2	T11	D08	0.336	0.285	0.285	0.012	2.75	0.033
T11SN3	T11	D09	0.471	0.420	0.420	0.018	2.75	0.050
T11SN3	T11	D10	0.480	0.429	0.429	0.019	2.75	0.052
T11SN4	T11	D11	0.747	0.696	0.696	0.031	2.75	0.087
T11SN4	T11	D12	0.716	0.665	0.665	0.030	2.75	0.083
0	T11	E01	0	0	kleiner LLOQ	#VALUE!	1	#VALUE!
0	T11	E02	0	0	kleiner LLOQ	#VALUE!	1	#VALUE!
0	T11	E03	0	0	kleiner LLOQ	#VALUE!	1	#VALUE!
0	T11	E04	0	0	kleiner LLOQ	#VALUE!	1	#VALUE!
T11SS11	T11	E05	0.423	0.372	0.372	0.016	2.75	0.044
T11SS11	T11	E06	0.402	0.351	0.351	0.015	2.75	0.041
T11SS12	T11	E07	0.227	0.176	0.176	0.007	2.75	0.019
T11SS12	T11	E08	0.267	0.216	0.216	0.009	2.75	0.024
T11SS13	T11	E09	0.154	0.103	kleiner LLOQ	#VALUE!	2.75	#VALUE!
T11SS13	T11	E10	0.156	0.105	kleiner LLOQ	#VALUE!	2.75	#VALUE!
T11SS14	T11	E11	0.234	0.183	0.183	0.007	2.75	0.020
T11SS14	T11	E12	0.235	0.184	0.184	0.007	2.75	0.020
0	T11	F01	0	0	kleiner LLOQ	#VALUE!	1	#VALUE!
0	T11	F02	0	0	kleiner LLOQ	#VALUE!	1	#VALUE!
0	T11	F03	0	0	kleiner LLOQ	#VALUE!	1	#VALUE!
0	T11	F04	0	0	kleiner LLOQ	#VALUE!	1	#VALUE!
T11SS21	T11	F05	0.354	0.303	0.303	0.013	2.75	0.035
T11SS21	T11	F06	0.398	0.347	0.347	0.015	2.75	0.041
T11SS22	T11	F07	0.217	0.166	kleiner LLOQ	#VALUE!	2.75	#VALUE!
T11SS22	T11	F08	0.229	0.178	0.178	0.007	2.75	0.019
T11SS23	T11	F09	0.248	0.198	0.198	0.008	2.75	0.021
T11SS23	T11	F10	0.272	0.221	0.221	0.009	2.75	0.024
T11SS24	T11	F11	0.308	0.257	0.257	0.011	2.75	0.029
T11SS24	T11	F12	0.317	0.266	0.266	0.011	2.75	0.030
0	T11	G01	0	0	kleiner LLOQ	#VALUE!	1	#VALUE!
0	T11	G02	0	0	kleiner LLOQ	#VALUE!	1	#VALUE!
0	T11	G03	0	0	kleiner LLOQ	#VALUE!	1	#VALUE!
0	T11	G04	0	0	kleiner LLOQ	#VALUE!	1	#VALUE!
0	T11	G05	0	0	kleiner LLOQ	#VALUE!	1	#VALUE!
0	T11	G06	0	0	kleiner LLOQ	#VALUE!	1	#VALUE!
Blank PEG	T11	G07	0	0	kleiner LLOQ	#VALUE!	2.75	#VALUE!

Plate: T11

	1	2	3	4	5	6	7	8	9	10	11	12
A												
B												
C												
D							0.358	0.336	0.471	0.480	0.747	0.716
E					0.423	0.402	0.227	0.267	0.154	0.156	0.234	0.235
F					0.354	0.398	0.217	0.229	0.248	0.272	0.308	0.317
G							0.052	0.052	0.052	0.056	0.052	0.052
H							0.050	0.050	0.050	0.050	0.050	0.049

Plattenbelegung

	1	2	3	4	5	6	7	8	9	10	11	12
A												
B												
C												
D							T11SN2	T11SN2	T11SN3	T11SN3	T11SN4	T11SN4
E					T11SS11	T11SS11	T11SS12	T11SS12	T11SS13	T11SS13	T11SS14	T11SS14
F					T11SS21	T11SS21	T11SS22	T11SS22	T11SS23	T11SS23	T11SS24	T11SS24
G							Blank PEG	Blank PEG	Blank PEG	Blank PEG	Blank PEG	Blank PEG
H							Blank no PEG	Blank no PEG	Blank no PEG	Blank no PEG	Blank no PEG	Blank no PEG

Comment:

Date Operator

Date Control

Blank PEG	T11	G08	0	0	kleiner LLOQ	#VALUE!	2.75	#VALUE!
Blank PEG	T11	G09	0	0	kleiner LLOQ	#VALUE!	2.75	#VALUE!
Blank PEG	T11	G10	0	0	kleiner LLOQ	#VALUE!	2.75	#VALUE!
Blank PEG	T11	G11	0	0	kleiner LLOQ	#VALUE!	2.75	#VALUE!
Blank PEG	T11	G12	0	0	kleiner LLOQ	#VALUE!	2.75	#VALUE!
0	T11	H01	0	0	kleiner LLOQ	#VALUE!	1	#VALUE!
0	T11	H02	0	0	kleiner LLOQ	#VALUE!	1	#VALUE!
0	T11	H03	0	0	kleiner LLOQ	#VALUE!	1	#VALUE!
0	T11	H04	0	0	kleiner LLOQ	#VALUE!	1	#VALUE!
0	T11	H05	0	0	kleiner LLOQ	#VALUE!	1	#VALUE!
0	T11	H06	0	0	kleiner LLOQ	#VALUE!	1	#VALUE!
Blank no PEG	T11	H07	0	0	kleiner LLOQ	#VALUE!	2.75	#VALUE!
Blank no PEG	T11	H08	0	0	kleiner LLOQ	#VALUE!	2.75	#VALUE!
Blank no PEG	T11	H09	0	0	kleiner LLOQ	#VALUE!	2.75	#VALUE!
Blank no PEG	T11	H10	0	0	kleiner LLOQ	#VALUE!	2.75	#VALUE!
Blank no PEG	T11	H11	0	0	kleiner LLOQ	#VALUE!	2.75	#VALUE!
Blank no PEG	T11	H12	0	0	kleiner LLOQ	#VALUE!	2.75	#VALUE!

Project number		F-120	Apparatus	Wallac Victor	Operator	IsBa
GLP Study (Number)		n.a.	Protocol (Instrument meth	LDH test 2016	Date of preparation	19-04-18
hot substance	isotope	n.a.	File name (results)	IsBa_180419/20_LDH_full_	Date of measurement	19-04-18
	name	n.a.	Kind of well plate	96 well	shaking time [min]	30
	ACB-ID	n.a.	sample volume [µL]	100	stirring rate (Target) [rpm]	150
	Batch number	n.a.	Cocktail volume [µL]	175	Kind of measurement	UV-vis
cold substance	name	LDH	ACB-ID of cocktail		Wave length [nm]	450
	ACB-ID	L23982R	Matrix	DMEM (from powder)+PE(	Remarks	Cocktail 100µl RM, 75µl STOP
	Batch number	SLBK6345V	Blank description	DMEM/PEG, H2O	Remarks	7 standards split low/high
n.a.		n.a.	Pipettes (No. / volume)	50-200µl	Remarks	KLP4 common for both
n.a.		n.a.	Pipettes (No. / volume)	n.a.	Remarks	n.a.

Messdaten (diese Tabelle in Bericht übernehmen)

Sample name *	concentration (theor.) *	measured data	measured data	measured data	mean measured	SD	RSD	Blank *	measured data after * Blank subtraction	concentration (calc.) *	Deviation *	Residuen
	[µg/mL]	[AU]	[AU]	[AU]	[AU]	[AU]	[%]	[AU]		[µg/mL]	[%]	
KLP1	0.148	1.579	1.652	1.627	1.62	0.03	1.87	0.047	1.568	0.148	-0.24	0.00
KLP2	0.114	1.357	1.443	1.372	1.39	0.04	2.70	0.054	1.340	0.110	-3.40	0.00
KLP3	0.074	1.272	1.193	1.228	1.23	0.03	2.63	0.051	1.180	0.084	13.04	0.01
KLP4	0.041	0.959	0.972	0.883	0.94	0.04	4.19	0.051	0.887	0.035	-13.32	-0.01
KLP5								0.052				
KLP6								0.051				
KLP7												
KLP8												

Statistical data

Geradensteigung	Slope	m	6.05
Y-Achsenabschnitt	Y-Intercept	b	0.67
Standardabw. Geradensteigung	SD-Slope	s_m	0.620291227
Standardabw. Achsenabschnittes	SD-Y-Intercept	s_b	0.063694294
Anzahl Messpunkte	number of measuring points	n	4
Quadratsumme	sum of squares	Qxx	0.006578604
Bereichsmitte			0.094336926
Freiheitsgrade	degree of freedom	f	2
Student-t-Faktor für (P = 95 %; f = n-2)	Student-t-factor for (P = 95 %; f = n-2)	t	4.303
Vertrauensbereich Steig. (95 %) Obergrenze		$m + VB_m$	8.722319527
Vertrauensbereich Steig. (95 %) Untergrenze		$m - VB_m$	3.384093228
Vertrauensbereich Achsenabschnitt (95 %) Og.		$b + Vb_b$	0.946914615
Vertrauensbereich Achsenabschnitt (95 %) Ug.		$b - Vb_b$	0.398761518
Korrelationskoeffizient	correlation coefficient	r	0.9897
Bestimmtheitsmaß	determiantion coefficient	r^2	0.9794
Reststandardabweichung		s_0	0.050310948
Summe Restquadrate		sd	2.895583345
Verfahrensstandardabw.		s_{x0}	0.008311454
Rel. Verfahrensstandardabw. %		V_{x0}	8.810393374

mean Blank	0	
SD Blank	0.00	
RSD Blank	3.79	x=
x*SD (LLOQ)	0	5
x*SD (LOD)	0	3
**LLOQ (AU)**	0	
**LOD (AU)**	0	
ULOQ	1.568	
LLOQ (Lin)	0.887	

Evaluation / Comment

LDH linearity valid with 7 standards, split in lin high and lin low, each with 4 standards and KLP4 common standard for both. R² 0,9981, deviations for both between -13.34% and +13.04%

Date	Operator	Date	Control

Figure 1 Linearity

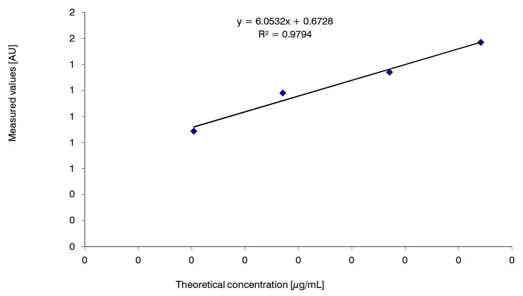

Figure 2 Method validation Residuen Plot

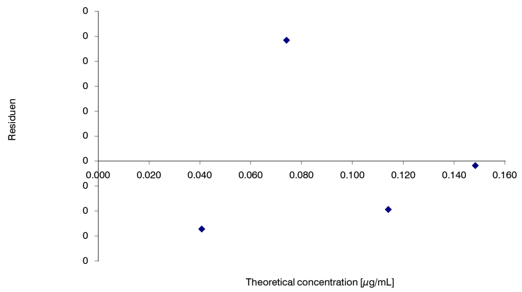

Evaluation / Comment

lin passed

Date Operator Date Control

Sample name	Plate	Position	[AU]	[AU]-Blank	Rating	[µg/mL]	Dilution factor	[µg/mL]
0	T8	A01	0	0	kleiner LLOQ	#VALUE!	1	#VALUE!
0	T8	A02	0	0	kleiner LLOQ	#VALUE!	1	#VALUE!
0	T8	A03	0	0	kleiner LLOQ	#VALUE!	1	#VALUE!
0	T8	A04	0	0	kleiner LLOQ	#VALUE!	1	#VALUE!
0	T8	A05	0.000	-0.051	kleiner LLOQ	#VALUE!		#VALUE!
0	T8	A06	0.000	-0.051	kleiner LLOQ	#VALUE!		#VALUE!
0	T8	A07	0	0	kleiner LLOQ	#VALUE!		#VALUE!
0	T8	A08	0	0	kleiner LLOQ	#VALUE!		#VALUE!
0	T8	A09	0.000	-0.051	kleiner LLOQ	#VALUE!		#VALUE!
0	T8	A10	0.000	-0.051	kleiner LLOQ	#VALUE!		#VALUE!
0	T8	A11	0	0	kleiner LLOQ	#VALUE!		#VALUE!
0	T8	A12	0	0	kleiner LLOQ	#VALUE!		#VALUE!
0	T8	B01	0	0	kleiner LLOQ	#VALUE!		#VALUE!
0	T8	B02	0	0	kleiner LLOQ	#VALUE!		#VALUE!
0	T8	B03	0	0	kleiner LLOQ	#VALUE!		#VALUE!
0	T8	B04	0	0	kleiner LLOQ	#VALUE!		#VALUE!
0	T8	B05	0	0	kleiner LLOQ	#VALUE!		#VALUE!
0	T8	B06	0.000	-0.051	kleiner LLOQ	#VALUE!		#VALUE!
0	T8	B07	0.000	-0.051	kleiner LLOQ	#VALUE!		#VALUE!
0	T8	B08	0.000	-0.051	kleiner LLOQ	#VALUE!		#VALUE!
0	T8	B09	0	0	kleiner LLOQ	#VALUE!		#VALUE!
0	T8	B10	0	0	kleiner LLOQ	#VALUE!		#VALUE!
0	T8	B11	0	0	kleiner LLOQ	#VALUE!		#VALUE!
0	T8	B12	0	0	kleiner LLOQ	#VALUE!		#VALUE!
0	T8	C01	0	0	kleiner LLOQ	#VALUE!		#VALUE!
0	T8	C02	0	0	kleiner LLOQ	#VALUE!		#VALUE!
0	T8	C03	0	0	kleiner LLOQ	#VALUE!		#VALUE!
0	T8	C04	0	0	kleiner LLOQ	#VALUE!		#VALUE!
0	T8	C05	0.000	-0.051	kleiner LLOQ	#VALUE!		#VALUE!
0	T8	C06	0.000	-0.051	kleiner LLOQ	#VALUE!		#VALUE!
0	T8	C07	0.000	-0.051	kleiner LLOQ	#VALUE!		#VALUE!
0	T8	C08	0.000	-0.051	kleiner LLOQ	#VALUE!		#VALUE!
0	T8	C09	0.000	-0.051	kleiner LLOQ	#VALUE!		#VALUE!
0	T8	C10	0.000	-0.051	kleiner LLOQ	#VALUE!		#VALUE!
0	T8	C11	0	0	kleiner LLOQ	#VALUE!	1	#VALUE!
0	T8	C12	0	0	kleiner LLOQ	#VALUE!	1	#VALUE!
0	T8	D01	0	0	kleiner LLOQ	#VALUE!	1	#VALUE!
0	T8	D02	0	0	kleiner LLOQ	#VALUE!	1	#VALUE!
0	T8	D03	0	0	kleiner LLOQ	#VALUE!	1	#VALUE!
0	T8	D04	0	0	kleiner LLOQ	#VALUE!	1	#VALUE!
T8SN1	T8	D05	0	0	kleiner LLOQ	#VALUE!	1	#VALUE!
T8SN1	T8	D06	0	0	kleiner LLOQ	#VALUE!	1	#VALUE!
T8SN2	T8	D07	0	0	kleiner LLOQ	#VALUE!	1	#VALUE!
T8SN2	T8	D08	0	0	kleiner LLOQ	#VALUE!	1	#VALUE!
T8SN3	T8	D09	0	0	kleiner LLOQ	#VALUE!	1	#VALUE!
T8SN3	T8	D10	0	0	kleiner LLOQ	#VALUE!	1	#VALUE!
T8SN4	T8	D11	0	0	kleiner LLOQ	#VALUE!	1	#VALUE!
T8SN4	T8	D12	0	0	kleiner LLOQ	#VALUE!	1	#VALUE!

Plate: T8

	1	2	3	4	5	6	7	8	9	10	11	12
A												
B												
C												
D												
E												
F												
G												
H												

Plattenbelegung

	1	2	3	4	5	6	7	8	9	10	11	12
A												
B												
C												
D					T8SN1	T8SN1	T8SN2	T8SN2	T8SN3	T8SN3	T8SN4	T8SN4
E					T8SS11	T8SS11	T8SS12	T8SS12	T8SS13	T8SS13	T8SS14	T8SS14
F					T8SS21	T8SS21	T8SS22	T8SS22	T8SS23	T8SS23	T8SS24	T8SS24
G							Blank PEG	Blank PEG	Blank PEG	Blank PEG	Blank PEG	Blank PEG
H							Blank no PEG	Blank no PEG	Blank no PEG	Blank no PEG	Blank no PEG	Blank no PEG

Comment:

alle Messwerte bei lin low

Samples aufgeteilt in lin high und low:niedriger konzentrierte Samples bei lin low zu finden.

Date Operator

Date Control

0	T8	E01	0	0	kleiner LLOQ	#VALUE!	1	#VALUE!
0	T8	E02	0	0	kleiner LLOQ	#VALUE!	1	#VALUE!
0	T8	E03	0	0	kleiner LLOQ	#VALUE!	1	#VALUE!
0	T8	E04	0	0	kleiner LLOQ	#VALUE!	1	#VALUE!
T8SS11	T8	E05	0	0	kleiner LLOQ	#VALUE!	1	#VALUE!
T8SS11	T8	E06	0	0	kleiner LLOQ	#VALUE!	1	#VALUE!
T8SS12	T8	E07	0	0	kleiner LLOQ	#VALUE!	1	#VALUE!
T8SS12	T8	E08	0	0	kleiner LLOQ	#VALUE!	1	#VALUE!
T8SS13	T8	E09	0	0	kleiner LLOQ	#VALUE!	1	#VALUE!
T8SS13	T8	E10	0	0	kleiner LLOQ	#VALUE!	1	#VALUE!
T8SS14	T8	E11	0	0	kleiner LLOQ	#VALUE!	1	#VALUE!
T8SS14	T8	E12	0	0	kleiner LLOQ	#VALUE!	1	#VALUE!
0	T8	F01	0	0	kleiner LLOQ	#VALUE!	1	#VALUE!
0	T8	F02	0	0	kleiner LLOQ	#VALUE!	1	#VALUE!
0	T8	F03	0	0	kleiner LLOQ	#VALUE!	1	#VALUE!
0	T8	F04	0	0	kleiner LLOQ	#VALUE!	1	#VALUE!
T8SS21	T8	F05	0	0	kleiner LLOQ	#VALUE!	1	#VALUE!
T8SS21	T8	F06	0	0	kleiner LLOQ	#VALUE!	1	#VALUE!
T8SS22	T8	F07	0	0	kleiner LLOQ	#VALUE!	1	#VALUE!
T8SS22	T8	F08	0	0	kleiner LLOQ	#VALUE!	1	#VALUE!
T8SS23	T8	F09	0	0	kleiner LLOQ	#VALUE!	1	#VALUE!
T8SS23	T8	F10	0	0	kleiner LLOQ	#VALUE!	1	#VALUE!
T8SS24	T8	F11	0	0	kleiner LLOQ	#VALUE!	1	#VALUE!
T8SS24	T8	F12	0	0	kleiner LLOQ	#VALUE!	1	#VALUE!
0	T8	G01	0	0	kleiner LLOQ	#VALUE!	1	#VALUE!
0	T8	G02	0	0	kleiner LLOQ	#VALUE!	1	#VALUE!
0	T8	G03	0	0	kleiner LLOQ	#VALUE!	1	#VALUE!
0	T8	G04	0	0	kleiner LLOQ	#VALUE!	1	#VALUE!
0	T8	G05	0	0	kleiner LLOQ	#VALUE!	1	#VALUE!
0	T8	G06	0	0	kleiner LLOQ	#VALUE!	1	#VALUE!
Blank PEG	T8	G07	0.000	-0.051	kleiner LLOQ	#VALUE!	2.75	#VALUE!
Blank PEG	T8	G08	0.000	-0.051	kleiner LLOQ	#VALUE!	2.75	#VALUE!
Blank PEG	T8	G09	0.000	-0.051	kleiner LLOQ	#VALUE!	2.75	#VALUE!
Blank PEG	T8	G10	0.000	-0.051	kleiner LLOQ	#VALUE!	2.75	#VALUE!
Blank PEG	T8	G11	0.000	-0.051	kleiner LLOQ	#VALUE!	2.75	#VALUE!
Blank PEG	T8	G12	0.000	-0.051	kleiner LLOQ	#VALUE!	2.75	#VALUE!
0	T8	H01	0	0	kleiner LLOQ	#VALUE!	1	#VALUE!
0	T8	H02	0	0	kleiner LLOQ	#VALUE!	1	#VALUE!
0	T8	H03	0	0	kleiner LLOQ	#VALUE!	1	#VALUE!
0	T8	H04	0	0	kleiner LLOQ	#VALUE!	1	#VALUE!
0	T8	H05	0	0	kleiner LLOQ	#VALUE!	1	#VALUE!
0	T8	H06	0	0	kleiner LLOQ	#VALUE!	1	#VALUE!
Blank no PEG	T8	H07	0.000	-0.051	kleiner LLOQ	#VALUE!	2.75	#VALUE!
Blank no PEG	T8	H08	0.000	-0.051	kleiner LLOQ	#VALUE!	2.75	#VALUE!
Blank no PEG	T8	H09	0.000	-0.051	kleiner LLOQ	#VALUE!	2.75	#VALUE!
Blank no PEG	T8	H10	0.000	-0.051	kleiner LLOQ	#VALUE!	2.75	#VALUE!
Blank no PEG	T8	H11	0.000	-0.051	kleiner LLOQ	#VALUE!	2.75	#VALUE!
Blank no PEG	T8	H12	0.000	-0.051	kleiner LLOQ	#VALUE!	2.75	#VALUE!

0	T9	A01	0	0	kleiner LLOQ	#VALUE!	2	#VALUE!
0	T9	A02	0	0	kleiner LLOQ	#VALUE!	3	#VALUE!
0	T9	A03	0	0	kleiner LLOQ	#VALUE!	4	#VALUE!
0	T9	A04	0	0	kleiner LLOQ	#VALUE!	5	#VALUE!
0	T9	A05	0	0	kleiner LLOQ	#VALUE!	6	#VALUE!
0	T9	A06	0	0	kleiner LLOQ	#VALUE!	7	#VALUE!
0	T9	A07	0	0	kleiner LLOQ	#VALUE!	8	#VALUE!
0	T9	A08	0	0	kleiner LLOQ	#VALUE!	9	#VALUE!
0	T9	A09	0	0	kleiner LLOQ	#VALUE!	10	#VALUE!
0	T9	A10	0	0	kleiner LLOQ	#VALUE!	11	#VALUE!
0	T9	A11	0	0	kleiner LLOQ	#VALUE!	12	#VALUE!
0	T9	A12	0	0	kleiner LLOQ	#VALUE!	13	#VALUE!
0	T9	B01	0	0	kleiner LLOQ	#VALUE!	14	#VALUE!
0	T9	B02	0	0	kleiner LLOQ	#VALUE!	15	#VALUE!
0	T9	B03	0	0	kleiner LLOQ	#VALUE!	16	#VALUE!
0	T9	B04	0	0	kleiner LLOQ	#VALUE!	17	#VALUE!
0	T9	B05	0	0	kleiner LLOQ	#VALUE!	18	#VALUE!
0	T9	B06	0	0	kleiner LLOQ	#VALUE!	19	#VALUE!
0	T9	B07	0	0	kleiner LLOQ	#VALUE!	20	#VALUE!
0	T9	B08	0	0	kleiner LLOQ	#VALUE!	21	#VALUE!
0	T9	B09	0	0	kleiner LLOQ	#VALUE!	22	#VALUE!
0	T9	B10	0	0	kleiner LLOQ	#VALUE!	23	#VALUE!
0	T9	B11	0	0	kleiner LLOQ	#VALUE!	24	#VALUE!
0	T9	B12	0	0	kleiner LLOQ	#VALUE!	25	#VALUE!
0	T9	C01	0	0	kleiner LLOQ	#VALUE!	26	#VALUE!
0	T9	C02	0	0	kleiner LLOQ	#VALUE!	27	#VALUE!
0	T9	C03	0	0	kleiner LLOQ	#VALUE!	28	#VALUE!
0	T9	C04	0	0	kleiner LLOQ	#VALUE!	29	#VALUE!
0	T9	C05	0	0	kleiner LLOQ	#VALUE!	30	#VALUE!
0	T9	C06	0	0	kleiner LLOQ	#VALUE!	31	#VALUE!
0	T9	C07	0	0	kleiner LLOQ	#VALUE!	32	#VALUE!
0	T9	C08	0	0	kleiner LLOQ	#VALUE!	33	#VALUE!
0	T9	C09	0	0	kleiner LLOQ	#VALUE!	34	#VALUE!
0	T9	C10	0	0	kleiner LLOQ	#VALUE!	35	#VALUE!
0	T9	C11	0	0	kleiner LLOQ	#VALUE!	36	#VALUE!
0	T9	C12	0	0	kleiner LLOQ	#VALUE!	37	#VALUE!
0	T9	D01	0	0	kleiner LLOQ	#VALUE!	38	#VALUE!
0	T9	D02	0	0	kleiner LLOQ	#VALUE!	39	#VALUE!
0	T9	D03	0	0	kleiner LLOQ	#VALUE!	40	#VALUE!
0	T9	D04	0	0	kleiner LLOQ	#VALUE!	41	#VALUE!
0	T9	D05	0	0	kleiner LLOQ	#VALUE!	42	#VALUE!
0	T9	D06	0	0	kleiner LLOQ	#VALUE!	43	#VALUE!
T9SN2	T9	D07	0	0	kleiner LLOQ	#VALUE!	44	#VALUE!
T9SN2	T9	D08	0	0	kleiner LLOQ	#VALUE!	45	#VALUE!
T9SN3	T9	D09	0	0	kleiner LLOQ	#VALUE!	46	#VALUE!
T9SN3	T9	D10	0	0	kleiner LLOQ	#VALUE!	47	#VALUE!
T9SN4	T9	D11	0	0	kleiner LLOQ	#VALUE!	48	#VALUE!
T9SN4	T9	D12	0	0	kleiner LLOQ	#VALUE!	49	#VALUE!

Plate: T9

	1	2	3	4	5	6	7	8	9	10	11	12
A												
B												
C												
D												
E												
F												
G												
H												

Plattenbelegung

	1	2	3	4	5	6	7	8	9	10	11	12
A												
B												
C												
D							T9SN2	T9SN2	T9SN3	T9SN3	T9SN4	T9SN4
E					T9SS11	T9SS11	T9SS12	T9SS12	T9SS13	T9SS13	T9SS14	T9SS14
F					T9SS21	T9SS21	T9SS22	T9SS22	T9SS23	T9SS23	T9SS24	T9SS24
G							Blank PEG	Blank PEG	Blank PEG	Blank PEG	Blank PEG	Blank PEG
H							Blank no PEG	Blank no PEG	Blank no PEG	Blank no PEG	Blank no PEG	Blank no PEG

Comment:

alle Messwerte bei lin low

Date Operator

Date Control

0	T9	E01	0	0	kleiner LLOQ	#VALUE!	50	#VALUE!
0	T9	E02	0	0	kleiner LLOQ	#VALUE!	51	#VALUE!
0	T9	E03	0	0	kleiner LLOQ	#VALUE!	52	#VALUE!
0	T9	E04	0	0	kleiner LLOQ	#VALUE!	53	#VALUE!
T9SS11	T9	E05	0	0	kleiner LLOQ	#VALUE!	54	#VALUE!
T9SS11	T9	E06	0	0	kleiner LLOQ	#VALUE!	55	#VALUE!
T9SS12	T9	E07	0	0	kleiner LLOQ	#VALUE!	56	#VALUE!
T9SS12	T9	E08	0	0	kleiner LLOQ	#VALUE!	57	#VALUE!
T9SS13	T9	E09	0	0	kleiner LLOQ	#VALUE!	58	#VALUE!
T9SS13	T9	E10	0	0	kleiner LLOQ	#VALUE!	59	#VALUE!
T9SS14	T9	E11	0	0	kleiner LLOQ	#VALUE!	60	#VALUE!
T9SS14	T9	E12	0	0	kleiner LLOQ	#VALUE!	61	#VALUE!
0	T9	F01	0	0	kleiner LLOQ	#VALUE!	62	#VALUE!
0	T9	F02	0	0	kleiner LLOQ	#VALUE!	63	#VALUE!
0	T9	F03	0	0	kleiner LLOQ	#VALUE!	64	#VALUE!
0	T9	F04	0	0	kleiner LLOQ	#VALUE!	65	#VALUE!
T9SS21	T9	F05	0	0	kleiner LLOQ	#VALUE!	66	#VALUE!
T9SS21	T9	F06	0	0	kleiner LLOQ	#VALUE!	67	#VALUE!
T9SS22	T9	F07	0	0	kleiner LLOQ	#VALUE!	68	#VALUE!
T9SS22	T9	F08	0	0	kleiner LLOQ	#VALUE!	69	#VALUE!
T9SS23	T9	F09	0	0	kleiner LLOQ	#VALUE!	70	#VALUE!
T9SS23	T9	F10	0	0	kleiner LLOQ	#VALUE!	71	#VALUE!
T9SS24	T9	F11	0	0	kleiner LLOQ	#VALUE!	72	#VALUE!
T9SS24	T9	F12	0	0	kleiner LLOQ	#VALUE!	73	#VALUE!
0	T9	G01	0	0	kleiner LLOQ	#VALUE!	74	#VALUE!
0	T9	G02	0	0	kleiner LLOQ	#VALUE!	75	#VALUE!
0	T9	G03	0	0	kleiner LLOQ	#VALUE!	76	#VALUE!
0	T9	G04	0	0	kleiner LLOQ	#VALUE!	77	#VALUE!
0	T9	G05	0	0	kleiner LLOQ	#VALUE!	78	#VALUE!
0	T9	G06	0	0	kleiner LLOQ	#VALUE!	79	#VALUE!
Blank PEG	T9	G07	0	0	kleiner LLOQ	#VALUE!	80	#VALUE!
Blank PEG	T9	G08	0	0	kleiner LLOQ	#VALUE!	81	#VALUE!
Blank PEG	T9	G09	0	0	kleiner LLOQ	#VALUE!	82	#VALUE!
Blank PEG	T9	G10	0	0	kleiner LLOQ	#VALUE!	83	#VALUE!
Blank PEG	T9	G11	0	0	kleiner LLOQ	#VALUE!	84	#VALUE!
Blank PEG	T9	G12	0	0	kleiner LLOQ	#VALUE!	85	#VALUE!
0	T9	H01	0	0	kleiner LLOQ	#VALUE!	86	#VALUE!
0	T9	H02	0	0	kleiner LLOQ	#VALUE!	87	#VALUE!
0	T9	H03	0	0	kleiner LLOQ	#VALUE!	88	#VALUE!
0	T9	H04	0	0	kleiner LLOQ	#VALUE!	89	#VALUE!
0	T9	H05	0	0	kleiner LLOQ	#VALUE!	90	#VALUE!
0	T9	H06	0	0	kleiner LLOQ	#VALUE!	91	#VALUE!
Blank no PEG	T9	H07	0	0	kleiner LLOQ	#VALUE!	92	#VALUE!
Blank no PEG	T9	H08	0	0	kleiner LLOQ	#VALUE!	93	#VALUE!
Blank no PEG	T9	H09	0	0	kleiner LLOQ	#VALUE!	94	#VALUE!
Blank no PEG	T9	H10	0	0	kleiner LLOQ	#VALUE!	95	#VALUE!
Blank no PEG	T9	H11	0	0	kleiner LLOQ	#VALUE!	96	#VALUE!
Blank no PEG	T9	H12	0	0	kleiner LLOQ	#VALUE!	97	#VALUE!
0	T10	A01	0	0	kleiner LLOQ	#VALUE!	98	#VALUE!
0	T10	A02	0	0	kleiner LLOQ	#VALUE!	99	#VALUE!
0	T10	A03	0	0	kleiner LLOQ	#VALUE!	100	#VALUE!
0	T10	A04	0	0	kleiner LLOQ	#VALUE!	101	#VALUE!
0	T10	A05	0	0	kleiner LLOQ	#VALUE!	102	#VALUE!
0	T10	A06	0	0	kleiner LLOQ	#VALUE!	103	#VALUE!
0	T10	A07	0	0	kleiner LLOQ	#VALUE!	104	#VALUE!
0	T10	A08	0	0	kleiner LLOQ	#VALUE!	105	#VALUE!
0	T10	A09	0	0	kleiner LLOQ	#VALUE!	106	#VALUE!
0	T10	A10	0	0	kleiner LLOQ	#VALUE!	107	#VALUE!
0	T10	A11	0	0	kleiner LLOQ	#VALUE!	108	#VALUE!
0	T10	A12	0	0	kleiner LLOQ	#VALUE!	109	#VALUE!
0	T10	B01	0	0	kleiner LLOQ	#VALUE!	110	#VALUE!
0	T10	B02	0	0	kleiner LLOQ	#VALUE!	111	#VALUE!
0	T10	B03	0	0	kleiner LLOQ	#VALUE!	112	#VALUE!
0	T10	B04	0	0	kleiner LLOQ	#VALUE!	113	#VALUE!
0	T10	B05	0	0	kleiner LLOQ	#VALUE!	114	#VALUE!
0	T10	B06	0	0	kleiner LLOQ	#VALUE!	115	#VALUE!
0	T10	B07	0	0	kleiner LLOQ	#VALUE!	116	#VALUE!
0	T10	B08	0	0	kleiner LLOQ	#VALUE!	117	#VALUE!
0	T10	B09	0	0	kleiner LLOQ	#VALUE!	118	#VALUE!
0	T10	B10	0	0	kleiner LLOQ	#VALUE!	119	#VALUE!
0	T10	B11	0	0	kleiner LLOQ	#VALUE!	120	#VALUE!
0	T10	B12	0	0	kleiner LLOQ	#VALUE!	121	#VALUE!
0	T10	C01	0	0	kleiner LLOQ	#VALUE!	122	#VALUE!
0	T10	C02	0	0	kleiner LLOQ	#VALUE!	123	#VALUE!
0	T10	C03	0	0	kleiner LLOQ	#VALUE!	124	#VALUE!

Plate: T10

	1	2	3	4	5	6	7	8	9	10	11	12
A												
B												
C												
D												
E												
F												
G												
H												

Plattenbelegung

	1	2	3	4	5	6	7	8	9	10	11	12
A												
B												
C												
D							T10SN2	T10SN2	T10SN3	T10SN3	T10SN4	T10SN4
E					T10SS11	T10SS11	T10SS12	T10SS12			T10SS14	T10SS14
F					T10SS21	T10SS21	T10SS22	T10SS22	T10SS23	T10SS23	T10SS24	T10SS24
G							Blank PEG	Blank PEG	Blank PEG	Blank PEG	Blank PEG	Blank PEG

0	T10	C04	0	0	kleiner LLOQ	#VALUE!	125	#VALUE!
0	T10	C05	0	0	kleiner LLOQ	#VALUE!	126	#VALUE!
0	T10	C06	0	0	kleiner LLOQ	#VALUE!	127	#VALUE!
0	T10	C07	0	0	kleiner LLOQ	#VALUE!	128	#VALUE!
0	T10	C08	0	0	kleiner LLOQ	#VALUE!	129	#VALUE!
0	T10	C09	0	0	kleiner LLOQ	#VALUE!	130	#VALUE!
0	T10	C10	0	0	kleiner LLOQ	#VALUE!	131	#VALUE!
0	T10	C11	0	0	kleiner LLOQ	#VALUE!	132	#VALUE!
0	T10	C12	0	0	kleiner LLOQ	#VALUE!	133	#VALUE!
0	T10	D01	0	0	kleiner LLOQ	#VALUE!	134	#VALUE!
0	T10	D02	0	0	kleiner LLOQ	#VALUE!	135	#VALUE!
0	T10	D03	0	0	kleiner LLOQ	#VALUE!	136	#VALUE!
0	T10	D04	0	0	kleiner LLOQ	#VALUE!	137	#VALUE!
0	T10	D05	0	0	kleiner LLOQ	#VALUE!	138	#VALUE!
0	T10	D06	0	0	kleiner LLOQ	#VALUE!	139	#VALUE!
T10SN2	T10	D07	0	0	kleiner LLOQ	#VALUE!	140	#VALUE!
T10SN2	T10	D08	0	0	kleiner LLOQ	#VALUE!	141	#VALUE!
T10SN3	T10	D09	0	0	kleiner LLOQ	#VALUE!	142	#VALUE!
T10SN3	T10	D10	0	0	kleiner LLOQ	#VALUE!	143	#VALUE!
T10SN4	T10	D11	0	0	kleiner LLOQ	#VALUE!	144	#VALUE!
T10SN4	T10	D12	0	0	kleiner LLOQ	#VALUE!	145	#VALUE!
0	T10	E01	0	0	kleiner LLOQ	#VALUE!	146	#VALUE!
0	T10	E02	0	0	kleiner LLOQ	#VALUE!	147	#VALUE!
0	T10	E03	0	0	kleiner LLOQ	#VALUE!	148	#VALUE!
0	T10	E04	0	0	kleiner LLOQ	#VALUE!	149	#VALUE!
T10SS11	T10	E05	0	0	kleiner LLOQ	#VALUE!	150	#VALUE!
T10SS11	T10	E06	0	0	kleiner LLOQ	#VALUE!	151	#VALUE!
T10SS12	T10	E07	0	0	kleiner LLOQ	#VALUE!	152	#VALUE!
T10SS12	T10	E08	0	0	kleiner LLOQ	#VALUE!	153	#VALUE!
0	T10	E09	0	0	kleiner LLOQ	#VALUE!	154	#VALUE!
0	T10	E10	0	0	kleiner LLOQ	#VALUE!	155	#VALUE!
T10SS14	T10	E11	0	0	kleiner LLOQ	#VALUE!	156	#VALUE!
T10SS14	T10	E12	0	0	kleiner LLOQ	#VALUE!	157	#VALUE!
0	T10	F01	0	0	kleiner LLOQ	#VALUE!	158	#VALUE!
0	T10	F02	0	0	kleiner LLOQ	#VALUE!	159	#VALUE!
0	T10	F03	0	0	kleiner LLOQ	#VALUE!	160	#VALUE!
0	T10	F04	0	0	kleiner LLOQ	#VALUE!	161	#VALUE!
T10SS21	T10	F05	0	0	kleiner LLOQ	#VALUE!	162	#VALUE!
T10SS21	T10	F06	0	0	kleiner LLOQ	#VALUE!	163	#VALUE!
T10SS22	T10	F07	0	0	kleiner LLOQ	#VALUE!	164	#VALUE!
T10SS22	T10	F08	0	0	kleiner LLOQ	#VALUE!	165	#VALUE!
T10SS23	T10	F09	0	0	kleiner LLOQ	#VALUE!	166	#VALUE!
T10SS23	T10	F10	0	0	kleiner LLOQ	#VALUE!	167	#VALUE!
T10SS24	T10	F11	0	0	kleiner LLOQ	#VALUE!	168	#VALUE!
T10SS24	T10	F12	0	0	kleiner LLOQ	#VALUE!	169	#VALUE!
0	T10	G01	0	0	kleiner LLOQ	#VALUE!	170	#VALUE!
0	T10	G02	0	0	kleiner LLOQ	#VALUE!	171	#VALUE!
0	T10	G03	0	0	kleiner LLOQ	#VALUE!	172	#VALUE!
0	T10	G04	0	0	kleiner LLOQ	#VALUE!	173	#VALUE!
0	T10	G05	0	0	kleiner LLOQ	#VALUE!	174	#VALUE!
0	T10	G06	0	0	kleiner LLOQ	#VALUE!	175	#VALUE!
Blank PEG	T10	G07	0	0	kleiner LLOQ	#VALUE!	176	#VALUE!
Blank PEG	T10	G08	0	0	kleiner LLOQ	#VALUE!	177	#VALUE!
Blank PEG	T10	G09	0	0	kleiner LLOQ	#VALUE!	178	#VALUE!
Blank PEG	T10	G10	0	0	kleiner LLOQ	#VALUE!	179	#VALUE!
Blank PEG	T10	G11	0	0	kleiner LLOQ	#VALUE!	180	#VALUE!
Blank PEG	T10	G12	0	0	kleiner LLOQ	#VALUE!	181	#VALUE!
0	T10	H01	0	0	kleiner LLOQ	#VALUE!	182	#VALUE!
0	T10	H02	0	0	kleiner LLOQ	#VALUE!	183	#VALUE!
0	T10	H03	0	0	kleiner LLOQ	#VALUE!	184	#VALUE!
0	T10	H04	0	0	kleiner LLOQ	#VALUE!	185	#VALUE!
0	T10	H05	0	0	kleiner LLOQ	#VALUE!	186	#VALUE!
0	T10	H06	0	0	kleiner LLOQ	#VALUE!	187	#VALUE!
Blank no PEG	T10	H07	0	0	kleiner LLOQ	#VALUE!	188	#VALUE!
Blank no PEG	T10	H08	0	0	kleiner LLOQ	#VALUE!	189	#VALUE!
Blank no PEG	T10	H09	0	0	kleiner LLOQ	#VALUE!	190	#VALUE!
Blank no PEG	T10	H10	0	0	kleiner LLOQ	#VALUE!	191	#VALUE!
Blank no PEG	T10	H11	0	0	kleiner LLOQ	#VALUE!	192	#VALUE!
Blank no PEG	T10	H12	0	0	kleiner LLOQ	#VALUE!	193	#VALUE!

H							Blank no PEG	Blank no PEG	Blank no PEG	Blank no PEG	Blank no PEG	Blank no PEG

Comment:

alle Messwerte bei lin low

Date Operator

Date Control

0	T11	A01	0	0	kleiner LLOQ	#VALUE!	194	#VALUE!
0	T11	A02	0	0	kleiner LLOQ	#VALUE!	195	#VALUE!
0	T11	A03	0	0	kleiner LLOQ	#VALUE!	196	#VALUE!
0	T11	A04	0	0	kleiner LLOQ	#VALUE!	197	#VALUE!
0	T11	A05	0	0	kleiner LLOQ	#VALUE!	198	#VALUE!
0	T11	A06	0	0	kleiner LLOQ	#VALUE!	199	#VALUE!
0	T11	A07	0	0	kleiner LLOQ	#VALUE!	200	#VALUE!
0	T11	A08	0	0	kleiner LLOQ	#VALUE!	201	#VALUE!
0	T11	A09	0	0	kleiner LLOQ	#VALUE!	202	#VALUE!
0	T11	A10	0	0	kleiner LLOQ	#VALUE!	203	#VALUE!
0	T11	A11	0	0	kleiner LLOQ	#VALUE!	204	#VALUE!
0	T11	A12	0	0	kleiner LLOQ	#VALUE!	205	#VALUE!
0	T11	B01	0	0	kleiner LLOQ	#VALUE!	206	#VALUE!
0	T11	B02	0	0	kleiner LLOQ	#VALUE!	207	#VALUE!
0	T11	B03	0	0	kleiner LLOQ	#VALUE!	208	#VALUE!
0	T11	B04	0	0	kleiner LLOQ	#VALUE!	209	#VALUE!
0	T11	B05	0	0	kleiner LLOQ	#VALUE!	210	#VALUE!
0	T11	B06	0	0	kleiner LLOQ	#VALUE!	211	#VALUE!
0	T11	B07	0	0	kleiner LLOQ	#VALUE!	212	#VALUE!
0	T11	B08	0	0	kleiner LLOQ	#VALUE!	213	#VALUE!
0	T11	B09	0	0	kleiner LLOQ	#VALUE!	214	#VALUE!
0	T11	B10	0	0	kleiner LLOQ	#VALUE!	215	#VALUE!
0	T11	B11	0	0	kleiner LLOQ	#VALUE!	216	#VALUE!
0	T11	B12	0	0	kleiner LLOQ	#VALUE!	217	#VALUE!
0	T11	C01	0	0	kleiner LLOQ	#VALUE!	218	#VALUE!
0	T11	C02	0	0	kleiner LLOQ	#VALUE!	219	#VALUE!
0	T11	C03	0	0	kleiner LLOQ	#VALUE!	220	#VALUE!
0	T11	C04	0	0	kleiner LLOQ	#VALUE!	221	#VALUE!
0	T11	C05	0	0	kleiner LLOQ	#VALUE!	222	#VALUE!
0	T11	C06	0	0	kleiner LLOQ	#VALUE!	223	#VALUE!
0	T11	C07	0	0	kleiner LLOQ	#VALUE!	224	#VALUE!
0	T11	C08	0	0	kleiner LLOQ	#VALUE!	225	#VALUE!
0	T11	C09	0	0	kleiner LLOQ	#VALUE!	226	#VALUE!
0	T11	C10	0	0	kleiner LLOQ	#VALUE!	227	#VALUE!
0	T11	C11	0	0	kleiner LLOQ	#VALUE!	228	#VALUE!
0	T11	C12	0	0	kleiner LLOQ	#VALUE!	229	#VALUE!
0	T11	D01	0	0	kleiner LLOQ	#VALUE!	230	#VALUE!
0	T11	D02	0	0	kleiner LLOQ	#VALUE!	231	#VALUE!
0	T11	D03	0	0	kleiner LLOQ	#VALUE!	232	#VALUE!
0	T11	D04	0	0	kleiner LLOQ	#VALUE!	233	#VALUE!
0	T11	D05	0	0	kleiner LLOQ	#VALUE!	234	#VALUE!
0	T11	D06	0	0	kleiner LLOQ	#VALUE!	235	#VALUE!
T11SN2	T11	D07	0	0	kleiner LLOQ	#VALUE!	236	#VALUE!
T11SN2	T11	D08	0	0	kleiner LLOQ	#VALUE!	237	#VALUE!
T11SN3	T11	D09	0	0	kleiner LLOQ	#VALUE!	238	#VALUE!
T11SN3	T11	D10	0	0	kleiner LLOQ	#VALUE!	239	#VALUE!
T11SN4	T11	D11	0	0	kleiner LLOQ	#VALUE!	240	#VALUE!
T11SN4	T11	D12	0	0	kleiner LLOQ	#VALUE!	241	#VALUE!
0	T11	E01	0	0	kleiner LLOQ	#VALUE!	242	#VALUE!
0	T11	E02	0	0	kleiner LLOQ	#VALUE!	243	#VALUE!
0	T11	E03	0	0	kleiner LLOQ	#VALUE!	244	#VALUE!
0	T11	E04	0	0	kleiner LLOQ	#VALUE!	245	#VALUE!
T11SS11	T11	E05	0	0	kleiner LLOQ	#VALUE!	246	#VALUE!
T11SS11	T11	E06	0	0	kleiner LLOQ	#VALUE!	247	#VALUE!
T11SS12	T11	E07	0	0	kleiner LLOQ	#VALUE!	248	#VALUE!
T11SS12	T11	E08	0	0	kleiner LLOQ	#VALUE!	249	#VALUE!
T11SS13	T11	E09	0	0	kleiner LLOQ	#VALUE!	250	#VALUE!

Plate: T11

	1	2	3	4	5	6	7	8	9	10	11	12
A												
B												
C												
D												
E												
F												
G												
H												

Plattenbelegung

	1	2	3	4	5	6	7	8	9	10	11	12
A												
B												
C												
D							T11SN2	T11SN2	T11SN3	T11SN3	T11SN4	T11SN4
E					T11SS11	T11SS11	T11SS12	T11SS12	T11SS13	T11SS13	T11SS14	T11SS14
F					T11SS21	T11SS21	T11SS22	T11SS22	T11SS23	T11SS23	T11SS24	T11SS24
G							Blank PEG	Blank PEG	Blank PEG	Blank PEG	Blank PEG	Blank PEG
H							Blank no PEG	Blank no PEG	Blank no PEG	Blank no PEG	Blank no PEG	Blank no PEG

Comment:

alle Messwerte bei lin low

Date Operator

Date Control

T11SS13	T11	E10	0	0	kleiner LLOQ	#VALUE!	251	#VALUE!
T11SS14	T11	E11	0	0	kleiner LLOQ	#VALUE!	252	#VALUE!
T11SS14	T11	E12	0	0	kleiner LLOQ	#VALUE!	253	#VALUE!
0	T11	F01	0	0	kleiner LLOQ	#VALUE!	254	#VALUE!
0	T11	F02	0	0	kleiner LLOQ	#VALUE!	255	#VALUE!
0	T11	F03	0	0	kleiner LLOQ	#VALUE!	256	#VALUE!
0	T11	F04	0	0	kleiner LLOQ	#VALUE!	257	#VALUE!
T11SS21	T11	F05	0	0	kleiner LLOQ	#VALUE!	258	#VALUE!
T11SS21	T11	F06	0	0	kleiner LLOQ	#VALUE!	259	#VALUE!
T11SS22	T11	F07	0	0	kleiner LLOQ	#VALUE!	260	#VALUE!
T11SS22	T11	F08	0	0	kleiner LLOQ	#VALUE!	261	#VALUE!
T11SS23	T11	F09	0	0	kleiner LLOQ	#VALUE!	262	#VALUE!
T11SS23	T11	F10	0	0	kleiner LLOQ	#VALUE!	263	#VALUE!
T11SS24	T11	F11	0	0	kleiner LLOQ	#VALUE!	264	#VALUE!
T11SS24	T11	F12	0	0	kleiner LLOQ	#VALUE!	265	#VALUE!
0	T11	G01	0	0	kleiner LLOQ	#VALUE!	266	#VALUE!
0	T11	G02	0	0	kleiner LLOQ	#VALUE!	267	#VALUE!
0	T11	G03	0	0	kleiner LLOQ	#VALUE!	268	#VALUE!
0	T11	G04	0	0	kleiner LLOQ	#VALUE!	269	#VALUE!
0	T11	G05	0	0	kleiner LLOQ	#VALUE!	270	#VALUE!
0	T11	G06	0	0	kleiner LLOQ	#VALUE!	271	#VALUE!
Blank PEG	T11	G07	0	0	kleiner LLOQ	#VALUE!	272	#VALUE!
Blank PEG	T11	G08	0	0	kleiner LLOQ	#VALUE!	273	#VALUE!
Blank PEG	T11	G09	0	0	kleiner LLOQ	#VALUE!	274	#VALUE!
Blank PEG	T11	G10	0	0	kleiner LLOQ	#VALUE!	275	#VALUE!
Blank PEG	T11	G11	0	0	kleiner LLOQ	#VALUE!	276	#VALUE!
Blank PEG	T11	G12	0	0	kleiner LLOQ	#VALUE!	277	#VALUE!
0	T11	H01	0	0	kleiner LLOQ	#VALUE!	278	#VALUE!
0	T11	H02	0	0	kleiner LLOQ	#VALUE!	279	#VALUE!
0	T11	H03	0	0	kleiner LLOQ	#VALUE!	280	#VALUE!
0	T11	H04	0	0	kleiner LLOQ	#VALUE!	281	#VALUE!
0	T11	H05	0	0	kleiner LLOQ	#VALUE!	282	#VALUE!
0	T11	H06	0	0	kleiner LLOQ	#VALUE!	283	#VALUE!
Blank no PEG	T11	H07	0	0	kleiner LLOQ	#VALUE!	284	#VALUE!
Blank no PEG	T11	H08	0	0	kleiner LLOQ	#VALUE!	285	#VALUE!
Blank no PEG	T11	H09	0	0	kleiner LLOQ	#VALUE!	286	#VALUE!
Blank no PEG	T11	H10	0	0	kleiner LLOQ	#VALUE!	287	#VALUE!
Blank no PEG	T11	H11	0	0	kleiner LLOQ	#VALUE!	288	#VALUE!
Blank no PEG	T11	H12	0	0	kleiner LLOQ	#VALUE!	289	#VALUE!

1.447	1.739		1.170						0.667	0.440	0.043	1
1.447	1.332		1.392	0.137	0.146	0.130	0.138	0.109	0.729	0.478	0.042	
1.391	1.281		1.311	0.204	0.184	0.184	0.198	0.201	0.743	0.474	0.043	
0.961	0.835		1.405	0.336	0.347	0.375	0.387	0.300	0.824	0.483	0.044	
0.445	0.441		1.280	1.085	1.086	1.218	1.004	0.873	0.704	0.457	0.041	
0.355	0.315		1.332	1.768	1.925	1.753	1.739	1.606			0.036	
0.223	0.188	1.666	1.499	1.632		0.048	0.046	0.047	0.048	0.048	0.048	
0.118	0.106	1.556	1.622	1.573		0.047	0.054	0.051	0.051	0.052	0.051	

1.652	1.627		1.188	0.748	0.811	0.764	0.753	0.674	0.751	0.529	0.044	2
1.443	1.372		1.364	0.139	0.147	0.131	0.139	0.110	0.811	0.561	0.043	
1.377	1.281		1.284	0.206	0.189	0.184	0.200	0.202	0.821	0.560	0.044	
0.972	0.883		1.387	0.339	0.349	0.386	0.406	0.312	0.903	0.565	0.044	
0.484	0.474		1.298	1.087	1.113	1.203	1.022	0.903	0.790	0.544	0.042	
0.359	0.326		1.308	1.622	1.622	1.630	1.625	1.550			0.037	
0.228	0.199	1.527	1.373	1.462		0.048	0.046	0.048	0.049	0.049	0.049	
0.125	0.109	1.473	1.410	1.371		0.048	0.054	0.052	0.052	0.052	0.051	

1.579	1.475		1.150	1.210	1.303	1.202	1.219	1.125	0.759	0.549	0.043	3
1.357	1.303		1.283	0.140	0.148	0.131	0.139	0.111	0.820	0.575	0.043	
1.323	1.228		1.227	0.206	0.188	0.184	0.198	0.201	0.824	0.579	0.043	
0.959	0.872		1.321	0.339	0.348	0.387	0.406	0.314	0.909	0.580	0.044	
0.483	0.473		1.270	1.069	1.103	1.181	1.017	0.903	0.798	0.560	0.042	
0.360	0.326		1.270	1.497	1.448	1.496	1.494	1.449			0.037	
0.228	0.199	1.374	1.262	1.314		0.048	0.046	0.048	0.049	0.049	0.049	
0.125	0.110	1.346	1.262	1.253		0.048	0.054	0.052	0.052	0.052	0.051	

1.511	1.369			1.538	1.712	1.528	1.502	1.425			0.043	4
1.296	1.261										0.043	
1.272	1.193										0.043	
0.947	0.859										0.044	
0.481	0.471										0.042	
0.361	0.325										0.037	
0.229	0.199					0.048	0.046	0.048	0.049	0.049	0.049	
0.125	0.110					0.047	0.054	0.052	0.052	0.052	0.051	

1.493	1.317	1.678	1.110	1.621	1.807	1.618	1.584	1.508	0.765	0.597	0.043	5
1.259	1.238	1.850	1.197	0.139	0.150	0.132	0.139	0.111	0.821	0.588	0.043	
1.248	1.175	1.816	1.153	0.204	0.188	0.184	0.197	0.199	0.831	0.599	0.044	
0.943	0.859	1.970	1.231	0.338	0.357	0.386	0.406	0.316	0.916	0.595	0.044	
0.480	0.470	2.220	1.220	1.054	1.088	1.154	1.006	0.902	0.805	0.579	0.042	
0.359	0.325	2.149	1.211	1.395	1.324	1.348	1.345	1.317	0.037	0.037	0.037	
0.228	0.199	1.240	1.151	1.183	0.034	0.049	0.046	0.048	0.049	0.049	0.049	
0.125	0.110	1.223	1.132	1.146	0.038	0.048	0.054	0.052	0.052	0.052	0.051	

1.434	1.258		1.090	1.648	1.876	1.649	1.627	1.535				6
1.217	1.201		1.149									
1.205	1.143		1.109									
0.935	0.845		1.182									
0.477	0.469		1.181									
0.360	0.323		1.167									
0.229	0.200					0.049	0.047	0.048	0.049	0.049	0.049	
0.125	0.110					0.048	0.054	0.052	0.052	0.052	0.051	

1.013	0.810		0.668	0.808	0.940	0.720	0.798	0.687	0.904	0.845	0.043	7
0.709	0.827		0.703	0.142	0.156	0.136	0.137	0.116	0.990	0.846	0.042	
0.682	0.692		0.680	0.197	0.188	0.190	0.191	0.184	1.023	0.861	0.043	
0.507	0.519		0.740	0.316	0.264	0.364	0.377	0.312	1.111	0.870	0.043	
0.394	0.406		0.682	0.580	0.561	0.600	0.529	0.563	0.962	0.834	0.042	
0.333	0.322		0.661	0.995	0.930	0.848	0.832	0.797	0.037	0.037	0.037	
0.229	0.201	0.843	0.679	0.732		0.048	0.046	0.047			0.047	
0.124	0.111	0.773	0.682	0.701		0.047	0.053	0.051	0.051	0.052	0.051	

skin cultivation

0.685	0.649	0.387	0.342									T8 1 hum sui
0.154	0.141	0.128	0.091									
0.558	0.521	0.531	0.224									
	0.277	0.599	0.442									
0.305	0.254	0.135	0.201									
0.266	0.234	0.279	0.262									
						0.051	0.054	0.050	0.052	0.051	0.051	
						0.051	0.051	0.050	0.050	0.051	0.050	

				0.635	0.607	0.628	0.661	0.404	0.309	0.229	0.222	T8 2 hum sui
				0.460	0.171	0.156	0.167	0.129	0.136	0.081	0.084	
				0.458	0.708	0.553	0.584	0.670	0.659	0.222	0.224	
						0.262	0.241	0.621	0.621	0.449	0.458	
				0.260	0.234	0.197	0.193	0.128	0.132	0.204	0.210	
				0.245	0.247	0.151	0.146	0.271	0.265	0.268	0.253	
						0.053	0.055	0.053	0.054	0.053	0.053	
						0.053	0.052	0.051	0.052	0.052	0.052	

0.488	0.481	0.313	0.358									T9 1 hum sui
0.137	0.134	0.176	0.097									
0.473	0.445	0.459	0.179									
	0.323	0.536	0.588									
0.374	0.244	0.122	0.203									
0.333	0.253	0.304	0.282									
						0.049	0.049	0.049	0.050	0.049	0.049	
						0.050	0.048	0.048	0.048	0.047	0.048	

				0.379	0.370	0.484	0.478	0.220	0.291	0.404	0.398	T9 2 hum sui
				0.157	0.155	0.155	0.157	0.194	0.200	0.080	0.091	
				0.623	0.633	0.542	0.548	0.625	0.640	0.197	0.192	
						0.331	0.343	0.621	0.661	0.691	0.644	
				0.415	0.405	0.204	0.207			0.233	0.231	
				0.295	0.327	0.199	0.193	0.277	0.307	0.271	0.291	
						0.051	0.051	0.051	0.051	0.051	0.050	
						0.050	0.049	0.050	0.049	0.050	0.050	

												T9 hum sui 2 wells vergesse
								0.138	0.133			
						0.051	0.051	0.051	0.051	0.051	0.050	
						0.050	0.049	0.050	0.050	0.050	0.050	

0.349	0.421	0.408	0.372									T10 1 hum sui
0.125	0.140	0.183	0.089									
0.538	0.449	0.477	0.178									
	0.486	0.476	0.666									
0.367	0.243		0.213									
0.431	0.273	0.263	0.314									
						0.049	0.049	0.049	0.051	0.049	0.052	
						0.049	0.048	0.052	0.050	0.049	0.049	

				0.337	0.335	0.457	0.454	0.421	0.439	0.367	0.373	T10 2 hum sui
				0.137	0.138	0.156	0.166	0.178	0.182	0.087	0.082	
				0.645	0.646	0.519	0.550	0.557	0.572	0.193	0.182	
						0.493	0.502	0.540	0.537	0.680	0.722	
				0.377	0.373	0.217	0.208			0.232	0.233	
				0.367	0.392	0.213	0.195	0.226	0.257	0.308	0.408	

						0.051	0.051	0.051	0.053	0.051	0.054
						0.050	0.050	0.052	0.051	0.050	0.050

0.491	0.574	0.518	0.380									T11 1 hum sui
0.074	0.073	0.106	0.089									
0.689	0.516	0.535	0.209									
	0.352	0.464	0.727									
0.433	0.269	0.165	0.224									
0.402	0.254	0.296	0.329									
						0.052	0.051	0.051	0.053	0.051	0.051	
						0.049	0.049	0.050	0.050	0.049	0.049	

				0.502	0.545	0.613	0.593	0.579	0.537	0.385	0.388	T11 2 hum sui
				0.206	0.205	0.187	0.189	0.187	0.177	0.122	0.114	
				0.731	0.737	0.575	0.570	0.538	0.531	0.208	0.207	
						0.358	0.336	0.471	0.480	0.747	0.716	
				0.423	0.402	0.227	0.267	0.154	0.156	0.234	0.235	
				0.354	0.398	0.217	0.229	0.248	0.272	0.308	0.317	
						0.052	0.052	0.052	0.056	0.052	0.052	
						0.050	0.050	0.050	0.050	0.050	0.049	

Project number		F-120	Apparatus	Wallac Victor	Operator	IsBa
GLP Study (Number)		n.a.	Protocol (Instrument method)	LDH test 2016	Date of preparation	19-04-18
hot substance	isotope	n.a.	File name (results)	IsBa_180419/20_LDH_full_va	Date of measurement	19-04-18
	name	n.a.	Kind of well plate	96 well	shaking time [min]	30
	ACB-ID	n.a.	sample volume [µL]	100	stirring rate (Target) [rpm]	150
	Batch number	n.a.	Cocktail volume [µL]	175	Kind of measurement	UV-vis
cold substance	name	LDH	ACB-ID of cocktail		Wave length [nm]	450
	ACB-ID	L23982R	Matrix	DMEM (from powder)+PEG	Remarks	Cocktail 100µl RM, 75µl STOP
	Batch number	SLBK6345V	Blank description	DMEM/PEG, H2O	Remarks	7 standards split low/high
n.a.		n.a.	Pipettes (No. / volume)	50-200µl	Remarks	KLP4 common for both
n.a.		n.a.	Pipettes (No. / volume)	n.a.	Remarks	n.a.

Messdaten (diese Tabelle in Bericht übernehmen)

Sample name *	concentration (theor.) *	measured data	measured data	measured data	mean measured	SD	RSD	Blank *	measured data after * Blank subtraction	concentration (calc.) *	Deviation *	Residuen
	[µg/mL]	[AU]	[AU]	[AU]	[AU]	[AU]	[%]	[AU]		[µg/mL]	[%]	
KLP1								0.047				
KLP2								0.054				
KLP3								0.051				
KLP4	0.041	0.959	0.972	0.883	0.938	0.04	4.19	0.051	0.887	0.041	-0.57	0.00
KLP5	0.018	0.483	0.484	0.474	0.480	0.00	0.98	0.052	0.429	0.019	2.42	0.00
KLP6	0.012	0.360	0.359	0.326	0.348	0.02	4.54	0.051	0.297	0.013	5.00	0.00
KLP7	0.007	0.228	0.228	0.199	0.218	0.014	6.35		0.168	0.006	-11.29	0.00
KLP8												

Statistical data

Geradensteigung	Slope	m	21.03
Y-Achsenabschnitt	Y-Intercept	b	0.03
Standardabw. Geradensteigung	SD-Slope	s_m	0.648055501
Standardabw. Achsenabschnittes	SD-Y-Intercept	s_b	0.015177452
Anzahl Messpunkte	number of measuring points	n	4
Quadratsumme	sum of squares	Qxx	0.000664048
Bereichsmitte			0.019557193
Freiheitsgrade	degree of freedom	f	2
Student-t-Faktor für (P = 95 %; f = n-2)	Student-t-factor for (P = 95 %; f = n-2)	t	4.303
Vertrauensbereich Steig. (95 %) Obergrenze		$m + VB_m$	23.81763508
Vertrauensbereich Steig. (95 %) Untergrenze		$m - VB_m$	18.24046944
Vertrauensbereich Achsenabschnitt (95 %) Og.		$b + Vb_b$	0.099335829
Vertrauensbereich Achsenabschnitt (95 %) Ug.		$b - Vb_b$	-0.031281327
Korrelationskoeffizient	correlation coefficient	r	0.9991
Bestimmtheitsmaß	determiantion coefficient	r^2	0.9981
Reststandardabweichung		s_0	0.01669983
Summe Restquadrate		sd	0.657385838
Verfahrensstandardabw.		s_{x0}	0.000794131
Rel. Verfahrensstandardabw. %		V_{x0}	4.060559029

Berichten

mean Blank	**0**	
SD Blank	0.00	
RSD Blank	3.79	x=
x*SD (LLOQ)	0.01	5
x*SD (LOD)	0.01	3
**LLOQ (AU)**	**0.061**	
**LOD (AU)**	**0.057**	
ULOQ	0.887	
LLOQ (Lin)	0.168	

Evaluation / Comment

LDH linearity valid with 7 standards, split in lin high and lin low, each with 4 standards and KLP4 common standard for both. R² 0,9981, deviations for both between -13.34% and +13.04%

Date Operator Date Control

Figure 1 Linearity

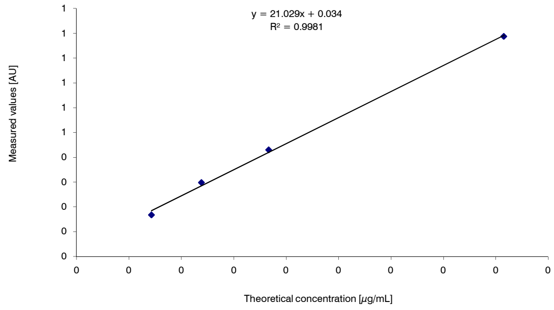

Figure 2 Method validation Residuen Plot

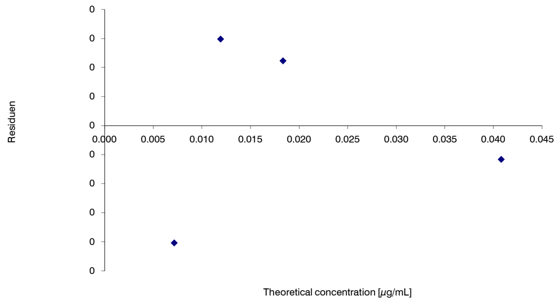

Evaluation / Comment

lin passed

Date	Operator	Date	Control

Sample name	Plate	Position	[AU]	[AU]-Blank	Rating	[µg/mL]	Dilution factor	[µg/mL]
0	T12	A01	0	0	kleiner LLOQ	#VALUE!	1	#VALUE!
0	T12	A02	0	0	kleiner LLOQ	#VALUE!	1	#VALUE!
0	T12	A03	0	0	kleiner LLOQ	#VALUE!	1	#VALUE!
0	T12	A04	0	0	kleiner LLOQ	#VALUE!	1	#VALUE!
0	T12	A05	0	0	kleiner LLOQ	#VALUE!	1	#VALUE!
0	T12	A06	0	0	kleiner LLOQ	#VALUE!	1	#VALUE!
0	T12	A07	0	0	kleiner LLOQ	#VALUE!	1	#VALUE!
0	T12	A08	0	0	kleiner LLOQ	#VALUE!	1	#VALUE!
0	T12	A09	0	0	kleiner LLOQ	#VALUE!	1	#VALUE!
0	T12	A10	0	0	kleiner LLOQ	#VALUE!	1	#VALUE!
0	T12	A11	0	0	kleiner LLOQ	#VALUE!	1	#VALUE!
0	T12	A12	0	0	kleiner LLOQ	#VALUE!	1	#VALUE!
0	T12	B01	0	0	kleiner LLOQ	#VALUE!	1	#VALUE!
0	T12	B02	0	0	kleiner LLOQ	#VALUE!	1	#VALUE!
0	T12	B03	0	0	kleiner LLOQ	#VALUE!	1	#VALUE!
0	T12	B04	0	0	kleiner LLOQ	#VALUE!	1	#VALUE!
0	T12	B05	0	0	kleiner LLOQ	#VALUE!	1	#VALUE!
0	T12	B06	0	0	kleiner LLOQ	#VALUE!	1	#VALUE!
0	T12	B07	0	0	kleiner LLOQ	#VALUE!	1	#VALUE!
0	T12	B08	0	0	kleiner LLOQ	#VALUE!	1	#VALUE!
0	T12	B09	0	0	kleiner LLOQ	#VALUE!	1	#VALUE!
0	T12	B10	0	0	kleiner LLOQ	#VALUE!	1	#VALUE!
0	T12	B11	0	0	kleiner LLOQ	#VALUE!	1	#VALUE!
0	T12	B12	0	0	kleiner LLOQ	#VALUE!	1	#VALUE!
0	T12	C01	0	0	kleiner LLOQ	#VALUE!	1	#VALUE!
0	T12	C02	0	0	kleiner LLOQ	#VALUE!	1	#VALUE!
0	T12	C03	0	0	kleiner LLOQ	#VALUE!	1	#VALUE!
0	T12	C04	0	0	kleiner LLOQ	#VALUE!	1	#VALUE!
0	T12	C05	0	0	kleiner LLOQ	#VALUE!	1	#VALUE!
0	T12	C06	0	0	kleiner LLOQ	#VALUE!	1	#VALUE!
0	T12	C07	0	0	kleiner LLOQ	#VALUE!	1	#VALUE!
0	T12	C08	0	0	kleiner LLOQ	#VALUE!	1	#VALUE!
0	T12	C09	0	0	kleiner LLOQ	#VALUE!	1	#VALUE!
0	T12	C10	0	0	kleiner LLOQ	#VALUE!	1	#VALUE!
0	T12	C11	0	0	kleiner LLOQ	#VALUE!	1	#VALUE!
0	T12	C12	0	0	kleiner LLOQ	#VALUE!	1	#VALUE!
0	T12	D01	0	0	kleiner LLOQ	#VALUE!	1	#VALUE!
0	T12	D02	0	0	kleiner LLOQ	#VALUE!	1	#VALUE!
0	T12	D03	0	0	kleiner LLOQ	#VALUE!	1	#VALUE!
0	T12	D04	0	0	kleiner LLOQ	#VALUE!	1	#VALUE!
0	T12	D05	0	0	kleiner LLOQ	#VALUE!	1	#VALUE!
0	T12	D06	0	0	kleiner LLOQ	#VALUE!	1	#VALUE!
T12SN2	T12	D07	0.392	0.341	0.341	0.015	2.75	0.040
T12SN2	T12	D08	0.404	0.353	0.353	0.015	2.75	0.042
T12SN3	T12	D09	0.444	0.393	0.393	0.017	2.75	0.047
T12SN3	T12	D10	0.467	0.416	0.416	0.018	2.75	0.050
T12SN4	T12	D11	0.574	0.523	0.523	0.023	2.75	0.064
T12SN4	T12	D12	0.587	0.536	0.536	0.024	2.75	0.066

Plate:	T12

	Test	Test	Test	Test								
	1	2	3	4	5	6	7	8	9	10	11	12
A												
B												
C												
D							0.392	0.404	0.444	0.467	0.574	0.587
E					0.636	0.622	0.342	0.371	0.439	0.463	0.228	0.226
F					0.481	0.487	0.294	0.226	0.298	0.299	0.306	0.308
G							0.049	0.049	0.050	0.049	0.049	0.049
H							0.047	0.047	0.047	0.048	0.048	0.047

Plattenbelegung

	1	2	3	4	5	6	7	8	9	10	11	12
A												
B												
C												
D							T12SN2	T12SN2	T12SN3	T12SN3	T12SN4	T12SN4
E					T12SS11	T12SS11	T12SS12	T12SS12	T12SS13	T12SS13	T12SS14	T12SS14
F					T12SS21	T12SS21	T12SS22	T12SS22	T12SS23	T12SS23	T12SS24	T12SS24
G							Blank PEG	Blank PEG	Blank PEG	Blank PEG	Blank PEG	Blank PEG
H							Blank no PEG	Blank no PEG	Blank no PEG	Blank no PEG	Blank no PEG	Blank no PEG

Samples aufgeteilt in lin high und low:höher konzentrierte Samples bei lin high zu finden.

Comment:

Erste 4 Reihen immer Testmessung zur Evaluierung der benötigten Verdünnung. Wird nicht mit ausgewertet. Unverdünnte Messungen außerhalb der Linearität werden ebenfalls nicht ausgewertet. Dil.Faktor unverdünnte samples=2,75 (100µl sample+100µlRM+75µl stop sol.) Verdünnung (gelb) Dil.8 Dil8*Dil2,75=Dil.22

Date Operator

Date Control

0	T12	E01	0	0	kleiner LLOQ	#VALUE!	1	#VALUE!
0	T12	E02	0	0	kleiner LLOQ	#VALUE!	1	#VALUE!
0	T12	E03	0	0	kleiner LLOQ	#VALUE!	1	#VALUE!
0	T12	E04	0	0	kleiner LLOQ	#VALUE!	1	#VALUE!
T12SS11	T12	E05	0.636	0.585	0.585	0.026	2.75	0.072
T12SS11	T12	E06	0.622	0.571	0.571	0.026	2.75	0.070
T12SS12	T12	E07	0.342	0.291	0.291	0.012	2.75	0.034
T12SS12	T12	E08	0.371	0.320	0.320	0.014	2.75	0.037
T12SS13	T12	E09	0.439	0.388	0.388	0.017	2.75	0.046
T12SS13	T12	E10	0.463	0.412	0.412	0.018	2.75	0.049
T12SS14	T12	E11	0.228	0.177	0.177	0.007	2.75	0.019
T12SS14	T12	E12	0.226	0.175	0.175	0.007	2.75	0.018
0	T12	F01	0	0	kleiner LLOQ	#VALUE!	1	#VALUE!
0	T12	F02	0	0	kleiner LLOQ	#VALUE!	1	#VALUE!
0	T12	F03	0	0	kleiner LLOQ	#VALUE!	1	#VALUE!
0	T12	F04	0	0	kleiner LLOQ	#VALUE!	1	#VALUE!
T12SS21	T12	F05	0.481	0.430	0.430	0.019	2.75	0.052
T12SS21	T12	F06	0.487	0.436	0.436	0.019	2.75	0.053
T12SS22	T12	F07	0.294	0.244	0.244	0.010	2.75	0.027
T12SS22	T12	F08	0.226	0.175	0.175	0.007	2.75	0.018
T12SS23	T12	F09	0.298	0.247	0.247	0.010	2.75	0.028
T12SS23	T12	F10	0.299	0.248	0.248	0.010	2.75	0.028
T12SS24	T12	F11	0.306	0.255	0.255	0.011	2.75	0.029
T12SS24	T12	F12	0.308	0.257	0.257	0.011	2.75	0.029
0	T12	G01	0	0	kleiner LLOQ	#VALUE!	1	#VALUE!
0	T12	G02	0	0	kleiner LLOQ	#VALUE!	1	#VALUE!
0	T12	G03	0	0	kleiner LLOQ	#VALUE!	1	#VALUE!
0	T12	G04	0	0	kleiner LLOQ	#VALUE!	1	#VALUE!
0	T12	G05	0	0	kleiner LLOQ	#VALUE!	1	#VALUE!
0	T12	G06	0	0	kleiner LLOQ	#VALUE!	1	#VALUE!
Blank PEG	T12	G07	0	0	kleiner LLOQ	#VALUE!	2.75	#VALUE!
Blank PEG	T12	G08	0	0	kleiner LLOQ	#VALUE!	2.75	#VALUE!
Blank PEG	T12	G09	0	0	kleiner LLOQ	#VALUE!	2.75	#VALUE!
Blank PEG	T12	G10	0	0	kleiner LLOQ	#VALUE!	2.75	#VALUE!
Blank PEG	T12	G11	0	0	kleiner LLOQ	#VALUE!	2.75	#VALUE!
Blank PEG	T12	G12	0	0	kleiner LLOQ	#VALUE!	2.75	#VALUE!
0	T12	H01	0	0	kleiner LLOQ	#VALUE!	1	#VALUE!
0	T12	H02	0	0	kleiner LLOQ	#VALUE!	1	#VALUE!
0	T12	H03	0	0	kleiner LLOQ	#VALUE!	1	#VALUE!
0	T12	H04	0	0	kleiner LLOQ	#VALUE!	1	#VALUE!
0	T12	H05	0	0	kleiner LLOQ	#VALUE!	1	#VALUE!
0	T12	H06	0	0	kleiner LLOQ	#VALUE!	1	#VALUE!
Blank no PEG	T12	H07	0	0	kleiner LLOQ	#VALUE!	2.75	#VALUE!
Blank no PEG	T12	H08	0	0	kleiner LLOQ	#VALUE!	2.75	#VALUE!
Blank no PEG	T12	H09	0	0	kleiner LLOQ	#VALUE!	2.75	#VALUE!
Blank no PEG	T12	H10	0	0	kleiner LLOQ	#VALUE!	2.75	#VALUE!
Blank no PEG	T12	H11	0	0	kleiner LLOQ	#VALUE!	2.75	#VALUE!
Blank no PEG	T12	H12	0	0	kleiner LLOQ	#VALUE!	2.75	#VALUE!

0	T13	A01	0	0	kleiner LLOQ	#VALUE!	1	#VALUE!
0	T13	A02	0	0	kleiner LLOQ	#VALUE!	1	#VALUE!
0	T13	A03	0	0	kleiner LLOQ	#VALUE!	1	#VALUE!
0	T13	A04	0	0	kleiner LLOQ	#VALUE!	1	#VALUE!
0	T13	A05	0	0	kleiner LLOQ	#VALUE!	1	#VALUE!
0	T13	A06	0	0	kleiner LLOQ	#VALUE!	1	#VALUE!
0	T13	A07	0	0	kleiner LLOQ	#VALUE!	1	#VALUE!
0	T13	A08	0	0	kleiner LLOQ	#VALUE!	1	#VALUE!
0	T13	A09	0	0	kleiner LLOQ	#VALUE!	1	#VALUE!
0	T13	A10	0	0	kleiner LLOQ	#VALUE!	1	#VALUE!
0	T13	A11	0	0	kleiner LLOQ	#VALUE!	1	#VALUE!
0	T13	A12	0	0	kleiner LLOQ	#VALUE!	1	#VALUE!
0	T13	B01	0	0	kleiner LLOQ	#VALUE!	1	#VALUE!
0	T13	B02	0	0	kleiner LLOQ	#VALUE!	1	#VALUE!
0	T13	B03	0	0	kleiner LLOQ	#VALUE!	1	#VALUE!
0	T13	B04	0	0	kleiner LLOQ	#VALUE!	1	#VALUE!
0	T13	B05	0	0	kleiner LLOQ	#VALUE!	1	#VALUE!
0	T13	B06	0	0	kleiner LLOQ	#VALUE!	1	#VALUE!
0	T13	B07	0	0	kleiner LLOQ	#VALUE!	1	#VALUE!
0	T13	B08	0	0	kleiner LLOQ	#VALUE!	1	#VALUE!
0	T13	B09	0	0	kleiner LLOQ	#VALUE!	1	#VALUE!
0	T13	B10	0	0	kleiner LLOQ	#VALUE!	1	#VALUE!
0	T13	B11	0	0	kleiner LLOQ	#VALUE!	1	#VALUE!
0	T13	B12	0	0	kleiner LLOQ	#VALUE!	1	#VALUE!
0	T13	C01	0	0	kleiner LLOQ	#VALUE!	1	#VALUE!
0	T13	C02	0	0	kleiner LLOQ	#VALUE!	1	#VALUE!
0	T13	C03	0	0	kleiner LLOQ	#VALUE!	1	#VALUE!
0	T13	C04	0	0	kleiner LLOQ	#VALUE!	1	#VALUE!
0	T13	C05	0	0	kleiner LLOQ	#VALUE!	1	#VALUE!
0	T13	C06	0	0	kleiner LLOQ	#VALUE!	1	#VALUE!
0	T13	C07	0	0	kleiner LLOQ	#VALUE!	1	#VALUE!
0	T13	C08	0	0	kleiner LLOQ	#VALUE!	1	#VALUE!
0	T13	C09	0	0	kleiner LLOQ	#VALUE!	1	#VALUE!
0	T13	C10	0	0	kleiner LLOQ	#VALUE!	1	#VALUE!
0	T13	C11	0	0	kleiner LLOQ	#VALUE!	1	#VALUE!
0	T13	C12	0	0	kleiner LLOQ	#VALUE!	1	#VALUE!
0	T13	D01	0	0	kleiner LLOQ	#VALUE!	1	#VALUE!
0	T13	D02	0	0	kleiner LLOQ	#VALUE!	1	#VALUE!
0	T13	D03	0	0	kleiner LLOQ	#VALUE!	1	#VALUE!
0	T13	D04	0	0	kleiner LLOQ	#VALUE!	1	#VALUE!
0	T13	D05	0	0	kleiner LLOQ	#VALUE!	1	#VALUE!
0	T13	D06	0	0	kleiner LLOQ	#VALUE!	1	#VALUE!
T13SN2	T13	D07	0.554	0.503	0.503	0.022	2.75	0.061
T13SN2	T13	D08	0.540	0.489	0.489	0.022	2.75	0.059
T13SN3	T13	D09	0.464	0.413	0.413	0.018	2.75	0.050
T13SN3	T13	D10	0.471	0.420	0.420	0.018	2.75	0.051
T13SN4	T13	D11	0.680	0.629	0.629	0.028	2.75	0.078
T13SN4	T13	D12	0.708	0.657	0.657	0.030	2.75	0.081

Plate: T13

	1	2	3	4	5	6	7	8	9	10	11	12
A												
B												
C												
D							0.554	0.540	0.464	0.471	0.680	0.708
E					0.472	0.430	0.233	0.247	0.137	0.142	0.228	0.235
F					0.320	0.258	0.191	0.220	0.250	0.250	0.294	0.305
G							0.050	0.050	0.053	0.051	0.051	0.050
H							0.048	0.048	0.048	0.048	0.048	0.048

Plattenbelegung

	1	2	3	4	5	6	7	8	9	10	11	12
A												
B												
C												
D							T13SN2	T13SN2	T13SN3	T13SN3	T13SN4	T13SN4
E					T13SS11	T13SS11	T13SS12	T13SS12	T13SS13	T13SS13	T13SS14	T13SS14
F					T13SS21	T13SS21	T13SS22	T13SS22	T13SS23	T13SS23	T13SS24	T13SS24
G							Blank PEG	Blank PEG	Blank PEG	Blank PEG	Blank PEG	Blank PEG
H							Blank no PEG	Blank no PEG	Blank no PEG	Blank no PEG	Blank no PEG	Blank no PEG

Comment:

Date Operator

Date Control

0	T13	E01	0	0	kleiner LLOQ	#VALUE!	1	#VALUE!
0	T13	E02	0	0	kleiner LLOQ	#VALUE!	1	#VALUE!
0	T13	E03	0	0	kleiner LLOQ	#VALUE!	1	#VALUE!
0	T13	E04	0	0	kleiner LLOQ	#VALUE!	1	#VALUE!
T13SS11	T13	E05	0.472	0.421	0.421	0.018	2.75	0.051
T13SS11	T13	E06	0.430	0.379	0.379	0.016	2.75	0.045
T13SS12	T13	E07	0.233	0.182	0.182	0.007	2.75	0.019
T13SS12	T13	E08	0.247	0.196	0.196	0.008	2.75	0.021
T13SS13	T13	E09	0.137	0.086	kleiner LLOQ	#VALUE!	2.75	#VALUE!
T13SS13	T13	E10	0.142	0.091	kleiner LLOQ	#VALUE!	2.75	#VALUE!
T13SS14	T13	E11	0.228	0.177	0.177	0.007	2.75	0.019
T13SS14	T13	E12	0.235	0.184	0.184	0.007	2.75	0.020
0	T13	F01	0	0	kleiner LLOQ	#VALUE!	1	#VALUE!
0	T13	F02	0	0	kleiner LLOQ	#VALUE!	1	#VALUE!
0	T13	F03	0	0	kleiner LLOQ	#VALUE!	1	#VALUE!
0	T13	F04	0	0	kleiner LLOQ	#VALUE!	1	#VALUE!
T13SS21	T13	F05	0.320	0.269	0.269	0.011	2.75	0.031
T13SS21	T13	F06	0.258	0.208	0.208	0.008	2.75	0.023
T13SS22	T13	F07	0.191	0.140	kleiner LLOQ	#VALUE!	2.75	#VALUE!
T13SS22	T13	F08	0.220	0.169	0.169	0.006	2.75	0.018
T13SS23	T13	F09	0.250	0.199	0.199	0.008	2.75	0.022
T13SS23	T13	F10	0.250	0.199	0.199	0.008	2.75	0.022
T13SS24	T13	F11	0.294	0.243	0.243	0.010	2.75	0.027
T13SS24	T13	F12	0.305	0.254	0.254	0.010	2.75	0.029
0	T13	G01	0	0	kleiner LLOQ	#VALUE!	1	#VALUE!
0	T13	G02	0	0	kleiner LLOQ	#VALUE!	1	#VALUE!
0	T13	G03	0	0	kleiner LLOQ	#VALUE!	1	#VALUE!
0	T13	G04	0	0	kleiner LLOQ	#VALUE!	1	#VALUE!
0	T13	G05	0	0	kleiner LLOQ	#VALUE!	1	#VALUE!
0	T13	G06	0	0	kleiner LLOQ	#VALUE!	1	#VALUE!
Blank PEG	T13	G07	0	0	kleiner LLOQ	#VALUE!	2.75	#VALUE!
Blank PEG	T13	G08	0	0	kleiner LLOQ	#VALUE!	2.75	#VALUE!
Blank PEG	T13	G09	0	0	kleiner LLOQ	#VALUE!	2.75	#VALUE!
Blank PEG	T13	G10	0	0	kleiner LLOQ	#VALUE!	2.75	#VALUE!
Blank PEG	T13	G11	0	0	kleiner LLOQ	#VALUE!	2.75	#VALUE!
Blank PEG	T13	G12	0	0	kleiner LLOQ	#VALUE!	2.75	#VALUE!
0	T13	H01	0	0	kleiner LLOQ	#VALUE!	1	#VALUE!
0	T13	H02	0	0	kleiner LLOQ	#VALUE!	1	#VALUE!
0	T13	H03	0	0	kleiner LLOQ	#VALUE!	1	#VALUE!
0	T13	H04	0	0	kleiner LLOQ	#VALUE!	1	#VALUE!
0	T13	H05	0	0	kleiner LLOQ	#VALUE!	1	#VALUE!
0	T13	H06	0	0	kleiner LLOQ	#VALUE!	1	#VALUE!
Blank no PEG	T13	H07	0	0	kleiner LLOQ	#VALUE!	2.75	#VALUE!
Blank no PEG	T13	H08	0	0	kleiner LLOQ	#VALUE!	2.75	#VALUE!
Blank no PEG	T13	H09	0	0	kleiner LLOQ	#VALUE!	2.75	#VALUE!
Blank no PEG	T13	H10	0	0	kleiner LLOQ	#VALUE!	2.75	#VALUE!
Blank no PEG	T13	H11	0	0	kleiner LLOQ	#VALUE!	2.75	#VALUE!
Blank no PEG	T13	H12	0	0	kleiner LLOQ	#VALUE!	2.75	#VALUE!
0	T14	A01	0	0	kleiner LLOQ	#VALUE!	1	#VALUE!
0	T14	A02	0	0	kleiner LLOQ	#VALUE!	1	#VALUE!
0	T14	A03	0	0	kleiner LLOQ	#VALUE!	1	#VALUE!
0	T14	A04	0	0	kleiner LLOQ	#VALUE!	1	#VALUE!
0	T14	A05	0	0	kleiner LLOQ	#VALUE!	1	#VALUE!
0	T14	A06	0	0	kleiner LLOQ	#VALUE!	1	#VALUE!
0	T14	A07	0	0	kleiner LLOQ	#VALUE!	1	#VALUE!
0	T14	A08	0	0	kleiner LLOQ	#VALUE!	1	#VALUE!
0	T14	A09	0	0	kleiner LLOQ	#VALUE!	1	#VALUE!
0	T14	A10	0	0	kleiner LLOQ	#VALUE!	1	#VALUE!
0	T14	A11	0	0	kleiner LLOQ	#VALUE!	1	#VALUE!
0	T14	A12	0	0	kleiner LLOQ	#VALUE!	1	#VALUE!
0	T14	B01	0	0	kleiner LLOQ	#VALUE!	1	#VALUE!
0	T14	B02	0	0	kleiner LLOQ	#VALUE!	1	#VALUE!
0	T14	B03	0	0	kleiner LLOQ	#VALUE!	1	#VALUE!
0	T14	B04	0	0	kleiner LLOQ	#VALUE!	1	#VALUE!
0	T14	B05	0	0	kleiner LLOQ	#VALUE!	1	#VALUE!
0	T14	B06	0	0	kleiner LLOQ	#VALUE!	1	#VALUE!
0	T14	B07	0	0	kleiner LLOQ	#VALUE!	1	#VALUE!
0	T14	B08	0	0	kleiner LLOQ	#VALUE!	1	#VALUE!
0	T14	B09	0	0	kleiner LLOQ	#VALUE!	1	#VALUE!
0	T14	B10	0	0	kleiner LLOQ	#VALUE!	1	#VALUE!
0	T14	B11	0	0	kleiner LLOQ	#VALUE!	1	#VALUE!
0	T14	B12	0	0	kleiner LLOQ	#VALUE!	1	#VALUE!
0	T14	C01	0	0	kleiner LLOQ	#VALUE!	1	#VALUE!

Plate:	T14

	1	2	3	4	5	6	7	8	9	10	11	12
A												
B												
C												
D							0.467	0.518	0.607	0.573	0.510	0.574
E					0.448	0.454	0.290	0.288	0.177	0.183	0.311	0.279
F					0.471	0.511	0.236	0.261	0.259	0.349	0.350	0.224
G							0.041	0.048	0.048	0.048	0.048	0.047
H							0.044	0.045	0.046	0.048	0.046	0.048

Plattenbelegung

	1	2	3	4	5	6	7	8	9	10	11	12
A												
B												
C												
D							T14SN2	T14SN2	T14SN3	T14SN3	T14SN4	T14SN4
E					T14SS11	T14SS11	T14SS12	T14SS12	T14SS13	T14SS13	T14SS14	T14SS14

0	T14	C02	0	0	kleiner LLOQ	#VALUE!	1	#VALUE!
0	T14	C03	0	0	kleiner LLOQ	#VALUE!	1	#VALUE!
0	T14	C04	0	0	kleiner LLOQ	#VALUE!	1	#VALUE!
0	T14	C05	0	0	kleiner LLOQ	#VALUE!	1	#VALUE!
0	T14	C06	0	0	kleiner LLOQ	#VALUE!	1	#VALUE!
0	T14	C07	0	0	kleiner LLOQ	#VALUE!	1	#VALUE!
0	T14	C08	0	0	kleiner LLOQ	#VALUE!	1	#VALUE!
0	T14	C09	0	0	kleiner LLOQ	#VALUE!	1	#VALUE!
0	T14	C10	0	0	kleiner LLOQ	#VALUE!	1	#VALUE!
0	T14	C11	0	0	kleiner LLOQ	#VALUE!	1	#VALUE!
0	T14	C12	0	0	kleiner LLOQ	#VALUE!	1	#VALUE!
0	T14	D01	0	0	kleiner LLOQ	#VALUE!	1	#VALUE!
0	T14	D02	0	0	kleiner LLOQ	#VALUE!	1	#VALUE!
0	T14	D03	0	0	kleiner LLOQ	#VALUE!	1	#VALUE!
0	T14	D04	0	0	kleiner LLOQ	#VALUE!	1	#VALUE!
0	T14	D05	0	0	kleiner LLOQ	#VALUE!	1	#VALUE!
0	T14	D06	0	0	kleiner LLOQ	#VALUE!	1	#VALUE!
T14SN2	T14	D07	0.467	0.416	0.416	0.018	2.75	0.050
T14SN2	T14	D08	0.518	0.467	0.467	0.021	2.75	0.057
T14SN3	T14	D09	0.607	0.556	0.556	0.025	2.75	0.068
T14SN3	T14	D10	0.573	0.522	0.522	0.023	2.75	0.064
T14SN4	T14	D11	0.510	0.459	0.459	0.020	2.75	0.056
T14SN4	T14	D12	0.574	0.523	0.523	0.023	2.75	0.064
0	T14	E01	0	0	kleiner LLOQ	#VALUE!	1	#VALUE!
0	T14	E02	0	0	kleiner LLOQ	#VALUE!	1	#VALUE!
0	T14	E03	0	0	kleiner LLOQ	#VALUE!	1	#VALUE!
0	T14	E04	0	0	kleiner LLOQ	#VALUE!	1	#VALUE!
T14SS11	T14	E05	0.448	0.397	0.397	0.017	2.75	0.047
T14SS11	T14	E06	0.454	0.403	0.403	0.018	2.75	0.048
T14SS12	T14	E07	0.290	0.239	0.239	0.010	2.75	0.027
T14SS12	T14	E08	0.288	0.237	0.237	0.010	2.75	0.027
T14SS13	T14	E09	0.177	0.126	kleiner LLOQ	#VALUE!	2.75	#VALUE!
T14SS13	T14	E10	0.183	0.132	kleiner LLOQ	#VALUE!	2.75	#VALUE!
T14SS14	T14	E11	0.311	0.260	0.260	0.011	2.75	0.030
T14SS14	T14	E12	0.279	0.228	0.228	0.009	2.75	0.025
0	T14	F01	0	0	kleiner LLOQ	#VALUE!	1	#VALUE!
0	T14	F02	0	0	kleiner LLOQ	#VALUE!	1	#VALUE!
0	T14	F03	0	0	kleiner LLOQ	#VALUE!	1	#VALUE!
0	T14	F04	0	0	kleiner LLOQ	#VALUE!	1	#VALUE!
T14SS21	T14	F05	0.471	0.420	0.420	0.018	2.75	0.05
T14SS21	T14	F06	0.511	0.460	0.460	0.020	2.75	0.06
T14SS22	T14	F07	0.236	0.185	0.185	0.007	2.75	0.02
T14SS22	T14	F08	0.261	0.210	0.210	0.008	2.75	0.02
T14SS23	T14	F09	0.259	0.208	0.208	0.008	2.75	0.02
T14SS23	T14	F10	0.349	0.298	0.298	0.013	2.75	0.03
T14SS24	T14	F11	0.350	0.299	0.299	0.013	2.75	0.03
T14SS24	T14	F12	0.224	0.173	0.173	0.007	2.75	0.02
0	T14	G01	0	0	kleiner LLOQ	#VALUE!	1	#VALUE!
0	T14	G02	0	0	kleiner LLOQ	#VALUE!	1	#VALUE!
0	T14	G03	0	0	kleiner LLOQ	#VALUE!	1	#VALUE!
0	T14	G04	0	0	kleiner LLOQ	#VALUE!	1	#VALUE!
0	T14	G05	0	0	kleiner LLOQ	#VALUE!	1	#VALUE!
0	T14	G06	0	0	kleiner LLOQ	#VALUE!	1	#VALUE!
Blank PEG	T14	G07	0	0	kleiner LLOQ	#VALUE!	2.75	#VALUE!
Blank PEG	T14	G08	0	0	kleiner LLOQ	#VALUE!	2.75	#VALUE!
Blank PEG	T14	G09	0	0	kleiner LLOQ	#VALUE!	2.75	#VALUE!
Blank PEG	T14	G10	0	0	kleiner LLOQ	#VALUE!	2.75	#VALUE!
Blank PEG	T14	G11	0	0	kleiner LLOQ	#VALUE!	2.75	#VALUE!
Blank PEG	T14	G12	0	0	kleiner LLOQ	#VALUE!	2.75	#VALUE!
0	T14	H01	0	0	kleiner LLOQ	#VALUE!	1	#VALUE!
0	T14	H02	0	0	kleiner LLOQ	#VALUE!	1	#VALUE!
0	T14	H03	0	0	kleiner LLOQ	#VALUE!	1	#VALUE!
0	T14	H04	0	0	kleiner LLOQ	#VALUE!	1	#VALUE!
0	T14	H05	0	0	kleiner LLOQ	#VALUE!	1	#VALUE!
0	T14	H06	0	0	kleiner LLOQ	#VALUE!	1	#VALUE!
Blank no PEG	T14	H07	0	0	kleiner LLOQ	#VALUE!	2.75	#VALUE!
Blank no PEG	T14	H08	0	0	kleiner LLOQ	#VALUE!	2.75	#VALUE!
Blank no PEG	T14	H09	0	0	kleiner LLOQ	#VALUE!	2.75	#VALUE!
Blank no PEG	T14	H10	0	0	kleiner LLOQ	#VALUE!	2.75	#VALUE!
Blank no PEG	T14	H11	0	0	kleiner LLOQ	#VALUE!	2.75	#VALUE!
Blank no PEG	T14	H12	0	0	kleiner LLOQ	#VALUE!	2.75	#VALUE!

F					T14SS21	T14SS21	T14SS22	T14SS22	T14SS23	T14SS23	T14SS24	T14SS24
G							Blank PEG	Blank PEG	Blank PEG	Blank PEG	Blank PEG	Blank PEG
H							Blank no PEG	Blank no PEG	Blank no PEG	Blank no PEG	Blank no PEG	Blank no PEG

Comment:

Date Operator

Date Control

Project number		F-120	Apparatus	Wallac Victor	Operator	IsBa
GLP Study (Number)		n.a.	Protocol (Instrument meth	LDH test 2016	Date of preparation	19-04-18
hot substance	isotope	n.a.	File name (results)	IsBa_180419/20_LDH_full_	Date of measurement	19-04-18
	name	n.a.	Kind of well plate	96 well	shaking time [min]	30
	ACB-ID	n.a.	sample volume [µL]	100	stirring rate (Target) [rpm]	150
	Batch number	n.a.	Cocktail volume [µL]	175	Kind of measurement	UV-vis
cold substance	name	LDH	ACB-ID of cocktail		Wave length [nm]	450
	ACB-ID	L23982R	Matrix	DMEM (from powder)+PE(	Remarks	Cocktail 100µl RM, 75µl STOP
	Batch number	SLBK6345V	Blank description	DMEM/PEG, H2O	Remarks	7 standards split low/high
n.a.		n.a.	Pipettes (No. / volume)	50-200µl	Remarks	KLP4 common for both
n.a.		n.a.	Pipettes (No. / volume)	n.a.	Remarks	n.a.

Messdaten (diese Tabelle in Bericht übernehmen)

Sample name *	concentration (theor.) *	measured data	measured data	measured data	mean measured	SD	RSD	Blank *	measured data after * Blank subtraction	concentration (calc.) *	Deviation *	Residuen
	[µg/mL]	[AU]	[AU]	[AU]	[AU]	[AU]	[%]	[AU]		[µg/mL]	[%]	
KLP1	0.148	1.579	1.652	1.627	1.62	0.03	1.87	0.047	1.568	0.148	-0.24	0.00
KLP2	0.114	1.357	1.443	1.372	1.39	0.04	2.70	0.054	1.340	0.110	-3.40	0.00
KLP3	0.074	1.272	1.193	1.228	1.23	0.03	2.63	0.051	1.180	0.084	13.04	0.01
KLP4	0.041	0.959	0.972	0.883	0.94	0.04	4.19	0.051	0.887	0.035	-13.32	-0.01
KLP5								0.052				
KLP6								0.051				
KLP7												
KLP8												

Statistical data

Geradensteigung	Slope	m	6.05
Y-Achsenabschnitt	Y-Intercept	b	0.67
Standardabw. Geradensteigung	SD-Slope	s_m	0.620291227
Standardabw. Achsenabschnittes	SD-Y-Intercept	s_b	0.063694294
Anzahl Messpunkte	number of measuring points	n	4
Quadratsumme	sum of squares	Qxx	0.006578604
Bereichsmitte			0.094336926
Freiheitsgrade	degree of freedom	f	2
Student-t-Faktor für (P = 95 %; f = n-2)	Student-t-factor for (P = 95 %; f = n-2)	t	4.303
Vertrauensbereich Steig. (95 %) Obergrenze		$m + VB_m$	8.722319527
Vertrauensbereich Steig. (95 %) Untergrenze		$m - VB_m$	3.384093228
Vertrauensbereich Achsenabschnitt (95 %) Og.		$b + Vb_b$	0.946914615
Vertrauensbereich Achsenabschnitt (95 %) Ug.		$b - Vb_b$	0.398761518
Korrelationskoeffizient	correlation coefficient	r	0.9897
Bestimmtheitsmaß	determiantion coefficient	r^2	0.9794
Reststandardabweichung		s_0	0.050310948
Summe Restquadrate		sd	2.895583345
Verfahrensstandardabw.		s_{x0}	0.008311454
Rel. Verfahrensstandardabw. %		V_{x0}	8.810393374

mean Blank	**0**	
SD Blank	0.00	
RSD Blank	3.79	x=
x*SD (LLOQ)	0	5
x*SD (LOD)	0	3
**LLOQ (AU)**	**0**	
**LOD (AU)**	**0**	
ULOQ	1.568	
LLOQ (Lin)	0.887	

Evaluation / Comment

LDH linearity valid with 7 standards, split in lin high and lin low, each with 4 standards and KLP4 common standard for both. R^2 0,9981, deviations for both between -13.34% and +13.04%

Date Operator Date Control

Figure 1 **Linearity**

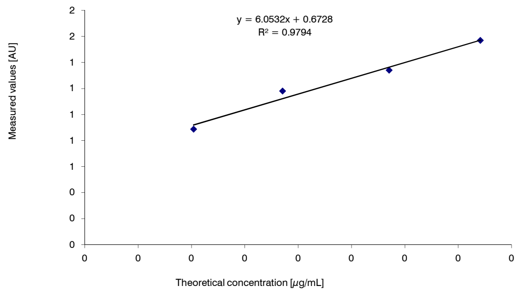

Figure 2 **Method validation Residuen Plot**

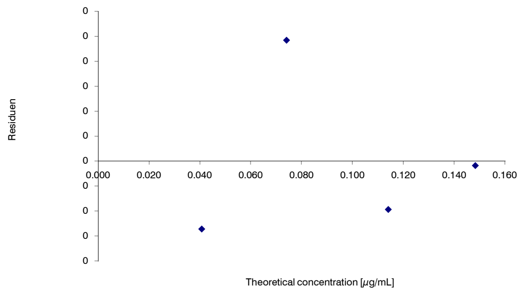

Evaluation / Comment

lin passed

Date	Operator	Date	Control

Sample name	Plate	Position	[AU]	[AU]-Blank	Rating	[µg/mL]	Dilution factor	[µg/mL]
0	T12	A01	0	0	kleiner LLOQ	#VALUE!	1	#VALUE!
0	T12	A02	0	0	kleiner LLOQ	#VALUE!	1	#VALUE!
0	T12	A03	0	0	kleiner LLOQ	#VALUE!	1	#VALUE!
0	T12	A04	0	0	kleiner LLOQ	#VALUE!	1	#VALUE!
0	T12	A05	0.000	-0.051	kleiner LLOQ	#VALUE!		#VALUE!
0	T12	A06	0.000	-0.051	kleiner LLOQ	#VALUE!		#VALUE!
0	T12	A07	0	0	kleiner LLOQ	#VALUE!		#VALUE!
0	T12	A08	0	0	kleiner LLOQ	#VALUE!		#VALUE!
0	T12	A09	0.000	-0.051	kleiner LLOQ	#VALUE!		#VALUE!
0	T12	A10	0.000	-0.051	kleiner LLOQ	#VALUE!		#VALUE!
0	T12	A11	0	0	kleiner LLOQ	#VALUE!		#VALUE!
0	T12	A12	0	0	kleiner LLOQ	#VALUE!		#VALUE!
0	T12	B01	0	0	kleiner LLOQ	#VALUE!		#VALUE!
0	T12	B02	0	0	kleiner LLOQ	#VALUE!		#VALUE!
0	T12	B03	0	0	kleiner LLOQ	#VALUE!		#VALUE!
0	T12	B04	0	0	kleiner LLOQ	#VALUE!		#VALUE!
0	T12	B05	0	0	kleiner LLOQ	#VALUE!		#VALUE!
0	T12	B06	0.000	-0.051	kleiner LLOQ	#VALUE!		#VALUE!
0	T12	B07	0.000	-0.051	kleiner LLOQ	#VALUE!		#VALUE!
0	T12	B08	0.000	-0.051	kleiner LLOQ	#VALUE!		#VALUE!
0	T12	B09	0	0	kleiner LLOQ	#VALUE!		#VALUE!
0	T12	B10	0	0	kleiner LLOQ	#VALUE!		#VALUE!
0	T12	B11	0	0	kleiner LLOQ	#VALUE!		#VALUE!
0	T12	B12	0	0	kleiner LLOQ	#VALUE!		#VALUE!
0	T12	C01	0	0	kleiner LLOQ	#VALUE!		#VALUE!
0	T12	C02	0	0	kleiner LLOQ	#VALUE!		#VALUE!
0	T12	C03	0	0	kleiner LLOQ	#VALUE!		#VALUE!
0	T12	C04	0	0	kleiner LLOQ	#VALUE!		#VALUE!
0	T12	C05	0.000	-0.051	kleiner LLOQ	#VALUE!		#VALUE!
0	T12	C06	0.000	-0.051	kleiner LLOQ	#VALUE!		#VALUE!
0	T12	C07	0.000	-0.051	kleiner LLOQ	#VALUE!		#VALUE!
0	T12	C08	0.000	-0.051	kleiner LLOQ	#VALUE!		#VALUE!
0	T12	C09	0.000	-0.051	kleiner LLOQ	#VALUE!		#VALUE!
0	T12	C10	0.000	-0.051	kleiner LLOQ	#VALUE!		#VALUE!
0	T12	C11	0	0	kleiner LLOQ	#VALUE!	1	#VALUE!
0	T12	C12	0	0	kleiner LLOQ	#VALUE!	1	#VALUE!
0	T12	D01	0	0	kleiner LLOQ	#VALUE!	1	#VALUE!
0	T12	D02	0	0	kleiner LLOQ	#VALUE!	1	#VALUE!
0	T12	D03	0	0	kleiner LLOQ	#VALUE!	1	#VALUE!
0	T12	D04	0	0	kleiner LLOQ	#VALUE!	1	#VALUE!
0	T12	D05	0	0	kleiner LLOQ	#VALUE!	1	#VALUE!
0	T12	D06	0	0	kleiner LLOQ	#VALUE!	1	#VALUE!
T12SN2	T12	D07	0	0	kleiner LLOQ	#VALUE!	1	#VALUE!
T12SN2	T12	D08	0	0	kleiner LLOQ	#VALUE!	1	#VALUE!
T12SN3	T12	D09	0	0	kleiner LLOQ	#VALUE!	1	#VALUE!
T12SN3	T12	D10	0	0	kleiner LLOQ	#VALUE!	1	#VALUE!
T12SN4	T12	D11	0	0	kleiner LLOQ	#VALUE!	1	#VALUE!
T12SN4	T12	D12	0	0	kleiner LLOQ	#VALUE!	1	#VALUE!

Plate: T12

	1	2	3	4	5	6	7	8	9	10	11	12
A												
B												
C												
D												
E												
F												
G												
H												

Plattenbelegung

	1	2	3	4	5	6	7	8	9	10	11	12
A												
B												
C												
D							T12SN2	T12SN2	T12SN3	T12SN3	T12SN4	T12SN4
E					T12SS11	T12SS11	T12SS12	T12SS12	T12SS13	T12SS13	T12SS14	T12SS14
F					T12SS21	T12SS21	T12SS22	T12SS22	T12SS23	T12SS23	T12SS24	T12SS24
G							Blank PEG	Blank PEG	Blank PEG	Blank PEG	Blank PEG	Blank PEG
H							Blank no PEG	Blank no PEG	Blank no PEG	Blank no PEG	Blank no PEG	Blank no PEG

Comment:

alle Messwerte bei lin low

Samples aufgeteilt in lin high und low:niedriger konzentrierte Samples bei lin low zu finden.

Date Operator

Date Control

0	T12	E01	0	0	kleiner LLOQ	#VALUE!	1	#VALUE!
0	T12	E02	0	0	kleiner LLOQ	#VALUE!	1	#VALUE!
0	T12	E03	0	0	kleiner LLOQ	#VALUE!	1	#VALUE!
0	T12	E04	0	0	kleiner LLOQ	#VALUE!	1	#VALUE!
T12SS11	T12	E05	0	0	kleiner LLOQ	#VALUE!	1	#VALUE!
T12SS11	T12	E06	0	0	kleiner LLOQ	#VALUE!	1	#VALUE!
T12SS12	T12	E07	0	0	kleiner LLOQ	#VALUE!	1	#VALUE!
T12SS12	T12	E08	0	0	kleiner LLOQ	#VALUE!	1	#VALUE!
T12SS13	T12	E09	0	0	kleiner LLOQ	#VALUE!	1	#VALUE!
T12SS13	T12	E10	0	0	kleiner LLOQ	#VALUE!	1	#VALUE!
T12SS14	T12	E11	0	0	kleiner LLOQ	#VALUE!	1	#VALUE!
T12SS14	T12	E12	0	0	kleiner LLOQ	#VALUE!	1	#VALUE!
0	T12	F01	0	0	kleiner LLOQ	#VALUE!	1	#VALUE!
0	T12	F02	0	0	kleiner LLOQ	#VALUE!	1	#VALUE!
0	T12	F03	0	0	kleiner LLOQ	#VALUE!	1	#VALUE!
0	T12	F04	0	0	kleiner LLOQ	#VALUE!	1	#VALUE!
T12SS21	T12	F05	0	0	kleiner LLOQ	#VALUE!	1	#VALUE!
T12SS21	T12	F06	0	0	kleiner LLOQ	#VALUE!	1	#VALUE!
T12SS22	T12	F07	0	0	kleiner LLOQ	#VALUE!	1	#VALUE!
T12SS22	T12	F08	0	0	kleiner LLOQ	#VALUE!	1	#VALUE!
T12SS23	T12	F09	0	0	kleiner LLOQ	#VALUE!	1	#VALUE!
T12SS23	T12	F10	0	0	kleiner LLOQ	#VALUE!	1	#VALUE!
T12SS24	T12	F11	0	0	kleiner LLOQ	#VALUE!	1	#VALUE!
T12SS24	T12	F12	0	0	kleiner LLOQ	#VALUE!	1	#VALUE!
0	T12	G01	0	0	kleiner LLOQ	#VALUE!	1	#VALUE!
0	T12	G02	0	0	kleiner LLOQ	#VALUE!	1	#VALUE!
0	T12	G03	0	0	kleiner LLOQ	#VALUE!	1	#VALUE!
0	T12	G04	0	0	kleiner LLOQ	#VALUE!	1	#VALUE!
0	T12	G05	0	0	kleiner LLOQ	#VALUE!	1	#VALUE!
0	T12	G06	0	0	kleiner LLOQ	#VALUE!	1	#VALUE!
Blank PEG	T12	G07	0.000	-0.051	kleiner LLOQ	#VALUE!	2.75	#VALUE!
Blank PEG	T12	G08	0.000	-0.051	kleiner LLOQ	#VALUE!	2.75	#VALUE!
Blank PEG	T12	G09	0.000	-0.051	kleiner LLOQ	#VALUE!	2.75	#VALUE!
Blank PEG	T12	G10	0.000	-0.051	kleiner LLOQ	#VALUE!	2.75	#VALUE!
Blank PEG	T12	G11	0.000	-0.051	kleiner LLOQ	#VALUE!	2.75	#VALUE!
Blank PEG	T12	G12	0.000	-0.051	kleiner LLOQ	#VALUE!	2.75	#VALUE!
0	T12	H01	0	0	kleiner LLOQ	#VALUE!	1	#VALUE!
0	T12	H02	0	0	kleiner LLOQ	#VALUE!	1	#VALUE!
0	T12	H03	0	0	kleiner LLOQ	#VALUE!	1	#VALUE!
0	T12	H04	0	0	kleiner LLOQ	#VALUE!	1	#VALUE!
0	T12	H05	0	0	kleiner LLOQ	#VALUE!	1	#VALUE!
0	T12	H06	0	0	kleiner LLOQ	#VALUE!	1	#VALUE!
Blank no PEG	T12	H07	0.000	-0.051	kleiner LLOQ	#VALUE!	2.75	#VALUE!
Blank no PEG	T12	H08	0.000	-0.051	kleiner LLOQ	#VALUE!	2.75	#VALUE!
Blank no PEG	T12	H09	0.000	-0.051	kleiner LLOQ	#VALUE!	2.75	#VALUE!
Blank no PEG	T12	H10	0.000	-0.051	kleiner LLOQ	#VALUE!	2.75	#VALUE!
Blank no PEG	T12	H11	0.000	-0.051	kleiner LLOQ	#VALUE!	2.75	#VALUE!
Blank no PEG	T12	H12	0.000	-0.051	kleiner LLOQ	#VALUE!	2.75	#VALUE!

0	T13	A01	0	0	kleiner LLOQ	#VALUE!	1	#VALUE!
0	T13	A02	0	0	kleiner LLOQ	#VALUE!	1	#VALUE!
0	T13	A03	0	0	kleiner LLOQ	#VALUE!	1	#VALUE!
0	T13	A04	0	0	kleiner LLOQ	#VALUE!	1	#VALUE!
0	T13	A05	0	0	kleiner LLOQ	#VALUE!	1	#VALUE!
0	T13	A06	0	0	kleiner LLOQ	#VALUE!	1	#VALUE!
0	T13	A07	0	0	kleiner LLOQ	#VALUE!	1	#VALUE!
0	T13	A08	0	0	kleiner LLOQ	#VALUE!	1	#VALUE!
0	T13	A09	0	0	kleiner LLOQ	#VALUE!	1	#VALUE!
0	T13	A10	0	0	kleiner LLOQ	#VALUE!	1	#VALUE!
0	T13	A11	0	0	kleiner LLOQ	#VALUE!	1	#VALUE!
0	T13	A12	0	0	kleiner LLOQ	#VALUE!	1	#VALUE!
0	T13	B01	0	0	kleiner LLOQ	#VALUE!	1	#VALUE!
0	T13	B02	0	0	kleiner LLOQ	#VALUE!	1	#VALUE!
0	T13	B03	0	0	kleiner LLOQ	#VALUE!	1	#VALUE!
0	T13	B04	0	0	kleiner LLOQ	#VALUE!	1	#VALUE!
0	T13	B05	0	0	kleiner LLOQ	#VALUE!	1	#VALUE!
0	T13	B06	0	0	kleiner LLOQ	#VALUE!	1	#VALUE!
0	T13	B07	0	0	kleiner LLOQ	#VALUE!	1	#VALUE!
0	T13	B08	0	0	kleiner LLOQ	#VALUE!	1	#VALUE!
0	T13	B09	0	0	kleiner LLOQ	#VALUE!	1	#VALUE!
0	T13	B10	0	0	kleiner LLOQ	#VALUE!	1	#VALUE!
0	T13	B11	0	0	kleiner LLOQ	#VALUE!	1	#VALUE!
0	T13	B12	0	0	kleiner LLOQ	#VALUE!	1	#VALUE!
0	T13	C01	0	0	kleiner LLOQ	#VALUE!	1	#VALUE!
0	T13	C02	0	0	kleiner LLOQ	#VALUE!	1	#VALUE!
0	T13	C03	0	0	kleiner LLOQ	#VALUE!	1	#VALUE!
0	T13	C04	0	0	kleiner LLOQ	#VALUE!	1	#VALUE!
0	T13	C05	0	0	kleiner LLOQ	#VALUE!	1	#VALUE!
0	T13	C06	0	0	kleiner LLOQ	#VALUE!	1	#VALUE!
0	T13	C07	0	0	kleiner LLOQ	#VALUE!	1	#VALUE!
0	T13	C08	0	0	kleiner LLOQ	#VALUE!	1	#VALUE!
0	T13	C09	0	0	kleiner LLOQ	#VALUE!	1	#VALUE!
0	T13	C10	0	0	kleiner LLOQ	#VALUE!	1	#VALUE!
0	T13	C11	0	0	kleiner LLOQ	#VALUE!	1	#VALUE!
0	T13	C12	0	0	kleiner LLOQ	#VALUE!	1	#VALUE!
0	T13	D01	0	0	kleiner LLOQ	#VALUE!	1	#VALUE!
0	T13	D02	0	0	kleiner LLOQ	#VALUE!	1	#VALUE!
0	T13	D03	0	0	kleiner LLOQ	#VALUE!	1	#VALUE!
0	T13	D04	0	0	kleiner LLOQ	#VALUE!	1	#VALUE!
0	T13	D05	0	0	kleiner LLOQ	#VALUE!	1	#VALUE!
0	T13	D06	0	0	kleiner LLOQ	#VALUE!	1	#VALUE!
T13SN2	T13	D07	0	0	kleiner LLOQ	#VALUE!	1	#VALUE!
T13SN2	T13	D08	0	0	kleiner LLOQ	#VALUE!	1	#VALUE!
T13SN3	T13	D09	0	0	kleiner LLOQ	#VALUE!	1	#VALUE!
T13SN3	T13	D10	0	0	kleiner LLOQ	#VALUE!	1	#VALUE!
T13SN4	T13	D11	0	0	kleiner LLOQ	#VALUE!	1	#VALUE!
T13SN4	T13	D12	0	0	kleiner LLOQ	#VALUE!	1	#VALUE!

Plate: T13

	1	2	3	4	5	6	7	8	9	10	11	12
A												
B												
C												
D												
E												
F												
G												
H												

Plattenbelegung

	1	2	3	4	5	6	7	8	9	10	11	12
A												
B												
C												
D							T13SN2	T13SN2	T13SN3	T13SN3	T13SN4	T13SN4
E					T13SS11	T13SS11	T13SS12	T13SS12	T13SS13	T13SS13	T13SS14	T13SS14
F					T13SS21	T13SS21	T13SS22	T13SS22	T13SS23	T13SS23	T13SS24	T13SS24
G							Blank PEG	Blank PEG	Blank PEG	Blank PEG	Blank PEG	Blank PEG
H							Blank no PE[illegible]	Blank no PE[illegible]	Blank no PE[illegible]	Blank no PE[illegible]	Blank no PE[illegible]	Blank no PEG

Comment:

alle Messwerte bei lin low

Date Operator

Date Control

0	T13	E01	0	0	kleiner LLOQ	#VALUE!	1	#VALUE!
0	T13	E02	0	0	kleiner LLOQ	#VALUE!	1	#VALUE!
0	T13	E03	0	0	kleiner LLOQ	#VALUE!	1	#VALUE!
0	T13	E04	0	0	kleiner LLOQ	#VALUE!	1	#VALUE!
T13SS11	T13	E05	0	0	kleiner LLOQ	#VALUE!	1	#VALUE!
T13SS11	T13	E06	0	0	kleiner LLOQ	#VALUE!	1	#VALUE!
T13SS12	T13	E07	0	0	kleiner LLOQ	#VALUE!	1	#VALUE!
T13SS12	T13	E08	0	0	kleiner LLOQ	#VALUE!	1	#VALUE!
T13SS13	T13	E09	0	0	kleiner LLOQ	#VALUE!	1	#VALUE!
T13SS13	T13	E10	0	0	kleiner LLOQ	#VALUE!	1	#VALUE!
T13SS14	T13	E11	0	0	kleiner LLOQ	#VALUE!	1	#VALUE!
T13SS14	T13	E12	0	0	kleiner LLOQ	#VALUE!	1	#VALUE!
0	T13	F01	0	0	kleiner LLOQ	#VALUE!	1	#VALUE!
0	T13	F02	0	0	kleiner LLOQ	#VALUE!	1	#VALUE!
0	T13	F03	0	0	kleiner LLOQ	#VALUE!	1	#VALUE!
0	T13	F04	0	0	kleiner LLOQ	#VALUE!	1	#VALUE!
T13SS21	T13	F05	0	0	kleiner LLOQ	#VALUE!	1	#VALUE!
T13SS21	T13	F06	0	0	kleiner LLOQ	#VALUE!	1	#VALUE!
T13SS22	T13	F07	0	0	kleiner LLOQ	#VALUE!	1	#VALUE!
T13SS22	T13	F08	0	0	kleiner LLOQ	#VALUE!	1	#VALUE!
T13SS23	T13	F09	0	0	kleiner LLOQ	#VALUE!	1	#VALUE!
T13SS23	T13	F10	0	0	kleiner LLOQ	#VALUE!	1	#VALUE!
T13SS24	T13	F11	0	0	kleiner LLOQ	#VALUE!	1	#VALUE!
T13SS24	T13	F12	0	0	kleiner LLOQ	#VALUE!	1	#VALUE!
0	T13	G01	0	0	kleiner LLOQ	#VALUE!	1	#VALUE!
0	T13	G02	0	0	kleiner LLOQ	#VALUE!	1	#VALUE!
0	T13	G03	0	0	kleiner LLOQ	#VALUE!	1	#VALUE!
0	T13	G04	0	0	kleiner LLOQ	#VALUE!	1	#VALUE!
0	T13	G05	0	0	kleiner LLOQ	#VALUE!	1	#VALUE!
0	T13	G06	0	0	kleiner LLOQ	#VALUE!	1	#VALUE!
Blank PEG	T13	G07	0	0	kleiner LLOQ	#VALUE!	1	#VALUE!
Blank PEG	T13	G08	0	0	kleiner LLOQ	#VALUE!	1	#VALUE!
Blank PEG	T13	G09	0	0	kleiner LLOQ	#VALUE!	1	#VALUE!
Blank PEG	T13	G10	0	0	kleiner LLOQ	#VALUE!	1	#VALUE!
Blank PEG	T13	G11	0	0	kleiner LLOQ	#VALUE!	1	#VALUE!
Blank PEG	T13	G12	0	0	kleiner LLOQ	#VALUE!	1	#VALUE!
0	T13	H01	0	0	kleiner LLOQ	#VALUE!	1	#VALUE!
0	T13	H02	0	0	kleiner LLOQ	#VALUE!	1	#VALUE!
0	T13	H03	0	0	kleiner LLOQ	#VALUE!	1	#VALUE!
0	T13	H04	0	0	kleiner LLOQ	#VALUE!	1	#VALUE!
0	T13	H05	0	0	kleiner LLOQ	#VALUE!	1	#VALUE!
0	T13	H06	0	0	kleiner LLOQ	#VALUE!	1	#VALUE!
Blank no PEG	T13	H07	0	0	kleiner LLOQ	#VALUE!	1	#VALUE!
Blank no PEG	T13	H08	0	0	kleiner LLOQ	#VALUE!	1	#VALUE!
Blank no PEG	T13	H09	0	0	kleiner LLOQ	#VALUE!	1	#VALUE!
Blank no PEG	T13	H10	0	0	kleiner LLOQ	#VALUE!	1	#VALUE!
Blank no PEG	T13	H11	0	0	kleiner LLOQ	#VALUE!	1	#VALUE!
Blank no PEG	T13	H12	0	0	kleiner LLOQ	#VALUE!	1	#VALUE!
0	T14	A01	0	0	kleiner LLOQ	#VALUE!	1	#VALUE!
0	T14	A02	0	0	kleiner LLOQ	#VALUE!	1	#VALUE!
0	T14	A03	0	0	kleiner LLOQ	#VALUE!	1	#VALUE!
0	T14	A04	0	0	kleiner LLOQ	#VALUE!	1	#VALUE!
0	T14	A05	0	0	kleiner LLOQ	#VALUE!	1	#VALUE!
0	T14	A06	0	0	kleiner LLOQ	#VALUE!	1	#VALUE!
0	T14	A07	0	0	kleiner LLOQ	#VALUE!	1	#VALUE!
0	T14	A08	0	0	kleiner LLOQ	#VALUE!	1	#VALUE!
0	T14	A09	0	0	kleiner LLOQ	#VALUE!	1	#VALUE!
0	T14	A10	0	0	kleiner LLOQ	#VALUE!	1	#VALUE!
0	T14	A11	0	0	kleiner LLOQ	#VALUE!	1	#VALUE!
0	T14	A12	0	0	kleiner LLOQ	#VALUE!	1	#VALUE!
0	T14	B01	0	0	kleiner LLOQ	#VALUE!	1	#VALUE!
0	T14	B02	0	0	kleiner LLOQ	#VALUE!	1	#VALUE!
0	T14	B03	0	0	kleiner LLOQ	#VALUE!	1	#VALUE!
0	T14	B04	0	0	kleiner LLOQ	#VALUE!	1	#VALUE!
0	T14	B05	0	0	kleiner LLOQ	#VALUE!	1	#VALUE!
0	T14	B06	0	0	kleiner LLOQ	#VALUE!	1	#VALUE!
0	T14	B07	0	0	kleiner LLOQ	#VALUE!	1	#VALUE!
0	T14	B08	0	0	kleiner LLOQ	#VALUE!	1	#VALUE!
0	T14	B09	0	0	kleiner LLOQ	#VALUE!	1	#VALUE!
0	T14	B10	0	0	kleiner LLOQ	#VALUE!	1	#VALUE!
0	T14	B11	0	0	kleiner LLOQ	#VALUE!	1	#VALUE!
0	T14	B12	0	0	kleiner LLOQ	#VALUE!	1	#VALUE!
0	T14	C01	0	0	kleiner LLOQ	#VALUE!	1	#VALUE!
0	T14	C02	0	0	kleiner LLOQ	#VALUE!	1	#VALUE!
0	T14	C03	0	0	kleiner LLOQ	#VALUE!	1	#VALUE!

Plate: T14

	1	2	3	4	5	6	7	8	9	10	11	12
A												
B												
C												
D												
E												
F												
G												
H												

Plattenbelegung

	1	2	3	4	5	6	7	8	9	10	11	12
A												
B												
C												
D							T14SN2	T14SN2	T14SN3	T14SN3	T14SN4	T14SN4
E					T14SS11	T14SS11	T14SS12	T14SS12	T14SS13	T14SS13	T14SS14	T14SS14
F					T14SS21	T14SS21	T14SS22	T14SS22	T14SS23	T14SS23	T14SS24	T14SS24
G							Blank PEG	Blank PEG	Blank PEG	Blank PEG	Blank PEG	Blank PEG

0	T14	C04	0	0	kleiner LLOQ	#VALUE!	1	#VALUE!
0	T14	C05	0	0	kleiner LLOQ	#VALUE!	1	#VALUE!
0	T14	C06	0	0	kleiner LLOQ	#VALUE!	1	#VALUE!
0	T14	C07	0	0	kleiner LLOQ	#VALUE!	1	#VALUE!
0	T14	C08	0	0	kleiner LLOQ	#VALUE!	1	#VALUE!
0	T14	C09	0	0	kleiner LLOQ	#VALUE!	1	#VALUE!
0	T14	C10	0	0	kleiner LLOQ	#VALUE!	1	#VALUE!
0	T14	C11	0	0	kleiner LLOQ	#VALUE!	1	#VALUE!
0	T14	C12	0	0	kleiner LLOQ	#VALUE!	1	#VALUE!
0	T14	D01	0	0	kleiner LLOQ	#VALUE!	1	#VALUE!
0	T14	D02	0	0	kleiner LLOQ	#VALUE!	1	#VALUE!
0	T14	D03	0	0	kleiner LLOQ	#VALUE!	1	#VALUE!
0	T14	D04	0	0	kleiner LLOQ	#VALUE!	1	#VALUE!
0	T14	D05	0	0	kleiner LLOQ	#VALUE!	1	#VALUE!
0	T14	D06	0	0	kleiner LLOQ	#VALUE!	1	#VALUE!
T14SN2	T14	D07	0	0	kleiner LLOQ	#VALUE!	1	#VALUE!
T14SN2	T14	D08	0	0	kleiner LLOQ	#VALUE!	1	#VALUE!
T14SN3	T14	D09	0	0	kleiner LLOQ	#VALUE!	1	#VALUE!
T14SN3	T14	D10	0	0	kleiner LLOQ	#VALUE!	1	#VALUE!
T14SN4	T14	D11	0	0	kleiner LLOQ	#VALUE!	1	#VALUE!
T14SN4	T14	D12	0	0	kleiner LLOQ	#VALUE!	1	#VALUE!
0	T14	E01	0	0	kleiner LLOQ	#VALUE!	1	#VALUE!
0	T14	E02	0	0	kleiner LLOQ	#VALUE!	1	#VALUE!
0	T14	E03	0	0	kleiner LLOQ	#VALUE!	1	#VALUE!
0	T14	E04	0	0	kleiner LLOQ	#VALUE!	1	#VALUE!
T14SS11	T14	E05	0	0	kleiner LLOQ	#VALUE!	1	#VALUE!
T14SS11	T14	E06	0	0	kleiner LLOQ	#VALUE!	1	#VALUE!
T14SS12	T14	E07	0	0	kleiner LLOQ	#VALUE!	1	#VALUE!
T14SS12	T14	E08	0	0	kleiner LLOQ	#VALUE!	1	#VALUE!
T14SS13	T14	E09	0	0	kleiner LLOQ	#VALUE!	1	#VALUE!
T14SS13	T14	E10	0	0	kleiner LLOQ	#VALUE!	1	#VALUE!
T14SS14	T14	E11	0	0	kleiner LLOQ	#VALUE!	1	#VALUE!
T14SS14	T14	E12	0	0	kleiner LLOQ	#VALUE!	1	#VALUE!
0	T14	F01	0	0	kleiner LLOQ	#VALUE!	1	#VALUE!
0	T14	F02	0	0	kleiner LLOQ	#VALUE!	1	#VALUE!
0	T14	F03	0	0	kleiner LLOQ	#VALUE!	1	#VALUE!
0	T14	F04	0	0	kleiner LLOQ	#VALUE!	1	#VALUE!
T14SS21	T14	F05	0	0	kleiner LLOQ	#VALUE!	1	#VALUE!
T14SS21	T14	F06	0	0	kleiner LLOQ	#VALUE!	1	#VALUE!
T14SS22	T14	F07	0	0	kleiner LLOQ	#VALUE!	1	#VALUE!
T14SS22	T14	F08	0	0	kleiner LLOQ	#VALUE!	1	#VALUE!
T14SS23	T14	F09	0	0	kleiner LLOQ	#VALUE!	1	#VALUE!
T14SS23	T14	F10	0	0	kleiner LLOQ	#VALUE!	1	#VALUE!
T14SS24	T14	F11	0	0	kleiner LLOQ	#VALUE!	1	#VALUE!
T14SS24	T14	F12	0	0	kleiner LLOQ	#VALUE!	1	#VALUE!
0	T14	G01	0	0	kleiner LLOQ	#VALUE!	1	#VALUE!
0	T14	G02	0	0	kleiner LLOQ	#VALUE!	1	#VALUE!
0	T14	G03	0	0	kleiner LLOQ	#VALUE!	1	#VALUE!
0	T14	G04	0	0	kleiner LLOQ	#VALUE!	1	#VALUE!
0	T14	G05	0	0	kleiner LLOQ	#VALUE!	1	#VALUE!
0	T14	G06	0	0	kleiner LLOQ	#VALUE!	1	#VALUE!
Blank PEG	T14	G07	0	0	kleiner LLOQ	#VALUE!	1	#VALUE!
Blank PEG	T14	G08	0	0	kleiner LLOQ	#VALUE!	1	#VALUE!
Blank PEG	T14	G09	0	0	kleiner LLOQ	#VALUE!	1	#VALUE!
Blank PEG	T14	G10	0	0	kleiner LLOQ	#VALUE!	1	#VALUE!
Blank PEG	T14	G11	0	0	kleiner LLOQ	#VALUE!	1	#VALUE!
Blank PEG	T14	G12	0	0	kleiner LLOQ	#VALUE!	1	#VALUE!
0	T14	H01	0	0	kleiner LLOQ	#VALUE!	1	#VALUE!
0	T14	H02	0	0	kleiner LLOQ	#VALUE!	1	#VALUE!
0	T14	H03	0	0	kleiner LLOQ	#VALUE!	1	#VALUE!
0	T14	H04	0	0	kleiner LLOQ	#VALUE!	1	#VALUE!
0	T14	H05	0	0	kleiner LLOQ	#VALUE!	1	#VALUE!
0	T14	H06	0	0	kleiner LLOQ	#VALUE!	1	#VALUE!
Blank no PEG	T14	H07	0	0	kleiner LLOQ	#VALUE!	1	#VALUE!
Blank no PEG	T14	H08	0	0	kleiner LLOQ	#VALUE!	1	#VALUE!
Blank no PEG	T14	H09	0	0	kleiner LLOQ	#VALUE!	1	#VALUE!
Blank no PEG	T14	H10	0	0	kleiner LLOQ	#VALUE!	1	#VALUE!
Blank no PEG	T14	H11	0	0	kleiner LLOQ	#VALUE!	1	#VALUE!
Blank no PEG	T14	H12	0	0	kleiner LLOQ	#VALUE!	1	#VALUE!

H							Blank no PEG	Blank no PEG	Blank no PEG	Blank no PEG	Blank no PEG	Blank no PEG

Comment:

alle Messwerte bei lin low

Date Operator

Date Control

1.447	1.739		1.170						0.667	0.440	0.043	1
1.447	1.332		1.392	0.137	0.146	0.130	0.138	0.109	0.729	0.478	0.042	
1.391	1.281		1.311	0.204	0.184	0.184	0.198	0.201	0.743	0.474	0.043	
0.961	0.835		1.405	0.336	0.347	0.375	0.387	0.300	0.824	0.483	0.044	
0.445	0.441		1.280	1.085	1.086	1.218	1.004	0.873	0.704	0.457	0.041	
0.355	0.315		1.332	1.768	1.925	1.753	1.739	1.606			0.036	
0.223	0.188	1.666	1.499	1.632		0.048	0.046	0.047	0.048	0.048	0.048	
0.118	0.106	1.556	1.622	1.573		0.047	0.054	0.051	0.051	0.052	0.051	

1.652	1.627		1.188	0.748	0.811	0.764	0.753	0.674	0.751	0.529	0.044	2
1.443	1.372		1.364	0.139	0.147	0.131	0.139	0.110	0.811	0.561	0.043	
1.377	1.281		1.284	0.206	0.189	0.184	0.200	0.202	0.821	0.560	0.044	
0.972	0.883		1.387	0.339	0.349	0.386	0.406	0.312	0.903	0.565	0.044	
0.484	0.474		1.298	1.087	1.113	1.203	1.022	0.903	0.790	0.544	0.042	
0.359	0.326		1.308	1.622	1.622	1.630	1.625	1.550			0.037	
0.228	0.199	1.527	1.373	1.462		0.048	0.046	0.048	0.049	0.049	0.049	
0.125	0.109	1.473	1.410	1.371		0.048	0.054	0.052	0.052	0.052	0.051	

1.579	1.475		1.150	1.210	1.303	1.202	1.219	1.125	0.759	0.549	0.043	3
1.357	1.303		1.283	0.140	0.148	0.131	0.139	0.111	0.820	0.575	0.043	
1.323	1.228		1.227	0.206	0.188	0.184	0.198	0.201	0.824	0.579	0.043	
0.959	0.872		1.321	0.339	0.348	0.387	0.406	0.314	0.909	0.580	0.044	
0.483	0.473		1.270	1.069	1.103	1.181	1.017	0.903	0.798	0.560	0.042	
0.360	0.326		1.270	1.497	1.448	1.496	1.494	1.449			0.037	
0.228	0.199	1.374	1.262	1.314		0.048	0.046	0.048	0.049	0.049	0.049	
0.125	0.110	1.346	1.262	1.253		0.048	0.054	0.052	0.052	0.052	0.051	

1.511	1.369			1.538	1.712	1.528	1.502	1.425			0.043	4
1.296	1.261										0.043	
1.272	1.193										0.043	
0.947	0.859										0.044	
0.481	0.471										0.042	
0.361	0.325										0.037	
0.229	0.199					0.048	0.046	0.048	0.049	0.049	0.049	
0.125	0.110					0.047	0.054	0.052	0.052	0.052	0.051	

1.493	1.317	1.678	1.110	1.621	1.807	1.618	1.584	1.508	0.765	0.597	0.043	5
1.259	1.238	1.850	1.197	0.139	0.150	0.132	0.139	0.111	0.821	0.588	0.043	
1.248	1.175	1.816	1.153	0.204	0.188	0.184	0.197	0.199	0.831	0.599	0.044	
0.943	0.859	1.970	1.231	0.338	0.357	0.386	0.406	0.316	0.916	0.595	0.044	
0.480	0.470	2.220	1.220	1.054	1.088	1.154	1.006	0.902	0.805	0.579	0.042	
0.359	0.325	2.149	1.211	1.395	1.324	1.348	1.345	1.317	0.037	0.037	0.037	
0.228	0.199	1.240	1.151	1.183	0.034	0.049	0.046	0.048	0.049	0.049	0.049	
0.125	0.110	1.223	1.132	1.146	0.038	0.048	0.054	0.052	0.052	0.052	0.051	

1.434	1.258		1.090	1.648	1.876	1.649	1.627	1.535				6
1.217	1.201		1.149									
1.205	1.143		1.109									
0.935	0.845		1.182									
0.477	0.469		1.181									
0.360	0.323		1.167									
0.229	0.200					0.049	0.047	0.048	0.049	0.049	0.049	
0.125	0.110					0.048	0.054	0.052	0.052	0.052	0.051	

1.013	0.810		0.668	0.808	0.940	0.720	0.798	0.687	0.904	0.845	0.043	7
0.709	0.827		0.703	0.142	0.156	0.136	0.137	0.116	0.990	0.846	0.042	
0.682	0.692		0.680	0.197	0.188	0.190	0.191	0.184	1.023	0.861	0.043	
0.507	0.519		0.740	0.316	0.264	0.364	0.377	0.312	1.111	0.870	0.043	
0.394	0.406		0.682	0.580	0.561	0.600	0.529	0.563	0.962	0.834	0.042	
0.333	0.322		0.661	0.995	0.930	0.848	0.832	0.797	0.037	0.037	0.037	
0.229	0.201	0.843	0.679	0.732		0.048	0.046	0.047			0.047	
0.124	0.111	0.773	0.682	0.701		0.047	0.053	0.051	0.051	0.052	0.051	

skin cultivation

0.462	0.469	0.446	0.350									T12 1 hum sui
0.210	0.164	0.206	0.204									
0.846	0.781	0.669	0.234									
	0.384	0.451	0.605									
0.660	0.298	0.139	0.235									
0.568	0.248	0.331	0.314									
						0.048	0.049	0.049	0.049	0.048	0.048	
						0.047	0.047	0.047	0.047	0.047	0.047	

				0.519	0.533	0.518	0.519	0.484	0.513	0.362	0.354	T12 2 hum sui
				0.206	0.207	0.164	0.166	0.196	0.182	0.199	0.199	
				0.835	0.831	0.740	0.760	0.624	0.633	0.245	0.234	
						0.392	0.404	0.444	0.467	0.574	0.587	
				0.636	0.622	0.342	0.371	0.439	0.463	0.228	0.226	
				0.481	0.487	0.294	0.226	0.298	0.299	0.306	0.308	
						0.049	0.049	0.050	0.049	0.049	0.049	
						0.047	0.047	0.047	0.048	0.048	0.047	

0.425	0.375	0.413	0.352									T13 1 hum sui
0.157	0.171	0.185	0.174									
0.885	0.650	0.522	0.254									
	0.490	0.430	0.632									
0.474	0.257	0.146	0.232									
0.497	0.247	0.286	0.298									
						0.049	0.049	0.049	0.050	0.050	0.049	
						0.048	0.047	0.049	0.048	0.047	0.048	

				0.454	0.424	0.350	0.386	0.444	0.434	0.382	0.382	T13 2 hum su
				0.158	0.201	0.183	0.182	0.159	0.156	0.184	0.190	
				0.933	0.873	0.744	0.758	0.539	0.581	0.256	0.289	
						0.554	0.540	0.464	0.471	0.680	0.708	
				0.472	0.430	0.233	0.247	0.137	0.142	0.228	0.235	
				0.320	0.258	0.191	0.220	0.250	0.250	0.294	0.305	
						0.050	0.050	0.053	0.051	0.051	0.050	
						0.048	0.048	0.048	0.048	0.048	0.048	

0.389	0.411	0.492	0.417									T14 1 hum sui
0.172	0.168	0.194	0.181									
0.877	0.727	0.696	0.348									
0.040	0.140	0.155	0.244									
0.511	0.320	0.164	0.243									
0.617	0.263	0.314	0.321									
						0.042	0.048	0.048	0.050	0.049	0.048	
						0.045	0.046	0.047	0.049	0.047	0.048	

				0.308	0.290	0.352	0.377	0.386	0.406	0.362	0.392	T14 2 hum sui
				0.183	0.180	0.174	0.178	0.170	0.174	0.187	0.256	
				0.667	0.663	0.572	0.566	0.541	0.553	0.416	0.389	
						0.467	0.518	0.607	0.573	0.510	0.574	
				0.448	0.454	0.290	0.288	0.177	0.183	0.311	0.279	
				0.471	0.511	0.236	0.261	0.259	0.349	0.350	0.224	
						0.041	0.048	0.048	0.048	0.048	0.047	
						0.044	0.045	0.046	0.048	0.046	0.048	

**Project number**		F-120	**Apparatus**	**Wallac Victor**	**Operator**	**IsBa**
**GLP Study (Number)**		n.a.	**Protocol (Instrument method)**	**LDH test 2016**	**Date of preparation**	**19-04-18**
**hot substance**	**isotope**	n.a.	**File name (results)**	**IsBa_180419/20_LDH_full_va**	**Date of measurement**	**19-04-18**
	**name**	n.a.	**Kind of well plate**	**96 well**	**shaking time [min]**	**30**
	**ACB-ID**	n.a.	**sample volume [µL]**	**100**	**stirring rate (Target) [rpm]**	**150**
	**Batch number**	n.a.	**Cocktail volume [µL]**	**175**	**Kind of measurement**	**UV-vis**
**cold substance**	**name**	LDH	**ACB-ID of cocktail**	n.a.	**Wave length [nm]**	**450**
	**ACB-ID**	L23982R	**Matrix**	**DMEM (from powder)+PEG**	**Remarks**	**Cocktail 100µl RM, 75µl STOP**
	**Batch number**	SLBK6345V	**Blank description**	**DMEM/PEG, H2O**	**Remarks**	**7 standards split low/high**
**n.a.**		n.a.	**Pipettes (No. / volume)**	**50-200µl**	**Remarks**	**KLP4 common for both**
**n.a.**		n.a.	**Pipettes (No. / volume)**	**n.a.**	**Remarks**	**n.a.**

Messdaten (diese Tabelle in Bericht übernehmen)

Sample name *	concentration (theor.) *	measured data	measured data	measured data	mean measured	SD	RSD	Blank *	measured data after * Blank subtraction	concentration (calc.) *	Deviation *	Residuen
	[µg/mL]	[AU]	[AU]	[AU]	[AU]	[AU]	[%]	[AU]		[µg/mL]	[%]	
KLP1								0.047				
KLP2								0.054				
KLP3								0.051				
KLP4	0.041	0.959	0.972	0.883	0.938	0.04	4.19	0.051	0.887	0.041	-0.57	0.00
KLP5	0.018	0.483	0.484	0.474	0.480	0.00	0.98	0.052	0.429	0.019	2.42	0.00
KLP6	0.012	0.360	0.359	0.326	0.348	0.02	4.54	0.051	0.297	0.013	5.00	0.00
KLP7	0.007	0.228	0.228	0.199	0.218	0.014	6.35		0.168	0.006	-11.29	0.00
KLP8												

Statistical data

Geradensteigung	Slope	m	21.03
Y-Achsenabschnitt	Y-Intercept	b	0.03
Standardabw. Geradensteigung	SD-Slope	s_m	0.648055501
Standardabw. Achsenabschnittes	SD-Y-Intercept	s_b	0.015177452
Anzahl Messpunkte	number of measuring points	n	4
Quadratsumme	sum of squares	Qxx	0.000664048
Bereichsmitte			0.019557193
Freiheitsgrade	degree of freedom	f	2
Student-t-Faktor für (P = 95 %; f = n-2)	Student-t-factor for (P = 95 %; f = n-2)	t	4.303
Vertrauensbereich Steig. (95 %) Obergrenze		$m + VB_m$	23.81763508
Vertrauensbereich Steig. (95 %) Untergrenze		$m - VB_m$	18.24046944
Vertrauensbereich Achsenabschnitt (95 %) Og.		$b + Vb_b$	0.099335829
Vertrauensbereich Achsenabschnitt (95 %) Ug.		$b - Vb_b$	-0.031281327
Korrelationskoeffizient	correlation coefficient	r	0.9991
Bestimmtheitsmaß	determiantion coefficient	r^2	0.9981
Reststandardabweichung		s_0	0.01669983
Summe Restquadrate		sd	0.657385838
Verfahrensstandardabw.		s_{x0}	0.000794131
Rel. Verfahrensstandardabw. %		V_{x0}	4.060559029

Berichten

mean Blank	**0**		
SD Blank	0.00		
RSD Blank	3.79	x=	
x*SD (LLOQ)	0.01		5
x*SD (LOD)	0.01		3
**LLOQ (AU)**	**0.061**		
**LOD (AU)**	**0.057**		
ULOQ	0.887		
LLOQ (Lin)	0.168		

Evaluation / Comment

LDH linearity valid with 7 standards, split in lin high and lin low, each with 4 standards and KLP4 common standard for both. R² 0,9981, deviations for both between -13.34% and +13.04%

Figure 1 Linearity

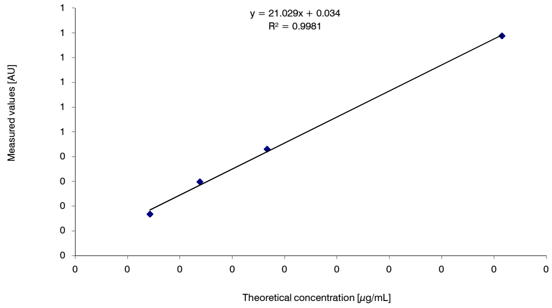

Figure 2 Method validation Residuen Plot

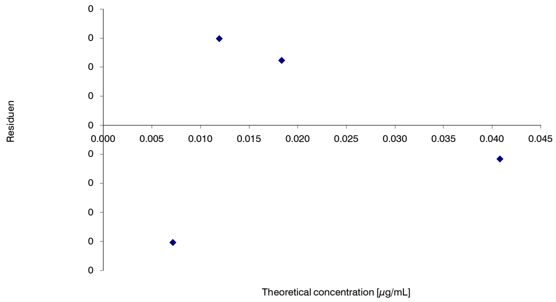

Evaluation / Comment

lin passed.

							Dilution	
Sample name	Plate	Position	[AU]	[AU]-Blank	Rating	[µg/mL]	factor	[µg/mL]
0	T0/T1	A01	0	0	kleiner LLOQ	#VALUE!	1	#VALUE!
0	T0/T1	A02	0	0	kleiner LLOQ	#VALUE!	1	#VALUE!
0	T0/T1	A03	0	0	kleiner LLOQ	#VALUE!	1	#VALUE!
0	T0/T1	A04	0	0	kleiner LLOQ	#VALUE!	1	#VALUE!
T0KltN1	T0/T1	A05	0.799	0.748	0.748	0.034	30.25	1.026
T0KltN1	T0/T1	A06	0.787	0.736	0.736	0.033	30.25	1.010
T0KltN2	T0/T1	A07	0	0	kleiner LLOQ	#VALUE!	1	#VALUE!
T0KltN2	T0/T1	A08	0	0	kleiner LLOQ	#VALUE!	1	#VALUE!
T0KltN3	T0/T1	A09	0	0	kleiner LLOQ	#VALUE!	1	#VALUE!
T0KltN3	T0/T1	A10	0	0	kleiner LLOQ	#VALUE!	1	#VALUE!
T0KltN4	T0/T1	A11	0.192	0.141	kleiner LLOQ	#VALUE!	2.75	#VALUE!
T0KltN4	T0/T1	A12	0.198	0.147	kleiner LLOQ	#VALUE!	2.75	#VALUE!
0	T0/T1	B01	0	0	kleiner LLOQ	#VALUE!	1	#VALUE!
0	T0/T1	B02	0	0	kleiner LLOQ	#VALUE!	1	#VALUE!
0	T0/T1	B03	0	0	kleiner LLOQ	#VALUE!	1	#VALUE!
0	T0/T1	B04	0	0	kleiner LLOQ	#VALUE!	1	#VALUE!
T0KltS11	T0/T1	B05	0	0	kleiner LLOQ	#VALUE!	1	#VALUE!
T0KltS11	T0/T1	B06	0	0	kleiner LLOQ	#VALUE!	1	#VALUE!
T0KltS12	T0/T1	B07	0.343	0.292	0.292	0.012	30.25	0.372
T0KltS12	T0/T1	B08	0.772	0.721	0.721	0.033	30.25	0.988
T0KltS13	T0/T1	B09	0.624	0.573	0.573	0.026	30.25	0.776
T0KltS13	T0/T1	B10	0.875	0.824	0.824	0.038	30.25	1.136
T0KltS14	T0/T1	B11	0.228	0.177	0.177	0.007	2.75	0.019
T0KltS14	T0/T1	B12	0.278	0.227	0.227	0.009	2.75	0.025
0	T0/T1	C01	0	0	kleiner LLOQ	#VALUE!	1	#VALUE!
0	T0/T1	C02	0	0	kleiner LLOQ	#VALUE!	1	#VALUE!
0	T0/T1	C03	0	0	kleiner LLOQ	#VALUE!	1	#VALUE!
0	T0/T1	C04	0	0	kleiner LLOQ	#VALUE!	1	#VALUE!
T0KltS21	T0/T1	C05	0.616	0.565	0.565	0.025	30.25	0.763
T0KltS21	T0/T1	C06	0.793	0.742	0.742	0.034	30.25	1.018
T0KltS22	T0/T1	C07	0	0	kleiner LLOQ	#VALUE!	1	#VALUE!
T0KltS22	T0/T1	C08	0	0	kleiner LLOQ	#VALUE!	1	#VALUE!
T0KltS23	T0/T1	C09	0	0	kleiner LLOQ	#VALUE!	1	#VALUE!
T0KltS23	T0/T1	C10	0	0	kleiner LLOQ	#VALUE!	1	#VALUE!
T0KltS24	T0/T1	C11	0.173	0.122	kleiner LLOQ	#VALUE!	2.75	#VALUE!
T0KltS24	T0/T1	C12	0.188	0.137	kleiner LLOQ	#VALUE!	2.75	#VALUE!
0	T0/T1	D01	0	0	kleiner LLOQ	#VALUE!	1	#VALUE!
0	T0/T1	D02	0	0	kleiner LLOQ	#VALUE!	1	#VALUE!
0	T0/T1	D03	0	0	kleiner LLOQ	#VALUE!	1	#VALUE!
0	T0/T1	D04	0	0	kleiner LLOQ	#VALUE!	1	#VALUE!
T1KltN1	T0/T1	D05	0.461	0.410	0.410	0.018	27.5	0.491
T1KltN1	T0/T1	D06	0.435	0.384	0.384	0.017	27.5	0.458
T1KltN2	T0/T1	D07	0.387	0.336	0.336	0.014	27.5	0.395
T1KltN2	T0/T1	D08	0.371	0.320	0.320	0.014	27.5	0.374
T1KltN3	T0/T1	D09	0.386	0.335	0.335	0.014	27.5	0.393
T1KltN3	T0/T1	D10	0.389	0.338	0.338	0.014	27.5	0.397
T1KltN4	T0/T1	D11	0.128	0.077	kleiner LLOQ	#VALUE!	2.75	#VALUE!
T1KltN4	T0/T1	D12	0.131	0.080	kleiner LLOQ	#VALUE!	2.75	#VALUE!

Plate:	T0/T1

	Test	Test	Test	Test								
	1	2	3	4	5	6	7	8	9	10	11	12
A					0.799	0.787					0.192	0.198
B							0.343	0.772	0.624	0.875	0.228	0.278
C					0.616	0.793					0.173	0.188
D					0.461	0.435	0.387	0.371	0.386	0.389	0.128	0.131
E					0.644	0.656	0.482	0.491	0.431	0.422	0.171	0.166
F					0.150	0.150	0.189	0.191	0.155	0.154	0.055	0.127
G							0.050	0.051	0.049	0.045	0.047	0.049
H							0.049	0.048	0.048	0.048	0.048	0.048

Plattenbelegung

	1	2	3	4	5	6	7	8	9	10	11	12
A					T0KltN1	T0KltN1	T0KltN2	T0KltN2	T0KltN3	T0KltN3	T0KltN4	T0KltN4
B					T0KltS11	T0KltS11	T0KltS12	T0KltS12	T0KltS13	T0KltS13	T0KltS14	T0KltS14
C					T0KltS21	T0KltS21	T0KltS22	T0KltS22	T0KltS23	T0KltS23	T0KltS24	T0KltS24
D					T1KltN1	T1KltN1	T1KltN2	T1KltN2	T1KltN3	T1KltN3	T1KltN4	T1KltN4
E					T1KltS11	T1KltS11	T1KltS12	T1KltS12	T1KltS13	T1KltS13	T1KltS14	T1KltS14
F					T1KltS21	T1KltS21	T1KltS22	T1KltS22	T1KltS23	T1KltS23	T1KltS24	T1KltS24
G							Blank PEG	Blank PEG	Blank PEG	Blank PEG	Blank PEG	Blank PEG
H							Blank no PEG	Blank no PEG	Blank no PEG	Blank no PEG	Blank no PEG	Blank no PEG

Samples aufgeteilt in lin high und low:höher konzentrierte Samples bei lin high zu finden.

T0 dil.11*dil2,75=dil.30,25

Comment: T1 dil.10*dil2,75=dil.27,5

Date Operator

Date Control

0	T0/T1	E01	0	0	kleiner LLOQ	#VALUE!	1	#VALUE!
0	T0/T1	E02	0	0	kleiner LLOQ	#VALUE!	1	#VALUE!
0	T0/T1	E03	0	0	kleiner LLOQ	#VALUE!	1	#VALUE!
0	T0/T1	E04	0	0	kleiner LLOQ	#VALUE!	1	#VALUE!
T1KltS11	T0/T1	E05	0.644	0.593	0.593	0.027	27.5	0.731
T1KltS11	T0/T1	E06	0.656	0.605	0.605	0.027	27.5	0.747
T1KltS12	T0/T1	E07	0.482	0.432	0.432	0.019	27.5	0.520
T1KltS12	T0/T1	E08	0.491	0.440	0.440	0.019	27.5	0.531
T1KltS13	T0/T1	E09	0.431	0.380	0.380	0.016	27.5	0.452
T1KltS13	T0/T1	E10	0.422	0.371	0.371	0.016	27.5	0.441
T1KltS14	T0/T1	E11	0.171	0.120	kleiner LLOQ	#VALUE!	2.75	#VALUE!
T1KltS14	T0/T1	E12	0.166	0.115	kleiner LLOQ	#VALUE!	2.75	#VALUE!
0	T0/T1	F01	0	0	kleiner LLOQ	#VALUE!	1	#VALUE!
0	T0/T1	F02	0	0	kleiner LLOQ	#VALUE!	1	#VALUE!
0	T0/T1	F03	0	0	kleiner LLOQ	#VALUE!	1	#VALUE!
0	T0/T1	F04	0	0	kleiner LLOQ	#VALUE!	1	#VALUE!
T1KltS21	T0/T1	F05	0.150	0.099	kleiner LLOQ	#VALUE!	27.5	#VALUE!
T1KltS21	T0/T1	F06	0.150	0.099	kleiner LLOQ	#VALUE!	27.5	#VALUE!
T1KltS22	T0/T1	F07	0.189	0.138	kleiner LLOQ	#VALUE!	27.5	#VALUE!
T1KltS22	T0/T1	F08	0.191	0.140	kleiner LLOQ	#VALUE!	27.5	#VALUE!
T1KltS23	T0/T1	F09	0.155	0.104	kleiner LLOQ	#VALUE!	27.5	#VALUE!
T1KltS23	T0/T1	F10	0.154	0.103	kleiner LLOQ	#VALUE!	27.5	#VALUE!
T1KltS24	T0/T1	F11	0.055	0.005	kleiner LLOQ	#VALUE!	2.75	#VALUE!
T1KltS24	T0/T1	F12	0.127	0.076	kleiner LLOQ	#VALUE!	2.75	#VALUE!
0	T0/T1	G01	0	0	kleiner LLOQ	#VALUE!	1	#VALUE!
0	T0/T1	G02	0	0	kleiner LLOQ	#VALUE!	1	#VALUE!
0	T0/T1	G03	0	0	kleiner LLOQ	#VALUE!	1	#VALUE!
0	T0/T1	G04	0	0	kleiner LLOQ	#VALUE!	1	#VALUE!
0	T0/T1	G05	0	0	kleiner LLOQ	#VALUE!	1	#VALUE!
0	T0/T1	G06	0	0	kleiner LLOQ	#VALUE!	1	#VALUE!
Blank PEG	T0/T1	G07	0	0	kleiner LLOQ	#VALUE!	2.75	#VALUE!
Blank PEG	T0/T1	G08	0	0	kleiner LLOQ	#VALUE!	2.75	#VALUE!
Blank PEG	T0/T1	G09	0	0	kleiner LLOQ	#VALUE!	2.75	#VALUE!
Blank PEG	T0/T1	G10	0	0	kleiner LLOQ	#VALUE!	2.75	#VALUE!
Blank PEG	T0/T1	G11	0	0	kleiner LLOQ	#VALUE!	2.75	#VALUE!
Blank PEG	T0/T1	G12	0	0	kleiner LLOQ	#VALUE!	2.75	#VALUE!
0	T0/T1	H01	0	0	kleiner LLOQ	#VALUE!	1	#VALUE!
0	T0/T1	H02	0	0	kleiner LLOQ	#VALUE!	1	#VALUE!
0	T0/T1	H03	0	0	kleiner LLOQ	#VALUE!	1	#VALUE!
0	T0/T1	H04	0	0	kleiner LLOQ	#VALUE!	1	#VALUE!
0	T0/T1	H05	0	0	kleiner LLOQ	#VALUE!	1	#VALUE!
0	T0/T1	H06	0	0	kleiner LLOQ	#VALUE!	1	#VALUE!
Blank no PEG	T0/T1	H07	0	0	kleiner LLOQ	#VALUE!	2.75	#VALUE!
Blank no PEG	T0/T1	H08	0	0	kleiner LLOQ	#VALUE!	2.75	#VALUE!
Blank no PEG	T0/T1	H09	0	0	kleiner LLOQ	#VALUE!	2.75	#VALUE!
Blank no PEG	T0/T1	H10	0	0	kleiner LLOQ	#VALUE!	2.75	#VALUE!
Blank no PEG	T0/T1	H11	0	0	kleiner LLOQ	#VALUE!	2.75	#VALUE!
Blank no PEG	T0/T1	H12	0	0	kleiner LLOQ	#VALUE!	2.75	#VALUE!

0	T2/T3	A01	0	0	kleiner LLOQ	#VALUE!	1	#VALUE!
0	T2/T3	A02	0	0	kleiner LLOQ	#VALUE!	1	#VALUE!
0	T2/T3	A03	0	0	kleiner LLOQ	#VALUE!	1	#VALUE!
0	T2/T3	A04	0	0	kleiner LLOQ	#VALUE!	1	#VALUE!
T2KltN1	T2/T3	A05	1	0.455	0.455	0.020	24.75	0.496
T2KltN1	T2/T3	A06	1	0.468	0.468	0.021	24.75	0.510
T2KltN2	T2/T3	A07	0	0.425	0.425	0.019	24.75	0.460
T2KltN2	T2/T3	A08	0	0.441	0.441	0.019	24.75	0.479
T2KltN3	T2/T3	A09	0	0.437	0.437	0.019	24.75	0.475
T2KltN3	T2/T3	A10	0	0.443	0.443	0.019	24.75	0.481
T2KltN4	T2/T3	A11	0	0.042	kleiner LLOQ	#VALUE!	2.75	#VALUE!
T2KltN4	T2/T3	A12	0	0.028	kleiner LLOQ	#VALUE!	2.75	#VALUE!
0	T2/T3	B01	0	0	kleiner LLOQ	#VALUE!	1	#VALUE!
0	T2/T3	B02	0	0	kleiner LLOQ	#VALUE!	1	#VALUE!
0	T2/T3	B03	0	0	kleiner LLOQ	#VALUE!	1	#VALUE!
0	T2/T3	B04	0	0	kleiner LLOQ	#VALUE!	1	#VALUE!
T2KltS11	T2/T3	B05	0.618	0.567	0.567	0.025	24.75	0.628
T2KltS11	T2/T3	B06	0.641	0.590	0.590	0.026	24.75	0.655
T2KltS12	T2/T3	B07	0.569	0.518	0.518	0.023	24.75	0.569
T2KltS12	T2/T3	B08	0.559	0.508	0.508	0.023	24.75	0.558
T2KltS13	T2/T3	B09	0.545	0.494	0.494	0.022	24.75	0.541
T2KltS13	T2/T3	B10	0.530	0.479	0.479	0.021	24.75	0.524
T2KltS14	T2/T3	B11	0.074	0.023	kleiner LLOQ	#VALUE!	2.75	#VALUE!
T2KltS14	T2/T3	B12	0.075	0.024	kleiner LLOQ	#VALUE!	2.75	#VALUE!
0	T2/T3	C01	0	0	kleiner LLOQ	#VALUE!	1	#VALUE!
0	T2/T3	C02	0	0	kleiner LLOQ	#VALUE!	1	#VALUE!
0	T2/T3	C03	0	0	kleiner LLOQ	#VALUE!	1	#VALUE!
0	T2/T3	C04	0	0	kleiner LLOQ	#VALUE!	1	#VALUE!
T2KltS21	T2/T3	C05	0.509	0.458	0.458	0.020	24.75	0.499
T2KltS21	T2/T3	C06	0.518	0.467	0.467	0.021	24.75	0.510
T2KltS22	T2/T3	C07	0.600	0.550	0.550	0.025	24.75	0.607
T2KltS22	T2/T3	C08	0.610	0.560	0.560	0.025	24.75	0.618
T2KltS23	T2/T3	C09	0.451	0.400	0.400	0.017	24.75	0.430
T2KltS23	T2/T3	C10	0.451	0.400	0.400	0.017	24.75	0.431
T2KltS24	T2/T3	C11	0.079	0.028	kleiner LLOQ	#VALUE!	2.75	#VALUE!
T2KltS24	T2/T3	C12	0.079	0.028	kleiner LLOQ	#VALUE!	2.75	#VALUE!
0	T2/T3	D01	0	0	kleiner LLOQ	#VALUE!	1	#VALUE!
0	T2/T3	D02	0	0	kleiner LLOQ	#VALUE!	1	#VALUE!
0	T2/T3	D03	0	0	kleiner LLOQ	#VALUE!	1	#VALUE!
0	T2/T3	D04	0	0	kleiner LLOQ	#VALUE!	1	#VALUE!
T3KltN1	T2/T3	D05	0.374	0.324	0.324	0.014	16.5	0.227
T3KltN1	T2/T3	D06	0.374	0.323	0.323	0.014	16.5	0.227
T3KltN2	T2/T3	D07	0.407	0.356	0.356	0.015	16.5	0.253
T3KltN2	T2/T3	D08	0.402	0.351	0.351	0.015	16.5	0.249
T3KltN3	T2/T3	D09	0.280	0.229	0.229	0.009	16.5	0.153
T3KltN3	T2/T3	D10	0.279	0.228	0.228	0.009	16.5	0.152
T3KltN4	T2/T3	D11	0.065	0.014	kleiner LLOQ	#VALUE!	2.75	#VALUE!
T3KltN4	T2/T3	D12	0.066	0.015	kleiner LLOQ	#VALUE!	2.75	#VALUE!

Plate: T2/T3

	1	2	3	4	5	6	7	8	9	10	11	12
A					0.506	0.519	0.476	0.492	0.488	0.493	0.093	0.079
B					0.618	0.641	0.569	0.559	0.545	0.530	0.074	0.075
C					0.509	0.518	0.600	0.610	0.451	0.451	0.079	0.079
D					0.374	0.374	0.407	0.402	0.280	0.279	0.065	0.066
E					0.169	0.170	0.393	0.405	0.376	0.386		
F					0.293	0.279	0.365	0.371	0.193	0.200	0.062	0.061
G							0.050	0.050	0.048	0.050	0.050	0.050
H							0.050	0.049	0.051	0.050	0.050	0.050

Plattenbelegung

	1	2	3	4	5	6	7	8	9	10	11	12
A					T2KltN1	T2KltN1	T2KltN2	T2KltN2	T2KltN3	T2KltN3	T2KltN4	T2KltN4
B					T2KltS11	T2KltS11	T2KltS12	T2KltS12	T2KltS13	T2KltS13	T2KltS14	T2KltS14
C					T2KltS21	T2KltS21	T2KltS22	T2KltS22	T2KltS23	T2KltS23	T2KltS24	T2KltS24
D					T3KltN1	T3KltN1	T3KltN2	T3KltN2	T3KltN3	T3KltN3	T3KltN4	T3KltN4
E					T3KltS11	T3KltS11	T3KltS12	T3KltS12	T3KltS13	T3KltS13		
F					T3KltS21	T3KltS21	T3KltS22	T3KltS22	T3KltS23	T3KltS23	T3KltS24	T3KltS24
G							Blank PEG	Blank PEG	Blank PEG	Blank PEG	Blank PEG	Blank PEG
H							Blank no PEG	Blank no PEG	Blank no PEG	Blank no PEG	Blank no PEG	Blank no PEG

T2 dil.9*dil.2,75=dil.24,75

T3 dil.6*dil.2,75=dil.16,5

Comment:

Probe von T3KltS14 fehlt, daher nicht gemessen

Date Operator

Date Control

0	T2/T3	E01	0	0	kleiner LLOQ	#VALUE!	1	#VALUE!
0	T2/T3	E02	0	0	kleiner LLOQ	#VALUE!	1	#VALUE!
0	T2/T3	E03	0	0	kleiner LLOQ	#VALUE!	1	#VALUE!
0	T2/T3	E04	0	0	kleiner LLOQ	#VALUE!	1	#VALUE!
T3KltS11	T2/T3	E05	0.169	0.118	kleiner LLOQ	#VALUE!	16.5	#VALUE!
T3KltS11	T2/T3	E06	0.170	0.119	kleiner LLOQ	#VALUE!	16.5	#VALUE!
T3KltS12	T2/T3	E07	0.393	0.342	0.342	0.015	16.5	0.242
T3KltS12	T2/T3	E08	0.405	0.354	0.354	0.015	16.5	0.251
T3KltS13	T2/T3	E09	0.376	0.325	0.325	0.014	16.5	0.228
T3KltS13	T2/T3	E10	0.386	0.335	0.335	0.014	16.5	0.237
0	T2/T3	E11	0	0	kleiner LLOQ	#VALUE!	1	#VALUE!
0	T2/T3	E12		#VALUE!	#VALUE!	#VALUE!	1	#VALUE!
0	T2/T3	F01	0	0	kleiner LLOQ	#VALUE!	1	#VALUE!
0	T2/T3	F02	0	0	kleiner LLOQ	#VALUE!	1	#VALUE!
0	T2/T3	F03	0	0	kleiner LLOQ	#VALUE!	1	#VALUE!
0	T2/T3	F04	0	0	kleiner LLOQ	#VALUE!	1	#VALUE!
T3KltS21	T2/T3	F05	0.293	0.242	0.242	0.010	16.5	0.163
T3KltS21	T2/T3	F06	0.279	0.228	0.228	0.009	16.5	0.152
T3KltS22	T2/T3	F07	0.365	0.314	0.314	0.013	16.5	0.220
T3KltS22	T2/T3	F08	0.371	0.320	0.320	0.014	16.5	0.224
T3KltS23	T2/T3	F09	0.193	0.142	kleiner LLOQ	#VALUE!	16.5	#VALUE!
T3KltS23	T2/T3	F10	0.200	0.149	kleiner LLOQ	#VALUE!	16.5	#VALUE!
T3KltS24	T2/T3	F11	0.062	0.012	kleiner LLOQ	#VALUE!	2.75	#VALUE!
T3KltS24	T2/T3	F12	0.061	0.010	kleiner LLOQ	#VALUE!	2.75	#VALUE!
0	T2/T3	G01	0	0	kleiner LLOQ	#VALUE!	1	#VALUE!
0	T2/T3	G02	0	0	kleiner LLOQ	#VALUE!	1	#VALUE!
0	T2/T3	G03	0	0	kleiner LLOQ	#VALUE!	1	#VALUE!
0	T2/T3	G04	0	0	kleiner LLOQ	#VALUE!	1	#VALUE!
0	T2/T3	G05	0	0	kleiner LLOQ	#VALUE!	1	#VALUE!
0	T2/T3	G06	0	0	kleiner LLOQ	#VALUE!	1	#VALUE!
Blank PEG	T2/T3	G07	0	0	kleiner LLOQ	#VALUE!	2.75	#VALUE!
Blank PEG	T2/T3	G08	0	0	kleiner LLOQ	#VALUE!	2.75	#VALUE!
Blank PEG	T2/T3	G09	0	0	kleiner LLOQ	#VALUE!	2.75	#VALUE!
Blank PEG	T2/T3	G10	0	0	kleiner LLOQ	#VALUE!	2.75	#VALUE!
Blank PEG	T2/T3	G11	0	0	kleiner LLOQ	#VALUE!	2.75	#VALUE!
Blank PEG	T2/T3	G12	0	0	kleiner LLOQ	#VALUE!	2.75	#VALUE!
0	T2/T3	H01	0	0	kleiner LLOQ	#VALUE!	1	#VALUE!
0	T2/T3	H02	0	0	kleiner LLOQ	#VALUE!	1	#VALUE!
0	T2/T3	H03	0	0	kleiner LLOQ	#VALUE!	1	#VALUE!
0	T2/T3	H04	0	0	kleiner LLOQ	#VALUE!	1	#VALUE!
0	T2/T3	H05	0	0	kleiner LLOQ	#VALUE!	1	#VALUE!
0	T2/T3	H06	0	0	kleiner LLOQ	#VALUE!	1	#VALUE!
Blank no PEG	T2/T3	H07	0	0	kleiner LLOQ	#VALUE!	2.75	#VALUE!
Blank no PEG	T2/T3	H08	0	0	kleiner LLOQ	#VALUE!	2.75	#VALUE!
Blank no PEG	T2/T3	H09	0	0	kleiner LLOQ	#VALUE!	2.75	#VALUE!
Blank no PEG	T2/T3	H10	0	0	kleiner LLOQ	#VALUE!	2.75	#VALUE!
Blank no PEG	T2/T3	H11	0	0	kleiner LLOQ	#VALUE!	2.75	#VALUE!
Blank no PEG	T2/T3	H12	0	0	kleiner LLOQ	#VALUE!	2.75	#VALUE!

0	T4/T5	A01	0	0	kleiner LLOQ	#VALUE!	1	#VALUE!
0	T4/T5	A02	0	0	kleiner LLOQ	#VALUE!	1	#VALUE!
0	T4/T5	A03	0	0	kleiner LLOQ	#VALUE!	1	#VALUE!
0	T4/T5	A04	0	0	kleiner LLOQ	#VALUE!	1	#VALUE!
T4KltN1	T4/T5	A05	0.447	0.396	0.396	0.017	8.25	0.142
T4KltN1	T4/T5	A06	0.456	0.405	0.405	0.018	8.25	0.146
T4KltN2	T4/T5	A07	0.457	0.407	0.407	0.018	8.25	0.146
T4KltN2	T4/T5	A08	0.463	0.412	0.412	0.018	8.25	0.148
T4KltN3	T4/T5	A09	0.586	0.536	0.536	0.024	2.75	0.066
T4KltN3	T4/T5	A10	0.600	0.549	0.549	0.025	2.75	0.067
T4KltN4	T4/T5	A11	0.072	0.021	kleiner LLOQ	#VALUE!	2.75	#VALUE!
T4KltN4	T4/T5	A12	0.072	0.021	kleiner LLOQ	#VALUE!	2.75	#VALUE!
0	T4/T5	B01	0	0	kleiner LLOQ	#VALUE!	1	#VALUE!
0	T4/T5	B02	0	0	kleiner LLOQ	#VALUE!	1	#VALUE!
0	T4/T5	B03	0	0	kleiner LLOQ	#VALUE!	1	#VALUE!
0	T4/T5	B04	0	0	kleiner LLOQ	#VALUE!	1	#VALUE!
T4KltS11	T4/T5	B05	0.284	0.233	0.233	0.009	8.25	0.078
T4KltS11	T4/T5	B06	0.281	0.230	0.230	0.009	8.25	0.077
T4KltS12	T4/T5	B07	0.290	0.239	0.239	0.010	2.75	0.027
T4KltS12	T4/T5	B08	0.292	0.241	0.241	0.010	2.75	0.027
T4KltS13	T4/T5	B09	0.692	0.641	0.641	0.029	2.75	0.079
T4KltS13	T4/T5	B10	0.864	0.813	0.813	0.037	2.75	0.102
T4KltS14	T4/T5	B11	0.065	0.014	kleiner LLOQ	#VALUE!	2.75	#VALUE!
T4KltS14	T4/T5	B12	0.065	0.014	kleiner LLOQ	#VALUE!	2.75	#VALUE!
0	T4/T5	C01	0	0	kleiner LLOQ	#VALUE!	1	#VALUE!
0	T4/T5	C02	0	0	kleiner LLOQ	#VALUE!	1	#VALUE!
0	T4/T5	C03	0	0	kleiner LLOQ	#VALUE!	1	#VALUE!
0	T4/T5	C04	0	0	kleiner LLOQ	#VALUE!	1	#VALUE!
T4KltS21	T4/T5	C05	0.415	0.364	0.364	0.016	2.75	0.043
T4KltS21	T4/T5	C06	0.429	0.379	0.379	0.016	2.75	0.045
T4KltS22	T4/T5	C07	0.475	0.424	0.424	0.019	2.75	0.051
T4KltS22	T4/T5	C08	0.532	0.481	0.481	0.021	2.75	0.058
T4KltS23	T4/T5	C09	0.663	0.612	0.612	0.027	2.75	0.076
T4KltS23	T4/T5	C10	0.665	0.614	0.614	0.028	2.75	0.076
T4KltS24	T4/T5	C11	0.061	0.010	kleiner LLOQ	#VALUE!	2.75	#VALUE!
T4KltS24	T4/T5	C12	0.061	0.010	kleiner LLOQ	#VALUE!	2.75	#VALUE!
0	T4/T5	D01	0	0	kleiner LLOQ	#VALUE!	1	#VALUE!
0	T4/T5	D02	0	0	kleiner LLOQ	#VALUE!	1	#VALUE!
0	T4/T5	D03	0	0	kleiner LLOQ	#VALUE!	1	#VALUE!
0	T4/T5	D04	0	0	kleiner LLOQ	#VALUE!	1	#VALUE!
T5KltN1	T4/T5	D05	0	0.342	0.342	0.015	2.75	0.040
T5KltN1	T4/T5	D06	0	0.333	0.333	0.014	2.75	0.039
T5KltN2	T4/T5	D07	1	0.561	0.561	0.025	2.75	0.069
T5KltN2	T4/T5	D08	1	0.556	0.556	0.025	2.75	0.068
T5KltN3	T4/T5	D09	0	0.308	0.308	0.013	2.75	0.036

Plate: T4/T5

	1	2	3	4	5	6	7	8	9	10	11	12
A					0.447	0.456	0.457	0.463	0.586	0.600	0.072	0.072
B					0.284	0.281	0.290	0.292	0.692	0.864	0.065	0.065
C					0.415	0.429	0.475	0.532	0.663	0.665	0.061	0.061
D					0.393	0.384	0.612	0.607	0.359	0.425	0.059	0.058
E					0.184	0.278	0.529	0.715	0.218	0.232	0.059	0.059
F					0.496	0.489	0.103	0.201	0.106	0.120	0.054	0.054
G							0.050	0.050	0.051	0.050	0.050	0.050
H							0.050	0.050	0.050	0.048	0.049	0.049

Plattenbelegung

	1	2	3	4	5	6	7	8	9	10	11	12
A					T4KltN1	T4KltN1	T4KltN2	T4KltN2	T4KltN3	T4KltN3	T4KltN4	T4KltN4
B					T4KltS11	T4KltS11	T4KltS12	T4KltS12	T4KltS13	T4KltS13	T4KltS14	T4KltS14
C					T4KltS21	T4KltS21	T4KltS22	T4KltS22	T4KltS23	T4KltS23	T4KltS24	T4KltS24
D					T5KltN1	T5KltN1	T5KltN2	T5KltN2	T5KltN3	T5KltN3	T5KltN4	T5KltN4
E					T5KltS11	T5KltS11	T5KltS12	T5KltS12	T5KltS13	T5KltS13	T5KltS14	T5KltS14
F					T5KltS21	T5KltS21	T5KltS22	T5KltS22	T5KltS23	T5KltS23	T5KltS24	T5KltS24
G							Blank PEG	Blank PEG	Blank PEG	Blank PEG	Blank PEG	Blank PEG
H							Blank no PEG	Blank no PEG	Blank no PEG	Blank no PEG	Blank no PEG	Blank no PEG

dil.3*dil2,75=dil.8,25

Comment:

Date Operator

Date Control

T5KltN3	T4/T5	D10	0	0.374	0.374	0.016	2.75	0.045
T5KltN4	T4/T5	D11	0	0.008	kleiner LLOQ	#VALUE!	2.75	#VALUE!
T5KltN4	T4/T5	D12	0	0.007	kleiner LLOQ	#VALUE!	2.75	#VALUE!
0	T4/T5	E01	0	0	kleiner LLOQ	#VALUE!	1	#VALUE!
0	T4/T5	E02	0	0	kleiner LLOQ	#VALUE!	1	#VALUE!
0	T4/T5	E03	0	0	kleiner LLOQ	#VALUE!	1	#VALUE!
0	T4/T5	E04	0	0	kleiner LLOQ	#VALUE!	1	#VALUE!
T5KltS11	T4/T5	E05	0	0.133	kleiner LLOQ	#VALUE!	2.75	#VALUE!
T5KltS11	T4/T5	E06	0	0.227	0.227	0.009	2.75	0.025
T5KltS12	T4/T5	E07	1	0.478	0.478	0.021	2.75	0.058
T5KltS12	T4/T5	E08	1	0.664	0.664	0.030	2.75	0.082
T5KltS13	T4/T5	E09	0	0.167	kleiner LLOQ	#VALUE!	2.75	#VALUE!
T5KltS13	T4/T5	E10	0	0.181	0.181	0.007	2.75	0.019
T5KltS14	T4/T5	E11	0	0.008	kleiner LLOQ	#VALUE!	2.75	#VALUE!
T5KltS14	T4/T5	E12	0	0.008	kleiner LLOQ	#VALUE!	2.75	#VALUE!
0	T4/T5	F01	0	0	kleiner LLOQ	#VALUE!	1	#VALUE!
0	T4/T5	F02	0	0	kleiner LLOQ	#VALUE!	1	#VALUE!
0	T4/T5	F03	0	0	kleiner LLOQ	#VALUE!	1	#VALUE!
0	T4/T5	F04	0	0	kleiner LLOQ	#VALUE!	1	#VALUE!
T5KltS21	T4/T5	F05	0	0.445	0.445	0.020	2.75	0.054
T5KltS21	T4/T5	F06	0	0.438	0.438	0.019	2.75	0.053
T5KltS22	T4/T5	F07	0	0.052	kleiner LLOQ	#VALUE!	2.75	#VALUE!
T5KltS22	T4/T5	F08	0	0.150	kleiner LLOQ	#VALUE!	2.75	#VALUE!
T5KltS23	T4/T5	F09	0	0.055	kleiner LLOQ	#VALUE!	2.75	#VALUE!
T5KltS23	T4/T5	F10	0	0.069	kleiner LLOQ	#VALUE!	2.75	#VALUE!
T5KltS24	T4/T5	F11	0	0.003	kleiner LLOQ	#VALUE!	2.75	#VALUE!
T5KltS24	T4/T5	F12	0	0.003	kleiner LLOQ	#VALUE!	2.75	#VALUE!
0	T4/T5	G01	0	0	kleiner LLOQ	#VALUE!	1	#VALUE!
0	T4/T5	G02	0	0	kleiner LLOQ	#VALUE!	1	#VALUE!
0	T4/T5	G03	0	0	kleiner LLOQ	#VALUE!	1	#VALUE!
0	T4/T5	G04	0	0	kleiner LLOQ	#VALUE!	1	#VALUE!
0	T4/T5	G05	0	0	kleiner LLOQ	#VALUE!	1	#VALUE!
0	T4/T5	G06	0	0	kleiner LLOQ	#VALUE!	1	#VALUE!
Blank PEG	T4/T5	G07	0	0	kleiner LLOQ	#VALUE!	2.75	#VALUE!
Blank PEG	T4/T5	G08	0	0	kleiner LLOQ	#VALUE!	2.75	#VALUE!
Blank PEG	T4/T5	G09	0	0	kleiner LLOQ	#VALUE!	2.75	#VALUE!
Blank PEG	T4/T5	G10	0	0	kleiner LLOQ	#VALUE!	2.75	#VALUE!
Blank PEG	T4/T5	G11	0	0	kleiner LLOQ	#VALUE!	2.75	#VALUE!
Blank PEG	T4/T5	G12	0	0	kleiner LLOQ	#VALUE!	2.75	#VALUE!
0	T4/T5	H01	0	0	kleiner LLOQ	#VALUE!	1	#VALUE!
0	T4/T5	H02	0	0	kleiner LLOQ	#VALUE!	1	#VALUE!
0	T4/T5	H03	0	0	kleiner LLOQ	#VALUE!	1	#VALUE!
0	T4/T5	H04	0	0	kleiner LLOQ	#VALUE!	1	#VALUE!
0	T4/T5	H05	0	0	kleiner LLOQ	#VALUE!	1	#VALUE!
0	T4/T5	H06	0	0	kleiner LLOQ	#VALUE!	1	#VALUE!
Blank no PEG	T4/T5	H07	0	0	kleiner LLOQ	#VALUE!	2.75	#VALUE!
Blank no PEG	T4/T5	H08	0	0	kleiner LLOQ	#VALUE!	2.75	#VALUE!
Blank no PEG	T4/T5	H09	0	0	kleiner LLOQ	#VALUE!	2.75	#VALUE!
Blank no PEG	T4/T5	H10	0	0	kleiner LLOQ	#VALUE!	2.75	#VALUE!
Blank no PEG	T4/T5	H11	0	0	kleiner LLOQ	#VALUE!	2.75	#VALUE!
Blank no PEG	T4/T5	H12	0	0	kleiner LLOQ	#VALUE!	2.75	#VALUE!

0	T6/T7	A01	0	0	kleiner LLOQ	#VALUE!	1	#VALUE!
0	T6/T7	A02	0	0	kleiner LLOQ	#VALUE!	1	#VALUE!
0	T6/T7	A03	0	0	kleiner LLOQ	#VALUE!	1	#VALUE!
0	T6/T7	A04	0	0	kleiner LLOQ	#VALUE!	1	#VALUE!
T6KltN1	T6/T7	A05	1	0.461	0.461	0.020	2.75	0.056
T6KltN1	T6/T7	A06	0	0.417	0.417	0.018	2.75	0.050
T6KltN2	T6/T7	A07	0	0.438	0.438	0.019	2.75	0.053
T6KltN2	T6/T7	A08	1	0.496	0.496	0.022	2.75	0.060
T6KltN3	T6/T7	A09	0	0.141	kleiner LLOQ	#VALUE!	2.75	#VALUE!
T6KltN3	T6/T7	A10	0	0.154	kleiner LLOQ	#VALUE!	2.75	#VALUE!
T6KltN4	T6/T7	A11	0	0.012	kleiner LLOQ	#VALUE!	2.75	#VALUE!
T6KltN4	T6/T7	A12	0	0.019	kleiner LLOQ	#VALUE!	2.75	#VALUE!
0	T6/T7	B01	0	0	kleiner LLOQ	#VALUE!	1	#VALUE!
0	T6/T7	B02	0	0	kleiner LLOQ	#VALUE!	1	#VALUE!
0	T6/T7	B03	0	0	kleiner LLOQ	#VALUE!	1	#VALUE!
0	T6/T7	B04	0	0	kleiner LLOQ	#VALUE!	1	#VALUE!
T6KltS11	T6/T7	B05	0.154	0.103	kleiner LLOQ	#VALUE!	2.75	#VALUE!
T6KltS11	T6/T7	B06	0.226	0.175	0.175	0.007	2.75	0.018
T6KltS12	T6/T7	B07	0.460	0.409	0.409	0.018	2.75	0.049
T6KltS12	T6/T7	B08	0.263	0.212	0.212	0.008	2.75	0.023
T6KltS13	T6/T7	B09	0.214	0.163	kleiner LLOQ	#VALUE!	2.75	#VALUE!
T6KltS13	T6/T7	B10	0.401	0.350	0.350	0.015	2.75	0.041
T6KltS14	T6/T7	B11	0.060	0.009	kleiner LLOQ	#VALUE!	2.75	#VALUE!
T6KltS14	T6/T7	B12	0.059	0.009	kleiner LLOQ	#VALUE!	2.75	#VALUE!
0	T6/T7	C01	0	0	kleiner LLOQ	#VALUE!	1	#VALUE!
0	T6/T7	C02	0	0	kleiner LLOQ	#VALUE!	1	#VALUE!
0	T6/T7	C03	0	0	kleiner LLOQ	#VALUE!	1	#VALUE!
0	T6/T7	C04	0	0	kleiner LLOQ	#VALUE!	1	#VALUE!
T6KltS21	T6/T7	C05	0	0.345	0.345	0.015	2.75	0.041
T6KltS21	T6/T7	C06	0	0.360	0.360	0.015	2.75	0.043
T6KltS22	T6/T7	C07	0	0.115	kleiner LLOQ	#VALUE!	2.75	#VALUE!
T6KltS22	T6/T7	C08	0	0.121	kleiner LLOQ	#VALUE!	2.75	#VALUE!
T6KltS23	T6/T7	C09	0	0.077	kleiner LLOQ	#VALUE!	2.75	#VALUE!
T6KltS23	T6/T7	C10	0	0.084	kleiner LLOQ	#VALUE!	2.75	#VALUE!
T6KltS24	T6/T7	C11	0	0.005	kleiner LLOQ	#VALUE!	2.75	#VALUE!
T6KltS24	T6/T7	C12	0	0.009	kleiner LLOQ	#VALUE!	2.75	#VALUE!
0	T6/T7	D01	0	0	kleiner LLOQ	#VALUE!	1	#VALUE!
0	T6/T7	D02	0	0	kleiner LLOQ	#VALUE!	1	#VALUE!
0	T6/T7	D03	0	0	kleiner LLOQ	#VALUE!	1	#VALUE!
0	T6/T7	D04	0	0	kleiner LLOQ	#VALUE!	1	#VALUE!
T7KltN1	T6/T7	D05	0.443	0.392	0.392	0.017	2.75	0.047
T7KltN1	T6/T7	D06	0.450	0.399	0.399	0.017	2.75	0.048
T7KltN2	T6/T7	D07	0.369	0.319	0.319	0.014	2.75	0.037
T7KltN2	T6/T7	D08	0.418	0.367	0.367	0.016	2.75	0.043
T7KltN3	T6/T7	D09	0.111	0.060	kleiner LLOQ	#VALUE!	2.75	#VALUE!
T7KltN3	T6/T7	D10	0.107	0.056	kleiner LLOQ	#VALUE!	2.75	#VALUE!
T7KltN4	T6/T7	D11	0.057	0.006	kleiner LLOQ	#VALUE!	2.75	#VALUE!

Plate: T6/T7

	1	2	3	4	5	6	7	8	9	10	11	12
A					0.512	0.468	0.489	0.547	0.192	0.205	0.062	0.070
B					0.154	0.226	0.460	0.263	0.214	0.401	0.060	0.059
C					0.396	0.411	0.166	0.172	0.128	0.135	0.056	0.060
D					0.443	0.450	0.369	0.418	0.111	0.107	0.057	0.060
E					0.064	0.062	0.091	0.158	0.098	0.156	0.057	0.058
F					0.243	0.304	0.097	0.142	0.139	0.137	0.055	0.054
G							0.049	0.052	0.052	0.051	0.052	0.051
H							0.050	0.050	0.050	0.045	0.051	0.050

Plattenbelegung

	1	2	3	4	5	6	7	8	9	10	11	12
A					T6KltN1	T6KltN1	T6KltN2	T6KltN2	T6KltN3	T6KltN3	T6KltN4	T6KltN4
B					T6KltS11	T6KltS11	T6KltS12	T6KltS12	T6KltS13	T6KltS13	T6KltS14	T6KltS14
C					T6KltS21	T6KltS21	T6KltS22	T6KltS22	T6KltS23	T6KltS23	T6KltS24	T6KltS24
D					T7KltN1	T7KltN1	T7KltN2	T7KltN2	T7KltN3	T7KltN3	T7KltN4	T7KltN4
E					T7KltS11	T7KltS11	T7KltS12	T7KltS12	T7KltS13	T7KltS13	T7KltS14	T7KltS14
F					T7KltS21	T7KltS21	T7KltS22	T7KltS22	T7KltS23	T7KltS23	T7KltS24	T7KltS24
G							Blank PEG	Blank PEG	Blank PEG	Blank PEG	Blank PEG	Blank PEG
H							Blank no PEG	Blank no PEG	Blank no PEG	Blank no PEG	Blank no PEG	Blank no PEG

Comment:

Date Operator

Date Control

T7KltN4	T6/T7	D12	0.060	0.009	kleiner LLOQ	#VALUE!	2.75	#VALUE!
0	T6/T7	E01	0	0	kleiner LLOQ	#VALUE!	1	#VALUE!
0	T6/T7	E02	0	0	kleiner LLOQ	#VALUE!	1	#VALUE!
0	T6/T7	E03	0	0	kleiner LLOQ	#VALUE!	1	#VALUE!
0	T6/T7	E04	0	0	kleiner LLOQ	#VALUE!	1	#VALUE!
T7KltS11	T6/T7	E05	0.06	0.01	kleiner LLOQ	#VALUE!	2.75	#VALUE!
T7KltS11	T6/T7	E06	0.06	0.01	kleiner LLOQ	#VALUE!	2.75	#VALUE!
T7KltS12	T6/T7	E07	0.09	0.04	kleiner LLOQ	#VALUE!	2.75	#VALUE!
T7KltS12	T6/T7	E08	0.16	0.11	kleiner LLOQ	#VALUE!	2.75	#VALUE!
T7KltS13	T6/T7	E09	0.10	0.05	kleiner LLOQ	#VALUE!	2.75	#VALUE!
T7KltS13	T6/T7	E10	0.16	0.10	kleiner LLOQ	#VALUE!	2.75	#VALUE!
T7KltS14	T6/T7	E11	0.06	0.01	kleiner LLOQ	#VALUE!	2.75	#VALUE!
T7KltS14	T6/T7	E12	0.06	0.01	kleiner LLOQ	#VALUE!	2.75	#VALUE!
0	T6/T7	F01	0	0	kleiner LLOQ	#VALUE!	1	#VALUE!
0	T6/T7	F02	0	0	kleiner LLOQ	#VALUE!	1	#VALUE!
0	T6/T7	F03	0	0	kleiner LLOQ	#VALUE!	1	#VALUE!
0	T6/T7	F04	0	0	kleiner LLOQ	#VALUE!	1	#VALUE!
T7KltS21	T6/T7	F05	0.243	0.193	0.193	0.008	2.75	0.021
T7KltS21	T6/T7	F06	0.304	0.253	0.253	0.010	2.75	0.029
T7KltS22	T6/T7	F07	0.097	0.046	kleiner LLOQ	#VALUE!	2.75	#VALUE!
T7KltS22	T6/T7	F08	0.142	0.091	kleiner LLOQ	#VALUE!	2.75	#VALUE!
T7KltS23	T6/T7	F09	0.139	0.088	kleiner LLOQ	#VALUE!	2.75	#VALUE!
T7KltS23	T6/T7	F10	0.137	0.086	kleiner LLOQ	#VALUE!	2.75	#VALUE!
T7KltS24	T6/T7	F11	0.055	0.004	kleiner LLOQ	#VALUE!	2.75	#VALUE!
T7KltS24	T6/T7	F12	0.054	0.003	kleiner LLOQ	#VALUE!	2.75	#VALUE!
0	T6/T7	G01	0	0	kleiner LLOQ	#VALUE!	1	#VALUE!
0	T6/T7	G02	0	0	kleiner LLOQ	#VALUE!	1	#VALUE!
0	T6/T7	G03	0	0	kleiner LLOQ	#VALUE!	1	#VALUE!
0	T6/T7	G04	0	0	kleiner LLOQ	#VALUE!	1	#VALUE!
0	T6/T7	G05	0	0	kleiner LLOQ	#VALUE!	1	#VALUE!
0	T6/T7	G06	0	0	kleiner LLOQ	#VALUE!	1	#VALUE!
Blank PEG	T6/T7	G07	0	0	kleiner LLOQ	#VALUE!	2.75	#VALUE!
Blank PEG	T6/T7	G08	0	0	kleiner LLOQ	#VALUE!	2.75	#VALUE!
Blank PEG	T6/T7	G09	0	0	kleiner LLOQ	#VALUE!	2.75	#VALUE!
Blank PEG	T6/T7	G10	0	0	kleiner LLOQ	#VALUE!	2.75	#VALUE!
Blank PEG	T6/T7	G11	0	0	kleiner LLOQ	#VALUE!	2.75	#VALUE!
Blank PEG	T6/T7	G12	0	0	kleiner LLOQ	#VALUE!	2.75	#VALUE!
0	T6/T7	H01	0	0	kleiner LLOQ	#VALUE!	1	#VALUE!
0	T6/T7	H02	0	0	kleiner LLOQ	#VALUE!	1	#VALUE!
0	T6/T7	H03	0	0	kleiner LLOQ	#VALUE!	1	#VALUE!
0	T6/T7	H04	0	0	kleiner LLOQ	#VALUE!	1	#VALUE!
0	T6/T7	H05	0	0	kleiner LLOQ	#VALUE!	1	#VALUE!
0	T6/T7	H06	0	0	kleiner LLOQ	#VALUE!	1	#VALUE!
Blank no PEG	T6/T7	H07	0	0	kleiner LLOQ	#VALUE!	2.75	#VALUE!
Blank no PEG	T6/T7	H08	0	0	kleiner LLOQ	#VALUE!	2.75	#VALUE!
Blank no PEG	T6/T7	H09	0	0	kleiner LLOQ	#VALUE!	2.75	#VALUE!
Blank no PEG	T6/T7	H10	0	0	kleiner LLOQ	#VALUE!	2.75	#VALUE!
Blank no PEG	T6/T7	H11	0	0	kleiner LLOQ	#VALUE!	2.75	#VALUE!
Blank no PEG	T6/T7	H12	0	0	kleiner LLOQ	#VALUE!	2.75	#VALUE!

**Project number**		F-120	**Apparatus**	**Wallac Victor**	**Operator**	**IsBa**
**GLP Study (Number)**		n.a.	**Protocol (Instrument meth**	**LDH test 2016**	**Date of preparation**	**19-04-18**
**hot substance**	**isotope**	n.a.	**File name (results)**	**IsBa_180419/20_LDH_full_**	**Date of measurement**	**19-04-18**
	**name**	n.a.	**Kind of well plate**	**96 well**	**shaking time [min]**	**30**
	**ACB-ID**	n.a.	**sample volume [µL]**	**100**	**stirring rate (Target) [rpm]**	**150**
	**Batch number**	n.a.	**Cocktail volume [µL]**	**175**	**Kind of measurement**	**UV-vis**
**cold substance**	**name**	LDH	**ACB-ID of cocktail**		**Wave length [nm]**	**450**
	**ACB-ID**	L23982R	**Matrix**	**DMEM (from powder)+PE(**	**Remarks**	**Cocktail 100µl RM, 75µl STOP**
	**Batch number**	SLBK6345V	**Blank description**	**DMEM/PEG, H2O**	**Remarks**	**7 standards split low/high**
**n.a.**		n.a.	**Pipettes (No. / volume)**	**50-200µl**	**Remarks**	**KLP4 common for both**
**n.a.**		n.a.	**Pipettes (No. / volume)**	**n.a.**	**Remarks**	**n.a.**

Messdaten (diese Tabelle in Bericht übernehmen)

Sample name *	concentration (theor.) *	measured data	measured data	measured data	mean measured	SD	RSD	Blank *	measured data after * Blank subtraction	concentration (calc.) *	Deviation *	Residuen
	[µg/mL]	[AU]	[AU]	[AU]	[AU]	[AU]	[%]	[AU]		[µg/mL]	[%]	
KLP1	0.148	1.579	1.652	1.627	1.62	0.03	1.87	0.047	1.568	0.148	-0.24	0.00
KLP2	0.114	1.357	1.443	1.372	1.39	0.04	2.70	0.054	1.340	0.110	-3.40	0.00
KLP3	0.074	1.272	1.193	1.228	1.23	0.03	2.63	0.051	1.180	0.084	13.04	0.01
KLP4	0.041	0.959	0.972	0.883	0.94	0.04	4.19	0.051	0.887	0.035	-13.32	-0.01
KLP5								0.052				
KLP6								0.051				
KLP7												
KLP8												

Statistical data

Geradensteigung	Slope	m	6.05
Y-Achsenabschnitt	Y-Intercept	b	0.67
Standardabw. Geradensteigung	SD-Slope	s_m	0.620291227
Standardabw. Achsenabschnittes	SD-Y-Intercept	s_b	0.063694294
Anzahl Messpunkte	number of measuring points	n	4
Quadratsumme	sum of squares	Qxx	0.006578604
Bereichsmitte			0.094336926
Freiheitsgrade	degree of freedom	f	2
Student-t-Faktor für (P = 95 %; f = n-2)	Student-t-factor for (P = 95 %; f = n-2)	t	4.303
Vertrauensbereich Steig. (95 %) Obergrenze		$m + VB_m$	8.722319527
Vertrauensbereich Steig. (95 %) Untergrenze		$m - VB_m$	3.384093228
Vertrauensbereich Achsenabschnitt (95 %) Og.		$b + Vb_b$	0.946914615
Vertrauensbereich Achsenabschnitt (95 %) Ug.		$b - Vb_b$	0.398761518
Korrelationskoeffizient	correlation coefficient	r	0.9897
Bestimmtheitsmaß	determiantion coefficient	r^2	0.9794
Reststandardabweichung		s_0	0.050310948
Summe Restquadrate		sd	2.895583345
Verfahrensstandardabw.		s_{x0}	0.008311454
Rel. Verfahrensstandardabw. %		V_{x0}	8.810393374

mean Blank	0		
SD Blank	0.00		
RSD Blank	3.79	x=	
x*SD (LLOQ)	0		5
x*SD (LOD)	0		3
**LLOQ (AU)**	**0**		
**LOD (AU)**	**0**		
ULOQ	1.568		
LLOQ (Lin)	0.887		

Evaluation / Comment

LDH linearity valid with 7 standards, split in lin high and lin low, each with 4 standards and KLP4 common standard for both. R² 0,9981, deviations for both between -13.34% and +13.04%

Date	Operator	Date	Control

Figure 1 **Linearity**

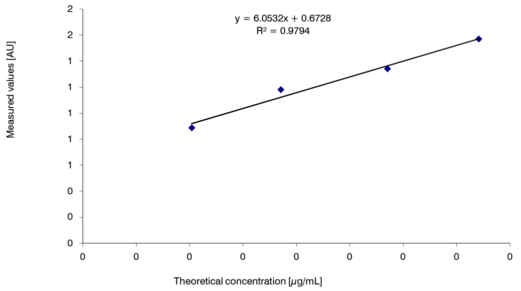

Figure 2 **Method validation Residuen Plot**

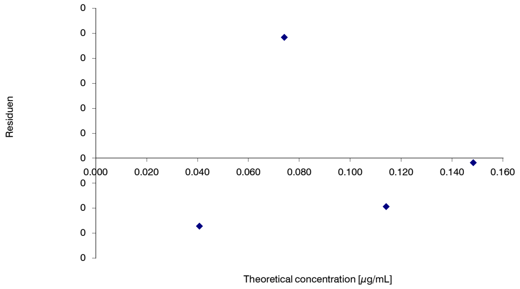

Evaluation / Comment

lin passed.

Date	Operator	Date	Control

Sample name	Plate	Position	[AU]	[AU]-Blank	Rating	[µg/mL]	Dilution factor	[µg/mL]
0	T0/T1	A01	0	0	kleiner LLOQ	#VALUE!	1	#VALUE!
0	T0/T1	A02	0	0	kleiner LLOQ	#VALUE!	1	#VALUE!
0	T0/T1	A03	0	0	kleiner LLOQ	#VALUE!	1	#VALUE!
0	T0/T1	A04	0	0	kleiner LLOQ	#VALUE!	1	#VALUE!
T0KItN1	T0/T1	A05	0	0	kleiner LLOQ	#VALUE!	1	#VALUE!
T0KItN1	T0/T1	A06	0	0	kleiner LLOQ	#VALUE!	1	#VALUE!
T0KItN2	T0/T1	A07	0.982	0.931	0.931	0.043	30.25	1.289
T0KItN2	T0/T1	A08	0.987	0.936	0.936	0.043	30.25	1.314
T0KItN3	T0/T1	A09	0.954	0.903	0.903	0.038	30.25	1.149
T0KItN3	T0/T1	A10	1.024	0.974	0.974	0.050	30.25	1.503
T0KItN4	T0/T1	A11	0	0	kleiner LLOQ	#VALUE!	1	#VALUE!
T0KItN4	T0/T1	A12	0	0	kleiner LLOQ	#VALUE!	1	#VALUE!
0	T0/T1	B01	0	0	kleiner LLOQ	#VALUE!	1	#VALUE!
0	T0/T1	B02	0	0	kleiner LLOQ	#VALUE!	1	#VALUE!
0	T0/T1	B03	0	0	kleiner LLOQ	#VALUE!	1	#VALUE!
0	T0/T1	B04	0	0	kleiner LLOQ	#VALUE!	1	#VALUE!
T0KItS11	T0/T1	B05	1.344	1.293	1.293	0.102	30.25	3.100
T0KItS11	T0/T1	B06	1.340	1.289	1.289	0.102	30.25	3.078
T0KItS12	T0/T1	B07	0	0	kleiner LLOQ	#VALUE!	1	#VALUE!
T0KItS12	T0/T1	B08	0	0	kleiner LLOQ	#VALUE!	1	#VALUE!
T0KItS13	T0/T1	B09	0	0	kleiner LLOQ	#VALUE!	1	#VALUE!
T0KItS13	T0/T1	B10	0	0	kleiner LLOQ	#VALUE!	1	#VALUE!
T0KItS14	T0/T1	B11	0	0	kleiner LLOQ	#VALUE!	1	#VALUE!
T0KItS14	T0/T1	B12	0	0	kleiner LLOQ	#VALUE!	1	#VALUE!
0	T0/T1	C01	0	0	kleiner LLOQ	#VALUE!	1	#VALUE!
0	T0/T1	C02	0	0	kleiner LLOQ	#VALUE!	1	#VALUE!
0	T0/T1	C03	0	0	kleiner LLOQ	#VALUE!	1	#VALUE!
0	T0/T1	C04	0	0	kleiner LLOQ	#VALUE!	1	#VALUE!
T0KItS21	T0/T1	C05	0	0	kleiner LLOQ	#VALUE!	1	#VALUE!
T0KItS21	T0/T1	C06	0	0	kleiner LLOQ	#VALUE!	1	#VALUE!
T0KItS22	T0/T1	C07	1.173	1.122	1.122	0.074	30.25	2.244
T0KItS22	T0/T1	C08	1.385	1.334	1.334	0.109	30.25	3.303
T0KItS23	T0/T1	C09	0.942	0.891	0.891	0.036	30.25	1.092
T0KItS23	T0/T1	C10	0.950	0.899	0.899	0.037	30.25	1.132
T0KItS24	T0/T1	C11	0	0	kleiner LLOQ	#VALUE!	1	#VALUE!
T0KItS24	T0/T1	C12	0	0	kleiner LLOQ	#VALUE!	1	#VALUE!
0	T0/T1	D01	0	0	kleiner LLOQ	#VALUE!	1	#VALUE!
0	T0/T1	D02	0	0	kleiner LLOQ	#VALUE!	1	#VALUE!
0	T0/T1	D03	0	0	kleiner LLOQ	#VALUE!	1	#VALUE!
0	T0/T1	D04	0	0	kleiner LLOQ	#VALUE!	1	#VALUE!
T1KItN1	T0/T1	D05	0	0	kleiner LLOQ	#VALUE!	1	#VALUE!
T1KItN1	T0/T1	D06	0	0	kleiner LLOQ	#VALUE!	1	#VALUE!
T1KItN2	T0/T1	D07	0	0	kleiner LLOQ	#VALUE!	1	#VALUE!
T1KItN2	T0/T1	D08	0	0	kleiner LLOQ	#VALUE!	1	#VALUE!
T1KItN3	T0/T1	D09	0	0	kleiner LLOQ	#VALUE!	1	#VALUE!
T1KItN3	T0/T1	D10	0	0	kleiner LLOQ	#VALUE!	1	#VALUE!
T1KItN4	T0/T1	D11	0	0	kleiner LLOQ	#VALUE!	1	#VALUE!

Plate: T0/T1

	1	2	3	4	5	6	7	8	9	10	11	12
A							0.982	0.987	0.954	1.024		
B					1.344	1.340						
C							1.173	1.385	0.942	0.950		
D												
E												
F												
G							0.050	0.051	0.049	0.045	0.047	0.049
H							0.049	0.048	0.048	0.048	0.048	0.048

Plattenbelegung

	1	2	3	4	5	6	7	8	9	10	11	12
A					T0KItN1	T0KItN1	T0KItN2	T0KItN2	T0KItN3	T0KItN3	T0KItN4	T0KItN4
B					T0KItS11	T0KItS11	T0KItS12	T0KItS12	T0KItS13	T0KItS13	T0KItS14	T0KItS14
C					T0KItS21	T0KItS21	T0KItS22	T0KItS22	T0KItS23	T0KItS23	T0KItS24	T0KItS24
D					T1KItN1	T1KItN1	T1KItN2	T1KItN2	T1KItN3	T1KItN3	T1KItN4	T1KItN4
E					T1KItS11	T1KItS11	T1KItS12	T1KItS12	T1KItS13	T1KItS13	T1KItS14	T1KItS14
F					T1KItS21	T1KItS21	T1KItS22	T1KItS22	T1KItS23	T1KItS23	T1KItS24	T1KItS24
G							Blank PEG	Blank PEG	Blank PEG	Blank PEG	Blank PEG	Blank PEG
H							Blank no PEG	Blank no PEG	Blank no PEG	Blank no PEG	Blank no PEG	Blank no PEG

T0 dil.11*dil2,75=dil.30,25
T1 dil.10*dil2,75=dil.27,5

Comment:

Samples aufgeteilt in lin high und low:niedriger konzentrierte Samples bei lin low zu finden.

Date Operator

Date Control

T1KltN4	T0/T1	D12	0	0	kleiner LLOQ	#VALUE!	1	#VALUE!
0	T0/T1	E01	0	0	kleiner LLOQ	#VALUE!	1	#VALUE!
0	T0/T1	E02	0	0	kleiner LLOQ	#VALUE!	1	#VALUE!
0	T0/T1	E03	0	0	kleiner LLOQ	#VALUE!	1	#VALUE!
0	T0/T1	E04	0	0	kleiner LLOQ	#VALUE!	1	#VALUE!
T1KltS11	T0/T1	E05	0	0	kleiner LLOQ	#VALUE!	1	#VALUE!
T1KltS11	T0/T1	E06	0	0	kleiner LLOQ	#VALUE!	1	#VALUE!
T1KltS12	T0/T1	E07	0	0	kleiner LLOQ	#VALUE!	1	#VALUE!
T1KltS12	T0/T1	E08	0	0	kleiner LLOQ	#VALUE!	1	#VALUE!
T1KltS13	T0/T1	E09	0	0	kleiner LLOQ	#VALUE!	1	#VALUE!
T1KltS13	T0/T1	E10	0	0	kleiner LLOQ	#VALUE!	1	#VALUE!
T1KltS14	T0/T1	E11	0	0	kleiner LLOQ	#VALUE!	1	#VALUE!
T1KltS14	T0/T1	E12	0	0	kleiner LLOQ	#VALUE!	1	#VALUE!
0	T0/T1	F01	0	0	kleiner LLOQ	#VALUE!	1	#VALUE!
0	T0/T1	F02	0	0	kleiner LLOQ	#VALUE!	1	#VALUE!
0	T0/T1	F03	0	0	kleiner LLOQ	#VALUE!	1	#VALUE!
0	T0/T1	F04	0	0	kleiner LLOQ	#VALUE!	1	#VALUE!
T1KltS21	T0/T1	F05	0	0	kleiner LLOQ	#VALUE!	1	#VALUE!
T1KltS21	T0/T1	F06	0	0	kleiner LLOQ	#VALUE!	1	#VALUE!
T1KltS22	T0/T1	F07	0	0	kleiner LLOQ	#VALUE!	1	#VALUE!
T1KltS22	T0/T1	F08	0	0	kleiner LLOQ	#VALUE!	1	#VALUE!
T1KltS23	T0/T1	F09	0	0	kleiner LLOQ	#VALUE!	1	#VALUE!
T1KltS23	T0/T1	F10	0	0	kleiner LLOQ	#VALUE!	1	#VALUE!
T1KltS24	T0/T1	F11	0	0	kleiner LLOQ	#VALUE!	1	#VALUE!
T1KltS24	T0/T1	F12	0	0	kleiner LLOQ	#VALUE!	1	#VALUE!
0	T0/T1	G01	0	0	kleiner LLOQ	#VALUE!	1	#VALUE!
0	T0/T1	G02	0	0	kleiner LLOQ	#VALUE!	1	#VALUE!
0	T0/T1	G03	0	0	kleiner LLOQ	#VALUE!	1	#VALUE!
0	T0/T1	G04	0	0	kleiner LLOQ	#VALUE!	1	#VALUE!
0	T0/T1	G05	0	0	kleiner LLOQ	#VALUE!	1	#VALUE!
0	T0/T1	G06	0	0	kleiner LLOQ	#VALUE!	1	#VALUE!
Blank PEG	T0/T1	G07	0.050	0	kleiner LLOQ	#VALUE!	2.75	#VALUE!
Blank PEG	T0/T1	G08	0.051	0	kleiner LLOQ	#VALUE!	2.75	#VALUE!
Blank PEG	T0/T1	G09	0.049	0	kleiner LLOQ	#VALUE!	2.75	#VALUE!
Blank PEG	T0/T1	G10	0.045	0	kleiner LLOQ	#VALUE!	2.75	#VALUE!
Blank PEG	T0/T1	G11	0.047	0	kleiner LLOQ	#VALUE!	2.75	#VALUE!
Blank PEG	T0/T1	G12	0.049	0	kleiner LLOQ	#VALUE!	2.75	#VALUE!
0	T0/T1	H01	0	0	kleiner LLOQ	#VALUE!	1	#VALUE!
0	T0/T1	H02	0	0	kleiner LLOQ	#VALUE!	1	#VALUE!
0	T0/T1	H03	0	0	kleiner LLOQ	#VALUE!	1	#VALUE!
0	T0/T1	H04	0	0	kleiner LLOQ	#VALUE!	1	#VALUE!
0	T0/T1	H05	0	0	kleiner LLOQ	#VALUE!	1	#VALUE!
0	T0/T1	H06	0	0	kleiner LLOQ	#VALUE!	1	#VALUE!
Blank no PEG	T0/T1	H07	0.049	0	kleiner LLOQ	#VALUE!	2.75	#VALUE!
Blank no PEG	T0/T1	H08	0.048	0	kleiner LLOQ	#VALUE!	2.75	#VALUE!
Blank no PEG	T0/T1	H09	0.048	0	kleiner LLOQ	#VALUE!	2.75	#VALUE!
Blank no PEG	T0/T1	H10	0.048	0	kleiner LLOQ	#VALUE!	2.75	#VALUE!
Blank no PEG	T0/T1	H11	0.048	0	kleiner LLOQ	#VALUE!	2.75	#VALUE!
Blank no PEG	T0/T1	H12	0.048	0	kleiner LLOQ	#VALUE!	2.75	#VALUE!

0	T2/T3	A01	0	0	kleiner LLOQ	#VALUE!	2	#VALUE!
0	T2/T3	A02	0	0	kleiner LLOQ	#VALUE!	3	#VALUE!
0	T2/T3	A03	0	0	kleiner LLOQ	#VALUE!	4	#VALUE!
0	T2/T3	A04	0	0	kleiner LLOQ	#VALUE!	5	#VALUE!
T2KltN1	T2/T3	A05	0	0	kleiner LLOQ	#VALUE!	6	#VALUE!
T2KltN1	T2/T3	A06	0	0	kleiner LLOQ	#VALUE!	7	#VALUE!
T2KltN2	T2/T3	A07	0	0	kleiner LLOQ	#VALUE!	8	#VALUE!
T2KltN2	T2/T3	A08	0	0	kleiner LLOQ	#VALUE!	9	#VALUE!
T2KltN3	T2/T3	A09	0	0	kleiner LLOQ	#VALUE!	10	#VALUE!
T2KltN3	T2/T3	A10	0	0	kleiner LLOQ	#VALUE!	11	#VALUE!
T2KltN4	T2/T3	A11	0	0	kleiner LLOQ	#VALUE!	12	#VALUE!
T2KltN4	T2/T3	A12	0	0	kleiner LLOQ	#VALUE!	13	#VALUE!
0	T2/T3	B01	0	0	kleiner LLOQ	#VALUE!	14	#VALUE!
0	T2/T3	B02	0	0	kleiner LLOQ	#VALUE!	15	#VALUE!
0	T2/T3	B03	0	0	kleiner LLOQ	#VALUE!	16	#VALUE!
0	T2/T3	B04	0	0	kleiner LLOQ	#VALUE!	17	#VALUE!
T2KltS11	T2/T3	B05	0	0	kleiner LLOQ	#VALUE!	18	#VALUE!
T2KltS11	T2/T3	B06	0	0	kleiner LLOQ	#VALUE!	19	#VALUE!
T2KltS12	T2/T3	B07	0	0	kleiner LLOQ	#VALUE!	20	#VALUE!
T2KltS12	T2/T3	B08	0	0	kleiner LLOQ	#VALUE!	21	#VALUE!
T2KltS13	T2/T3	B09	0	0	kleiner LLOQ	#VALUE!	22	#VALUE!
T2KltS13	T2/T3	B10	0	0	kleiner LLOQ	#VALUE!	23	#VALUE!
T2KltS14	T2/T3	B11	0	0	kleiner LLOQ	#VALUE!	24	#VALUE!
T2KltS14	T2/T3	B12	0	0	kleiner LLOQ	#VALUE!	25	#VALUE!
0	T2/T3	C01	0	0	kleiner LLOQ	#VALUE!	26	#VALUE!
0	T2/T3	C02	0	0	kleiner LLOQ	#VALUE!	27	#VALUE!
0	T2/T3	C03	0	0	kleiner LLOQ	#VALUE!	28	#VALUE!
0	T2/T3	C04	0	0	kleiner LLOQ	#VALUE!	29	#VALUE!
T2KltS21	T2/T3	C05	0	0	kleiner LLOQ	#VALUE!	30	#VALUE!
T2KltS21	T2/T3	C06	0	0	kleiner LLOQ	#VALUE!	31	#VALUE!
T2KltS22	T2/T3	C07	0	0	kleiner LLOQ	#VALUE!	32	#VALUE!
T2KltS22	T2/T3	C08	0	0	kleiner LLOQ	#VALUE!	33	#VALUE!
T2KltS23	T2/T3	C09	0	0	kleiner LLOQ	#VALUE!	34	#VALUE!
T2KltS23	T2/T3	C10	0	0	kleiner LLOQ	#VALUE!	35	#VALUE!
T2KltS24	T2/T3	C11	0	0	kleiner LLOQ	#VALUE!	36	#VALUE!
T2KltS24	T2/T3	C12	0	0	kleiner LLOQ	#VALUE!	37	#VALUE!
0	T2/T3	D01	0	0	kleiner LLOQ	#VALUE!	38	#VALUE!
0	T2/T3	D02	0	0	kleiner LLOQ	#VALUE!	39	#VALUE!
0	T2/T3	D03	0	0	kleiner LLOQ	#VALUE!	40	#VALUE!
0	T2/T3	D04	0	0	kleiner LLOQ	#VALUE!	41	#VALUE!
T3KltN1	T2/T3	D05	0	0	kleiner LLOQ	#VALUE!	42	#VALUE!
T3KltN1	T2/T3	D06	0	0	kleiner LLOQ	#VALUE!	43	#VALUE!
T3KltN2	T2/T3	D07	0	0	kleiner LLOQ	#VALUE!	44	#VALUE!
T3KltN2	T2/T3	D08	0	0	kleiner LLOQ	#VALUE!	45	#VALUE!
T3KltN3	T2/T3	D09	0	0	kleiner LLOQ	#VALUE!	46	#VALUE!
T3KltN3	T2/T3	D10	0	0	kleiner LLOQ	#VALUE!	47	#VALUE!
T3KltN4	T2/T3	D11	0	0	kleiner LLOQ	#VALUE!	48	#VALUE!
T3KltN4	T2/T3	D12	0	0	kleiner LLOQ	#VALUE!	49	#VALUE!

Plate: T2/T3

	1	2	3	4	5	6	7	8	9	10	11	12
A												
B												
C												
D												
E												
F												
G							0.050	0.050	0.048	0.050	0.050	0.050
H							0.050	0.049	0.051	0.050	0.050	0.050

Plattenbelegung

	1	2	3	4	5	6	7	8	9	10	11	12
A					T2KltN1	T2KltN1	T2KltN2	T2KltN2	T2KltN3	T2KltN3	T2KltN4	T2KltN4
B					T2KltS11	T2KltS11	T2KltS12	T2KltS12	T2KltS13	T2KltS13	T2KltS14	T2KltS14
C					T2KltS21	T2KltS21	T2KltS22	T2KltS22	T2KltS23	T2KltS23	T2KltS24	T2KltS24
D					T3KltN1	T3KltN1	T3KltN2	T3KltN2	T3KltN3	T3KltN3	T3KltN4	T3KltN4
E					T3KltS11	T3KltS11	T3KltS12	T3KltS12	T3KltS13	T3KltS13	T3KltS14	T3KltS14
F					T3KltS21	T3KltS21	T3KltS22	T3KltS22	T3KltS23	T3KltS23	T3KltS24	T3KltS24
G							Blank PEG	Blank PEG	Blank PEG	Blank PEG	Blank PEG	Blank PEG
H							Blank no PEG	Blank no PEG	Blank no PEG	Blank no PEG	Blank no PEG	Blank no PEG

T2 dil.9*dil.2,75=dil.24,75
T3 dil.6*dil.2,75=dil.16,5

Comment:
alle Messwerte lin low

Date Operator

Date Control

0	T2/T3	E01	0	0	kleiner LLOQ	#VALUE!	50	#VALUE!
0	T2/T3	E02	0	0	kleiner LLOQ	#VALUE!	51	#VALUE!
0	T2/T3	E03	0	0	kleiner LLOQ	#VALUE!	52	#VALUE!
0	T2/T3	E04	0	0	kleiner LLOQ	#VALUE!	53	#VALUE!
T3KltS11	T2/T3	E05	0	0	kleiner LLOQ	#VALUE!	54	#VALUE!
T3KltS11	T2/T3	E06	0	0	kleiner LLOQ	#VALUE!	55	#VALUE!
T3KltS12	T2/T3	E07	0	0	kleiner LLOQ	#VALUE!	56	#VALUE!
T3KltS12	T2/T3	E08	0	0	kleiner LLOQ	#VALUE!	57	#VALUE!
T3KltS13	T2/T3	E09	0	0	kleiner LLOQ	#VALUE!	58	#VALUE!
T3KltS13	T2/T3	E10	0	0	kleiner LLOQ	#VALUE!	59	#VALUE!
T3KltS14	T2/T3	E11	0	0	kleiner LLOQ	#VALUE!	60	#VALUE!
T3KltS14	T2/T3	E12	0	0	kleiner LLOQ	#VALUE!	61	#VALUE!
0	T2/T3	F01	0	0	kleiner LLOQ	#VALUE!	62	#VALUE!
0	T2/T3	F02	0	0	kleiner LLOQ	#VALUE!	63	#VALUE!
0	T2/T3	F03	0	0	kleiner LLOQ	#VALUE!	64	#VALUE!
0	T2/T3	F04	0	0	kleiner LLOQ	#VALUE!	65	#VALUE!
T3KltS21	T2/T3	F05	0	0	kleiner LLOQ	#VALUE!	66	#VALUE!
T3KltS21	T2/T3	F06	0	0	kleiner LLOQ	#VALUE!	67	#VALUE!
T3KltS22	T2/T3	F07	0	0	kleiner LLOQ	#VALUE!	68	#VALUE!
T3KltS22	T2/T3	F08	0	0	kleiner LLOQ	#VALUE!	69	#VALUE!
T3KltS23	T2/T3	F09	0	0	kleiner LLOQ	#VALUE!	70	#VALUE!
T3KltS23	T2/T3	F10	0	0	kleiner LLOQ	#VALUE!	71	#VALUE!
T3KltS24	T2/T3	F11	0	0	kleiner LLOQ	#VALUE!	72	#VALUE!
T3KltS24	T2/T3	F12	0	0	kleiner LLOQ	#VALUE!	73	#VALUE!
0	T2/T3	G01	0	0	kleiner LLOQ	#VALUE!	74	#VALUE!
0	T2/T3	G02	0	0	kleiner LLOQ	#VALUE!	75	#VALUE!
0	T2/T3	G03	0	0	kleiner LLOQ	#VALUE!	76	#VALUE!
0	T2/T3	G04	0	0	kleiner LLOQ	#VALUE!	77	#VALUE!
0	T2/T3	G05	0	0	kleiner LLOQ	#VALUE!	78	#VALUE!
0	T2/T3	G06	0	0	kleiner LLOQ	#VALUE!	79	#VALUE!
Blank PEG	T2/T3	G07	0	0	kleiner LLOQ	#VALUE!	80	#VALUE!
Blank PEG	T2/T3	G08	0	0	kleiner LLOQ	#VALUE!	81	#VALUE!
Blank PEG	T2/T3	G09	0	0	kleiner LLOQ	#VALUE!	82	#VALUE!
Blank PEG	T2/T3	G10	0	0	kleiner LLOQ	#VALUE!	83	#VALUE!
Blank PEG	T2/T3	G11	0	0	kleiner LLOQ	#VALUE!	84	#VALUE!
Blank PEG	T2/T3	G12	0	0	kleiner LLOQ	#VALUE!	85	#VALUE!
0	T2/T3	H01	0	0	kleiner LLOQ	#VALUE!	86	#VALUE!
0	T2/T3	H02	0	0	kleiner LLOQ	#VALUE!	87	#VALUE!
0	T2/T3	H03	0	0	kleiner LLOQ	#VALUE!	88	#VALUE!
0	T2/T3	H04	0	0	kleiner LLOQ	#VALUE!	89	#VALUE!
0	T2/T3	H05	0	0	kleiner LLOQ	#VALUE!	90	#VALUE!
0	T2/T3	H06	0	0	kleiner LLOQ	#VALUE!	91	#VALUE!
Blank no PEG	T2/T3	H07	0	0	kleiner LLOQ	#VALUE!	92	#VALUE!
Blank no PEG	T2/T3	H08	0	0	kleiner LLOQ	#VALUE!	93	#VALUE!
Blank no PEG	T2/T3	H09	0	0	kleiner LLOQ	#VALUE!	94	#VALUE!
Blank no PEG	T2/T3	H10	0	0	kleiner LLOQ	#VALUE!	95	#VALUE!
Blank no PEG	T2/T3	H11	0	0	kleiner LLOQ	#VALUE!	96	#VALUE!
Blank no PEG	T2/T3	H12	0	0	kleiner LLOQ	#VALUE!	97	#VALUE!

0	T4/T5	A01	0	0	kleiner LLOQ	#VALUE!	98	#VALUE!
0	T4/T5	A02	0	0	kleiner LLOQ	#VALUE!	99	#VALUE!
0	T4/T5	A03	0	0	kleiner LLOQ	#VALUE!	100	#VALUE!
0	T4/T5	A04	0	0	kleiner LLOQ	#VALUE!	101	#VALUE!
T4KltN1	T4/T5	A05	0	0	kleiner LLOQ	#VALUE!	102	#VALUE!
T4KltN1	T4/T5	A06	0	0	kleiner LLOQ	#VALUE!	103	#VALUE!
T4KltN2	T4/T5	A07	0	0	kleiner LLOQ	#VALUE!	104	#VALUE!
T4KltN2	T4/T5	A08	0	0	kleiner LLOQ	#VALUE!	105	#VALUE!
T4KltN3	T4/T5	A09	0	0	kleiner LLOQ	#VALUE!	106	#VALUE!
T4KltN3	T4/T5	A10	0	0	kleiner LLOQ	#VALUE!	107	#VALUE!
T4KltN4	T4/T5	A11	0	0	kleiner LLOQ	#VALUE!	108	#VALUE!
T4KltN4	T4/T5	A12	0	0	kleiner LLOQ	#VALUE!	109	#VALUE!
0	T4/T5	B01	0	0	kleiner LLOQ	#VALUE!	110	#VALUE!
0	T4/T5	B02	0	0	kleiner LLOQ	#VALUE!	111	#VALUE!
0	T4/T5	B03	0	0	kleiner LLOQ	#VALUE!	112	#VALUE!
0	T4/T5	B04	0	0	kleiner LLOQ	#VALUE!	113	#VALUE!
T4KltS11	T4/T5	B05	0	0	kleiner LLOQ	#VALUE!	114	#VALUE!
T4KltS11	T4/T5	B06	0	0	kleiner LLOQ	#VALUE!	115	#VALUE!
T4KltS12	T4/T5	B07	0	0	kleiner LLOQ	#VALUE!	116	#VALUE!
T4KltS12	T4/T5	B08	0	0	kleiner LLOQ	#VALUE!	117	#VALUE!
T4KltS13	T4/T5	B09	0	0	kleiner LLOQ	#VALUE!	118	#VALUE!
T4KltS13	T4/T5	B10	0	0	kleiner LLOQ	#VALUE!	119	#VALUE!
T4KltS14	T4/T5	B11	0	0	kleiner LLOQ	#VALUE!	120	#VALUE!
T4KltS14	T4/T5	B12	0	0	kleiner LLOQ	#VALUE!	121	#VALUE!
0	T4/T5	C01	0	0	kleiner LLOQ	#VALUE!	122	#VALUE!
0	T4/T5	C02	0	0	kleiner LLOQ	#VALUE!	123	#VALUE!
0	T4/T5	C03	0	0	kleiner LLOQ	#VALUE!	124	#VALUE!
0	T4/T5	C04	0	0	kleiner LLOQ	#VALUE!	125	#VALUE!
T4KltS21	T4/T5	C05	0	0	kleiner LLOQ	#VALUE!	126	#VALUE!
T4KltS21	T4/T5	C06	0	0	kleiner LLOQ	#VALUE!	127	#VALUE!
T4KltS22	T4/T5	C07	0	0	kleiner LLOQ	#VALUE!	128	#VALUE!
T4KltS22	T4/T5	C08	0	0	kleiner LLOQ	#VALUE!	129	#VALUE!
T4KltS23	T4/T5	C09	0	0	kleiner LLOQ	#VALUE!	130	#VALUE!
T4KltS23	T4/T5	C10	0	0	kleiner LLOQ	#VALUE!	131	#VALUE!
T4KltS24	T4/T5	C11	0	0	kleiner LLOQ	#VALUE!	132	#VALUE!
T4KltS24	T4/T5	C12	0	0	kleiner LLOQ	#VALUE!	133	#VALUE!
0	T4/T5	D01	0	0	kleiner LLOQ	#VALUE!	134	#VALUE!
0	T4/T5	D02	0	0	kleiner LLOQ	#VALUE!	135	#VALUE!
0	T4/T5	D03	0	0	kleiner LLOQ	#VALUE!	136	#VALUE!
0	T4/T5	D04	0	0	kleiner LLOQ	#VALUE!	137	#VALUE!
T5KltN1	T4/T5	D05	0	0	kleiner LLOQ	#VALUE!	138	#VALUE!
T5KltN1	T4/T5	D06	0	0	kleiner LLOQ	#VALUE!	139	#VALUE!
T5KltN2	T4/T5	D07	0	0	kleiner LLOQ	#VALUE!	140	#VALUE!
T5KltN2	T4/T5	D08	0	0	kleiner LLOQ	#VALUE!	141	#VALUE!
T5KltN3	T4/T5	D09	0	0	kleiner LLOQ	#VALUE!	142	#VALUE!

Plate: T4/T5

	1	2	3	4	5	6	7	8	9	10	11	12
A												
B												
C												
D												
E												
F												
G												
H												

Plattenbelegung

	1	2	3	4	5	6	7	8	9	10	11	12
A					T4KltN1	T4KltN1	T4KltN2	T4KltN2	T4KltN3	T4KltN3	T4KltN4	T4KltN4
B					T4KltS11	T4KltS11	T4KltS12	T4KltS12	T4KltS13	T4KltS13	T4KltS14	T4KltS14
C					T4KltS21	T4KltS21	T4KltS22	T4KltS22	T4KltS23	T4KltS23	T4KltS24	T4KltS24
D					T5KltN1	T5KltN1	T5KltN2	T5KltN2	T5KltN3	T5KltN3	T5KltN4	T5KltN4
E					T5KltS11	T5KltS11	T5KltS12	T5KltS12	T5KltS13	T5KltS13	T5KltS14	T5KltS14
F					T5KltS21	T5KltS21	T5KltS22	T5KltS22	T5KltS23	T5KltS23	T5KltS24	T5KltS24
G							Blank PEG	Blank PEG	Blank PEG	Blank PEG	Blank PEG	Blank PEG
H							Blank no PEG	Blank no PEG	Blank no PEG	Blank no PEG	Blank no PEG	Blank no PEG

Comment:
alle Messwerte bei lin low

Date Operator

Date Control

T5KltN3	T4/T5	D10	0	0	kleiner LLOQ	#VALUE!	143	#VALUE!
T5KltN4	T4/T5	D11	0	0	kleiner LLOQ	#VALUE!	144	#VALUE!
T5KltN4	T4/T5	D12	0	0	kleiner LLOQ	#VALUE!	145	#VALUE!
0	T4/T5	E01	0	0	kleiner LLOQ	#VALUE!	146	#VALUE!
0	T4/T5	E02	0	0	kleiner LLOQ	#VALUE!	147	#VALUE!
0	T4/T5	E03	0	0	kleiner LLOQ	#VALUE!	148	#VALUE!
0	T4/T5	E04	0	0	kleiner LLOQ	#VALUE!	149	#VALUE!
T5KltS11	T4/T5	E05	0	0	kleiner LLOQ	#VALUE!	150	#VALUE!
T5KltS11	T4/T5	E06	0	0	kleiner LLOQ	#VALUE!	151	#VALUE!
T5KltS12	T4/T5	E07	0	0	kleiner LLOQ	#VALUE!	152	#VALUE!
T5KltS12	T4/T5	E08	0	0	kleiner LLOQ	#VALUE!	153	#VALUE!
T5KltS13	T4/T5	E09	0	0	kleiner LLOQ	#VALUE!	154	#VALUE!
T5KltS13	T4/T5	E10	0	0	kleiner LLOQ	#VALUE!	155	#VALUE!
T5KltS14	T4/T5	E11	0	0	kleiner LLOQ	#VALUE!	156	#VALUE!
T5KltS14	T4/T5	E12	0	0	kleiner LLOQ	#VALUE!	157	#VALUE!
0	T4/T5	F01	0	0	kleiner LLOQ	#VALUE!	158	#VALUE!
0	T4/T5	F02	0	0	kleiner LLOQ	#VALUE!	159	#VALUE!
0	T4/T5	F03	0	0	kleiner LLOQ	#VALUE!	160	#VALUE!
0	T4/T5	F04	0	0	kleiner LLOQ	#VALUE!	161	#VALUE!
T5KltS21	T4/T5	F05	0	0	kleiner LLOQ	#VALUE!	162	#VALUE!
T5KltS21	T4/T5	F06	0	0	kleiner LLOQ	#VALUE!	163	#VALUE!
T5KltS22	T4/T5	F07	0	0	kleiner LLOQ	#VALUE!	164	#VALUE!
T5KltS22	T4/T5	F08	0	0	kleiner LLOQ	#VALUE!	165	#VALUE!
T5KltS23	T4/T5	F09	0	0	kleiner LLOQ	#VALUE!	166	#VALUE!
T5KltS23	T4/T5	F10	0	0	kleiner LLOQ	#VALUE!	167	#VALUE!
T5KltS24	T4/T5	F11	0	0	kleiner LLOQ	#VALUE!	168	#VALUE!
T5KltS24	T4/T5	F12	0	0	kleiner LLOQ	#VALUE!	169	#VALUE!
0	T4/T5	G01	0	0	kleiner LLOQ	#VALUE!	170	#VALUE!
0	T4/T5	G02	0	0	kleiner LLOQ	#VALUE!	171	#VALUE!
0	T4/T5	G03	0	0	kleiner LLOQ	#VALUE!	172	#VALUE!
0	T4/T5	G04	0	0	kleiner LLOQ	#VALUE!	173	#VALUE!
0	T4/T5	G05	0	0	kleiner LLOQ	#VALUE!	174	#VALUE!
0	T4/T5	G06	0	0	kleiner LLOQ	#VALUE!	175	#VALUE!
Blank PEG	T4/T5	G07	0	0	kleiner LLOQ	#VALUE!	176	#VALUE!
Blank PEG	T4/T5	G08	0	0	kleiner LLOQ	#VALUE!	177	#VALUE!
Blank PEG	T4/T5	G09	0	0	kleiner LLOQ	#VALUE!	178	#VALUE!
Blank PEG	T4/T5	G10	0	0	kleiner LLOQ	#VALUE!	179	#VALUE!
Blank PEG	T4/T5	G11	0	0	kleiner LLOQ	#VALUE!	180	#VALUE!
Blank PEG	T4/T5	G12	0	0	kleiner LLOQ	#VALUE!	181	#VALUE!
0	T4/T5	H01	0	0	kleiner LLOQ	#VALUE!	182	#VALUE!
0	T4/T5	H02	0	0	kleiner LLOQ	#VALUE!	183	#VALUE!
0	T4/T5	H03	0	0	kleiner LLOQ	#VALUE!	184	#VALUE!
0	T4/T5	H04	0	0	kleiner LLOQ	#VALUE!	185	#VALUE!
0	T4/T5	H05	0	0	kleiner LLOQ	#VALUE!	186	#VALUE!
0	T4/T5	H06	0	0	kleiner LLOQ	#VALUE!	187	#VALUE!
Blank no PEG	T4/T5	H07	0	0	kleiner LLOQ	#VALUE!	188	#VALUE!
Blank no PEG	T4/T5	H08	0	0	kleiner LLOQ	#VALUE!	189	#VALUE!
Blank no PEG	T4/T5	H09	0	0	kleiner LLOQ	#VALUE!	190	#VALUE!
Blank no PEG	T4/T5	H10	0	0	kleiner LLOQ	#VALUE!	191	#VALUE!
Blank no PEG	T4/T5	H11	0	0	kleiner LLOQ	#VALUE!	192	#VALUE!
Blank no PEG	T4/T5	H12	0	0	kleiner LLOQ	#VALUE!	193	#VALUE!

0	T6/T7	A01	0	0	kleiner LLOQ	#VALUE!	194	#VALUE!
0	T6/T7	A02	0	0	kleiner LLOQ	#VALUE!	195	#VALUE!
0	T6/T7	A03	0	0	kleiner LLOQ	#VALUE!	196	#VALUE!
0	T6/T7	A04	0	0	kleiner LLOQ	#VALUE!	197	#VALUE!
T6KltN1	T6/T7	A05	0	0	kleiner LLOQ	#VALUE!	198	#VALUE!
T6KltN1	T6/T7	A06	0	0	kleiner LLOQ	#VALUE!	199	#VALUE!
T6KltN2	T6/T7	A07	0	0	kleiner LLOQ	#VALUE!	200	#VALUE!
T6KltN2	T6/T7	A08	0	0	kleiner LLOQ	#VALUE!	201	#VALUE!
T6KltN3	T6/T7	A09	0	0	kleiner LLOQ	#VALUE!	202	#VALUE!
T6KltN3	T6/T7	A10	0	0	kleiner LLOQ	#VALUE!	203	#VALUE!
T6KltN4	T6/T7	A11	0	0	kleiner LLOQ	#VALUE!	204	#VALUE!
T6KltN4	T6/T7	A12	0	0	kleiner LLOQ	#VALUE!	205	#VALUE!
0	T6/T7	B01	0	0	kleiner LLOQ	#VALUE!	206	#VALUE!
0	T6/T7	B02	0	0	kleiner LLOQ	#VALUE!	207	#VALUE!
0	T6/T7	B03	0	0	kleiner LLOQ	#VALUE!	208	#VALUE!
0	T6/T7	B04	0	0	kleiner LLOQ	#VALUE!	209	#VALUE!
T6KltS11	T6/T7	B05	0	0	kleiner LLOQ	#VALUE!	210	#VALUE!
T6KltS11	T6/T7	B06	0	0	kleiner LLOQ	#VALUE!	211	#VALUE!
T6KltS12	T6/T7	B07	0	0	kleiner LLOQ	#VALUE!	212	#VALUE!
T6KltS12	T6/T7	B08	0	0	kleiner LLOQ	#VALUE!	213	#VALUE!
T6KltS13	T6/T7	B09	0	0	kleiner LLOQ	#VALUE!	214	#VALUE!
T6KltS13	T6/T7	B10	0	0	kleiner LLOQ	#VALUE!	215	#VALUE!
T6KltS14	T6/T7	B11	0	0	kleiner LLOQ	#VALUE!	216	#VALUE!
T6KltS14	T6/T7	B12	0	0	kleiner LLOQ	#VALUE!	217	#VALUE!
0	T6/T7	C01	0	0	kleiner LLOQ	#VALUE!	218	#VALUE!
0	T6/T7	C02	0	0	kleiner LLOQ	#VALUE!	219	#VALUE!
0	T6/T7	C03	0	0	kleiner LLOQ	#VALUE!	220	#VALUE!
0	T6/T7	C04	0	0	kleiner LLOQ	#VALUE!	221	#VALUE!
T6KltS21	T6/T7	C05	0	0	kleiner LLOQ	#VALUE!	222	#VALUE!
T6KltS21	T6/T7	C06	0	0	kleiner LLOQ	#VALUE!	223	#VALUE!
T6KltS22	T6/T7	C07	0	0	kleiner LLOQ	#VALUE!	224	#VALUE!
T6KltS22	T6/T7	C08	0	0	kleiner LLOQ	#VALUE!	225	#VALUE!
T6KltS23	T6/T7	C09	0	0	kleiner LLOQ	#VALUE!	226	#VALUE!
T6KltS23	T6/T7	C10	0	0	kleiner LLOQ	#VALUE!	227	#VALUE!
T6KltS24	T6/T7	C11	0	0	kleiner LLOQ	#VALUE!	228	#VALUE!
T6KltS24	T6/T7	C12	0	0	kleiner LLOQ	#VALUE!	229	#VALUE!
0	T6/T7	D01	0	0	kleiner LLOQ	#VALUE!	230	#VALUE!
0	T6/T7	D02	0	0	kleiner LLOQ	#VALUE!	231	#VALUE!
0	T6/T7	D03	0	0	kleiner LLOQ	#VALUE!	232	#VALUE!
0	T6/T7	D04	0	0	kleiner LLOQ	#VALUE!	233	#VALUE!
T7KltN1	T6/T7	D05	0	0	kleiner LLOQ	#VALUE!	234	#VALUE!
T7KltN1	T6/T7	D06	0	0	kleiner LLOQ	#VALUE!	235	#VALUE!
T7KltN2	T6/T7	D07	0	0	kleiner LLOQ	#VALUE!	236	#VALUE!
T7KltN2	T6/T7	D08	0	0	kleiner LLOQ	#VALUE!	237	#VALUE!
T7KltN3	T6/T7	D09	0	0	kleiner LLOQ	#VALUE!	238	#VALUE!
T7KltN3	T6/T7	D10	0	0	kleiner LLOQ	#VALUE!	239	#VALUE!
T7KltN4	T6/T7	D11	0	0	kleiner LLOQ	#VALUE!	240	#VALUE!
T7KltN4	T6/T7	D12	0	0	kleiner LLOQ	#VALUE!	241	#VALUE!
0	T6/T7	E01	0	0	kleiner LLOQ	#VALUE!	242	#VALUE!

Plate: T6/T7

	1	2	3	4	5	6	7	8	9	10	11	12
A												
B												
C												
D												
E												
F												
G												
H												

Plattenbelegung

	1	2	3	4	5	6	7	8	9	10	11	12
A					T6KltN1	T6KltN1	T6KltN2	T6KltN2	T6KltN3	T6KltN3	T6KltN4	T6KltN4
B					T6KltS11	T6KltS11	T6KltS12	T6KltS12	T6KltS13	T6KltS13	T6KltS14	T6KltS14
C					T6KltS21	T6KltS21	T6KltS22	T6KltS22	T6KltS23	T6KltS23	T6KltS24	T6KltS24
D					T7KltN1	T7KltN1	T7KltN2	T7KltN2	T7KltN3	T7KltN3	T7KltN4	T7KltN4
E					T7KltS11	T7KltS11	T7KltS12	T7KltS12	T7KltS13	T7KltS13	T7KltS14	T7KltS14
F					T7KltS21	T7KltS21	T7KltS22	T7KltS22	T7KltS23	T7KltS23	T7KltS24	T7KltS24
G												
H												

Comment:

alle Messwerte bei lin low.

Date Operator

Date Control

0	T6/T7	E02	0	0	kleiner LLOQ	#VALUE!	243	#VALUE!
0	T6/T7	E03	0	0	kleiner LLOQ	#VALUE!	244	#VALUE!
0	T6/T7	E04	0	0	kleiner LLOQ	#VALUE!	245	#VALUE!
T7KltS11	T6/T7	E05	0	0	kleiner LLOQ	#VALUE!	246	#VALUE!
T7KltS11	T6/T7	E06	0	0	kleiner LLOQ	#VALUE!	247	#VALUE!
T7KltS12	T6/T7	E07	0	0	kleiner LLOQ	#VALUE!	248	#VALUE!
T7KltS12	T6/T7	E08	0	0	kleiner LLOQ	#VALUE!	249	#VALUE!
T7KltS13	T6/T7	E09	0	0	kleiner LLOQ	#VALUE!	250	#VALUE!
T7KltS13	T6/T7	E10	0	0	kleiner LLOQ	#VALUE!	251	#VALUE!
T7KltS14	T6/T7	E11	0	0	kleiner LLOQ	#VALUE!	252	#VALUE!
T7KltS14	T6/T7	E12	0	0	kleiner LLOQ	#VALUE!	253	#VALUE!
0	T6/T7	F01	0	0	kleiner LLOQ	#VALUE!	254	#VALUE!
0	T6/T7	F02	0	0	kleiner LLOQ	#VALUE!	255	#VALUE!
0	T6/T7	F03	0	0	kleiner LLOQ	#VALUE!	256	#VALUE!
0	T6/T7	F04	0	0	kleiner LLOQ	#VALUE!	257	#VALUE!
T7KltS21	T6/T7	F05	0	0	kleiner LLOQ	#VALUE!	258	#VALUE!
T7KltS21	T6/T7	F06	0	0	kleiner LLOQ	#VALUE!	259	#VALUE!
T7KltS22	T6/T7	F07	0	0	kleiner LLOQ	#VALUE!	260	#VALUE!
T7KltS22	T6/T7	F08	0	0	kleiner LLOQ	#VALUE!	261	#VALUE!
T7KltS23	T6/T7	F09	0	0	kleiner LLOQ	#VALUE!	262	#VALUE!
T7KltS23	T6/T7	F10	0	0	kleiner LLOQ	#VALUE!	263	#VALUE!
T7KltS24	T6/T7	F11	0	0	kleiner LLOQ	#VALUE!	264	#VALUE!
T7KltS24	T6/T7	F12	0	0	kleiner LLOQ	#VALUE!	265	#VALUE!
0	T6/T7	G01	0	0	kleiner LLOQ	#VALUE!	266	#VALUE!
0	T6/T7	G02	0	0	kleiner LLOQ	#VALUE!	267	#VALUE!
0	T6/T7	G03	0	0	kleiner LLOQ	#VALUE!	268	#VALUE!
0	T6/T7	G04	0	0	kleiner LLOQ	#VALUE!	269	#VALUE!
0	T6/T7	G05	0	0	kleiner LLOQ	#VALUE!	270	#VALUE!
0	T6/T7	G06	0	0	kleiner LLOQ	#VALUE!	271	#VALUE!
0	T6/T7	G07	0	0	kleiner LLOQ	#VALUE!	272	#VALUE!
0	T6/T7	G08	0	0	kleiner LLOQ	#VALUE!	273	#VALUE!
0	T6/T7	G09	0	0	kleiner LLOQ	#VALUE!	274	#VALUE!
0	T6/T7	G10	0	0	kleiner LLOQ	#VALUE!	275	#VALUE!
0	T6/T7	G11	0	0	kleiner LLOQ	#VALUE!	276	#VALUE!
0	T6/T7	G12	0	0	kleiner LLOQ	#VALUE!	277	#VALUE!
0	T6/T7	H01	0	0	kleiner LLOQ	#VALUE!	278	#VALUE!
0	T6/T7	H02	0	0	kleiner LLOQ	#VALUE!	279	#VALUE!
0	T6/T7	H03	0	0	kleiner LLOQ	#VALUE!	280	#VALUE!
0	T6/T7	H04	0	0	kleiner LLOQ	#VALUE!	281	#VALUE!
0	T6/T7	H05	0	0	kleiner LLOQ	#VALUE!	282	#VALUE!
0	T6/T7	H06	0	0	kleiner LLOQ	#VALUE!	283	#VALUE!
0	T6/T7	H07	0	0	kleiner LLOQ	#VALUE!	284	#VALUE!
0	T6/T7	H08	0	0	kleiner LLOQ	#VALUE!	285	#VALUE!
0	T6/T7	H09	0	0	kleiner LLOQ	#VALUE!	286	#VALUE!
0	T6/T7	H10	0	0	kleiner LLOQ	#VALUE!	287	#VALUE!
0	T6/T7	H11	0	0	kleiner LLOQ	#VALUE!	288	#VALUE!
0	T6/T7	H12	0	0	kleiner LLOQ	#VALUE!	289	#VALUE!

1.447	1.739		1.170						0.667	0.440	0.043	1
1.447	1.332		1.392	0.137	0.146	0.130	0.138	0.109	0.729	0.478	0.042	
1.391	1.281		1.311	0.204	0.184	0.184	0.198	0.201	0.743	0.474	0.043	
0.961	0.835		1.405	0.336	0.347	0.375	0.387	0.300	0.824	0.483	0.044	
0.445	0.441		1.280	1.085	1.086	1.218	1.004	0.873	0.704	0.457	0.041	
0.355	0.315		1.332	1.768	1.925	1.753	1.739	1.606			0.036	
0.223	0.188	1.666	1.499	1.632		0.048	0.046	0.047	0.048	0.048	0.048	
0.118	0.106	1.556	1.622	1.573		0.047	0.054	0.051	0.051	0.052	0.051	

1.652	1.627		1.188	0.748	0.811	0.764	0.753	0.674	0.751	0.529	0.044	2
1.443	1.372		1.364	0.139	0.147	0.131	0.139	0.110	0.811	0.561	0.043	
1.377	1.281		1.284	0.206	0.189	0.184	0.200	0.202	0.821	0.560	0.044	
0.972	0.883		1.387	0.339	0.349	0.386	0.406	0.312	0.903	0.565	0.044	
0.484	0.474		1.298	1.087	1.113	1.203	1.022	0.903	0.790	0.544	0.042	
0.359	0.326		1.308	1.622	1.622	1.630	1.625	1.550			0.037	
0.228	0.199	1.527	1.373	1.462		0.048	0.046	0.048	0.049	0.049	0.049	
0.125	0.109	1.473	1.410	1.371		0.048	0.054	0.052	0.052	0.052	0.051	

1.579	1.475		1.150	1.210	1.303	1.202	1.219	1.125	0.759	0.549	0.043	3
1.357	1.303		1.283	0.140	0.148	0.131	0.139	0.111	0.820	0.575	0.043	
1.323	1.228		1.227	0.206	0.188	0.184	0.198	0.201	0.824	0.579	0.043	
0.959	0.872		1.321	0.339	0.348	0.387	0.406	0.314	0.909	0.580	0.044	
0.483	0.473		1.270	1.069	1.103	1.181	1.017	0.903	0.798	0.560	0.042	
0.360	0.326		1.270	1.497	1.448	1.496	1.494	1.449			0.037	
0.228	0.199	1.374	1.262	1.314		0.048	0.046	0.048	0.049	0.049	0.049	
0.125	0.110	1.346	1.262	1.253		0.048	0.054	0.052	0.052	0.052	0.051	

1.511	1.369			1.538	1.712	1.528	1.502	1.425			0.043	4
1.296	1.261										0.043	
1.272	1.193										0.043	
0.947	0.859										0.044	
0.481	0.471										0.042	
0.361	0.325										0.037	
0.229	0.199					0.048	0.046	0.048	0.049	0.049	0.049	
0.125	0.110					0.047	0.054	0.052	0.052	0.052	0.051	

1.493	1.317	1.678	1.110	1.621	1.807	1.618	1.584	1.508	0.765	0.597	0.043	5
1.259	1.238	1.850	1.197	0.139	0.150	0.132	0.139	0.111	0.821	0.588	0.043	
1.248	1.175	1.816	1.153	0.204	0.188	0.184	0.197	0.199	0.831	0.599	0.044	
0.943	0.859	1.970	1.231	0.338	0.357	0.386	0.406	0.316	0.916	0.595	0.044	
0.480	0.470	2.220	1.220	1.054	1.088	1.154	1.006	0.902	0.805	0.579	0.042	
0.359	0.325	2.149	1.211	1.395	1.324	1.348	1.345	1.317	0.037	0.037	0.037	
0.228	0.199	1.240	1.151	1.183	0.034	0.049	0.046	0.048	0.049	0.049	0.049	
0.125	0.110	1.223	1.132	1.146	0.038	0.048	0.054	0.052	0.052	0.052	0.051	falsch gedrückt, jedes well ob leer oder voll gemessen

1.434	1.258		1.090	1.648	1.876	1.649	1.627	1.535				6
1.217	1.201		1.149									
1.205	1.143		1.109									
0.935	0.845		1.182									
0.477	0.469		1.181									
0.360	0.323		1.167									
0.229	0.200					0.049	0.047	0.048	0.049	0.049	0.049	
0.125	0.110					0.048	0.054	0.052	0.052	0.052	0.051	

1.013	0.810		0.668	0.808	0.940	0.720	0.798	0.687	0.904	0.845	0.043	7
0.709	0.827		0.703	0.142	0.156	0.136	0.137	0.116	0.990	0.846	0.042	
0.682	0.692		0.680	0.197	0.188	0.190	0.191	0.184	1.023	0.861	0.043	
0.507	0.519		0.740	0.316	0.264	0.364	0.377	0.312	1.111	0.870	0.043	
0.394	0.406		0.682	0.580	0.561	0.600	0.529	0.563	0.962	0.834	0.042	
0.333	0.322		0.661	0.995	0.930	0.848	0.832	0.797	0.037	0.037	0.037	
0.229	0.201	0.843	0.679	0.732		0.048	0.046	0.047			0.047	
0.124	0.111	0.773	0.682	0.701		0.047	0.053	0.051	0.051	0.052	0.051	messung am nächsten tag, ca 24 h, bei 4°C

skin cultivation

3.015	3.090	3.028	0.727									T0/T1 1 hd
3.134	3.069	3.065	0.655									
3.055	3.129	3.032	0.418									
2.613	2.463	2.221	0.192									
2.914	2.846	2.705	0.363									
2.618	2.492	2.513	0.185									
						0.049	0.050	0.048	0.047	0.049	0.049	
						0.048	0.047	0.047	0.047	0.047	0.048	

				0.799	0.787	0.982	0.987	0.954	1.024	0.192	0.198	T0/T1 2 hd
				1.344	1.340	0.343	0.772	0.624	0.875	0.228	0.278	
				0.616	0.793	1.173	1.385	0.942	0.950	0.173	0.188	
				0.461	0.435	0.387	0.371	0.386	0.389	0.128	0.131	
				0.644	0.656	0.482	0.491	0.431	0.422	0.171	0.166	
				0.150	0.150	0.189	0.191	0.155	0.154	0.055	0.127	
						0.050	0.051	0.049	0.045	0.047	0.049	
						0.049	0.048	0.048	0.048	0.048	0.048	

2.494	2.582	2.398	0.150									T2/T3 1 hd
2.782	2.728	2.593	0.173									
2.495	2.513	2.338	0.142									
2.150	2.192	1.796	0.107									
1.421	2.199	2.389	0.045									
1.769	1.961	1.354	0.094									
						0.049	0.048	0.048	0.049	0.049	0.049	
						0.049	0.048	0.051	0.050	0.049	0.049	

				0.506	0.519	0.476	0.492	0.488	0.493	0.093	0.079	T2/T3 2 hd
				0.618	0.641	0.569	0.559	0.545	0.530	0.074	0.075	
				0.509	0.518	0.600	0.610	0.451	0.451	0.079	0.079	
				0.374	0.374	0.407	0.402	0.280	0.279	0.065	0.066	
				0.169	0.170	0.393	0.405	0.376	0.386			
				0.293	0.279	0.365	0.371	0.193	0.200	0.062	0.061	
						0.050	0.050	0.048	0.050	0.050	0.050	
						0.050	0.049	0.051	0.050	0.050	0.050	

1.270	1.342	0.806	0.077									T4/T5 1 hd
0.662	0.475	1.174	0.072									
1.243	0.724	1.016	0.068									
0.492	1.057	0.783	0.065									
0.349	1.129	0.473	0.064									
0.675	0.138	0.118	0.056									
						0.049	0.048	0.049	0.049	0.049	0.048	
						0.049	0.049	0.049	0.051	0.048	0.048	

				0.447	0.456	0.457	0.463	0.586	0.600	0.072	0.072	T4/T5 2 hd
				0.284	0.281	0.290	0.292	0.692	0.864	0.065	0.065	
				0.415	0.429	0.475	0.532	0.663	0.665	0.061	0.061	
				0.393	0.384	0.612	0.607	0.359	0.425	0.059	0.058	
				0.184	0.278	0.529	0.715	0.218	0.232	0.059	0.059	
				0.496	0.489	0.103	0.201	0.106	0.120	0.054	0.054	
						0.050	0.050	0.051	0.050	0.050	0.050	
						0.050	0.050	0.050	0.048	0.049	0.049	

0.505	0.546	0.291	0.060									T6/T7 1 hd
0.125	0.436	0.461	0.061									
0.473	0.159	0.127	0.057									
0.542	0.422	0.111	0.055									
0.081	0.114	0.133	0.055									
0.331	0.117	0.142	0.054									
						0.048	0.049	0.049	0.049	0.050	0.050	
						0.049	0.049	0.049	0.044	0.050	0.048	

				0.512	0.468	0.489	0.547	0.192	0.205	0.062	0.070	T6/T7 2 hd
				0.154	0.226	0.460	0.263	0.214	0.401	0.060	0.059	
				0.396	0.411	0.166	0.172	0.128	0.135	0.056	0.060	
				0.443	0.450	0.369	0.418	0.111	0.107	0.057	0.060	
				0.064	0.062	0.091	0.158	0.098	0.156	0.057	0.058	
				0.243	0.304	0.097	0.142	0.139	0.137	0.055	0.054	
						0.049	0.052	0.052	0.051	0.052	0.051	
						0.050	0.050	0.050	0.045	0.051	0.050	

**Project number**		F-120	**Apparatus**	**Wallac Victor**	**Operator**	**IsBa**
**GLP Study (Number)**		n.a.	**Protocol (Instrument method**	**LDH test 2016**	**Date of preparation**	**19-04-18**
**hot substance**	**isotope**	n.a.	**File name (results)**	**IsBa_180419/20_LDH_full_va**	**Date of measurement**	**19-04-18**
	**name**	n.a.	**Kind of well plate**	**96 well**	**shaking time [min]**	**30**
	**ACB-ID**	n.a.	**sample volume [µL]**	**100**	**stirring rate (Target) [rpm]**	**150**
	**Batch number**	n.a.	**Cocktail volume [µL]**	**175**	**Kind of measurement**	**UV-vis**
**cold substance**	**name**	LDH	**ACB-ID of cocktail**		**Wave length [nm]**	**450**
	**ACB-ID**	L23982R	**Matrix**	**DMEM (from powder)+PEG**	**Remarks**	**Cocktail 100µl RM, 75µl STOP**
	**Batch number**	SLBK6345V	**Blank description**	**DMEM/PEG, H2O**	**Remarks**	**7 standards split low/high**
**n.a.**		n.a.	**Pipettes (No. / volume)**	**50-200µl**	**Remarks**	**KLP4 common for both**
**n.a.**		n.a.	**Pipettes (No. / volume)**	**n.a.**	**Remarks**	**n.a.**

Messdaten (diese Tabelle in Bericht übernehmen)

Sample name *	concentration (theor.) *	measured data	measured data	measured data	mean measured	SD	RSD	Blank *	measured data after * Blank subtraction	concentration (calc.) *	Deviation *	Residuen
	[µg/mL]	[AU]	[AU]	[AU]	[AU]	[AU]	[%]	[AU]		[µg/mL]	[%]	
KLP1								0.047				
KLP2								0.054				
KLP3								0.051				
KLP4	0.041	0.959	0.972	0.883	0.938	0.04	4.19	0.051	0.887	0.041	-0.57	0.00
KLP5	0.018	0.483	0.484	0.474	0.480	0.00	0.98	0.052	0.429	0.019	2.42	0.00
KLP6	0.012	0.360	0.359	0.326	0.348	0.02	4.54	0.051	0.297	0.013	5.00	0.00
KLP7	0.007	0.228	0.228	0.199	0.218	0.014	6.35		0.168	0.006	-11.29	0.00
KLP8												

Statistical data

Geradensteigung	Slope	m	21.03
Y-Achsenabschnitt	Y-Intercept	b	0.03
Standardabw. Geradensteigung	SD-Slope	s_m	0.648055501
Standardabw. Achsenabschnittes	SD-Y-Intercept	s_b	0.015177452
Anzahl Messpunkte	number of measuring points	n	4
Quadratsumme	sum of squares	Qxx	0.000664048
Bereichsmitte			0.019557193
Freiheitsgrade	degree of freedom	f	2
Student-t-Faktor für (P = 95 %; f = n-2)	Student-t-factor for (P = 95 %; f = n-2)	t	4.303
Vertrauensbereich Steig. (95 %) Obergrenze		$m + VB_m$	23.81763508
Vertrauensbereich Steig. (95 %) Untergrenze		$m - VB_m$	18.24046944
Vertrauensbereich Achsenabschnitt (95 %) Og.		$b + Vb_b$	0.099335829
Vertrauensbereich Achsenabschnitt (95 %) Ug.		$b - Vb_b$	-0.031281327
Korrelationskoeffizient	correlation coefficient	r	0.9991
Bestimmtheitsmaß	determiantion coefficient	r^2	0.9981
Reststandardabweichung		s_0	0.01669983
Summe Restquadrate		sd	0.657385838
Verfahrensstandardabw.		s_{x0}	0.000794131
Rel. Verfahrensstandardabw. %		V_{x0}	4.060559029

Berichten

mean Blank	**0**	
SD Blank	0.00	
RSD Blank	3.79	x=
x*SD (LLOQ)	0.01	5
x*SD (LOD)	0.01	3
**LLOQ (AU)**	**0.061**	
**LOD (AU)**	**0.057**	
ULOQ	0.887	
LLOQ (Lin)	0.168	

Evaluation / Comment

LDH linearity valid with 7 standards, split in lin high and lin low, each with 4 standards and KLP4 common standard for both. R² 0,9981, deviations for both between -13.34% and +13.04%

Date Operator Date Control

Across Barriers GmbH
Science Park 1
66123 Saarbrücken

Tel: 0681 95918-800
Fax: 0681 95918-802
e-Mail: sales@acrossbarriers.de
http://www.acrossbarriers.de

Formblatt-Nr.		Version	
Titel			
Gültig ab		Ablage	Projektordner

Figure 1 Linearity

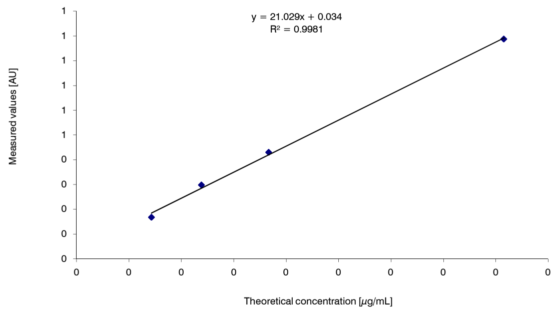

Figure 2 Method validation Residuen Plot

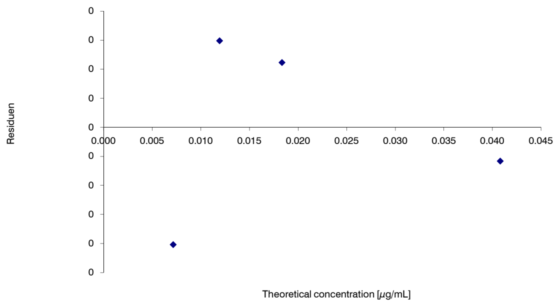

Evaluation / Comment

lin passed

Date	Operator	Date	Control

Sample name	Plate	Position	[AU]	[AU]-Blank	Rating	[µg/mL]	Dilution factor	[µg/mL]
0	T8/T9	A01	0	0	kleiner LLOQ	#VALUE!	1	#VALUE!
0	T8/T9	A02	0	0	kleiner LLOQ	#VALUE!	1	#VALUE!
0	T8/T9	A03	0	0	kleiner LLOQ	#VALUE!	1	#VALUE!
0	T8/T9	A04	0	0	kleiner LLOQ	#VALUE!	1	#VALUE!
T8KltN1	T8/T9	A05	0.742	0.691	0.691	0.031	2.75	0.09
T8KltN1	T8/T9	A06	0.708	0.657	0.657	0.030	2.75	0.08
T8KltN2	T8/T9	A07	0.415	0.364	0.364	0.016	2.75	0.04
T8KltN2	T8/T9	A08	0.444	0.393	0.393	0.017	2.75	0.047
T8KltN3	T8/T9	A09	0.158	0.107	kleiner LLOQ	#VALUE!	2.75	#VALUE!
T8KltN3	T8/T9	A10	0.154	0.103	kleiner LLOQ	#VALUE!	2.75	#VALUE!
T8KltN4	T8/T9	A11	0.055	0.004	kleiner LLOQ	#VALUE!	2.75	#VALUE!
T8KltN4	T8/T9	A12	0.055	0.004	kleiner LLOQ	#VALUE!	2.75	#VALUE!
0	T8/T9	B01	0	0	kleiner LLOQ	#VALUE!	1	#VALUE!
0	T8/T9	B02	0	0	kleiner LLOQ	#VALUE!	1	#VALUE!
0	T8/T9	B03	0	0	kleiner LLOQ	#VALUE!	1	#VALUE!
0	T8/T9	B04	0	0	kleiner LLOQ	#VALUE!	1	#VALUE!
T8KltS11	T8/T9	B05	0.063	0.012	kleiner LLOQ	#VALUE!	2.75	#VALUE!
T8KltS11	T8/T9	B06	0.064	0.013	kleiner LLOQ	#VALUE!	2.75	#VALUE!
T8KltS12	T8/T9	B07	0.221	0.170	0.170	0.006	2.75	0.02
T8KltS12	T8/T9	B08	0.273	0.222	0.222	0.009	2.75	0.02
T8KltS13	T8/T9	B09	0.109	0.058	kleiner LLOQ	#VALUE!	2.75	#VALUE!
T8KltS13	T8/T9	B10	0.217	0.166	kleiner LLOQ	#VALUE!	2.75	#VALUE!
T8KltS14	T8/T9	B11	0.074	0.023	kleiner LLOQ	#VALUE!	2.75	#VALUE!
T8KltS14	T8/T9	B12	0.061	0.010	kleiner LLOQ	#VALUE!	2.75	#VALUE!
0	T8/T9	C01	0	0	kleiner LLOQ	#VALUE!	1	#VALUE!
0	T8/T9	C02	0	0	kleiner LLOQ	#VALUE!	1	#VALUE!
0	T8/T9	C03	0	0	kleiner LLOQ	#VALUE!	1	#VALUE!
0	T8/T9	C04	0	0	kleiner LLOQ	#VALUE!	1	#VALUE!
T8KltS21	T8/T9	C05	0.346	0.295	0.295	0.012	2.75	0.03
T8KltS21	T8/T9	C06	0.348	0.297	0.297	0.012	2.75	0.03
T8KltS22	T8/T9	C07	0.086	0.035	kleiner LLOQ	#VALUE!	2.75	#VALUE!
T8KltS22	T8/T9	C08	0.090	0.039	kleiner LLOQ	#VALUE!	2.75	#VALUE!
T8KltS23	T8/T9	C09	0.202	0.151	kleiner LLOQ	#VALUE!	2.75	#VALUE!
T8KltS23	T8/T9	C10	0.149	0.098	kleiner LLOQ	#VALUE!	2.75	#VALUE!
T8KltS24	T8/T9	C11	0.061	0.010	kleiner LLOQ	#VALUE!	2.75	#VALUE!
T8KltS24	T8/T9	C12	0.054	0.003	kleiner LLOQ	#VALUE!	2.75	#VALUE!
0	T8/T9	D01	0	0	kleiner LLOQ	#VALUE!	1	#VALUE!
0	T8/T9	D02	0	0	kleiner LLOQ	#VALUE!	1	#VALUE!
0	T8/T9	D03	0	0	kleiner LLOQ	#VALUE!	1	#VALUE!
0	T8/T9	D04	0	0	kleiner LLOQ	#VALUE!	1	#VALUE!
T9KltN1	T8/T9	D05	0.692	0.641	0.641	0.029	2.75	0.08
T9KltN1	T8/T9	D06	0.579	0.528	0.528	0.023	2.75	0.06
T9KltN2	T8/T9	D07	0.503	0.453	0.453	0.020	2.75	0.05
T9KltN2	T8/T9	D08	0.503	0.452	0.452	0.020	2.75	0.05
T9KltN3	T8/T9	D09	0.249	0.198	0.198	0.008	2.75	0.02
T9KltN3	T8/T9	D10	0.260	0.209	0.209	0.008	2.75	0.02
T9KltN4	T8/T9	D11	0.146	0.095	kleiner LLOQ	#VALUE!	2.75	#VALUE!
T9KltN4	T8/T9	D12	0.144	0.093	kleiner LLOQ	#VALUE!	2.75	#VALUE!

Plate:	T8/T9

	Test 1	Test 2	Test 3	Test 4	5	6	7	8	9	10	11	12
A					0.742	0.708	0.415	0.444	0.158	0.154	0.055	0.055
B					0.063	0.064	0.221	0.273	0.109	0.217	0.074	0.061
C					0.346	0.348	0.086	0.090	0.202	0.149	0.061	0.054
D					0.692	0.579	0.503	0.503	0.249	0.260	0.146	0.144
E					0.037	0.159	0.164	0.211	0.209	0.141	0.109	0.251
F					0.255	0.243	0.076	0.078	0.233	0.242	0.387	0.318
G							0.050	0.043	0.047	0.050	0.049	0.047
H							0.042	0.045	0.041	0.040	0.040	0.047

Plattenbelegung

	1	2	3	4	5	6	7	8	9	10	11	12
A					T8KltN1	T8KltN1	T8KltN2	T8KltN2	T8KltN3	T8KltN3	T8KltN4	T8KltN4
B					T8KltS11	T8KltS11	T8KltS12	T8KltS12	T8KltS13	T8KltS13	T8KltS14	T8KltS14
C					T8KltS21	T8KltS21	T8KltS22	T8KltS22	T8KltS23	T8KltS23	T8KltS24	T8KltS24
D					T9KltN1	T9KltN1	T9KltN2	T9KltN2	T9KltN3	T9KltN3	T9KltN4	T9KltN4
E					T9KltS11	T9KltS11	T9KltS12	T9KltS12	T9KltS13	T9KltS13	T9KltS14	T9KltS14
F					T9KltS21	T9KltS21	T9KltS22	T9KltS22	T9KltS23	T9KltS23	T9KltS24	T9KltS24
G							Blank PEG	Blank PEG	Blank PEG	Blank PEG	Blank PEG	Blank PEG
H							Blank no PEG	Blank no PEG	Blank no PEG	Blank no PEG	Blank no PEG	Blank no PEG

Samples aufgeteilt in lin high und low:höher konzentrierte Samples bei lin high zu finden.

Comment:

erste messung verwendet

Date Operator

Date Control

0	T8/T9	E01	0	0	kleiner LLOQ	#VALUE!	1	#VALUE!
0	T8/T9	E02	0	0	kleiner LLOQ	#VALUE!	1	#VALUE!
0	T8/T9	E03	0	0	kleiner LLOQ	#VALUE!	1	#VALUE!
0	T8/T9	E04	0	0	kleiner LLOQ	#VALUE!	1	#VALUE!
T9KltS11	T8/T9	E05	0.037	-0.014	kleiner LLOQ	#VALUE!	2.75	#VALUE!
T9KltS11	T8/T9	E06	0.159	0.108	kleiner LLOQ	#VALUE!	2.75	#VALUE!
T9KltS12	T8/T9	E07	0.164	0.113	kleiner LLOQ	#VALUE!	2.75	#VALUE!
T9KltS12	T8/T9	E08	0.211	0.160	kleiner LLOQ	#VALUE!	2.75	#VALUE!
T9KltS13	T8/T9	E09	0.209	0.158	kleiner LLOQ	#VALUE!	2.75	#VALUE!
T9KltS13	T8/T9	E10	0.141	0.090	kleiner LLOQ	#VALUE!	2.75	#VALUE!
T9KltS14	T8/T9	E11	0.109	0.058	kleiner LLOQ	#VALUE!	2.75	#VALUE!
T9KltS14	T8/T9	E12	0.251	0.200	0.200	0.008	2.75	0.02
0	T8/T9	F01	0	0	kleiner LLOQ	#VALUE!	1	#VALUE!
0	T8/T9	F02	0	0	kleiner LLOQ	#VALUE!	1	#VALUE!
0	T8/T9	F03	0	0	kleiner LLOQ	#VALUE!	1	#VALUE!
0	T8/T9	F04	0	0	kleiner LLOQ	#VALUE!	1	#VALUE!
T9KltS21	T8/T9	F05	0.255	0.204	0.204	0.008	2.75	0.02
T9KltS21	T8/T9	F06	0.243	0.192	0.192	0.008	2.75	0.02
T9KltS22	T8/T9	F07	0.076	0.026	kleiner LLOQ	#VALUE!	2.75	#VALUE!
T9KltS22	T8/T9	F08	0.078	0.028	kleiner LLOQ	#VALUE!	2.75	#VALUE!
T9KltS23	T8/T9	F09	0.233	0.182	0.182	0.007	2.75	0.02
T9KltS23	T8/T9	F10	0.242	0.191	0.191	0.007	2.75	0.02
T9KltS24	T8/T9	F11	0.387	0.336	0.336	0.014	2.75	0.04
T9KltS24	T8/T9	F12	0.318	0.268	0.268	0.011	2.75	0.03
0	T8/T9	G01	0	0	kleiner LLOQ	#VALUE!	1	#VALUE!
0	T8/T9	G02	0	0	kleiner LLOQ	#VALUE!	1	#VALUE!
0	T8/T9	G03	0	0	kleiner LLOQ	#VALUE!	1	#VALUE!
0	T8/T9	G04	0	0	kleiner LLOQ	#VALUE!	1	#VALUE!
0	T8/T9	G05	0	0	kleiner LLOQ	#VALUE!	1	#VALUE!
0	T8/T9	G06	0	0	kleiner LLOQ	#VALUE!	1	#VALUE!
Blank PEG	T8/T9	G07	0	0	kleiner LLOQ	#VALUE!	2.75	#VALUE!
Blank PEG	T8/T9	G08	0	0	kleiner LLOQ	#VALUE!	2.75	#VALUE!
Blank PEG	T8/T9	G09	0	0	kleiner LLOQ	#VALUE!	2.75	#VALUE!
Blank PEG	T8/T9	G10	0	0	kleiner LLOQ	#VALUE!	2.75	#VALUE!
Blank PEG	T8/T9	G11	0	0	kleiner LLOQ	#VALUE!	2.75	#VALUE!
Blank PEG	T8/T9	G12	0	0	kleiner LLOQ	#VALUE!	2.75	#VALUE!
0	T8/T9	H01	0	0	kleiner LLOQ	#VALUE!	1	#VALUE!
0	T8/T9	H02	0	0	kleiner LLOQ	#VALUE!	1	#VALUE!
0	T8/T9	H03	0	0	kleiner LLOQ	#VALUE!	1	#VALUE!
0	T8/T9	H04	0	0	kleiner LLOQ	#VALUE!	1	#VALUE!
0	T8/T9	H05	0	0	kleiner LLOQ	#VALUE!	1	#VALUE!
0	T8/T9	H06	0	0	kleiner LLOQ	#VALUE!	1	#VALUE!
Blank no PEG	T8/T9	H07	0	0	kleiner LLOQ	#VALUE!	2.75	#VALUE!
Blank no PEG	T8/T9	H08	0	0	kleiner LLOQ	#VALUE!	2.75	#VALUE!
Blank no PEG	T8/T9	H09	0	0	kleiner LLOQ	#VALUE!	2.75	#VALUE!
Blank no PEG	T8/T9	H10	0	0	kleiner LLOQ	#VALUE!	2.75	#VALUE!
Blank no PEG	T8/T9	H11	0	0	kleiner LLOQ	#VALUE!	2.75	#VALUE!
Blank no PEG	T8/T9	H12	0	0	kleiner LLOQ	#VALUE!	2.75	#VALUE!

0	T10/T11	A01	0	0	kleiner LLOQ	#VALUE!	1	#VALUE!
0	T10/T11	A02	0	0	kleiner LLOQ	#VALUE!	1	#VALUE!
0	T10/T11	A03	0	0	kleiner LLOQ	#VALUE!	1	#VALUE!
0	T10/T11	A04	0	0	kleiner LLOQ	#VALUE!	1	#VALUE!
T10KltN1	T10/T11	A05	0.598	0.547	0.547	0.024	2.75	0.067
T10KltN1	T10/T11	A06	0.575	0.525	0.525	0.023	2.75	0.064
T10KltN2	T10/T11	A07	0.524	0.473	0.473	0.021	2.75	0.057
T10KltN2	T10/T11	A08	0.523	0.472	0.472	0.021	2.75	0.057
T10KltN3	T10/T11	A09	0.235	0.184	0.184	0.007	2.75	0.020
T10KltN3	T10/T11	A10	0.225	0.174	0.174	0.007	2.75	0.018
T10KltN4	T10/T11	A11	0.117	0.066	kleiner LLOQ	#VALUE!	2.75	#VALUE!
T10KltN4	T10/T11	A12	0.106	0.055	kleiner LLOQ	#VALUE!	2.75	#VALUE!
0	T10/T11	B01	0	0	kleiner LLOQ	#VALUE!	1	#VALUE!
0	T10/T11	B02	0	0	kleiner LLOQ	#VALUE!	1	#VALUE!
0	T10/T11	B03	0	0	kleiner LLOQ	#VALUE!	1	#VALUE!
0	T10/T11	B04	0	0	kleiner LLOQ	#VALUE!	1	#VALUE!
T10KltS11	T10/T11	B05	0.115	0.064	kleiner LLOQ	#VALUE!	2.75	#VALUE!
T10KltS11	T10/T11	B06	0.109	0.058	kleiner LLOQ	#VALUE!	2.75	#VALUE!
T10KltS12	T10/T11	B07	0.177	0.126	kleiner LLOQ	#VALUE!	2.75	#VALUE!
T10KltS12	T10/T11	B08	0.191	0.140	kleiner LLOQ	#VALUE!	2.75	#VALUE!
T10KltS13	T10/T11	B09	0.110	0.059	kleiner LLOQ	#VALUE!	2.75	#VALUE!
T10KltS13	T10/T11	B10	0.109	0.058	kleiner LLOQ	#VALUE!	2.75	#VALUE!
T10KltS14	T10/T11	B11	0.098	0.047	kleiner LLOQ	#VALUE!	2.75	#VALUE!
T10KltS14	T10/T11	B12	0.090	0.039	kleiner LLOQ	#VALUE!	2.75	#VALUE!
0	T10/T11	C01	0	0	kleiner LLOQ	#VALUE!	1	#VALUE!
0	T10/T11	C02	0	0	kleiner LLOQ	#VALUE!	1	#VALUE!
0	T10/T11	C03	0	0	kleiner LLOQ	#VALUE!	1	#VALUE!
0	T10/T11	C04	0	0	kleiner LLOQ	#VALUE!	1	#VALUE!
T10KltS21	T10/T11	C05	0.149	0.098	kleiner LLOQ	#VALUE!	2.75	#VALUE!
T10KltS21	T10/T11	C06	0.152	0.101	kleiner LLOQ	#VALUE!	2.75	#VALUE!
T10KltS22	T10/T11	C07	0.190	0.139	kleiner LLOQ	#VALUE!	2.75	#VALUE!
T10KltS22	T10/T11	C08	0.196	0.145	kleiner LLOQ	#VALUE!	2.75	#VALUE!
T10KltS23	T10/T11	C09	0.211	0.160	kleiner LLOQ	#VALUE!	2.75	#VALUE!
T10KltS23	T10/T11	C10	0.205	0.154	kleiner LLOQ	#VALUE!	2.75	#VALUE!
T10KltS24	T10/T11	C11	0.070	0.019	kleiner LLOQ	#VALUE!	2.75	#VALUE!
T10KltS24	T10/T11	C12	0.078	0.027	kleiner LLOQ	#VALUE!	2.75	#VALUE!
0	T10/T11	D01	0	0	kleiner LLOQ	#VALUE!	1	#VALUE!
0	T10/T11	D02	0	0	kleiner LLOQ	#VALUE!	1	#VALUE!
0	T10/T11	D03	0	0	kleiner LLOQ	#VALUE!	1	#VALUE!
0	T10/T11	D04	0	0	kleiner LLOQ	#VALUE!	1	#VALUE!
T11KltN1	T10/T11	D05	0.648	0.597	0.597	0.027	2.75	0.074
T11KltN1	T10/T11	D06	0.647	0.596	0.596	0.027	2.75	0.074
T11KltN2	T10/T11	D07	0.729	0.678	0.678	0.031	2.75	0.084
T11KltN2	T10/T11	D08	0.693	0.642	0.642	0.029	2.75	0.080
T11KltN3	T10/T11	D09	0.275	0.224	0.224	0.009	2.75	0.025
T11KltN3	T10/T11	D10	0.219	0.168	kleiner LLOQ	#VALUE!	2.75	#VALUE!
T11KltN4	T10/T11	D11	0.151	0.100	kleiner LLOQ	#VALUE!	2.75	#VALUE!
T11KltN4	T10/T11	D12	0.153	0.102	kleiner LLOQ	#VALUE!	2.75	#VALUE!

Plate: T10/T11

	1	2	3	4	5	6	7	8	9	10	11	12
A					0.598	0.575	0.524	0.523	0.235	0.225	0.117	0.106
B					0.115	0.109	0.177	0.191	0.110	0.109	0.098	0.090
C					0.149	0.152	0.190	0.196	0.211	0.205	0.070	0.078
D					0.648	0.647	0.729	0.693	0.275	0.219	0.151	0.153
E					0.101	0.096	0.178	0.181	0.092	0.087	0.089	0.092
F					0.252	0.256	0.176	0.176	0.223	0.221	0.069	0.068
G							0.044	0.043	0.046	0.047	0.044	0.044
H							0.041	0.041	0.042	0.042	0.041	0.042

Plattenbelegung

	1	2	3	4	5	6	7	8	9	10	11	12
A					T10KltN1	T10KltN1	T10KltN2	T10KltN2	T10KltN3	T10KltN3	T10KltN4	T10KltN4
B					T10KltS11	T10KltS11	T10KltS12	T10KltS12	T10KltS13	T10KltS13	T10KltS14	T10KltS14
C					T10KltS21	T10KltS21	T10KltS22	T10KltS22	T10KltS23	T10KltS23	T10KltS24	T10KltS24
D					T11KltN1	T11KltN1	T11KltN2	T11KltN2	T11KltN3	T11KltN3	T11KltN4	T11KltN4
E					T11KltS11	T11KltS11	T11KltS12	T11KltS12	T11KltS13	T11KltS13	T11KltS14	T11KltS14
F					T11KltS21	T11KltS21	T11KltS22	T11KltS22	T11KltS23	T11KltS23	T11KltS24	T11KltS24
G							Blank PEG	Blank PEG	Blank PEG	Blank PEG	Blank PEG	Blank PEG
H							Blank no PEG	Blank no PEG	Blank no PEG	Blank no PEG	Blank no PEG	Blank no PEG

Comment:

Date Operator

Date Control

0	T10/T11	E01	0	0	kleiner LLOQ	#VALUE!	1	#VALUE!
0	T10/T11	E02	0	0	kleiner LLOQ	#VALUE!	1	#VALUE!
0	T10/T11	E03	0	0	kleiner LLOQ	#VALUE!	1	#VALUE!
0	T10/T11	E04	0	0	kleiner LLOQ	#VALUE!	1	#VALUE!
T11KltS11	T10/T11	E05	0.101	0.050	kleiner LLOQ	#VALUE!	2.75	#VALUE!
T11KltS11	T10/T11	E06	0.096	0.045	kleiner LLOQ	#VALUE!	2.75	#VALUE!
T11KltS12	T10/T11	E07	0.178	0.127	kleiner LLOQ	#VALUE!	2.75	#VALUE!
T11KltS12	T10/T11	E08	0.181	0.131	kleiner LLOQ	#VALUE!	2.75	#VALUE!
T11KltS13	T10/T11	E09	0.092	0.041	kleiner LLOQ	#VALUE!	2.75	#VALUE!
T11KltS13	T10/T11	E10	0.087	0.036	kleiner LLOQ	#VALUE!	2.75	#VALUE!
T11KltS14	T10/T11	E11	0.089	0.038	kleiner LLOQ	#VALUE!	2.75	#VALUE!
T11KltS14	T10/T11	E12	0.092	0.041	kleiner LLOQ	#VALUE!	2.75	#VALUE!
0	T10/T11	F01	0	0	kleiner LLOQ	#VALUE!	1	#VALUE!
0	T10/T11	F02	0	0	kleiner LLOQ	#VALUE!	1	#VALUE!
0	T10/T11	F03	0	0	kleiner LLOQ	#VALUE!	1	#VALUE!
0	T10/T11	F04	0	0	kleiner LLOQ	#VALUE!	1	#VALUE!
T11KltS21	T10/T11	F05	0.252	0.202	0.202	0.008	2.75	0.022
T11KltS21	T10/T11	F06	0.256	0.205	0.205	0.008	2.75	0.022
T11KltS22	T10/T11	F07	0.176	0.125	kleiner LLOQ	#VALUE!	2.75	#VALUE!
T11KltS22	T10/T11	F08	0.176	0.126	kleiner LLOQ	#VALUE!	2.75	#VALUE!
T11KltS23	T10/T11	F09	0.223	0.172	0.172	0.007	2.75	0.018
T11KltS23	T10/T11	F10	0.221	0.170	0.170	0.006	2.75	0.018
T11KltS24	T10/T11	F11	0.069	0.018	kleiner LLOQ	#VALUE!	2.75	#VALUE!
T11KltS24	T10/T11	F12	0.068	0.017	kleiner LLOQ	#VALUE!	2.75	#VALUE!
0	T10/T11	G01	0	0	kleiner LLOQ	#VALUE!	1	#VALUE!
0	T10/T11	G02	0	0	kleiner LLOQ	#VALUE!	1	#VALUE!
0	T10/T11	G03	0	0	kleiner LLOQ	#VALUE!	1	#VALUE!
0	T10/T11	G04	0	0	kleiner LLOQ	#VALUE!	1	#VALUE!
0	T10/T11	G05	0	0	kleiner LLOQ	#VALUE!	1	#VALUE!
0	T10/T11	G06	0	0	kleiner LLOQ	#VALUE!	1	#VALUE!
Blank PEG	T10/T11	G07	0	0	kleiner LLOQ	#VALUE!	2.75	#VALUE!
Blank PEG	T10/T11	G08	0	0	kleiner LLOQ	#VALUE!	2.75	#VALUE!
Blank PEG	T10/T11	G09	0	0	kleiner LLOQ	#VALUE!	2.75	#VALUE!
Blank PEG	T10/T11	G10	0	0	kleiner LLOQ	#VALUE!	2.75	#VALUE!
Blank PEG	T10/T11	G11	0	0	kleiner LLOQ	#VALUE!	2.75	#VALUE!
Blank PEG	T10/T11	G12	0	0	kleiner LLOQ	#VALUE!	2.75	#VALUE!
0	T10/T11	H01	0	0	kleiner LLOQ	#VALUE!	1	#VALUE!
0	T10/T11	H02	0	0	kleiner LLOQ	#VALUE!	1	#VALUE!
0	T10/T11	H03	0	0	kleiner LLOQ	#VALUE!	1	#VALUE!
0	T10/T11	H04	0	0	kleiner LLOQ	#VALUE!	1	#VALUE!
0	T10/T11	H05	0	0	kleiner LLOQ	#VALUE!	1	#VALUE!
0	T10/T11	H06	0	0	kleiner LLOQ	#VALUE!	1	#VALUE!
Blank no PEG	T10/T11	H07	0	0	kleiner LLOQ	#VALUE!	2.75	#VALUE!
Blank no PEG	T10/T11	H08	0	0	kleiner LLOQ	#VALUE!	2.75	#VALUE!
Blank no PEG	T10/T11	H09	0	0	kleiner LLOQ	#VALUE!	2.75	#VALUE!
Blank no PEG	T10/T11	H10	0	0	kleiner LLOQ	#VALUE!	2.75	#VALUE!
Blank no PEG	T10/T11	H11	0	0	kleiner LLOQ	#VALUE!	2.75	#VALUE!
Blank no PEG	T10/T11	H12	0	0	kleiner LLOQ	#VALUE!	2.75	#VALUE!
0	T12/T13	A01	0	0	kleiner LLOQ	#VALUE!	1	#VALUE!
0	T12/T13	A02	0	0	kleiner LLOQ	#VALUE!	1	#VALUE!
0	T12/T13	A03	0	0	kleiner LLOQ	#VALUE!	1	#VALUE!
0	T12/T13	A04	0	0	kleiner LLOQ	#VALUE!	1	#VALUE!
T12KltN1	T12/T13	A05	0.670	0.619	0.619	0.028	2.75	0.076
T12KltN1	T12/T13	A06	0.663	0.612	0.612	0.027	2.75	0.076
T12KltN2	T12/T13	A07	0.524	0.473	0.473	0.021	2.75	0.057
T12KltN2	T12/T13	A08	0.538	0.487	0.487	0.022	2.75	0.059
T12KltN3	T12/T13	A09	0.187	0.136	kleiner LLOQ	#VALUE!	2.75	#VALUE!
T12KltN3	T12/T13	A10	0.184	0.133	kleiner LLOQ	#VALUE!	2.75	#VALUE!
T12KltN4	T12/T13	A11	0.111	0.061	kleiner LLOQ	#VALUE!	2.75	#VALUE!
T12KltN4	T12/T13	A12	0.107	0.056	kleiner LLOQ	#VALUE!	2.75	#VALUE!
0	T12/T13	B01	0	0	kleiner LLOQ	#VALUE!	1	#VALUE!
0	T12/T13	B02	0	0	kleiner LLOQ	#VALUE!	1	#VALUE!
0	T12/T13	B03	0	0	kleiner LLOQ	#VALUE!	1	#VALUE!
0	T12/T13	B04	0	0	kleiner LLOQ	#VALUE!	1	#VALUE!
T12KltS11	T12/T13	B05	0.065	0.014	kleiner LLOQ	#VALUE!	2.75	#VALUE!
T12KltS11	T12/T13	B06	0.066	0.015	kleiner LLOQ	#VALUE!	2.75	#VALUE!
T12KltS12	T12/T13	B07	0.111	0.060	kleiner LLOQ	#VALUE!	2.75	#VALUE!
T12KltS12	T12/T13	B08	0.113	0.062	kleiner LLOQ	#VALUE!	2.75	#VALUE!
T12KltS13	T12/T13	B09	0.070	0.019	kleiner LLOQ	#VALUE!	2.75	#VALUE!
T12KltS13	T12/T13	B10	0.069	0.018	kleiner LLOQ	#VALUE!	2.75	#VALUE!
T12KltS14	T12/T13	B11	0.061	0.011	kleiner LLOQ	#VALUE!	2.75	#VALUE!
T12KltS14	T12/T13	B12	0.062	0.011	kleiner LLOQ	#VALUE!	2.75	#VALUE!
0	T12/T13	C01	0	0	kleiner LLOQ	#VALUE!	1	#VALUE!
0	T12/T13	C02	0	0	kleiner LLOQ	#VALUE!	1	#VALUE!

Plate: T12/T13

	1	2	3	4	5	6	7	8	9	10	11	12
A					0.670	0.663	0.524	0.538	0.187	0.184	0.111	0.107
B					0.065	0.066	0.111	0.113	0.070	0.069	0.061	0.062
C					0.180	0.181	0.102	0.104	0.171	0.172	0.061	0.062
D					0.678	0.577	0.479	0.502	0.148	0.165	0.137	0.129
E					0.067	0.068	0.095	0.107	0.064	0.065	0.090	0.099
F					0.255	0.263	0.081	0.085	0.255	0.249	0.077	0.075
G							0.044	0.042	0.043	0.042	0.045	0.042
H							0.041	0.040	0.041	0.042	0.042	0.042

Plattenbelegung

	1	2	3	4	5	6	7	8	9	10	11	12
A					T12KltN1	T12KltN1	T12KltN2	T12KltN2	T12KltN3	T12KltN3	T12KltN4	T12KltN4
B					T12KltS11	T12KltS11	T12KltS12	T12KltS12	T12KltS13	T12KltS13	T12KltS14	T12KltS14
C					T12KltS21	T12KltS21	T12KltS22	T12KltS22	T12KltS23	T12KltS23	T12KltS24	T12KltS24
D					T13KltN1	T13KltN1	T13KltN2	T13KltN2	T13KltN3	T13KltN3	T13KltN4	T13KltN4
E					T13KltS11	T13KltS11	T13KltS12	T13KltS12	T13KltS13	T13KltS13	T13KltS14	T13KltS14
F					T13KltS21	T13KltS21	T13KltS22	T13KltS22	T13KltS23	T13KltS23	T13KltS24	T13KltS24

0	T12/T13	C03	0	0	kleiner LLOQ	#VALUE!	1	#VALUE!
0	T12/T13	C04	0	0	kleiner LLOQ	#VALUE!	1	#VALUE!
T12KltS21	T12/T13	C05	0.180	0.129	kleiner LLOQ	#VALUE!	2.75	#VALUE!
T12KltS21	T12/T13	C06	0.181	0.130	kleiner LLOQ	#VALUE!	2.75	#VALUE!
T12KltS22	T12/T13	C07	0.102	0.051	kleiner LLOQ	#VALUE!	2.75	#VALUE!
T12KltS22	T12/T13	C08	0.104	0.053	kleiner LLOQ	#VALUE!	2.75	#VALUE!
T12KltS23	T12/T13	C09	0.171	0.120	kleiner LLOQ	#VALUE!	2.75	#VALUE!
T12KltS23	T12/T13	C10	0.172	0.121	kleiner LLOQ	#VALUE!	2.75	#VALUE!
T12KltS24	T12/T13	C11	0.061	0.011	kleiner LLOQ	#VALUE!	2.75	#VALUE!
T12KltS24	T12/T13	C12	0.062	0.011	kleiner LLOQ	#VALUE!	2.75	#VALUE!
0	T12/T13	D01	0	0	kleiner LLOQ	#VALUE!	1	#VALUE!
0	T12/T13	D02	0	0	kleiner LLOQ	#VALUE!	1	#VALUE!
0	T12/T13	D03	0	0	kleiner LLOQ	#VALUE!	1	#VALUE!
0	T12/T13	D04	0	0	kleiner LLOQ	#VALUE!	1	#VALUE!
T13KltN1	T12/T13	D05	0.678	0.627	0.627	0.028	2.75	0.078
T13KltN1	T12/T13	D06	0.577	0.526	0.526	0.023	2.75	0.064
T13KltN2	T12/T13	D07	0.479	0.428	0.428	0.019	2.75	0.052
T13KltN2	T12/T13	D08	0.502	0.451	0.451	0.020	2.75	0.055
T13KltN3	T12/T13	D09	0.148	0.097	kleiner LLOQ	#VALUE!	2.75	#VALUE!
T13KltN3	T12/T13	D10	0.165	0.114	kleiner LLOQ	#VALUE!	2.75	#VALUE!
T13KltN4	T12/T13	D11	0.137	0.086	kleiner LLOQ	#VALUE!	2.75	#VALUE!
T13KltN4	T12/T13	D12	0.129	0.079	kleiner LLOQ	#VALUE!	2.75	#VALUE!
0	T12/T13	E01	0	0	kleiner LLOQ	#VALUE!	1	#VALUE!
0	T12/T13	E02	0	0	kleiner LLOQ	#VALUE!	1	#VALUE!
0	T12/T13	E03	0	0	kleiner LLOQ	#VALUE!	1	#VALUE!
0	T12/T13	E04	0	0	kleiner LLOQ	#VALUE!	1	#VALUE!
T13KltS11	T12/T13	E05	0.067	0.016	kleiner LLOQ	#VALUE!	2.75	#VALUE!
T13KltS11	T12/T13	E06	0.068	0.017	kleiner LLOQ	#VALUE!	2.75	#VALUE!
T13KltS12	T12/T13	E07	0.095	0.044	kleiner LLOQ	#VALUE!	2.75	#VALUE!
T13KltS12	T12/T13	E08	0.107	0.056	kleiner LLOQ	#VALUE!	2.75	#VALUE!
T13KltS13	T12/T13	E09	0.064	0.013	kleiner LLOQ	#VALUE!	2.75	#VALUE!
T13KltS13	T12/T13	E10	0.065	0.014	kleiner LLOQ	#VALUE!	2.75	#VALUE!
T13KltS14	T12/T13	E11	0.090	0.039	kleiner LLOQ	#VALUE!	2.75	#VALUE!
T13KltS14	T12/T13	E12	0.099	0.048	kleiner LLOQ	#VALUE!	2.75	#VALUE!
0	T12/T13	F01	0	0	kleiner LLOQ	#VALUE!	1	#VALUE!
0	T12/T13	F02	0	0	kleiner LLOQ	#VALUE!	1	#VALUE!
0	T12/T13	F03	0	0	kleiner LLOQ	#VALUE!	1	#VALUE!
0	T12/T13	F04	0	0	kleiner LLOQ	#VALUE!	1	#VALUE!
T13KltS21	T12/T13	F05	0.255	0.204	0.204	0.008	2.75	0.022
T13KltS21	T12/T13	F06	0.263	0.213	0.213	0.008	2.75	0.023
T13KltS22	T12/T13	F07	0.081	0.030	kleiner LLOQ	#VALUE!	2.75	#VALUE!
T13KltS22	T12/T13	F08	0.085	0.034	kleiner LLOQ	#VALUE!	2.75	#VALUE!
T13KltS23	T12/T13	F09	0.255	0.204	0.204	0.008	2.75	0.022
T13KltS23	T12/T13	F10	0.249	0.198	0.198	0.008	2.75	0.021
T13KltS24	T12/T13	F11	0.077	0.026	kleiner LLOQ	#VALUE!	2.75	#VALUE!
T13KltS24	T12/T13	F12	0.075	0.024	kleiner LLOQ	#VALUE!	2.75	#VALUE!
0	T12/T13	G01	0	0	kleiner LLOQ	#VALUE!	1	#VALUE!
0	T12/T13	G02	0	0	kleiner LLOQ	#VALUE!	1	#VALUE!
0	T12/T13	G03	0	0	kleiner LLOQ	#VALUE!	1	#VALUE!
0	T12/T13	G04	0	0	kleiner LLOQ	#VALUE!	1	#VALUE!
0	T12/T13	G05	0	0	kleiner LLOQ	#VALUE!	1	#VALUE!
0	T12/T13	G06	0	0	kleiner LLOQ	#VALUE!	1	#VALUE!
Blank PEG	T12/T13	G07	0	0	kleiner LLOQ	#VALUE!	2.75	#VALUE!
Blank PEG	T12/T13	G08	0	0	kleiner LLOQ	#VALUE!	2.75	#VALUE!
Blank PEG	T12/T13	G09	0	0	kleiner LLOQ	#VALUE!	2.75	#VALUE!
Blank PEG	T12/T13	G10	0	0	kleiner LLOQ	#VALUE!	2.75	#VALUE!
Blank PEG	T12/T13	G11	0	0	kleiner LLOQ	#VALUE!	2.75	#VALUE!
Blank PEG	T12/T13	G12	0	0	kleiner LLOQ	#VALUE!	2.75	#VALUE!
0	T12/T13	H01	0	0	kleiner LLOQ	#VALUE!	1	#VALUE!
0	T12/T13	H02	0	0	kleiner LLOQ	#VALUE!	1	#VALUE!
0	T12/T13	H03	0	0	kleiner LLOQ	#VALUE!	1	#VALUE!
0	T12/T13	H04	0	0	kleiner LLOQ	#VALUE!	1	#VALUE!
0	T12/T13	H05	0	0	kleiner LLOQ	#VALUE!	1	#VALUE!
0	T12/T13	H06	0	0	kleiner LLOQ	#VALUE!	1	#VALUE!
Blank no PEG	T12/T13	H07	0	0	kleiner LLOQ	#VALUE!	2.75	#VALUE!
Blank no PEG	T12/T13	H08	0	0	kleiner LLOQ	#VALUE!	2.75	#VALUE!
Blank no PEG	T12/T13	H09	0	0	kleiner LLOQ	#VALUE!	2.75	#VALUE!
Blank no PEG	T12/T13	H10	0	0	kleiner LLOQ	#VALUE!	2.75	#VALUE!
Blank no PEG	T12/T13	H11	0	0	kleiner LLOQ	#VALUE!	2.75	#VALUE!
Blank no PEG	T12/T13	H12	0	0	kleiner LLOQ	#VALUE!	2.75	#VALUE!
0	T14	A01	0	0	kleiner LLOQ	#VALUE!	1	#VALUE!
0	T14	A02	0	0	kleiner LLOQ	#VALUE!	1	#VALUE!
0	T14	A03	0	0	kleiner LLOQ	#VALUE!	1	#VALUE!
0	T14	A04	0	0	kleiner LLOQ	#VALUE!	1	#VALUE!
T14KltN1	T14	A05	0.736	0.685	0.685	0.031	2.75	0.085
T14KltN1	T14	A06	0.721	0.670	0.670	0.030	2.75	0.083

G							Blank PEG	Blank PEG	Blank PEG	Blank PEG	Blank PEG	Blank PEG
H							Blank no PEG	Blank no PEG	Blank no PEG	Blank no PEG	Blank no PEG	Blank no PEG

Comment:

Date Operator

Date Control

Plate:	T14

T14KltN2	T14	A07	0.619	0.568	0.568	0.025	2.75	0.070
T14KltN2	T14	A08	0.625	0.574	0.574	0.026	2.75	0.071
T14KltN3	T14	A09	0.122	0.071	kleiner LLOQ	#VALUE!	2.75	#VALUE!
T14KltN3	T14	A10	0.123	0.072	kleiner LLOQ	#VALUE!	2.75	#VALUE!
T14KltN4	T14	A11	0.140	0.089	kleiner LLOQ	#VALUE!	2.75	#VALUE!
T14KltN4	T14	A12	0.139	0.088	kleiner LLOQ	#VALUE!	2.75	#VALUE!
0	T14	B01	0	0	kleiner LLOQ	#VALUE!	1	#VALUE!
0	T14	B02	0	0	kleiner LLOQ	#VALUE!	1	#VALUE!
0	T14	B03	0	0	kleiner LLOQ	#VALUE!	1	#VALUE!
0	T14	B04	0	0	kleiner LLOQ	#VALUE!	1	#VALUE!
T14KltS11	T14	B05	0.064	0.013	kleiner LLOQ	#VALUE!	2.75	#VALUE!
T14KltS11	T14	B06	0.066	0.015	kleiner LLOQ	#VALUE!	2.75	#VALUE!
T14KltS12	T14	B07	0.066	0.015	kleiner LLOQ	#VALUE!	2.75	#VALUE!
T14KltS12	T14	B08	0.067	0.016	kleiner LLOQ	#VALUE!	2.75	#VALUE!
T14KltS13	T14	B09	0.096	0.046	kleiner LLOQ	#VALUE!	2.75	#VALUE!
T14KltS13	T14	B10	0.100	0.049	kleiner LLOQ	#VALUE!	2.75	#VALUE!
T14KltS14	T14	B11	0.099	0.048	kleiner LLOQ	#VALUE!	2.75	#VALUE!
T14KltS14	T14	B12	0.098	0.047	kleiner LLOQ	#VALUE!	2.75	#VALUE!
0	T14	C01	0	0	kleiner LLOQ	#VALUE!	1	#VALUE!
0	T14	C02	0	0	kleiner LLOQ	#VALUE!	1	#VALUE!
0	T14	C03	0	0	kleiner LLOQ	#VALUE!	1	#VALUE!
0	T14	C04	0	0	kleiner LLOQ	#VALUE!	1	#VALUE!
T14KltS21	T14	C05	0.062	0.011	kleiner LLOQ	#VALUE!	2.75	#VALUE!
T14KltS21	T14	C06	0.063	0.012	kleiner LLOQ	#VALUE!	2.75	#VALUE!
T14KltS22	T14	C07	0.074	0.023	kleiner LLOQ	#VALUE!	2.75	#VALUE!
T14KltS22	T14	C08	0.076	0.025	kleiner LLOQ	#VALUE!	2.75	#VALUE!
T14KltS23	T14	C09	0.240	0.190	0.190	0.007	2.75	0.020
T14KltS23	T14	C10	0.247	0.196	0.196	0.008	2.75	0.021
T14KltS24	T14	C11	0.093	0.042	kleiner LLOQ	#VALUE!	2.75	#VALUE!
T14KltS24	T14	C12	0.090	0.039	kleiner LLOQ	#VALUE!	2.75	#VALUE!
0	T14	D01	0	0	kleiner LLOQ	#VALUE!	1	#VALUE!
0	T14	D02	0	0	kleiner LLOQ	#VALUE!	1	#VALUE!
0	T14	D03	0	0	kleiner LLOQ	#VALUE!	1	#VALUE!
0	T14	D04	0	0	kleiner LLOQ	#VALUE!	1	#VALUE!
0	T14	D05	0	0	kleiner LLOQ	#VALUE!	1	#VALUE!
0	T14	D06	0	0	kleiner LLOQ	#VALUE!	1	#VALUE!
0	T14	D07	0	0	kleiner LLOQ	#VALUE!	1	#VALUE!
0	T14	D08	0	0	kleiner LLOQ	#VALUE!	1	#VALUE!
0	T14	D09	0	0	kleiner LLOQ	#VALUE!	1	#VALUE!
0	T14	D10	0	0	kleiner LLOQ	#VALUE!	1	#VALUE!
0	T14	D11	0	0	kleiner LLOQ	#VALUE!	1	#VALUE!
0	T14	D12		#VALUE!	#VALUE!	#VALUE!	1	#VALUE!
0	T14	E01	0	0	kleiner LLOQ	#VALUE!	1	#VALUE!
0	T14	E02	0	0	kleiner LLOQ	#VALUE!	1	#VALUE!
0	T14	E03	0	0	kleiner LLOQ	#VALUE!	1	#VALUE!
0	T14	E04	0	0	kleiner LLOQ	#VALUE!	1	#VALUE!
0	T14	E05	0	0	kleiner LLOQ	#VALUE!	1	#VALUE!
0	T14	E06	0	0	kleiner LLOQ	#VALUE!	1	#VALUE!
0	T14	E07	0	0	kleiner LLOQ	#VALUE!	1	#VALUE!
0	T14	E08	0	0	kleiner LLOQ	#VALUE!	1	#VALUE!
0	T14	E09	0	0	kleiner LLOQ	#VALUE!	1	#VALUE!
0	T14	E10	0	0	kleiner LLOQ	#VALUE!	1	#VALUE!
0	T14	E11	0	0	kleiner LLOQ	#VALUE!	1	#VALUE!
0	T14	E12		#VALUE!	#VALUE!	#VALUE!	1	#VALUE!
0	T14	F01	0	0	kleiner LLOQ	#VALUE!	1	#VALUE!
0	T14	F02	0	0	kleiner LLOQ	#VALUE!	1	#VALUE!
0	T14	F03	0	0	kleiner LLOQ	#VALUE!	1	#VALUE!
0	T14	F04	0	0	kleiner LLOQ	#VALUE!	1	#VALUE!
0	T14	F05	0	0	kleiner LLOQ	#VALUE!	1	#VALUE!
0	T14	F06	0	0	kleiner LLOQ	#VALUE!	1	#VALUE!
0	T14	F07	0	0	kleiner LLOQ	#VALUE!	1	#VALUE!
0	T14	F08	0	0	kleiner LLOQ	#VALUE!	1	#VALUE!
0	T14	F09	0	0	kleiner LLOQ	#VALUE!	1	#VALUE!
0	T14	F10	0	0	kleiner LLOQ	#VALUE!	1	#VALUE!
0	T14	F11	0	0	kleiner LLOQ	#VALUE!	1	#VALUE!
0	T14	F12		#VALUE!	#VALUE!	#VALUE!	1	#VALUE!
0	T14	G01	0	0	kleiner LLOQ	#VALUE!	1	#VALUE!
0	T14	G02	0	0	kleiner LLOQ	#VALUE!	1	#VALUE!
0	T14	G03	0	0	kleiner LLOQ	#VALUE!	1	#VALUE!
0	T14	G04	0	0	kleiner LLOQ	#VALUE!	1	#VALUE!
0	T14	G05	0	0	kleiner LLOQ	#VALUE!	1	#VALUE!
0	T14	G06	0	0	kleiner LLOQ	#VALUE!	1	#VALUE!
Blank PEG	T14	G07	0	0	kleiner LLOQ	#VALUE!	1	#VALUE!
Blank PEG	T14	G08	0	0	kleiner LLOQ	#VALUE!	1	#VALUE!
Blank PEG	T14	G09	0	0	kleiner LLOQ	#VALUE!	1	#VALUE!

	1	2	3	4	5	6	7	8	9	10	11	12
A					0.736	0.721	0.619	0.625	0.122	0.123	0.140	0.139
B					0.064	0.066	0.066	0.067	0.096	0.100	0.099	0.098
C					0.062	0.063	0.074	0.076	0.240	0.247	0.093	0.090
D												
E												
F												
G							0.044	0.043	0.044	0.048	0.046	0.043
H							0.042	0.044	0.041	0.042	0.045	0.044

Plattenbelegung

	1	2	3	4	5	6	7	8	9	10	11	12
A					T14KltN1	T14KltN1	T14KltN2	T14KltN2	T14KltN3	T14KltN3	T14KltN4	T14KltN4
B					T14KltS11	T14KltS11	T14KltS12	T14KltS12	T14KltS13	T14KltS13	T14KltS14	T14KltS14
C					T14KltS21	T14KltS21	T14KltS22	T14KltS22	T14KltS23	T14KltS23	T14KltS24	T14KltS24
D												
E												
F												
G							Blank PEG	Blank PEG	Blank PEG	Blank PEG	Blank PEG	Blank PEG
H							Blank no PEG	Blank no PEG	Blank no PEG	Blank no PEG	Blank no PEG	Blank no PEG

Comment:

Date Operator

Date Control

Blank PEG	T14	G10	0	0	kleiner LLOQ	#VALUE!	1	#VALUE!
Blank PEG	T14	G11	0	0	kleiner LLOQ	#VALUE!	1	#VALUE!
Blank PEG	T14	G12	0	0	kleiner LLOQ	#VALUE!	1	#VALUE!
0	T14	H01	0	0	kleiner LLOQ	#VALUE!	1	#VALUE!
0	T14	H02	0	0	kleiner LLOQ	#VALUE!	1	#VALUE!
0	T14	H03	0	0	kleiner LLOQ	#VALUE!	1	#VALUE!
0	T14	H04	0	0	kleiner LLOQ	#VALUE!	1	#VALUE!
0	T14	H05	0	0	kleiner LLOQ	#VALUE!	1	#VALUE!
0	T14	H06	0	0	kleiner LLOQ	#VALUE!	1	#VALUE!
Blank no PEG	T14	H07	0	0	kleiner LLOQ	#VALUE!	1	#VALUE!
Blank no PEG	T14	H08	0	0	kleiner LLOQ	#VALUE!	1	#VALUE!
Blank no PEG	T14	H09	0	0	kleiner LLOQ	#VALUE!	1	#VALUE!
Blank no PEG	T14	H10	0	0	kleiner LLOQ	#VALUE!	1	#VALUE!
Blank no PEG	T14	H11	0	0	kleiner LLOQ	#VALUE!	1	#VALUE!
Blank no PEG	T14	H12	0	0	kleiner LLOQ	#VALUE!	1	#VALUE!

**Project number**		F-120	**Apparatus**	**Wallac Victor**	**Operator**	**IsBa**
**GLP Study (Number)**		n.a.	**Protocol (Instrument meth**	**LDH test 2016**	**Date of preparation**	**19-04-18**
**hot substance**	**isotope**	n.a.	**File name (results)**	**IsBa_180419/20_LDH_full_**	**Date of measurement**	**19-04-18**
	**name**	n.a.	**Kind of well plate**	**96 well**	**shaking time [min]**	**30**
	**ACB-ID**	n.a.	**sample volume [µL]**	**100**	**stirring rate (Target) [rpm]**	**150**
	**Batch number**	n.a.	**Cocktail volume [µL]**	**175**	**Kind of measurement**	**UV-vis**
**cold substance**	**name**	LDH	**ACB-ID of cocktail**		**Wave length [nm]**	**450**
	**ACB-ID**	L23982R	**Matrix**	**DMEM (from powder)+PE(**	**Remarks**	**Cocktail 100µl RM, 75µl STOP**
	**Batch number**	SLBK6345V	**Blank description**	**DMEM/PEG, H2O**	**Remarks**	**7 standards split low/high**
**n.a.**		n.a.	**Pipettes (No. / volume)**	**50-200µl**	**Remarks**	**KLP4 common for both**
**n.a.**		n.a.	**Pipettes (No. / volume)**	**n.a.**	**Remarks**	**n.a.**

Messdaten (diese Tabelle in Bericht übernehmen)

Sample name *	concentration (theor.) *	measured data	measured data	measured data	mean measured	SD	RSD	Blank *	measured data after * Blank subtraction	concentration (calc.) *	Deviation *	Residuen
	[µg/mL]	[AU]	[AU]	[AU]	[AU]	[AU]	[%]	[AU]		[µg/mL]	[%]	
KLP1	0.148	1.579	1.652	1.627	1.62	0.03	1.87	0.047	1.568	0.148	-0.24	0.00
KLP2	0.114	1.357	1.443	1.372	1.39	0.04	2.70	0.054	1.340	0.110	-3.40	0.00
KLP3	0.074	1.272	1.193	1.228	1.23	0.03	2.63	0.051	1.180	0.084	13.04	0.01
KLP4	0.041	0.959	0.972	0.883	0.94	0.04	4.19	0.051	0.887	0.035	-13.32	-0.01
KLP5								0.052				
KLP6								0.051				
KLP7												
KLP8												

Statistical data

Geradensteigung	Slope	m	6.05
Y-Achsenabschnitt	Y-Intercept	b	0.67
Standardabw. Geradensteigung	SD-Slope	s_m	0.620291227
Standardabw. Achsenabschnittes	SD-Y-Intercept	s_b	0.063694294
Anzahl Messpunkte	number of measuring points	n	4
Quadratsumme	sum of squares	Qxx	0.006578604
Bereichsmitte			0.094336926
Freiheitsgrade	degree of freedom	f	2
Student-t-Faktor für (P = 95 %; f = n-2)	Student-t-factor for (P = 95 %; f = n-2)	t	4.303
Vertrauensbereich Steig. (95 %) Obergrenze		$m + VB_m$	8.722319527
Vertrauensbereich Steig. (95 %) Untergrenze		$m - VB_m$	3.384093228
Vertrauensbereich Achsenabschnitt (95 %) Og.		$b + Vb_b$	0.946914615
Vertrauensbereich Achsenabschnitt (95 %) Ug.		$b - Vb_b$	0.398761518
Korrelationskoeffizient	correlation coefficient	r	0.9897
Bestimmtheitsmaß	determiantion coefficient	r^2	0.9794
Reststandardabweichung		s_0	0.050310948
Summe Restquadrate		sd	2.895583345
Verfahrensstandardabw.		s_{x0}	0.008311454
Rel. Verfahrensstandardabw. %		V_{x0}	8.810393374

mean Blank	**0**	
SD Blank	0.00	
RSD Blank	3.79	x=
x*SD (LLOQ)	0	5
x*SD (LOD)	0	3
**LLOQ (AU)**	**0**	
**LOD (AU)**	**0**	
ULOQ	1.568	
LLOQ (Lin)	0.887	

Evaluation / Comment

LDH linearity valid with 7 standards, split in lin high and lin low, each with 4 standards and KLP4 common standard for both. R² 0,9981, deviations for both between -13.34% and +13.04%

Date	Operator	Date	Control

Across Barriers GmbH
Science Park 1
66123 Saarbrücken

Tel: 0681 95918-800
Fax: 0681 95918-802
e-Mail: sales@acrossbarriers.de
http://www.acrossbarriers.de

Formblatt-Nr.		Version	
Titel			
Gültig ab		Ablage	Projektordner

Figure 1 **Linearity**

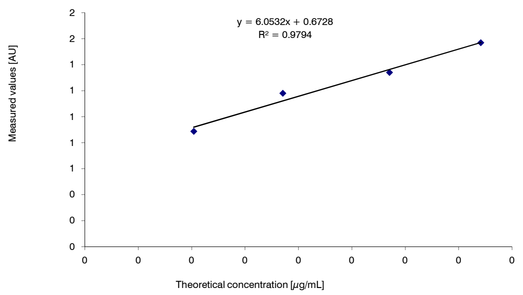

Figure 2 **Method validation Residuen Plot**

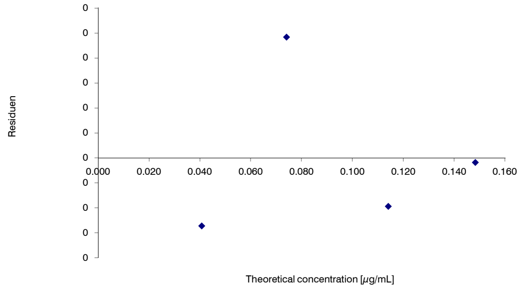

Evaluation / Comment

lin passed

Date	Operator	Date	Control

Sample name	Plate	Position	[AU]	[AU]-Blank	Rating	[µg/mL]	Dilution factor	[µg/mL]
0	T8/T9	A01	0	0	kleiner LLOQ	#VALUE!	1	#VALUE!
0	T8/T9	A02	0	0	kleiner LLOQ	#VALUE!	1	#VALUE!
0	T8/T9	A03	0	0	kleiner LLOQ	#VALUE!	1	#VALUE!
0	T8/T9	A04	0	0	kleiner LLOQ	#VALUE!	1	#VALUE!
T8KltN1	T8/T9	A05	0.000	-0.051	kleiner LLOQ	#VALUE!		#VALUE!
T8KltN1	T8/T9	A06	0.000	-0.051	kleiner LLOQ	#VALUE!		#VALUE!
T8KltN2	T8/T9	A07	0	0	kleiner LLOQ	#VALUE!		#VALUE!
T8KltN2	T8/T9	A08	0	0	kleiner LLOQ	#VALUE!		#VALUE!
T8KltN3	T8/T9	A09	0.000	-0.051	kleiner LLOQ	#VALUE!		#VALUE!
T8KltN3	T8/T9	A10	0.000	-0.051	kleiner LLOQ	#VALUE!		#VALUE!
T8KltN4	T8/T9	A11	0	0	kleiner LLOQ	#VALUE!		#VALUE!
T8KltN4	T8/T9	A12	0	0	kleiner LLOQ	#VALUE!		#VALUE!
0	T8/T9	B01	0	0	kleiner LLOQ	#VALUE!		#VALUE!
0	T8/T9	B02	0	0	kleiner LLOQ	#VALUE!		#VALUE!
0	T8/T9	B03	0	0	kleiner LLOQ	#VALUE!		#VALUE!
0	T8/T9	B04	0	0	kleiner LLOQ	#VALUE!		#VALUE!
T8KltS11	T8/T9	B05	0	0	kleiner LLOQ	#VALUE!		#VALUE!
T8KltS11	T8/T9	B06	0.000	-0.051	kleiner LLOQ	#VALUE!		#VALUE!
T8KltS12	T8/T9	B07	0.000	-0.051	kleiner LLOQ	#VALUE!		#VALUE!
T8KltS12	T8/T9	B08	0.000	-0.051	kleiner LLOQ	#VALUE!		#VALUE!
T8KltS13	T8/T9	B09	0	0	kleiner LLOQ	#VALUE!		#VALUE!
T8KltS13	T8/T9	B10	0	0	kleiner LLOQ	#VALUE!		#VALUE!
T8KltS14	T8/T9	B11	0	0	kleiner LLOQ	#VALUE!		#VALUE!
T8KltS14	T8/T9	B12	0	0	kleiner LLOQ	#VALUE!		#VALUE!
0	T8/T9	C01	0	0	kleiner LLOQ	#VALUE!		#VALUE!
0	T8/T9	C02	0	0	kleiner LLOQ	#VALUE!		#VALUE!
0	T8/T9	C03	0	0	kleiner LLOQ	#VALUE!		#VALUE!
0	T8/T9	C04	0	0	kleiner LLOQ	#VALUE!		#VALUE!
T8KltS21	T8/T9	C05	0.000	-0.051	kleiner LLOQ	#VALUE!		#VALUE!
T8KltS21	T8/T9	C06	0.000	-0.051	kleiner LLOQ	#VALUE!		#VALUE!
T8KltS22	T8/T9	C07	0.000	-0.051	kleiner LLOQ	#VALUE!		#VALUE!
T8KltS22	T8/T9	C08	0.000	-0.051	kleiner LLOQ	#VALUE!		#VALUE!
T8KltS23	T8/T9	C09	0.000	-0.051	kleiner LLOQ	#VALUE!		#VALUE!
T8KltS23	T8/T9	C10	0.000	-0.051	kleiner LLOQ	#VALUE!		#VALUE!
T8KltS24	T8/T9	C11	0	0	kleiner LLOQ	#VALUE!	1	#VALUE!
T8KltS24	T8/T9	C12	0	0	kleiner LLOQ	#VALUE!	1	#VALUE!
0	T8/T9	D01	0	0	kleiner LLOQ	#VALUE!	1	#VALUE!
0	T8/T9	D02	0	0	kleiner LLOQ	#VALUE!	1	#VALUE!
0	T8/T9	D03	0	0	kleiner LLOQ	#VALUE!	1	#VALUE!
0	T8/T9	D04	0	0	kleiner LLOQ	#VALUE!	1	#VALUE!
T9KltN1	T8/T9	D05	0	0	kleiner LLOQ	#VALUE!	1	#VALUE!
T9KltN1	T8/T9	D06	0	0	kleiner LLOQ	#VALUE!	1	#VALUE!
T9KltN2	T8/T9	D07	0	0	kleiner LLOQ	#VALUE!	1	#VALUE!
T9KltN2	T8/T9	D08	0	0	kleiner LLOQ	#VALUE!	1	#VALUE!
T9KltN3	T8/T9	D09	0	0	kleiner LLOQ	#VALUE!	1	#VALUE!
T9KltN3	T8/T9	D10	0	0	kleiner LLOQ	#VALUE!	1	#VALUE!
T9KltN4	T8/T9	D11	0	0	kleiner LLOQ	#VALUE!	1	#VALUE!
T9KltN4	T8/T9	D12	0	0	kleiner LLOQ	#VALUE!	1	#VALUE!

Plate: T8/T9

	1	2	3	4	5	6	7	8	9	10	11	12
A												
B												
C												
D												
E												
F												
G												
H												

Plattenbelegung

	1	2	3	4	5	6	7	8	9	10	11	12
A					T8KltN1	T8KltN1	T8KltN2	T8KltN2	T8KltN3	T8KltN3	T8KltN4	T8KltN4
B					T8KltS11	T8KltS11	T8KltS12	T8KltS12	T8KltS13	T8KltS13	T8KltS14	T8KltS14
C					T8KltS21	T8KltS21	T8KltS22	T8KltS22	T8KltS23	T8KltS23	T8KltS24	T8KltS24
D					T9KltN1	T9KltN1	T9KltN2	T9KltN2	T9KltN3	T9KltN3	T9KltN4	T9KltN4
E					T9KltS11	T9KltS11	T9KltS12	T9KltS12	T9KltS13	T9KltS13	T9KltS14	T9KltS14
F					T9KltS21	T9KltS21	T9KltS22	T9KltS22	T9KltS23	T9KltS23	T9KltS24	T9KltS24
G							Blank PEG	Blank PEG	Blank PEG	Blank PEG	Blank PEG	Blank PEG
H							Blank no PEG	Blank no PEG	Blank no PEG	Blank no PEG	Blank no PEG	Blank no PEG

Comment:

alle Messwerte bei lin low

Samples aufgeteilt in lin high und low:niedriger konzentrierte Samples bei lin low zu finden.

Date Operator

Date Control

0	T8/T9	E01	0	0	kleiner LLOQ	#VALUE!	1	#VALUE!	
0	T8/T9	E02	0	0	kleiner LLOQ	#VALUE!	1	#VALUE!	
0	T8/T9	E03	0	0	kleiner LLOQ	#VALUE!	1	#VALUE!	
0	T8/T9	E04	0	0	kleiner LLOQ	#VALUE!	1	#VALUE!	
T9KltS11	T8/T9	E05	0	0	kleiner LLOQ	#VALUE!	1	#VALUE!	
T9KltS11	T8/T9	E06	0	0	kleiner LLOQ	#VALUE!	1	#VALUE!	
T9KltS12	T8/T9	E07	0	0	kleiner LLOQ	#VALUE!	1	#VALUE!	
T9KltS12	T8/T9	E08	0	0	kleiner LLOQ	#VALUE!	1	#VALUE!	
T9KltS13	T8/T9	E09	0	0	kleiner LLOQ	#VALUE!	1	#VALUE!	
T9KltS13	T8/T9	E10	0	0	kleiner LLOQ	#VALUE!	1	#VALUE!	
T9KltS14	T8/T9	E11	0	0	kleiner LLOQ	#VALUE!	1	#VALUE!	
T9KltS14	T8/T9	E12	0	0	kleiner LLOQ	#VALUE!	1	#VALUE!	
0	T8/T9	F01	0	0	kleiner LLOQ	#VALUE!	1	#VALUE!	
0	T8/T9	F02	0	0	kleiner LLOQ	#VALUE!	1	#VALUE!	
0	T8/T9	F03	0	0	kleiner LLOQ	#VALUE!	1	#VALUE!	
0	T8/T9	F04	0	0	kleiner LLOQ	#VALUE!	1	#VALUE!	
T9KltS21	T8/T9	F05	0	0	kleiner LLOQ	#VALUE!	1	#VALUE!	
T9KltS21	T8/T9	F06	0	0	kleiner LLOQ	#VALUE!	1	#VALUE!	
T9KltS22	T8/T9	F07	0	0	kleiner LLOQ	#VALUE!	1	#VALUE!	
T9KltS22	T8/T9	F08	0	0	kleiner LLOQ	#VALUE!	1	#VALUE!	
T9KltS23	T8/T9	F09	0	0	kleiner LLOQ	#VALUE!	1	#VALUE!	
T9KltS23	T8/T9	F10	0	0	kleiner LLOQ	#VALUE!	1	#VALUE!	
T9KltS24	T8/T9	F11	0	0	kleiner LLOQ	#VALUE!	1	#VALUE!	
T9KltS24	T8/T9	F12	0	0	kleiner LLOQ	#VALUE!	1	#VALUE!	
0	T8/T9	G01	0	0	kleiner LLOQ	#VALUE!	1	#VALUE!	
0	T8/T9	G02	0	0	kleiner LLOQ	#VALUE!	1	#VALUE!	
0	T8/T9	G03	0	0	kleiner LLOQ	#VALUE!	1	#VALUE!	
0	T8/T9	G04	0	0	kleiner LLOQ	#VALUE!	1	#VALUE!	
0	T8/T9	G05	0	0	kleiner LLOQ	#VALUE!	1	#VALUE!	
0	T8/T9	G06	0	0	kleiner LLOQ	#VALUE!	1	#VALUE!	
Blank PEG	T8/T9	G07	0.000	-0.051	kleiner LLOQ	#VALUE!	2.75	#VALUE!	
Blank PEG	T8/T9	G08	0.000	-0.051	kleiner LLOQ	#VALUE!	2.75	#VALUE!	
Blank PEG	T8/T9	G09	0.000	-0.051	kleiner LLOQ	#VALUE!	2.75	#VALUE!	
Blank PEG	T8/T9	G10	0.000	-0.051	kleiner LLOQ	#VALUE!	2.75	#VALUE!	
Blank PEG	T8/T9	G11	0.000	-0.051	kleiner LLOQ	#VALUE!	2.75	#VALUE!	
Blank PEG	T8/T9	G12	0.000	-0.051	kleiner LLOQ	#VALUE!	2.75	#VALUE!	
0	T8/T9	H01	0	0	kleiner LLOQ	#VALUE!	1	#VALUE!	
0	T8/T9	H02	0	0	kleiner LLOQ	#VALUE!	1	#VALUE!	
0	T8/T9	H03	0	0	kleiner LLOQ	#VALUE!	1	#VALUE!	
0	T8/T9	H04	0	0	kleiner LLOQ	#VALUE!	1	#VALUE!	
0	T8/T9	H05	0	0	kleiner LLOQ	#VALUE!	1	#VALUE!	
0	T8/T9	H06	0	0	kleiner LLOQ	#VALUE!	1	#VALUE!	
Blank no PEG	T8/T9	H07	0.000	-0.051	kleiner LLOQ	#VALUE!	2.75	#VALUE!	
Blank no PEG	T8/T9	H08	0.000	-0.051	kleiner LLOQ	#VALUE!	2.75	#VALUE!	
Blank no PEG	T8/T9	H09	0.000	-0.051	kleiner LLOQ	#VALUE!	2.75	#VALUE!	
Blank no PEG	T8/T9	H10	0.000	-0.051	kleiner LLOQ	#VALUE!	2.75	#VALUE!	
Blank no PEG	T8/T9	H11	0.000	-0.051	kleiner LLOQ	#VALUE!	2.75	#VALUE!	
Blank no PEG	T8/T9	H12	0.000	-0.051	kleiner LLOQ	#VALUE!	2.75	#VALUE!	

0	T10/T11	A01	0	0	kleiner LLOQ	#VALUE!	2	#VALUE!
0	T10/T11	A02	0	0	kleiner LLOQ	#VALUE!	3	#VALUE!
0	T10/T11	A03	0	0	kleiner LLOQ	#VALUE!	4	#VALUE!
0	T10/T11	A04	0	0	kleiner LLOQ	#VALUE!	5	#VALUE!
T10KltN1	T10/T11	A05	0	0	kleiner LLOQ	#VALUE!	6	#VALUE!
T10KltN1	T10/T11	A06	0	0	kleiner LLOQ	#VALUE!	7	#VALUE!
T10KltN2	T10/T11	A07	0	0	kleiner LLOQ	#VALUE!	8	#VALUE!
T10KltN2	T10/T11	A08	0	0	kleiner LLOQ	#VALUE!	9	#VALUE!
T10KltN3	T10/T11	A09	0	0	kleiner LLOQ	#VALUE!	10	#VALUE!
T10KltN3	T10/T11	A10	0	0	kleiner LLOQ	#VALUE!	11	#VALUE!
T10KltN4	T10/T11	A11	0	0	kleiner LLOQ	#VALUE!	12	#VALUE!
T10KltN4	T10/T11	A12	0	0	kleiner LLOQ	#VALUE!	13	#VALUE!
0	T10/T11	B01	0	0	kleiner LLOQ	#VALUE!	14	#VALUE!
0	T10/T11	B02	0	0	kleiner LLOQ	#VALUE!	15	#VALUE!
0	T10/T11	B03	0	0	kleiner LLOQ	#VALUE!	16	#VALUE!
0	T10/T11	B04	0	0	kleiner LLOQ	#VALUE!	17	#VALUE!
T10KltS11	T10/T11	B05	0	0	kleiner LLOQ	#VALUE!	18	#VALUE!
T10KltS11	T10/T11	B06	0	0	kleiner LLOQ	#VALUE!	19	#VALUE!
T10KltS12	T10/T11	B07	0	0	kleiner LLOQ	#VALUE!	20	#VALUE!
T10KltS12	T10/T11	B08	0	0	kleiner LLOQ	#VALUE!	21	#VALUE!
T10KltS13	T10/T11	B09	0	0	kleiner LLOQ	#VALUE!	22	#VALUE!
T10KltS13	T10/T11	B10	0	0	kleiner LLOQ	#VALUE!	23	#VALUE!
T10KltS14	T10/T11	B11	0	0	kleiner LLOQ	#VALUE!	24	#VALUE!
T10KltS14	T10/T11	B12	0	0	kleiner LLOQ	#VALUE!	25	#VALUE!
0	T10/T11	C01	0	0	kleiner LLOQ	#VALUE!	26	#VALUE!
0	T10/T11	C02	0	0	kleiner LLOQ	#VALUE!	27	#VALUE!
0	T10/T11	C03	0	0	kleiner LLOQ	#VALUE!	28	#VALUE!
0	T10/T11	C04	0	0	kleiner LLOQ	#VALUE!	29	#VALUE!
T10KltS21	T10/T11	C05	0	0	kleiner LLOQ	#VALUE!	30	#VALUE!
T10KltS21	T10/T11	C06	0	0	kleiner LLOQ	#VALUE!	31	#VALUE!
T10KltS22	T10/T11	C07	0	0	kleiner LLOQ	#VALUE!	32	#VALUE!
T10KltS22	T10/T11	C08	0	0	kleiner LLOQ	#VALUE!	33	#VALUE!
T10KltS23	T10/T11	C09	0	0	kleiner LLOQ	#VALUE!	34	#VALUE!
T10KltS23	T10/T11	C10	0	0	kleiner LLOQ	#VALUE!	35	#VALUE!
T10KltS24	T10/T11	C11	0	0	kleiner LLOQ	#VALUE!	36	#VALUE!
T10KltS24	T10/T11	C12	0	0	kleiner LLOQ	#VALUE!	37	#VALUE!
0	T10/T11	D01	0	0	kleiner LLOQ	#VALUE!	38	#VALUE!
0	T10/T11	D02	0	0	kleiner LLOQ	#VALUE!	39	#VALUE!
0	T10/T11	D03	0	0	kleiner LLOQ	#VALUE!	40	#VALUE!
0	T10/T11	D04	0	0	kleiner LLOQ	#VALUE!	41	#VALUE!
T11KltN1	T10/T11	D05	0	0	kleiner LLOQ	#VALUE!	42	#VALUE!
T11KltN1	T10/T11	D06	0	0	kleiner LLOQ	#VALUE!	43	#VALUE!
T11KltN2	T10/T11	D07	0	0	kleiner LLOQ	#VALUE!	44	#VALUE!
T11KltN2	T10/T11	D08	0	0	kleiner LLOQ	#VALUE!	45	#VALUE!
T11KltN3	T10/T11	D09	0	0	kleiner LLOQ	#VALUE!	46	#VALUE!
T11KltN3	T10/T11	D10	0	0	kleiner LLOQ	#VALUE!	47	#VALUE!
T11KltN4	T10/T11	D11	0	0	kleiner LLOQ	#VALUE!	48	#VALUE!
T11KltN4	T10/T11	D12	0	0	kleiner LLOQ	#VALUE!	49	#VALUE!

Plate: T10/T11

	1	2	3	4	5	6	7	8	9	10	11	12
A												
B												
C												
D												
E												
F												
G												
H												

Plattenbelegung

	1	2	3	4	5	6	7	8	9	10	11	12
A					T10KltN1	T10KltN1	T10KltN2	T10KltN2	T10KltN3	T10KltN3	T10KltN4	T10KltN4
B					T10KltS11	T10KltS11	T10KltS12	T10KltS12	T10KltS13	T10KltS13	T10KltS14	T10KltS14
C					T10KltS21	T10KltS21	T10KltS22	T10KltS22	T10KltS23	T10KltS23	T10KltS24	T10KltS24
D					T11KltN1	T11KltN1	T11KltN2	T11KltN2	T11KltN3	T11KltN3	T11KltN4	T11KltN4
E					T11KltS11	T11KltS11	T11KltS12	T11KltS12	T11KltS13	T11KltS13	T11KltS14	T11KltS14
F					T11KltS21	T11KltS21	T11KltS22	T11KltS22	T11KltS23	T11KltS23	T11KltS24	T11KltS24
G							Blank PEG	Blank PEG	Blank PEG	Blank PEG	Blank PEG	Blank PEG
H							Blank no PEG	Blank no PEG	Blank no PEG	Blank no PEG	Blank no PEG	Blank no PEG

Comment:

alle Messwerte bei lin low

Date Operator

Date Control

0	T10/T11	E01	0	0	kleiner LLOQ	#VALUE!	50	#VALUE!
0	T10/T11	E02	0	0	kleiner LLOQ	#VALUE!	51	#VALUE!
0	T10/T11	E03	0	0	kleiner LLOQ	#VALUE!	52	#VALUE!
0	T10/T11	E04	0	0	kleiner LLOQ	#VALUE!	53	#VALUE!
T11KltS11	T10/T11	E05	0	0	kleiner LLOQ	#VALUE!	54	#VALUE!
T11KltS11	T10/T11	E06	0	0	kleiner LLOQ	#VALUE!	55	#VALUE!
T11KltS12	T10/T11	E07	0	0	kleiner LLOQ	#VALUE!	56	#VALUE!
T11KltS12	T10/T11	E08	0	0	kleiner LLOQ	#VALUE!	57	#VALUE!
T11KltS13	T10/T11	E09	0	0	kleiner LLOQ	#VALUE!	58	#VALUE!
T11KltS13	T10/T11	E10	0	0	kleiner LLOQ	#VALUE!	59	#VALUE!
T11KltS14	T10/T11	E11	0	0	kleiner LLOQ	#VALUE!	60	#VALUE!
T11KltS14	T10/T11	E12	0	0	kleiner LLOQ	#VALUE!	61	#VALUE!
0	T10/T11	F01	0	0	kleiner LLOQ	#VALUE!	62	#VALUE!
0	T10/T11	F02	0	0	kleiner LLOQ	#VALUE!	63	#VALUE!
0	T10/T11	F03	0	0	kleiner LLOQ	#VALUE!	64	#VALUE!
0	T10/T11	F04	0	0	kleiner LLOQ	#VALUE!	65	#VALUE!
T11KltS21	T10/T11	F05	0	0	kleiner LLOQ	#VALUE!	66	#VALUE!
T11KltS21	T10/T11	F06	0	0	kleiner LLOQ	#VALUE!	67	#VALUE!
T11KltS22	T10/T11	F07	0	0	kleiner LLOQ	#VALUE!	68	#VALUE!
T11KltS22	T10/T11	F08	0	0	kleiner LLOQ	#VALUE!	69	#VALUE!
T11KltS23	T10/T11	F09	0	0	kleiner LLOQ	#VALUE!	70	#VALUE!
T11KltS23	T10/T11	F10	0	0	kleiner LLOQ	#VALUE!	71	#VALUE!
T11KltS24	T10/T11	F11	0	0	kleiner LLOQ	#VALUE!	72	#VALUE!
T11KltS24	T10/T11	F12	0	0	kleiner LLOQ	#VALUE!	73	#VALUE!
0	T10/T11	G01	0	0	kleiner LLOQ	#VALUE!	74	#VALUE!
0	T10/T11	G02	0	0	kleiner LLOQ	#VALUE!	75	#VALUE!
0	T10/T11	G03	0	0	kleiner LLOQ	#VALUE!	76	#VALUE!
0	T10/T11	G04	0	0	kleiner LLOQ	#VALUE!	77	#VALUE!
0	T10/T11	G05	0	0	kleiner LLOQ	#VALUE!	78	#VALUE!
0	T10/T11	G06	0	0	kleiner LLOQ	#VALUE!	79	#VALUE!
Blank PEG	T10/T11	G07	0	0	kleiner LLOQ	#VALUE!	80	#VALUE!
Blank PEG	T10/T11	G08	0	0	kleiner LLOQ	#VALUE!	81	#VALUE!
Blank PEG	T10/T11	G09	0	0	kleiner LLOQ	#VALUE!	82	#VALUE!
Blank PEG	T10/T11	G10	0	0	kleiner LLOQ	#VALUE!	83	#VALUE!
Blank PEG	T10/T11	G11	0	0	kleiner LLOQ	#VALUE!	84	#VALUE!
Blank PEG	T10/T11	G12	0	0	kleiner LLOQ	#VALUE!	85	#VALUE!
0	T10/T11	H01	0	0	kleiner LLOQ	#VALUE!	86	#VALUE!
0	T10/T11	H02	0	0	kleiner LLOQ	#VALUE!	87	#VALUE!
0	T10/T11	H03	0	0	kleiner LLOQ	#VALUE!	88	#VALUE!
0	T10/T11	H04	0	0	kleiner LLOQ	#VALUE!	89	#VALUE!
0	T10/T11	H05	0	0	kleiner LLOQ	#VALUE!	90	#VALUE!
0	T10/T11	H06	0	0	kleiner LLOQ	#VALUE!	91	#VALUE!
Blank no PEG	T10/T11	H07	0	0	kleiner LLOQ	#VALUE!	92	#VALUE!
Blank no PEG	T10/T11	H08	0	0	kleiner LLOQ	#VALUE!	93	#VALUE!
Blank no PEG	T10/T11	H09	0	0	kleiner LLOQ	#VALUE!	94	#VALUE!
Blank no PEG	T10/T11	H10	0	0	kleiner LLOQ	#VALUE!	95	#VALUE!
Blank no PEG	T10/T11	H11	0	0	kleiner LLOQ	#VALUE!	96	#VALUE!
Blank no PEG	T10/T11	H12	0	0	kleiner LLOQ	#VALUE!	97	#VALUE!
0	T12/T13	A01	0	0	kleiner LLOQ	#VALUE!	98	#VALUE!
0	T12/T13	A02	0	0	kleiner LLOQ	#VALUE!	99	#VALUE!
0	T12/T13	A03	0	0	kleiner LLOQ	#VALUE!	100	#VALUE!
0	T12/T13	A04	0	0	kleiner LLOQ	#VALUE!	101	#VALUE!
T12KltN1	T12/T13	A05	0	0	kleiner LLOQ	#VALUE!	102	#VALUE!
T12KltN1	T12/T13	A06	0	0	kleiner LLOQ	#VALUE!	103	#VALUE!
T12KltN2	T12/T13	A07	0	0	kleiner LLOQ	#VALUE!	104	#VALUE!
T12KltN2	T12/T13	A08	0	0	kleiner LLOQ	#VALUE!	105	#VALUE!
T12KltN3	T12/T13	A09	0	0	kleiner LLOQ	#VALUE!	106	#VALUE!
T12KltN3	T12/T13	A10	0	0	kleiner LLOQ	#VALUE!	107	#VALUE!
T12KltN4	T12/T13	A11	0	0	kleiner LLOQ	#VALUE!	108	#VALUE!
T12KltN4	T12/T13	A12	0	0	kleiner LLOQ	#VALUE!	109	#VALUE!
0	T12/T13	B01	0	0	kleiner LLOQ	#VALUE!	110	#VALUE!
0	T12/T13	B02	0	0	kleiner LLOQ	#VALUE!	111	#VALUE!
0	T12/T13	B03	0	0	kleiner LLOQ	#VALUE!	112	#VALUE!
0	T12/T13	B04	0	0	kleiner LLOQ	#VALUE!	113	#VALUE!
T12KltS11	T12/T13	B05	0	0	kleiner LLOQ	#VALUE!	114	#VALUE!
T12KltS11	T12/T13	B06	0	0	kleiner LLOQ	#VALUE!	115	#VALUE!
T12KltS12	T12/T13	B07	0	0	kleiner LLOQ	#VALUE!	116	#VALUE!
T12KltS12	T12/T13	B08	0	0	kleiner LLOQ	#VALUE!	117	#VALUE!
T12KltS13	T12/T13	B09	0	0	kleiner LLOQ	#VALUE!	118	#VALUE!
T12KltS13	T12/T13	B10	0	0	kleiner LLOQ	#VALUE!	119	#VALUE!
T12KltS14	T12/T13	B11	0	0	kleiner LLOQ	#VALUE!	120	#VALUE!
T12KltS14	T12/T13	B12	0	0	kleiner LLOQ	#VALUE!	121	#VALUE!
0	T12/T13	C01	0	0	kleiner LLOQ	#VALUE!	122	#VALUE!
0	T12/T13	C02	0	0	kleiner LLOQ	#VALUE!	123	#VALUE!
0	T12/T13	C03	0	0	kleiner LLOQ	#VALUE!	124	#VALUE!
0	T12/T13	C04	0	0	kleiner LLOQ	#VALUE!	125	#VALUE!
T12KltS21	T12/T13	C05	0	0	kleiner LLOQ	#VALUE!	126	#VALUE!
T12KltS21	T12/T13	C06	0	0	kleiner LLOQ	#VALUE!	127	#VALUE!
T12KltS22	T12/T13	C07	0	0	kleiner LLOQ	#VALUE!	128	#VALUE!
T12KltS22	T12/T13	C08	0	0	kleiner LLOQ	#VALUE!	129	#VALUE!
T12KltS23	T12/T13	C09	0	0	kleiner LLOQ	#VALUE!	130	#VALUE!

Plate: T12/T13

	1	2	3	4	5	6	7	8	9	10	11	12
A												
B												
C												
D												
E												
F												
G												
H												

Plattenbelegung

	1	2	3	4	5	6	7	8	9	10	11	12
A					T12KltN1	T12KltN1	T12KltN2	T12KltN2	T12KltN3	T12KltN3	T12KltN4	T12KltN4
B					T12KltS11	T12KltS11	T12KltS12	T12KltS12	T12KltS13	T12KltS13	T12KltS14	T12KltS14
C					T12KltS21	T12KltS21	T12KltS22	T12KltS22	T12KltS23	T12KltS23	T12KltS24	T12KltS24
D					T13KltN1	T13KltN1	T13KltN2	T13KltN2	T13KltN3	T13KltN3	T13KltN4	T13KltN4
E					T13KltS11	T13KltS11	T13KltS12	T13KltS12	T13KltS13	T13KltS13	T13KltS14	T13KltS14
F					T13KltS21	T13KltS21	T13KltS22	T13KltS22	T13KltS23	T13KltS23	T13KltS24	T13KltS24
G							Blank PEG	Blank PEG	Blank PEG	Blank PEG	Blank PEG	Blank PEG
H							Blank no PEG	Blank no PEG	Blank no PEG	Blank no PEG	Blank no PEG	Blank no PEG

Comment:

alle Messwerte bei lin low

T12KltS23	T12/T13	C10	0	0	kleiner LLOQ	#VALUE!	131	#VALUE!
T12KltS24	T12/T13	C11	0	0	kleiner LLOQ	#VALUE!	132	#VALUE!
T12KltS24	T12/T13	C12	0	0	kleiner LLOQ	#VALUE!	133	#VALUE!
0	T12/T13	D01	0	0	kleiner LLOQ	#VALUE!	134	#VALUE!
0	T12/T13	D02	0	0	kleiner LLOQ	#VALUE!	135	#VALUE!
0	T12/T13	D03	0	0	kleiner LLOQ	#VALUE!	136	#VALUE!
0	T12/T13	D04	0	0	kleiner LLOQ	#VALUE!	137	#VALUE!
T13KltN1	T12/T13	D05	0	0	kleiner LLOQ	#VALUE!	138	#VALUE!
T13KltN1	T12/T13	D06	0	0	kleiner LLOQ	#VALUE!	139	#VALUE!
T13KltN2	T12/T13	D07	0	0	kleiner LLOQ	#VALUE!	140	#VALUE!
T13KltN2	T12/T13	D08	0	0	kleiner LLOQ	#VALUE!	141	#VALUE!
T13KltN3	T12/T13	D09	0	0	kleiner LLOQ	#VALUE!	142	#VALUE!
T13KltN3	T12/T13	D10	0	0	kleiner LLOQ	#VALUE!	143	#VALUE!
T13KltN4	T12/T13	D11	0	0	kleiner LLOQ	#VALUE!	144	#VALUE!
T13KltN4	T12/T13	D12	0	0	kleiner LLOQ	#VALUE!	145	#VALUE!
0	T12/T13	E01	0	0	kleiner LLOQ	#VALUE!	146	#VALUE!
0	T12/T13	E02	0	0	kleiner LLOQ	#VALUE!	147	#VALUE!
0	T12/T13	E03	0	0	kleiner LLOQ	#VALUE!	148	#VALUE!
0	T12/T13	E04	0	0	kleiner LLOQ	#VALUE!	149	#VALUE!
T13KltS11	T12/T13	E05	0	0	kleiner LLOQ	#VALUE!	150	#VALUE!
T13KltS11	T12/T13	E06	0	0	kleiner LLOQ	#VALUE!	151	#VALUE!
T13KltS12	T12/T13	E07	0	0	kleiner LLOQ	#VALUE!	152	#VALUE!
T13KltS12	T12/T13	E08	0	0	kleiner LLOQ	#VALUE!	153	#VALUE!
T13KltS13	T12/T13	E09	0	0	kleiner LLOQ	#VALUE!	154	#VALUE!
T13KltS13	T12/T13	E10	0	0	kleiner LLOQ	#VALUE!	155	#VALUE!
T13KltS14	T12/T13	E11	0	0	kleiner LLOQ	#VALUE!	156	#VALUE!
T13KltS14	T12/T13	E12	0	0	kleiner LLOQ	#VALUE!	157	#VALUE!
0	T12/T13	F01	0	0	kleiner LLOQ	#VALUE!	158	#VALUE!
0	T12/T13	F02	0	0	kleiner LLOQ	#VALUE!	159	#VALUE!
0	T12/T13	F03	0	0	kleiner LLOQ	#VALUE!	160	#VALUE!
0	T12/T13	F04	0	0	kleiner LLOQ	#VALUE!	161	#VALUE!
T13KltS21	T12/T13	F05	0	0	kleiner LLOQ	#VALUE!	162	#VALUE!
T13KltS21	T12/T13	F06	0	0	kleiner LLOQ	#VALUE!	163	#VALUE!
T13KltS22	T12/T13	F07	0	0	kleiner LLOQ	#VALUE!	164	#VALUE!
T13KltS22	T12/T13	F08	0	0	kleiner LLOQ	#VALUE!	165	#VALUE!
T13KltS23	T12/T13	F09	0	0	kleiner LLOQ	#VALUE!	166	#VALUE!
T13KltS23	T12/T13	F10	0	0	kleiner LLOQ	#VALUE!	167	#VALUE!
T13KltS24	T12/T13	F11	0	0	kleiner LLOQ	#VALUE!	168	#VALUE!
T13KltS24	T12/T13	F12	0	0	kleiner LLOQ	#VALUE!	169	#VALUE!
0	T12/T13	G01	0	0	kleiner LLOQ	#VALUE!	170	#VALUE!
0	T12/T13	G02	0	0	kleiner LLOQ	#VALUE!	171	#VALUE!
0	T12/T13	G03	0	0	kleiner LLOQ	#VALUE!	172	#VALUE!
0	T12/T13	G04	0	0	kleiner LLOQ	#VALUE!	173	#VALUE!
0	T12/T13	G05	0	0	kleiner LLOQ	#VALUE!	174	#VALUE!
0	T12/T13	G06	0	0	kleiner LLOQ	#VALUE!	175	#VALUE!
Blank PEG	T12/T13	G07	0	0	kleiner LLOQ	#VALUE!	176	#VALUE!
Blank PEG	T12/T13	G08	0	0	kleiner LLOQ	#VALUE!	177	#VALUE!
Blank PEG	T12/T13	G09	0	0	kleiner LLOQ	#VALUE!	178	#VALUE!
Blank PEG	T12/T13	G10	0	0	kleiner LLOQ	#VALUE!	179	#VALUE!
Blank PEG	T12/T13	G11	0	0	kleiner LLOQ	#VALUE!	180	#VALUE!
Blank PEG	T12/T13	G12	0	0	kleiner LLOQ	#VALUE!	181	#VALUE!
0	T12/T13	H01	0	0	kleiner LLOQ	#VALUE!	182	#VALUE!
0	T12/T13	H02	0	0	kleiner LLOQ	#VALUE!	183	#VALUE!
0	T12/T13	H03	0	0	kleiner LLOQ	#VALUE!	184	#VALUE!
0	T12/T13	H04	0	0	kleiner LLOQ	#VALUE!	185	#VALUE!
0	T12/T13	H05	0	0	kleiner LLOQ	#VALUE!	186	#VALUE!
0	T12/T13	H06	0	0	kleiner LLOQ	#VALUE!	187	#VALUE!
Blank no PEG	T12/T13	H07	0	0	kleiner LLOQ	#VALUE!	188	#VALUE!
Blank no PEG	T12/T13	H08	0	0	kleiner LLOQ	#VALUE!	189	#VALUE!
Blank no PEG	T12/T13	H09	0	0	kleiner LLOQ	#VALUE!	190	#VALUE!
Blank no PEG	T12/T13	H10	0	0	kleiner LLOQ	#VALUE!	191	#VALUE!
Blank no PEG	T12/T13	H11	0	0	kleiner LLOQ	#VALUE!	192	#VALUE!
Blank no PEG	T12/T13	H12	0	0	kleiner LLOQ	#VALUE!	193	#VALUE!

Date Operator

Date Control

0	T14	A01	0	0	kleiner LLOQ	#VALUE!	194	#VALUE!
0	T14	A02	0	0	kleiner LLOQ	#VALUE!	195	#VALUE!
0	T14	A03	0	0	kleiner LLOQ	#VALUE!	196	#VALUE!
0	T14	A04	0	0	kleiner LLOQ	#VALUE!	197	#VALUE!
T14KltN1	T14	A05	0	0	kleiner LLOQ	#VALUE!	198	#VALUE!
T14KltN1	T14	A06	0	0	kleiner LLOQ	#VALUE!	199	#VALUE!
T14KltN2	T14	A07	0	0	kleiner LLOQ	#VALUE!	200	#VALUE!
T14KltN2	T14	A08	0	0	kleiner LLOQ	#VALUE!	201	#VALUE!
T14KltN3	T14	A09	0	0	kleiner LLOQ	#VALUE!	202	#VALUE!
T14KltN3	T14	A10	0	0	kleiner LLOQ	#VALUE!	203	#VALUE!
T14KltN4	T14	A11	0	0	kleiner LLOQ	#VALUE!	204	#VALUE!
T14KltN4	T14	A12	0	0	kleiner LLOQ	#VALUE!	205	#VALUE!
0	T14	B01	0	0	kleiner LLOQ	#VALUE!	206	#VALUE!
0	T14	B02	0	0	kleiner LLOQ	#VALUE!	207	#VALUE!
0	T14	B03	0	0	kleiner LLOQ	#VALUE!	208	#VALUE!
0	T14	B04	0	0	kleiner LLOQ	#VALUE!	209	#VALUE!
T14KltS11	T14	B05	0	0	kleiner LLOQ	#VALUE!	210	#VALUE!
T14KltS11	T14	B06	0	0	kleiner LLOQ	#VALUE!	211	#VALUE!
T14KltS12	T14	B07	0	0	kleiner LLOQ	#VALUE!	212	#VALUE!
T14KltS12	T14	B08	0	0	kleiner LLOQ	#VALUE!	213	#VALUE!
T14KltS13	T14	B09	0	0	kleiner LLOQ	#VALUE!	214	#VALUE!
T14KltS13	T14	B10	0	0	kleiner LLOQ	#VALUE!	215	#VALUE!
T14KltS14	T14	B11	0	0	kleiner LLOQ	#VALUE!	216	#VALUE!
T14KltS14	T14	B12	0	0	kleiner LLOQ	#VALUE!	217	#VALUE!
0	T14	C01	0	0	kleiner LLOQ	#VALUE!	218	#VALUE!
0	T14	C02	0	0	kleiner LLOQ	#VALUE!	219	#VALUE!
0	T14	C03	0	0	kleiner LLOQ	#VALUE!	220	#VALUE!
0	T14	C04	0	0	kleiner LLOQ	#VALUE!	221	#VALUE!
T14KltS21	T14	C05	0	0	kleiner LLOQ	#VALUE!	222	#VALUE!
T14KltS21	T14	C06	0	0	kleiner LLOQ	#VALUE!	223	#VALUE!
T14KltS22	T14	C07	0	0	kleiner LLOQ	#VALUE!	224	#VALUE!
T14KltS22	T14	C08	0	0	kleiner LLOQ	#VALUE!	225	#VALUE!
T14KltS23	T14	C09	0	0	kleiner LLOQ	#VALUE!	226	#VALUE!
T14KltS23	T14	C10	0	0	kleiner LLOQ	#VALUE!	227	#VALUE!
T14KltS24	T14	C11	0	0	kleiner LLOQ	#VALUE!	228	#VALUE!
T14KltS24	T14	C12	0	0	kleiner LLOQ	#VALUE!	229	#VALUE!
0	T14	D01	0	0	kleiner LLOQ	#VALUE!	230	#VALUE!
0	T14	D02	0	0	kleiner LLOQ	#VALUE!	231	#VALUE!
0	T14	D03	0	0	kleiner LLOQ	#VALUE!	232	#VALUE!
0	T14	D04	0	0	kleiner LLOQ	#VALUE!	233	#VALUE!
0	T14	D05	0	0	kleiner LLOQ	#VALUE!	234	#VALUE!
0	T14	D06	0	0	kleiner LLOQ	#VALUE!	235	#VALUE!
0	T14	D07	0	0	kleiner LLOQ	#VALUE!	236	#VALUE!
0	T14	D08	0	0	kleiner LLOQ	#VALUE!	237	#VALUE!
0	T14	D09	0	0	kleiner LLOQ	#VALUE!	238	#VALUE!
0	T14	D10	0	0	kleiner LLOQ	#VALUE!	239	#VALUE!
0	T14	D11	0	0	kleiner LLOQ	#VALUE!	240	#VALUE!
0	T14	D12	0	0	kleiner LLOQ	#VALUE!	241	#VALUE!
0	T14	E01	0	0	kleiner LLOQ	#VALUE!	242	#VALUE!
0	T14	E02	0	0	kleiner LLOQ	#VALUE!	243	#VALUE!
0	T14	E03	0	0	kleiner LLOQ	#VALUE!	244	#VALUE!
0	T14	E04	0	0	kleiner LLOQ	#VALUE!	245	#VALUE!
0	T14	E05	0	0	kleiner LLOQ	#VALUE!	246	#VALUE!
0	T14	E06	0	0	kleiner LLOQ	#VALUE!	247	#VALUE!
0	T14	E07	0	0	kleiner LLOQ	#VALUE!	248	#VALUE!
0	T14	E08	0	0	kleiner LLOQ	#VALUE!	249	#VALUE!
0	T14	E09	0	0	kleiner LLOQ	#VALUE!	250	#VALUE!

Plate: T14

	1	2	3	4	5	6	7	8	9	10	11	12
A												
B												
C												
D												
E												
F												
G												
H												

Plattenbelegung

	1	2	3	4	5	6	7	8	9	10	11	12
A					T14KltN1	T14KltN1	T14KltN2	T14KltN2	T14KltN3	T14KltN3	T14KltN4	T14KltN4
B					T14KltS11	T14KltS11	T14KltS12	T14KltS12	T14KltS13	T14KltS13	T14KltS14	T14KltS14
C					T14KltS21	T14KltS21	T14KltS22	T14KltS22	T14KltS23	T14KltS23	T14KltS24	T14KltS24
D												
E												
F												
G							Blank PEG	Blank PEG	Blank PEG	Blank PEG	Blank PEG	Blank PEG
H							Blank no PEG	Blank no PEG	Blank no PEG	Blank no PEG	Blank no PEG	Blank no PEG

Comment:

alle Messwerte bei lin low

Date Operator

Date Control

0	T14	E10	0	0	kleiner LLOQ	#VALUE!	251	#VALUE!
0	T14	E11	0	0	kleiner LLOQ	#VALUE!	252	#VALUE!
0	T14	E12	0	0	kleiner LLOQ	#VALUE!	253	#VALUE!
0	T14	F01	0	0	kleiner LLOQ	#VALUE!	254	#VALUE!
0	T14	F02	0	0	kleiner LLOQ	#VALUE!	255	#VALUE!
0	T14	F03	0	0	kleiner LLOQ	#VALUE!	256	#VALUE!
0	T14	F04	0	0	kleiner LLOQ	#VALUE!	257	#VALUE!
0	T14	F05	0	0	kleiner LLOQ	#VALUE!	258	#VALUE!
0	T14	F06	0	0	kleiner LLOQ	#VALUE!	259	#VALUE!
0	T14	F07	0	0	kleiner LLOQ	#VALUE!	260	#VALUE!
0	T14	F08	0	0	kleiner LLOQ	#VALUE!	261	#VALUE!
0	T14	F09	0	0	kleiner LLOQ	#VALUE!	262	#VALUE!
0	T14	F10	0	0	kleiner LLOQ	#VALUE!	263	#VALUE!
0	T14	F11	0	0	kleiner LLOQ	#VALUE!	264	#VALUE!
0	T14	F12	0	0	kleiner LLOQ	#VALUE!	265	#VALUE!
0	T14	G01	0	0	kleiner LLOQ	#VALUE!	266	#VALUE!
0	T14	G02	0	0	kleiner LLOQ	#VALUE!	267	#VALUE!
0	T14	G03	0	0	kleiner LLOQ	#VALUE!	268	#VALUE!
0	T14	G04	0	0	kleiner LLOQ	#VALUE!	269	#VALUE!
0	T14	G05	0	0	kleiner LLOQ	#VALUE!	270	#VALUE!
0	T14	G06	0	0	kleiner LLOQ	#VALUE!	271	#VALUE!
Blank PEG	T14	G07	0	0	kleiner LLOQ	#VALUE!	272	#VALUE!
Blank PEG	T14	G08	0	0	kleiner LLOQ	#VALUE!	273	#VALUE!
Blank PEG	T14	G09	0	0	kleiner LLOQ	#VALUE!	274	#VALUE!
Blank PEG	T14	G10	0	0	kleiner LLOQ	#VALUE!	275	#VALUE!
Blank PEG	T14	G11	0	0	kleiner LLOQ	#VALUE!	276	#VALUE!
Blank PEG	T14	G12	0	0	kleiner LLOQ	#VALUE!	277	#VALUE!
0	T14	H01	0	0	kleiner LLOQ	#VALUE!	278	#VALUE!
0	T14	H02	0	0	kleiner LLOQ	#VALUE!	279	#VALUE!
0	T14	H03	0	0	kleiner LLOQ	#VALUE!	280	#VALUE!
0	T14	H04	0	0	kleiner LLOQ	#VALUE!	281	#VALUE!
0	T14	H05	0	0	kleiner LLOQ	#VALUE!	282	#VALUE!
0	T14	H06	0	0	kleiner LLOQ	#VALUE!	283	#VALUE!
Blank no PEG	T14	H07	0	0	kleiner LLOQ	#VALUE!	284	#VALUE!
Blank no PEG	T14	H08	0	0	kleiner LLOQ	#VALUE!	285	#VALUE!
Blank no PEG	T14	H09	0	0	kleiner LLOQ	#VALUE!	286	#VALUE!
Blank no PEG	T14	H10	0	0	kleiner LLOQ	#VALUE!	287	#VALUE!
Blank no PEG	T14	H11	0	0	kleiner LLOQ	#VALUE!	288	#VALUE!
Blank no PEG	T14	H12	0	0	kleiner LLOQ	#VALUE!	289	#VALUE!

1.447	1.739		1.170						0.667	0.440	0.043	1
1.447	1.332		1.392	0.137	0.146	0.130	0.138	0.109	0.729	0.478	0.042	
1.391	1.281		1.311	0.204	0.184	0.184	0.198	0.201	0.743	0.474	0.043	
0.961	0.835		1.405	0.336	0.347	0.375	0.387	0.300	0.824	0.483	0.044	
0.445	0.441		1.280	1.085	1.086	1.218	1.004	0.873	0.704	0.457	0.041	
0.355	0.315		1.332	1.768	1.925	1.753	1.739	1.606			0.036	
0.223	0.188	1.666	1.499	1.632		0.048	0.046	0.047	0.048	0.048	0.048	
0.118	0.106	1.556	1.622	1.573		0.047	0.054	0.051	0.051	0.052	0.051	

1.652	1.627		1.188	0.748	0.811	0.764	0.753	0.674	0.751	0.529	0.044	2
1.443	1.372		1.364	0.139	0.147	0.131	0.139	0.110	0.811	0.561	0.043	
1.377	1.281		1.284	0.206	0.189	0.184	0.200	0.202	0.821	0.560	0.044	
0.972	0.883		1.387	0.339	0.349	0.386	0.406	0.312	0.903	0.565	0.044	
0.484	0.474		1.298	1.087	1.113	1.203	1.022	0.903	0.790	0.544	0.042	
0.359	0.326		1.308	1.622	1.622	1.630	1.625	1.550			0.037	
0.228	0.199	1.527	1.373	1.462		0.048	0.046	0.048	0.049	0.049	0.049	
0.125	0.109	1.473	1.410	1.371		0.048	0.054	0.052	0.052	0.052	0.051	

1.579	1.475		1.150	1.210	1.303	1.202	1.219	1.125	0.759	0.549	0.043	3
1.357	1.303		1.283	0.140	0.148	0.131	0.139	0.111	0.820	0.575	0.043	
1.323	1.228		1.227	0.206	0.188	0.184	0.198	0.201	0.824	0.579	0.043	
0.959	0.872		1.321	0.339	0.348	0.387	0.406	0.314	0.909	0.580	0.044	
0.483	0.473		1.270	1.069	1.103	1.181	1.017	0.903	0.798	0.560	0.042	
0.360	0.326		1.270	1.497	1.448	1.496	1.494	1.449			0.037	
0.228	0.199	1.374	1.262	1.314		0.048	0.046	0.048	0.049	0.049	0.049	
0.125	0.110	1.346	1.262	1.253		0.048	0.054	0.052	0.052	0.052	0.051	

1.511	1.369			1.538	1.712	1.528	1.502	1.425			0.043	4
1.296	1.261										0.043	
1.272	1.193										0.043	
0.947	0.859										0.044	
0.481	0.471										0.042	
0.361	0.325										0.037	
0.229	0.199					0.048	0.046	0.048	0.049	0.049	0.049	
0.125	0.110					0.047	0.054	0.052	0.052	0.052	0.051	

1.493	1.317	1.678	1.110	1.621	1.807	1.618	1.584	1.508	0.765	0.597	0.043	5
1.259	1.238	1.850	1.197	0.139	0.150	0.132	0.139	0.111	0.821	0.588	0.043	
1.248	1.175	1.816	1.153	0.204	0.188	0.184	0.197	0.199	0.831	0.599	0.044	
0.943	0.859	1.970	1.231	0.338	0.357	0.386	0.406	0.316	0.916	0.595	0.044	
0.480	0.470	2.220	1.220	1.054	1.088	1.154	1.006	0.902	0.805	0.579	0.042	
0.359	0.325	2.149	1.211	1.395	1.324	1.348	1.345	1.317	0.037	0.037	0.037	
0.228	0.199	1.240	1.151	1.183	0.034	0.049	0.046	0.048	0.049	0.049	0.049	
0.125	0.110	1.223	1.132	1.146	0.038	0.048	0.054	0.052	0.052	0.052	0.051	

1.434	1.258		1.090	1.648	1.876	1.649	1.627	1.535				6
1.217	1.201		1.149									
1.205	1.143		1.109									
0.935	0.845		1.182									
0.477	0.469		1.181									
0.360	0.323		1.167									
0.229	0.200					0.049	0.047	0.048	0.049	0.049	0.049	
0.125	0.110					0.048	0.054	0.052	0.052	0.052	0.051	

1.013	0.810		0.668	0.808	0.940	0.720	0.798	0.687	0.904	0.845	0.043	7
0.709	0.827		0.703	0.142	0.156	0.136	0.137	0.116	0.990	0.846	0.042	
0.682	0.692		0.680	0.197	0.188	0.190	0.191	0.184	1.023	0.861	0.043	
0.507	0.519		0.740	0.316	0.264	0.364	0.377	0.312	1.111	0.870	0.043	
0.394	0.406		0.682	0.580	0.561	0.600	0.529	0.563	0.962	0.834	0.042	
0.333	0.322		0.661	0.995	0.930	0.848	0.832	0.797	0.037	0.037	0.037	
0.229	0.201	0.843	0.679	0.732		0.048	0.046	0.047			0.047	
0.124	0.111	0.773	0.682	0.701		0.047	0.053	0.051	0.051	0.052	0.051	

skin cultivation

				0.742	0.708	0.415	0.444	0.158	0.154	0.055	0.055	T8/T9 hd
				0.063	0.064	0.221	0.273	0.109	0.217	0.074	0.061	
				0.346	0.348	0.086	0.090	0.202	0.149	0.061	0.054	
				0.692	0.579	0.503	0.503	0.249	0.260	0.146	0.144	
				0.037	0.159	0.164	0.211	0.209	0.141	0.109	0.251	
				0.255	0.243	0.076	0.078	0.233	0.242	0.387	0.318	
						0.050	0.043	0.047	0.050	0.049	0.047	
						0.042	0.045	0.041	0.040	0.040	0.047	

0.038	0.038	0.037	0.038	1.321	1.278	0.836	0.885	0.345	0.335	0.073	0.072	T8/T9 hd WH
0.038	0.037	0.037	0.037	0.099	0.101	0.502	0.625	0.243	0.531	0.108	0.094	
0.037	0.035	0.037	0.037	0.778	0.793	0.161	0.171	0.445	0.336	0.078	0.064	
0.037	0.036	0.037	0.037	1.502	1.288	1.112	1.116	0.578	0.591	0.319	0.299	
0.038	0.037	0.036	0.037	0.037	0.368	0.380	0.506	0.502	0.327	0.225	0.621	
0.036	0.037	0.034	0.037	0.587	0.567	0.132	0.135	0.535	0.553	0.904	0.800	
0.037	0.037	0.037	0.037	0.036	0.037	0.053	0.045	0.045	0.056	0.054	0.053	
0.037	0.037	0.035	0.038	0.033	0.037	0.042	0.053	0.044	0.040	0.043	0.052	

				0.598	0.575	0.524	0.523	0.235	0.225	0.117	0.106	T10/T11 hd
				0.115	0.109	0.177	0.191	0.110	0.109	0.098	0.090	
				0.149	0.152	0.190	0.196	0.211	0.205	0.070	0.078	
				0.648	0.647	0.729	0.693	0.275	0.219	0.151	0.153	
				0.101	0.096	0.178	0.181	0.092	0.087	0.089	0.092	
				0.252	0.256	0.176	0.176	0.223	0.221	0.069	0.068	
						0.044	0.043	0.046	0.047	0.044	0.044	
						0.041	0.041	0.042	0.042	0.041	0.042	

				0.670	0.663	0.524	0.538	0.187	0.184	0.111	0.107	T12/T13 hd
				0.065	0.066	0.111	0.113	0.070	0.069	0.061	0.062	
				0.180	0.181	0.102	0.104	0.171	0.172	0.061	0.062	
				0.678	0.577	0.479	0.502	0.148	0.165	0.137	0.129	
				0.067	0.068	0.095	0.107	0.064	0.065	0.090	0.099	
				0.255	0.263	0.081	0.085	0.255	0.249	0.077	0.075	
						0.044	0.042	0.043	0.042	0.045	0.042	
						0.041	0.040	0.041	0.042	0.042	0.042	

				0.736	0.721	0.619	0.625	0.122	0.123	0.140	0.139	T14 hd
				0.064	0.066	0.066	0.067	0.096	0.100	0.099	0.098	
				0.062	0.063	0.074	0.076	0.240	0.247	0.093	0.090	
						0.044	0.043	0.044	0.048	0.046	0.043	
						0.042	0.044	0.041	0.042	0.045	0.044	

**Project number**		F-120	**Apparatus**	**Wallac Victor**	**Operator**	**IsBa**
**GLP Study (Number)**		n.a.	**Protocol (Instrument method)**	**LDH test 2016**	**Date of preparation**	**19-04-18**
**hot substance**	**isotope**	n.a.	**File name (results)**	**IsBa_180419/20_LDH_full_va**	**Date of measurement**	**19-04-18**
	**name**	n.a.	**Kind of well plate**	**96 well**	**shaking time [min]**	**30**
	**ACB-ID**	n.a.	**sample volume [µL]**	**100**	**stirring rate (Target) [rpm]**	**150**
	**Batch number**	n.a.	**Cocktail volume [µL]**	**175**	**Kind of measurement**	**UV-vis**
**cold substance**	**name**	LDH	**ACB-ID of cocktail**		**Wave length [nm]**	**450**
	**ACB-ID**	L23982R	**Matrix**	**DMEM (from powder)+PEG**	**Remarks**	**Cocktail 100µl RM, 75µl STOP**
	**Batch number**	SLBK6345V	**Blank description**	**DMEM/PEG, H2O**	**Remarks**	**7 standards split low/high**
**n.a.**		n.a.	**Pipettes (No. / volume)**	**50-200µl**	**Remarks**	**KLP4 common for both**
**n.a.**		n.a.	**Pipettes (No. / volume)**	**n.a.**	**Remarks**	**n.a.**

Messdaten (diese Tabelle in Bericht übernehmen)

Sample name *	concentration (theor.) *	measured data	measured data	measured data	mean measured	SD	RSD	Blank *	measured data after * Blank subtraction	concentration (calc.) *	Deviation *	Residuen
	[µg/mL]	[AU]	[AU]	[AU]	[AU]	[AU]	[%]	[AU]		[µg/mL]	[%]	
KLP1								0.047				
KLP2								0.054				
KLP3								0.051				
KLP4	0.041	0.959	0.972	0.883	0.938	0.04	4.19	0.051	0.887	0.041	-0.57	0.00
KLP5	0.018	0.483	0.484	0.474	0.480	0.00	0.98	0.052	0.429	0.019	2.42	0.00
KLP6	0.012	0.360	0.359	0.326	0.348	0.02	4.54	0.051	0.297	0.013	5.00	0.00
KLP7	0.007	0.228	0.228	0.199	0.218	0.014	6.35		0.168	0.006	-11.29	0.00
KLP8												

Statistical data

Geradensteigung	Slope	m	21.03
Y-Achsenabschnitt	Y-Intercept	b	0.03
Standardabw. Geradensteigung	SD-Slope	s_m	0.648055501
Standardabw. Achsenabschnittes	SD-Y-Intercept	s_b	0.015177452
Anzahl Messpunkte	number of measuring points	n	4
Quadratsumme	sum of squares	Qxx	0.000664048
Bereichsmitte			0.019557193
Freiheitsgrade	degree of freedom	f	2
Student-t-Faktor für (P = 95 %; f = n-2)	Student-t-factor for (P = 95 %; f = n-2)	t	4.303
Vertrauensbereich Steig. (95 %) Obergrenze		$m + VB_m$	23.81763508
Vertrauensbereich Steig. (95 %) Untergrenze		$m - VB_m$	18.24046944
Vertrauensbereich Achsenabschnitt (95 %) Og.		$b + Vb_b$	0.099335829
Vertrauensbereich Achsenabschnitt (95 %) Ug.		$b - Vb_b$	-0.031281327
Korrelationskoeffizient	correlation coefficient	r	0.9991
Bestimmtheitsmaß	determiantion coefficient	r^2	0.9981
Reststandardabweichung		s_0	0.01669983
Summe Restquadrate		sd	0.657385838
Verfahrensstandardabw.		s_{x0}	0.000794131
Rel. Verfahrensstandardabw. %		V_{x0}	4.060559029

Berichten

mean Blank	**0**		
SD Blank	0.00		
RSD Blank	3.79	x=	
x*SD (LLOQ)	0.01		5
x*SD (LOD)	0.01		3
**LLOQ (AU)**	**0.061**		
**LOD (AU)**	**0.057**		
ULOQ	0.887		
LLOQ (Lin)	0.168		

Evaluation / Comment

LDH linearity valid with 7 standards, split in lin high and lin low, each with 4 standards and KLP4 common standard for both. R² 0,9981, deviations for both between -13.34% and +13.04%

Date | Operator | Date | Control

Figure 1 Linearity

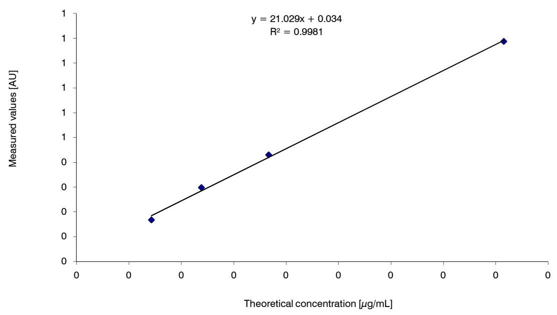

Figure 2 Method validation Residuen Plot

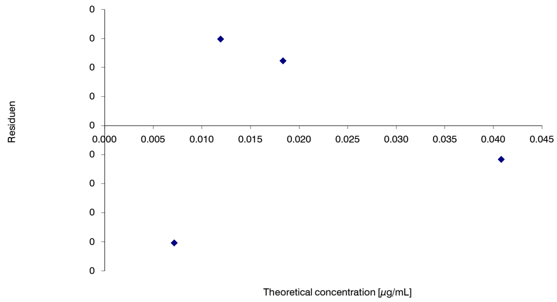

Evaluation / Comment

lin passed

Date	Operator	Date	Control

Sample name	Plate	Position	[AU]	[AU]-Blank	Rating	[µg/mL]	Dilution factor	[µg/mL]
0	0	A01	0	0	kleiner LLOQ	#VALUE!	1	#VALUE!
0	0	A02	0	0	kleiner LLOQ	#VALUE!	1	#VALUE!
0	0	A03	0	0	kleiner LLOQ	#VALUE!	1	#VALUE!
0	0	A04	0	0	kleiner LLOQ	#VALUE!	1	#VALUE!
0	0	A05	0	0	kleiner LLOQ	#VALUE!	1	#VALUE!
0	0	A06	0	0	kleiner LLOQ	#VALUE!	1	#VALUE!
0	0	A07	0	0	kleiner LLOQ	#VALUE!	1	#VALUE!
0	0	A08	0	0	kleiner LLOQ	#VALUE!	1	#VALUE!
0	0	A09	0	0	kleiner LLOQ	#VALUE!	1	#VALUE!
0	0	A10	0	0	kleiner LLOQ	#VALUE!	1	#VALUE!
0	0	A11	0	0	kleiner LLOQ	#VALUE!	1	#VALUE!
0	0	A12	0	0	kleiner LLOQ	#VALUE!	1	#VALUE!
0	0	B01	0	0	kleiner LLOQ	#VALUE!	1	#VALUE!
pos.sui.d1	0	B02	0.897	0.846	0.846	0.039	13.75	0.531
pos.sui.d1	0	B03	0.909	0.858	0.858	0.039	13.75	0.539
pos.sui.d1	0	B04	0.928	0.877	0.877	0.040	13.75	0.551
neg.sui.d1	0	B05	0.088	0.037	kleiner LLOQ	#VALUE!	13.75	#VALUE!
neg.sui.d1	0	B06	0.089	0.038	kleiner LLOQ	#VALUE!	13.75	#VALUE!
neg.sui.d1	0	B07	0.087	0.036	kleiner LLOQ	#VALUE!	13.75	#VALUE!
0	0	B08	0	0	kleiner LLOQ	#VALUE!	1	#VALUE!
0	0	B09	0	0	kleiner LLOQ	#VALUE!	1	#VALUE!
0	0	B10	0	0	kleiner LLOQ	#VALUE!	1	#VALUE!
0	0	B11	0	0	kleiner LLOQ	#VALUE!	1	#VALUE!
0	0	B12	0	0	kleiner LLOQ	#VALUE!	1	#VALUE!
0	0	C01	0	0	kleiner LLOQ	#VALUE!	1	#VALUE!
0	0	C02	0	0	kleiner LLOQ	#VALUE!	1	#VALUE!
0	0	C03	0	0	kleiner LLOQ	#VALUE!	1	#VALUE!
0	0	C04	0	0	kleiner LLOQ	#VALUE!	1	#VALUE!
0	0	C05	0	0	kleiner LLOQ	#VALUE!	1	#VALUE!
0	0	C06	0	0	kleiner LLOQ	#VALUE!	1	#VALUE!
0	0	C07	0	0	kleiner LLOQ	#VALUE!	1	#VALUE!
0	0	C08	0	0	kleiner LLOQ	#VALUE!	1	#VALUE!
0	0	C09	0	0	kleiner LLOQ	#VALUE!	1	#VALUE!
0	0	C10	0	0	kleiner LLOQ	#VALUE!	1	#VALUE!
0	0	C11	0	0	kleiner LLOQ	#VALUE!	1	#VALUE!
0	0	C12	0	0	kleiner LLOQ	#VALUE!	1	#VALUE!
0	0	D01	0	0	kleiner LLOQ	#VALUE!	1	#VALUE!
0	0	D02	0	0	kleiner LLOQ	#VALUE!	1	#VALUE!
0	0	D03	0	0	kleiner LLOQ	#VALUE!	1	#VALUE!
0	0	D04	0	0	kleiner LLOQ	#VALUE!	1	#VALUE!
0	0	D05	0	0	kleiner LLOQ	#VALUE!	1	#VALUE!
0	0	D06	0	0	kleiner LLOQ	#VALUE!	1	#VALUE!
0	0	D07	0	0	kleiner LLOQ	#VALUE!	1	#VALUE!
0	0	D08	0	0	kleiner LLOQ	#VALUE!	1	#VALUE!
0	0	D09	0	0	kleiner LLOQ	#VALUE!	1	#VALUE!
0	0	D10	0	0	kleiner LLOQ	#VALUE!	1	#VALUE!
0	0	D11	0	0	kleiner LLOQ	#VALUE!	1	#VALUE!
0	0	D12	0	0	kleiner LLOQ	#VALUE!	1	#VALUE!

Plate:

	Test	Test	Test	Test								
	1	2	3	4	5	6	7	8	9	10	11	12
A												
B		0.897	0.909	0.928	0.088	0.089	0.087					
C												
D												
E					0.077	0.076	0.073					
F					0.117	0.116	0.115					
G							0.045	0.047	0.049	0.050	0.048	0.050
H							0.048	0.046	0.050	0.048	0.047	0.049

Plattenbelegung

	1	2	3	4	5	6	7	8	9	10	11	12
A												
B		pos.sui.d1	pos.sui.d1	pos.sui.d1	neg.sui.d1	neg.sui.d1	neg.sui.d1					
C												
D												
E		pos.hum.d2	pos.hum.d2	pos.hum.d2	neg.hum.d2	neg.hum.d2	neg.hum.d2					
F		pos.hd.d2	pos.hd.d2	pos.hd.d2	neg.hd.d2	neg.hd.d2	neg.hd.d2					
G							Blank PEG	Blank PEG	Blank PEG	Blank PEG	Blank PEG	Blank PEG
H							Blank no PEG	Blank no PEG	Blank no PEG	Blank no PEG	Blank no PEG	Blank no PEG

Samples aufgeteilt in lin high und low:höher konzentrierte Samples bei lin high zu finden.
d1=dil.5*dil2,75=dil.13,75
d2=dil.30*dil.2,75=dil.82,5

Comment:

Date Operator

Date Control

0	0	E01	0	0	kleiner LLOQ	#VALUE!	1	#VALUE!
pos.hum.d2	0	E02	0	0	kleiner LLOQ	#VALUE!	82.5	#VALUE!
pos.hum.d2	0	E03	0	0	kleiner LLOQ	#VALUE!	82.5	#VALUE!
pos.hum.d2	0	E04	0	0	kleiner LLOQ	#VALUE!	82.5	#VALUE!
neg.hum.d2	0	E05	0.077	0.026	kleiner LLOQ	#VALUE!	82.5	#VALUE!
neg.hum.d2	0	E06	0.076	0.025	kleiner LLOQ	#VALUE!	82.5	#VALUE!
neg.hum.d2	0	E07	0.073	0.022	kleiner LLOQ	#VALUE!	82.5	#VALUE!
0	0	E08	0	0	kleiner LLOQ	#VALUE!	1	#VALUE!
0	0	E09	0	0	kleiner LLOQ	#VALUE!	1	#VALUE!
0	0	E10	0	0	kleiner LLOQ	#VALUE!	1	#VALUE!
0	0	E11	0	0	kleiner LLOQ	#VALUE!	1	#VALUE!
0	0	E12		#VALUE!	#VALUE!	#VALUE!	1	#VALUE!
0	0	F01	0	0	kleiner LLOQ	#VALUE!	1	#VALUE!
pos.hd.d2	0	F02	0	0	kleiner LLOQ	#VALUE!	82.5	#VALUE!
pos.hd.d2	0	F03	0	0	kleiner LLOQ	#VALUE!	82.5	#VALUE!
pos.hd.d2	0	F04	0	0	kleiner LLOQ	#VALUE!	82.5	#VALUE!
neg.hd.d2	0	F05	0.117	0.067	kleiner LLOQ	#VALUE!	82.5	#VALUE!
neg.hd.d2	0	F06	0.116	0.065	kleiner LLOQ	#VALUE!	82.5	#VALUE!
neg.hd.d2	0	F07	0.115	0.064	kleiner LLOQ	#VALUE!	82.5	#VALUE!
0	0	F08	0	0	kleiner LLOQ	#VALUE!	1	#VALUE!
0	0	F09	0	0	kleiner LLOQ	#VALUE!	1	#VALUE!
0	0	F10	0	0	kleiner LLOQ	#VALUE!	1	#VALUE!
0	0	F11	0	0	kleiner LLOQ	#VALUE!	1	#VALUE!
0	0	F12		#VALUE!	#VALUE!	#VALUE!	1	#VALUE!
0	0	G01	0	0	kleiner LLOQ	#VALUE!	1	#VALUE!
0	0	G02	0	0	kleiner LLOQ	#VALUE!	1	#VALUE!
0	0	G03	0	0	kleiner LLOQ	#VALUE!	1	#VALUE!
0	0	G04	0	0	kleiner LLOQ	#VALUE!	1	#VALUE!
0	0	G05	0	0	kleiner LLOQ	#VALUE!	1	#VALUE!
0	0	G06	0	0	kleiner LLOQ	#VALUE!	1	#VALUE!
Blank PEG	0	G07	0	0	kleiner LLOQ	#VALUE!	2.75	#VALUE!
Blank PEG	0	G08	0	0	kleiner LLOQ	#VALUE!	2.75	#VALUE!
Blank PEG	0	G09	0	0	kleiner LLOQ	#VALUE!	2.75	#VALUE!
Blank PEG	0	G10	0	0	kleiner LLOQ	#VALUE!	2.75	#VALUE!
Blank PEG	0	G11	0	0	kleiner LLOQ	#VALUE!	2.75	#VALUE!
Blank PEG	0	G12	0	0	kleiner LLOQ	#VALUE!	2.75	#VALUE!
0	0	H01	0	0	kleiner LLOQ	#VALUE!	1	#VALUE!
0	0	H02	0	0	kleiner LLOQ	#VALUE!	1	#VALUE!
0	0	H03	0	0	kleiner LLOQ	#VALUE!	1	#VALUE!
0	0	H04	0	0	kleiner LLOQ	#VALUE!	1	#VALUE!
0	0	H05	0	0	kleiner LLOQ	#VALUE!	1	#VALUE!
0	0	H06	0	0	kleiner LLOQ	#VALUE!	1	#VALUE!
Blank no PEG	0	H07	0	0	kleiner LLOQ	#VALUE!	2.75	#VALUE!
Blank no PEG	0	H08	0	0	kleiner LLOQ	#VALUE!	2.75	#VALUE!
Blank no PEG	0	H09	0	0	kleiner LLOQ	#VALUE!	2.75	#VALUE!
Blank no PEG	0	H10	0	0	kleiner LLOQ	#VALUE!	2.75	#VALUE!
Blank no PEG	0	H11	0	0	kleiner LLOQ	#VALUE!	2.75	#VALUE!
Blank no PEG	0	H12	0	0	kleiner LLOQ	#VALUE!	2.75	#VALUE!

0	0	A01	0	0	kleiner LLOQ	#VALUE!	1	#VALUE!
0	0	A02	0	0	kleiner LLOQ	#VALUE!	1	#VALUE!
0	0	A03	0	0	kleiner LLOQ	#VALUE!	1	#VALUE!
0	0	A04	0	0	kleiner LLOQ	#VALUE!	1	#VALUE!
0	0	A05	0	0	kleiner LLOQ	#VALUE!	1	#VALUE!
0	0	A06	0	0	kleiner LLOQ	#VALUE!	1	#VALUE!
0	0	A07	0	0	kleiner LLOQ	#VALUE!	1	#VALUE!
0	0	A08	0	0	kleiner LLOQ	#VALUE!	1	#VALUE!
0	0	A09	0	0	kleiner LLOQ	#VALUE!	1	#VALUE!
0	0	A10	0	0	kleiner LLOQ	#VALUE!	1	#VALUE!
0	0	A11	0	0	kleiner LLOQ	#VALUE!	1	#VALUE!
0	0	A12	0	0	kleiner LLOQ	#VALUE!	1	#VALUE!
0	0	B01	0	0	kleiner LLOQ	#VALUE!	1	#VALUE!
0	0	B02	0	0	kleiner LLOQ	#VALUE!	1	#VALUE!
0	0	B03	0	0	kleiner LLOQ	#VALUE!	1	#VALUE!
0	0	B04	0	0	kleiner LLOQ	#VALUE!	1	#VALUE!
0	0	B05	0	0	kleiner LLOQ	#VALUE!	1	#VALUE!
0	0	B06	0	0	kleiner LLOQ	#VALUE!	1	#VALUE!
0	0	B07	0	0	kleiner LLOQ	#VALUE!	1	#VALUE!
0	0	B08	0	0	kleiner LLOQ	#VALUE!	1	#VALUE!
0	0	B09	0	0	kleiner LLOQ	#VALUE!	1	#VALUE!
0	0	B10	0	0	kleiner LLOQ	#VALUE!	1	#VALUE!
0	0	B11	0	0	kleiner LLOQ	#VALUE!	1	#VALUE!
0	0	B12	0	0	kleiner LLOQ	#VALUE!	1	#VALUE!
0	0	C01	0	0	kleiner LLOQ	#VALUE!	1	#VALUE!
0	0	C02	0	0	kleiner LLOQ	#VALUE!	1	#VALUE!
0	0	C03	0	0	kleiner LLOQ	#VALUE!	1	#VALUE!
0	0	C04	0	0	kleiner LLOQ	#VALUE!	1	#VALUE!
0	0	C05	0	0	kleiner LLOQ	#VALUE!	1	#VALUE!
0	0	C06	0	0	kleiner LLOQ	#VALUE!	1	#VALUE!
0	0	C07	0	0	kleiner LLOQ	#VALUE!	1	#VALUE!
0	0	C08	0	0	kleiner LLOQ	#VALUE!	1	#VALUE!
0	0	C09	0	0	kleiner LLOQ	#VALUE!	1	#VALUE!
0	0	C10	0	0	kleiner LLOQ	#VALUE!	1	#VALUE!
0	0	C11	0	0	kleiner LLOQ	#VALUE!	1	#VALUE!
0	0	C12	0	0	kleiner LLOQ	#VALUE!	1	#VALUE!
0	0	D01	0	0	kleiner LLOQ	#VALUE!	1	#VALUE!
0	0	D02	0	0	kleiner LLOQ	#VALUE!	1	#VALUE!
0	0	D03	0	0	kleiner LLOQ	#VALUE!	1	#VALUE!
0	0	D04	0	0	kleiner LLOQ	#VALUE!	1	#VALUE!
0	0	D05	0	0	kleiner LLOQ	#VALUE!	1	#VALUE!
0	0	D06	0	0	kleiner LLOQ	#VALUE!	1	#VALUE!
0	0	D07	0.000	-0.051	kleiner LLOQ	#VALUE!	2.75	#VALUE!
0	0	D08	0.000	-0.051	kleiner LLOQ	#VALUE!	2.75	#VALUE!
0	0	D09	0.000	-0.051	kleiner LLOQ	#VALUE!	2.75	#VALUE!
0	0	D10	0.000	-0.051	kleiner LLOQ	#VALUE!	2.75	#VALUE!
0	0	D11	0.000	-0.051	kleiner LLOQ	#VALUE!	2.75	#VALUE!
0	0	D12	0.000	-0.051	kleiner LLOQ	#VALUE!	2.75	#VALUE!

Plate:

	1	2	3	4	5	6	7	8	9	10	11	12
A												
B												
C												
D												
E												
F												
G												
H												

Plattenbelegung

	1	2	3	4	5	6	7	8	9	10	11	12
A												
B												
C												
D												
E												
F												
G												
H												

Comment:

Date Operator

Date Control

0	0	E01	0	0	kleiner LLOQ	#VALUE!	1	#VALUE!
0	0	E02	0	0	kleiner LLOQ	#VALUE!	1	#VALUE!
0	0	E03	0	0	kleiner LLOQ	#VALUE!	1	#VALUE!
0	0	E04	0	0	kleiner LLOQ	#VALUE!	1	#VALUE!
0	0	E05	0.000	-0.051	kleiner LLOQ	#VALUE!	2.75	#VALUE!
0	0	E06	0.000	-0.051	kleiner LLOQ	#VALUE!	2.75	#VALUE!
0	0	E07	0.000	-0.051	kleiner LLOQ	#VALUE!	2.75	#VALUE!
0	0	E08	0.000	-0.051	kleiner LLOQ	#VALUE!	2.75	#VALUE!
0	0	E09	0.000	-0.051	kleiner LLOQ	#VALUE!	2.75	#VALUE!
0	0	E10	0.000	-0.051	kleiner LLOQ	#VALUE!	2.75	#VALUE!
0	0	E11	0.000	-0.051	kleiner LLOQ	#VALUE!	2.75	#VALUE!
0	0	E12	0.000	-0.051	kleiner LLOQ	#VALUE!	2.75	#VALUE!
0	0	F01	0	0	kleiner LLOQ	#VALUE!	1	#VALUE!
0	0	F02	0	0	kleiner LLOQ	#VALUE!	1	#VALUE!
0	0	F03	0	0	kleiner LLOQ	#VALUE!	1	#VALUE!
0	0	F04	0	0	kleiner LLOQ	#VALUE!	1	#VALUE!
0	0	F05	0.000	-0.051	kleiner LLOQ	#VALUE!	2.75	#VALUE!
0	0	F06	0.000	-0.051	kleiner LLOQ	#VALUE!	2.75	#VALUE!
0	0	F07	0.000	-0.051	kleiner LLOQ	#VALUE!	2.75	#VALUE!
0	0	F08	0.000	-0.051	kleiner LLOQ	#VALUE!	2.75	#VALUE!
0	0	F09	0.000	-0.051	kleiner LLOQ	#VALUE!	2.75	#VALUE!
0	0	F10	0.000	-0.051	kleiner LLOQ	#VALUE!	2.75	#VALUE!
0	0	F11	0.000	-0.051	kleiner LLOQ	#VALUE!	2.75	#VALUE!
0	0	F12	0.000	-0.051	kleiner LLOQ	#VALUE!	2.75	#VALUE!
0	0	G01	0	0	kleiner LLOQ	#VALUE!	1	#VALUE!
0	0	G02	0	0	kleiner LLOQ	#VALUE!	1	#VALUE!
0	0	G03	0	0	kleiner LLOQ	#VALUE!	1	#VALUE!
0	0	G04	0	0	kleiner LLOQ	#VALUE!	1	#VALUE!
0	0	G05	0	0	kleiner LLOQ	#VALUE!	1	#VALUE!
0	0	G06	0	0	kleiner LLOQ	#VALUE!	1	#VALUE!
0	0	G07	0	0	kleiner LLOQ	#VALUE!	2.75	#VALUE!
0	0	G08	0	0	kleiner LLOQ	#VALUE!	2.75	#VALUE!
0	0	G09	0	0	kleiner LLOQ	#VALUE!	2.75	#VALUE!
0	0	G10	0	0	kleiner LLOQ	#VALUE!	2.75	#VALUE!
0	0	G11	0	0	kleiner LLOQ	#VALUE!	2.75	#VALUE!
0	0	G12	0	0	kleiner LLOQ	#VALUE!	2.75	#VALUE!
0	0	H01	0	0	kleiner LLOQ	#VALUE!	1	#VALUE!
0	0	H02	0	0	kleiner LLOQ	#VALUE!	1	#VALUE!
0	0	H03	0	0	kleiner LLOQ	#VALUE!	1	#VALUE!
0	0	H04	0	0	kleiner LLOQ	#VALUE!	1	#VALUE!
0	0	H05	0	0	kleiner LLOQ	#VALUE!	1	#VALUE!
0	0	H06	0	0	kleiner LLOQ	#VALUE!	1	#VALUE!
0	0	H07	0	0	kleiner LLOQ	#VALUE!	2.75	#VALUE!
0	0	H08	0	0	kleiner LLOQ	#VALUE!	2.75	#VALUE!
0	0	H09	0	0	kleiner LLOQ	#VALUE!	2.75	#VALUE!
0	0	H10	0	0	kleiner LLOQ	#VALUE!	2.75	#VALUE!
0	0	H11	0	0	kleiner LLOQ	#VALUE!	2.75	#VALUE!
0	0	H12	0	0	kleiner LLOQ	#VALUE!	2.75	#VALUE!
0	0	A01	0	0	kleiner LLOQ	#VALUE!	1	#VALUE!
0	0	A02	0	0	kleiner LLOQ	#VALUE!	1	#VALUE!
0	0	A03	0	0	kleiner LLOQ	#VALUE!	1	#VALUE!
0	0	A04	0	0	kleiner LLOQ	#VALUE!	1	#VALUE!
0	0	A05	0	0	kleiner LLOQ	#VALUE!	1	#VALUE!
0	0	A06	0	0	kleiner LLOQ	#VALUE!	1	#VALUE!
0	0	A07	0	0	kleiner LLOQ	#VALUE!	1	#VALUE!
0	0	A08	0	0	kleiner LLOQ	#VALUE!	1	#VALUE!
0	0	A09	0	0	kleiner LLOQ	#VALUE!	1	#VALUE!
0	0	A10	0	0	kleiner LLOQ	#VALUE!	1	#VALUE!
0	0	A11	0	0	kleiner LLOQ	#VALUE!	1	#VALUE!
0	0	A12	0	0	kleiner LLOQ	#VALUE!	1	#VALUE!
0	0	B01	0	0	kleiner LLOQ	#VALUE!	1	#VALUE!
0	0	B02	0	0	kleiner LLOQ	#VALUE!	1	#VALUE!
0	0	B03	0	0	kleiner LLOQ	#VALUE!	1	#VALUE!
0	0	B04	0	0	kleiner LLOQ	#VALUE!	1	#VALUE!
0	0	B05	0	0	kleiner LLOQ	#VALUE!	1	#VALUE!
0	0	B06	0	0	kleiner LLOQ	#VALUE!	1	#VALUE!
0	0	B07	0	0	kleiner LLOQ	#VALUE!	1	#VALUE!
0	0	B08	0	0	kleiner LLOQ	#VALUE!	1	#VALUE!
0	0	B09	0	0	kleiner LLOQ	#VALUE!	1	#VALUE!
0	0	B10	0	0	kleiner LLOQ	#VALUE!	1	#VALUE!
0	0	B11	0	0	kleiner LLOQ	#VALUE!	1	#VALUE!
0	0	B12	0	0	kleiner LLOQ	#VALUE!	1	#VALUE!
0	0	C01	0	0	kleiner LLOQ	#VALUE!	1	#VALUE!

Plate:

	1	2	3	4	5	6	7	8	9	10	11	12
A												
B												
C												
D												
E												
F												
G												
H												

Plattenbelegung

	1	2	3	4	5	6	7	8	9	10	11	12
A												
B												
C												
D												
E												

0	0	C02	0	0	kleiner LLOQ	#VALUE!	1	#VALUE!
0	0	C03	0	0	kleiner LLOQ	#VALUE!	1	#VALUE!
0	0	C04	0	0	kleiner LLOQ	#VALUE!	1	#VALUE!
0	0	C05	0	0	kleiner LLOQ	#VALUE!	1	#VALUE!
0	0	C06	0	0	kleiner LLOQ	#VALUE!	1	#VALUE!
0	0	C07	0	0	kleiner LLOQ	#VALUE!	1	#VALUE!
0	0	C08	0	0	kleiner LLOQ	#VALUE!	1	#VALUE!
0	0	C09	0	0	kleiner LLOQ	#VALUE!	1	#VALUE!
0	0	C10	0	0	kleiner LLOQ	#VALUE!	1	#VALUE!
0	0	C11	0	0	kleiner LLOQ	#VALUE!	1	#VALUE!
0	0	C12	0	0	kleiner LLOQ	#VALUE!	1	#VALUE!
0	0	D01	0	0	kleiner LLOQ	#VALUE!	1	#VALUE!
0	0	D02	0	0	kleiner LLOQ	#VALUE!	1	#VALUE!
0	0	D03	0	0	kleiner LLOQ	#VALUE!	1	#VALUE!
0	0	D04	0	0	kleiner LLOQ	#VALUE!	1	#VALUE!
0	0	D05	0	0	kleiner LLOQ	#VALUE!	1	#VALUE!
0	0	D06	0	0	kleiner LLOQ	#VALUE!	1	#VALUE!
0	0	D07	0.000	-0.051	kleiner LLOQ	#VALUE!	2.75	#VALUE!
0	0	D08	0.000	-0.051	kleiner LLOQ	#VALUE!	2.75	#VALUE!
0	0	D09	0.000	-0.051	kleiner LLOQ	#VALUE!	2.75	#VALUE!
0	0	D10	0.000	-0.051	kleiner LLOQ	#VALUE!	2.75	#VALUE!
0	0	D11	0.000	-0.051	kleiner LLOQ	#VALUE!	2.75	#VALUE!
0	0	D12	0.000	-0.051	kleiner LLOQ	#VALUE!	2.75	#VALUE!
0	0	E01	0	0	kleiner LLOQ	#VALUE!	1	#VALUE!
0	0	E02	0	0	kleiner LLOQ	#VALUE!	1	#VALUE!
0	0	E03	0	0	kleiner LLOQ	#VALUE!	1	#VALUE!
0	0	E04	0	0	kleiner LLOQ	#VALUE!	1	#VALUE!
0	0	E05	0.000	-0.051	kleiner LLOQ	#VALUE!	2.75	#VALUE!
0	0	E06	0.000	-0.051	kleiner LLOQ	#VALUE!	2.75	#VALUE!
0	0	E07	0.000	-0.051	kleiner LLOQ	#VALUE!	2.75	#VALUE!
0	0	E08	0.000	-0.051	kleiner LLOQ	#VALUE!	2.75	#VALUE!
0	0	E09	0.000	-0.051	kleiner LLOQ	#VALUE!	2.75	#VALUE!
0	0	E10	0.000	-0.051	kleiner LLOQ	#VALUE!	2.75	#VALUE!
0	0	E11	0.000	-0.051	kleiner LLOQ	#VALUE!	2.75	#VALUE!
0	0	E12	0.000	-0.051	kleiner LLOQ	#VALUE!	2.75	#VALUE!
0	0	F01	0	0	kleiner LLOQ	#VALUE!	1	#VALUE!
0	0	F02	0	0	kleiner LLOQ	#VALUE!	1	#VALUE!
0	0	F03	0	0	kleiner LLOQ	#VALUE!	1	#VALUE!
0	0	F04	0	0	kleiner LLOQ	#VALUE!	1	#VALUE!
0	0	F05	0.000	-0.051	kleiner LLOQ	#VALUE!	2.75	#VALUE!
0	0	F06	0.000	-0.051	kleiner LLOQ	#VALUE!	2.75	#VALUE!
0	0	F07	0.000	-0.051	kleiner LLOQ	#VALUE!	2.75	#VALUE!
0	0	F08	0.000	-0.051	kleiner LLOQ	#VALUE!	2.75	#VALUE!
0	0	F09	0.000	-0.051	kleiner LLOQ	#VALUE!	2.75	#VALUE!
0	0	F10	0.000	-0.051	kleiner LLOQ	#VALUE!	2.75	#VALUE!
0	0	F11	0.000	-0.051	kleiner LLOQ	#VALUE!	2.75	#VALUE!
0	0	F12	0.000	-0.051	kleiner LLOQ	#VALUE!	2.75	#VALUE!
0	0	G01	0	0	kleiner LLOQ	#VALUE!	1	#VALUE!
0	0	G02	0	0	kleiner LLOQ	#VALUE!	1	#VALUE!
0	0	G03	0	0	kleiner LLOQ	#VALUE!	1	#VALUE!
0	0	G04	0	0	kleiner LLOQ	#VALUE!	1	#VALUE!
0	0	G05	0	0	kleiner LLOQ	#VALUE!	1	#VALUE!
0	0	G06	0	0	kleiner LLOQ	#VALUE!	1	#VALUE!
0	0	G07	0	0	kleiner LLOQ	#VALUE!	2.75	#VALUE!
0	0	G08	0	0	kleiner LLOQ	#VALUE!	2.75	#VALUE!
0	0	G09	0	0	kleiner LLOQ	#VALUE!	2.75	#VALUE!
0	0	G10	0	0	kleiner LLOQ	#VALUE!	2.75	#VALUE!
0	0	G11	0	0	kleiner LLOQ	#VALUE!	2.75	#VALUE!
0	0	G12	0	0	kleiner LLOQ	#VALUE!	2.75	#VALUE!
0	0	H01	0	0	kleiner LLOQ	#VALUE!	1	#VALUE!
0	0	H02	0	0	kleiner LLOQ	#VALUE!	1	#VALUE!
0	0	H03	0	0	kleiner LLOQ	#VALUE!	1	#VALUE!
0	0	H04	0	0	kleiner LLOQ	#VALUE!	1	#VALUE!
0	0	H05	0	0	kleiner LLOQ	#VALUE!	1	#VALUE!
0	0	H06	0	0	kleiner LLOQ	#VALUE!	1	#VALUE!
0	0	H07	0	0	kleiner LLOQ	#VALUE!	2.75	#VALUE!
0	0	H08	0	0	kleiner LLOQ	#VALUE!	2.75	#VALUE!
0	0	H09	0	0	kleiner LLOQ	#VALUE!	2.75	#VALUE!
0	0	H10	0	0	kleiner LLOQ	#VALUE!	2.75	#VALUE!
0	0	H11	0	0	kleiner LLOQ	#VALUE!	2.75	#VALUE!
0	0	H12	0	0	kleiner LLOQ	#VALUE!	2.75	#VALUE!

F												
G												
H												

Comment:

Date Operator

Date Control

**Project number**		F-120	**Apparatus**	**Wallac Victor**	**Operator**	**IsBa**
**GLP Study (Number)**		n.a.	**Protocol (Instrument meth**	**LDH test 2016**	**Date of preparation**	**19-04-18**
	**isotope**	n.a.	**File name (results)**	**IsBa_180419/20_LDH_full_**	**Date of measurement**	**19-04-18**
**hot**	**name**	n.a.	**Kind of well plate**	**96 well**	**shaking time [min]**	**30**
**substance**	**ACB-ID**	n.a.	**sample volume [µL]**	**100**	**stirring rate (Target) [rpm]**	**150**
	**Batch number**	n.a.	**Cocktail volume [µL]**	**175**	**Kind of measurement**	**UV-vis**
**cold**	**name**	LDH	**ACB-ID of cocktail**		**Wave length [nm]**	**450**
**substance**	**ACB-ID**	L23982R	**Matrix**	**DMEM (from powder)+PE(**	**Remarks**	**Cocktail 100µl RM, 75µl STOP**
	**Batch number**	SLBK6345V	**Blank description**	**DMEM/PEG, H2O**	**Remarks**	**7 standards split low/high**
**n.a.**		n.a.	**Pipettes (No. / volume)**	**50-200µl**	**Remarks**	**KLP4 common for both**
**n.a.**		n.a.	**Pipettes (No. / volume)**	**n.a.**	**Remarks**	**n.a.**

Messdaten (diese Tabelle in Bericht übernehmen)

Sample name *	concentration (theor.) *	measured data	measured data	measured data	mean measured	SD	RSD	Blank *	measured data after * Blank subtraction	concentration (calc.) *	Deviation *	Residuen
	[µg/mL]	[AU]	[AU]	[AU]	[AU]	[AU]	[%]	[AU]		[µg/mL]	[%]	
KLP1	0.148	1.579	1.652	1.627	1.62	0.03	1.87	0.047	1.568	0.148	-0.24	0.00
KLP2	0.114	1.357	1.443	1.372	1.39	0.04	2.70	0.054	1.340	0.110	-3.40	0.00
KLP3	0.074	1.272	1.193	1.228	1.23	0.03	2.63	0.051	1.180	0.084	13.04	0.01
KLP4	0.041	0.959	0.972	0.883	0.94	0.04	4.19	0.051	0.887	0.035	-13.32	-0.01
KLP5								0.052				
KLP6								0.051				
KLP7												
KLP8												

Statistical data

Geradensteigung	Slope	m	6.05
Y-Achsenabschnitt	Y-Intercept	b	0.67
Standardabw. Geradensteigung	SD-Slope	s_m	0.620291227
Standardabw. Achsenabschnittes	SD-Y-Intercept	s_b	0.063694294
Anzahl Messpunkte	number of measuring points	n	4
Quadratsumme	sum of squares	Qxx	0.006578604
Bereichsmitte			0.094336926
Freiheitsgrade	degree of freedom	f	2
Student-t-Faktor für (P = 95 %; f = n-2)	Student-t-factor for (P = 95 %; f = n-2)	t	4.303
Vertrauensbereich Steig. (95 %) Obergrenze		$m + VB_m$	8.722319527
Vertrauensbereich Steig. (95 %) Untergrenze		$m - VB_m$	3.384093228
Vertrauensbereich Achsenabschnitt (95 %) Og.		$b + Vb_b$	0.946914615
Vertrauensbereich Achsenabschnitt (95 %) Ug.		$b - Vb_b$	0.398761518
Korrelationskoeffizient	correlation coefficient	r	0.9897
Bestimmtheitsmaß	determiantion coefficient	r^2	0.9794
Reststandardabweichung		s_0	0.050310948
Summe Restquadrate		sd	2.895583345
Verfahrensstandardabw.		s_{x0}	0.008311454
Rel. Verfahrensstandardabw. %		V_{x0}	8.810393374

mean Blank	**0**	
SD Blank	0.00	
RSD Blank	3.79	x=
x*SD (LLOQ)	0	5
x*SD (LOD)	0	3
**LLOQ (AU)**	**0**	
**LOD (AU)**	**0**	
ULOQ	1.568	
LLOQ (Lin)	0.887	

Evaluation / Comment

LDH linearity valid with 7 standards, split in lin high and lin low, each with 4 standards and KLP4 common standard for both. R^2 0,9981, deviations for both between -13.34% and +13.04%

Date | Operator | Date | Control

Figure 1 **Linearity**

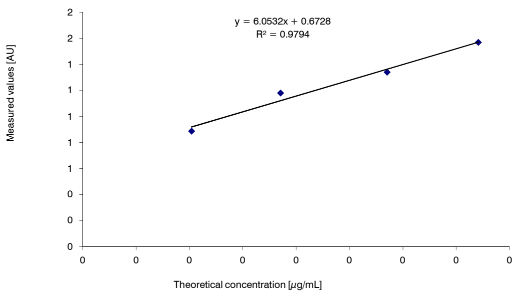

Figure 2 **Method validation Residuen Plot**

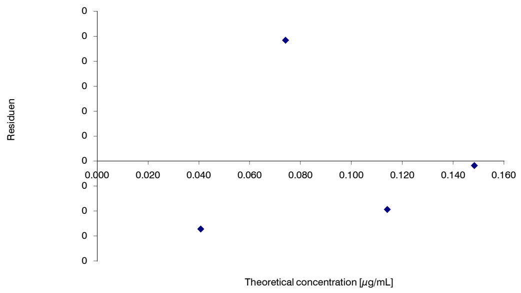

Evaluation / Comment

lin passed

Date	Operator	Date	Control

Sample name	Plate	Position	[AU]	[AU]-Blank	Rating	[µg/mL]	Dilution factor	[µg/mL]
0	Kontrollen	A01	0	0	kleiner LLOQ	#VALUE!	1	#VALUE!
0	Kontrollen	A02	0	0	kleiner LLOQ	#VALUE!	1	#VALUE!
0	Kontrollen	A03	0	0	kleiner LLOQ	#VALUE!	1	#VALUE!
0	Kontrollen	A04	0	0	kleiner LLOQ	#VALUE!	1	#VALUE!
0	Kontrollen	A05	0	0	kleiner LLOQ	#VALUE!	1	#VALUE!
0	Kontrollen	A06	0	0	kleiner LLOQ	#VALUE!	1	#VALUE!
0	Kontrollen	A07	0	0	kleiner LLOQ	#VALUE!	1	#VALUE!
0	Kontrollen	A08	0	0	kleiner LLOQ	#VALUE!	1	#VALUE!
0	Kontrollen	A09	0	0	kleiner LLOQ	#VALUE!	1	#VALUE!
0	Kontrollen	A10	0	0	kleiner LLOQ	#VALUE!	1	#VALUE!
0	Kontrollen	A11	0	0	kleiner LLOQ	#VALUE!	1	#VALUE!
0	Kontrollen	A12	0	0	kleiner LLOQ	#VALUE!	1	#VALUE!
0	Kontrollen	B01	0	0	kleiner LLOQ	#VALUE!	1	#VALUE!
pos.sui.d1	Kontrollen	B02	0	0	kleiner LLOQ	#VALUE!	1	#VALUE!
pos.sui.d1	Kontrollen	B03	0	0	kleiner LLOQ	#VALUE!	1	#VALUE!
pos.sui.d1	Kontrollen	B04	0	0	kleiner LLOQ	#VALUE!	1	#VALUE!
neg.sui.d1	Kontrollen	B05	0.000	-0.051	kleiner LLOQ	#VALUE!	13.75	#VALUE!
neg.sui.d1	Kontrollen	B06	0.000	-0.051	kleiner LLOQ	#VALUE!	13.75	#VALUE!
neg.sui.d1	Kontrollen	B07	0.000	-0.051	kleiner LLOQ	#VALUE!	13.75	#VALUE!
0	Kontrollen	B08	0	0	kleiner LLOQ	#VALUE!	1	#VALUE!
0	Kontrollen	B09	0	0	kleiner LLOQ	#VALUE!	1	#VALUE!
0	Kontrollen	B10	0	0	kleiner LLOQ	#VALUE!	1	#VALUE!
0	Kontrollen	B11	0	0	kleiner LLOQ	#VALUE!	1	#VALUE!
0	Kontrollen	B12	0	0	kleiner LLOQ	#VALUE!	1	#VALUE!
0	Kontrollen	C01	0	0	kleiner LLOQ	#VALUE!	1	#VALUE!
0	Kontrollen	C02	0	0	kleiner LLOQ	#VALUE!	1	#VALUE!
0	Kontrollen	C03	0	0	kleiner LLOQ	#VALUE!	1	#VALUE!
0	Kontrollen	C04	0	0	kleiner LLOQ	#VALUE!	1	#VALUE!
0	Kontrollen	C05	0	0	kleiner LLOQ	#VALUE!	1	#VALUE!
0	Kontrollen	C06	0	0	kleiner LLOQ	#VALUE!	1	#VALUE!
0	Kontrollen	C07	0	0	kleiner LLOQ	#VALUE!	1	#VALUE!
0	Kontrollen	C08	0	0	kleiner LLOQ	#VALUE!	1	#VALUE!
0	Kontrollen	C09	0	0	kleiner LLOQ	#VALUE!	1	#VALUE!
0	Kontrollen	C10	0	0	kleiner LLOQ	#VALUE!	1	#VALUE!
0	Kontrollen	C11	0	0	kleiner LLOQ	#VALUE!	1	#VALUE!
0	Kontrollen	C12	0	0	kleiner LLOQ	#VALUE!	1	#VALUE!
0	Kontrollen	D01	0	0	kleiner LLOQ	#VALUE!	1	#VALUE!
0	Kontrollen	D02	0	0	kleiner LLOQ	#VALUE!	1	#VALUE!
0	Kontrollen	D03	0	0	kleiner LLOQ	#VALUE!	1	#VALUE!
0	Kontrollen	D04	0	0	kleiner LLOQ	#VALUE!	1	#VALUE!
0	Kontrollen	D05	0	0	kleiner LLOQ	#VALUE!	1	#VALUE!
0	Kontrollen	D06	0	0	kleiner LLOQ	#VALUE!	1	#VALUE!
0	Kontrollen	D07	0	0	kleiner LLOQ	#VALUE!	1	#VALUE!
0	Kontrollen	D08	0	0	kleiner LLOQ	#VALUE!	1	#VALUE!
0	Kontrollen	D09	0	0	kleiner LLOQ	#VALUE!	1	#VALUE!
0	Kontrollen	D10	0	0	kleiner LLOQ	#VALUE!	1	#VALUE!
0	Kontrollen	D11	0	0	kleiner LLOQ	#VALUE!	1	#VALUE!
0	Kontrollen	D12	0	0	kleiner LLOQ	#VALUE!	1	#VALUE!

Plate: Kontrollen

	1	2	3	4	5	6	7	8	9	10	11	12
A												
B												
C												
D												
E		1.040	1.053	1.067								
F		1.334	1.265	1.290								
G							0.045	0.047	0.049	0.050	0.048	0.050
H							0.048	0.046	0.050	0.048	0.047	0.049

Plattenbelegung

	1	2	3	4	5	6	7	8	9	10	11	12
A												
B		pos.sui.d1	pos.sui.d1	pos.sui.d1	neg.sui.d1	neg.sui.d1	neg.sui.d1					
C												
D												
E		pos.hum.d2	pos.hum.d2	pos.hum.d2	neg.hum.d2	neg.hum.d2	neg.hum.d2					
F		pos.hd.d2	pos.hd.d2	pos.hd.d2	neg.hd.d2	neg.hd.d2	neg.hd.d2					
G							Blank PEG	Blank PEG	Blank PEG	Blank PEG	Blank PEG	Blank PEG
H							Blank no PEG	Blank no PEG	Blank no PEG	Blank no PEG	Blank no PEG	Blank no PEG

Comment:

Samples aufgeteilt in lin high und low:niedriger konzentrierte Samples bei lin low zu finden.

Date Operator

Date Control

0	Kontrollen	E01	0	0	kleiner LLOQ	#VALUE!	1	#VALUE!
pos.hum.d2	Kontrollen	E02	1.040	0.989	0.989	0.052	82.5	4.306
pos.hum.d2	Kontrollen	E03	1.053	1.002	1.002	0.054	82.5	4.489
pos.hum.d2	Kontrollen	E04	1.067	1.016	1.016	0.057	82.5	4.682
neg.hum.d2	Kontrollen	E05	0.000	-0.051	kleiner LLOQ	#VALUE!	82.5	#VALUE!
neg.hum.d2	Kontrollen	E06	0.000	-0.051	kleiner LLOQ	#VALUE!	82.5	#VALUE!
neg.hum.d2	Kontrollen	E07	0.000	-0.051	kleiner LLOQ	#VALUE!	82.5	#VALUE!
0	Kontrollen	E08	0	0	kleiner LLOQ	#VALUE!	1	#VALUE!
0	Kontrollen	E09	0	0	kleiner LLOQ	#VALUE!	1	#VALUE!
0	Kontrollen	E10	0	0	kleiner LLOQ	#VALUE!	1	#VALUE!
0	Kontrollen	E11	0	0	kleiner LLOQ	#VALUE!	1	#VALUE!
0	Kontrollen	E12		#VALUE!	#VALUE!	#VALUE!	1	#VALUE!
0	Kontrollen	F01	0	0	kleiner LLOQ	#VALUE!	1	#VALUE!
pos.hd.d2	Kontrollen	F02	1.334	1.283	1.283	0.101	82.5	8.317
pos.hd.d2	Kontrollen	F03	1.265	1.214	1.214	0.089	82.5	7.376
pos.hd.d2	Kontrollen	F04	1.290	1.239	1.239	0.094	82.5	7.722
neg.hd.d2	Kontrollen	F05	0.000	-0.051	kleiner LLOQ	#VALUE!	82.5	#VALUE!
neg.hd.d2	Kontrollen	F06	0.000	-0.051	kleiner LLOQ	#VALUE!	82.5	#VALUE!
neg.hd.d2	Kontrollen	F07	0.000	-0.051	kleiner LLOQ	#VALUE!	82.5	#VALUE!
0	Kontrollen	F08	0	0	kleiner LLOQ	#VALUE!	1	#VALUE!
0	Kontrollen	F09	0	0	kleiner LLOQ	#VALUE!	1	#VALUE!
0	Kontrollen	F10	0	0	kleiner LLOQ	#VALUE!	1	#VALUE!
0	Kontrollen	F11	0	0	kleiner LLOQ	#VALUE!	1	#VALUE!
0	Kontrollen	F12		#VALUE!	#VALUE!	#VALUE!	1	#VALUE!
0	Kontrollen	G01	0	0	kleiner LLOQ	#VALUE!	1	#VALUE!
0	Kontrollen	G02	0	0	kleiner LLOQ	#VALUE!	1	#VALUE!
0	Kontrollen	G03	0	0	kleiner LLOQ	#VALUE!	1	#VALUE!
0	Kontrollen	G04	0	0	kleiner LLOQ	#VALUE!	1	#VALUE!
0	Kontrollen	G05	0	0	kleiner LLOQ	#VALUE!	1	#VALUE!
0	Kontrollen	G06	0	0	kleiner LLOQ	#VALUE!	1	#VALUE!
Blank PEG	Kontrollen	G07	0	0	kleiner LLOQ	#VALUE!	2.75	#VALUE!
Blank PEG	Kontrollen	G08	0	0	kleiner LLOQ	#VALUE!	2.75	#VALUE!
Blank PEG	Kontrollen	G09	0	0	kleiner LLOQ	#VALUE!	2.75	#VALUE!
Blank PEG	Kontrollen	G10	0	0	kleiner LLOQ	#VALUE!	2.75	#VALUE!
Blank PEG	Kontrollen	G11	0	0	kleiner LLOQ	#VALUE!	2.75	#VALUE!
Blank PEG	Kontrollen	G12	0	0	kleiner LLOQ	#VALUE!	2.75	#VALUE!
0	Kontrollen	H01	0	0	kleiner LLOQ	#VALUE!	1	#VALUE!
0	Kontrollen	H02	0	0	kleiner LLOQ	#VALUE!	1	#VALUE!
0	Kontrollen	H03	0	0	kleiner LLOQ	#VALUE!	1	#VALUE!
0	Kontrollen	H04	0	0	kleiner LLOQ	#VALUE!	1	#VALUE!
0	Kontrollen	H05	0	0	kleiner LLOQ	#VALUE!	1	#VALUE!
0	Kontrollen	H06	0	0	kleiner LLOQ	#VALUE!	1	#VALUE!
Blank no PEG	Kontrollen	H07	0	0	kleiner LLOQ	#VALUE!	2.75	#VALUE!
Blank no PEG	Kontrollen	H08	0	0	kleiner LLOQ	#VALUE!	2.75	#VALUE!
Blank no PEG	Kontrollen	H09	0	0	kleiner LLOQ	#VALUE!	2.75	#VALUE!
Blank no PEG	Kontrollen	H10	0	0	kleiner LLOQ	#VALUE!	2.75	#VALUE!
Blank no PEG	Kontrollen	H11	0	0	kleiner LLOQ	#VALUE!	2.75	#VALUE!
Blank no PEG	Kontrollen	H12	0	0	kleiner LLOQ	#VALUE!	2.75	#VALUE!

0	0	A01	0	0	kleiner LLOQ	#VALUE!	1	#VALUE!
0	0	A02	0	0	kleiner LLOQ	#VALUE!	1	#VALUE!
0	0	A03	0	0	kleiner LLOQ	#VALUE!	1	#VALUE!
0	0	A04	0	0	kleiner LLOQ	#VALUE!	1	#VALUE!
0	0	A05	0	0	kleiner LLOQ	#VALUE!	1	#VALUE!
0	0	A06	0	0	kleiner LLOQ	#VALUE!	1	#VALUE!
0	0	A07	0	0	kleiner LLOQ	#VALUE!	1	#VALUE!
0	0	A08	0	0	kleiner LLOQ	#VALUE!	1	#VALUE!
0	0	A09	0	0	kleiner LLOQ	#VALUE!	1	#VALUE!
0	0	A10	0	0	kleiner LLOQ	#VALUE!	1	#VALUE!
0	0	A11	0	0	kleiner LLOQ	#VALUE!	1	#VALUE!
0	0	A12	0	0	kleiner LLOQ	#VALUE!	1	#VALUE!
0	0	B01	0	0	kleiner LLOQ	#VALUE!	1	#VALUE!
0	0	B02	0	0	kleiner LLOQ	#VALUE!	1	#VALUE!
0	0	B03	0	0	kleiner LLOQ	#VALUE!	1	#VALUE!
0	0	B04	0	0	kleiner LLOQ	#VALUE!	1	#VALUE!
0	0	B05	0	0	kleiner LLOQ	#VALUE!	1	#VALUE!
0	0	B06	0	0	kleiner LLOQ	#VALUE!	1	#VALUE!
0	0	B07	0	0	kleiner LLOQ	#VALUE!	1	#VALUE!
0	0	B08	0	0	kleiner LLOQ	#VALUE!	1	#VALUE!
0	0	B09	0	0	kleiner LLOQ	#VALUE!	1	#VALUE!
0	0	B10	0	0	kleiner LLOQ	#VALUE!	1	#VALUE!
0	0	B11	0	0	kleiner LLOQ	#VALUE!	1	#VALUE!
0	0	B12	0	0	kleiner LLOQ	#VALUE!	1	#VALUE!
0	0	C01	0	0	kleiner LLOQ	#VALUE!	1	#VALUE!
0	0	C02	0	0	kleiner LLOQ	#VALUE!	1	#VALUE!
0	0	C03	0	0	kleiner LLOQ	#VALUE!	1	#VALUE!
0	0	C04	0	0	kleiner LLOQ	#VALUE!	1	#VALUE!
0	0	C05	0	0	kleiner LLOQ	#VALUE!	1	#VALUE!
0	0	C06	0	0	kleiner LLOQ	#VALUE!	1	#VALUE!
0	0	C07	0	0	kleiner LLOQ	#VALUE!	1	#VALUE!
0	0	C08	0	0	kleiner LLOQ	#VALUE!	1	#VALUE!
0	0	C09	0	0	kleiner LLOQ	#VALUE!	1	#VALUE!
0	0	C10	0	0	kleiner LLOQ	#VALUE!	1	#VALUE!
0	0	C11	0	0	kleiner LLOQ	#VALUE!	1	#VALUE!
0	0	C12	0	0	kleiner LLOQ	#VALUE!	1	#VALUE!
0	0	D01	0	0	kleiner LLOQ	#VALUE!	1	#VALUE!
0	0	D02	0	0	kleiner LLOQ	#VALUE!	1	#VALUE!
0	0	D03	0	0	kleiner LLOQ	#VALUE!	1	#VALUE!
0	0	D04	0	0	kleiner LLOQ	#VALUE!	1	#VALUE!
0	0	D05	0	0	kleiner LLOQ	#VALUE!	1	#VALUE!
0	0	D06	0	0	kleiner LLOQ	#VALUE!	1	#VALUE!
0	0	D07	0	0	kleiner LLOQ	#VALUE!	1	#VALUE!
0	0	D08	0	0	kleiner LLOQ	#VALUE!	1	#VALUE!
0	0	D09	0	0	kleiner LLOQ	#VALUE!	1	#VALUE!
0	0	D10	0	0	kleiner LLOQ	#VALUE!	1	#VALUE!
0	0	D11	0	0	kleiner LLOQ	#VALUE!	1	#VALUE!
0	0	D12	0	0	kleiner LLOQ	#VALUE!	1	#VALUE!

Plate:

	1	2	3	4	5	6	7	8	9	10	11	12
A												
B												
C												
D												
E												
F												
G												
H												

Plattenbelegung

	1	2	3	4	5	6	7	8	9	10	11	12
A												
B												
C												
D												
E												
F												
G												
H												

Comment:

Date Operator

Date Control

0	0	E01	0	0	kleiner LLOQ	#VALUE!	1	#VALUE!
0	0	E02	0	0	kleiner LLOQ	#VALUE!	1	#VALUE!
0	0	E03	0	0	kleiner LLOQ	#VALUE!	1	#VALUE!
0	0	E04	0	0	kleiner LLOQ	#VALUE!	1	#VALUE!
0	0	E05	0	0	kleiner LLOQ	#VALUE!	1	#VALUE!
0	0	E06	0	0	kleiner LLOQ	#VALUE!	1	#VALUE!
0	0	E07	0	0	kleiner LLOQ	#VALUE!	1	#VALUE!
0	0	E08	0	0	kleiner LLOQ	#VALUE!	1	#VALUE!
0	0	E09	0	0	kleiner LLOQ	#VALUE!	1	#VALUE!
0	0	E10	0	0	kleiner LLOQ	#VALUE!	1	#VALUE!
0	0	E11	0	0	kleiner LLOQ	#VALUE!	1	#VALUE!
0	0	E12	0	0	kleiner LLOQ	#VALUE!	1	#VALUE!
0	0	F01	0	0	kleiner LLOQ	#VALUE!	1	#VALUE!
0	0	F02	0	0	kleiner LLOQ	#VALUE!	1	#VALUE!
0	0	F03	0	0	kleiner LLOQ	#VALUE!	1	#VALUE!
0	0	F04	0	0	kleiner LLOQ	#VALUE!	1	#VALUE!
0	0	F05	0	0	kleiner LLOQ	#VALUE!	1	#VALUE!
0	0	F06	0	0	kleiner LLOQ	#VALUE!	1	#VALUE!
0	0	F07	0	0	kleiner LLOQ	#VALUE!	1	#VALUE!
0	0	F08	0	0	kleiner LLOQ	#VALUE!	1	#VALUE!
0	0	F09	0	0	kleiner LLOQ	#VALUE!	1	#VALUE!
0	0	F10	0	0	kleiner LLOQ	#VALUE!	1	#VALUE!
0	0	F11	0	0	kleiner LLOQ	#VALUE!	1	#VALUE!
0	0	F12	0	0	kleiner LLOQ	#VALUE!	1	#VALUE!
0	0	G01	0	0	kleiner LLOQ	#VALUE!	1	#VALUE!
0	0	G02	0	0	kleiner LLOQ	#VALUE!	1	#VALUE!
0	0	G03	0	0	kleiner LLOQ	#VALUE!	1	#VALUE!
0	0	G04	0	0	kleiner LLOQ	#VALUE!	1	#VALUE!
0	0	G05	0	0	kleiner LLOQ	#VALUE!	1	#VALUE!
0	0	G06	0	0	kleiner LLOQ	#VALUE!	1	#VALUE!
0	0	G07	0	0	kleiner LLOQ	#VALUE!	1	#VALUE!
0	0	G08	0	0	kleiner LLOQ	#VALUE!	1	#VALUE!
0	0	G09	0	0	kleiner LLOQ	#VALUE!	1	#VALUE!
0	0	G10	0	0	kleiner LLOQ	#VALUE!	1	#VALUE!
0	0	G11	0	0	kleiner LLOQ	#VALUE!	1	#VALUE!
0	0	G12	0	0	kleiner LLOQ	#VALUE!	1	#VALUE!
0	0	H01	0	0	kleiner LLOQ	#VALUE!	1	#VALUE!
0	0	H02	0	0	kleiner LLOQ	#VALUE!	1	#VALUE!
0	0	H03	0	0	kleiner LLOQ	#VALUE!	1	#VALUE!
0	0	H04	0	0	kleiner LLOQ	#VALUE!	1	#VALUE!
0	0	H05	0	0	kleiner LLOQ	#VALUE!	1	#VALUE!
0	0	H06	0	0	kleiner LLOQ	#VALUE!	1	#VALUE!
0	0	H07	0	0	kleiner LLOQ	#VALUE!	1	#VALUE!
0	0	H08	0	0	kleiner LLOQ	#VALUE!	1	#VALUE!
0	0	H09	0	0	kleiner LLOQ	#VALUE!	1	#VALUE!
0	0	H10	0	0	kleiner LLOQ	#VALUE!	1	#VALUE!
0	0	H11	0	0	kleiner LLOQ	#VALUE!	1	#VALUE!
0	0	H12	0	0	kleiner LLOQ	#VALUE!	1	#VALUE!
0	0	A01	0	0	kleiner LLOQ	#VALUE!	1	#VALUE!
0	0	A02	0	0	kleiner LLOQ	#VALUE!	1	#VALUE!
0	0	A03	0	0	kleiner LLOQ	#VALUE!	1	#VALUE!
0	0	A04	0	0	kleiner LLOQ	#VALUE!	1	#VALUE!
0	0	A05	0	0	kleiner LLOQ	#VALUE!	1	#VALUE!
0	0	A06	0	0	kleiner LLOQ	#VALUE!	1	#VALUE!
0	0	A07	0	0	kleiner LLOQ	#VALUE!	1	#VALUE!
0	0	A08	0	0	kleiner LLOQ	#VALUE!	1	#VALUE!
0	0	A09	0	0	kleiner LLOQ	#VALUE!	1	#VALUE!
0	0	A10	0	0	kleiner LLOQ	#VALUE!	1	#VALUE!
0	0	A11	0	0	kleiner LLOQ	#VALUE!	1	#VALUE!
0	0	A12	0	0	kleiner LLOQ	#VALUE!	1	#VALUE!
0	0	B01	0	0	kleiner LLOQ	#VALUE!	1	#VALUE!
0	0	B02	0	0	kleiner LLOQ	#VALUE!	1	#VALUE!
0	0	B03	0	0	kleiner LLOQ	#VALUE!	1	#VALUE!
0	0	B04	0	0	kleiner LLOQ	#VALUE!	1	#VALUE!
0	0	B05	0	0	kleiner LLOQ	#VALUE!	1	#VALUE!
0	0	B06	0	0	kleiner LLOQ	#VALUE!	1	#VALUE!
0	0	B07	0	0	kleiner LLOQ	#VALUE!	1	#VALUE!
0	0	B08	0	0	kleiner LLOQ	#VALUE!	1	#VALUE!
0	0	B09	0	0	kleiner LLOQ	#VALUE!	1	#VALUE!
0	0	B10	0	0	kleiner LLOQ	#VALUE!	1	#VALUE!
0	0	B11	0	0	kleiner LLOQ	#VALUE!	1	#VALUE!
0	0	B12	0	0	kleiner LLOQ	#VALUE!	1	#VALUE!
0	0	C01	0	0	kleiner LLOQ	#VALUE!	1	#VALUE!
0	0	C02	0	0	kleiner LLOQ	#VALUE!	1	#VALUE!
0	0	C03	0	0	kleiner LLOQ	#VALUE!	1	#VALUE!

Plate:

	1	2	3	4	5	6	7	8	9	10	11	12
A												
B												
C												
D												
E												
F												
G												
H												

Plattenbelegung

	1	2	3	4	5	6	7	8	9	10	11	12
A												
B												
C												
D												
E												
F												
G												

0	0	C04	0	0	kleiner LLOQ	#VALUE!	1	#VALUE!	H
0	0	C05	0	0	kleiner LLOQ	#VALUE!	1	#VALUE!	
0	0	C06	0	0	kleiner LLOQ	#VALUE!	1	#VALUE!	
0	0	C07	0	0	kleiner LLOQ	#VALUE!	1	#VALUE!	
0	0	C08	0	0	kleiner LLOQ	#VALUE!	1	#VALUE!	
0	0	C09	0	0	kleiner LLOQ	#VALUE!	1	#VALUE!	
0	0	C10	0	0	kleiner LLOQ	#VALUE!	1	#VALUE!	
0	0	C11	0	0	kleiner LLOQ	#VALUE!	1	#VALUE!	
0	0	C12	0	0	kleiner LLOQ	#VALUE!	1	#VALUE!	
0	0	D01	0	0	kleiner LLOQ	#VALUE!	1	#VALUE!	
0	0	D02	0	0	kleiner LLOQ	#VALUE!	1	#VALUE!	
0	0	D03	0	0	kleiner LLOQ	#VALUE!	1	#VALUE!	
0	0	D04	0	0	kleiner LLOQ	#VALUE!	1	#VALUE!	
0	0	D05	0	0	kleiner LLOQ	#VALUE!	1	#VALUE!	
0	0	D06	0	0	kleiner LLOQ	#VALUE!	1	#VALUE!	
0	0	D07	0	0	kleiner LLOQ	#VALUE!	1	#VALUE!	
0	0	D08	0	0	kleiner LLOQ	#VALUE!	1	#VALUE!	
0	0	D09	0	0	kleiner LLOQ	#VALUE!	1	#VALUE!	
0	0	D10	0	0	kleiner LLOQ	#VALUE!	1	#VALUE!	
0	0	D11	0	0	kleiner LLOQ	#VALUE!	1	#VALUE!	
0	0	D12	0	0	kleiner LLOQ	#VALUE!	1	#VALUE!	
0	0	E01	0	0	kleiner LLOQ	#VALUE!	1	#VALUE!	
0	0	E02	0	0	kleiner LLOQ	#VALUE!	1	#VALUE!	
0	0	E03	0	0	kleiner LLOQ	#VALUE!	1	#VALUE!	
0	0	E04	0	0	kleiner LLOQ	#VALUE!	1	#VALUE!	
0	0	E05	0	0	kleiner LLOQ	#VALUE!	1	#VALUE!	
0	0	E06	0	0	kleiner LLOQ	#VALUE!	1	#VALUE!	
0	0	E07	0	0	kleiner LLOQ	#VALUE!	1	#VALUE!	
0	0	E08	0	0	kleiner LLOQ	#VALUE!	1	#VALUE!	
0	0	E09	0	0	kleiner LLOQ	#VALUE!	1	#VALUE!	
0	0	E10	0	0	kleiner LLOQ	#VALUE!	1	#VALUE!	
0	0	E11	0	0	kleiner LLOQ	#VALUE!	1	#VALUE!	
0	0	E12	0	0	kleiner LLOQ	#VALUE!	1	#VALUE!	
0	0	F01	0	0	kleiner LLOQ	#VALUE!	1	#VALUE!	
0	0	F02	0	0	kleiner LLOQ	#VALUE!	1	#VALUE!	
0	0	F03	0	0	kleiner LLOQ	#VALUE!	1	#VALUE!	
0	0	F04	0	0	kleiner LLOQ	#VALUE!	1	#VALUE!	
0	0	F05	0	0	kleiner LLOQ	#VALUE!	1	#VALUE!	
0	0	F06	0	0	kleiner LLOQ	#VALUE!	1	#VALUE!	
0	0	F07	0	0	kleiner LLOQ	#VALUE!	1	#VALUE!	
0	0	F08	0	0	kleiner LLOQ	#VALUE!	1	#VALUE!	
0	0	F09	0	0	kleiner LLOQ	#VALUE!	1	#VALUE!	
0	0	F10	0	0	kleiner LLOQ	#VALUE!	1	#VALUE!	
0	0	F11	0	0	kleiner LLOQ	#VALUE!	1	#VALUE!	
0	0	F12	0	0	kleiner LLOQ	#VALUE!	1	#VALUE!	
0	0	G01	0	0	kleiner LLOQ	#VALUE!	1	#VALUE!	
0	0	G02	0	0	kleiner LLOQ	#VALUE!	1	#VALUE!	
0	0	G03	0	0	kleiner LLOQ	#VALUE!	1	#VALUE!	
0	0	G04	0	0	kleiner LLOQ	#VALUE!	1	#VALUE!	
0	0	G05	0	0	kleiner LLOQ	#VALUE!	1	#VALUE!	
0	0	G06	0	0	kleiner LLOQ	#VALUE!	1	#VALUE!	
0	0	G07	0	0	kleiner LLOQ	#VALUE!	1	#VALUE!	
0	0	G08	0	0	kleiner LLOQ	#VALUE!	1	#VALUE!	
0	0	G09	0	0	kleiner LLOQ	#VALUE!	1	#VALUE!	
0	0	G10	0	0	kleiner LLOQ	#VALUE!	1	#VALUE!	
0	0	G11	0	0	kleiner LLOQ	#VALUE!	1	#VALUE!	
0	0	G12	0	0	kleiner LLOQ	#VALUE!	1	#VALUE!	
0	0	H01	0	0	kleiner LLOQ	#VALUE!	1	#VALUE!	
0	0	H02	0	0	kleiner LLOQ	#VALUE!	1	#VALUE!	
0	0	H03	0	0	kleiner LLOQ	#VALUE!	1	#VALUE!	
0	0	H04	0	0	kleiner LLOQ	#VALUE!	1	#VALUE!	
0	0	H05	0	0	kleiner LLOQ	#VALUE!	1	#VALUE!	
0	0	H06	0	0	kleiner LLOQ	#VALUE!	1	#VALUE!	
0	0	H07	0	0	kleiner LLOQ	#VALUE!	1	#VALUE!	
0	0	H08	0	0	kleiner LLOQ	#VALUE!	1	#VALUE!	
0	0	H09	0	0	kleiner LLOQ	#VALUE!	1	#VALUE!	
0	0	H10	0	0	kleiner LLOQ	#VALUE!	1	#VALUE!	
0	0	H11	0	0	kleiner LLOQ	#VALUE!	1	#VALUE!	
0	0	H12	0	0	kleiner LLOQ	#VALUE!	1	#VALUE!	

Comment:

Date Operator

Date Control

1.447	1.739		1.170						0.667	0.440	0.043	1
1.447	1.332		1.392	0.137	0.146	0.130	0.138	0.109	0.729	0.478	0.042	
1.391	1.281		1.311	0.204	0.184	0.184	0.198	0.201	0.743	0.474	0.043	
0.961	0.835		1.405	0.336	0.347	0.375	0.387	0.300	0.824	0.483	0.044	
0.445	0.441		1.280	1.085	1.086	1.218	1.004	0.873	0.704	0.457	0.041	
0.355	0.315		1.332	1.768	1.925	1.753	1.739	1.606			0.036	
0.223	0.188	1.666	1.499	1.632		0.048	0.046	0.047	0.048	0.048	0.048	
0.118	0.106	1.556	1.622	1.573		0.047	0.054	0.051	0.051	0.052	0.051	

1.652	1.627		1.188	0.748	0.811	0.764	0.753	0.674	0.751	0.529	0.044	2
1.443	1.372		1.364	0.139	0.147	0.131	0.139	0.110	0.811	0.561	0.043	
1.377	1.281		1.284	0.206	0.189	0.184	0.200	0.202	0.821	0.560	0.044	
0.972	0.883		1.387	0.339	0.349	0.386	0.406	0.312	0.903	0.565	0.044	
0.484	0.474		1.298	1.087	1.113	1.203	1.022	0.903	0.790	0.544	0.042	
0.359	0.326		1.308	1.622	1.622	1.630	1.625	1.550			0.037	
0.228	0.199	1.527	1.373	1.462		0.048	0.046	0.048	0.049	0.049	0.049	
0.125	0.109	1.473	1.410	1.371		0.048	0.054	0.052	0.052	0.052	0.051	

1.579	1.475		1.150	1.210	1.303	1.202	1.219	1.125	0.759	0.549	0.043	3
1.357	1.303		1.283	0.140	0.148	0.131	0.139	0.111	0.820	0.575	0.043	
1.323	1.228		1.227	0.206	0.188	0.184	0.198	0.201	0.824	0.579	0.043	
0.959	0.872		1.321	0.339	0.348	0.387	0.406	0.314	0.909	0.580	0.044	
0.483	0.473		1.270	1.069	1.103	1.181	1.017	0.903	0.798	0.560	0.042	
0.360	0.326		1.270	1.497	1.448	1.496	1.494	1.449			0.037	
0.228	0.199	1.374	1.262	1.314		0.048	0.046	0.048	0.049	0.049	0.049	
0.125	0.110	1.346	1.262	1.253		0.048	0.054	0.052	0.052	0.052	0.051	

1.511	1.369			1.538	1.712	1.528	1.502	1.425			0.043	4
1.296	1.261										0.043	
1.272	1.193										0.043	
0.947	0.859										0.044	
0.481	0.471										0.042	
0.361	0.325										0.037	
0.229	0.199					0.048	0.046	0.048	0.049	0.049	0.049	
0.125	0.110					0.047	0.054	0.052	0.052	0.052	0.051	

1.493	1.317	1.678	1.110	1.621	1.807	1.618	1.584	1.508	0.765	0.597	0.043	5
1.259	1.238	1.850	1.197	0.139	0.150	0.132	0.139	0.111	0.821	0.588	0.043	
1.248	1.175	1.816	1.153	0.204	0.188	0.184	0.197	0.199	0.831	0.599	0.044	
0.943	0.859	1.970	1.231	0.338	0.357	0.386	0.406	0.316	0.916	0.595	0.044	
0.480	0.470	2.220	1.220	1.054	1.088	1.154	1.006	0.902	0.805	0.579	0.042	
0.359	0.325	2.149	1.211	1.395	1.324	1.348	1.345	1.317	0.037	0.037	0.037	
0.228	0.199	1.240	1.151	1.183	0.034	0.049	0.046	0.048	0.049	0.049	0.049	
0.125	0.110	1.223	1.132	1.146	0.038	0.048	0.054	0.052	0.052	0.052	0.051	

1.434	1.258		1.090	1.648	1.876	1.649	1.627	1.535				6
1.217	1.201		1.149									
1.205	1.143		1.109									
0.935	0.845		1.182									
0.477	0.469		1.181									
0.360	0.323		1.167									
0.229	0.200					0.049	0.047	0.048	0.049	0.049	0.049	
0.125	0.110					0.048	0.054	0.052	0.052	0.052	0.051	

1.013	0.810		0.668	0.808	0.940	0.720	0.798	0.687	0.904	0.845	0.043	7
0.709	0.827		0.703	0.142	0.156	0.136	0.137	0.116	0.990	0.846	0.042	
0.682	0.692		0.680	0.197	0.188	0.190	0.191	0.184	1.023	0.861	0.043	
0.507	0.519		0.740	0.316	0.264	0.364	0.377	0.312	1.111	0.870	0.043	
0.394	0.406		0.682	0.580	0.561	0.600	0.529	0.563	0.962	0.834	0.042	
0.333	0.322		0.661	0.995	0.930	0.848	0.832	0.797	0.037	0.037	0.037	
0.229	0.201	0.843	0.679	0.732		0.048	0.046	0.047			0.047	
0.124	0.111	0.773	0.682	0.701		0.047	0.053	0.051	0.051	0.052	0.051	

Kontrollen

3.126												1
1.664												
3.141												
						0.046	0.047	0.048	0.049	0.048	0.048	
						0.047	0.046	0.048	0.048	0.046	0.052	

	2.229	2.249	2.221	0.105	0.106	0.109						2
	0.897	0.909	0.928	0.088	0.089	0.087						
	1.755	1.748	1.733	0.186	0.184	0.182						
						0.046	0.046	0.049	0.050	0.048	0.050	
						0.048	0.047	0.048	0.047	0.046	0.050	

												3
	1.040	1.053	1.067	0.077	0.076	0.073						
	1.334	1.265	1.290	0.117	0.116	0.115						
						0.045	0.047	0.049	0.050	0.048	0.050	
						0.048	0.046	0.050	0.048	0.047	0.049	
