# Supplementary material for: Establishment of an in vitro model of cultured viable human, porcine and canine skin and comparison of different media supplements
Source: PeerJ. 2019 Oct 3;7:e7811. doi: 10.7717/peerj.7811 (PMC6778665; doi:10.7717/peerj.7811)
Supplement: Supplemental Information 2 — Evaluation of absolute and relative amount LDH released, species difference in viability throughout the cultivatio [file peerj-07-7811-s002.pdf]

OriginPro 9 32-bit - N:\5_Labor\5_1_Auftraege\5_1_1_laufende_Auftraege\F_120_ZIM_Topas\IsBa\Publikation\Paper 3\Origin_statistics * - /Folder1/ - [Book1]

Kruskal-Wallis ANOVA (17.04.2019 15:29:40)

Notes

X-Function	Kruskal-Wallis ANOVA
User Name	i.bauhammer
Time	17.04.2019 15:29:40

Input Data

	Data	Range
sui	[Book1]Sheet1!B"s	[2:4]
hd	[Book1]Sheet1!C"h	[2:4]

Descriptive Statistics

	N	Min	Q1	Median	Q3	Max
B	3	47.72	47.72	50.63	54.85	54.85
C	3	64.93	64.93	65.43	67.83	67.83

Ranks

	N	Mean Rank	Sum Rank
B	3	2	6
C	3	5	15

Test Statistics

Chi-Square	DF	Prob>Chi-Square
3.85714	1	0.04953

Null Hypothesis:The samples come from the same population.
Alternative Hypothesis:The samples come from different populations.
: At the 0.05 level, the populations are significantly different.

Sheet1 | KWANOVA1 | KWANOVA2 | KWANOVA3 | NPHKSamples1 | NPHKSamples2 | NPHKSamples3 | NPHKSamples5 | NPHKSamples6 | NPHKSamples4 | NPHKSamples7 | NPHKSamples8 | NPHKSamp

For Help, press F1 Average=0 Sum=0 Count=0 AU : ON 2: [Book1]KWANOVA1! Radian

OriginPro 9 32-bit - N:\5_Labor\5_1_Auftraege\5_1_1_laufende_Auftraege\F_120_ZIM_Topas\IsBa\Publikation\Paper 3\Origin_statistics * - /Folder1/ - [Book1]

File Edit View Plot Column Worksheet Analysis Statistics Image Tools Format Window Help

Kruskal-Wallis ANOVA (17.04.2019 15:31:03)

Notes

X-Function	Kruskal-Wallis ANOVA
User Name	i.bauhammer
Time	17.04.2019 15:31:03

Input Data

	Data	Range
sui	[Book1]Sheet1!B"s	[2:4]
hd	[Book1]Sheet1!C"h	[2:4]
hu	[Book1]Sheet1!D"h	[2:4]

Descriptive Statistics

	N	Min	Q1	Median	Q3	Max
B	3	47.72	47.72	50.63	54.85	54.85
C	3	64.93	64.93	65.43	67.83	67.83
D	3	51.9	51.9	52.05	55.12	55.12

Ranks

	N	Mean Rank	Sum Rank
B	3	2.66667	8
C	3	8	24
D	3	4.33333	13

Test Statistics

Chi-Square	DF	Prob>Chi-Square
5.95556	2	0.05091

Null Hypothesis:The samples come from the same population.
Alternative Hypothesis:The samples come from different populations.
: At the 0.05 level, the populations are NOT significantly different.

Sheet1 | KWANOVA1 | KWANOVA2 | KWANOVA3 | NPHKSamples1 | NPHKSamples2 | NPHKSamples3 | NPHKSamples5 | NPHKSamples6 | NPHKSamples4 | NPHKSamples7 | NPHKSamples8 | NPHKSam

For Help, press F1 | Average=0 Sum=0 Count=0 AU : ON | 3: [Book1]KWANOVA2! Radian

Start | OriginPro 9 32-bit - N... | 1.JPG - Paint | DE

OriginPro 9 32-bit - N:\5_Labor\5_1_Auftraege\5_1_1_laufende_Auftraege\F_120_ZIM_Topas\IsBa\Publikation\Paper 3\Origin_statistics * - /Folder1/ - [Book1]

File Edit View Plot Column Worksheet Analysis Statistics Image Tools Format Window Help

Kruskal-Wallis ANOVA (17.04.2019 15:33:00)

Notes

X-Function	Kruskal-Wallis ANOVA
User Name	i.bauhammer
Time	17.04.2019 15:33:00

Input Data

	Data	Range
hd	[Book1]Sheet1!C"hd"	[2:4]
hum	[Book1]Sheet1!D"hum"	[2:4]

Descriptive Statistics

	N	Min	Q1	Median	Q3	Max
"hd"	3	64.93	64.93	65.43	67.83	67.83
"hum"	3	51.9	51.9	52.05	55.12	55.12

Ranks

	N	Mean Rank	Sum Rank
"hd"	3	5	15
"hum"	3	2	6

Test Statistics

Chi-Square	DF	Prob>Chi-Square
3.85714	1	0.04953

Null Hypothesis:The samples come from the same population.
Alternative Hypothesis:The samples come from different populations.
: At the 0.05 level, the populations are significantly different.

Sheet1 | KWANOVA1 | KWANOVA2 | KWANOVA3 | NPHKSamples1 | NPHKSamples2 | NPHKSamples3 | NPHKSamples5 | NPHKSamples6 | NPHKSamples4 | NPHKSamples7 | NPHKSamples8 | NPHKSam

For Help, press F1 Average=0 Sum=0 Count=0 AU : ON 4: [Book1]KWANOVA3! Radian

OriginPro 9 32-bit - N:\5_Labor\5_1_Auftraege\5_1_1_laufende_Auftraege\F_120_ZIM_Topas\IsBa\Publikation\Paper 3\Origin_statistics * - /Folder1/ - [Book1]

File Edit View Plot Column Worksheet Analysis Statistics Image Tools Format Window Help

Nonparametric Analysis (K Sample) (17.04.2019 15:43:45)

Notes

X-Function	Nonparametric Analysis (K Sample)
User Name	i.bauhammer
Time	17.04.2019 15:43:45

Input Data

	Data	Range
sui	[Book1]Sheet1!B"sui"	[2:4]
hd	[Book1]Sheet1!C"hd"	[2:4]
hum	[Book1]Sheet1!D"hum"	[2:4]

Descriptive Statistics

	N	Min	Q1	Median	Q3	Max
"sui"	3	47.72	47.72	50.63	54.85	54.85
"hd"	3	64.93	64.93	65.43	67.83	67.83
"hum"	3	51.9	51.9	52.05	55.12	55.12

Kruskal-Wallis ANOVA test

Ranks

	N	Mean Rank	Sum Rank
"sui"	3	2.66667	8
"hd"	3	8	24
"hum"	3	4.33333	13

Test Statistics

Chi-Square	DF	Prob>Chi-Square
5.95556	2	0.05091

Null Hypothesis:The samples come from the same population.
Alternative Hypothesis:The samples come from different populations.
: At the 0.05 level, the populations are NOT significantly different.

Median test

Frequencies

	N	>Median	<=Median
"sui"	3	0	3
"hd"	3	3	0
"hum"	3	1	2

Test Statistics

Median	Chi-Square	DF	Prob>Chi-Square
54.85	6.3	2	0.04285

Null Hypothesis:The samples come from the same population.
Alternative Hypothesis:The samples come from different populations.
: At the 0.05 level, the populations are significantly different.

Sheet1 | KWANOVA1 | KWANOVA2 | KWANOVA3 | NPHKSamples1 | NPHKSamples2 | NPHKSamples3 | NPHKSamples5 | NPHKSamples6 | NPHKSamples4 | NPHKSamples7 | NPHKSamples8 | NPHKSamp

For Help, press F1 Average=0 Sum=0 Count=0 AU : ON 5: [Book1]NPHKSamples1! Radian

Start OriginPro 9 32-bit - N... 3.JPG - Paint DE

OriginPro 9 32-bit - N:\5_Labor\5_1_Auftraege\5_1_1_laufende_Auftraege\F_120_ZIM_Topas\IsBa\Publikation\Paper 3\Origin_statistics * - /Folder1/ - [Book1]

File Edit View Plot Column Worksheet Analysis Statistics Image Tools Format Window Help

1 Nonparametric Analysis (K Sample) (17.04.2019 15:45:01)

Notes

X-Function	Nonparametric Analysis (K Sample)
User Name	i.bauhammer
Time	17.04.2019 15:45:01

Input Data

	Data	Range
sui	[Book1]Sheet1!B"sui"	[2:4]
hd	[Book1]Sheet1!C"hd"	[2:4]

Descriptive Statistics

	N	Min	Q1	Median	Q3	Max
"sui"	3	47.72	47.72	50.63	54.85	54.85
"hd"	3	64.93	64.93	65.43	67.83	67.83

Kruskal-Wallis ANOVA test

Ranks

	N	Mean Rank	Sum Rank
"sui"	3	2	6
"hd"	3	5	15

Test Statistics

Chi-Square	DF	Prob>Chi-Square
3.85714	1	0.04953

Null Hypothesis:The samples come from the same population.
Alternative Hypothesis:The samples come from different populations.
: At the 0.05 level, the populations are significantly different.

Median test

Frequencies

	N	>Median	<=Median
"sui"	3	0	3
"hd"	3	3	0

Test Statistics

Median	Chi-Square	DF	Prob>Chi-Square
59.89	6	1	0.01431

Null Hypothesis:The samples come from the same population.
Alternative Hypothesis:The samples come from different populations.
: At the 0.05 level, the populations are significantly different.

Sheet1 | KWANOVA1 | KWANOVA2 | KWANOVA3 | NPHKSamples1 | **NPHKSamples2** | NPHKSamples3 | NPHKSamples5 | NPHKSamples6 | NPHKSamples4 | NPHKSamples7 | NPHKSamples8 | NPHKSam[illegible]

For Help, press F1 — Average=0 Sum=0 Count=0 AU : ON — 6: [Book1]NPHKSamples2! Radian

Start — OriginPro 9 32-bit - N... — 4.JPG - Paint — DE

OriginPro 9 32-bit - N:\5_Labor\5_1_Auftraege\5_1_1_laufende_Auftraege\F_120_ZIM_Topas\IsBa\Publikation\Paper 3\Origin_statistics * - /Folder1/ - [Book1]

File Edit View Plot Column Worksheet Analysis Statistics Image Tools Format Window Help

Nonparametric Analysis (K Sample) (17.04.2019 16:34:50)

Notes

X-Function	Nonparametric Analysis (K Sample)
User Name	i.bauhammer
Time	17.04.2019 16:34:50

Input Data

	Data	Range
hum	[Book1]Sheet1!H"hum"	[1:3]
sui	[Book1]Sheet1!I"sui"	[1:3]
hd	[Book1]Sheet1!J"hd"	[1:3]

Descriptive Statistics

	N	Min	Q1	Median	Q3	Max
"hum"	3	73.19	73.19	78.2	78.43	78.43
"sui"	3	56.36	56.36	61.63	65.26	65.26
"hd"	3	18.43	18.43	19.8	20.1	20.1

Kruskal-Wallis ANOVA test

Ranks

	N	Mean Rank	Sum Rank
"hum"	3	8	24
"sui"	3	5	15
"hd"	3	2	6

Test Statistics

Chi-Square	DF	Prob>Chi-Square
7.2	2	0.02732

Null Hypothesis:The samples come from the same population.
Alternative Hypothesis:The samples come from different populations.
: At the 0.05 level, the populations are significantly different.

Median test

Frequencies

	N	>Median	<=Median
"hum"	3	3	0
"sui"	3	1	2
"hd"	3	0	3

Test Statistics

Median	Chi-Square	DF	Prob>Chi-Square
61.63	6.3	2	0.04285

Null Hypothesis:The samples come from the same population.
Alternative Hypothesis:The samples come from different populations.
: At the 0.05 level, the populations are significantly different.

Sheet1 | KWANOVA1 | KWANOVA2 | KWANOVA3 | NPHKSamples1 | NPHKSamples2 | **NPHKSamples3** | NPHKSamples5 | NPHKSamples6 | NPHKSamples4 | NPHKSamples7 | NPHKSamples8 | NPHKSamp

For Help, press F1 | Average=0 Sum=0 Count=0 | AU : ON | 7: [Book1]NPHKSamples3! | Radian

Start | OriginPro 9 32-bit - N... | 5.JPG - Paint | DE

OriginPro 9 32-bit - N:\5_Labor\5_1_Auftraege\5_1_1_laufende_Auftraege\F_120_ZIM_Topas\IsBa\Publikation\Paper 3\Origin_statistics * - /Folder1/ - [Book1]

File Edit View Plot Column Worksheet Analysis Statistics Image Tools Format Window Help

Nonparametric Analysis (K Sample) (17.04.2019 16:38:43)

Notes

X-Function	Nonparametric Analysis (K Sample)
User Name	i.bauhammer
Time	17.04.2019 16:38:43

Input Data

	Data	Range
hum r	[Book1]Sheet1!K"hum r"	[1:6]
sui r	[Book1]Sheet1!L"sui r"	[1:6]
hd r	[Book1]Sheet1!M"hd r"	[1:6]

Descriptive Statistics

	N	Min	Q1	Median	Q3	Max
"hum r"	6	44.88	44.88	47.95	48.95	48.95
"sui r"	6	45.15	45.15	49.37	52.28	52.28
"hd r"	6	32.17	32.17	34.57	35.07	35.07

Kruskal-Wallis ANOVA test

Ranks

	N	Mean Rank	Sum Rank
"hum r"	6	10.83333	65
"sui r"	6	14.16667	85
"hd r"	6	3.5	21

Test Statistics

Chi-Square	DF	Prob>Chi-Square
12.65556	2	0.00179

Null Hypothesis:The samples come from the same population.
Alternative Hypothesis:The samples come from different populations.
: At the 0.05 level, the populations are significantly different.

Median test

Frequencies

	N	>Median	<=Median
"hum r"	6	4	2
"sui r"	6	4	2
"hd r"	6	0	6

Test Statistics

Median	Chi-Square	DF	Prob>Chi-Square
45.15	7.2	2	0.02732

Null Hypothesis:The samples come from the same population.
Alternative Hypothesis:The samples come from different populations.
: At the 0.05 level, the populations are significantly different.

Sheet1 | KWANOVA1 | KWANOVA2 | KWANOVA3 | NPHKSamples1 | NPHKSamples2 | NPHKSamples3 | NPHKSamples5 | NPHKSamples6 | NPHKSamples4 | NPHKSamples7 | NPHKSamples8 | NPHKSamp

For Help, press F1 Average=0 Sum=0 Count=0 AU : ON 8: [Book1]NPHKSamples5! Radian

Start OriginPro 9 32-bit - N... 6.JPG - Paint DE

OriginPro 9 32-bit - N:\5_Labor\5_1_Auftraege\5_1_1_laufende_Auftraege\F_120_ZIM_Topas\IsBa\Publikation\Paper 3\Origin_statistics * - /Folder1/ - [Book1]

File Edit View Plot Column Worksheet Analysis Statistics Image Tools Format Window Help

Nonparametric Analysis (K Sample) (17.04.2019 16:59:18)

Notes

X-Function	Nonparametric Analysis (K Sample)
User Name	i.bauhammer
Time	17.04.2019 16:59:18

Input Data

	Data	Range
hd	[Book1]Sheet1!N"hd"	[1:1]
hum	[Book1]Sheet1!O"hum"	[1:1]
sui	[Book1]Sheet1!P"sui"	[1:1]

Descriptive Statistics

	N	Min	Q1	Median	Q3	Max
"hd"	1	83.26	83.26	83.26	83.26	83.26
"hum"	1	73.76	73.76	73.76	73.76	73.76
"sui"	1	67.46	67.46	67.46	67.46	67.46

Kruskal-Wallis ANOVA test

Ranks

	N	Mean Rank	Sum Rank
"hd"	1	3	3
"hum"	1	2	2
"sui"	1	1	1

Test Statistics

Chi-Square	DF	Prob>Chi-Square
2	2	0.36788

Null Hypothesis:The samples come from the same population.
Alternative Hypothesis:The samples come from different populations.
: At the 0.05 level, the populations are NOT significantly different.

Median test

Frequencies

	N	>Median	<=Median
"hd"	1	1	0
"hum"	1	0	1
"sui"	1	0	1

Test Statistics

Median	Chi-Square	DF	Prob>Chi-Square
73.76	3	2	0.22313

Null Hypothesis:The samples come from the same population.
Alternative Hypothesis:The samples come from different populations.
: At the 0.05 level, the populations are NOT significantly different.

Sheet1 | KWANOVA1 | KWANOVA2 | KWANOVA3 | NPHKSamples1 | NPHKSamples2 | NPHKSamples3 | NPHKSamples5 | NPHKSamples6 | NPHKSamples4 | **NPHKSamples7** | NPHKSamples8 | NPHKSamp

For Help, press F1 Average=0 Sum=0 Count=0 AU : ON 11: [Book1]NPHKSamples7! Radian

Start OriginPro 9 32-bit - N... 9.JPG - Paint DE

Nonparametric Analysis (K Sample) (17.04.2019 17:00:50)

Notes

X-Function	Nonparametric Analysis (K Sample)
User Name	i.bauhammer
Time	17.04.2019 17:00:50

Input Data

	Data	Range
hd	[Book1]Sheet1!N"hd"	[1:3]
hum	[Book1]Sheet1!O"hum"	[1:3]
sui	[Book1]Sheet1!P"sui"	[1:3]

Descriptive Statistics

	N	Min	Q1	Median	Q3	Max
"hd"	3	83.26	83.26	83.26	83.26	83.26
"hum"	3	73.76	73.76	73.76	73.76	73.76
"sui"	3	67.46	67.46	67.46	67.46	67.46

Kruskal-Wallis ANOVA test

Ranks

	N	Mean Rank	Sum Rank
"hd"	3	8	24
"hum"	3	5	15
"sui"	3	2	6

Test Statistics

Chi-Square	DF	Prob>Chi-Square
8	2	0.01832

Null Hypothesis:The samples come from the same population.
Alternative Hypothesis:The samples come from different populations.
: At the 0.05 level, the populations are significantly different.

Median test

Frequencies

	N	>Median	<=Median
"hd"	3	3	0
"hum"	3	0	3
"sui"	3	0	3

Test Statistics

Median	Chi-Square	DF	Prob>Chi-Square
73.76	9	2	0.01111

Null Hypothesis:The samples come from the same population.
Alternative Hypothesis:The samples come from different populations.
: At the 0.05 level, the populations are significantly different.

OriginPro 9 32-bit - N:\5_Labor\5_1_Auftraege\5_1_1_laufende_Auftraege\F_120_ZIM_Topas\IsBa\Publikation\Paper 3\Origin_statistics * - /Folder1/ - [Book1]

File Edit View Plot Column Worksheet Analysis Statistics Image Tools Format Window Help

1 Nonparametric Analysis (K Sample) (17.04.2019 17:01:56)

Notes

X-Function	Nonparametric Analysis (K Sample)
User Name	i.bauhammer
Time	17.04.2019 17:01:56

Input Data

	Data	Range
hd	[Book1]Sheet1!N"hd"	[15:17]
hum	[Book1]Sheet1!O"hum"	[15:17]
sui	[Book1]Sheet1!P"sui"	[15:17]

Descriptive Statistics

	N	Min	Q1	Median	Q3	Max
"hd"	3	67.7	67.7	67.7	67.7	67.7
"hum"	3	54.18	54.18	54.18	54.18	54.18
"sui"	3	52.13	52.13	52.13	52.13	52.13

Kruskal-Wallis ANOVA test

Ranks

	N	Mean Rank	Sum Rank
"hd"	3	8	24
"hum"	3	5	15
"sui"	3	2	6

Test Statistics

Chi-Square	DF	Prob>Chi-Square
8	2	0.01832

Null Hypothesis:The samples come from the same population.
Alternative Hypothesis:The samples come from different populations.
: At the 0.05 level, the populations are significantly different.

Median test

Frequencies

	N	>Median	<=Median
"hd"	3	3	0
"hum"	3	0	3
"sui"	3	0	3

Test Statistics

Median	Chi-Square	DF	Prob>Chi-Square
54.18	9	2	0.01111

Null Hypothesis:The samples come from the same population.
Alternative Hypothesis:The samples come from different populations.
: At the 0.05 level, the populations are significantly different.

KWANOVA1 KWANOVA2 KWANOVA3 NPHKSamples1 NPHKSamples2 NPHKSamples3 NPHKSamples5 NPHKSamples6 NPHKSamples4 NPHKSamples7 NPHKSamples8 **NPHKSamples9** NF

For Help, press F1 Average=0 Sum=0 Count=0 AU : ON 13: [Book1]NPHKSamples9! Radian

Start OriginPro 9 32-bit - N... 11.JPG - Paint DE
