# Supplementary figures and images for: Establishment of an in vitro model of cultured viable human, porcine and canine skin and comparison of different media supplements
Source: PeerJ. 2019 Oct 3;7:e7811. doi: 10.7717/peerj.7811 (PMC6778665; doi:10.7717/peerj.7811)

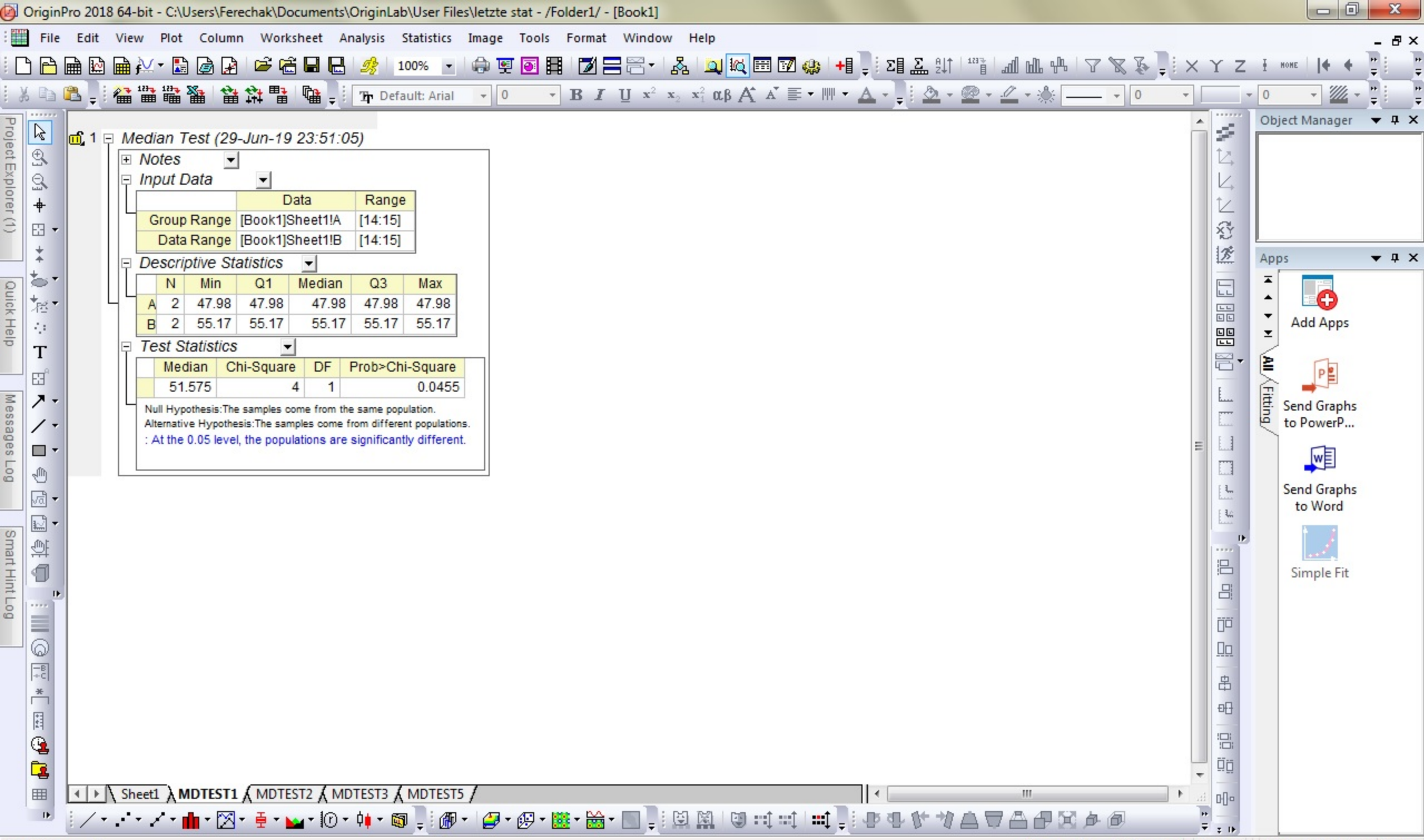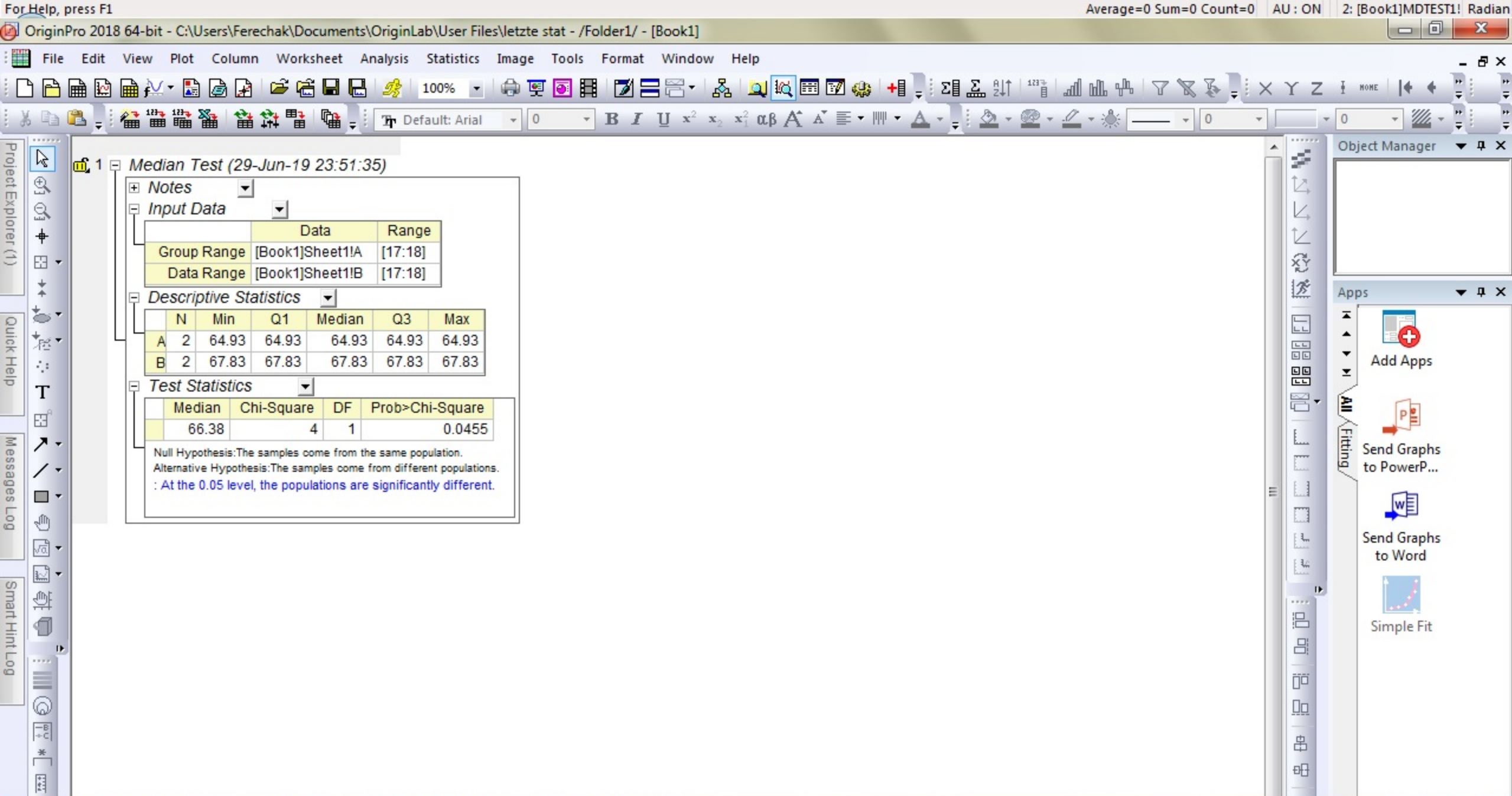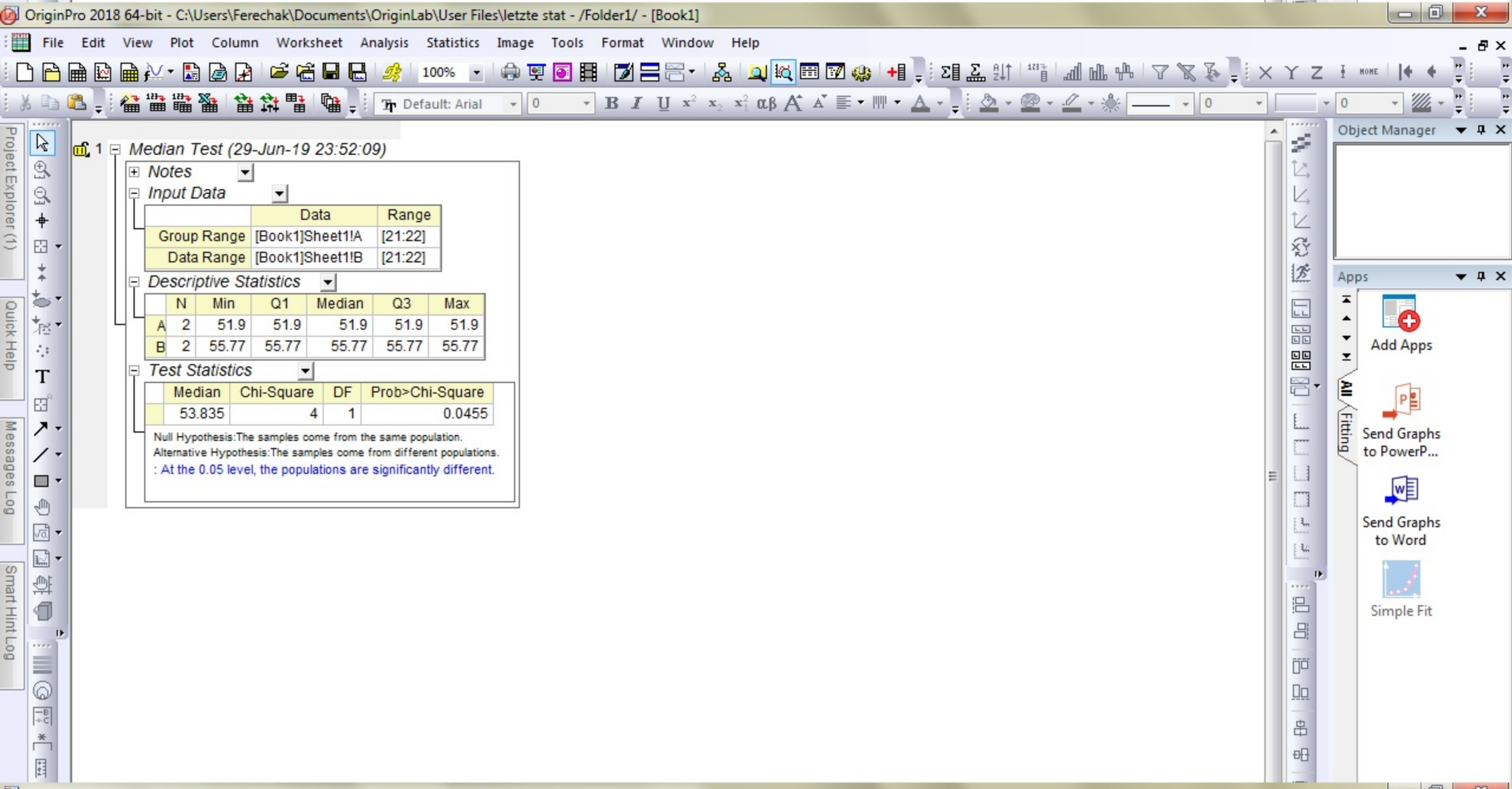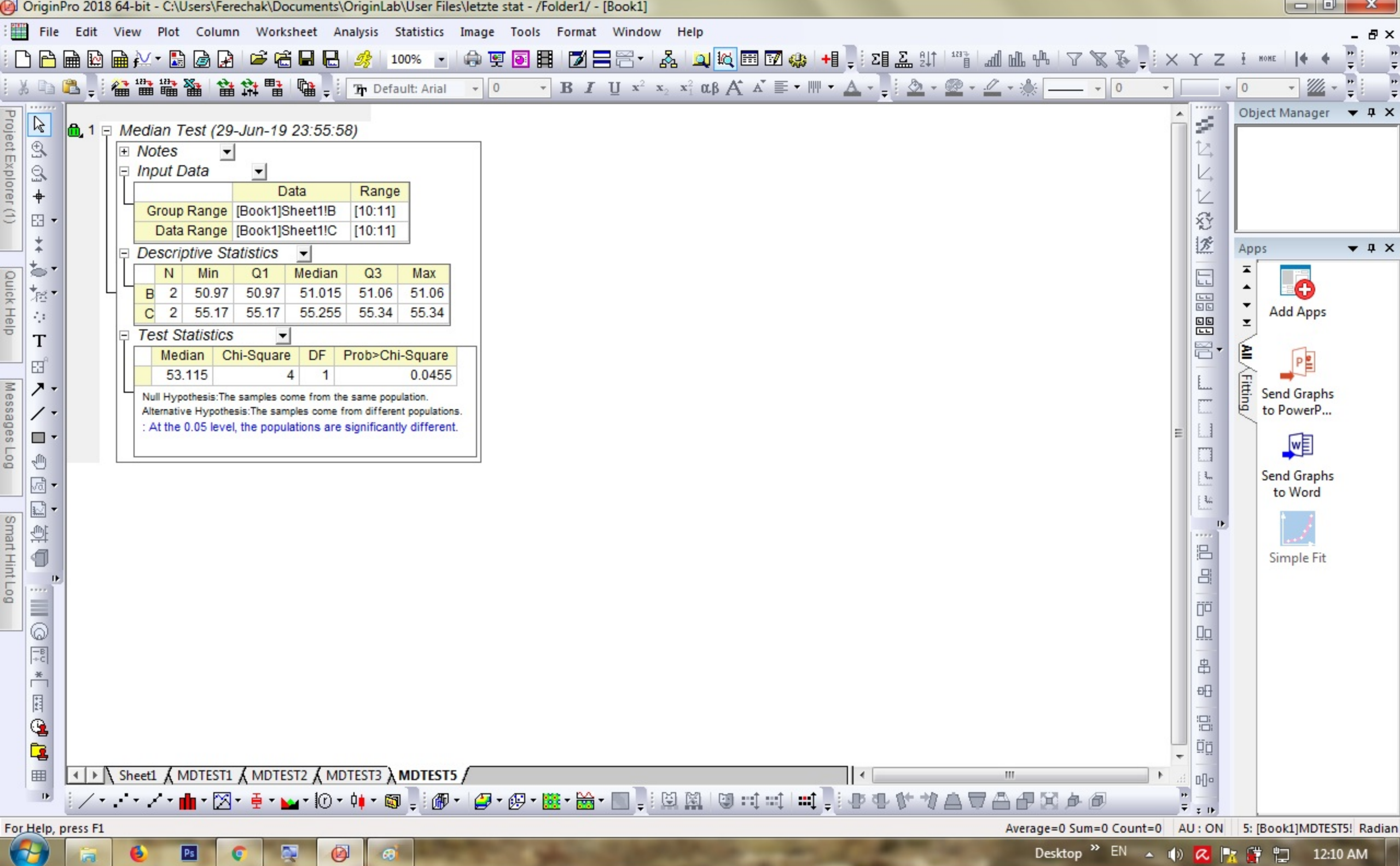

Supplement: Supplemental Information 3 [file peerj-07-7811-s003.pdf]
