# Supplementary material for: Establishment of an in vitro model of cultured viable human, porcine and canine skin and comparison of different media supplements
Source: PeerJ. 2019 Oct 3;7:e7811. doi: 10.7717/peerj.7811 (PMC6778665; doi:10.7717/peerj.7811)
Supplement: Supplemental Information 5 — The protocols in the supplementary files containing German references are locked and cannot be changed as they are validated for the Lab. Relevant German references and evaluation comments have been translated to English in this list to facilitate the understanding of these files. [file peerj-07-7811-s005.pdf]

Word list German-English (green=english)

File: **Evaluation skin cultivation with lin high and lin low (chronologic order, exemplary first three pages)**

- Messdaten – measured data
- Tabelle – table
- Bericht – report
- Vetrauensbereich Steig. (95%) Obergrenze/Untergrenze – confidence interval (95%) upper/lower limit
- Vertrauensbereich Achsenabschnitt (95%) Obergrenze/Untergrenze – confidence interval axis intercept (95%) upper/lower limit
- Summe Restquadrate – sum of residual squares
- Verfahrensstandardabweichung – process standard deviation
- Kleiner LLOQ – smaller than the lower limit
- Verdünnung – dilution
- Gelb – yellow
- Rot – red

Remark: samples aufgeteilt in lin high und lin low: höher konzentrierte samples sind bei lin high zu finden =

->samples are divided into linearity high and low. Higher concentrated samples can be found therefore in lin high.

Comment field: (first comment, exemplary, only difference is dilution factors)

Erste vier Reihen immer Testmessung zur Evaluierung der benötigten Verdünnung. Wird nicht mit ausgewertet. Unverdünnte Messungen außerhalb der Linearität werden ebenfalls nicht ausgewertet. Dil Faktor unverdünnte samples 2.75 (100µl sample+100µl reaction mix+75µl stop solution). Verdünnung (gelb) Dil.8*Dil.8*Dil.2.75=Dil. 176 =

->First 4 rows always test measurements for the assessment of the needed dilution. Will not be evaluated. Undiluted measurements outside of the linearity will not be evaluated either. Dilution factor for undiluted samples = 2.75 (100µl sample+100µl reaction mix+75µl stop solution). Dilution (yellow) Dil.8*Dil.8*Dil.2.75=Dil. 176

File: **Summary skin viability V01 (last page summary part)**

- Schwein – porcine
- Hund – canine
- Nativ – basic (medium)
- Kumulativ Sum. – cumulated sum
- Positivkontrolle – positive control
